# Chronic pain and associated factors in remote work during the COVID-19 pandemic in Brazil

**DOI:** 10.1590/0034-7167-2023-0012

**Published:** 2023-12-04

**Authors:** Brenda Alves Silvestre, Luiz Paulo Miotto, Karina Gramani-Say, Maria Helena Barbosa, Priscilla Hortense

**Affiliations:** I Universidade Federal de São Carlos. São Carlos, São Paulo, Brazil; II Universidade Federal do Triângulo Mineiro. Uberaba, Minas Gerais, Brazil

**Keywords:** Chronic Pain, Sleep Quality, Anxiety, Teleworking, COVID-19, Dolor Crónico, Calidad del Sueño, Ansiedad, Teletrabajo, COVID-19, Dor Crônica, Qualidade do Sono, Ansiedade, Teletrabalho, COVID-19

## Abstract

**Objective:**

Estimate the prevalence of chronic pain and its association with symptoms of anxiety, sleep disorders, and aspects of remote work in the context of the COVID-19 pandemic.

**Method:**

A cross-sectional and descriptive study conducted with 328 adults engaged in remote work. Data was collected online from February 2021 to January 2022. For pain investigation and evaluations of sleep and anxiety, a structured questionnaire, the Pittsburgh Sleep Quality Index, and the Generalized Anxiety Disorder-7 were used, respectively.

**Results:**

The prevalence of chronic pain was 47.9% (CI 95% = 42.5-53.3). Associations were identified between pain and anxiety, sleep disorders, and sitting time (p<0.01).

**Conclusion:**

The prevalence of chronic pain in remote work was found to be high, with pain being of moderate intensity and associated with anxiety, sleep disorders, and prolonged sitting time.

## RESUMO

**Objetivo:**

Estimar a prevalência de dor crônica e sua associação com sintomas de ansiedade, distúrbios do sono e aspectos do trabalho remoto no contexto da pandemia da COVID-19.

**Método:**

Estudo transversal e descritivo realizado com 328 adultos em teletrabalho. Os dados foram coletados *online* de fevereiro de 2021 a janeiro de 2022. Para a investigação da dor e avaliações do sono e da ansiedade, foram utilizados um questionário estruturado, o Questionário Índice de Qualidade do Sono de Pittsburgh e o *Generalized Anxiety Disorder-7,* respectivamente.

**Resultados:**

A prevalência de dor crônica foi de 47,9% (IC 95% = 42,5-53,3). Foram identificadas associações entre a dor e a ansiedade, distúrbios do sono e tempo sentado (p<0,01).

**Conclusão:**

A prevalência de dor crônica no teletrabalho mostrou-se elevada, com a dor sendo de intensidade moderada e associada à ansiedade, aos distúrbios do sono e ao tempo passado sentado.

## RESUMEN

**Objetivo:**

Estimar la prevalencia del dolor crónico y su asociación con síntomas de ansiedad, trastornos del sueño y aspectos del trabajo remoto en el contexto de la pandemia de COVID-19.

**Método:**

Estudio transversal y descriptivo realizado con 328 adultos que trabajan a distancia. Los datos se recopilaron en línea de febrero de 2021 a enero de 2022. Para la investigación del dolor y las evaluaciones del sueño y la ansiedad, se utilizó un cuestionario estructurado, el Cuestionario de Índice de Calidad del Sueño de Pittsburgh y el Generalized Anxiety Disorder-7, respectivamente.

**Resultados:**

La prevalencia de dolor crónico fue del 47,9% (IC del 95% = 42,5-53,3). Se identificaron asociaciones entre el dolor y la ansiedad, los trastornos del sueño y el tiempo sentado (p<0,01).

**Conclusión:**

La prevalencia del dolor crónico en el trabajo a distancia fue alta, con dolor de intensidad moderada asociado a la ansiedad, los trastornos del sueño y el tiempo sentado.

## INTRODUCTION

During the COVID-19 pandemic, many countries adopted social distancing as a strategy to reduce the spread of the disease and protect their populations. This led to a reduction in interactions among individuals due to the adoption of remote work, decreased circulation of people in public spaces, closure of businesses, and other measures^(1)^.

Globally, there was a significant push towards the remote work format, also known as teleworking^(2)^. However, full-time remote work can have certain drawbacks, including decreased social interaction, limited movement, excessive screen time, and blurred boundaries between professional and personal lives. All these factors can contribute to physical and mental fatigue, stress, anxiety, pain, and exhaustion^(3)^.

Everyone affected by the COVID-19 pandemic felt its impact due to changes in routine and continuous exposure to physical, psychological, and social stressors. These factors might have led to symptoms of anxiety and sleep disorders^(4)^, the emergence of new cases of chronic pain (CP), and the exacerbation of preexisting pain symptoms^(5, 6)^, especially among those who made drastic changes to their work routines. Pain is known to be multifactorial and can be linked to psychosocial factors. Therefore, during a crisis like the pandemic, the negative effects can be even more pronounced on individuals’ mental, social, and physical well-being^(7)^.

Based on the data from studies^(4, 5, 6, 7)^ and given the lack of specific research on CP and its correlations with sleep, anxiety, and the nuances of remote work among Brazilians during the pandemic, there was a recognized need to expand knowledge in this unique period. As a result, this study sought to explore the prevalence of CP among remote workers during the COVID-19 pandemic and determine if CP correlated with sleep disorders, anxiety symptoms, and the nature of remote work in that context.

## OBJECTIVE

To estimate the prevalence of chronic pain and its association with symptoms of anxiety, sleep disorders, and aspects of remote work during the COVID-19 pandemic in Brazil.

## METHODS

### Ethical Aspects

The study adhered to both national and international ethical standards and received approval from the Human Research Ethics Committee of the Federal University of São Carlos. All participants provided their consent by clicking on the designated area of the Informed Consent form, which is displayed on the first page of the interview questionnaire.

This research is based on a master’s thesis titled “Pain and associated factors in adults working remotely during the COVID-19 pandemic in Brazil.” The study was carried out under the Postgraduate Program in Nursing at the Federal University of São Carlos in 2022.

### Study Design, Period, and Location

Following the STROBE guidelines, this descriptive, cross-sectional study employed a quantitative methodology. Data was collected online between February 2021 and January 2022 using a structured questionnaire on the Google Forms® platform. Invitations to partake in the research were disseminated via email. Brazilian university graduate programs were asked to forward the invite to faculty and professors, and companies that transitioned to remote work were also targeted. The study was promoted through the Federal University of São Carlos’ institutional network and on the Meta® group’s social media channels, including Facebook, Instagram, and WhatsApp.

### Population or Sample; Inclusion and Exclusion Criteria

To determine the study’s sample size, calculations were made with the goal of estimating the prevalence of CP in an open-ended population. The prevalence value used in the calculation was sourced from a study^(8)^ that focused on the Brazilian populace. For all hypothetical sample sizes, a significance level of 5% was upheld. Variations in the absolute tolerable error ranged from 1 to 8 percentage points (p.p.). Thus, the final sample size for this research fluctuated between 368 to 387 participants, allowing for a 5 p.p. tolerable error.

The inclusion criteria covered adults 18 years and older who were engaged in remote work, be it full-time or part-time, during Brazil’s COVID-19 pandemic. The exclusion criteria ruled out individuals diagnosed with fibromyalgia, rheumatoid arthritis, or cancer, as well as frontline healthcare workers battling COVID-19.

### Study Protocol

To characterize the sample, we designed an online questionnaire to identify sociodemographic and economic profiles, the characteristics of remote work, and lifestyle habits.

For pain assessment, we structured a questionnaire to ascertain its presence, intensity, location, and duration. To evaluate pain intensity, participants were asked about the average pain they experienced during the pandemic, using an 11-point numerical pain scale ranging from 0 (indicating “no pain”) to 10 (representing the “worst possible pain”)^(9)^. Pain intensity was categorized as: 1-4 for mild pain, 5-6 for moderate pain, and 7-10 for severe pain^(10)^. Pain was defined as chronic if it persisted for a period of 6 months or more from the data collection date^(11)^.

For sleep evaluation, we used the Pittsburgh Sleep Quality Index (PSQI) questionnaire, developed by Buysse et al.^(12)^ and validated for Brazilian Portuguese by Bertolazi et al.^(13)^. This instrument, which assesses sleep quality and disturbances, comprises 19 questions. The highest possible score is 21 points, with a higher score indicating poorer sleep quality. A score between 0-4 suggests good sleep quality, 5-10 indicates poor quality, and scores above 10 point to sleep disorders^(12, 13)^.

For the assessment of anxiety, we employed the Generalized Anxiety Disorder-7 (GAD-7). This tool gauges the frequency of anxiety signs and symptoms over the past two weeks. It was devised by Spitzer et al.^(14)^, based on the DSM-IV criteria, and was validated by Kroenke et al.^(15)^. Its translation into Portuguese was done by Pfizer (Copyright, 2005 Pfizer Inc. New York, NY) and subsequently validated in Brazil by Moreno et al.^(16)^. Comprising 7 items, each is rated on a 4-point scale, ranging from 0 (not at all) to 3 (nearly every day). The total score can range from 0 to 21, with scores of 10 or higher potentially indicating symptoms of an anxiety disorder^(14)^.

### Results Analysis and Statistics

Descriptive statistics were used to characterize the sample. Categorical variables were depicted in both raw and relative frequencies, while continuous variables were represented with means and standard deviations (SD). In the association analyses, we employed the Poisson regression model with robust variance^(17)^, both in simple and multiple forms. A significance level of 5% (p<0.05) was maintained for all analyses.

## RESULTS

The study included 328 participants, with an average age of 32.96 (SD=8) years, ranging between 20 to 61 years. The majority were females (55.2%), and individuals with a completed higher education made up 86.3% of the sample. Socioeconomic and demographic data are detailed in Table 1.

**Table 1:** Socioeconomic and demographic profiles of Brazilian remote workers during the COVID-19 pandemic (N=328), Brazil, 2022

Variables	Frequency (%)
Sex	
Male	147 (44.8)
Female	181 (55.2)
Skin color
White	223 (68.0)
Brown/Mixed	70 (21.3)
Black	13 (4.0)
Yellow/Asian	14 (4.3)
Indigenous	1 (0.3)
Unknown/Ignored	7 (2.1)
Marital status	
Single	154 (47.0)
Married	113 (34.5)
Divorced	7 (2.1)
Living with partner	54 (16.5)
Education level	
High school completed	6 (1.8)
College/university completed	283 (86.3)
College/university incomplete	39 (11.9)
Family income before the pandemic	
Up to 1 minimum wage	4 (1.2)
From 1 to 4 minimum wages	81 (24.7)
From 4 to 10 minimum wages	149 (45.4)
More than 10 minimum wages	94 (28.7)
Current family income	
Up to 1 minimum wage	3 (0.9)
From 1 to 4 minimum wages	78 (23.8)
From 4 to 10 minimum wages	143 (43.6)
More than 10 minimum wages	104 (31.7)
Children	
Yes	85 (25.9)
No	243 (74.1)
Who they live with	
With 2 or more people	154 (47.0)
With one person	136 (41.5)
Alone	38 (11.6)

Regarding lifestyle habits, it’s notable that 95.7% of the participants did not smoke, and 62.5% consumed alcohol only socially. Before the pandemic, 61.3% of the sample engaged in physical activity, but this percentage dropped to 52.7% during the pandemic.

Table 2 presents data on remote work during the pandemic. Of the total sample, 74.4% (n=244) worked remotely full-time, while 25.6% (n=84) worked remotely part-time. Furthermore, 62.8% of respondents spent more than 8 hours a day sitting. When asked how much they missed their routines before the pandemic (with 0 being “not at all” and 10 being “extremely”), the average response was 7.17 (SD=2.67).

**Table 2:** Characterization of remote work and social behavior of Brazilian workers during the COVID-19 pandemic (N=328), Brazil, 2022

Remote work during the pandemic	Frequency (%)(1)
Period	
Full-time	244 (74.4)
Part-time	84 (25.6)
Hours worked per day	
Up to 6 hours of work	54 (16.5)
From 6 to 10 hours of work	216 (65.9)
More than 10 hours of work	58 (17.7)
Time spent sitting per day (in hours)	
Less than 4 hours	17 (5.2)
From 4 to 6 hours	34 (10.4)
From 6 to 8 hours	71 (21.7)
From 8 to 10 hours	123 (37.5)
From 10 to 12 hours	44 (13.4)
More than 12 hours	39 (11.9)
Social behavior	
Isolation	17 (5.2)
Leaving home as usual	34 (10.4)
Leaving home only when necessary	277 (84.5)

(1) *Values are presented as average ± standard deviation or as absolute and relative frequencies.*

The prevalence of CP stood at 47.9% (95% CI; 42.46%; 53.27%). Of the 157 people with CP, 53.50% (n=84) had already felt pain before the pandemic and continued to do so during this period, while 46.49% (n=73) began to experience CP after the pandemic began. The most affected areas of the body were the lumbar spine (62.4%), the cervical spine (47.1%), and the head (42.0%).

The average pain intensity during the pandemic was rated at 5.8 (SD=2.0), categorizing it as moderate. Among the participants with CP (n=157), the majority exhibited symptoms of Generalized Anxiety Disorder (GAD) at 53.5%. More than half reported poor sleep quality (59.2%), and 29.9% had sleep disorders. Notably, among people without CP, these percentages were significantly lower: 31.6% with GAD and 11.7% with sleep disorders. The average score on the GAD-7 was 10.9 (SD=5.8), and on the PSQI, it was 8.8 (SD=3.7).

Table 3 displays the association between CP and its intensity with symptoms of an anxiety disorder (≥10) and the presence of sleep disorders (>10). The results indicate that the presence of CP is associated with symptoms of an anxiety disorder (p<0.01), with the prevalence of anxiety being 69% higher in individuals with CP compared to those without.

**Table 3:** Association of chronic pain with anxiety symptoms and sleep disorders among Brazilian remote workers during the COVID-19 pandemic (n=157), Brazil, 2022

Models	PR	95% CI		p value^(1)^
Anxiety symptoms				
Pain intensity	1.11	1.03	1.20	<0.01
Chronic pain (yes/no)	1.69	1.30	2.21	<0.01
Sleep disorder				
Pain intensity	1.24	1.10	1.40	<0.01
Chronic pain (yes/no)	2.56	1.59	4.12	<0.01

*PR = prevalence ratio; 95% CI = Confidence Interval; (1) statistical significance.*

Further observation revealed that CP is correlated with sleep disorders (p<0.01). Individuals with CP have a 156% higher prevalence of sleep disorders compared to those without CP (Table 3).

No sociodemographic, economic, or lifestyle characteristics were associated with CP. However, the duration of sitting during remote work correlated with CP. The longer the sitting duration, the higher the prevalence of C P. Individuals who sit for 12 hours a day have a prevalence 3.35 times higher of having C P, and those sitting between 6 and 8 hours have a prevalence 3.28 times higher, compared to those who sit up to 6 hours a day (Table 4).

**Table 4:** Association of chronic pain with socioeconomic, demographic, work, and lifestyle characteristics among Brazilian remote workers during the COVID-19 pandemic (n=157), Brazil, 2022

Chronic pain	PR	95% CI		p value^(1)^
Age	1.01	0.99	1.02	0.46
Sex (female/male)	1.15	0.90	1.48	0.26
Marital status (with/without partner)	1.07	0.84	1.37	0.57
Completed education (higher/secondary)	1.16	0.80	1.68	0.42
Current family income (4 minimum wages or more/up to 4 minimum wages)	1.00	0.75	1.35	0.98
Has children (yes/no)	1.11	0.80	1.53	0.54
Who they live with (2 or more/1 person)	0.86	0.66	1.11	0.25
Who they live with (2 or more/alone)	1.13	0.70	1.83	0.61
Who they live with (1 person/alone)	1.32	0.84	2.09	0.23
Engages in physical activity during the pandemic (no/yes)	1.20	0.96	1.51	0.11
Smoking habit (yes/no)	1.18	0.74	1.87	0.49
Consumes alcoholic beverages (never/regularly)	1.11	0.76	1.62	0.58
Consumes alcoholic beverages (never/socially)	1.07	0.84	1.37	0.58
Consumes alcoholic beverages (regularly/socially)	0.96	0.68	1.37	0.83
Home office (exclusively/partially)	1.05	0.79	1.38	0.75
Sitting time (12h or more/10h-12h)	1.34	0.87	2.06	0.18
Sitting time (12h or more/8h-10h)	1.23	0.87	1.75	0.24
Sitting time (12h or more/6h-8h)	1.02	0.70	1.48	0.92
Sitting time (12h or more/up to 6h)	3.35	1.73	6.47	**<0.01**
Sitting time (10h-12h/8h-10h)	0.92	0.63	1.34	0.66
Sitting time (10h-12h/6h-8h)	0.76	0.52	1.12	0.16
Sitting time (10h-12h/up to 6h)	2.50	1.28	4.86	**<0.01**
Sitting time (8h-10h/6h-8h)	0.83	0.64	1.07	0.15
Sitting time (8h-10h/up to 6h)	2.72	1.49	4.97	**<0.01**
Sitting time (6h-8h/up to 6h)	3.28	1.81	5.97	**<0.01**
Working hours per day (up to 6h/6h-10h)	0.99	0.70	1.39	0.95
Working hours per day (up to 6h/more than 10h)	1.16	0.75	1.80	0.51
Working hours per day (6h-10h/more than 10h)	1.17	0.85	1.63	0.34

*The model is adjusted for age, gender, marital status, education, current family income, children, living situation, physical activity, smoking, drinking, medication, home office, sitting duration, and working hours. RP = prevalence ratio; 95% CI = 95% Confidence Interval; SM refers to Minimum wages; (1) p<0,01 indicates statistical significance.*

## DISCUSSION

The results of the current study revealed a prevalence of 47.9% (95% CI: 42.46%–53.27%) of individuals working remotely with C P, with this pain being of moderate intensity (average of 5.8, SD=2.0) and associated with symptoms of anxiety, sleep disorders, and prolonged sitting. It can be stated that a 47.9% prevalence of CP during the pandemic is a high figure, especially when considering studies that assessed pain in the general Brazilian population in the years prior to the pandemic. These studies reported rates ranging from 22% to 42% of CP^(8, 18, 19, 20, 21, 22)^. However, it should be noted that such studies did not focus on remote workers, which could help explain the elevated rate.

After the onset of the pandemic, a systematic review of the general population in Brazil (from April to August 2020) showed an average CP prevalence of 45.59%. However, observed CP prevalence in these studies ranged from 23.02% to 76.01%, with the lumbar region (41.96%) being the most affected^(21)^. Another systematic review noted that both the prevalence (95% CI: 0.29 to 0.96) and intensity (95% CI: -2.18 to -0.63) of pain were significantly higher during the pandemic compared to the pre-pandemic period, but it did not specify if the focus was on CP^(22)^.

Findings similar to our study were identified in a study of adults from Malta (n=388), where the majority were remote workers. 49% of them reported experiencing lumbar spine pain after the pandemic began, whereas 30% had experienced it prior to the pandemic^(23)^. Other studies also highlighted significant increases in both pain intensity and prevalence, finding rates between 27.9% and 70.5% of CP in remote workers during the pandemic^(24, 25, 26, 27, 28)^.

In Italy, a study by Curro et al.^(29)^ produced different findings: remote work was associated with a reduction in migraine pain intensity during chronic episodes. Psychological symptoms have also been emphasized in the literature due to the shift in people’s routines. For instance, recent studies indicate high levels of anxiety symptoms during the pandemic (47.2% to 56.3%), both in the general population^(30, 31)^ and among new remote workers, with the latter showing 19.6% to 55.8% anxiety prevalence^(4, 29, 32)^.

In our study, 53.5% of those with CP had symptoms of GAD — a high percentage, especially when compared to Curro et al.^(29)^, which found rates between 18.5% and 25.6% of anxiety in migraine sufferers. Bilen and Kucukkepeci^(33)^ found a 66.4% anxiety prevalence in individuals with C P. Nonetheless, there’s a gap in the literature concerning studies linking CP with anxiety symptoms among remote workers during the pandemic. This association between CP and anxiety (p<0.01) was observed in our research, indicating that workers with CP had a 69% higher prevalence ratio compared to those without C P.

In the study by Erman et al.^(34)^, the authors found that poor sleep quality and increased workload were predictors of anxiety. Curro et al.^(29)^ reported that increased pain intensity was linked to high levels of anxiety and reduced sleep quality and duration. Our study also found an association between pain and sleep disturbances.

Regarding remote work, our study observed that prolonged sitting is associated with C P, meaning the longer an individual remains seated, the higher their likelihood of developing CP. This is consistent with other studies that found sitting posture to be related to pain intensity^(23, 27, 35)^. Sagat et al.^(36)^ also found that individuals who moved more reported lower pain intensity. It’s worth noting that while sitting posture appears to be a factor in pain, CP is influenced by multiple factors that should be considered.

### Study Limitations

The sample size, initially estimated between 368 and 387 (with a tolerable error of 5 percentage points), was not achieved. Therefore, an approximate tolerable error of 5.4 percentage points is admitted. According to stratification by regions of Brazil, the sample size was only achieved in the Central-West (67) and Southeast (196) regions. This represents a significant limitation of the study.

The lack of investigation regarding the intensity of CP before the pandemic is also a limitation. This information could have been collected for a better understanding of the situation, although it would be a subjective data relying on the recollection of pain intensity perception. However, it would be useful for comparative purposes.

Finally, the remote mode of data collection can also be considered a limitation, as it doesn’t allow for a diverse sample and limits potential participants’ access due to outreach issues (emails sent to institutions; social media posts).

### Contributions to Nursing, Public Health, or Health Policy

This study is relevant because it provides important information about remote work, a work arrangement that became more common during the pandemic. This work format has both positive and negative aspects, as identified during this period. Therefore, it is important to pay attention to the physical and mental health of remote workers to understand how changes in their routines might lead to pain and other comorbidities. This research can help develop strategies and interventions in both the public and private sectors to minimize the negative effects of remote work.

This research provides valuable insights for health professionals and pain specialists by providing an overview of chronic pain, anxiety symptoms, and sleep disorders in remote workers in Brazil. Companies and health professionals must work together to establish preventive and therapeutic measures for these symptoms in this group, as many companies are likely to continue to adopt remote work.

## CONCLUSIONS

We identified CP prevalence in approximately half of the Brazilian remote workers sample during the COVID-19 pandemic. The pain intensity was moderate among them, with the lumbar spine being the most affected region. Moreover, about half of those reporting CP manifested this symptom during the pandemic. Most showed GAD symptoms, and nearly a third of those with CP experienced sleep disorders. In contrast, in those without chronic pain, these rates were significantly lower. CP pain was associated with GAD symptoms, sleep disorders, and prolonged sitting.

## Supplementary Material

0034-7167-reben-76-s1-e20230012-suppl01

## Data Availability

https://doi.org/10.48331/scielodata.YZPRGT

## References

[1] Xavier F, Olenscki JRW, Acosta AL, Sallum MAM, Saraiva AM. (2020). Análise de redes sociais como estratégia de apoio à vigilância em saúde durante a COVID-19. Estud Av.

[2] Yoshimoto T, Fujii T, Oka H, Kasahara S, Kawamata K, Matsudaira K. (2021). Pain status and its association with physical activity, psychological stress, and telework among Japanese workers with pain during the COVID-19 Pandemic. Int J Environ Res Public Health.

[3] Majumdar P, Biswas A, Sahu S. (2020). COVID-19 pandemic and lockdown: cause of sleep disruption, depression, somatic pain, and increased screen exposure of office workers and students of India. Chronobiol Int.

[4] Afonso P, Fonseca M, Teodoro T. (2021). Evaluation of anxiety, depression and sleep quality in full-time teleworkers. J Public Health.

[5] Charles S, Carayannopoulos AG, Pathak S., Deer T, Pope J, Lamer T, Provenzano D. (2019). Deer's Treatment of Pain.

[6] Clauw DJ, Hauser W, Cohen S P, Fitzcharles M. (2020). Considering the potential for an increase in chronic pain after the COVID-19 pandemic. PAIN.

[7] Shanthanna H, Strand NH, Provenzano DA, Lobo CA, Eldabe S, Bhatia A (2020). Caring for patients with pain during the COVID-19 pandemic: consensus recommendations from an international expert panel. Anaesthesia.

[8] Souza JB, Grossmann E, Perissinotti DMN, Oliveira JO, Fonseca PRB, Posso E P. (2017). Prevalence of chronic pain, treatments, perception, and interference on life activities: brazilian population-based survey. Pain Res Manag.

[9] Huskisson EC. (1974). Measurement of pain. Lancet.

[10] Serlin RC, Mendoza TR, Nakamura Y, Edwards KR, Cleeland CS. (1995). When is cancer pain mild, moderate or severe? grading pain severity by its interference with function. PAIN.

[11] International Association on the Study of Pain (IASP) (1994). Classification of chronic pain: descriptions of chronic pain syndromes and definitions of pain terms [Internet].

[12] Buysse DJ, Reynolds CF, Monk TH, Berman SR, Kupfer DJ. (1989). The Pittsburgh Sleep Quality Index: a new instrument for psychiatric practice and research. Psychiatry Res.

[13] Bertolazi AN, Fagondes SC, Hoff LS, Dartora EG, Miozzo ICS, Barba MEF (2011). Validation of the Brazilian Portuguese version of the Pittsburgh Sleep Quality Index. Sleep Med.

[14] Spitzer RL, Kroenke K, Williams JBW, Lowe B. (2006). A brief measure for assessing generalized anxiety disorder: the GAD-7. Arch Int Med.

[15] Kroenke K, Spitzer RL, Williams JBW, Monahan PO, Lowe B. (2007). Anxiety disorders in primary care: prevalence, impairment, comorbidity, and detection. An Int Med.

[16] Moreno (2016). Factor structure, reliability, and item parameters of the brazilian portuguese version of the GAD-7 questionnaire. Temas Psicol [Internet].

[17] Zou G. (2004). A modified poisson regression approach to prospective studies with binary data. Am J Epidemiol.

[18] Pereira FG, França MH, Paiva MCA, Andrade LH, Viana MC. (2017). Prevalence and clinical profile of chronic pain and its association with mental disorders. Rev Saúde Pública.

[19] Cabral DMC, Bracher ESB, Depintor JDP, Eluf J. (2014). Chronic pain prevalence and associated factors in a segment of the population of São Paulo City. PAIN.

[20] Ferreira KASL, Bastos TRPD, Andrade DCD. (2016). Prevalence of chronic pain in a metropolitan area of a developing country: a population-based study. Arq Neuro-Psiquiatr.

[21] Aguiar D P, Souza CPQ, Barbosa WJM, Santos FFU, Oliveira AS. (2021). Prevalence of chronic pain in Brazil: systematic review. BrJP.

[22] Papalia GF (2022). COVID-19 Pandemic Increases the Impact of Low Back Pain: a systematic review and metanalysis. Int J Environ Res Public Health.

[23] Grech S, Borg JN, Cuschieri S. (2022). Back pain, an aftermath of COVID-19 pandemic?: a Malta perspective. Musculoskeletal Care.

[24] Tesuka M, Tomohisa N, Saeki K, Yamato T, Naoto F. (2022). Association between abrupt change to teleworking and physical symptoms during the Coronavirus Disease 2019 (COVID-19) Emergency Declaration in Japan. J Occup Environ Med.

[25] Sharma N, Vaish H. (2020). Impact of COVID – 19 on mental health and physical load on women professionals: an online cross-sectional survey. Health Care Women Int.

[26] Prieto-Gonzalez P, Šutvajová M, Lesňáková A, Bartík P, Buľáková K, Friediger T. (2021). Back pain prevalence, intensity, and associated risk factors among female teachers in Slovakia during the COVID-19 Pandemic: a cross-sectional study. Healthcare.

[27] Zyznawaska JM, Bartecka WM. (2021). Remote working forced by COVID-19 pandemic and its influence on neck pain and low back pain among teachers. Med Pr.

[28] Mcallister MJ, Costigan PA, Davies JP, Diesbourg TL. (2022). The effect of training and workstation adjustability on teleworker discomfort during the COVID-19 pandemic. Applied Ergonomics.

[29] Curro CT, Ciacciarelli A, Vitale C, Vinci ES, Toscano A, Vita G (2021). Chronic migraine in the first COVID-19 lockdown: the impact of sleep, remote working, and other life/psychological changes. Neurol Sci.

[30] Morin CM, Bjorvatn B, Chung F, Holzinger B, Partinen M, Penzelet T (2021). Insomnia, anxiety, and depression during the COVID-19 pandemic: an international collaborative study. Sleep Med.

[31] Guilland R, Knapik J, Klokner SGM, Carlotto PAC, Trevisan KRR, Zimath SC (2021). Sintomas de depressão e ansiedade em trabalhadores durante a pandemia de COVID-19. Rev Psicol Org Trab.

[32] Senturk E, Sagaltici E, Genis B, Toker OG. (2021). Predictors of depression, anxiety and stress among remote workers during the COVID-19 pandemic. Work.

[33] Bilen A, Kucukkepeci H. (2022). Pain Intensity, Depression, and anxiety levels among patients with chronic pain during COVID-19 Pandemic. J Nerv Ment Dis.

[34] Erman S, Eser S, Bahadir G, Omur GT. (2021). Predictors of depression, anxiety and stress among remote workers during the COVID-19 pandemic. Work.

[35] Aegerter AM, Deforth M, Johnston V, Sjøgaard G, Volken T, Luomajoki H (2021). No evidence for an effect of working from home on neck pain and neck disability among Swiss office workers: short-term impact of COVID-19. Eur Spine J.

[36] Sagat P, Bartík P, Prieto González P, Tohănean DI, Knjaz D. (2020). Impact of COVID-19 quarantine on low back pain intensity, prevalence, and associated risk factors among adult citizens residing in Riyadh (Saudi Arabia): a cross-sectional study. Int J Environ Res Public Health.

