# Supplementary material for: Chronic pain and associated factors in remote work during the COVID-19 pandemic in Brazil
Source: Rev Bras Enferm. 2023 Dec 4;76(Suppl 1):e20230012. doi: 10.1590/0034-7167-2023-0012 (PMC10695055; doi:10.1590/0034-7167-2023-0012)
Supplement: 0034-7167-reben-76-s1-e20230012-suppl01 [file 0034-7167-reben-76-s1-e20230012-suppl01.pdf]

	Registro da resposta	Termo de Consentimento			
Entrevistados	Data	Você concorda com o termo acima e aceita participar do estudo?	Idade:	CIDADE e ESTADO que reside:	ESTADO
E01	2021-05-26	SIM	20	São Paulo SP	SP
E02	2021-05-29	SIM	20	Rio de Janeiro	RJ
E03	2021-06-29	SIM	21	São Carlos/SP	SP
E04	2021-02-09	SIM	22	São Carlos	SP
E05	2021-03-19	SIM	22	Goiânia, Goiás.	GO
E06	2021-04-24	SIM	22	Palmeira das Missões RS	RS
E07	2021-05-24	SIM	22	São Paulo/SP	SP
E08	2021-02-09	SIM	23	São Carlos / São Paulo	SP
E09	2021-02-09	SIM	23	São Carlos - SP	SP

E10	2021-04-24	SIM	23	São Paulo-SP	SP
E11	2021-04-26	SIM	23	Jundiaí, São Paulo	SP
E12	2021-05-23	SIM	23	Brasília	DF
E13	2022-01-06	SIM	23	São Paulo SP	SP
E14	2022-01-08	SIM	23	Santa Cruz do Capibaribe - PE	PE
E15	2022-01-09	SIM	23	São Paulo - SP	SP
E16	2021-02-09	SIM	24	Quatá SP	SP
E17	2021-02-26	SIM	24	Limeira, São Paulo	SP
E18	2021-03-10	SIM	24	Goiânia Goiás	GO
E19	2021-03-11	SIM	24	Cirilândia- Go	GO
E20	2021-03-20	SIM	24	São Paulo SP	SP

E21	2021-04-23	SIM	24	Rio de Janeiro - RJ	RJ
E22	2021-04-24	SIM	24	Guaxupé-MG	MG
E23	2021-05-24	SIM	24	Goiânia- GO	GO
E24	2021-05-25	SIM	24	São Carlos SP	SP
E25	2021-05-26	SIM	24	Garça sp	SP
E26	2022-01-09	SIM	24	JABOATÃO DOS GUARARAPES, PE	PB
E27	2021-02-06	SIM	25	São Paulo-SP	SP
E28	2021-02-09	SIM	25	São Carlos	SP
E29	2021-03-10	SIM	25	Maceió-AL	AL
E30	2021-03-19	SIM	25	Curitiba - PR	PR

E31	2021-03-19	SIM	25	São Paulo SP	SP
E32	2021-03-19	SIM	25	SP/SP	SP
E33	2021-04-24	SIM	25	Salvador	BA
E34	2021-04-25	SIM	25	Araraquara - SP	SP
E35	2021-04-25	SIM	25	Itupeva-SP	SP
E36	2021-05-31	SIM	25	Goiânia-GO	GO
E37	2021-06-24	SIM	25	Guarulhos/SP	SP
E38	2021-09-16	SIM	25	Teresina/Piauí	PI
E39	2022-01-05	SIM	25	Praia Grande, SP	SP
E40	2022-01-05	SIM	25	Blumenau, SC	SC
E41	2022-01-05	SIM	25	SÃO PAULO	SP
E42	2022-01-06	SIM	25	Santa Rosa - RS	RS

E43	2022-01-08	SIM	25	Brasília DF	DF
E44	2022-01-09	SIM	25	Juazeiro do Norte, CE	CE
E45	2022-01-09	SIM	25	SE	SE
E46	2022-01-09	SIM	25	Ribeirão Preto- SP	SP
E47	2021-02-06	SIM	26	SÃO PAULO, SP	SP
E48	2021-02-07	SIM	26	Aparecida de Goiânia/ GO	GO
E49	2021-02-10	SIM	26	São Paulo	SP
E50	2021-02-10	SIM	26	Cavalcante/GO	GO
E51	2021-02-12	SIM	26	São Carlos, São Paulo	SP
E52	2021-02-28	SIM	26	São Carlos/sp	SP

E53	2021-03-10	SIM	26	São Luiz Gonzaga - RS	RS
E54	2021-03-19	SIM	26	Goiânia/ Goiás	GO
E55	2021-03-19	SIM	26	São Carlos - SP	SP
E56	2021-03-20	SIM	26	Goiânia- Goiás	GO
E57	2021-03-20	SIM	26	Aparecidas de Goiânia, Goiás	GO
E58	2021-03-23	SIM	26	Goiânia/Goiás	GO
E59	2021-04-24	SIM	26	São Paulo - SP	SP
E60	2021-04-24	SIM	26	São Paulo/SP	SP
E61	2021-04-24	SIM	26	Maceió	AL
E62	2021-04-24	SIM	26	Niterói/RJ	RJ
E63	2021-04-24	SIM	26	São José dos Campos	SP

E64	2021-05-23	SIM	26	Goiânia-GO	GO
E65	2021-05-24	SIM	26	Impertariz- Ma	MA
E66	2021-05-26	SIM	26	Goiânia	GO
E67	2021-05-31	SIM	26	São Carlos - SP	SP
E68	2021-06-24	SIM	26	São Carlos - SP	SP
E69	2021-06-24	SIM	26	São Carlos - SP	SP
E70	2022-01-04	SIM	26	São Carlos - São Paulo	SP
E71	2022-01-06	SIM	26	São Paulo - SP	SP
E72	2022-01-07	SIM	26	Florianópolis SC	SC
E73	2022-01-10	SIM	26	Maracanaú - Ceará	CE
E74	2022-01-12	SIM	26	Curitiba, Paraná	PR

E75	2022-01-24	SIM	26	Goiânia - Goiás	GO
E76	2022-01-24	SIM	26	Goiânia - Goiás	GO
E77	2021-03-20	SIM	27	Goiânia - Goiás	GO
E78	2021-03-20	SIM	27	Aracruz/ES	ES
E79	2021-03-23	SIM	27	Ananindeua e Pará	PA
E80	2021-04-14	SIM	27	Belo Horizonte	MG
E81	2021-04-24	SIM	27	Florianópolis	SC
E82	2021-04-26	SIM	27	São Bernardo do Campo	SP
E83	2021-05-24	SIM	27	Goiânia, Goiás	GO
E84	2021-05-24	SIM	27	Goiânia	GO
E85	2021-05-25	SIM	27	Aparecida de Goiânia/GO	GO
E86	2021-06-24	SIM	27	SÃO PAULO - SP	SP

E87	2022-01-06	SIM	27	São Paulo capital	SP
E88	2022-01-08	SIM	27	Fortaleza, Ceará	CE
E89	2022-01-09	SIM	27	São Paulo - SP	SP
E90	2022-01-09	SIM	27	Lajeado, RS	RS
E91	2022-01-10	SIM	27	Recife PE	PE
E92	2021-02-06	SIM	28	Japi RN	RN
E93	2021-02-07	SIM	28	Inhumas, Goiás	GO
E94	2021-02-10	SIM	28	São Paulo/SP	SP
E95	2021-02-20	SIM	28	São Paulo	SP
E96	2021-02-23	SIM	28	São Carlos, São Paulo	SP
E97	2021-03-11	SIM	28	Ceres/GO	GO
E98	2021-03-19	SIM	28	Sao paulo sp	SP

E99	2021-03-20	SIM	28	Ribeirão Preto	SP
E100	2021-04-24	SIM	28	Curitiba, Paraná	PR
E101	2021-04-24	SIM	28	Rio de Janeiro/RJ	RJ
E102	2021-04-24	SIM	28	Goiânia Goias	GO
E103	2021-04-25	SIM	28	Vinhedo	SP
E104	2021-05-23	SIM	28	GOIÂNIA - GOIÁS	GO
E105	2021-05-24	SIM	28	Brasília DF	DF
E106	2021-05-24	SIM	28	Goiânia- GO	GO
E107	2021-05-24	SIM	28	Goiânia, Goiás	GO
E108	2021-05-24	SIM	28	Goiânia-GO	GO
E109	2021-05-26	SIM	28	São Paulo SP	SP

E110	2022-01-06	SIM	28	São Paulo - SP	SP
E111	2022-01-09	SIM	28	São Bento do Sul / SC	SC
E112	2022-01-10	SIM	28	Maracanaú-CE	CE
E113	2022-01-24	SIM	28	GOIÂNIA	GO
E114	2021-02-06	SIM	29	Unerlandia/MG	MG
E115	2021-02-09	SIM	29	São Carlos SP	SP
E116	2021-02-09	SIM	29	São Carlos	SP
E117	2021-02-09	SIM	29	Aparecida de Goiânia, Goiás	GO
E118	2021-02-09	SIM	29	Aparecida de Goiânia	GO
E119	2021-02-10	SIM	29	Goiânia	GO

E120	2021-02-20	SIM	29	São Paulo	SP
E121	2021-03-10	SIM	29	Goiânia - GO	GO
E122	2021-03-19	SIM	29	Aparecida de Goiania GO	GO
E123	2021-03-20	SIM	29	São Carlos SP	SP
E124	2021-03-20	SIM	29	Cariacica/ES	ES
E125	2021-03-23	SIM	29	Goiania e goiás	GO
E126	2021-03-23	SIM	29	Vitória ES	ES
E127	2021-04-23	SIM	29	Goiânia	GO
E128	2021-04-24	SIM	29	São Luís - Maranhão	MA
E129	2021-04-24	SIM	29	Feira de Santanta, BA	BA
E130	2021-04-24	SIM	29	São Paulo	SP

E131	2021-05-24	SIM	29	Goiânia, Goiás	GO
E132	2021-05-24	SIM	29	Goiânia	GO
E133	2021-05-24	SIM	29	Goiania, Goiás	GO
E134	2021-05-24	SIM	29	Goiânia-GO	GO
E135	2021-05-26	SIM	29	São Carlos - SP	SP
E136	2021-07-06	SIM	29	ARARAQUARA, SP	SP
E137	2022-01-08	SIM	29	Campo Limpo Paulista - SP	SP
E138	2022-01-08	SIM	29	Feira de Santana, BA	BA
E139	2022-01-08	SIM	29	Rio Tinto Paraíba	PB
E140	2022-01-10	SIM	29	Ponta Grossa - PR	PR
E141	2021-02-08	SIM	30	São Carlos, SP	SP

E142	2021-02-09	SIM	30	São Carlos/ SP	SP
E143	2021-02-10	SIM	30	Arujá-SP	SP
E144	2021-02-17	SIM	30	São Carlos sp	SP
E145	2021-02-23	SIM	30	São Carlos - SP	SP
E146	2021-04-15	SIM	30	Barra do Garças MT	MT
E147	2021-04-21	SIM	30	Goiânia/Goiás	GO
E148	2021-04-23	SIM	30	DF	DF
E149	2021-04-24	SIM	30	buri/ sp	SP
E150	2021-05-20	SIM	30	Cataguases - MG	MG
E151	2021-05-23	SIM	30	São Paulo/ SP	SP
E152	2021-05-26	SIM	30	Goiânia-Go	GO
E153	2021-05-26	SIM	30	Betim - MG	MG

E154	2021-05-27	SIM	30	Bauru/SP	SP
E155	2021-05-28	SIM	30	Campinas-sp	SP
E156	2021-07-06	SIM	30	Arujá, SP	SP
E157	2022-01-05	SIM	30	Santo André - SP	SP
E158	2022-01-08	SIM	30	Caraguatatuba/SP	SP
E159	2022-01-09	SIM	30	SÃO PAULO	SP
E160	2022-01-09	SIM	30	RJ	RJ
E161	2022-01-09	SIM	30	Brasília-DF	DF
E162	2022-01-09	SIM	30	Campinas, SP	SP
E163	2021-02-09	SIM	31	Uberlândia - MG	MG
E164	2021-03-19	SIM	31	Goiânia	GO
E165	2021-03-19	SIM	31	São Paulo	SP

E166	2021-04-23	SIM	31	Vitória	ES
E167	2021-04-24	SIM	31	São Paulo	SP
E168	2021-04-24	SIM	31	Pinhais	PR
E169	2021-05-24	SIM	31	São Paulo, SP	SP
E170	2021-05-25	SIM	31	Vila Velha ES	ES
E171	2022-01-06	SIM	31	São Paulo sp	SP
E172	2022-01-08	SIM	31	Pedreira SP	SP
E173	2022-01-08	SIM	31	Porto Alegre	RS
E174	2022-01-09	SIM	31	Bragança , s.p.	SP
E175	2021-02-06	SIM	32	SAO PAULO	SP
E176	2021-02-09	SIM	32	Barueri SP	SP

E177	2021-02-09	SIM	32	São Paulo- SP	SP
E178	2021-02-09	SIM	32	São Paulo/SP	SP
E179	2021-02-21	SIM	32	Sacramento, MG	MG
E180	2021-02-26	SIM	32	São Carlos - São Paulo	SP
E181	2021-03-19	SIM	32	Porto Velho - Rondônia	RO
E182	2021-04-23	SIM	32	Vitória ES	ES
E183	2021-04-24	SIM	32	Brasília/DF	DF
E184	2021-04-24	SIM	32	Guararema, SP	SP
E185	2021-04-25	SIM	32	Maceio alagoas	AL
E186	2021-05-23	SIM	32	Goiânia, goiás	GO
E187	2021-05-24	SIM	32	Aparecida de Goiânia, Goiás	GO

E188	2021-05-24	SIM	32	Aparecida de Goiânia- Goias	GO
E189	2021-05-28	SIM	32	São José dos Campos - SP	SP
E190	2022-01-09	SIM	32	Goiania GO	GO
E191	2022-01-11	SIM	32	Rio de Janeiro - RJ	RJ
E192	2021-03-19	SIM	33	Santos/SP	SP
E193	2021-04-25	SIM	33	Curitiba Paraná	PR
E194	2021-04-26	SIM	33	Sorocaba-sp	SP
E195	2021-04-26	SIM	33	Três lagoas MS	MS
E196	2021-05-29	SIM	33	PAULINIA - SP	SP
E197	2021-06-24	SIM	33	Rondonopolis mt	MT
E198	2021-06-24	SIM	33	São Paulo - SP	SP

E199	2022-01-05	SIM	33	Igrejinha, RS	RS
E200	2022-01-08	SIM	33	Rio de Janeiro/RJ	RJ
E201	2022-01-09	SIM	33	São Paulo, SP	SP
E202	2022-01-10	SIM	33	são paulo - sp	SP
E203	2022-01-10	SIM	33	Mogi das Cruzes - SP	SP
E204	2022-01-10	SIM	33	Sorocaba	SP
E205	2021-02-08	SIM	34	São Carlos / SP	SP
E206	2021-02-09	SIM	34	Piracicaba	SP
E207	2021-03-06	SIM	34	Botucatu/SP	SP
E208	2021-03-19	SIM	34	Goiânia/Goiás	GO
E209	2021-03-29	SIM	34	Piumhi/MG	MG
E210	2021-04-19	SIM	34	São Carlos/SP	SP

E211	2021-04-23	SIM	34	Contagem MG	MG
E212	2021-04-24	SIM	34	Salvador/BA	BA
E213	2021-04-24	SIM	34	São Paulo / SP	SP
E214	2021-05-23	SIM	34	Goiânia	GO
E215	2021-05-24	SIM	34	Anápolis/ Goiás	GO
E216	2022-01-06	SIM	34	Rio de Janeiro/RJ	RJ
E217	2022-01-09	SIM	34	Ananindeua/Pará	PA
E218	2022-01-09	SIM	34	Gama - DF	DF
E219	2021-02-23	SIM	35	Alpinópolis / MG	MG
E220	2021-03-19	SIM	35	Goiânia Goiás	GO
E221	2021-04-23	SIM	35	Paulistana	PI

E222	2021-04-24	SIM	35	São Carlos SP	SP
E223	2021-04-25	SIM	35	São Paulo/SP	SP
E224	2021-05-24	SIM	35	Goiânia	GO
E225	2021-05-24	SIM	35	São Carlos-SP	SP
E226	2021-06-12	SIM	35	São Carlos/SP	SP
E227	2021-06-24	SIM	35	São Paulo - SP	SP
E228	2022-01-10	SIM	35	Mogi Guaçu - SP	SP
E229	2022-01-10	SIM	35	São Paulo/SP	SP
E230	2021-02-06	SIM	36	São Carlos/SP	SP
E231	2021-02-08	SIM	36	Ribeirão Preto, SP	SP

E232	2021-02-23	SIM	36	Rio Claro	SP
E233	2021-03-22	SIM	36	Toledo, Paraná	PR
E234	2021-04-22	SIM	36	Goiânia	GO
E235	2021-04-26	SIM	36	São Paulo - São Paulo	SP
E236	2021-05-23	SIM	36	Campinas-sp	SP
E237	2021-05-23	SIM	36	Rio de Janeiro RJ	RJ
E238	2021-05-31	SIM	36	São Carlos	SP
E239	2021-06-24	SIM	36	São Paulo/SP	SP
E240	2022-01-08	SIM	36	GUARUJÁ SP	SP
E241	2022-01-08	SIM	36	Curitiba PR	PR
E242	2022-01-09	SIM	36	Cabo Frio - RJ	RJ

E243	2022-01-10	SIM	36	Florianópolis	SC
E244	2021-02-08	SIM	37	São Carlos	SP
E245	2021-02-09	SIM	37	Nova Resende MG	MG
E246	2021-02-09	SIM	37	São Carlos	SP
E247	2021-02-09	SIM	37	São Carlos -SP	SP
E248	2021-03-19	SIM	37	Porto Velho, RONDÔNIA	RO
E249	2021-05-25	SIM	37	São Paulo/SP	SP
E250	2021-05-28	SIM	37	São José dos Campos SP	SP
E251	2022-01-08	SIM	37	Campos dos goytacazes - RJ	RJ
E252	2021-02-08	SIM	38	Araraquara S. P	SP
E253	2021-03-19	SIM	38	GOIANIA	GO

E254	2021-03-22	SIM	38	Vitória	ES
E255	2021-04-24	SIM	38	Uberlândia-MG	MG
E256	2021-04-24	SIM	38	Goiânia GO	GO
E257	2021-05-21	SIM	38	Cataguases-MG	MG
E258	2021-06-24	SIM	38	Santo André - São Paulo	SP
E259	2022-01-09	SIM	38	Porto Alegre	RS
E260	2021-02-09	SIM	39	Jaragua do sul - SC	SC
E261	2021-03-20	SIM	39	Araraquara SP	SP
E262	2021-03-22	SIM	39	Rio de Janeiro	RJ
E263	2021-04-24	SIM	39	São Paulo (SP)	SP

E264	2021-05-24	SIM	39	Goiânia- GO	GO
E265	2021-05-27	SIM	39	São José dos Campos/SP	SP
E266	2021-06-24	SIM	39	são paulo, sp	SP
E267	2022-01-08	SIM	39	João Pessoa Paraíba	PB
E268	2021-04-01	SIM	40	Recife - Pernambuco	PE
E269	2021-04-23	SIM	40	Rio das Ostras RJ	RJ
E270	2021-04-24	SIM	40	São Paulo	SP
E271	2021-05-05	SIM	40	São carlos-SP	SP
E272	2021-05-23	SIM	40	Goiania GO	GO
E273	2021-05-28	SIM	40	SÃO JOSÉ DOS CAMPOS	SP
E274	2022-01-06	SIM	40	Belo Horizonte	MG
E275	2021-02-08	SIM	41	SÃO CARLOS	SP

E276	2021-02-20	SIM	41	São Carlos	SP
E277	2021-03-19	SIM	41	Florianópolis - SC	SC
E278	2021-04-08	SIM	41	Aparecida de Goiânia, Goiás	GO
E279	2021-04-24	SIM	41	São Paulo	SP
E280	2021-05-28	SIM	41	Sao Jose dos Campos SP	SP
E281	2021-02-09	SIM	42	Florianópolis	SC
E282	2021-05-24	SIM	42	Goiânia	GO
E283	2021-05-25	SIM	42	Goiânia- Goiás	GO
E284	2021-05-27	SIM	42	Brasilia/DF	DF
E285	2022-01-06	SIM	42	Piranguinho/MG	MG
E286	2021-02-10	SIM	43	Goiânia	GO
E287	2021-02-23	SIM	43	São Carlos SP	SP

E288	2021-03-20	SIM	43	Araraquara	SP
E289	2021-03-24	SIM	43	Rio de Janeiro	RJ
E290	2021-05-01	SIM	43	RJ	RJ
E291	2021-05-28	SIM	43	São José dos Campos	SP
E292	2022-01-09	SIM	43	Praia Grande SP	SP
E293	2021-02-08	SIM	44	São Carlos/SP	SP
E294	2021-02-22	SIM	44	São Carlos, SP	SP
E295	2021-03-19	SIM	44	Descalvado / SP	SP
E296	2021-04-27	SIM	44	Ribeirao Preto SP	SP
E297	2021-05-28	SIM	44	Salvador Ba	BA
E298	2021-07-07	SIM	44	Marília	SP
E299	2021-02-25	SIM	45	Salvador-Ba	BA

E300	2021-02-26	SIM	45	São Carlos- Sao Paulo	SP
E301	2021-07-08	SIM	45	MARÍLIA-SP	SP
E302	2021-02-22	SIM	46	Novo Hamburgo/RS	RS
E303	2021-03-30	SIM	46	Maceió AL	AL
E304	2021-07-07	SIM	46	São Carlos	SP
E305	2021-03-19	SIM	47	Goiânia	GO
E306	2021-03-22	SIM	47	São Paulo	SP
E307	2021-04-24	SIM	47	Belo Horizonte	MG
E308	2021-03-29	SIM	48	Maceió	AL
E309	2021-04-23	SIM	48	Petrópolis/RJ	RJ
E310	2021-03-19	SIM	49	Goiânia-Go	GO
E311	2021-05-28	SIM	49	São José Campos SP	SP

E312	2021-07-07	SIM	49	São Carlos	SP
E313	2021-03-10	SIM	50	Goiânia	GO
E314	2022-01-09	SIM	50	maringa=PR	PR
E315	2021-02-08	SIM	51	São Carlos - SP	SP
E316	2021-02-10	SIM	51	Piracicaba- SP	SP
E317	2021-05-27	SIM	51	Goiânia GO	GO
E318	2021-07-06	SIM	54	São Carlos SP	SP
E319	2021-02-20	SIM	55	Lins	SP
E320	2021-02-08	SIM	56	São Carlos - SP	SP
E321	2021-02-08	SIM	56	Araraquara São Paulo	SP
E322	2021-05-27	SIM	56	São Paulo	SP
E323	2021-05-27	SIM	56	Goiânia	GO

E324	2021-02-08	SIM	57	São Carlos-SP	SP
E325	2021-02-20	SIM	57	Araraquara/SP	SP
E326	2021-03-10	SIM	57	Curitibanos/SC	SC
E327	2021-03-19	SIM	58	São Paulo	SP
E328	2021-07-06	SIM	61	São Carlos - SP	SP

REGIÃO	Sexo:	Raça/cor:	Estado civil:	Renda familiar antes da pandemia (março de 2020):
Sudeste	Feminino	Branco	Solteiro (a)	mais de 10 salários mínimos
Sudeste	Feminino	Preto	Solteiro (a)	de 1 a 4 salários mínimos
Sudeste	Feminino	Branco	Solteiro (a)	de 1 a 4 salários mínimos
Sudeste	Feminino	parda	Solteiro (a)	de 1 a 4 salários mínimos
Centro-Oeste	Masculino	parda	Vive com companheiro (a)	de 1 a 4 salários mínimos
Sul	Masculino	parda	Solteiro (a)	de 1 a 4 salários mínimos
Sudeste	Masculino	parda	Solteiro (a)	de 1 a 4 salários mínimos
Sudeste	Feminino	parda	Solteiro (a)	de 1 a 4 salários mínimos
Sudeste	Feminino	Branco	Vive com companheiro (a)	de 1 a 4 salários mínimos

Sudeste	Masculino	parda	Solteiro (a)	de 4 a 10 salários mínimos
Sudeste	Masculino	Branco	Solteiro (a)	de 4 a 10 salários mínimos
Distrito Federal	Feminino	parda	Solteiro (a)	mais de 10 salários mínimos
Sudeste	Feminino	Ignorado	Vive com companheiro (a)	de 1 a 4 salários mínimos
Nordeste	Masculino	Branco	Solteiro (a)	de 4 a 10 salários mínimos
Sudeste	Masculino	Branco	Solteiro (a)	de 4 a 10 salários mínimos
Sudeste	Feminino	Branco	Solteiro (a)	mais de 10 salários mínimos
Sudeste	Feminino	Branco	Vive com companheiro (a)	de 1 a 4 salários mínimos
Centro-Oeste	Feminino	Preto	Solteiro (a)	de 4 a 10 salários mínimos
Centro-Oeste	Feminino	Branco	Solteiro (a)	mais de 4 salários mínimos
Sudeste	Feminino	Branco	Vive com companheiro (a)	mais de 10 salários mínimos

Sudeste	Masculino	Branco	Solteiro (a)	até um salário mínimo
Sudeste	Masculino	Branco	Solteiro (a)	de 1 a 4 salários mínimos
Centro-Oeste	Masculino	parda	Casado (a)	mais de 10 salários mínimos
Sudeste	Feminino	parda	Vive com companheiro (a)	de 4 a 10 salários mínimos
Sudeste	Feminino	Branco	Solteiro (a)	de 1 a 4 salários mínimos
Nordeste	Masculino	Branco	Solteiro (a)	de 1 a 4 salários mínimos
Sudeste	Feminino	Branco	Solteiro (a)	de 1 a 4 salários mínimos
Sudeste	Feminino	Branco	Solteiro (a)	mais de 10 salários mínimos
Nordeste	Feminino	parda	Solteiro (a)	até um salário mínimo
Sul	Masculino	Branco	Vive com companheiro (a)	mais de 10 salários mínimos

Sudeste	Feminino	Ignorado	Vive com companheiro (a)	mais de 10 salários mínimos
Sudeste	Masculino	parda	Solteiro (a)	de 1 a 4 salários mínimos
Nordeste	Masculino	parda	Solteiro (a)	de 4 a 10 salários mínimos
Sudeste	Masculino	Branco	Solteiro (a)	de 4 a 10 salários mínimos
Sudeste	Masculino	Branco	Solteiro (a)	de 1 a 4 salários mínimos
Centro-Oeste	Masculino	parda	Solteiro (a)	de 1 a 4 salários mínimos
Sudeste	Feminino	Branco	Casado (a)	de 4 a 10 salários mínimos
Nordeste	Feminino	Amarelo	Solteiro (a)	de 4 a 10 salários mínimos
Sudeste	Feminino	Branco	Solteiro (a)	de 1 a 4 salários mínimos
Sul	Masculino	Branco	Solteiro (a)	de 1 a 4 salários mínimos
Sudeste	Feminino	Amarelo	Solteiro (a)	de 4 a 10 salários mínimos
Sul	Masculino	Branco	Solteiro (a)	de 1 a 4 salários mínimos

Distrito Federal	Masculino	Branco	Vive com companheiro (a)	de 1 a 4 salários mínimos
Nordeste	Feminino	Branco	Solteiro (a)	de 4 a 10 salários mínimos
Nordeste	Masculino	Branco	Vive com companheiro (a)	de 1 a 4 salários mínimos
Sudeste	Masculino	Branco	Vive com companheiro (a)	de 1 a 4 salários mínimos
Sudeste	Feminino	Branco	Solteiro (a)	de 4 a 10 salários mínimos
Centro-Oeste	Feminino	Branco	Solteiro (a)	mais de 4 salários mínimos
Sudeste	Feminino	parda	Solteiro (a)	de 4 a 10 salários mínimos
Centro-Oeste	Feminino	parda	Solteiro (a)	mais de 4 salários mínimos
Sudeste	Masculino	Branco	Solteiro (a)	de 4 a 10 salários mínimos
Sudeste	Feminino	parda	Solteiro (a)	de 1 a 4 salários mínimos

Sul	Feminino	Branco	Solteiro (a)	de 1 a 4 salários mínimos
Centro-Oeste	Feminino	parda	Solteiro (a)	de 1 a 4 salários mínimos
Sudeste	Masculino	Branco	Solteiro (a)	de 4 a 10 salários mínimos
Centro-Oeste	Feminino	parda	Vive com companheiro (a)	de 4 a 10 salários mínimos
Centro-Oeste	Masculino	parda	Solteiro (a)	de 1 a 4 salários mínimos
Centro-Oeste	Feminino	Branco	Solteiro (a)	mais de 4 salários mínimos
Sudeste	Masculino	parda	Solteiro (a)	de 4 a 10 salários mínimos
Sudeste	Masculino	Branco	Solteiro (a)	de 4 a 10 salários mínimos
Nordeste	Masculino	Branco	Solteiro (a)	mais de 4 salários mínimos
Sudeste	Masculino	Branco	Solteiro (a)	de 4 a 10 salários mínimos
Sudeste	Masculino	Branco	Casado (a)	de 4 a 10 salários mínimos

Centro-Oeste	Feminino	parda	Solteiro (a)	de 1 a 4 salários mínimos
Nordeste	Feminino	Branco	Casado (a)	de 1 a 4 salários mínimos
Centro-Oeste	Feminino	parda	Casado (a)	de 1 a 4 salários mínimos
Sudeste	Masculino	Branco	Solteiro (a)	de 1 a 4 salários mínimos
Sudeste	Masculino	Branco	Vive com companheiro (a)	de 1 a 4 salários mínimos
Sudeste	Feminino	Branco	Vive com companheiro (a)	de 1 a 4 salários mínimos
Sudeste	Feminino	Branco	Vive com companheiro (a)	de 4 a 10 salários mínimos
Sudeste	Masculino	Branco	Solteiro (a)	de 4 a 10 salários mínimos
Sul	Feminino	Branco	Solteiro (a)	mais de 10 salários mínimos
Nordeste	Masculino	Branco	Solteiro (a)	de 1 a 4 salários mínimos
Sul	Masculino	Branco	Solteiro (a)	de 4 a 10 salários mínimos

Centro-Oeste	Feminino	Branco	Vive com companheiro (a)	de 1 a 4 salários mínimos
Centro-Oeste	Feminino	Branco	Solteiro (a)	de 1 a 4 salários mínimos
Centro-Oeste	Feminino	Branco	Vive com companheiro (a)	de 4 a 10 salários mínimos
Sudeste	Masculino	Branco	Solteiro (a)	mais de 4 salários mínimos
Norte	Feminino	Branco	Casado (a)	mais de 4 salários mínimos
Sudeste	Feminino	Branco	Solteiro (a)	de 1 a 4 salários mínimos
Sul	Masculino	Branco	Solteiro (a)	de 1 a 4 salários mínimos
Sudeste	Masculino	Branco	Vive com companheiro (a)	mais de 10 salários mínimos
Centro-Oeste	Feminino	Branco	Solteiro (a)	mais de 10 salários mínimos
Centro-Oeste	Feminino	parda	Solteiro (a)	de 1 a 4 salários mínimos
Centro-Oeste	Feminino	Branco	Solteiro (a)	de 1 a 4 salários mínimos
Sudeste	Feminino	parda	Solteiro (a)	de 1 a 4 salários mínimos

Sudeste	Masculino	parda	Solteiro (a)	de 1 a 4 salários mínimos
Nordeste	Masculino	parda	Solteiro (a)	de 4 a 10 salários mínimos
Sudeste	Masculino	parda	Solteiro (a)	de 4 a 10 salários mínimos
Sul	Masculino	Branco	Vive com companheiro (a)	mais de 10 salários mínimos
Nordeste	Masculino	Branco	Solteiro (a)	mais de 10 salários mínimos
Norte	Feminino	parda	Solteiro (a)	de 1 a 4 salários mínimos
Centro-Oeste	Masculino	parda	Solteiro (a)	mais de 10 salários mínimos
Sudeste	Feminino	Branco	Solteiro (a)	mais de 10 salários mínimos
Sudeste	Feminino	Amarelo	Solteiro (a)	mais de 10 salários mínimos
Sudeste	Feminino	Branco	Solteiro (a)	até um salário mínimo
Centro-Oeste	Masculino	Branco	Solteiro (a)	mais de 10 salários mínimos
Sudeste	Feminino	Branco	Solteiro (a)	de 4 a 10 salários mínimos

Sudeste	Feminino	Branco	Vive com companheiro (a)	mais de 10 salários mínimos
Sul	Masculino	Branco	Vive com companheiro (a)	de 4 a 10 salários mínimos
Sudeste	Feminino	Branco	Vive com companheiro (a)	mais de 4 salários mínimos
Centro-Oeste	Masculino	Branco	Casado (a)	de 4 a 10 salários mínimos
Sudeste	Masculino	Amarelo	Casado (a)	de 4 a 10 salários mínimos
Centro-Oeste	Masculino	Branco	Solteiro (a)	de 1 a 4 salários mínimos
Distrito Federal	Feminino	Ignorado	Casado (a)	mais de 10 salários mínimos
Centro-Oeste	Feminino	Branco	Vive com companheiro (a)	de 4 a 10 salários mínimos
Centro-Oeste	Feminino	parda	Solteiro (a)	de 4 a 10 salários mínimos
Centro-Oeste	Feminino	Branco	Casado (a)	de 4 a 10 salários mínimos
Sudeste	Feminino	Branco	Solteiro (a)	mais de 10 salários mínimos

Sudeste	Feminino	Branco	Casado (a)	de 4 a 10 salários mínimos
Sul	Masculino	Branco	Solteiro (a)	de 1 a 4 salários mínimos
Nordeste	Masculino	parda	Casado (a)	de 4 a 10 salários mínimos
Centro-Oeste	Masculino	Branco	Solteiro (a)	de 1 a 4 salários mínimos
Sudeste	Feminino	Branco	Vive com companheiro (a)	mais de 4 salários mínimos
Sudeste	Feminino	Branco	Solteiro (a)	mais de 10 salários mínimos
Sudeste	Feminino	Amarelo	Solteiro (a)	de 1 a 4 salários mínimos
Centro-Oeste	Masculino	Branco	Solteiro (a)	mais de 4 salários mínimos
Centro-Oeste	Masculino	parda	Casado (a)	de 4 a 10 salários mínimos
Centro-Oeste	Masculino	Branco	Casado (a)	de 1 a 4 salários mínimos

Sudeste	Feminino	Branco	Solteiro (a)	mais de 4 salários mínimos
Centro-Oeste	Feminino	parda	Solteiro (a)	de 1 a 4 salários mínimos
Centro-Oeste	Feminino	Branco	Solteiro (a)	de 4 a 10 salários mínimos
Sudeste	Masculino	Branco	Solteiro (a)	mais de 10 salários mínimos
Sudeste	Masculino	parda	Solteiro (a)	de 4 a 10 salários mínimos
Centro-Oeste	Feminino	Branco	Vive com companheiro (a)	mais de 10 salários mínimos
Sudeste	Feminino	Branco	Solteiro (a)	de 1 a 4 salários mínimos
Centro-Oeste	Masculino	Branco	Vive com companheiro (a)	de 4 a 10 salários mínimos
Nordeste	Feminino	Branco	Vive com companheiro (a)	de 4 a 10 salários mínimos
Nordeste	Masculino	parda	Solteiro (a)	de 1 a 4 salários mínimos
Sudeste	Masculino	Branco	Solteiro (a)	mais de 4 salários mínimos

Centro-Oeste	Feminino	Preto	Solteiro (a)	de 1 a 4 salários mínimos
Centro-Oeste	Masculino	Branco	Casado (a)	de 4 a 10 salários mínimos
Centro-Oeste	Feminino	Branco	Vive com companheiro (a)	mais de 10 salários mínimos
Centro-Oeste	Feminino	Branco	Solteiro (a)	de 4 a 10 salários mínimos
Sudeste	Feminino	Branco	Casado (a)	mais de 10 salários mínimos
Sudeste	Feminino	Branco	Solteiro (a)	de 4 a 10 salários mínimos
Sudeste	Feminino	Branco	Solteiro (a)	de 1 a 4 salários mínimos
Nordeste	Masculino	parda	Solteiro (a)	de 1 a 4 salários mínimos
Nordeste	Masculino	Branco	Solteiro (a)	de 1 a 4 salários mínimos
Sul	Feminino	Branco	Solteiro (a)	de 1 a 4 salários mínimos
Sudeste	Masculino	Branco	Solteiro (a)	de 4 a 10 salários mínimos

Sudeste	Feminino	Branco	Casado (a)	de 4 a 10 salários mínimos
Sudeste	Feminino	Branco	Casado (a)	de 4 a 10 salários mínimos
Sudeste	Feminino	Branco	Solteiro (a)	de 4 a 10 salários mínimos
Sudeste	Masculino	Amarelo	Solteiro (a)	de 1 a 4 salários mínimos
Centro-Oeste	Masculino	Branco	Solteiro (a)	mais de 10 salários mínimos
Centro-Oeste	Feminino	parda	Casado (a)	mais de 4 salários mínimos
Distrito Federal	Masculino	parda	Solteiro (a)	de 4 a 10 salários mínimos
Sudeste	Masculino	Branco	Solteiro (a)	de 4 a 10 salários mínimos
Sudeste	Masculino	Branco	Solteiro (a)	de 4 a 10 salários mínimos
Sudeste	Feminino	parda	Casado (a)	mais de 10 salários mínimos
Centro-Oeste	Masculino	Branco	Casado (a)	de 4 a 10 salários mínimos
Sudeste	Feminino	parda	Casado (a)	de 1 a 4 salários mínimos

Sudeste	Masculino	Branco	Solteiro (a)	mais de 10 salários mínimos
Sudeste	Feminino	Branco	Casado (a)	de 4 a 10 salários mínimos
Sudeste	Feminino	Branco	Casado (a)	mais de 10 salários mínimos
Sudeste	Masculino	Branco	Casado (a)	mais de 10 salários mínimos
Sudeste	Masculino	Branco	Casado (a)	mais de 10 salários mínimos
Sudeste	Masculino	Branco	Casado (a)	mais de 10 salários mínimos
Sudeste	Masculino	Preto	Casado (a)	de 4 a 10 salários mínimos
Distrito Federal	Masculino	Preto	Solteiro (a)	de 4 a 10 salários mínimos
Sudeste	Masculino	Branco	Vive com companheiro (a)	de 4 a 10 salários mínimos
Sudeste	Masculino	Branco	Solteiro (a)	mais de 4 salários mínimos
Centro-Oeste	Feminino	Branco	Solteiro (a)	de 4 a 10 salários mínimos
Sudeste	Feminino	Branco	Solteiro (a)	de 4 a 10 salários mínimos

Sudeste	Masculino	Branco	Casado (a)	mais de 4 salários mínimos
Sudeste	Masculino	parda	Casado (a)	mais de 10 salários mínimos
Sul	Masculino	parda	Casado (a)	de 1 a 4 salários mínimos
Sudeste	Feminino	Branco	Solteiro (a)	de 4 a 10 salários mínimos
Sudeste	Feminino	Branco	Solteiro (a)	de 4 a 10 salários mínimos
Sudeste	Feminino	Branco	Solteiro (a)	de 1 a 4 salários mínimos
Sudeste	Feminino	Branco	Casado (a)	mais de 10 salários mínimos
Sul	Masculino	Branco	Solteiro (a)	mais de 10 salários mínimos
Sudeste	Masculino	Branco	Vive com companheiro (a)	de 4 a 10 salários mínimos
Sudeste	Feminino	Branco	Solteiro (a)	de 4 a 10 salários mínimos
Sudeste	Feminino	Branco	Vive com companheiro (a)	mais de 10 salários mínimos

Sudeste	Masculino	Branco	Vive com companheiro (a)	mais de 10 salários mínimos
Sudeste	Masculino	Amarelo	Vive com companheiro (a)	mais de 10 salários mínimos
Sudeste	Feminino	Branco	Solteiro (a)	de 1 a 4 salários mínimos
Sudeste	Feminino	Branco	Casado (a)	mais de 4 salários mínimos
Norte	Feminino	parda	Casado (a)	de 1 a 4 salários mínimos
Sudeste	Feminino	Branco	Casado (a)	mais de 4 salários mínimos
Distrito Federal	Masculino	parda	Casado (a)	mais de 10 salários mínimos
Sudeste	Masculino	Branco	Vive com companheiro (a)	mais de 4 salários mínimos
Nordeste	Masculino	Ignorado	Casado (a)	mais de 10 salários mínimos
Centro-Oeste	Feminino	Preto	Casado (a)	de 4 a 10 salários mínimos
Centro-Oeste	Masculino	parda	Casado (a)	de 4 a 10 salários mínimos

Centro-Oeste	Feminino	Branco	Casado (a)	de 4 a 10 salários mínimos
Sudeste	Feminino	Branco	Casado (a)	mais de 10 salários mínimos
Centro-Oeste	Masculino	parda	Casado (a)	de 1 a 4 salários mínimos
Sudeste	Masculino	Branco	Vive com companheiro (a)	de 4 a 10 salários mínimos
Sudeste	Masculino	Branco	Solteiro (a)	de 4 a 10 salários mínimos
Sul	Feminino	Branco	Casado (a)	mais de 10 salários mínimos
Sudeste	Feminino	Branco	Solteiro (a)	de 1 a 4 salários mínimos
Centro-Oeste	Feminino	Branco	Vive com companheiro (a)	mais de 10 salários mínimos
Sudeste	Masculino	parda	Casado (a)	de 4 a 10 salários mínimos
Centro-Oeste	Feminino	Branco	Casado (a)	mais de 10 salários mínimos
Sudeste	Feminino	Branco	Casado (a)	de 4 a 10 salários mínimos

Sul	Masculino	Branco	Solteiro (a)	de 4 a 10 salários mínimos
Sudeste	Masculino	Branco	Vive com companheiro (a)	de 1 a 4 salários mínimos
Sudeste	Feminino	Branco	Solteiro (a)	de 1 a 4 salários mínimos
Sudeste	Feminino	Branco	Casado (a)	mais de 10 salários mínimos
Sudeste	Masculino	Amarelo	Solteiro (a)	de 4 a 10 salários mínimos
Sudeste	Masculino	parda	Casado (a)	mais de 10 salários mínimos
Sudeste	Masculino	Preto	Solteiro (a)	de 4 a 10 salários mínimos
Sudeste	Feminino	Branco	Solteiro (a)	mais de 4 salários mínimos
Sudeste	Feminino	Preto	Solteiro (a)	de 1 a 4 salários mínimos
Centro-Oeste	Feminino	parda	Casado (a)	mais de 10 salários mínimos
Sudeste	Masculino	Branco	Casado (a)	de 4 a 10 salários mínimos
Sudeste	Feminino	Branco	Divorciado (a)	de 4 a 10 salários mínimos

Sudeste	Masculino	Branco	Casado (a)	de 4 a 10 salários mínimos
Nordeste	Masculino	Ignorado	Casado (a)	de 4 a 10 salários mínimos
Sudeste	Feminino	parda	Solteiro (a)	de 1 a 4 salários mínimos
Centro-Oeste	Feminino	Branco	Casado (a)	de 4 a 10 salários mínimos
Centro-Oeste	Feminino	parda	Casado (a)	de 4 a 10 salários mínimos
Sudeste	Masculino	Branco	Solteiro (a)	de 4 a 10 salários mínimos
Nordeste	Masculino	Preto	Solteiro (a)	de 4 a 10 salários mínimos
Distrito Federal	Masculino	parda	Vive com companheiro (a)	de 4 a 10 salários mínimos
Sudeste	Feminino	parda	Casado (a)	de 1 a 4 salários mínimos
Centro-Oeste	Feminino	Branco	Solteiro (a)	de 1 a 4 salários mínimos
Nordeste	Masculino	parda	Vive com companheiro (a)	de 1 a 4 salários mínimos

Sudeste	Masculino	Branco	Casado (a)	de 4 a 10 salários mínimos
Sudeste	Feminino	Branco	Solteiro (a)	de 4 a 10 salários mínimos
Centro-Oeste	Feminino	parda	Solteiro (a)	de 1 a 4 salários mínimos
Sudeste	Feminino	Branco	Casado (a)	mais de 10 salários mínimos
Sudeste	Feminino	parda	Vive com companheiro (a)	de 1 a 4 salários mínimos
Sudeste	Feminino	Branco	Casado (a)	mais de 10 salários mínimos
Sudeste	Masculino	Branco	Casado (a)	mais de 10 salários mínimos
Sudeste	Masculino	Branco	Vive com companheiro (a)	mais de 10 salários mínimos
Sudeste	Feminino	Branco	Casado (a)	mais de 10 salários mínimos
Sudeste	Feminino	Branco	Casado (a)	mais de 10 salários mínimos

Sudeste	Feminino	Branco	Casado (a)	de 4 a 10 salários mínimos
Sul	Feminino	Branco	Casado (a)	de 4 a 10 salários mínimos
Centro-Oeste	Feminino	Amarelo	Casado (a)	de 4 a 10 salários mínimos
Sudeste	Feminino	parda	Casado (a)	de 4 a 10 salários mínimos
Sudeste	Feminino	Branco	Casado (a)	mais de 10 salários mínimos
Sudeste	Masculino	Branco	Solteiro (a)	de 1 a 4 salários mínimos
Sudeste	Feminino	Branco	Solteiro (a)	até um salário mínimo
Sudeste	Masculino	Branco	Casado (a)	de 4 a 10 salários mínimos
Sudeste	Masculino	Branco	Solteiro (a)	mais de 10 salários mínimos
Sul	Masculino	parda	Casado (a)	de 4 a 10 salários mínimos
Sudeste	Feminino	Branco	Solteiro (a)	de 4 a 10 salários mínimos

Sul	Masculino	Branco	Solteiro (a)	de 4 a 10 salários mínimos
Sudeste	Masculino	Branco	Casado (a)	de 4 a 10 salários mínimos
Sudeste	Feminino	parda	Solteiro (a)	de 4 a 10 salários mínimos
Sudeste	Feminino	Branco	Casado (a)	de 1 a 4 salários mínimos
Sudeste	Feminino	Branco	Solteiro (a)	de 4 a 10 salários mínimos
Norte	Masculino	Indígeno	Solteiro (a)	mais de 4 salários mínimos
Sudeste	Feminino	Branco	Vive com companheiro (a)	mais de 10 salários mínimos
Sudeste	Feminino	Branco	Vive com companheiro (a)	de 4 a 10 salários mínimos
Sudeste	Masculino	Branco	Casado (a)	mais de 10 salários mínimos
Sudeste	Feminino	Preto	Solteiro (a)	de 1 a 4 salários mínimos
Centro-Oeste	Feminino	Branco	Casado (a)	de 4 a 10 salários mínimos

Sudeste	Feminino	parda	Vive com companheiro (a)	mais de 4 salários mínimos
Sudeste	Masculino	parda	Solteiro (a)	de 1 a 4 salários mínimos
Centro-Oeste	Masculino	Branco	Casado (a)	mais de 10 salários mínimos
Sudeste	Masculino	parda	Solteiro (a)	de 4 a 10 salários mínimos
Sudeste	Feminino	Branco	Vive com companheiro (a)	de 4 a 10 salários mínimos
Sul	Masculino	Branco	Vive com companheiro (a)	de 4 a 10 salários mínimos
Sul	Masculino	Branco	Casado (a)	mais de 10 salários mínimos
Sudeste	Feminino	Branco	Casado (a)	mais de 4 salários mínimos
Sudeste	Feminino	Branco	Divorciado (a)	de 4 a 10 salários mínimos
Sudeste	Masculino	Branco	Vive com companheiro (a)	de 4 a 10 salários mínimos

Centro-Oeste	Masculino	Branco	Casado (a)	mais de 10 salários mínimos
Sudeste	Feminino	parda	Casado (a)	mais de 10 salários mínimos
Sudeste	Feminino	Branco	Solteiro (a)	de 1 a 4 salários mínimos
Nordeste	Masculino	parda	Vive com companheiro (a)	de 4 a 10 salários mínimos
Nordeste	Feminino	Preto	Solteiro (a)	de 4 a 10 salários mínimos
Sudeste	Masculino	Ignorado	Casado (a)	mais de 10 salários mínimos
Sudeste	Masculino	Amarelo	Solteiro (a)	mais de 10 salários mínimos
Sudeste	Feminino	Branco	Casado (a)	mais de 10 salários mínimos
Centro-Oeste	Feminino	Branco	Vive com companheiro (a)	mais de 10 salários mínimos
Sudeste	Feminino	Branco	Casado (a)	mais de 10 salários mínimos
Sudeste	Masculino	Branco	Solteiro (a)	de 1 a 4 salários mínimos
Sudeste	Masculino	Branco	Divorciado (a)	mais de 10 salários mínimos

Sudeste	Feminino	Branco	Vive com companheiro (a)	mais de 4 salários mínimos
Sul	Feminino	Branco	Vive com companheiro (a)	de 4 a 10 salários mínimos
Centro-Oeste	Feminino	Branco	Solteiro (a)	de 4 a 10 salários mínimos
Sudeste	Masculino	Amarelo	Solteiro (a)	mais de 10 salários mínimos
Sudeste	Feminino	Branco	Casado (a)	mais de 10 salários mínimos
Sul	Feminino	Branco	Casado (a)	mais de 10 salários mínimos
Centro-Oeste	Masculino	Branco	Casado (a)	mais de 10 salários mínimos
Centro-Oeste	Feminino	parda	Solteiro (a)	mais de 10 salários mínimos
Distrito Federal	Masculino	Branco	Casado (a)	mais de 10 salários mínimos
Sudeste	Feminino	Preto	Vive com companheiro (a)	mais de 10 salários mínimos
Centro-Oeste	Feminino	Ignorado	Casado (a)	de 4 a 10 salários mínimos
Sudeste	Masculino	Branco	Casado (a)	mais de 10 salários mínimos

Sudeste	Feminino	Preto	Casado (a)	de 1 a 4 salários mínimos
Sudeste	Masculino	Branco	Solteiro (a)	mais de 10 salários mínimos
Sudeste	Feminino	Branco	Solteiro (a)	de 4 a 10 salários mínimos
Sudeste	Feminino	parda	Casado (a)	de 4 a 10 salários mínimos
Sudeste	Masculino	Branco	Casado (a)	mais de 10 salários mínimos
Sudeste	Feminino	Branco	Vive com companheiro (a)	de 4 a 10 salários mínimos
Sudeste	Feminino	Amarelo	Casado (a)	mais de 10 salários mínimos
Sudeste	Feminino	Branco	Vive com companheiro (a)	mais de 10 salários mínimos
Sudeste	Feminino	Branco	Solteiro (a)	de 4 a 10 salários mínimos
Nordeste	Feminino	Branco	Casado (a)	mais de 10 salários mínimos
Sudeste	Feminino	Branco	Casado (a)	de 4 a 10 salários mínimos
Nordeste	Masculino	parda	Casado (a)	de 4 a 10 salários mínimos

Sudeste	Feminino	Branco	Solteiro (a)	mais de 4 salários mínimos
Sudeste	Masculino	parda	Casado (a)	de 4 a 10 salários mínimos
Sul	Masculino	Branco	Casado (a)	de 4 a 10 salários mínimos
Nordeste	Feminino	Branco	Solteiro (a)	de 4 a 10 salários mínimos
Sudeste	Feminino	Branco	Casado (a)	de 4 a 10 salários mínimos
Centro-Oeste	Feminino	parda	Casado (a)	mais de 10 salários mínimos
Sudeste	Feminino	Branco	Solteiro (a)	de 4 a 10 salários mínimos
Sudeste	Masculino	Branco	Casado (a)	mais de 4 salários mínimos
Nordeste	Masculino	Branco	Casado (a)	mais de 10 salários mínimos
Sudeste	Masculino	Branco	Casado (a)	mais de 10 salários mínimos
Centro-Oeste	Feminino	Branco	Casado (a)	mais de 10 salários mínimos
Sudeste	Feminino	Branco	Solteiro (a)	mais de 10 salários mínimos

Sudeste	Feminino	Branco	Divorciado (a)	mais de 10 salários mínimos
Centro-Oeste	Feminino	Branco	Divorciado (a)	mais de 4 salários mínimos
Sul	Masculino	Branco	Casado (a)	de 4 a 10 salários mínimos
Sudeste	Feminino	Branco	Divorciado (a)	de 1 a 4 salários mínimos
Sudeste	Masculino	Branco	Casado (a)	mais de 4 salários mínimos
Centro-Oeste	Masculino	Branco	Casado (a)	mais de 10 salários mínimos
Sudeste	Feminino	Branco	Casado (a)	mais de 10 salários mínimos
Sudeste	Feminino	Amarelo	Casado (a)	mais de 4 salários mínimos
Sudeste	Masculino	Branco	Casado (a)	de 4 a 10 salários mínimos
Sudeste	Feminino	Branco	Casado (a)	mais de 10 salários mínimos
Sudeste	Masculino	Branco	Casado (a)	de 4 a 10 salários mínimos
Centro-Oeste	Masculino	Branco	Casado (a)	mais de 10 salários mínimos

Sudeste	Feminino	Branco	Casado (a)	mais de 10 salários mínimos
Sudeste	Masculino	Amarelo	Casado (a)	mais de 10 salários mínimos
Sul	Feminino	Branco	Divorciado (a)	mais de 10 salários mínimos
Sudeste	Feminino	parda	Solteiro (a)	mais de 10 salários mínimos
Sudeste	Feminino	Branco	Casado (a)	de 4 a 10 salários mínimos

Qu			
**Renda familiar atual:**	**Escolaridade:**	**Ocupação / Profissão:**	**Tem filhos(as)?**
mais de 10 salários mínimos	ensino superior incompleto	Relações públicas	não
de 1 a 4 salários mínimos	ensino médio completo	Estudante	não
de 1 a 4 salários mínimos	ensino superior incompleto	Estudante	não
de 1 a 4 salários mínimos	ensino superior incompleto	Designer	não
de 1 a 4 salários mínimos	ensino superior completo	Publicitário	não
de 1 a 4 salários mínimos	ensino superior incompleto	Garçom	não
de 1 a 4 salários mínimos	ensino superior incompleto	Estudante	não
de 1 a 4 salários mínimos	ensino superior incompleto	Analista	não
de 1 a 4 salários mínimos	ensino superior incompleto	Analista	não

de 4 a 10 salários mínimos	ensino superior incompleto	Servid or Público	não
de 4 a 10 salários mínimos	ensino superior completo	Progra mador	não
mais de 10 salários mínimos	ensino superior completo	Psicólo go	não
de 4 a 10 salários mínimos	ensino superior incompleto		não
de 4 a 10 salários mínimos	ensino superior completo	Profes sor	não
mais de 10 salários mínimos	ensino superior completo		não
mais de 10 salários mínimos	ensino superior incompleto	Analist a	não
mais de 4 salários mínimos	ensino superior completo	Psicólo go	não
mais de 10 salários mínimos	ensino superior completo	Analist a	não
de 1 a 4 salários mínimos	ensino superior completo	Fisioter apueta	não
mais de 10 salários mínimos	ensino superior completo	Empre sario	não

até um salário mínimo	ensino superior incompleto	Redato r	não
mais de 4 salários mínimos	ensino superior completo	Progra mador	não
de 4 a 10 salários mínimos	ensino superior completo	Fisioter apeuta	não
de 4 a 10 salários mínimos	ensino superior completo	Estagi ário	não
de 1 a 4 salários mínimos	ensino superior completo	Advog ado	não
de 1 a 4 salários mínimos	ensino superior incompleto	Estagi ário	não
de 1 a 4 salários mínimos	ensino superior incompleto	Engen heiro	não
mais de 10 salários mínimos	ensino superior completo	Cirurgi ã Dentist a	não
de 1 a 4 salários mínimos	ensino superior completo	Pós gradua ndo	não
de 4 a 10 salários mínimos	ensino superior completo	Engen heiro	não

mais de 10 salários mínimos	ensino superior completo	Cientis ta de dados	não
de 4 a 10 salários mínimos	ensino superior completo	Analist a	não
mais de 4 salários mínimos	ensino superior completo	Advog ado	não
de 1 a 4 salários mínimos	ensino superior completo	Desen volved or	não
de 1 a 4 salários mínimos	ensino superior incompleto	Admini stração	não
de 4 a 10 salários mínimos	ensino superior completo	Analist a	não
de 4 a 10 salários mínimos	ensino superior completo	Engen heiro	não
de 4 a 10 salários mínimos	ensino superior incompleto	Estuda nte	não
de 1 a 4 salários mínimos	ensino superior completo	Jornali sta	não
mais de 10 salários mínimos	ensino superior completo	Desen volved or	não
de 4 a 10 salários mínimos	ensino superior incompleto	Estagi ário	não
de 1 a 4 salários mínimos	ensino superior completo	Progra mador	não

de 4 a 10 salários mínimos	ensino superior completo	Profes sor	não
de 4 a 10 salários mínimos	ensino superior completo	Advog ado	não
de 1 a 4 salários mínimos	ensino superior completo	Advog ado	não
de 4 a 10 salários mínimos	ensino superior completo	Engen heiro	não
de 4 a 10 salários mínimos	ensino superior completo	Engen heiro	não
mais de 4 salários mínimos	ensino superior completo	Pós gradua ndo	não
de 4 a 10 salários mínimos	ensino superior incompleto	Estuda nte	não
mais de 4 salários mínimos	ensino superior completo	Servid or Público	não
de 4 a 10 salários mínimos	ensino superior completo	Cinegr afista	não
de 1 a 4 salários mínimos	ensino superior completo	Profes sor	não

de 1 a 4 salários mínimos	ensino superior incompleto	Estuda nte	não
de 1 a 4 salários mínimos	ensino superior completo	Tatuad ora	não
de 4 a 10 salários mínimos	ensino superior completo	Engen heiro	não
de 4 a 10 salários mínimos	ensino superior completo	Psicólo go	não
de 4 a 10 salários mínimos	ensino superior completo	Person al trainer	não
de 1 a 4 salários mínimos	ensino superior completo	Engen heiro	não
de 4 a 10 salários mínimos	ensino superior completo	Progra mador	não
de 4 a 10 salários mínimos	ensino superior incompleto	Desen volved or	não
mais de 4 salários mínimos	ensino superior completo	Engen heiro	não
de 4 a 10 salários mínimos	ensino superior completo	Militar	não
de 4 a 10 salários mínimos	ensino superior completo	Servid or Público	não

de 1 a 4 salários mínimos	ensino superior completo	Farma<br>cêutico	não
de 1 a 4 salários mínimos	ensino superior completo	Analist<br>a	não
de 1 a 4 salários mínimos	ensino superior completo	Fisioter<br>apeuta	não
de 4 a 10 salários mínimos	ensino superior incompleto	Engen<br>heiro	não
mais de 10 salários mínimos	ensino superior completo	Analist<br>a	não
de 4 a 10 salários mínimos	ensino superior completo	Analist<br>a	não
de 4 a 10 salários mínimos	ensino superior completo	Empre<br>sario	não
de 4 a 10 salários mínimos	ensino superior incompleto		não
mais de 10 salários mínimos	ensino superior completo	Advog<br>ado	não
de 1 a 4 salários mínimos	ensino superior incompleto		não
de 4 a 10 salários mínimos	ensino superior completo	Engen<br>heiro	não

de 1 a 4 salários mínimos	ensino superior completo	Advogado	não
de 1 a 4 salários mínimos	ensino superior completo	Advogado	não
de 4 a 10 salários mínimos	ensino superior completo	Analista	não
de 1 a 4 salários mínimos	ensino superior completo	Psicólogo	não
mais de 4 salários mínimos	ensino superior completo	Servidor Público	não
de 1 a 4 salários mínimos	ensino superior completo	Professor	não
de 1 a 4 salários mínimos	ensino superior completo	#REF!	não
mais de 10 salários mínimos	ensino superior completo	Profissional nível Técnico	sim
mais de 10 salários mínimos	ensino superior completo	Professor	não
de 1 a 4 salários mínimos	ensino superior completo	Pesquisador	sim
de 4 a 10 salários mínimos	ensino superior completo	Advogado	não
de 1 a 4 salários mínimos	ensino superior completo	Relações Públicas	sim

de 1 a 4 salários mínimos	ensino superior completo	Profes sor	não
de 4 a 10 salários mínimos	ensino superior completo		não
de 1 a 4 salários mínimos	ensino superior completo	Design er	não
mais de 10 salários mínimos	ensino superior incompleto	Admini stração	não
mais de 10 salários mínimos	ensino superior completo	Estuda nte	não
de 1 a 4 salários mínimos	ensino superior completo	Fisioter apueta	não
mais de 10 salários mínimos	ensino superior completo	Engen heiro	não
de 4 a 10 salários mínimos	ensino superior completo	Traine e	não
mais de 10 salários mínimos	ensino superior completo	Engen heiro	não
até um salário mínimo	ensino superior completo	Profes sor	não
mais de 10 salários mínimos	ensino superior completo	Profes sor	não
de 4 a 10 salários mínimos	ensino superior completo	Farma cêutico	não

mais de 10 salários mínimos	ensino superior completo	Psicólogo	não
de 4 a 10 salários mínimos	ensino superior incompleto	Engenheiro	não
de 1 a 4 salários mínimos	ensino superior incompleto	Confeiteira	sim
de 4 a 10 salários mínimos	ensino superior completo	Profissional de TI	não
de 4 a 10 salários mínimos	ensino superior completo	Consultor	não
de 1 a 4 salários mínimos	ensino superior completo	Professor	não
mais de 10 salários mínimos	ensino superior completo	Engenheiro	não
de 4 a 10 salários mínimos	ensino superior completo	Estudante	não
de 4 a 10 salários mínimos	ensino superior completo	Programador	não
de 4 a 10 salários mínimos	ensino superior completo	Designer	não
mais de 10 salários mínimos	ensino superior completo	Socióloga	não

mais de 10 salários mínimos	ensino superior completo		não
de 1 a 4 salários mínimos	ensino superior completo	Profes sor	não
de 4 a 10 salários mínimos	ensino superior completo		não
de 1 a 4 salários mínimos	ensino superior completo	Advog ado	não
mais de 4 salários mínimos	ensino médio completo	Analist a	não
mais de 4 salários mínimos	ensino superior completo	Farma cêutico	não
de 1 a 4 salários mínimos	ensino superior completo	Coord de Marketi ng Digital	não
de 4 a 10 salários mínimos	ensino superior completo	Admini strador	não
de 4 a 10 salários mínimos	ensino superior completo	Advog ado	não
de 1 a 4 salários mínimos	ensino superior incompleto	Analist a	sim

de 1 a 4 salários mínimos	ensino superior completo	Fisioter apueta	não
de 1 a 4 salários mínimos	ensino superior completo	Fisioter apueta	não
de 4 a 10 salários mínimos	ensino superior completo	Gerent e Admini strativo	não
mais de 10 salários mínimos	ensino superior completo	Consul tor	não
de 4 a 10 salários mínimos	ensino superior completo	Assiste nte Admini strativo	não
mais de 10 salários mínimos	ensino superior completo	Psicólo go	sim
mais de 4 salários mínimos	ensino superior completo	Profes sor	não
de 4 a 10 salários mínimos	ensino superior completo	Profes sor	sim
de 4 a 10 salários mínimos	ensino superior completo	Biólog o	não
de 1 a 4 salários mínimos	ensino superior completo	Profes sor	não
mais de 4 salários mínimos	ensino superior completo	CS	sim

de 1 a 4 salários mínimos	ensino superior completo	Pós graduando	não
de 1 a 4 salários mínimos	ensino superior completo	Jornalista	não
mais de 10 salários mínimos	ensino superior completo	Desenvolvedor	não
mais de 10 salários mínimos	ensino superior completo	Advogado	não
mais de 10 salários mínimos	ensino superior completo	Psicólogo	não
de 4 a 10 salários mínimos	ensino superior completo	Fisioterapeuta	não
de 1 a 4 salários mínimos	ensino superior incompleto		não
de 1 a 4 salários mínimos	ensino superior completo	Professor	não
de 1 a 4 salários mínimos	ensino superior incompleto	Professor	não
de 1 a 4 salários mínimos	ensino superior completo		sim
de 4 a 10 salários mínimos	ensino superior completo	Professor	não

de 4 a 10 salários mínimos	ensino superior completo	Pesqui sador	não
de 4 a 10 salários mínimos	ensino superior completo	Fisioter apueta	não
de 4 a 10 salários mínimos	ensino superior completo	Estuda nte	não
de 4 a 10 salários mínimos	ensino superior completo	Desen volved or	não
mais de 10 salários mínimos	ensino superior completo	Admini strador	não
de 1 a 4 salários mínimos	ensino superior completo	Fisioter apueta	sim
de 4 a 10 salários mínimos	ensino superior completo	Desen volved or	não
de 4 a 10 salários mínimos	ensino superior incompleto	Servid or Público	não
de 4 a 10 salários mínimos	ensino superior completo	Profes sor	não
mais de 10 salários mínimos	ensino superior completo	Consul tor	não
de 4 a 10 salários mínimos	ensino superior completo	Fisioter apeuta	não
de 4 a 10 salários mínimos	ensino superior completo	Profes sor	não

mais de 10 salários mínimos	ensino superior completo	Professor	não
de 4 a 10 salários mínimos	ensino superior completo	Professor	não
mais de 10 salários mínimos	ensino superior completo	Fisioterapeuta	não
mais de 10 salários mínimos	ensino superior completo		não
mais de 10 salários mínimos	ensino superior completo	Bancário	sim
mais de 10 salários mínimos	ensino superior completo	Administração	sim
mais de 10 salários mínimos	ensino superior incompleto		não
de 4 a 10 salários mínimos	ensino superior completo	Servidor Público	não
de 4 a 10 salários mínimos	ensino superior completo	Professor	não
mais de 4 salários mínimos	ensino superior incompleto	Analista	não
de 4 a 10 salários mínimos	ensino superior completo	Publicitário	não
de 4 a 10 salários mínimos	ensino superior completo	Gerente de produtos	não

de 1 a 4 salários mínimos	ensino superior completo	Profissi onal nível Técnic o	não
mais de 10 salários mínimos	ensino superior completo	Advog ado	sim
de 1 a 4 salários mínimos	ensino superior incompleto	Analist a	sim
de 4 a 10 salários mínimos	ensino superior completo	Engen heiro	não
de 4 a 10 salários mínimos	ensino superior completo	Fisioter apeuta	não
de 4 a 10 salários mínimos	ensino superior completo	Desen volved or	não
mais de 10 salários mínimos	ensino superior completo		não
mais de 10 salários mínimos	ensino superior completo		não
de 4 a 10 salários mínimos	ensino superior completo	Engen heiro	não
de 4 a 10 salários mínimos	ensino superior completo	Engen heiro	não
mais de 10 salários mínimos	ensino superior completo	Publicit ário	não

mais de 10 salários mínimos	ensino superior completo	Gerente de Subscrição-Seguradora	não
mais de 10 salários mínimos	ensino superior completo	Engenheiro	não
de 1 a 4 salários mínimos	ensino superior completo	Secretariado	não
mais de 4 salários mínimos	ensino superior completo	Professor	não
de 1 a 4 salários mínimos	ensino superior completo	Professor	sim
mais de 4 salários mínimos	ensino superior completo	Assistente social	não
mais de 10 salários mínimos	ensino superior completo	Profissional de TI	não
de 1 a 4 salários mínimos	ensino superior completo	Empresario	não
mais de 10 salários mínimos	ensino superior completo	Professor	não
de 4 a 10 salários mínimos	ensino superior completo	Enfermeiro	não
de 4 a 10 salários mínimos	ensino superior completo	Analista	sim

de 4 a 10 salários mínimos	ensino superior completo	Admini<br>stração	não
mais de 10 salários mínimos	ensino superior completo	Engen<br>heiro	sim
mais de 10 salários mínimos	ensino superior incompleto	Marketi<br>ng	sim
mais de 10 salários mínimos	ensino superior completo		não
de 4 a 10 salários mínimos	ensino superior completo	Estatís<br>tico	não
mais de 10 salários mínimos	ensino superior completo	Bancár<br>io	não
de 1 a 4 salários mínimos	ensino superior completo	Servid<br>or<br>Público	sim
mais de 10 salários mínimos	ensino superior completo	Profes<br>sor	não
de 4 a 10 salários mínimos	ensino superior completo	Engen<br>heiro	sim
mais de 10 salários mínimos	ensino superior completo	Psicólo<br>go	não
de 4 a 10 salários mínimos	ensino superior completo	Servid<br>or<br>Público	não

mais de 10 salários mínimos	ensino médio completo	Progra mador	não
de 1 a 4 salários mínimos	ensino superior completo	Psicólo go	sim
de 1 a 4 salários mínimos	ensino superior completo	Profes sor	não
mais de 10 salários mínimos	ensino superior completo	Servid or Público	não
de 4 a 10 salários mínimos	ensino superior completo	Progra mador	não
de 4 a 10 salários mínimos	ensino superior completo	Empre sario	sim
de 4 a 10 salários mínimos	ensino superior completo	Profes sor	não
mais de 4 salários mínimos	ensino superior completo	Psicólo go	não
de 1 a 4 salários mínimos	ensino superior completo	Profes sor	não
mais de 10 salários mínimos	ensino superior completo	Servid or Público	sim
de 4 a 10 salários mínimos	ensino superior completo	Cientis ta Ambie ntal	não
de 1 a 4 salários mínimos	ensino superior completo	Fisioter apueta	não

de 4 a 10 salários mínimos	ensino superior completo	Compr ador	sim
de 4 a 10 salários mínimos	ensino médio completo	Profes sor	não
de 1 a 4 salários mínimos	ensino médio completo	Analist a	não
mais de 10 salários mínimos	ensino superior completo	Profes sor	sim
de 4 a 10 salários mínimos	ensino superior completo	Empre ended ora	sim
de 4 a 10 salários mínimos	ensino superior completo	Ocean ógrafo	não
de 4 a 10 salários mínimos	ensino superior completo	Engen heiro	não
mais de 10 salários mínimos	ensino superior completo	Desen volved or	sim
de 1 a 4 salários mínimos	ensino superior completo	Profes sor	não
de 1 a 4 salários mínimos	ensino superior completo	Fisioter apueta	não
de 1 a 4 salários mínimos	ensino superior completo	Profissi onal de TI	sim

de 4 a 10 salários mínimos	ensino superior completo	Servid or Público	sim
de 4 a 10 salários mínimos	ensino superior completo	Advog ado	não
de 1 a 4 salários mínimos	ensino superior completo	Fisioter apeuta	não
mais de 10 salários mínimos	ensino superior completo	Profes sor	sim
de 1 a 4 salários mínimos	ensino superior completo	Profes sor	não
mais de 10 salários mínimos	ensino superior completo	Comun icóloga	sim
mais de 10 salários mínimos	ensino superior incompleto	Progra mador	sim
mais de 10 salários mínimos	ensino superior completo		não
mais de 10 salários mínimos	ensino superior completo	Enferm eiro	sim
mais de 10 salários mínimos	ensino superior completo	Profes sor	não

de 4 a 10 salários mínimos	ensino superior completo	Pedag oga	não
de 4 a 10 salários mínimos	ensino superior completo	Profes sor	sim
de 4 a 10 salários mínimos	ensino superior completo	Biólog o	sim
de 4 a 10 salários mínimos	ensino superior completo	Admini stração	sim
mais de 10 salários mínimos	ensino superior completo	Pesqui sador	não
de 1 a 4 salários mínimos	ensino médio completo	Músico	não
até um salário mínimo	ensino superior completo	Profes sor	não
de 4 a 10 salários mínimos	ensino superior completo	Arqueó logo	sim
mais de 10 salários mínimos	ensino superior completo	Advog ado	não
de 4 a 10 salários mínimos	ensino superior completo		não
de 4 a 10 salários mínimos	ensino superior completo	Servid or Público	sim

de 4 a 10 salários mínimos	ensino superior completo	Profes sor	não
de 4 a 10 salários mínimos	ensino superior completo	Assiste nte Admini strativo	não
de 4 a 10 salários mínimos	ensino superior completo	Profes sor	sim
de 1 a 4 salários mínimos	ensino superior completo	Profes sor	sim
de 4 a 10 salários mínimos	ensino superior completo	Pedag oga	sim
mais de 4 salários mínimos	ensino superior completo	Servid or Público	não
de 4 a 10 salários mínimos	ensino superior completo	Profes sor	não
de 4 a 10 salários mínimos	ensino superior completo	Advog ado	sim
mais de 10 salários mínimos	ensino superior completo	Engen heiro	não
de 1 a 4 salários mínimos	ensino superior completo	Profes sor	não
de 4 a 10 salários mínimos	ensino superior completo	Servid or Público	não

de 4 a 10 salários mínimos	ensino superior completo	Servid or Público	não
de 1 a 4 salários mínimos	ensino superior incompleto	Analist a	não
mais de 10 salários mínimos	ensino superior completo	Advog ado	sim
de 4 a 10 salários mínimos	ensino superior completo	Enferm eiro	não
de 4 a 10 salários mínimos	ensino superior completo	nador de Planej ament o Financ	não
mais de 10 salários mínimos	ensino superior completo	Desen volved or	não
mais de 10 salários mínimos	ensino superior completo	Empre sario	sim
de 1 a 4 salários mínimos	ensino superior completo	Profes sor	sim
de 4 a 10 salários mínimos	ensino superior completo	Profes sor	sim
mais de 10 salários mínimos	ensino superior completo	Editor de livros	não

mais de 10 salários mínimos	ensino superior completo	Fisioter apeuta	não
mais de 10 salários mínimos	ensino superior completo	Analist a	sim
de 1 a 4 salários mínimos	ensino superior completo	produt ora gráfica	não
de 4 a 10 salários mínimos	ensino superior completo		sim
de 4 a 10 salários mínimos	ensino superior completo	Profes sor	não
mais de 10 salários mínimos	ensino superior completo	Engen heiro	sim
mais de 10 salários mínimos	ensino superior completo	Bancár io	não
mais de 10 salários mínimos	ensino superior completo	Psicólo go	sim
mais de 10 salários mínimos	ensino superior completo	Profes sor	não
mais de 10 salários mínimos	ensino superior completo	Gestão de projeto s	sim
de 1 a 4 salários mínimos	ensino superior completo	Desen volved or	não
de 4 a 10 salários mínimos	ensino superior completo	Vende dor	não

de 1 a 4 salários mínimos	ensino superior completo	Profes sor	sim
de 4 a 10 salários mínimos	ensino superior completo	Assiste nte Admini strativo	não
de 4 a 10 salários mínimos	ensino superior completo	Profes sor	não
mais de 10 salários mínimos	ensino superior completo	Profes sor	não
de 4 a 10 salários mínimos	ensino superior incompleto	Empre sario	sim
mais de 10 salários mínimos	ensino superior completo	Profes sor	sim
mais de 10 salários mínimos	ensino superior completo	Fisioter apeuta	sim
mais de 10 salários mínimos	ensino superior completo	Profes sor	não
mais de 10 salários mínimos	ensino superior completo	Analist a	não
mais de 10 salários mínimos	ensino superior incompleto		não
de 4 a 10 salários mínimos	ensino superior completo	Profes sor	sim
mais de 10 salários mínimos	ensino superior completo	Bancár io	sim

de 1 a 4 salários mínimos	ensino superior completo	Profes sor	sim
mais de 10 salários mínimos	ensino superior completo	Profes sor	não
de 4 a 10 salários mínimos	ensino superior completo	Servid or Público	não
de 4 a 10 salários mínimos	ensino superior completo	Gestor a Comer cial	sim
mais de 10 salários mínimos	ensino superior completo	Gerent e	sim
de 4 a 10 salários mínimos	ensino superior completo	Servid or Público	não
mais de 10 salários mínimos	ensino superior completo	Servid or Público	sim
mais de 10 salários mínimos	ensino superior completo	Profes sor	não
de 4 a 10 salários mínimos	ensino superior completo	Profes sor	não
mais de 10 salários mínimos	ensino superior completo	Fisioter apeuta	sim
de 4 a 10 salários mínimos	ensino superior completo	Escrev ente	sim
mais de 10 salários mínimos	ensino superior completo	Profes sor	não

mais de 4 salários mínimos	ensino superior completo	Pós graduando	não
de 4 a 10 salários mínimos	ensino superior completo	CONTABILISTA	sim
mais de 10 salários mínimos	ensino superior completo	Biólogo	não
de 4 a 10 salários mínimos	ensino superior completo	Professor	sim
mais de 10 salários mínimos	ensino superior completo	Psicólogo	não
mais de 10 salários mínimos	ensino superior completo	Professor	sim
de 4 a 10 salários mínimos	ensino superior completo	geóloga	não
mais de 4 salários mínimos	ensino superior completo	Web Designer	não
mais de 10 salários mínimos	ensino superior completo	Professor	sim
mais de 10 salários mínimos	ensino superior completo	Servidor Público	não
mais de 10 salários mínimos	ensino superior completo	Fisioterapueta	sim
mais de 10 salários mínimos	ensino superior completo	Professor	não

mais de 10 salários mínimos	ensino superior completo	Profes sor	sim
mais de 4 salários mínimos	ensino superior completo	Profes sor	sim
de 4 a 10 salários mínimos	ensino superior completo	Desen volved or	sim
de 1 a 4 salários mínimos	ensino superior completo	Servid or Público	sim
de 4 a 10 salários mínimos	ensino superior completo	Apose ntado	sim
mais de 10 salários mínimos	ensino superior completo	Fisioter apeuta	sim
mais de 10 salários mínimos	ensino superior completo	Profes sor	sim
de 4 a 10 salários mínimos	ensino superior incompleto	Bancár io	sim
mais de 10 salários mínimos	ensino superior completo	Engen heiro	sim
mais de 10 salários mínimos	ensino superior completo	Profes sor	sim
de 4 a 10 salários mínimos	ensino superior completo	Profes sor	sim
mais de 10 salários mínimos	ensino superior completo	Profes sor	sim

mais de 10 salários mínimos	ensino superior completo	Profes sor	sim
mais de 10 salários mínimos	ensino superior completo	Servid or Público	sim
mais de 10 salários mínimos	ensino superior completo	Engen heiro	sim
mais de 10 salários mínimos	ensino superior incompleto	Pesqui sador	sim
de 4 a 10 salários mínimos	ensino superior completo	Profes sor	sim

estionário para a avaliação sociodemográ

Com quem mora:	Praticava atividade física antes da pandemia?	Pratica atividade física durante a pandemia?
Com 2 ou mais pessoas	Sim	Não
Com uma pessoa	Não	Sim
Com 2 ou mais pessoas	Sim	Sim
Sozinho	Não	Não
Com uma pessoa	Sim	Não
Com 2 ou mais pessoas	Não	Sim
Com uma pessoa	Não	Não
Sozinho	Não	Sim
Com uma pessoa	Não	Sim

Com 2 ou mais pessoas	Não	Sim
Com 2 ou mais pessoas	Não	Não
Com 2 ou mais pessoas	Sim	Sim
Com uma pessoa	Sim	Sim
Com 2 ou mais pessoas	Não	Não
Com 2 ou mais pessoas	Não	Não
Com 2 ou mais pessoas	Sim	Não
Com uma pessoa	Não	Sim
Com 2 ou mais pessoas	Sim	Não
Com 2 ou mais pessoas	Não	Sim
Com uma pessoa	Sim	Sim

Com 2 ou mais pessoas	Não	Não
Com 2 ou mais pessoas	Não	Sim
Com uma pessoa	Sim	Sim
Com uma pessoa	Sim	Sim
Com 2 ou mais pessoas	Não	Não
Com uma pessoa	Sim	Não
Sozinho	Sim	Não
Com 2 ou mais pessoas	Sim	Não
Com 2 ou mais pessoas	Não	Não
Com uma pessoa	Sim	Sim

Com uma pessoa	Sim	Não
Com 2 ou mais pessoas	Sim	Não
Com 2 ou mais pessoas	Sim	Não
Com 2 ou mais pessoas	Sim	Sim
Com uma pessoa	Não	Não
Com 2 ou mais pessoas	Sim	Sim
Com uma pessoa	Não	Não
Com 2 ou mais pessoas	Sim	Não
Com uma pessoa	Sim	Não
Sozinho	Sim	Sim
Com 2 ou mais pessoas	Sim	Não
Sozinho	Sim	Sim

Com uma pessoa	Sim	Não
Com 2 ou mais pessoas	Sim	Não
Com uma pessoa	Sim	Sim
Com uma pessoa	Sim	Sim
Com 2 ou mais pessoas	Sim	Não
Com uma pessoa	Não	Sim
Com 2 ou mais pessoas	Sim	Sim
Sozinho	Sim	Não
Com 2 ou mais pessoas	Sim	Sim
Sozinho	Sim	Sim

Com uma pessoa	Não	Sim
Sozinho	Sim	Não
Com 2 ou mais pessoas	Sim	Sim
Com uma pessoa	Não	Sim
Com 2 ou mais pessoas	Sim	Sim
Com uma pessoa	Sim	Não
Com uma pessoa	Sim	Sim
Com 2 ou mais pessoas	Não	Não
Com uma pessoa	Sim	Sim
Sozinho	Sim	Sim
Com uma pessoa	Não	Não

Com 2 ou mais pessoas	Sim	Não
Com uma pessoa	Não	Não
Com uma pessoa	Não	Sim
Com 2 ou mais pessoas	Sim	Não
Com uma pessoa	Não	Sim
Com uma pessoa	Não	Sim
Com uma pessoa	Sim	Sim
Com uma pessoa	Sim	Sim
Com uma pessoa	Não	Não
Com 2 ou mais pessoas	Não	Não
Com uma pessoa	Sim	Não

Com uma pessoa	Sim	Sim
Com 2 ou mais pessoas	Não	Sim
Com uma pessoa	Não	Não
Com 2 ou mais pessoas	Não	Sim
Com uma pessoa	Não	Sim
Com uma pessoa	Sim	Sim
Com 2 ou mais pessoas	Sim	Não
Com 2 ou mais pessoas	Sim	Não
Com 2 ou mais pessoas	Sim	Sim
Com 2 ou mais pessoas	Não	Não
Com 2 ou mais pessoas	Sim	Não
Com 2 ou mais pessoas	Sim	Sim

Com uma pessoa	Não	Sim
Com uma pessoa	Sim	Sim
Sozinho	Sim	Não
Com uma pessoa	Não	Não
Com 2 ou mais pessoas	Sim	Sim
Com 2 ou mais pessoas	Não	Não
Com 2 ou mais pessoas	Sim	Sim
Com uma pessoa	Sim	Não
Com 2 ou mais pessoas	Não	Sim
Com uma pessoa	Sim	Não
Com 2 ou mais pessoas	Sim	Sim
Com uma pessoa	Sim	Sim

Com uma pessoa	Sim	Sim
Com uma pessoa	Não	Não
Com 2 ou mais pessoas	Não	Sim
Com uma pessoa	Não	Não
Com uma pessoa	Não	Não
Sozinho	Não	Sim
Com uma pessoa	Sim	Não
Com uma pessoa	Sim	Sim
Sozinho	Sim	Não
Com uma pessoa	Sim	Não
Com 2 ou mais pessoas	Não	Sim

Com uma pessoa	Sim	Não
Com uma pessoa	Sim	Sim
Com uma pessoa	Sim	Sim
Sozinho	Sim	Sim
Com uma pessoa	Não	Não
Com uma pessoa	Sim	Sim
Com uma pessoa	Não	Não
Com 2 ou mais pessoas	Não	Sim
Com uma pessoa	Sim	Sim
Com 2 ou mais pessoas	Sim	Não

Com uma pessoa	Sim	Sim
Com uma pessoa	Não	Sim
Com uma pessoa	Sim	Sim
Com 2 ou mais pessoas	Sim	Não
Com uma pessoa	Não	Não
Com 2 ou mais pessoas	Sim	Sim
Com uma pessoa	Não	Sim
Com 2 ou mais pessoas	Sim	Sim
Com uma pessoa	Sim	Sim
Sozinho	Não	Não
Com 2 ou mais pessoas	Não	Não

Com 2 ou mais pessoas	Sim	Sim
Com uma pessoa	Sim	Não
Com 2 ou mais pessoas	Sim	Sim
Sozinho	Não	Sim
Com uma pessoa	Não	Não
Com 2 ou mais pessoas	Sim	Sim
Com uma pessoa	Não	Sim
Sozinho	Não	Não
Com 2 ou mais pessoas	Sim	Sim
Com uma pessoa	Sim	Não
Com 2 ou mais pessoas	Não	Não

Com uma pessoa	Sim	Não
Com uma pessoa	Não	Não
Com 2 ou mais pessoas	Não	Sim
Com uma pessoa	Sim	Não
Com 2 ou mais pessoas	Sim	Sim
Com 2 ou mais pessoas	Sim	Sim
Sozinho	Sim	Sim
Com uma pessoa	Não	Não
Com 2 ou mais pessoas	Sim	Sim
Com uma pessoa	Sim	Não
Com uma pessoa	Sim	Sim
Com uma pessoa	Não	Não

Com 2 ou mais pessoas	Sim	Sim
Com uma pessoa	Sim	Sim
Com uma pessoa	Não	Não
Com uma pessoa	Não	Sim
Com 2 ou mais pessoas	Não	Não
Com 2 ou mais pessoas	Sim	Sim
Com uma pessoa	Não	Sim
Com 2 ou mais pessoas	Sim	Sim
Com uma pessoa	Não	Sim
Sozinho	Não	Não
Sozinho	Sim	Não
Sozinho	Sim	Sim

Com 2 ou mais pessoas	Sim	Sim
Com 2 ou mais pessoas	Sim	Sim
Com 2 ou mais pessoas	Não	Não
Com uma pessoa	Sim	Sim
Com 2 ou mais pessoas	Sim	Sim
Sozinho	Não	Não
Com uma pessoa	Sim	Sim
Sozinho	Sim	Sim
Com 2 ou mais pessoas	Sim	Não
Com 2 ou mais pessoas	Sim	Sim
Com uma pessoa	Sim	Sim

Com 2 ou mais pessoas	Sim	Não
Com 2 ou mais pessoas	Não	Não
Sozinho	Sim	Sim
Com uma pessoa	Sim	Sim
Com 2 ou mais pessoas	Sim	Não
Com 2 ou mais pessoas	Sim	Não
Com uma pessoa	Sim	Sim
Com uma pessoa	Sim	Sim
Com uma pessoa	Sim	Sim
Com uma pessoa	Não	Sim
Com 2 ou mais pessoas	Sim	Não

Com uma pessoa	Sim	Não
Com 2 ou mais pessoas	Não	Sim
Com 2 ou mais pessoas	Não	Não
Com uma pessoa	Não	Não
Sozinho	Não	Não
Com uma pessoa	Sim	Não
Com uma pessoa	Sim	Não
Com uma pessoa	Sim	Sim
Com 2 ou mais pessoas	Sim	Não
Com uma pessoa	Sim	Sim
Com uma pessoa	Não	Não

Com uma pessoa	Não	Não
Com 2 ou mais pessoas	Sim	Não
Com uma pessoa	Sim	Sim
Com uma pessoa	Sim	Sim
Sozinho	Não	Sim
Com 2 ou mais pessoas	Sim	Não
Com uma pessoa	Sim	Não
Com 2 ou mais pessoas	Não	Sim
Com 2 ou mais pessoas	Sim	Não
Com 2 ou mais pessoas	Sim	Sim
Com uma pessoa	Sim	Sim
Com 2 ou mais pessoas	Sim	Sim

Com 2 ou mais pessoas	Não	Não
Com uma pessoa	Não	Não
Com 2 ou mais pessoas	Não	Não
Com 2 ou mais pessoas	Não	Não
Com 2 ou mais pessoas	Sim	Sim
Sozinho	Sim	Sim
Com 2 ou mais pessoas	Sim	Não
Com 2 ou mais pessoas	Não	Sim
Com uma pessoa	Sim	Não
Sozinho	Sim	Sim
Com 2 ou mais pessoas	Não	Não

Com 2 ou mais pessoas	Sim	Não
Com 2 ou mais pessoas	Sim	Não
Com 2 ou mais pessoas	Sim	Sim
Com 2 ou mais pessoas	Não	Não
Com uma pessoa	Sim	Sim
Com 2 ou mais pessoas	Não	Não
Com 2 ou mais pessoas	Sim	Não
Com uma pessoa	Sim	Sim
Com 2 ou mais pessoas	Sim	Não
Com uma pessoa	Sim	Não

Com uma pessoa	Sim	Sim
Com 2 ou mais pessoas	Não	Não
Com 2 ou mais pessoas	Sim	Sim
Com 2 ou mais pessoas	Não	Sim
Com uma pessoa	Sim	Não
Sozinho	Não	Sim
Com uma pessoa	Não	Não
Com 2 ou mais pessoas	Não	Não
Com uma pessoa	Não	Não
Com uma pessoa	Sim	Sim
Com uma pessoa	Sim	Sim

Sozinho	Não	Sim
Com uma pessoa	Sim	Sim
Com uma pessoa	Não	Não
Com 2 ou mais pessoas	Sim	Sim
Com 2 ou mais pessoas	Sim	Sim
Com 2 ou mais pessoas	Sim	Sim
Com 2 ou mais pessoas	Sim	Não
Com 2 ou mais pessoas	Sim	Não
Com uma pessoa	Não	Não
Sozinho	Sim	Não
Com uma pessoa	Sim	Não

Com 2 ou mais pessoas	Não	Não
Sozinho	Não	Sim
Com 2 ou mais pessoas	Sim	Sim
Sozinho	Não	Não
Com uma pessoa	Sim	Não
Com uma pessoa	Não	Não
Com 2 ou mais pessoas	Sim	Sim
Com 2 ou mais pessoas	Sim	Não
Com uma pessoa	Sim	Não
Com uma pessoa	Não	Não

Com uma pessoa	Sim	Sim
Com 2 ou mais pessoas	Sim	Sim
Sozinho	Não	Sim
Com 2 ou mais pessoas	Não	Não
Com uma pessoa	Sim	Sim
Com 2 ou mais pessoas	Sim	Sim
Com 2 ou mais pessoas	Sim	Sim
Com 2 ou mais pessoas	Sim	Sim
Com uma pessoa	Sim	Não
Com 2 ou mais pessoas	Sim	Sim
Com 2 ou mais pessoas	Sim	Não
Sozinho	Não	Sim

Com 2 ou mais pessoas	Sim	Não
Com 2 ou mais pessoas	Não	Não
Com uma pessoa	Não	Sim
Com uma pessoa	Não	Não
Com 2 ou mais pessoas	Sim	Não
Com 2 ou mais pessoas	Não	Não
Com 2 ou mais pessoas	Não	Sim
Com 2 ou mais pessoas	Sim	Sim
Com uma pessoa	Sim	Sim
Com uma pessoa	Não	Não
Com 2 ou mais pessoas	Não	Sim
Com 2 ou mais pessoas	Sim	Sim

Com 2 ou mais pessoas	Sim	Não
Sozinho	Sim	Sim
Sozinho	Não	Não
Com 2 ou mais pessoas	Sim	Sim
Com 2 ou mais pessoas	Não	Sim
Com uma pessoa	Sim	Não
Com 2 ou mais pessoas	Não	Sim
Com uma pessoa	Sim	Não
Sozinho	Sim	Sim
Com 2 ou mais pessoas	Sim	Sim
Com 2 ou mais pessoas	Sim	Sim
Com 2 ou mais pessoas	Não	Sim

Com uma pessoa	Sim	Sim
Com 2 ou mais pessoas	Sim	Sim
Com 2 ou mais pessoas	Não	Não
Com uma pessoa	Sim	Sim
Com uma pessoa	Sim	Sim
Com 2 ou mais pessoas	Não	Sim
Com uma pessoa	Sim	Não
Com uma pessoa	Sim	Sim
Com 2 ou mais pessoas	Sim	Não
Com 2 ou mais pessoas	Não	Não
Com 2 ou mais pessoas	Sim	Sim
Sozinho	Sim	Não

Com 2 ou mais pessoas	Sim	Sim
Com 2 ou mais pessoas	Não	Não
Com 2 ou mais pessoas	Não	Não
Com uma pessoa	Sim	Sim
Com 2 ou mais pessoas	Sim	Sim
Com 2 ou mais pessoas	Não	Não
Com 2 ou mais pessoas	Sim	Sim
Com uma pessoa	Não	Sim
Com 2 ou mais pessoas	Não	Não
Com uma pessoa	Sim	Sim
Com 2 ou mais pessoas	Não	Sim
Com 2 ou mais pessoas	Não	Não

Com 2 ou mais pessoas	Sim	Sim
Com 2 ou mais pessoas	Não	Não
Com 2 ou mais pessoas	Não	Não
Com 2 ou mais pessoas	Sim	Não
Com uma pessoa	Sim	Sim

áfica, de saúde, hábitos de vida e caracter

Quanto tempo (em horas) você gasta sentado durante um dia?	Tem o hábito de fumar?	Ingere bebida alcoólica?
Mais que 12 h	Não	Socialmente
Mais que 12 h	Não	Socialmente
De 8 a 10 horas	Não	Socialmente
De 8 a 10 horas	Não	Socialmente
De 10 a 12 horas	Não	Regularmente
De 4 a 6 horas	Não	Socialmente
Mais que 12 h	Não	Socialmente
De 8 a 10 horas	Não	Regularmente
De 6 a 8 horas	Não	Socialmente

De 8 a 10 horas	Não	Socialmente
De 10 a 12 horas	Não	Socialmente
De 8 a 10 horas	Não	Socialmente
Mais que 12h	Sim	Nunca
De 8 a 10 horas	Não	Nunca
Mais que 12h	Não	Socialmente
De 8 a 10 horas	Não	Socialmente
De 6 a 8 horas	Não	Socialmente
De 6 a 8 horas	Não	Nunca
De 8 a 10 horas	Não	Socialmente
De 4 a 6 horas	Não	Socialmente

Mais que 12 h	Não	Socialmente
De 10 a 12 horas	Não	Nunca
Menos que 4 horas	Não	Socialmente
De 10 a 12 horas	Não	Socialmente
Mais que 12 h	Não	Nunca
Menos que 4h	Não	Socialmente
De 8 a 10 horas	Sim	Regularmente
De 6 a 8 horas	Não	Socialmente
De 6 a 8 horas	Não	Nunca
De 8 a 10 horas	Não	Socialmente

De 8 a 10 horas	Não	Socialmente
De 8 a 10 horas	Não	Regularmente
Mais que 12 h	Não	Socialmente
Mais que 12 h	Não	Nunca
De 8 a 10 horas	Não	Regularmente
De 10 a 12 horas	Não	Regularmente
De 10 a 12 horas	Não	Socialmente
Menos que 4h	Não	Socialmente
De 10 a 12 horas	Não	Socialmente
De 8 a 10 horas	Não	Socialmente
De 8 a 10 horas	Não	Socialmente
De 8 a 10 horas	Não	Nunca

De 10 a 12 horas	Não	Nunca
De 6 a 8 horas	Não	Nunca
De 10 a 12 horas	Não	Socialmente
De 10 a 12 horas	Não	Socialmente
De 8 a 10 horas	Não	Socialmente
De 8 a 10 horas	Não	Socialmente
De 8 a 10 horas	Não	Socialmente
De 6 a 8 horas	Sim	Socialmente
De 10 a 12 horas	Não	Socialmente
De 4 a 6 horas	Não	Socialmente

De 6 a 8 horas	Não	Nunca
De 10 a 12 horas	Não	Socialmente
Mais que 12 h	Não	Socialmente
De 6 a 8 horas	Não	Regularmente
De 4 a 6 horas	Não	Socialmente
De 8 a 10 horas	Não	Socialmente
De 8 a 10 horas	Não	Socialmente
Mais que 12 h	Não	Socialmente
De 8 a 10 horas	Não	Socialmente
De 8 a 10 horas	Não	Socialmente
De 8 a 10 horas	Não	Nunca

De 6 a 8 horas	Não	Nunca
De 8 a 10 horas	Não	Nunca
De 4 a 6 horas	Não	Socialmente
De 8 a 10 horas	Não	Socialmente
De 8 a 10 horas	Não	Socialmente
De 8 a 10 horas	Não	Socialmente
De 4 a 6 horas	Não	Socialmente
De 6 a 8 horas	Não	Socialmente
De 8 a 10 horas	Não	Socialmente
De 8 a 10 horas	Não	Regularmente
De 8 a 10 horas	Não	Regularmente

De 10 a 12 horas	Não	Socialmente
De 6 a 8 horas	Não	Socialmente
De 6 a 8 horas	Não	Socialmente
De 6 a 8 horas	Não	Regularmente
De 8 a 10 horas	Não	Socialmente
De 6 a 8 horas	Não	Socialmente
Mais que 12 h	Não	Socialmente
De 8 a 10 horas	Não	Regularmente
De 8 a 10 horas	Não	Socialmente
De 6 a 8 horas	Não	Nunca
De 4 a 6 horas	Não	Nunca
De 10 a 12 horas	Não	Regularmente

De 8 a 10 horas	Não	Nunca
De 6 a 8 horas	Não	Regularmente
Mais que 12h	Não	Socialmente
De 8 a 10 horas	Não	Socialmente
De 10 a 12 horas	Não	Nunca
Mais que 12 h	Não	Socialmente
De 8 a 10 horas	Não	Regularmente
De 6 a 8 horas	Não	Socialmente
De 8 a 10 horas	Não	Regularmente
De 8 a 10 horas	Não	Socialmente
De 4 a 6 horas	Não	Socialmente
Mais que 12 h	Não	Nunca

De 6 a 8 horas	Não	Socialmente
Mais que 12 h	Sim	Socialmente
De 8 a 10 horas	Não	Socialmente
De 8 a 10 horas	Não	Nunca
Mais que 12 h	Não	Nunca
De 10 a 12 horas	Não	Socialmente
Mais que 12 h	Não	Socialmente
De 6 a 8 horas	Não	Socialmente
De 8 a 10 horas	Não	Socialmente
De 6 a 8 horas	Não	Socialmente
De 8 a 10 horas	Não	Socialmente

De 10 a 12 horas	Sim	Socialmente
De 10 a 12 horas	Não	Socialmente
De 8 a 10 horas	Não	Socialmente
De 6 a 8 horas	Não	Socialmente
De 8 a 10 horas	Não	Nunca
De 4 a 6 horas	Não	Socialmente
De 8 a 10 horas	Não	Socialmente
De 8 a 10 horas	Não	Nunca
De 6 a 8 horas	Não	Socialmente
De 10 a 12 horas	Não	Nunca

De 4 a 6 horas	Não	Socialmente
De 10 a 12 horas	Não	Socialmente
Menos que 4 horas	Não	Socialmente
Mais que 12 h	Não	Regularmente
De 4 a 6 horas	Não	Socialmente
De 8 a 10 horas	Não	Socialmente
Mais que 12 h	Não	Nunca
De 8 a 10 horas	Não	Nunca
De 8 a 10 horas	Não	Socialmente
Mais que 12 h	Não	Regularmente
De 6 a 8 horas	Não	Nunca

De 8 a 10 horas	Não	Socialmente
Mais que 12 h	Não	Regularmente
De 8 a 10 horas	Não	Regularmente
De 8 a 10 horas	Não	Regularmente
De 6 a 8 horas	Não	Socialmente
De 4 a 6 horas	Não	Socialmente
De 8 a 10 horas	Não	Nunca
De 10 a 12 horas	Não	Socialmente
De 6 a 8 horas	Não	Socialmente
De 8 a 10 horas	Não	Nunca
De 6 a 8 horas	Não	Socialmente

Mais que 12 h	Não	Socialmente
De 8 a 10 horas	Não	Regularmente
Mais que 12 h	Não	Socialmente
Mais que 12 h	Não	Socialmente
De 8 a 10 horas	Não	Nunca
De 8 a 10 horas	Não	Socialmente
De 8 a 10 horas	Não	Socialmente
De 4 a 6 horas	Não	Socialmente
De 8 a 10 horas	Sim	Socialmente
De 10 a 12 horas	Não	Socialmente
De 4 a 6 horas	Não	Socialmente
De 10 a 12 horas	Não	Socialmente

De 8 a 10 horas	Não	Nunca
De 8 a 10 horas	Não	Socialmente
De 10 a 12 horas	Não	Socialmente
De 8 a 10 horas	Não	Socialmente
Menos que 4h	Não	Socialmente
De 8 a 10 horas	Não	Socialmente
De 8 a 10 horas	Não	Nunca
De 8 a 10 horas	Não	Socialmente
De 8 a 10 horas	Não	Socialmente
Mais que 12 h	Não	Socialmente
De 6 a 8 horas	Não	Regularmente
De 6 a 8 horas	Não	Socialmente

De 8 a 10 horas	Não	Nunca
De 10 a 12 horas	Não	Socialmente
De 10 a 12 horas	Não	Nunca
Mais que 12 h	Não	Socialmente
Menos que 4 horas	Não	Socialmente
De 8 a 10 horas	Não	Socialmente
De 8 a 10 horas	Não	Nunca
De 8 a 10 horas	Não	Socialmente
De 10 a 12 horas	Não	Nunca
De 4 a 6 horas	Não	Socialmente
De 6 a 8 horas	Não	Socialmente

Mais que 12 h	Não	Nunca
De 6 a 8 horas	Sim	Socialmente
De 6 a 8 horas	Não	Socialmente
De 8 a 10 horas	Não	Regularmente
De 10 a 12 horas	Não	Nunca
De 10 a 12 horas	Não	Nunca
De 8 a 10 horas	Não	Socialmente
De 4 a 6 horas	Não	Regularmente
De 6 a 8 horas	Não	Socialmente
De 10 a 12 horas	Não	Socialmente
De 10 a 12 horas	Não	Socialmente

De 8 a 10 horas	Não	Socialmente
De 8 a 10 horas	Não	Nunca
Mais que 12h	Não	Socialmente
Mais que 12h	Sim	Socialmente
Mais que 12 h	Não	Socialmente
De 8 a 10 horas	Não	Regularmente
De 8 a 10 horas	Não	Socialmente
De 6 a 8 horas	Não	Socialmente
De 8 a 10 horas	Não	Socialmente
De 6 a 8 horas	Não	Socialmente
De 10 a 12 horas	Não	Socialmente

Mais que 12h	Não	Nunca
De 10 a 12 horas	Não	Socialmente
De 6 a 8 horas	Não	Nunca
De 6 a 8 horas	Não	Socialmente
De 8 a 10 horas	Não	Socialmente
De 6 a 8 horas	Não	Regularmente
De 10 a 12 horas	Não	Socialmente
De 8 a 10 horas	Não	Nunca
De 8 a 10 horas	Não	Socialmente
De 6 a 8 horas	Não	Nunca
De 8 a 10 horas	Não	Regularmente
De 4 a 6 horas	Não	Socialmente

De 8 a 10 horas	Não	Nunca
De 4 a 6 horas	Não	Socialmente
De 8 a 10 horas	Não	Socialmente
De 6 a 8 horas	Não	Nunca
De 6 a 8 horas	Não	Socialmente
De 6 a 8 horas	Sim	Socialmente
De 8 a 10 horas	Não	Nunca
De 6 a 8 horas	Não	Socialmente
De 4 a 6 horas	Não	Socialmente
Menos que 4 horas	Não	Nunca
De 4 a 6 horas	Não	Socialmente

De 8 a 10 horas	Não	Nunca
De 8 a 10 horas	Não	Socialmente
Menos que 4 horas	Não	Nunca
Menos que 4 horas	Não	Socialmente
De 10 a 12 horas	Não	Regularmente
De 8 a 10 horas	Não	Socialmente
De 10 a 12 horas	Não	Socialmente
De 6 a 8 horas	Não	Regularmente
De 4 a 6 horas	Não	Nunca
De 8 a 10 horas	Não	Nunca

De 6 a 8 horas	Não	Nunca
Mais que 12 h	Não	Nunca
De 6 a 8 horas	Não	Regularmente
De 6 a 8 horas	Não	Socialmente
De 6 a 8 horas	Não	Socialmente
De 4 a 6 horas	Não	Socialmente
De 6 a 8 horas	Não	Socialmente
De 8 a 10 horas	Não	Nunca
De 8 a 10 horas	Não	Socialmente
De 4 a 6 horas	Não	Nunca
De 8 a 10 horas	Não	Socialmente

De 6 a 8 horas	Não	Socialmente
Menos que 4 horas	Não	Socialmente
De 8 a 10 horas	Não	Nunca
De 4 a 6 horas	Não	Socialmente
De 8 a 10 horas	Não	Socialmente
De 8 a 10 horas	Não	Nunca
De 10 a 12 horas	Sim	Regularmente
De 6 a 8 horas	Não	Socialmente
De 8 a 10 horas	Não	Nunca
De 4 a 6 horas	Não	Nunca
De 4 a 6 horas	Não	Nunca

De 6 a 8 horas	Não	Nunca
De 8 a 10 horas	Sim	Socialmente
De 6 a 8 horas	Não	Nunca
De 6 a 8 horas	Sim	Socialmente
De 8 a 10 horas	Não	Nunca
Mais que 12h	Não	Regularmente
De 4 a 6 horas	Não	Nunca
Menos que 4 horas	Não	Nunca
Mais que 12 h	Não	Nunca
De 8 a 10 horas	Não	Socialmente

Menos que 4 horas	Não	Socialmente
De 8 a 10 horas	Não	Nunca
Mais que 12 h	Não	Socialmente
De 6 a 8 horas	Não	Socialmente
De 8 a 10 horas	Não	Socialmente
De 8 a 10 horas	Não	Regularmente
De 8 a 10 horas	Não	Socialmente
De 6 a 8 horas	Não	Socialmente
De 10 a 12 horas	Não	Socialmente
De 8 a 10 horas	Não	Socialmente
De 8 a 10 horas	Não	Socialmente
De 8 a 10 horas	Não	Socialmente

De 6 a 8 horas	Não	Socialmente
De 8 a 10 horas	Não	Socialmente
De 8 a 10 horas	Não	Regularmente
De 10 a 12 horas	Não	Nunca
De 8 a 10 horas	Não	Socialmente
De 8 a 10 horas	Não	Socialmente
De 6 a 8 horas	Não	Socialmente
De 4 a 6 horas	Não	Regularmente
De 8 a 10 horas	Não	Socialmente
De 4 a 6 horas	Não	Socialmente
De 6 a 8 horas	Não	Nunca
De 6 a 8 horas	Não	Socialmente

Mais que 12 h	Não	Nunca
De 6 a 8 horas	Não	Nunca
Mais que 12 h	Não	Socialmente
De 4 a 6 horas	Não	Socialmente
De 8 a 10 horas	Sim	Regularmente
De 8 a 10 horas	Não	Socialmente
Menos que 4 horas	Não	Nunca
De 8 a 10 horas	Não	Socialmente
De 10 a 12 horas	Não	Socialmente
Menos que 4 horas	Não	Socialmente
De 8 a 10 horas	Não	Nunca
De 10 a 12 horas	Não	Socialmente

De 6 a 8 horas	Não	Nunca
De 6 a 8 horas	Não	Socialmente
De 6 a 8 horas	Não	Regularmente
De 10 a 12 horas	Não	Socialmente
De 6 a 8 horas	Não	Socialmente
De 8 a 10 horas	Não	Socialmente
De 10 a 12 horas	Não	Nunca
De 8 a 10 horas	Não	Nunca
Menos que 4 horas	Não	Nunca
De 8 a 10 horas	Não	Socialmente
De 6 a 8 horas	Não	Socialmente
Menos que 4 horas	Não	Socialmente

De 8 a 10 horas	Não	Socialmente
Mais que 12 h	Não	Nunca
De 10 a 12 horas	Não	Nunca
De 6 a 8 horas	Não	Socialmente
Menos que 4 horas	Não	Socialmente
De 4 a 6 horas	Não	Socialmente
De 8 a 10 horas	Não	Socialmente
De 6 a 8 horas	Não	Nunca
De 4 a 6 horas	Não	Regularmente
De 6 a 8 horas	Não	Socialmente
De 4 a 6 horas	Não	Nunca
De 4 a 6 horas	Não	Socialmente

De 8 a 10 horas	Não	Nunca
De 8 a 10 horas	Não	Nunca
De 8 a 10 horas	Não	Nunca
De 6 a 8 horas	Sim	Socialmente
De 6 a 8 horas	Não	Socialmente

ísticas do trabalho remoto

Tem algum problema de saúde?	Faz uso de medicamentos?	Se sim, quais?
Sim	Sim	Insulina, vacina para alergia
Não	Não	
Não	Não	
Não	Não	
Não	Não	
Sim	Não	
Sim	Não	
Não	Sim	Tâmisa 20 (anticoncepcional)
Sim	Sim	Espironolactona

Sim	Sim	Quetros (Quetiapina) e Exodus (Escitalopram)
Não	Não	
Não	Não	
Sim	Sim	Cefaliv para enxaqueca
Não	Não	
Sim	Não	
Sim	Não	
Sim	Não	
Não	Sim	Anticoncepcional
Sim	Não	
Não	Não	

Não	Não	
Não	Não	
Não	Não	
Sim	Não	
Sim	Sim	Vitaminas, anticoncepcional, remédios para dor, probiotico, enzima...
Não	Não	
Não	Não	
Não	Sim	Anticoncepcional
Sim	Sim	Histamin
Não	Não	

Sim	Sim	Allurene (dienogeste) e colecalciferol
Sim	Sim	Venlafaxina, Risperidona
Sim	Sim	Antidepressivo
Não	Não	
Não	Não	
Sim	Não	
Não	Sim	Vitaminas
Não	Não	
Sim	Não	
Não	Sim	Finalop
Não	Não	
Não	Não	

Não	Não	
Sim	Não	
Não	Não	
Não	Não	
Sim	Sim	Tandrilax
Não	Sim	Anticoncepcional
Não	Não	
Sim	Não	
Não	Não	
Não	Sim	Anticoncepcional

Sim	Sim	
Sim	Sim	Pamelor/ Anticoncepcional
Sim	Sim	Dexilant
Não	Sim	Mesigyna
Não	Não	
Não	Não	
Sim	Sim	Pristiq, Zolpidem
Não	Não	
Não	Não	
Sim	Sim	Suplementação Vitamina D
Sim	Sim	desvenlafaxina

Sim	Não	
Não	Não	
Não	Não	
Sim	Não	
Não	Não	
Sim	Sim	Vitaminas variadas
Não	Não	
Não	Não	
Não	Não	
Não	Não	
Sim	Não	

Não	Sim	Pondera XR e Repopil
Não	Não	
Não	Sim	Anticoncepcional
Não	Não	
Não	Não	
Não	Não	
Não	Não	
Sim	Não	Não
Não	Não	
Sim	Não	
Não	Não	
Sim	Sim	Brintellix e Rivotril

Não	Não	
Não	Não	
Não	Não	
Sim	Sim	Pimozida
Não	Não	
Não	Não	
Sim	Não	
Sim	Sim	Patz para insonia, depakote para enxaqueca
Sim	Sim	Escitalopram
Sim	Sim	Antidepressivos e remédio pra dormir
Não	Não	
Sim	Não	

Sim	Não	
Não	Não	
Sim	Não	
Não	Não	
Não	Não	
Não	Não	
Sim	Sim	Zoloft
Não	Não	
Não	Sim	Venlift-od
Sim	Não	
Não	Não	

Sim	Não	
Sim	Sim	Escitalopram
Não	Não	
Não	Não	
Não	Não	
Sim	Sim	Zodel, xarrlto, zetron, bactrin, omeprazol, impere
Não	Não	No máximo Dorflex (Relaxante muscular) e Resfenol (Resfriado)
Sim	Não	
Não	Não	
Não	Não	

Não	Não	
Não	Não	
Não	Não	
Não	Não	
Sim	Sim	Escitalopram
Não	Não	
Sim	Sim	Roacutan
Sim	Não	
Não	Não	
Não	Não	
Sim	Não	

Sim	Não	
Sim	Sim	Cloridrato de paroxetina e cloridrato de metilfenidato.
Não	Sim	Anticoncepcional
Não	Não	
Não	Sim	Sintroyde
Não	Não	
Não	Não	
Sim	Não	
Sim	Não	
Sim	Não	
Sim	Sim	

Sim	Sim	Puran T4 - 112mg
Não	Não	
Sim	Sim	Amitriptilina e naramig; Escitalopran
Sim	Não	
Não	Não	
Não	Sim	Preventivo de enxaqueca Depakote
Não	Não	
Não	Não	
Não	Sim	Fluoxetina
Não	Não	
Não	Sim	Puran t4 , complexo vitaminico
Sim	Sim	Topiramato

Não	Não	
Não	Não	
Não	Não	
Não	Não	
Sim	Não	
Sim	Sim	Alenia
Sim	Não	
Não	Não	
Não	Não	
Não	Não	
Não	Sim	Anticoncepcional Repopil 35
Sim	Sim	Naprix

Não	Não	não
Sim	Não	
Não	Não	Nenhum
Não	Sim	Tolrest e zetron x
Não	Sim	Anticoncepcional
Não	Não	
Não	Não	
Não	Não	
Sim	Sim	Tolrest
Não	Não	
Não	Não	

Sim	Sim	Aerolin, minoxidil, finasterida, escitalopram
Não	Não	
Não	Não	
Não	Não	
Não	Sim	Anticocepcional.
Sim	Sim	Patz, alcytam, quetros, torval, dexilant e trimeb
Sim	Sim	Puran
Não	Não	
Não	Não	
Sim	Sim	Fitoterápicos
Sim	Não	

Não	Sim	Anticoncepcional elani ciclo
Não	Não	
Sim	Sim	Aspirina todos os dias
Não	Não	
Não	Não	
Não	Não	
Não	Não	
Não	Não	
Não	Não	
Não	Não	
Sim	Sim	Busonid/reconter 10 mg

Sim	Sim	Quetros, Venvanse
Sim	Não	
Sim	Não	
Sim	Sim	rosucor 10 mg, cartigen 2
Não	Não	
Não	Não	
Sim	Não	
Sim	Sim	Frontal
Sim	Sim	Clortalidona
Sim	Sim	Antidepressivo
Sim	Sim	Rosovastatina
Não	Não	

Não	Não	Não
Não	Não	
Sim	Não	
Não	Não	
Não	Não	
Sim	Sim	Alenia 12/400
Não	Não	
Não	Não	
Não	Não	
Não	Não	
Não	Não	

Sim	Sim	Colchicina, para controle do ácido úrico
Sim	Não	
Não	Não	
Sim	Sim	Sertralina 75mg
Sim	Não	
Sim	Sim	Zoloft 100 mg
Não	Não	
Não	Não	
Não	Não	
Não	Sim	Succinato de desvenlafaxina

Sim	Sim	Eutirox 100mg
Não	Não	
Não	Sim	homeopaticos, florais de bach
Sim	Sim	Sulfato de Hidroxicloroquina - Prednisona
Sim	Sim	Puran
Não	Não	
Sim	Sim	Deferiprona, deferasirox e anticoncepcional.
Sim	Sim	Ritalina, Antidepressivo
Não	Não	
Sim	Sim	
Sim	Não	

Não	Não	
Sim	Sim	Humira
Sim	Sim	Puran
Não	Não	
Sim	Sim	Aradois 25mg
Sim	Não	não
Não	Não	nenhum
Não	Sim	
Não	Não	
Não	Não	
Sim	Sim	Dexilant, domperidona, iumi, topiramato, ômega 3, noripurum, metilfolato, minoxidil, zinco, biotina, metilcobalamina, Vit D, óleo de alho, óleo de linhaça, óleo de prímula, leucogen, trazodona, Zolpidem, piridoxal, glutamina, própolis, desloratadina, prednisona,

Sim	Sim	Pílula anticoncepcional (regularmente), Hyabak (colírio oftálmico), Deocil (pelo menos duas vezes por mês - dor de cabeça e cólica) e Domperidona (às vezes, para auxiliar digestão)
Não	Não	
Não	Não	
Sim	Sim	Buoropiona, Atenolol, Metformina
Não	Não	
Não	Não	
Não	Não	
Não	Não	
Não	Não	
Não	Não	

Não	Não	
Sim	Não	
Sim	Não	
Sim	Não	
Não	Não	
Não	Não	
Sim	Sim	Engov (1 vez a cada 3 dias), Dorflex, Ibuprofeno
Não	Não	
Sim	Sim	Anticoncepcional (Qlaira), Levoid 100 ug
Não	Não	
Sim	Sim	Venlaflaxina, Ritalina
Sim	Sim	Lozartana

Não	Não	
Não	Sim	Zoloft 50mg
Não	Não	
Não	Não	
Não	Sim	Anticoncepcional
Sim	Sim	Puran T4; Glifage 500mg; Brintellix 20mg
Sim	Não	
Não	Não	
Sim	Sim	Pressplus
Sim	Sim	Losartana potássica e besilato de anlodipino
Não	Não	
Sim	Sim	Atacand comb + hct

Sim	Não	
Não	Não	
Não	Não	
Não	Não	
Não	Não	
Sim	Sim	Victoza, barevit, dozemast, vitamina D 50.000 e noripurum
Sim	Não	
Não	Não	
Não	Não	
Não	Não	
Não	Sim	Propranolol
Não	Não	

Sim	Sim	Analgésicos e anti alérgicos
Sim	Sim	CORUS, LIPIDE
Sim	Não	
Sim	Sim	Donaren e apralzolan
Sim	Não	
Não	Não	
Sim	Não	
Sim	Sim	Insulina , maleato de enalapril, zolpidem, euthyrox
Sim	Sim	Pantoprazol, atenolol, naprix A 5+5, Dievari
Sim	Sim	Losartana
Sim	Sim	Wellbutrin e Puran
Não	Não	

Sim	Sim	Puran T4, duloxetina, razapina, Qlaira
Não	Sim	
Sim	Não	
Não	Não	
Não	Não	
Não	Não	
Sim	Sim	Benicar
Sim	Sim	Pressat, sertralina, fluxocor , hidroclorotiazida
Não	Não	
Sim	Sim	Candesartana 16mg HCZ 12.5mg e XGduo 10mg 10000mg
Sim	Sim	Metiformina
Sim	Sim	Coversyl 4 mmg

Sim	Sim	Rabeprazol sódico e Doss
Sim	Sim	Glifag
Não	Não	
Não	Não	
Sim	Sim	Zart 50 mg

Passou a usar algum medicamento durante a pandemia? Se sim, quais?	Qual?	Faz algum outro tratamento para a saúde?
Não		Sim
Não		Não
Não		Sim
Não		Não
Não		Não
Não		Não
Não		Não
Sim	Vitamina D	Sim
Sim	Espironolactona	Sim

Sim	Quetros (Quetiapina) e Exodus (Escitalopram)	Não
Não		Não
Não		Não
Não		Não
Não		Não
Não		Não
Não		Não
Não		Não
Sim	Vitamina D	Não
Não		Não
Não		Não

Não		Não
Não		Não
Não		Não
Sim	Tomei vitaminas d e b12 mas já foi suspendido o uso.	Sim
Não		Não
Não		Não
Não		Não
Não		Não
Não		Não
Não		Não

Sim	colecalciferol	Sim
Não		Não
Sim	Antidepressivo e suplemento vitamínico	Sim
Não		Não
Não		Não
Não		Não
Não		Sim
Não		Não
Não		Sim
Não		Não
Não		Não
Não		Não

Não		Não
Sim	Eventualmente calmantes fitoterápicos	Não
Não		Não
Não		Não
Não		Sim
Não		Não
Sim	Remédio manipulado para dormir	Não
Sim	Melatonina, complexos vitamínicos	Não
Não		Não
Não		Não

Não		Não
Não		Não
Sim	Dexilant	Não
Sim	Óleo de peixe, vitamina de A a Z	Não
Não		Não
Não		Não
Sim	Pristiq, Zolpidem	Não
Não		Não
Não		Não
Não		Não
Sim	desvenlafaxina	Não

Não	Não
Não	Não
Não	Não
Não	Não
Não	Sim
Não	Sim
Não	Não
Não	Não
Não	Não
Não	Não
Não	Não

Sim	Pondera XR	Não
Não		Não
Não		Não
Não		Não
Não		Não
Não		Não
Não		Não
Não		Não
Sim	Vitaminas	Não
Não		Não
Sim	Melatonina	Não
Não		Sim

Não		Não
Não		Não
Não		Não
Não		Não
Sim	Vitamina D	Não
Não		Não
Sim	Vitamina D e uma receita de reposição de vitaminas e minerais.	Sim
Sim	Flanax e Mioflex	Não
Não		Não
Não		Não
Não		Não
Não		Não

Sim	vitaminas	Sim
Não		Não
Sim	Fluoxetina	Não
Não		Não
Não		Não
Não		Não
Não		Não
Não		Não
Não		Sim
Não		Não
Não		Não

Não		Não
Não		Não
Sim	Novalgina para dores de cabeça	Não
Não		Não
Não		Não
Sim	zetron para potencializar efeito com zodel	Sim
Sim	No máximo Dorflex (Relaxante muscular)	Não
Não		Não
Não		Não
Sim	vitamina C, própolis, mel, gengibre	Não

Não		Não
Não		Não
Não		Não
Não		Não
Sim	Cremes para irritação na pele decorrente de stress.	Não
Não		Não
Sim	Roacutan	Não
Não		Não
Não		Não
Não		Não
Não		Não

Não		Não
Não		Não
Não		Não
Não		Não
Não		Sim
Sim	Mais recentemente analgésicos	Não
Não		Sim
Não		Não
Não		Não
Sim	Naproxeno, Codeína e Lugano	Não
Não		Sim

Não		Não
Não		Não
Sim	Aumento de analgésicos	Sim
Não		Não
Sim	Ansiolíticos fitoterápicos, Maracugina	Não
Não		Não
Não		Não
Não		Não
Não		Não
Não		Não
Não		Não
Não		Sim

Não		Não
Não		Não
Não		Não
Não		Não
Não		Não
Sim	Remedio para calvice	Sim
Não		Sim
Não		Não
Não		Não
Não		Não
Não		Não
Não		Sim

Não		Não
Não		Não
Não		Não
Sim	Quetiapina	Não
Não		Não
Não		Não
Não		Não
Não		Não
Não		Não
Não		Não
Não		Sim

Sim	escitalopram	Sim
Não		Não
Não		Não
Sim	Vitamina D	Não
Não		Não
Sim	Dexilant e trimeb	Sim
Não		Não
Não		Não
Não		Não
Não		Sim
Não		Não

Não	Não
Não	Não
Não	Não
Não	Não
Não	Não
Não	Não
Não	Não
Não	Não
Não	Não
Não	Não
Não	Não

Não		Não
Sim	Antialérgicos e torsilax	Não
Não		Não
Não		Não
Não		Não
Não		Não
Não		Não
Sim	Frontal	Não
Sim	Clortalidona	Sim
Não		Não
Não		Não
Não		Não

Não	Não
Não	Sim
Não	Não
Não	Não
Não	Não
Não	Não
Não	Não
Não	Não
Não	Não
Não	Não
Não	Não

Não		Sim
Não		Sim
Não		Não
Sim	Aumentei a dose da sertralina em 25mg	Não
Não		Não
Não		Sim
Não		Não
Não		Sim
Sim	Sertralina	Não
Sim	topiramato	Não

Não		Sim
Não		Sim
Sim	homeopaticos, florais de bach	Não
Não		Não
Não		Não
Não		Sim
Não		Sim
Não		Não
Não		Não
Sim	Rivotril sublingual	Sim
Sim	Desvelafaxina e Ácido Valproico	Sim

Não		Não
Não		Não
Não		Não
Não		Não
Sim	Orlistate e pasalix	Sim
Não		Sim
Não		Não
Não		Não
Não		Não
Não		Não
Sim	Dimpless, altilix, astaxantina, vit k, magnésio dimalato, Vit A, fibregum, nucleotídes, quetiapina	Sim

Não		Sim
Não		Não
Não		Não
Não		Não
Não		Não
Não		Não
Não		Não
Não		Não
Sim	remédios para dores na coluna/analgésicos e contra ansiedade	Não
Não		Sim

Não		Não
Não		Não
Sim	vitamina d	Sim
Não		Sim
Não		Não
Não		Não
Sim	Engov	Não
Não		Sim
Sim	Antidepressivos	Não
Não		Sim
Não		Não
Não		Sim

Sim	antiinflamatorio, analgesicos	Sim
Não		Não
Não		Não
Não		Não
Sim	As vezes para dormir... stilnox de 6,25 mg	Não
Sim	Glifage 500mg; Brintellix 20mg	Sim
Não		Não
Não		Não
Sim	Sinvatastina	Não
Não		Não
Sim	Precisei tomar relaxante muscular injetável	Não
Não		Não

Não	Não
Não	Não
Não	Não
Não	Sim
Não	Não
Não	Sim
Não	Sim
Não	Sim
Não	Não
Não	Não
Não	Sim
Não	Não

Não		Não
Não		Não
Sim	Infralax	Não
Sim	Tolrest	Sim
Não		Sim
Não		Não
Não		Não
Sim	Unoprost	Não
Sim	Dievari	Sim
Não		Não
Sim	Analgésico e relaxante muscular com mais frequência	Sim
Sim	Sertralina	Sim

Não		Sim
Sim	Ansiolítico	Não
Não		Não
Não		Sim
Não		Não
Não		Não
Não		Sim
Não		Não
Não		Não
Não		Não
Não		Não
Não		Não

Sim	Doss	Não
Não		Não
Não		Não
Não		Não
Não		Não

Qual?2	Assinale a opção que você se identifica em relação ao seu trabalho durante a pandemia:	Quantas horas por dia você trabalha durante a pandemia?
Tratamento para alergias	Você está trabalhando exclusivamente (período integral) em home office (em casa) durante a pandemia	de 6 a 10 horas de trabalho
	Você está trabalhando parcialmente (meio período) em home office durante a pandemia	até 6 horas de trabalho
Faço suplementação diária com vitaminas do complexo B	Você está trabalhando exclusivamente (período integral) em home office (em casa) durante a pandemia	de 6 a 10 horas de trabalho
	Você está trabalhando exclusivamente (período integral) em home office (em casa) durante a pandemia	de 6 a 10 horas de trabalho
	Você está trabalhando exclusivamente (período integral) em home office (em casa) durante a pandemia	de 6 a 10 horas de trabalho
	Você está trabalhando parcialmente (meio período) em home office durante a pandemia	até 6 horas de trabalho
	Você está trabalhando exclusivamente (período integral) em home office (em casa) durante a pandemia	mais de 10 horas de trabalho
Terapia	Você está trabalhando exclusivamente (período integral) em home office (em casa) durante a pandemia	de 6 a 10 horas de trabalho
Às vezes, uso salbutamol para asma (bombinha)	Você está trabalhando exclusivamente (período integral) em home office (em casa) durante a pandemia	de 6 a 10 horas de trabalho

	Você está trabalhando exclusivamente (período integral) em home office (em casa) durante a pandemia	de 6 a 10 horas de trabalho
	Você está trabalhando exclusivamente (período integral) em home office (em casa) durante a pandemia	de 6 a 10 horas de trabalho
	Você está trabalhando exclusivamente (período integral) em home office (em casa) durante a pandemia	de 6 a 10 horas de trabalho
	Você está trabalhando exclusivamente (período integral) em home office (em casa) durante a pandemia	de 6 a 10 horas de trabalho
	Você está trabalhando exclusivamente (período integral) em home office (em casa) durante a pandemia	de 6 a 10 horas de trabalho
	Você está trabalhando exclusivamente (período integral) em home office (em casa) durante a pandemia	de 6 a 10 horas de trabalho
	Você está trabalhando exclusivamente (período integral) em home office (em casa) durante a pandemia	de 6 a 10 horas de trabalho
	Você está trabalhando parcialmente (meio período) em home office durante a pandemia	de 6 a 10 horas de trabalho
	Você está trabalhando exclusivamente (período integral) em home office (em casa) durante a pandemia	de 6 a 10 horas de trabalho
	Você está trabalhando exclusivamente (período integral) em home office (em casa) durante a pandemia	de 6 a 10 horas de trabalho
	Você está trabalhando exclusivamente (período integral) em home office (em casa) durante a pandemia	de 6 a 10 horas de trabalho

	Você está trabalhando parcialmente (meio período) em home office durante a pandemia	até 6 horas de trabalho
	Você está trabalhando exclusivamente (período integral) em home office (em casa) durante a pandemia	de 6 a 10 horas de trabalho
	Você está trabalhando parcialmente (meio período) em home office durante a pandemia	até 6 horas de trabalho
Candidíase	Você está trabalhando exclusivamente (período integral) em home office (em casa) durante a pandemia	de 6 a 10 horas de trabalho
	Você está trabalhando exclusivamente (período integral) em home office (em casa) durante a pandemia	mais de 10 horas de trabalho
	Você está trabalhando exclusivamente (período integral) em home office (em casa) durante a pandemia	até 6 horas de trabalho
	Você está trabalhando exclusivamente (período integral) em home office (em casa) durante a pandemia	de 6 a 10 horas de trabalho
	Você está trabalhando exclusivamente (período integral) em home office (em casa) durante a pandemia	de 6 a 10 horas de trabalho
	Você está trabalhando exclusivamente (período integral) em home office (em casa) durante a pandemia	até 6 horas de trabalho
	Você está trabalhando exclusivamente (período integral) em home office (em casa) durante a pandemia	até 6 horas de trabalho

Nutricionista	Você está trabalhando exclusivamente (período integral) em home office (em casa) durante a pandemia	de 6 a 10 horas de trabalho
	Você está trabalhando exclusivamente (período integral) em home office (em casa) durante a pandemia	mais de 10 horas de trabalho
Terapia	Você está trabalhando parcialmente (meio período) em home office durante a pandemia	de 6 a 10 horas de trabalho
	Você está trabalhando exclusivamente (período integral) em home office (em casa) durante a pandemia	de 6 a 10 horas de trabalho
	Você está trabalhando exclusivamente (período integral) em home office (em casa) durante a pandemia	de 6 a 10 horas de trabalho
	Você está trabalhando parcialmente (meio período) em home office durante a pandemia	de 6 a 10 horas de trabalho
Acompanhamento por causa de falta de vitaminas	Você está trabalhando parcialmente (meio período) em home office durante a pandemia	de 6 a 10 horas de trabalho
	Você está trabalhando parcialmente (meio período) em home office durante a pandemia	até 6 horas de trabalho
Psicoterapia	Você está trabalhando exclusivamente (período integral) em home office (em casa) durante a pandemia	de 6 a 10 horas de trabalho
	Você está trabalhando exclusivamente (período integral) em home office (em casa) durante a pandemia	de 6 a 10 horas de trabalho
	Você está trabalhando exclusivamente (período integral) em home office (em casa) durante a pandemia	até 6 horas de trabalho
	Você está trabalhando exclusivamente (período integral) em home office (em casa) durante a pandemia	de 6 a 10 horas de trabalho

	Você está trabalhando parcialmente (meio período) em home office durante a pandemia	de 6 a 10 horas de trabalho
	Você está trabalhando exclusivamente (período integral) em home office (em casa) durante a pandemia	até 6 horas de trabalho
	Você está trabalhando exclusivamente (período integral) em home office (em casa) durante a pandemia	até 6 horas de trabalho
	Você está trabalhando exclusivamente (período integral) em home office (em casa) durante a pandemia	de 6 a 10 horas de trabalho
Terapia	Você está trabalhando exclusivamente (período integral) em home office (em casa) durante a pandemia	de 6 a 10 horas de trabalho
	Você está trabalhando exclusivamente (período integral) em home office (em casa) durante a pandemia	mais de 10 horas de trabalho
	Você está trabalhando exclusivamente (período integral) em home office (em casa) durante a pandemia	até 6 horas de trabalho
	Você está trabalhando parcialmente (meio período) em home office durante a pandemia	de 6 a 10 horas de trabalho
	Você está trabalhando parcialmente (meio período) em home office durante a pandemia	de 6 a 10 horas de trabalho
	Você está trabalhando parcialmente (meio período) em home office durante a pandemia	até 6 horas de trabalho

	Você está trabalhando exclusivamente (período integral) em home office (em casa) durante a pandemia	até 6 horas de trabalho
	Você está trabalhando exclusivamente (período integral) em home office (em casa) durante a pandemia	mais de 10 horas de trabalho
	Você está trabalhando exclusivamente (período integral) em home office (em casa) durante a pandemia	de 6 a 10 horas de trabalho
	Você está trabalhando exclusivamente (período integral) em home office (em casa) durante a pandemia	de 6 a 10 horas de trabalho
	Você está trabalhando parcialmente (meio período) em home office durante a pandemia	de 6 a 10 horas de trabalho
	Você está trabalhando exclusivamente (período integral) em home office (em casa) durante a pandemia	de 6 a 10 horas de trabalho
	Você está trabalhando exclusivamente (período integral) em home office (em casa) durante a pandemia	de 6 a 10 horas de trabalho
	Você está trabalhando exclusivamente (período integral) em home office (em casa) durante a pandemia	de 6 a 10 horas de trabalho
	Você está trabalhando exclusivamente (período integral) em home office (em casa) durante a pandemia	de 6 a 10 horas de trabalho
	Você está trabalhando parcialmente (meio período) em home office durante a pandemia	de 6 a 10 horas de trabalho
	Você está trabalhando exclusivamente (período integral) em home office (em casa) durante a pandemia	de 6 a 10 horas de trabalho

	Você está trabalhando parcialmente (meio período) em home office durante a pandemia	de 6 a 10 horas de trabalho
	Você está trabalhando exclusivamente (período integral) em home office (em casa) durante a pandemia	de 6 a 10 horas de trabalho
	Você está trabalhando parcialmente (meio período) em home office durante a pandemia	até 6 horas de trabalho
	Você está trabalhando exclusivamente (período integral) em home office (em casa) durante a pandemia	de 6 a 10 horas de trabalho
Sessões com psicóloga	Você está trabalhando exclusivamente (período integral) em home office (em casa) durante a pandemia	de 6 a 10 horas de trabalho
Fisioterapia para condromalacia	Você está trabalhando exclusivamente (período integral) em home office (em casa) durante a pandemia	de 6 a 10 horas de trabalho
	Você está trabalhando exclusivamente (período integral) em home office (em casa) durante a pandemia	de 6 a 10 horas de trabalho
	Você está trabalhando exclusivamente (período integral) em home office (em casa) durante a pandemia	de 6 a 10 horas de trabalho
	Você está trabalhando exclusivamente (período integral) em home office (em casa) durante a pandemia	até 6 horas de trabalho
	Você está trabalhando parcialmente (meio período) em home office durante a pandemia	de 6 a 10 horas de trabalho
	Você está trabalhando exclusivamente (período integral) em home office (em casa) durante a pandemia	de 6 a 10 horas de trabalho

	Você está trabalhando exclusivamente (período integral) em home office (em casa) durante a pandemia	de 6 a 10 horas de trabalho
	Você está trabalhando exclusivamente (período integral) em home office (em casa) durante a pandemia	de 6 a 10 horas de trabalho
	Você está trabalhando exclusivamente (período integral) em home office (em casa) durante a pandemia	de 6 a 10 horas de trabalho
	Você está trabalhando exclusivamente (período integral) em home office (em casa) durante a pandemia	até 6 horas de trabalho
	Você está trabalhando exclusivamente (período integral) em home office (em casa) durante a pandemia	até 6 horas de trabalho
	Você está trabalhando parcialmente (meio período) em home office durante a pandemia	de 6 a 10 horas de trabalho
	Você está trabalhando parcialmente (meio período) em home office durante a pandemia	mais de 10 horas de trabalho
	Você está trabalhando exclusivamente (período integral) em home office (em casa) durante a pandemia	de 6 a 10 horas de trabalho
	Você está trabalhando parcialmente (meio período) em home office durante a pandemia	mais de 10 horas de trabalho
	Você está trabalhando exclusivamente (período integral) em home office (em casa) durante a pandemia	de 6 a 10 horas de trabalho
	Você está trabalhando parcialmente (meio período) em home office durante a pandemia	até 6 horas de trabalho
Psicoterapias associadas	Você está trabalhando exclusivamente (período integral) em home office (em casa) durante a pandemia	de 6 a 10 horas de trabalho

	Você está trabalhando exclusivamente (período integral) em home office (em casa) durante a pandemia	de 6 a 10 horas de trabalho
	Você está trabalhando exclusivamente (período integral) em home office (em casa) durante a pandemia	de 6 a 10 horas de trabalho
	Você está trabalhando exclusivamente (período integral) em home office (em casa) durante a pandemia	de 6 a 10 horas de trabalho
	Você está trabalhando exclusivamente (período integral) em home office (em casa) durante a pandemia	de 6 a 10 horas de trabalho
	Você está trabalhando exclusivamente (período integral) em home office (em casa) durante a pandemia	de 6 a 10 horas de trabalho
	Você está trabalhando parcialmente (meio período) em home office durante a pandemia	até 6 horas de trabalho
Fiz dieta e reposição de minerais e vitaminas com Nutrólogo e Cardiologista	Você está trabalhando exclusivamente (período integral) em home office (em casa) durante a pandemia	mais de 10 horas de trabalho
	Você está trabalhando exclusivamente (período integral) em home office (em casa) durante a pandemia	de 6 a 10 horas de trabalho
	Você está trabalhando parcialmente (meio período) em home office durante a pandemia	de 6 a 10 horas de trabalho
	Você está trabalhando exclusivamente (período integral) em home office (em casa) durante a pandemia	de 6 a 10 horas de trabalho
	Você está trabalhando parcialmente (meio período) em home office durante a pandemia	de 6 a 10 horas de trabalho
	Você está trabalhando exclusivamente (período integral) em home office (em casa) durante a pandemia	mais de 10 horas de trabalho

Psicoterapia Analíitico Comportamental - com Psicologo	Você está trabalhando exclusivamente (período integral) em home office (em casa) durante a pandemia	de 6 a 10 horas de trabalho
	Você está trabalhando exclusivamente (período integral) em home office (em casa) durante a pandemia	mais de 10 horas de trabalho
	Você está trabalhando exclusivamente (período integral) em home office (em casa) durante a pandemia	até 6 horas de trabalho
	Você está trabalhando parcialmente (meio período) em home office durante a pandemia	de 6 a 10 horas de trabalho
	Você está trabalhando parcialmente (meio período) em home office durante a pandemia	de 6 a 10 horas de trabalho
	Você está trabalhando exclusivamente (período integral) em home office (em casa) durante a pandemia	mais de 10 horas de trabalho
	Você está trabalhando exclusivamente (período integral) em home office (em casa) durante a pandemia	de 6 a 10 horas de trabalho
	Você está trabalhando exclusivamente (período integral) em home office (em casa) durante a pandemia	de 6 a 10 horas de trabalho
Acompanhamento psicológico e psiquiátrico	Você está trabalhando exclusivamente (período integral) em home office (em casa) durante a pandemia	de 6 a 10 horas de trabalho
	Você está trabalhando parcialmente (meio período) em home office durante a pandemia	de 6 a 10 horas de trabalho
	Você está trabalhando exclusivamente (período integral) em home office (em casa) durante a pandemia	de 6 a 10 horas de trabalho

	Você está trabalhando exclusivamente (período integral) em home office (em casa) durante a pandemia	de 6 a 10 horas de trabalho
	Você está trabalhando parcialmente (meio período) em home office durante a pandemia	até 6 horas de trabalho
	Você está trabalhando exclusivamente (período integral) em home office (em casa) durante a pandemia	de 6 a 10 horas de trabalho
	Você está trabalhando exclusivamente (período integral) em home office (em casa) durante a pandemia	até 6 horas de trabalho
	Você está trabalhando exclusivamente (período integral) em home office (em casa) durante a pandemia	de 6 a 10 horas de trabalho
fisioterapia para reabilitação, acompanhamento em infecção cronica	Você está trabalhando exclusivamente (período integral) em home office (em casa) durante a pandemia	até 6 horas de trabalho
	Você está trabalhando exclusivamente (período integral) em home office (em casa) durante a pandemia	de 6 a 10 horas de trabalho
	Você está trabalhando exclusivamente (período integral) em home office (em casa) durante a pandemia	de 6 a 10 horas de trabalho
	Você está trabalhando exclusivamente (período integral) em home office (em casa) durante a pandemia	até 6 horas de trabalho
	Você está trabalhando exclusivamente (período integral) em home office (em casa) durante a pandemia	de 6 a 10 horas de trabalho

	Você está trabalhando parcialmente (meio período) em home office durante a pandemia	de 6 a 10 horas de trabalho
	Você está trabalhando exclusivamente (período integral) em home office (em casa) durante a pandemia	mais de 10 horas de trabalho
	Você está trabalhando parcialmente (meio período) em home office durante a pandemia	até 6 horas de trabalho
	Você está trabalhando exclusivamente (período integral) em home office (em casa) durante a pandemia	de 6 a 10 horas de trabalho
	Você está trabalhando exclusivamente (período integral) em home office (em casa) durante a pandemia	de 6 a 10 horas de trabalho
	Você está trabalhando parcialmente (meio período) em home office durante a pandemia	mais de 10 horas de trabalho
	Você está trabalhando exclusivamente (período integral) em home office (em casa) durante a pandemia	mais de 10 horas de trabalho
	Você está trabalhando exclusivamente (período integral) em home office (em casa) durante a pandemia	de 6 a 10 horas de trabalho
	Você está trabalhando exclusivamente (período integral) em home office (em casa) durante a pandemia	de 6 a 10 horas de trabalho
	Você está trabalhando exclusivamente (período integral) em home office (em casa) durante a pandemia	até 6 horas de trabalho
	Você está trabalhando exclusivamente (período integral) em home office (em casa) durante a pandemia	até 6 horas de trabalho

	Você está trabalhando exclusivamente (período integral) em home office (em casa) durante a pandemia	mais de 10 horas de trabalho
	Você está trabalhando exclusivamente (período integral) em home office (em casa) durante a pandemia	de 6 a 10 horas de trabalho
	Você está trabalhando exclusivamente (período integral) em home office (em casa) durante a pandemia	de 6 a 10 horas de trabalho
	Você está trabalhando exclusivamente (período integral) em home office (em casa) durante a pandemia	até 6 horas de trabalho
Tireoide	Você está trabalhando parcialmente (meio período) em home office durante a pandemia	mais de 10 horas de trabalho
	Você está trabalhando exclusivamente (período integral) em home office (em casa) durante a pandemia	de 6 a 10 horas de trabalho
Rosácea	Você está trabalhando exclusivamente (período integral) em home office (em casa) durante a pandemia	de 6 a 10 horas de trabalho
	Você está trabalhando exclusivamente (período integral) em home office (em casa) durante a pandemia	de 6 a 10 horas de trabalho
	Você está trabalhando parcialmente (meio período) em home office durante a pandemia	de 6 a 10 horas de trabalho
	Você está trabalhando exclusivamente (período integral) em home office (em casa) durante a pandemia	de 6 a 10 horas de trabalho
Terapia	Você está trabalhando exclusivamente (período integral) em home office (em casa) durante a pandemia	até 6 horas de trabalho

	Você está trabalhando exclusivamente (período integral) em home office (em casa) durante a pandemia	mais de 10 horas de trabalho
	Você está trabalhando parcialmente (meio período) em home office durante a pandemia	mais de 10 horas de trabalho
Tratamento medicamentoso para enxaqueca e não medicamentoso (atividade física e psicoterapia)	Você está trabalhando exclusivamente (período integral) em home office (em casa) durante a pandemia	mais de 10 horas de trabalho
	Você está trabalhando exclusivamente (período integral) em home office (em casa) durante a pandemia	de 6 a 10 horas de trabalho
	Você está trabalhando exclusivamente (período integral) em home office (em casa) durante a pandemia	de 6 a 10 horas de trabalho
	Você está trabalhando parcialmente (meio período) em home office durante a pandemia	mais de 10 horas de trabalho
	Você está trabalhando exclusivamente (período integral) em home office (em casa) durante a pandemia	de 6 a 10 horas de trabalho
	Você está trabalhando parcialmente (meio período) em home office durante a pandemia	até 6 horas de trabalho
	Você está trabalhando parcialmente (meio período) em home office durante a pandemia	de 6 a 10 horas de trabalho
	Você está trabalhando exclusivamente (período integral) em home office (em casa) durante a pandemia	mais de 10 horas de trabalho
	Você está trabalhando parcialmente (meio período) em home office durante a pandemia	de 6 a 10 horas de trabalho
Para enxaqueca	Você está trabalhando exclusivamente (período integral) em home office (em casa) durante a pandemia	de 6 a 10 horas de trabalho

	Você está trabalhando exclusivamente (período integral) em home office (em casa) durante a pandemia	de 6 a 10 horas de trabalho
	Você está trabalhando exclusivamente (período integral) em home office (em casa) durante a pandemia	de 6 a 10 horas de trabalho
	Você está trabalhando parcialmente (meio período) em home office durante a pandemia	mais de 10 horas de trabalho
	Você está trabalhando exclusivamente (período integral) em home office (em casa) durante a pandemia	de 6 a 10 horas de trabalho
	Você está trabalhando exclusivamente (período integral) em home office (em casa) durante a pandemia	até 6 horas de trabalho
Psicólogo	Você está trabalhando exclusivamente (período integral) em home office (em casa) durante a pandemia	de 6 a 10 horas de trabalho
somento o da vitiligo, que passo um creme	Você está trabalhando exclusivamente (período integral) em home office (em casa) durante a pandemia	de 6 a 10 horas de trabalho
	Você está trabalhando parcialmente (meio período) em home office durante a pandemia	de 6 a 10 horas de trabalho
	Você está trabalhando parcialmente (meio período) em home office durante a pandemia	de 6 a 10 horas de trabalho
	Você está trabalhando exclusivamente (período integral) em home office (em casa) durante a pandemia	de 6 a 10 horas de trabalho
	Você está trabalhando exclusivamente (período integral) em home office (em casa) durante a pandemia	de 6 a 10 horas de trabalho
Hipertensão	Você está trabalhando exclusivamente (período integral) em home office (em casa) durante a pandemia	de 6 a 10 horas de trabalho

	Você está trabalhando parcialmente (meio período) em home office durante a pandemia	mais de 10 horas de trabalho
	Você está trabalhando exclusivamente (período integral) em home office (em casa) durante a pandemia	de 6 a 10 horas de trabalho
	Você está trabalhando parcialmente (meio período) em home office durante a pandemia	até 6 horas de trabalho
	Você está trabalhando parcialmente (meio período) em home office durante a pandemia	mais de 10 horas de trabalho
	Você está trabalhando parcialmente (meio período) em home office durante a pandemia	de 6 a 10 horas de trabalho
	Você está trabalhando exclusivamente (período integral) em home office (em casa) durante a pandemia	de 6 a 10 horas de trabalho
	Você está trabalhando exclusivamente (período integral) em home office (em casa) durante a pandemia	de 6 a 10 horas de trabalho
	Você está trabalhando exclusivamente (período integral) em home office (em casa) durante a pandemia	de 6 a 10 horas de trabalho
	Você está trabalhando exclusivamente (período integral) em home office (em casa) durante a pandemia	de 6 a 10 horas de trabalho
	Você está trabalhando exclusivamente (período integral) em home office (em casa) durante a pandemia	de 6 a 10 horas de trabalho
Terapia	Você está trabalhando exclusivamente (período integral) em home office (em casa) durante a pandemia	de 6 a 10 horas de trabalho

Cabelo	Você está trabalhando exclusivamente (período integral) em home office (em casa) durante a pandemia	mais de 10 horas de trabalho
	Você está trabalhando exclusivamente (período integral) em home office (em casa) durante a pandemia	de 6 a 10 horas de trabalho
	Você está trabalhando exclusivamente (período integral) em home office (em casa) durante a pandemia	de 6 a 10 horas de trabalho
	Você está trabalhando exclusivamente (período integral) em home office (em casa) durante a pandemia	de 6 a 10 horas de trabalho
	Você está trabalhando exclusivamente (período integral) em home office (em casa) durante a pandemia	mais de 10 horas de trabalho
Úlcera estomacal	Você está trabalhando exclusivamente (período integral) em home office (em casa) durante a pandemia	até 6 horas de trabalho
	Você está trabalhando exclusivamente (período integral) em home office (em casa) durante a pandemia	até 6 horas de trabalho
	Você está trabalhando exclusivamente (período integral) em home office (em casa) durante a pandemia	de 6 a 10 horas de trabalho
	Você está trabalhando exclusivamente (período integral) em home office (em casa) durante a pandemia	de 6 a 10 horas de trabalho
medicina preventiva	Você está trabalhando exclusivamente (período integral) em home office (em casa) durante a pandemia	mais de 10 horas de trabalho
	Você está trabalhando exclusivamente (período integral) em home office (em casa) durante a pandemia	mais de 10 horas de trabalho

	Você está trabalhando exclusivamente (período integral) em home office (em casa) durante a pandemia	de 6 a 10 horas de trabalho
	Você está trabalhando exclusivamente (período integral) em home office (em casa) durante a pandemia	de 6 a 10 horas de trabalho
	Você está trabalhando exclusivamente (período integral) em home office (em casa) durante a pandemia	mais de 10 horas de trabalho
	Você está trabalhando exclusivamente (período integral) em home office (em casa) durante a pandemia	de 6 a 10 horas de trabalho
	Você está trabalhando exclusivamente (período integral) em home office (em casa) durante a pandemia	de 6 a 10 horas de trabalho
	Você está trabalhando exclusivamente (período integral) em home office (em casa) durante a pandemia	até 6 horas de trabalho
	Você está trabalhando exclusivamente (período integral) em home office (em casa) durante a pandemia	de 6 a 10 horas de trabalho
	Você está trabalhando exclusivamente (período integral) em home office (em casa) durante a pandemia	de 6 a 10 horas de trabalho
	Você está trabalhando exclusivamente (período integral) em home office (em casa) durante a pandemia	mais de 10 horas de trabalho
	Você está trabalhando parcialmente (meio período) em home office durante a pandemia	mais de 10 horas de trabalho
	Você está trabalhando exclusivamente (período integral) em home office (em casa) durante a pandemia	de 6 a 10 horas de trabalho

	Você está trabalhando exclusivamente (período integral) em home office (em casa) durante a pandemia	de 6 a 10 horas de trabalho
	Você está trabalhando exclusivamente (período integral) em home office (em casa) durante a pandemia	de 6 a 10 horas de trabalho
	Você está trabalhando exclusivamente (período integral) em home office (em casa) durante a pandemia	de 6 a 10 horas de trabalho
	Você está trabalhando parcialmente (meio período) em home office durante a pandemia	de 6 a 10 horas de trabalho
	Você está trabalhando exclusivamente (período integral) em home office (em casa) durante a pandemia	de 6 a 10 horas de trabalho
	Você está trabalhando exclusivamente (período integral) em home office (em casa) durante a pandemia	de 6 a 10 horas de trabalho
	Você está trabalhando exclusivamente (período integral) em home office (em casa) durante a pandemia	mais de 10 horas de trabalho
	Você está trabalhando parcialmente (meio período) em home office durante a pandemia	de 6 a 10 horas de trabalho
Drenagem linfática	Você está trabalhando parcialmente (meio período) em home office durante a pandemia	de 6 a 10 horas de trabalho
	Você está trabalhando parcialmente (meio período) em home office durante a pandemia	até 6 horas de trabalho
	Você está trabalhando exclusivamente (período integral) em home office (em casa) durante a pandemia	de 6 a 10 horas de trabalho
	Você está trabalhando parcialmente (meio período) em home office durante a pandemia	de 6 a 10 horas de trabalho

	Você está trabalhando exclusivamente (período integral) em home office (em casa) durante a pandemia	de 6 a 10 horas de trabalho
Aparelho odontológico	Você está trabalhando exclusivamente (período integral) em home office (em casa) durante a pandemia	de 6 a 10 horas de trabalho
	Você está trabalhando exclusivamente (período integral) em home office (em casa) durante a pandemia	de 6 a 10 horas de trabalho
	Você está trabalhando exclusivamente (período integral) em home office (em casa) durante a pandemia	de 6 a 10 horas de trabalho
	Você está trabalhando parcialmente (meio período) em home office durante a pandemia	de 6 a 10 horas de trabalho
	Você está trabalhando exclusivamente (período integral) em home office (em casa) durante a pandemia	de 6 a 10 horas de trabalho
	Você está trabalhando exclusivamente (período integral) em home office (em casa) durante a pandemia	mais de 10 horas de trabalho
	Você está trabalhando exclusivamente (período integral) em home office (em casa) durante a pandemia	de 6 a 10 horas de trabalho
	Você está trabalhando exclusivamente (período integral) em home office (em casa) durante a pandemia	de 6 a 10 horas de trabalho
	Você está trabalhando exclusivamente (período integral) em home office (em casa) durante a pandemia	de 6 a 10 horas de trabalho
	Você está trabalhando exclusivamente (período integral) em home office (em casa) durante a pandemia	até 6 horas de trabalho

Uso de CPAP	Você está trabalhando exclusivamente (período integral) em home office (em casa) durante a pandemia	de 6 a 10 horas de trabalho
Quiropraxia para aliviar as dores nas costas	Você está trabalhando exclusivamente (período integral) em home office (em casa) durante a pandemia	de 6 a 10 horas de trabalho
	Você está trabalhando parcialmente (meio período) em home office durante a pandemia	de 6 a 10 horas de trabalho
	Você está trabalhando parcialmente (meio período) em home office durante a pandemia	até 6 horas de trabalho
	Você está trabalhando exclusivamente (período integral) em home office (em casa) durante a pandemia	mais de 10 horas de trabalho
Ansiedade e depressão e alopecia androgenetica	Você está trabalhando exclusivamente (período integral) em home office (em casa) durante a pandemia	de 6 a 10 horas de trabalho
	Você está trabalhando exclusivamente (período integral) em home office (em casa) durante a pandemia	mais de 10 horas de trabalho
Tive um problema no ombro durante a pandemia e precisei fazer fisioterapia. Atualmente, faço exercícios em casa para o mesmo	Trabalho prioritariamente em casa mas tenho reuniões presenciais	de 6 a 10 horas de trabalho
	Você está trabalhando parcialmente (meio período) em home office durante a pandemia	de 6 a 10 horas de trabalho
	Você está trabalhando exclusivamente (período integral) em home office (em casa) durante a pandemia	de 6 a 10 horas de trabalho

Para Sinrome dos ovário policístico.	Você está trabalhando parcialmente (meio período) em home office durante a pandemia	até 6 horas de trabalho
Estou gestante, tomando vitaminas	Você está trabalhando parcialmente (meio período) em home office durante a pandemia	de 6 a 10 horas de trabalho
	Você está trabalhando exclusivamente (período integral) em home office (em casa) durante a pandemia	de 6 a 10 horas de trabalho
	Você está trabalhando exclusivamente (período integral) em home office (em casa) durante a pandemia	de 6 a 10 horas de trabalho
	Você está trabalhando exclusivamente (período integral) em home office (em casa) durante a pandemia	de 6 a 10 horas de trabalho
Terapia com psicólogo	Você está trabalhando exclusivamente (período integral) em home office (em casa) durante a pandemia	de 6 a 10 horas de trabalho
Transfusões de sangue.	Você está trabalhando exclusivamente (período integral) em home office (em casa) durante a pandemia	até 6 horas de trabalho
	Você está trabalhando exclusivamente (período integral) em home office (em casa) durante a pandemia	de 6 a 10 horas de trabalho
	Você está trabalhando parcialmente (meio período) em home office durante a pandemia	mais de 10 horas de trabalho
Tratamento de sequelas de espinha bífida e acompanhamento psicológico	Você está trabalhando exclusivamente (período integral) em home office (em casa) durante a pandemia	de 6 a 10 horas de trabalho
Psicoterapia - Eclxercícios Físicos	Você está trabalhando exclusivamente (período integral) em home office (em casa) durante a pandemia	de 6 a 10 horas de trabalho

	Você está trabalhando parcialmente (meio período) em home office durante a pandemia	até 6 horas de trabalho
	Você está trabalhando parcialmente (meio período) em home office durante a pandemia	até 6 horas de trabalho
	Você está trabalhando exclusivamente (período integral) em home office (em casa) durante a pandemia	de 6 a 10 horas de trabalho
	Você está trabalhando parcialmente (meio período) em home office durante a pandemia	mais de 10 horas de trabalho
Acompanhamento nutricional	Você está trabalhando exclusivamente (período integral) em home office (em casa) durante a pandemia	até 6 horas de trabalho
tratamento psicoterápico	Você está trabalhando exclusivamente (período integral) em home office (em casa) durante a pandemia	mais de 10 horas de trabalho
	Você está trabalhando parcialmente (meio período) em home office durante a pandemia	mais de 10 horas de trabalho
	Você está trabalhando parcialmente (meio período) em home office durante a pandemia	de 6 a 10 horas de trabalho
	Você está trabalhando exclusivamente (período integral) em home office (em casa) durante a pandemia	mais de 10 horas de trabalho
	Você está trabalhando parcialmente (meio período) em home office durante a pandemia	até 6 horas de trabalho
Tomos vários remédios, todos são tratamentos	Você está trabalhando exclusivamente (período integral) em home office (em casa) durante a pandemia	de 6 a 10 horas de trabalho

limpeza regular dos olhos com shampoo infantil e uso regular de colírio.	Você está trabalhando exclusivamente (período integral) em home office (em casa) durante a pandemia	de 6 a 10 horas de trabalho
	Você está trabalhando exclusivamente (período integral) em home office (em casa) durante a pandemia	de 6 a 10 horas de trabalho
	Você está trabalhando exclusivamente (período integral) em home office (em casa) durante a pandemia	de 6 a 10 horas de trabalho
	Você está trabalhando parcialmente (meio período) em home office durante a pandemia	de 6 a 10 horas de trabalho
	Você está trabalhando exclusivamente (período integral) em home office (em casa) durante a pandemia	mais de 10 horas de trabalho
	Você está trabalhando exclusivamente (período integral) em home office (em casa) durante a pandemia	mais de 10 horas de trabalho
	Você está trabalhando exclusivamente (período integral) em home office (em casa) durante a pandemia	de 6 a 10 horas de trabalho
	Você está trabalhando exclusivamente (período integral) em home office (em casa) durante a pandemia	até 6 horas de trabalho
	Você está trabalhando exclusivamente (período integral) em home office (em casa) durante a pandemia	mais de 10 horas de trabalho
Terapia	Você está trabalhando exclusivamente (período integral) em home office (em casa) durante a pandemia	de 6 a 10 horas de trabalho

	Você está trabalhando parcialmente (meio período) em home office durante a pandemia	de 6 a 10 horas de trabalho
	Você está trabalhando exclusivamente (período integral) em home office (em casa) durante a pandemia	de 6 a 10 horas de trabalho
espinha	Você está trabalhando exclusivamente (período integral) em home office (em casa) durante a pandemia	mais de 10 horas de trabalho
Litíase renal	Você está trabalhando exclusivamente (período integral) em home office (em casa) durante a pandemia	de 6 a 10 horas de trabalho
	Você está trabalhando exclusivamente (período integral) em home office (em casa) durante a pandemia	de 6 a 10 horas de trabalho
	Você está trabalhando exclusivamente (período integral) em home office (em casa) durante a pandemia	de 6 a 10 horas de trabalho
	Você está trabalhando exclusivamente (período integral) em home office (em casa) durante a pandemia	até 6 horas de trabalho
psicoterapia	Você está trabalhando parcialmente (meio período) em home office durante a pandemia	até 6 horas de trabalho
	Você está trabalhando exclusivamente (período integral) em home office (em casa) durante a pandemia	de 6 a 10 horas de trabalho
Terapia para suportar toda essa situação	Você está trabalhando exclusivamente (período integral) em home office (em casa) durante a pandemia	mais de 10 horas de trabalho
	Você está trabalhando exclusivamente (período integral) em home office (em casa) durante a pandemia	de 6 a 10 horas de trabalho
Ácido urico	Você está trabalhando parcialmente (meio período) em home office durante a pandemia	mais de 10 horas de trabalho

fisioterapia	Você está trabalhando exclusivamente (período integral) em home office (em casa) durante a pandemia	até 6 horas de trabalho
	Você está trabalhando exclusivamente (período integral) em home office (em casa) durante a pandemia	de 6 a 10 horas de trabalho
	Você está trabalhando exclusivamente (período integral) em home office (em casa) durante a pandemia	de 6 a 10 horas de trabalho
	Você está trabalhando parcialmente (meio período) em home office durante a pandemia	de 6 a 10 horas de trabalho
	Você está trabalhando parcialmente (meio período) em home office durante a pandemia	de 6 a 10 horas de trabalho
Psicoterapia	Você está trabalhando exclusivamente (período integral) em home office (em casa) durante a pandemia	de 6 a 10 horas de trabalho
	Você está trabalhando parcialmente (meio período) em home office durante a pandemia	de 6 a 10 horas de trabalho
	Você está trabalhando parcialmente (meio período) em home office durante a pandemia	mais de 10 horas de trabalho
	Você está trabalhando exclusivamente (período integral) em home office (em casa) durante a pandemia	mais de 10 horas de trabalho
	Você está trabalhando exclusivamente (período integral) em home office (em casa) durante a pandemia	até 6 horas de trabalho
	Você está trabalhando exclusivamente (período integral) em home office (em casa) durante a pandemia	até 6 horas de trabalho
	Você está trabalhando exclusivamente (período integral) em home office (em casa) durante a pandemia	de 6 a 10 horas de trabalho

	Você está trabalhando exclusivamente (período integral) em home office (em casa) durante a pandemia	de 6 a 10 horas de trabalho
	Você está trabalhando exclusivamente (período integral) em home office (em casa) durante a pandemia	mais de 10 horas de trabalho
	Você está trabalhando exclusivamente (período integral) em home office (em casa) durante a pandemia	de 6 a 10 horas de trabalho
Sim	Você está trabalhando exclusivamente (período integral) em home office (em casa) durante a pandemia	de 6 a 10 horas de trabalho
	Você está trabalhando exclusivamente (período integral) em home office (em casa) durante a pandemia	de 6 a 10 horas de trabalho
aplicação mensal de noripurum	Você está trabalhando exclusivamente (período integral) em home office (em casa) durante a pandemia	de 6 a 10 horas de trabalho
Fisioterapia	Você está trabalhando parcialmente (meio período) em home office durante a pandemia	de 6 a 10 horas de trabalho
Fisioterapia	Você está trabalhando exclusivamente (período integral) em home office (em casa) durante a pandemia	de 6 a 10 horas de trabalho
	Você está trabalhando exclusivamente (período integral) em home office (em casa) durante a pandemia	mais de 10 horas de trabalho
	Você está trabalhando parcialmente (meio período) em home office durante a pandemia	mais de 10 horas de trabalho
Para gastrite e combate à bactéria H. Pilori.	Você está trabalhando exclusivamente (período integral) em home office (em casa) durante a pandemia	de 6 a 10 horas de trabalho
	Você está trabalhando exclusivamente (período integral) em home office (em casa) durante a pandemia	mais de 10 horas de trabalho

	Você está trabalhando exclusivamente (período integral) em home office (em casa) durante a pandemia	de 6 a 10 horas de trabalho
	Você está trabalhando parcialmente (meio período) em home office durante a pandemia	de 6 a 10 horas de trabalho
	Você está trabalhando exclusivamente (período integral) em home office (em casa) durante a pandemia	de 6 a 10 horas de trabalho
Terapia	Você está trabalhando exclusivamente (período integral) em home office (em casa) durante a pandemia	de 6 a 10 horas de trabalho
Começarei fisio terapia para o joelho	Você está trabalhando exclusivamente (período integral) em home office (em casa) durante a pandemia	de 6 a 10 horas de trabalho
	Você está trabalhando exclusivamente (período integral) em home office (em casa) durante a pandemia	mais de 10 horas de trabalho
	Você está trabalhando exclusivamente (período integral) em home office (em casa) durante a pandemia	de 6 a 10 horas de trabalho
	Você está trabalhando exclusivamente (período integral) em home office (em casa) durante a pandemia	de 6 a 10 horas de trabalho
Circulação	Você está trabalhando exclusivamente (período integral) em home office (em casa) durante a pandemia	até 6 horas de trabalho
	Você está trabalhando parcialmente (meio período) em home office durante a pandemia	de 6 a 10 horas de trabalho
Para a repor hormônios da tireoide e para distimia .	Você está trabalhando parcialmente (meio período) em home office durante a pandemia	mais de 10 horas de trabalho
Resistência insulina	Você está trabalhando parcialmente (meio período) em home office durante a pandemia	de 6 a 10 horas de trabalho

Para hipotireoidismo e ansiedade	Você está trabalhando exclusivamente (período integral) em home office (em casa) durante a pandemia	de 6 a 10 horas de trabalho
	Você está trabalhando parcialmente (meio período) em home office durante a pandemia	mais de 10 horas de trabalho
	Você está trabalhando exclusivamente (período integral) em home office (em casa) durante a pandemia	de 6 a 10 horas de trabalho
Acomoanho nível ferritina e viramina D.	Você está trabalhando exclusivamente (período integral) em home office (em casa) durante a pandemia	de 6 a 10 horas de trabalho
	Você está trabalhando exclusivamente (período integral) em home office (em casa) durante a pandemia	até 6 horas de trabalho
	Você está trabalhando parcialmente (meio período) em home office durante a pandemia	mais de 10 horas de trabalho
Hipertensão	Você está trabalhando exclusivamente (período integral) em home office (em casa) durante a pandemia	de 6 a 10 horas de trabalho
	Você está trabalhando exclusivamente (período integral) em home office (em casa) durante a pandemia	até 6 horas de trabalho
	Você está trabalhando exclusivamente (período integral) em home office (em casa) durante a pandemia	de 6 a 10 horas de trabalho
	Você está trabalhando exclusivamente (período integral) em home office (em casa) durante a pandemia	de 6 a 10 horas de trabalho
	Você está trabalhando exclusivamente (período integral) em home office (em casa) durante a pandemia	de 6 a 10 horas de trabalho
	Você está trabalhando parcialmente (meio período) em home office durante a pandemia	de 6 a 10 horas de trabalho

	Você está trabalhando exclusivamente (período integral) em home office (em casa) durante a pandemia	mais de 10 horas de trabalho
	Você está trabalhando exclusivamente (período integral) em home office (em casa) durante a pandemia	de 6 a 10 horas de trabalho
	Você está trabalhando exclusivamente (período integral) em home office (em casa) durante a pandemia	mais de 10 horas de trabalho
	Você está trabalhando parcialmente (meio período) em home office durante a pandemia	de 6 a 10 horas de trabalho
	Você está trabalhando exclusivamente (período integral) em home office (em casa) durante a pandemia	de 6 a 10 horas de trabalho

		Caracterização geral da		
**Você está:**	**da pandemia? (0 corresp**	**Você sentia dor há pelo menos 06 meses antes da pandemia (março de 2020)?**	**Se sim, há quanto tempo você sente dores?**	**ntesdapand**
Saindo de casa somente quando necessário	9	Não		
Saindo de casa somente quando necessário	8	Sim	1 - 5 anos	3
Saindo de casa somente quando necessário	8	Sim	1 - 5 anos	4
Saindo de casa somente quando necessário	7	Sim	Menos de 1 ano	5
Saindo de casa somente quando necessário	7	Não		
Saindo de casa somente quando necessário	10	Não		
De quarentena	3	Não		
Saindo de casa somente quando necessário	7	Não		
Saindo de casa somente quando necessário	10	Não		

Saindo de casa somente quando necessário	4	Não		
Saindo de casa somente quando necessário	9	Não		
Saindo de casa normalmente	7	Não		
Saindo de casa normalmente	5	Sim	1 - 5 anos	6
Saindo de casa somente quando necessário	3	Não		
Saindo de casa somente quando necessário	8	Não		
Saindo de casa somente quando necessário	10	Não		
Saindo de casa somente quando necessário	10	Não		
Saindo de casa somente quando necessário	9	Sim	6 - 10 anos	7
Saindo de casa somente quando necessário	8	Sim	Menos de 1 ano	8
Saindo de casa somente quando necessário	7	Não		

Saindo de casa somente quando necessário	5	Não		
Saindo de casa somente quando necessário	7	Não		
Saindo de casa normalmente	8	Não		
Saindo de casa somente quando necessário	10	Não		
De quarentena	10	Não		
Saindo de casa normalmente	6	Não		
Saindo de casa somente quando necessário	10	Sim	1 - 5 anos	5
Saindo de casa normalmente	10	Não		
Saindo de casa somente quando necessário	8	Não		
Saindo de casa somente quando necessário	9	Não		

Saindo de casa somente quando necessário	8	Sim	1 - 5 anos	5
Saindo de casa somente quando necessário	7	Não		
Saindo de casa somente quando necessário	10	Não		
Saindo de casa somente quando necessário	8	Não		
Saindo de casa somente quando necessário	4	Não		
Saindo de casa somente quando necessário	8	Não		
Saindo de casa somente quando necessário	10	Não		
Saindo de casa somente quando necessário	5	Não		
De quarentena	10	Não		
Saindo de casa somente quando necessário	7	Sim	1 - 5 anos	3
Saindo de casa somente quando necessário	0	Não		
Saindo de casa somente quando necessário	7	Não		

Saindo de casa normalmente	10	Não		
Saindo de casa somente quando necessário	6	Não		
Saindo de casa somente quando necessário	1	Sim	Há mais de 10 anos	1
Saindo de casa normalmente	7	Sim	1 - 5 anos	3
Saindo de casa somente quando necessário	7	Sim	1 - 5 anos	4
Saindo de casa somente quando necessário	5	Sim	1 - 5 anos	5
Saindo de casa somente quando necessário	8	Não		
Saindo de casa somente quando necessário	5	Sim	Não sabe dizer	2
Saindo de casa somente quando necessário	9	Não		
Saindo de casa somente quando necessário	9	Não		

Saindo de casa somente quando necessário	9	Sim	Não sabe dizer	5
Saindo de casa somente quando necessário	9	Não		
Saindo de casa somente quando necessário	8	Não		
Saindo de casa somente quando necessário	10	Não		
Saindo de casa somente quando necessário	10	Não		
Saindo de casa somente quando necessário	10	Não		
Saindo de casa somente quando necessário	8	Não		
Saindo de casa somente quando necessário	7	Não		
Saindo de casa somente quando necessário	7	Não		
Saindo de casa somente quando necessário	8	Sim	6 - 10 anos	6
De quarentena	2	Sim	1 - 5 anos	1

Saindo de casa somente quando necessário	7	Não		
Saindo de casa somente quando necessário	5	Sim	1 - 5 anos	6
Saindo de casa somente quando necessário	10	Não		
Saindo de casa somente quando necessário	9	Não		
Saindo de casa somente quando necessário	8	Não		
Saindo de casa somente quando necessário	10	Não		
Saindo de casa somente quando necessário	10	Não		
Saindo de casa normalmente	7	Não		
Saindo de casa somente quando necessário	8	Não		
Saindo de casa somente quando necessário	1	Não		
Saindo de casa normalmente	7	Não		

Saindo de casa somente quando necessário	8	Não		
Saindo de casa normalmente	10	Não		
Saindo de casa somente quando necessário	10	Sim	Menos de 1 ano	5
Saindo de casa somente quando necessário	7	Não		
Saindo de casa somente quando necessário	8	Sim	1 - 5 anos	2
Saindo de casa somente quando necessário	10	Não		
Saindo de casa somente quando necessário	3	Não		
Saindo de casa somente quando necessário	3	Não		
Saindo de casa somente quando necessário	10	Não		
Saindo de casa somente quando necessário	9	Não		
Saindo de casa somente quando necessário	10	Não		
Saindo de casa somente quando necessário	7	Não		

Saindo de casa somente quando necessário	0	Não		
Saindo de casa somente quando necessário	5	Não		
Saindo de casa somente quando necessário	9	Não		
Saindo de casa normalmente	6	Não		
Saindo de casa normalmente	10	Não		
Saindo de casa somente quando necessário	9	Não		
Saindo de casa somente quando necessário	6	Não		
De quarentena	10	Sim	6 - 10 anos	5
Saindo de casa somente quando necessário	7	Não		
Saindo de casa somente quando necessário	8	Sim	1 - 5 anos	7
Saindo de casa somente quando necessário	7	Não		
Saindo de casa somente quando necessário	5	Sim	Há mais de 10 anos	4

Saindo de casa somente quando necessário	10	Não		
De quarentena	0	Sim	Há mais de 10 anos	4
Saindo de casa somente quando necessário	10	Sim	1 - 5 anos	4
Saindo de casa somente quando necessário	2	Não		
Saindo de casa somente quando necessário	3	Sim	1 - 5 anos	3
Saindo de casa somente quando necessário	10	Não		
De quarentena	4	Não		
Saindo de casa somente quando necessário	7	Não		
Saindo de casa somente quando necessário	8	Sim	6 - 10 anos	4
Saindo de casa somente quando necessário	10	Não		
Saindo de casa somente quando necessário	3	Não		

Saindo de casa somente quando necessário	2	Não		
Saindo de casa normalmente	3	Não		
Saindo de casa somente quando necessário	7	Não		
Saindo de casa normalmente	1	Não		
Saindo de casa somente quando necessário	10	Não		
Saindo de casa somente quando necessário	10	Não		
Saindo de casa somente quando necessário	8	Sim	Há mais de 10 anos	7
Saindo de casa somente quando necessário	9	Sim	Não sabe dizer	5
Saindo de casa somente quando necessário	4	Sim	1 - 5 anos	2
Saindo de casa somente quando necessário	7	Não		

Saindo de casa somente quando necessário	10	Não		
Saindo de casa somente quando necessário	9	Não		
Saindo de casa somente quando necessário	10	Não		
Saindo de casa somente quando necessário	7	Não		
Saindo de casa somente quando necessário	9	Não		
Saindo de casa normalmente	9	Não		
Saindo de casa somente quando necessário	10	Não		
Saindo de casa somente quando necessário	7	Não		
Saindo de casa somente quando necessário	8	Não		
Saindo de casa somente quando necessário	8	Sim	1 - 5 anos	6
Saindo de casa somente quando necessário	3	Não		

Saindo de casa somente quando necessário	10	Não		
Saindo de casa somente quando necessário	7	Não		
Saindo de casa somente quando necessário	10	Não		
Saindo de casa somente quando necessário	8	Não		
Saindo de casa somente quando necessário	8	Não		
Saindo de casa somente quando necessário	8	Não		
Saindo de casa somente quando necessário	5	Não		
Saindo de casa somente quando necessário	4	Sim	1 - 5 anos	4
Saindo de casa normalmente	2	Sim	Há mais de 10 anos	8
Saindo de casa somente quando necessário	7	Não		
Saindo de casa somente quando necessário	4	Não		

Saindo de casa somente quando necessário	10	Não		
Saindo de casa somente quando necessário	3	Não		
Saindo de casa somente quando necessário	9	Sim	Há mais de 10 anos	5
Saindo de casa somente quando necessário	5	Sim	1 - 5 anos	5
Saindo de casa somente quando necessário	7	Sim	1 - 5 anos	2
De quarentena	8	Não		
Saindo de casa somente quando necessário	7	Não		
Saindo de casa somente quando necessário	0	Não		
Saindo de casa normalmente	6	Não		
Saindo de casa somente quando necessário	3	Não		
Saindo de casa normalmente	7	Não		
Saindo de casa somente quando necessário	8	Não		

Saindo de casa somente quando necessário	4	Não			
Saindo de casa somente quando necessário	10	Não			
Saindo de casa somente quando necessário	1	Não			
Saindo de casa normalmente	4	Não			
Saindo de casa normalmente	3	Não			
Saindo de casa somente quando necessário	8	Sim	1 - 5 anos	9
Saindo de casa somente quando necessário	8	Não			
Saindo de casa somente quando necessário	7	Não			
Saindo de casa somente quando necessário	3	Não			
Saindo de casa somente quando necessário	7	Sim	1 - 5 anos	5
De quarentena	7	Sim	1 - 5 anos	4
Saindo de casa somente quando necessário	10	Sim	1 - 5 anos	7

Saindo de casa somente quando necessário	10	Não		
Saindo de casa somente quando necessário	7	Sim	6 - 10 anos	6
Saindo de casa somente quando necessário	7	Não		
Saindo de casa somente quando necessário	9	Não		
Saindo de casa somente quando necessário	7	Não		
Saindo de casa normalmente	8	Não		
Saindo de casa somente quando necessário	3	Não		
Saindo de casa normalmente	3	Não		
Saindo de casa somente quando necessário	9	Sim	1 - 5 anos	5
Saindo de casa somente quando necessário	5	Não		
Saindo de casa normalmente	10	Sim	6 - 10 anos	6

Saindo de casa somente quando necessário	10	Não		
Saindo de casa somente quando necessário	3	Sim	1 - 5 anos	2
Saindo de casa somente quando necessário	6	Não		
Saindo de casa somente quando necessário	9	Não		
Saindo de casa somente quando necessário	10	Não		
Saindo de casa somente quando necessário	10	Não		
Saindo de casa somente quando necessário	6	Não		
Saindo de casa somente quando necessário	7	Não		
Saindo de casa somente quando necessário	8	Sim	1 - 5 anos	5
Saindo de casa somente quando necessário	5	Não		
Saindo de casa somente quando necessário	3	Não		

Saindo de casa somente quando necessário	10	Não		
Saindo de casa somente quando necessário	10	Não		
Saindo de casa somente quando necessário	8	Sim	6 - 10 anos	8
Saindo de casa somente quando necessário	2	Não		
Saindo de casa somente quando necessário	7	Não		
Saindo de casa somente quando necessário	5	Sim	1 - 5 anos	5
Saindo de casa somente quando necessário	5	Não		
Saindo de casa somente quando necessário	8	Não		
Saindo de casa somente quando necessário	9	Não		
Saindo de casa normalmente	5	Sim	1 - 5 anos	3
De quarentena	10	Não		

Saindo de casa somente quando necessário	2	Não		
Saindo de casa somente quando necessário	7	Sim	6 - 10 anos	4
Saindo de casa somente quando necessário	5	Sim	Há mais de 10 anos	4
Saindo de casa somente quando necessário	2	Sim	1 - 5 anos	2
Saindo de casa somente quando necessário	0	Não		
Saindo de casa normalmente	9	Não		
Saindo de casa somente quando necessário	6	Não		
Saindo de casa somente quando necessário	7	Não		
Saindo de casa somente quando necessário	10	Não		
Saindo de casa somente quando necessário	10	Sim	Não sabe dizer	4
Saindo de casa somente quando necessário	10	Não		
Saindo de casa somente quando necessário	9	Não		

Saindo de casa somente quando necessário	5	Não
Saindo de casa somente quando necessário	7	Não
Saindo de casa somente quando necessário	6	Não
Saindo de casa somente quando necessário	9	Não
Saindo de casa somente quando necessário	8	Não
Saindo de casa somente quando necessário	7	Não
Saindo de casa normalmente	10	Não
Saindo de casa normalmente	8	Não
Saindo de casa somente quando necessário	10	Não
Saindo de casa somente quando necessário	8	Não
Saindo de casa somente quando necessário	8	Não

Saindo de casa somente quando necessário	8	Não		
Saindo de casa somente quando necessário	8	Não		
Saindo de casa somente quando necessário	10	Não		
Saindo de casa somente quando necessário	7	Sim	1 - 5 anos	2
Saindo de casa somente quando necessário	9	Não		
Saindo de casa somente quando necessário	7	Não		
Saindo de casa somente quando necessário	6	Sim	Há mais de 10 anos	4
Saindo de casa somente quando necessário	8	Não		
Saindo de casa somente quando necessário	10	Sim	1 - 5 anos	4
Saindo de casa somente quando necessário	10	Sim	1 - 5 anos	6

Saindo de casa somente quando necessário	8	Não		
Saindo de casa somente quando necessário	8	Não		
Saindo de casa somente quando necessário	10	Sim	1 - 5 anos	6
Saindo de casa somente quando necessário	10	Sim	1 - 5 anos	5
Saindo de casa somente quando necessário	8	Não		
Saindo de casa somente quando necessário	7	Não		
Saindo de casa somente quando necessário	8	Não		
Saindo de casa somente quando necessário	8	Não		
Saindo de casa normalmente	2	Não		
Saindo de casa somente quando necessário	0	Sim	Há mais de 10 anos	8
Saindo de casa somente quando necessário	8	Não		

Saindo de casa normalmente	10	Não		
Saindo de casa somente quando necessário	7	Não		
Saindo de casa somente quando necessário	9	Sim	1 - 5 anos	5
Saindo de casa somente quando necessário	8	Não		
De quarentena	10	Sim	6 - 10 anos	4
Saindo de casa somente quando necessário	10	Não		
De quarentena	10	Não		
Saindo de casa somente quando necessário	10	Não		
Saindo de casa somente quando necessário	7	Não		
Saindo de casa somente quando necessário	10	Não		
Saindo de casa somente quando necessário	4	Não		

Saindo de casa somente quando necessário	8	Sim	1 - 5 anos	6
De quarentena	10	Não		
Saindo de casa normalmente	8	Não		
Saindo de casa somente quando necessário	10	Não		
Saindo de casa somente quando necessário	7	Não		
Saindo de casa somente quando necessário	6	Não		
Saindo de casa somente quando necessário	8	Não		
Saindo de casa somente quando necessário	10	Não		
Saindo de casa somente quando necessário	10	Não		
De quarentena	7	Sim	1 - 5 anos	5

Saindo de casa somente quando necessário	9	Não		
Saindo de casa somente quando necessário	5	Não		
Saindo de casa somente quando necessário	7	Não		
Saindo de casa somente quando necessário	5	Não		
Saindo de casa somente quando necessário	10	Não		
Saindo de casa somente quando necessário	3	Não		
Saindo de casa somente quando necessário	8	Sim	Há mais de 10 anos	3
Saindo de casa somente quando necessário	8	Sim	6 - 10 anos	3
Saindo de casa somente quando necessário	8	Não		
Saindo de casa somente quando necessário	10	Não		
Saindo de casa normalmente	5	Não		
Saindo de casa somente quando necessário	8	Sim	6 - 10 anos	6

Saindo de casa somente quando necessário	10	Não		
Saindo de casa somente quando necessário	10	Não		
Saindo de casa somente quando necessário	10	Não		
Saindo de casa somente quando necessário	7	Não		
Saindo de casa somente quando necessário	8	Não		
Saindo de casa somente quando necessário	10	Não		
Saindo de casa somente quando necessário	4	Sim	1 - 5 anos	3
Saindo de casa somente quando necessário	5	Não		
Saindo de casa somente quando necessário	9	Sim	Não sabe dizer	2
Saindo de casa somente quando necessário	5	Não		
Saindo de casa somente quando necessário	0	Não		
Saindo de casa somente quando necessário	8	Sim	1 - 5 anos	3

Saindo de casa somente quando necessário	10	Não		
Saindo de casa somente quando necessário	7	Não		
Saindo de casa somente quando necessário	1	Não		
Saindo de casa somente quando necessário	5	Não		
Saindo de casa normalmente	0	Não		
Saindo de casa somente quando necessário	7	Não		
Saindo de casa somente quando necessário	8	Não		
Saindo de casa somente quando necessário	10	Não		
De quarentena	7	Não		
Saindo de casa somente quando necessário	10	Não		
Saindo de casa somente quando necessário	4	Sim	6 - 10 anos	8
Saindo de casa somente quando necessário	10	Não		

Saindo de casa somente quando necessário	5	Sim	Não sabe dizer	4
Saindo de casa somente quando necessário	3	Não		
Saindo de casa somente quando necessário	5	Sim	1 - 5 anos	3
Saindo de casa somente quando necessário	5	Sim	Ñão sabe dizer	5
Saindo de casa somente quando necessário	9	Não		
De quarentena	9	Não		
Saindo de casa somente quando necessário	2	Não		
De quarentena	10	Sim	1 - 5 anos	7
Saindo de casa somente quando necessário	9	Sim	Não sabe dizer	3
Saindo de casa somente quando necessário	10	Sim	Há mais de 10 anos	6
Saindo de casa somente quando necessário	9	Não		
Saindo de casa normalmente	10	Não		

Saindo de casa somente quando necessário	7	Sim	Menos de 1 ano	8
Saindo de casa somente quando necessário	9	Não		
Saindo de casa somente quando necessário	0	Não		
Saindo de casa somente quando necessário	9	Não		
Saindo de casa somente quando necessário	7	Não		
Saindo de casa normalmente	7	Sim	Não sabe dizer	4
Saindo de casa somente quando necessário	9	Não		
Saindo de casa somente quando necessário	4	Sim	1 - 5 anos	5
Saindo de casa somente quando necessário	8	Não		
Saindo de casa somente quando necessário	5	Sim	1 - 5 anos	5
Saindo de casa somente quando necessário	2	Não		
Saindo de casa somente quando necessário	10	Não		

Saindo de casa somente quando necessário	7	Sim	Não sabe dizer	3
Saindo de casa somente quando necessário	8	Sim	Há mais de 10 anos	3
Saindo de casa somente quando necessário	10	Não		
Saindo de casa somente quando necessário	8	Sim	1 - 5 anos	6
Saindo de casa somente quando necessário	2	Sim	6 - 10 anos	4

dor	Questionário para aqueles qu	
**Assinale a(s) região(s) do corpo que você sentia dor antes do início da pandemia:**	**Se você já sentia dores antes da pandemia, elas continuaram depois da pandemia?**	**Você passou a sentir dor em outros locais durante a pandemia além dos locais que já sentia? aponte a localização desses novos locais:**
Cervical/ pescoço, Coluna lombar (parte inferior das costas)	Sim	Punhos, Cervical (pescoço)
Cervical/ pescoço, Joelhos	Sim	Não
Cabeça, Ombros, Cervical/ pescoço, Coluna lombar (parte inferior das costas), Pés	Sim	Não

Cabeça, Coluna lombar (parte inferior das costas)	Sim	Mãos
Cervical/ pescoço, Coluna lombar (parte inferior das costas)	Sim	Ombros, Pernas
Punhos, Cervical/ pescoço, Coluna torácica (parte do meio das costas), Coluna lombar (parte inferior das costas)	Sim	Cabeça

Cabeça, Ombros, Cotovelos, Cervical/ pescoço, Coluna torácica (parte do meio das costas), Coluna lombar (parte inferior das costas), Quadril	Sim	Cabeça, Ombros

Cabeça, Cervical/ pescoço, Coluna lombar (parte inferior das costas)	Sim	Punhos
Cervical/ pescoço, Coluna torácica (parte do meio das costas)	Sim	Não

Ombros	Sim	Cervical (pescoço)
Ombros, Joelhos	Sim	Mãos, Coluna torácica (parte do meio das costas)
Ombros, Cervical/ pescoço	Sim	Não
Cabeça, Punhos, Coluna lombar (parte inferior das costas)	Sim	Ombros, Cotovelos
Cabeça, Cervical/ pescoço, Coluna torácica (parte do meio das costas), Coluna lombar (parte inferior das costas)	Sim	Não

Cabeça, Mãos, Cervical/ pescoço, Quadril, Joelhos, Pés	Sim	Não
Cervical/ pescoço, Coluna lombar (parte inferior das costas)	Sim	Mãos, Pés
Cabeça, Ombros, Cervical/ pescoço, Coluna torácica (parte do meio das costas)	Sim	Não

Cabeça, Mãos, Braços, Ombros, Punhos, Coluna torácica (parte do meio das costas), Coluna lombar (parte inferior das costas)	Sim	Não

Coluna lombar (parte inferior das costas)	Sim	Cervical (pescoço), Coluna lombar (parte inferior das costas)
Punhos, Coluna torácica (parte do meio das costas)	Sim	Ombros

Ombros, Cervical/ pescoço, Joelhos, Pés	Sim	Cabeça, Coluna lombar (parte inferior das costas), Quadril
Ombros, Cervical/ pescoço, Coluna lombar (parte inferior das costas)	Sim	Mãos, Antebraços, Cotovelos, Joelhos, Tornozelos, Pés
Punhos, Cervical/ pescoço, Coluna lombar (parte inferior das costas)	Sim	Cabeça, Mãos, Ombros, Coluna torácica (parte do meio das costas), Joelhos

Punhos, Joelhos, Pés, pernas	Sim	Não
Cabeça, Coluna lombar (parte inferior das costas)	Sim	Mãos, Braços, Antebraços, Ombros, Tornozelos
Coluna lombar (parte inferior das costas)	Sim	Não
Cabeça	Sim	Cabeça, Punhos, Pés

Cabeça, Ombros, Cervical/ pescoço, Coluna torácica (parte do meio das costas), Quadril	Sim	Joelhos, Tornozelos, Verilha
Cabeça, Cervical/ pescoço, Coluna lombar (parte inferior das costas)	Sim	Joelhos
Cervical/ pescoço, Coluna torácica (parte do meio das costas), Coluna lombar (parte inferior das costas)	Sim	Não

Cervical/ pescoço, Coluna torácica (parte do meio das costas), Coluna lombar (parte inferior das costas)	Sim	Não

Coluna lombar (parte inferior das costas)	Sim	Não
Cabeça, Mãos, Braços, Antebraços, Ombros, Punhos, Cervical/ pescoço, Coluna lombar (parte inferior das costas), Quadril, Joelhos, Tornozelos, Pés	Sim	Não

Cabeça	Sim	Antebraços, Ombros, Cervical (pescoço), Coluna torácica (parte do meio das costas), Coluna lombar (parte inferior das costas)
Coluna lombar (parte inferior das costas)	Sim	Não
Coluna lombar (parte inferior das costas)	Sim	Não

Coluna lombar (parte inferior das costas)	Sim	Não
Ombros, Punhos, Coluna lombar (parte inferior das costas), Tornozelos, Pés	Sim	Não
Cabeça, Ombros, Cervical/ pescoço, Coluna torácica (parte do meio das costas)	Sim	Não
Cabeça, Mãos	Sim	Não

Antebraços, Ombros, Punhos, Cervical/ pescoço, Coluna torácica (parte do meio das costas), Coluna lombar (parte inferior das costas), Joelhos, Tornozelos, Pés	Sim	Não
Coluna torácica (parte do meio das costas), Epidídimo	Sim	Cabeça
Cabeça, Joelhos	Sim	Mãos, Punhos

Cervical/ pescoço	Sim	Não
Quadril	Sim	Não

Joelhos	Sim	Ombros, Punhos, Cervical (pescoço), Coluna torácica (parte do meio das costas)
Cervical/ pescoço, Coluna lombar (parte inferior das costas), Tornozelos	Sim	Punhos
Coluna torácica (parte do meio das costas), Joelhos	Sim	Não

Coluna lombar (parte inferior das costas)	Sim	Cabeça, Mãos, Cervical (pescoço)
Cabeça	Sim	Cabeça
Coluna lombar (parte inferior das costas)	Sim	Ombros
Ombros, Cervical/ pescoço, Coluna torácica (parte do meio das costas), Coluna lombar (parte inferior das costas)	Sim	Cabeça, Problemas alimentares

Cabeça, Ombros, Cervical/ pescoço	Sim	Coluna torácica (parte do meio das costas), Coluna lombar (parte inferior das costas)
Mãos, Joelhos	Sim	Coluna torácica (parte do meio das costas)
Joelhos	Sim	Punhos, Coluna lombar (parte inferior das costas)
Coluna lombar (parte inferior das costas)	Sim	Ombros, Cervical (pescoço), Coluna torácica (parte do meio das costas)

Cervical/ pescoço, Coluna lombar (parte inferior das costas), Tornozelos, panturrilha esquerda	Sim	Ombros, Cervical (pescoço)
Cabeça, Ombros, Coluna lombar (parte inferior das costas)	Sim	Cabeça, Mãos, Braços, Antebraços, Ombros, Cotovelos, Punhos, Cervical (pescoço), Coluna torácica (parte do meio das costas), Coluna lombar (parte inferior das costas), Quadril, Joelhos, Tornozelos, Pés
Mãos, Cervical/ pescoço, Coluna torácica (parte do meio das costas), Coluna lombar (parte inferior das costas), Joelhos	Sim	Aparelho digestório

Cabeça, Coluna torácica (parte do meio das costas)	Sim	Não
Coluna lombar (parte inferior das costas)	Sim	Cervical (pescoço), Coluna torácica (parte do meio das costas), Coluna lombar (parte inferior das costas), Tornozelos

Coluna lombar (parte inferior das costas), pernas	Sim	Cabeça
Mãos, Ombros, Cervical/ pescoço, Coluna lombar (parte inferior das costas), Joelhos, Tornozelos	Sim	Não

Cabeça, Cervical/ pescoço, Coluna lombar (parte inferior das costas)	Sim	Não
Ombros, Cervical/ pescoço	Sim	Cervical (pescoço)
Cabeça, Coluna lombar (parte inferior das costas), Joelhos, Tornozelos	Sim	Quadril

Ombros, Cervical/ pescoço	Sim	Não
Joelhos, Pés	Sim	Não
Calcanhar	Sim	Coluna lombar (parte inferior das costas)

Cabeça	Sim	Ombros, Coluna torácica (parte do meio das costas)

Cabeça, Coluna lombar (parte inferior das costas)	Sim	Ombros, Cotovelos, Coluna lombar (parte inferior das costas)
Coluna torácica (parte do meio das costas), Joelhos	Sim	Coluna lombar (parte inferior das costas), Pés
Cabeça, Cervical/ pescoço, Coluna lombar (parte inferior das costas), Joelhos	Sim	Braços, Ombros, Coluna torácica (parte do meio das costas)
Ombros	Sim	Coluna lombar (parte inferior das costas), Joelhos
Ombros, Coluna lombar (parte inferior das costas), Pés	Sim	Quadril, Joelhos, Pés
Cabeça, Ombros, Cervical/ pescoço	Sim	Não

Mãos, Pernas	Sim	Quadril
Coluna torácica (parte do meio das costas), Coluna lombar (parte inferior das costas), Quadril, Joelhos, Tornozelos, Pés	Sim	Coluna torácica (parte do meio das costas), Coluna lombar (parte inferior das costas), Quadril, Joelhos, Tornozelos, Pés
Cabeça, Braços, Ombros, Coluna lombar (parte inferior das costas), Joelhos	Sim	Ombros
Ombros	Sim	Não

Coluna lombar (parte inferior das costas)	Sim	Mãos, Punhos, Pés
Coluna lombar (parte inferior das costas), Pés	Sim	Quadril
Cervical/ pescoço, Joelhos, Pés	Sim	Antebraços, Ombros, Cervical (pescoço), Joelhos, Pés
Mãos, Braços, Ombros, Cervical/ pescoço	Sim	Não

Avaliação da dor

e sentiam dor antes do início da pandemia									Questionário
**Se passou a sentir dor em outros locais, há quanto tempo você vem sentindo essas novas dores (da localização acima)?**	**Caso você tenha desenvolvido dor durante a pandemia ou piora do quadro já existente, qual fator você considera que seja a causa da dor?**	**do r qu e vo cê ve m se nti nd**	**te m int erf eri do no se u H U**	**do na s su as AT IVI D A DE S**	**do na su a RE LA Ç Ã O C**	**m int erf eri do no se u TR A**	**te m int erf eri do no se u S**		**Se você não apresentava dores frequentes antes da pandemia, você passou a apresentar alguma dor frequente durante a pandemia (março de 2020 até a presente data)?**
									Sim
menos que 6 meses	Estresse, Condições de trabalho em Home office (ambiente, horário e etc)	4	4	3	0	0	0		
Não tive novas dores	Preocupação, Condição financeira, Isolamento social, Sedentarismo (falta de atividade física), Estresse, Condições de trabalho em Home office (ambiente, horário e etc)	7	8	5	3	5	0		
Não tive novas dores	Preocupação, Sedentarismo (falta de atividade física), Estresse	8	6	4	3	6	7		
									Sim
									Sim
									Não
									Não
									Sim

								Sim
								Não
								Sim
menos que 6 meses	Tenho dor nas mãos estilo tendinite, provavelmente devido a ficar muito tempo no computador e celular	6	3	5	5	3	2	
								Sim
								Sim
								Sim
								Não
mais que 6 meses	Sedentarismo (falta de atividade física), Estresse, Condições de trabalho em Home office (ambiente, horário e etc)	8	10	5	8	7	6	
menos que 6 meses	Preocupação, Condição financeira, Sedentarismo (falta de atividade física), Estresse, Condições de trabalho em Home office (ambiente, horário e etc)	8	10	9	6	9	9	
								Não

								Não
								Sim
								Não
								Sim
								Sim
								Não
mais que 6 meses	Sedentarismo (falta de atividade física), Estresse, Condições de trabalho em Home office (ambiente, horário e etc)	8	7	3	4	7	8	
								Não
								Sim
								Sim

menos que 6 meses	Preocupação, Sedentarismo (falta de atividade física), Estresse, Condições de trabalho em Home office (ambiente, horário e etc)	7	9	8	3	5	8	
								Sim
								Não
								Sim
								Não
								Sim
								Sim
								Não
								Sim
Não tive novas dores		2	5	0	0	3	4	
								Não
								Não

								Não
								Sim
menos que 6 meses	Preocupação, Condição financeira, Estresse	9	10	10	3	10	10	
menos que 6 meses	Condições de trabalho em Home office (ambiente, horário e etc), Esporte	4	2	6	2	8	10	
Não tive novas dores	Sedentarismo (falta de atividade física), Estresse, Condições de trabalho em Home office (ambiente, horário e etc)	6	2	6	0	0	9	
mais que 6 meses	Preocupação, Estresse, Condições de trabalho em Home office (ambiente, horário e etc)	6	3	2	1	1	2	
								Não
mais que 6 meses	Sedentarismo (falta de atividade física), Estresse, Condições de trabalho em Home office (ambiente, horário e etc)	7	1	9	4	7	9	
								Sim
								Sim

Não tive novas dores		5	6	6	8	6	7	
								Sim
								Sim
								Sim
								Sim
								Sim
								Sim
								Não
								Não
mais que 6 meses	Sedentarismo (falta de atividade física)	8	8	8	5	7	7	
Não tive novas dores		2	0	0	0	2	0	

								Sim
mais que 6 meses	Condições de trabalho em Home office (ambiente, horário e etc)	5	3	7	0	7	8	
								Não
								Sim
								Sim
								Sim
								Não
								Não
								Sim
								Não
								Sim

								Não
								Não
menos que 6 meses	Sedentarismo (falta de atividade física), Condições de trabalho em Home office (ambiente, horário e etc)	7	6	1	0	0	4	
								Não
menos que 6 meses	Condições de trabalho em Home office (ambiente, horário e etc)	4	6	6	3	0	4	
								Sim
								Sim
								Não
								Sim
								Sim
								Não
								Sim

								Não
								Sim
								Sim
								Não
								Não
								Sim
								Sim
mais que 6 meses	Preocupação, Condição financeira, Isolamento social, Sedentarismo (falta de atividade física), Estresse	9	8	10	6	5	9	
								Não
mais que 6 meses	Sedentarismo (falta de atividade física), Condições de trabalho em Home office (ambiente, horário e etc)	8	10	9	8	8	8	
								Não
menos que 6 meses	Preocupação, Condições de trabalho em Home office (ambiente, horário e etc)	7	0	4	1	6	7	

								Sim
Não tive novas dores	Sedentarismo (falta de atividade física)	2	2	1	1	3	2	
menos que 6 meses	Sedentarismo (falta de atividade física), Estresse	4	7	4	8	9	8	
								Não
Não tive novas dores	Sedentarismo (falta de atividade física), Condições de trabalho em Home office (ambiente, horário e etc)	4	0	3	0	8	0	
								Sim
								Sim
								Sim
mais que 6 meses	Preocupação, Isolamento social, Sedentarismo (falta de atividade física), Estresse	8	9	6	9	4	4	
								Sim
								Não

								Sim
								Não
								Sim
								Não
								Não
								Sim
mais que 6 meses	Preocupação, Isolamento social, Sedentarismo (falta de atividade física), Estresse, Condições de trabalho em Home office (ambiente, horário e etc)	9	8	8	3	7	9	
menos que 6 meses	Preocupação, Estresse, Condições de trabalho em Home office (ambiente, horário e etc)	6	7	5	5	6	4	
Não tive novas dores		2	0	0	0	0	0	
								Não

								Não
								Sim
								Não
								Não
								Não
								Sim
								Sim
								Sim
								Não
Não tive novas dores	Isolamento social, Sedentarismo (falta de atividade física), Condições de trabalho em Home office (ambiente, horário e etc)	6	5	6	4	8	8	
								Não

								Sim
								Sim
								Sim
								Sim
								Sim
								Sim
								Sim
Não tive novas dores	Sedentarismo (falta de atividade física), Condições de trabalho em Home office (ambiente, horário e etc)	6	3	7	3	7	7	
Não tive novas dores	Preocupação, Condição financeira, Sedentarismo (falta de atividade física), Estresse	7	8	9	8	9	8	
								Sim
								Não

								Sim
								Sim
Não tive novas dores	Preocupação, Isolamento social, Estresse, Condições de trabalho em Home office (ambiente, horário e etc)	9	8	6	10	10	10	
Não tive novas dores	Sedentarismo (falta de atividade física), Condições de trabalho em Home office (ambiente, horário e etc)	4	5	5	1	3	7	
Não tive novas dores	Preocupação, Estresse	2	1	0	0	0	1	
								Não
								Sim
								Não
								Não
								Sim
								Sim
								Não

								Não
								Sim
								Não
								Sim
								Não
Não tive novas dores	Sedentarismo (falta de atividade física), Condições de trabalho em Home office (ambiente, horário e etc)	9	10	10	7	5	8	
								Não
								Sim
								Sim
Não tive novas dores	Preocupação, Sedentarismo (falta de atividade física), Estresse	6	6	6	2	4	6	
Não tive novas dores	Preocupação, Sedentarismo (falta de atividade física), Estresse	8	9	8	6	7	9	
Não tive novas dores	Preocupação, Isolamento social	7	3	3	1	2	0	

								Não
Não tive novas dores		6	3	5	0	0	3	
								Não
								Sim
								Não
								Não
								Sim
								Não
menos que 6 meses	Preocupação, Sedentarismo (falta de atividade física), Estresse, Condições de trabalho em Home office (ambiente, horário e etc)	5	6	3	7	4	7	
								Sim
menos que 6 meses	Condições de trabalho em Home office (ambiente, horário e etc)	7	1	2	0	0	0	

								Sim
Não tive novas dores	Sedentarismo (falta de atividade física), Uso frequente do celular	2	1	1	0	3	2	
								Não
								Sim
								Sim
								Sim
								Não
								Não
Não tive novas dores		4	1	3	0	0	0	
								Não
								Sim

								Sim
								Não
mais que 6 meses	Preocupação, Sedentarismo (falta de atividade física), Estresse	9	10	10	10	10	7	
								Sim
								Sim
menos que 6 meses	Sedentarismo (falta de atividade física), Estresse	7	2	5	0	6	7	
								Não
								Não
								Não
Não tive novas dores	Preocupação, Condição financeira, Estresse, Condições de trabalho em Home office (ambiente, horário e etc)	6	7	6	5	6	2	
								Sim

								Não
mais que 6 meses	Sedentarismo (falta de atividade física), Condições de trabalho em Home office (ambiente, horário e etc)	5	5	6	4	7	5	
Não tive novas dores	Sedentarismo (falta de atividade física), Estresse, Condições de trabalho em Home office (ambiente, horário e etc)	8	7	7	9	8	9	
mais que 6 meses	falta de acompanhamento profissional nas ativ fisicas uma vez que os treinos eram online	6	9	7	2	8	1	
								Sim
								Sim
								Sim
								Sim
								Sim
mais que 6 meses	Preocupação, Isolamento social, Estresse, Condições de trabalho em Home office (ambiente, horário e etc)	7	7	8	4	5	9	
								Não
								Sim

	Não
	Não
	Sim
	Sim
	Não
	Não
	Sim
	Não
	Não
	Não
	Sim

								Sim
								Sim
								Sim
mais que 6 meses	Sedentarismo (falta de atividade física), Estresse, Maternidade recente (noites mal dormidas, atividades com bebê o dia todo)	5	8	5	6	5	9	
								Sim
								Não
menos que 6 meses	Estresse	2	2	2	2	2	4	
								Sim
mais que 6 meses	Preocupação, Sedentarismo (falta de atividade física), Condições de trabalho em Home office (ambiente, horário e etc)	6	6	5	5	5	6	
mais que 6 meses	Sedentarismo (falta de atividade física), Estresse, Condições de trabalho em Home office (ambiente, horário e etc)	8	7	6	9	4	7	

									Sim
									Sim
mais que 6 meses	Preocupação, Isolamento social, Estresse, Condições de trabalho em Home office (ambiente, horário e etc)	7	6	6	2	6	5		
mais que 6 meses	Preocupação, Estresse, Condições de trabalho em Home office (ambiente, horário e etc)	10	10	10	10	10	10		
									Sim
									Não
									Sim
									Não
									Não
menos que 6 meses	Preocupação, Condição financeira, Estresse	8	9	9	7	7	7		
									Não

								Sim
								Não
Não tive novas dores	Preocupação, Estresse, Condições de trabalho em Home office (ambiente, horário e etc)	8	6	0	5	10	8	
								Sim
mais que 6 meses	Preocupação, Sedentarismo (falta de atividade física), Estresse, Condições de trabalho em Home office (ambiente, horário e etc)	6	9	7	5	5	8	
								Sim
								Sim
								Sim
								Não
								Não
								Sim

mais que 6 meses	Preocupação, Isolamento social, Estresse	7	5	6	4	7	8	
								Não
								Não
								Não
								Sim
								Não
								Não
								Não
								Sim
Não tive novas dores	Sedentarismo (falta de atividade física)	7	8	5	3	5	7	

								Não
								Sim
								Sim
								Sim
								Sim
								Não
Não tive novas dores	Condições de trabalho em Home office (ambiente, horário e etc)	4	3	3	2	5	3	
Não tive novas dores	Estresse, Condições de trabalho em Home office (ambiente, horário e etc)	7	8	6	7	6	3	
								Sim
								Não
								Sim
mais que 6 meses	Estresse, Condições de trabalho em Home office (ambiente, horário e etc)	9	7	5	4	7	9	

								Sim
								Sim
								Sim
								Não
								Sim
								Sim
Não tive novas dores	Condições de trabalho em Home office (ambiente, horário e etc)	5	2	4	2	2	7	
								Não
Não tive novas dores		2	0	2	0	0	0	
								Não
								Sim
mais que 6 meses	Condições de trabalho em Home office (ambiente, horário e etc)	5	3	5	0	3	1	

								Não
								Não
								Sim
								Não
								Não
								Sim
								Sim
								Não
								Não
								Não
mais que 6 meses	Condições de trabalho em Home office (ambiente, horário e etc)	5	3	3	0	4	0	
								Sim

Não tive novas dores	Condições de trabalho em Home office (ambiente, horário e etc)	4	4	4	4	4	2	
								Sim
mais que 6 meses	Sedentarismo (falta de atividade física), Condições de trabalho em Home office (ambiente, horário e etc)	7	2	6	0	3	5	
mais que 6 meses	Preocupação, Estresse, Condições de trabalho em Home office (ambiente, horário e etc)	8	7	9	6	7	8	
								Sim
								Não
								Sim
mais que 6 meses	Preocupação, Isolamento social, Estresse	7	10	6	7	7	10	
menos que 6 meses	Sedentarismo (falta de atividade física), Estresse	6	7	7	7	7	8	
Não tive novas dores	Preocupação, Sedentarismo (falta de atividade física)	6	7	7	7	7	10	
								Sim
								Sim

menos que 6 meses	Sedentarismo (falta de atividade física), Estresse	7	3	4	4	4	5	
								Sim
								Não
								Sim
								Não
Não tive novas dores	Sedentarismo (falta de atividade física), Estresse, Condições de trabalho em Home office (ambiente, horário e etc)	3	3	3	3	4	2	
								Não
menos que 6 meses	Sedentarismo (falta de atividade física), Condições de trabalho em Home office (ambiente, horário e etc)	9	8	6	7	8	1	
								Não
Não tive novas dores	Condições de trabalho em Home office (ambiente, horário e etc)	5	3	0	0	0	2	
								Não
								Sim

mais que 6 meses	Sedentarismo (falta de atividade física), Condições de trabalho em Home office (ambiente, horário e etc)	5	7	7	3	3	3	
mais que 6 meses	Condições de trabalho em Home office (ambiente, horário e etc)	4	4	0	0	5	0	
								Sim
mais que 6 meses	Condições de trabalho em Home office (ambiente, horário e etc)	6	7	8	6	6	4	
Não tive novas dores		4	4	4	2	3	3	

para aqueles que NÃO sentiam dor antes do início da pandemia									
Se sim, há quanto tempo você vêm sentido essas dores:	Assinale a(s) região(s) do corpo que você sente dor:	Caso você tenha desenvolvido dor durante a pandemia, qual fator você considera que seja a causa da dor?	dor que você vem sentind	tem interferido no seu HU	donas suas ATIVIDADES	dona sua RELAÇÃO C	m interferido no seu TRA	tem interferido no seu S	ce geralmente foi para a ca
Mais de 6 meses	Cabeça, Ombros, Torácica (parte do meio das costas), Lombar (parte inferior das costas)	Estresse	5	8	4	8	5	1	00h
									1h
									Entre 22 e 00h
									00h
Mais de 6 meses	Cabeça, Mãos, Lombar (parte inferior das costas)	Sedentarismo (falta de atividade física)	5	2	3	0	1	0	02:00
Menos de 6 meses	Ombros, Cotovelos, Lombar (parte inferior das costas)	Condições de trabalho em Home office (ambiente, horário e etc)	4	6	3	2	5	6	2 da manhã
									00:20
									23 horas
Mais de 6 meses	Mãos, Antebraços, Ombros, Cotovelos, Punhos, Quadril	Condições de trabalho em Home office (ambiente, horário e etc)	7	8	2	0	6	7	00h00

Menos de 6 meses	Lombar (parte inferior das costas), Joelhos	Sedentarismo (falta de atividade física)	4	3	3	2	1	5	da 01:00 ou 02:00
									2:30am
Menos de 6 meses	Antebraços, Lombar (parte inferior das costas)	Condições de trabalho em Home office (ambiente, horário e etc)	5	7	5	5	7	0	23h30
									1-2 da manhã
Mais de 6 meses	Cervical (pescoço), Lombar (parte inferior das costas), Pés	Sedentarismo (falta de atividade física)	2	1	0	1	6	3	Meia noite
Mais de 6 meses	Cabeça, Mãos, Antebraços, Cervical (pescoço), Lombar (parte inferior das costas), Quadril	Sedentarismo (falta de atividade física)	7	8	7	7	7	8	Meia-noite
Mais de 6 meses	Cabeça, Lombar (parte inferior das costas)	Estresse	6	8	6	7	7	2	22~23
									Meia noite
									23:30
									00:00/01:00
									23:00/22:00

									05
Menos de 6 meses	Torácica (parte do meio das costas), Lombar (parte inferior das costas), Joelhos	Sedentarismo (falta de atividade física)	3	1	1	0	0	0	0h 30
									23 hrs
Mais de 6 meses	Mãos, Antebraços, Ombros, Punhos, Cervical (pescoço), Torácica (parte do meio das costas), Lombar (parte inferior das costas)	Condições de trabalho em Home office (ambiente, horário e etc)	7	8	8	7	8	7	22 h
Mais de 6 meses	Cabeça, Ombros, Cervical (pescoço), Torácica (parte do meio das costas), Lombar (parte inferior das costas), Quadril, Joelhos	Selecionei outro porque acredito que seja o conjunto de todas as anteriores	6	10	7	10	7	10	00 h
									Meia noite.
									23
									1h
Menos de 6 meses	Lombar (parte inferior das costas), Quadril	Condições de trabalho em Home office (ambiente, horário e etc)	6	5	6	2	8	8	00:00
Mais de 6 meses	Cervical (pescoço), Lombar (parte inferior das costas)	Condições de trabalho em Home office (ambiente, horário e etc)	6	4	5	2	3	8	02:00

									Meia noite
Menos de 6 meses	Ombros, Punhos	Sedentarismo (falta de atividade física)	6	4	4	1	4	0	23 h
									[illegible] h meia-noi[illegible]
Mais de 6 meses	Cervical (pescoço), Torácica (parte do meio das costas), Lombar (parte inferior das costas)	Condições de trabalho em Home office (ambiente, horário e etc)	5	5	3	0	4	2	Sim
									22:30
Menos de 6 meses	Lombar (parte inferior das costas)	Sedentarismo (falta de atividade física)	4	1	1	0	2	1	1 am
Mais de 6 meses	Cabeça, Ombros, Cervical (pescoço), Lombar (parte inferior das costas), Joelhos	Preocupação	7	10	8	10	10	10	23 horas
									00 h
Menos de 6 meses	Punhos, Cervical (pescoço), Lombar (parte inferior das costas)	Condições de trabalho em Home office (ambiente, horário e etc)	4	7	5	3	7	7	23h30
									23
									1hr da manhã
									23:30

									23:00
Mais de 6 meses	Ombros, Cervical (pescoço)	Sedentarismo (falta de atividade física)	3	4	4	3	3	5	01h
									23h
									23
									23h30
									01:30
									23
									23
Mais de 6 meses	Lombar (parte inferior das costas)	Preocupação	10	10	10	10	10	10	01:00
Menos de 6 meses	Cervical (pescoço), Lombar (parte inferior das costas), Joelhos, Pés	Estresse	7	8	5	5	6	8	volta das [illegible]

									11h 12h
Menos de 6 meses	Cabeça, Mãos, Ombros, Cervical (pescoço), Lombar (parte inferior das costas)	Estresse	6	8	3	3	3	7	00:00
Menos de 6 meses	Cabeça, Lombar (parte inferior das costas)	Sedentarismo (falta de atividade física)	4	2	1	0	2	3	1h da manhã
Mais de 6 meses	Cabeça, Mãos, Antebraços, Ombros, Cotovelos, Punhos, Cervical (pescoço), Torácica (parte do meio das costas), Lombar (parte inferior das costas), Joelhos, Tornozelos, Pés	Condições de trabalho em Home office (ambiente, horário e etc)	8	7	7	6	9	8	22h
Menos de 6 meses	Quadril, Joelhos, Pés, Glúteo	Estresse	4	6	3	0	6	6	23:30
Menos de 6 meses	Cabeça, Ombros, Punhos, Cervical (pescoço), Torácica (parte do meio das costas), Lombar (parte inferior das costas)	Condições de trabalho em Home office (ambiente, horário e etc)	7	7	8	6	6	8	00:00h
Menos de 6 meses	Torácica (parte do meio das costas)	Condições de trabalho em Home office (ambiente, horário e etc)	2	0	1	0	0	0	4h
									01:00
									22h
									00h
									23h

Menos de 6 meses	Ombros, Cervical (pescoço), Lombar (parte inferior das costas)	Condições de trabalho em Home office (ambiente, horário e etc)	5	8	3	5	2	3	22 hrs
									12 h da noite
									23 hrs
Mais de 6 meses	Cabeça, Lombar (parte inferior das costas), Joelhos, Tornozelos	Sedentarismo (falta de atividade física)	3	3	2	0	3	2	Meia noite
Menos de 6 meses	Lombar (parte inferior das costas)	Estresse	1	1	0	0	0	1	21:30
Menos de 6 meses	Cabeça, Mãos, Ombros, Cotovelos, Lombar (parte inferior das costas)	Condições de trabalho em Home office (ambiente, horário e etc)	4	7	0	0	4	5	21 h
									22 h
									22h30
Mais de 6 meses	Mãos, Antebraços	Sedentarismo (falta de atividade física)	2	5	5	0	5	0	00:00
									[illegible]30 da manh[illegible]
Mais de 6 meses	Cotovelos, Cervical (pescoço), Lombar (parte inferior das costas), Joelhos	Condições de trabalho em Home office (ambiente, horário e etc)	4	6	3	0	0	6	23:00

									23h, meia noite
									23h 00h
									23:00
									3 da manhã
									23h
Menos de 6 meses	Cabeça, Ombros, Punhos, Cervical (pescoço), Torácica (parte do meio das costas), Lombar (parte inferior das costas), Joelhos	Estresse	3	8	3	4	3	7	Meia noite
Menos de 6 meses	Lombar (parte inferior das costas), Pés	Condições de trabalho em Home office (ambiente, horário e etc)	3	2	1	0	2	6	02:00
									00:30
Menos de 6 meses	Cervical (pescoço), Lombar (parte inferior das costas)	Condições de trabalho em Home office (ambiente, horário e etc)	3	0	0	0	0	1	22h
Mais de 6 meses	Cabeça, Ombros, Cervical (pescoço), Joelhos	Sedentarismo (falta de atividade física)	7	7	6	4	7	5	[illegible]pois das 00[illegible]
									23h
Mais de 6 meses	Cabeça, Punhos, Cervical (pescoço), Lombar (parte inferior das costas), Quadril, Joelhos	Estresse	8	10	9	10	8	10	00:00

									ta da s 11 ho ras
Mais de 6 meses	Ombros, Punhos, Lombar (parte inferior das costas)	Estresse	4	5	3	3	5	0	00
Mais de 6 meses	Ombros, Punhos, Lombar (parte inferior das costas), Joelhos	Sedentarismo (falta de atividade física)	3	3	3	0	0	0	00: 00
									23
									01: 30
Menos de 6 meses	Ombros, Lombar (parte inferior das costas)	Condições de trabalho em Home office (ambiente, horário e etc)	4	4	4	4	6	4	23
Menos de 6 meses	Cabeça, Mãos, Ombros, Cervical (pescoço), Torácica (parte do meio das costas), Lombar (parte inferior das costas)	Condições de trabalho em Home office (ambiente, horário e etc)	3	7	3	3	8	7	0h (M eia No ite)
									23: 30
									1h
									1:3 0 A m
									11: 30
									22

Mais de 6 meses	Cabeça, Ombros, Cotovelos, Punhos, Cervical (pescoço), Torácica (parte do meio das costas), Lombar (parte inferior das costas), Quadril, Joelhos	Estresse	4	8	8	8	4	0	24
									00:30
									23
									00:00
									Meia noite
Menos de 6 meses	Cabeça, Punhos	Condições de trabalho em Home office (ambiente, horário e etc)	4	7	6	2	5	5	23:30
Mais de 6 meses	Punhos, Lombar (parte inferior das costas), Tornozelos	Sedentarismo (falta de atividade física)	6	6	4	0	3	5	Todas
Menos de 6 meses	Torácica (parte do meio das costas), Lombar (parte inferior das costas)	Condições de trabalho em Home office (ambiente, horário e etc)	3	2	1	0	3	1	01:00
									treze e meia noi
Mais de 6 meses	Cabeça, Torácica (parte do meio das costas), Lombar (parte inferior das costas)	Sedentarismo (falta de atividade física)	7	3	5	2	5	7	00 h
									00h30

Mais de 6 meses	Cabeça, Ombros, Cervical (pescoço), Torácica (parte do meio das costas)	Estresse	4	8	3	6	4	10	22 h
									02:00
Mais de 6 meses	Cabeça, Ombros, Cervical (pescoço), Lombar (parte inferior das costas), Joelhos, Pés	Sedentarismo (falta de atividade física)	5	8	10	7	9	9	00:00
									1:00
									Apos as 22 h
Menos de 6 meses	Cabeça, Ombros, Punhos, Cervical (pescoço), Torácica (parte do meio das costas)	Condições de trabalho em Home office (ambiente, horário e etc)	5	2	8	1	8	10	10 h
									22 h
									23:00
									Meia noite
									22:00

									22 h
Mais de 6 meses	Cabeça, Ombros	Condições de trabalho em Home office (ambiente, horário e etc)	3	1	1	1	2	1	00:00
									23:00
									1:00
									[illegible] horas da manhã
Menos de 6 meses	Cabeça, Lombar (parte inferior das costas)	Condições de trabalho em Home office (ambiente, horário e etc)	5	7	2	5	2	0	23
Menos de 6 meses	Cabeça, Quadril, Pés	Condições de trabalho em Home office (ambiente, horário e etc)	3	1	1	0	3	2	23:55 00:00
Menos de 6 meses	Torácica (parte do meio das costas)	Mau jeito	2	1	2	0	0	1	23 h
									23 h
									3
									01h00

Mais de 6 meses	Cabeça, Mãos, Antebraços, Punhos, Cervical (pescoço), Torácica (parte do meio das costas), Lombar (parte inferior das costas), Quadril	Condições de trabalho em Home office (ambiente, horário e etc)	8	10	7	9	9	10	22:00
Mais de 6 meses	Cabeça, Mãos, Lombar (parte inferior das costas)	Sedentarismo (falta de atividade física)	4	4	2	6	8	5	00:00
Menos de 6 meses	Abdomen	Sedentarismo (falta de atividade física)	6	7	9	9	6	10	10
Menos de 6 meses	Mãos, Punhos, Torácica (parte do meio das costas), Lombar (parte inferior das costas)	Cadeira ruim	4	4	3	3	7	5	23 h-00 h
Menos de 6 meses	Cabeça, Cervical (pescoço), Lombar (parte inferior das costas)	Estresse	8	7	7	7	8	5	23:00 h
Menos de 6 meses	Cabeça, Torácica (parte do meio das costas), Lombar (parte inferior das costas)	Sedentarismo (falta de atividade física)	4	6	2	2	4	4	22 h
Mais de 6 meses	Ombros, Cervical (pescoço), Lombar (parte inferior das costas)	Condições de trabalho em Home office (ambiente, horário e etc)	3	4	1	0	4	3	22:50
									2
									23 h
Mais de 6 meses	Lombar (parte inferior das costas), Quadril	Sedentarismo (falta de atividade física)	5	7	7	2	4	10	22:30
									00 h

Menos de 6 meses	Punhos, Cervical (pescoço), Lombar (parte inferior das costas)	Sedentarismo (falta de atividade física)	5	2	3	0	3	0	23h
Mais de 6 meses	Cervical (pescoço), Lombar (parte inferior das costas)	Sedentarismo (falta de atividade física)	4	3	0	2	0	3	24:00/1:00
									Meis noite
									[illegible]noite / 1h da ma[illegible]
									Sim, à 00:00
									22:30
Menos de 6 meses	Joelhos, Tornozelos, Pés	posição do corpo	2	2	0	0	1	0	01
									23h
									23h30
Menos de 6 meses	Antebraços, Cervical (pescoço), Torácica (parte do meio das costas), Lombar (parte inferior das costas), Quadril, Joelhos, Pés	Condições de trabalho em Home office (ambiente, horário e etc)	9	5	8	6	5	5	22hs
Menos de 6 meses	Cabeça, Lombar (parte inferior das costas)	Estresse	4	2	1	0	0	2	23:00 - 00:00
									02:00

									23:30
Mais de 6 meses	Cabeça, Cervical (pescoço), Torácica (parte do meio das costas), Lombar (parte inferior das costas), Quadril	Estresse	6	6	5	5	5	6	Meia noite
									23 hrs
Mais de 6 meses	Lombar (parte inferior das costas)	Exercícios mal orientados	2	0	1	0	0	0	1 am
									22
									23
									00:00
Mais de 6 meses	Punhos, Lombar (parte inferior das costas)	Estresse	3	7	6	6	7	9	23h30
Mais de 6 meses	Cabeça, Ombros, Cervical (pescoço)	soma de praticamente todas as alternativas	4	3	3	3	3	7	22:00
									meia noite
									1h30 da manhã
									22

									21 h
									23
									00:00 h
Mais de 6 meses	Cabeça, Ombros, Cervical (pescoço), Lombar (parte inferior das costas)	Condições de trabalho em Home office (ambiente, horário e etc)	5	8	6	5	7	9	Meia noite
									22 h
									23:00
Menos de 6 meses	Cervical (pescoço), Torácica (parte do meio das costas), Lombar (parte inferior das costas), Joelhos	Condições de trabalho em Home office (ambiente, horário e etc)	7	7	7	8	5	8	23 hs
									23:00
									01:00
Menos de 6 meses	Ombros, Cervical (pescoço)	Condições de trabalho em Home office (ambiente, horário e etc)	6	3	3	2	4	2	23
									00:30

Mais de 6 meses	Cabeça, Pescoço	Condições de trabalho em Home office (ambiente, horário e etc)	8	8	8	8	8	10	12 h30min
									02:00
									22 h
Menos de 6 meses	Cabeça, Cervical (pescoço)	Condições de trabalho em Home office (ambiente, horário e etc)	6	5	3	3	5	7	22:30
Mais de 6 meses	Ombros, Torácica - peito.	Tenho várias alternativas acima.	5	1	0	0	1	7	1h.
Mais de 6 meses	Cabeça, Punhos, Lombar (parte inferior das costas), Quadril	Sedentarismo (falta de atividade física)	4	9	7	10	10	9	21
									23 h
									21:30
									23
									23:30
Menos de 6 meses	Cabeça, Ombros, Torácica (parte do meio das costas), Lombar (parte inferior das costas)	Sedentarismo (falta de atividade física)	5	5	6	2	1	5	[illegible] hora da manh[illegible]

Mais de 6 meses	Cabeça, Punhos, Lombar (parte inferior das costas)	Condições de trabalho em Home office (ambiente, horário e etc)	6	7	5	5	6	5	23 hrs
									22 h
									22:30
Mais de 6 meses	Mãos, Lombar (parte inferior das costas), Joelhos, Tornozelos, Pés	Sedentarismo (falta de atividade física)	2	1	1	0	2	0	11:30
Menos de 6 meses	Cervical (pescoço), Torácica (parte do meio das costas), Lombar (parte inferior das costas)	Condições de trabalho em Home office (ambiente, horário e etc)	8	7	3	1	9	8	1h 30
									23:30
									23 horas
									22:30
									00:40
									23:30
Mais de 6 meses	Cabeça, Cervical (pescoço), Herpes labial	Preocupação	5	7	4	6	3	3	1:00

									2
									1h da manhã
									1 da manhã
									23 h
Menos de 6 meses	Lombar (parte inferior das costas)	Condições de trabalho em Home office (ambiente, horário e etc)	3	1	2	0	1	0	23:30
Mais de 6 meses	Cabeça, Antebraços	Preocupação	6	7	7	8	10	8	Meia noite
Mais de 6 meses	Joelhos	Sedentarismo (falta de atividade física)	4	5	6	1	4	4	Meia Noite
Mais de 6 meses	Cabeça, Ombros, Cervical (pescoço), Torácica (parte do meio das costas), Lombar (parte inferior das costas)	Estresse	7	5	5	5	8	7	3
Mais de 6 meses	Cabeça, Lombar (parte inferior das costas), Quadril	Sedentarismo (falta de atividade física)	5	1	7	0	6	3	23 h
									22:00 horas
									23:30
Mais de 6 meses	Cabeça, Cervical (pescoço), Torácica (parte do meio das costas)	Condições de trabalho em Home office (ambiente, horário e etc)	6	6	2	2	7	7	23:00

									23 hrs
									10
Menos de 6 meses	Lombar (parte inferior das costas)	Sedentarismo (falta de atividade física)	3	3	2	0	2	2	23 horas
Mais de 6 meses	Ombros, Cotovelos, Punhos, Cervical (pescoço), Quadril	Condições de trabalho em Home office (ambiente, horário e etc)	6	2	2	1	7	1	00:00
									22:00
									0h
Mais de 6 meses	Cabeça, Ombros, Cervical (pescoço), Torácica (parte do meio das costas), Lombar (parte inferior das costas)	Isolamento social, sedentarismo, ansiedade, depressão,	4	2	8	0	6	10	23 horas
									23 h
									22 h
									22:30
Menos de 6 meses	Joelhos	Sedentarismo (falta de atividade física)	5	5	2	1	0	0	00:00

Menos de 6 meses	Cervical (pescoço), Torácica (parte do meio das costas)	Sedentarismo (falta de atividade física)	4	5	2	4	5	9	Meia noite
Mais de 6 meses	Ombros, Cervical (pescoço), Torácica (parte do meio das costas), Lombar (parte inferior das costas), Joelhos	Condições de trabalho em Home office (ambiente, horário e etc)	5	8	7	8	9	6	Por volta das 23 horas
Menos de 6 meses	Torácica (parte do meio das costas), Lombar (parte inferior das costas), Joelhos	Preocupação	6	9	8	7	7	6	23 h
									23:00
Mais de 6 meses	Cervical (pescoço), Lombar (parte inferior das costas)	Condição financeira	6	7	5	6	5	8	22
									22:00
									01:00
Mais de 6 meses	Cabeça, Ombros	Condições de trabalho em Home office (ambiente, horário e etc)	8	9	2	1	1	7	22 h
									21
									22 hs 60

Menos de 6 meses	Lombar (parte inferior das costas), Pernas	Sedentarismo (falta de atividade física)	5	7	4	0	3	8	23h
Mais de 6 meses	Lombar (parte inferior das costas)	Acredito ser da gestação	7	6	6	4	5	5	22h
									20:30/21:00
									23:00
Mais de 6 meses	Cabeça, Ombros, Punhos, Cervical (pescoço)	Sedentarismo (falta de atividade física)	6	2	0	3	1	3	0:00
									2h da madrugad[illegible]
Menos de 6 meses	Pernas (principalmente esquerda)	Sedentarismo (falta de atividade física)	5	3	4	0	0	3	23h30 a 24h
									1h
									1am
									23 hrs
									22

Mais de 6 meses	Ombros, Lombar (parte inferior das costas)	Condições de trabalho em Home office (ambiente, horário e etc)	2	0	2	0	0	0	2
									22:45
									23
Menos de 6 meses	Punhos, Cervical (pescoço), Lombar (parte inferior das costas)	Condições de trabalho em Home office (ambiente, horário e etc)	6	9	3	0	3	7	22 horas
									23 h
Menos de 6 meses	Mãos, Antebraços	Condições de trabalho em Home office (ambiente, horário e etc)	5	5	5	5	5	5	[illegible]ras da madrug[illegible]
Menos de 6 meses	Mãos, Cervical (pescoço), Torácica (parte do meio das costas), Quadril, Joelhos	Sedentarismo (falta de atividade física)	5	1	1	1	1	2	22 horas
Mais de 6 meses	Cabeça, Ombros	Estresse	7	8	6	7	8	8	Meia noite
									24
									1 da manhã
Mais de 6 meses	Cabeça, Ombros, Cervical (pescoço), Torácica (parte do meio das costas), Lombar (parte inferior das costas), Pés	Estresse	8	9	9	9	9	9	00 h

									23 h
									03:00
									23:30
									22:30
Mais de 6 meses	Ombros, Lombar (parte inferior das costas)	Estresse	9	6	8	2	5	10	23 horas
									Entre 00 e 02
									22
									23 horas
Mais de 6 meses	Cabeça, Mãos, Antebraços, Ombros, Cotovelos, Punhos, Cervical (pescoço), Lombar (parte inferior das costas)	Condições de trabalho em Home office (ambiente, horário e etc)	10	9	10	9	10	10	01 h da madrugada
									0 h

									22:00
Menos de 6 meses	Lombar (parte inferior das costas), Quadril	Condições de trabalho em Home office (ambiente, horário e etc)	2	2	1	1	1	1	23h00
Menos de 6 meses	Cervical (pescoço), Lombar (parte inferior das costas)	Sedentarismo (falta de atividade física)	7	9	9	8	10	10	00h00
Mais de 6 meses	Lombar (parte inferior das costas), Baixo ventre	Condições de trabalho em Home office (ambiente, horário e etc)	5	5	5	8	5	8	Sim
Mais de 6 meses	Cervical (pescoço), Lombar (parte inferior das costas), Quadril	Condições de trabalho em Home office (ambiente, horário e etc)	7	4	7	3	4	8	tre 3 e 5 da manh
									23:00
									4:00
									22:00
Menos de 6 meses	Lombar (parte inferior das costas), Cóccix	Sedentarismo (falta de atividade física)	4	2	2	1	3	3	23h
									, fds mais tarde
Mais de 6 meses	Lombar (parte inferior das costas), Joelhos	Sedentarismo (falta de atividade física)	5	6	3	3	2	5	00h00
									0

Mais de 6 meses	Lombar (parte inferior das costas), Quadril	Sedentarismo (falta de atividade física)	7	10	8	8	7	9	24 h
Menos de 6 meses	Cabeça, Ombros, Lombar (parte inferior das costas)	Condições de trabalho em Home office (ambiente, horário e etc)	5	5	5	5	5	5	24
Mais de 6 meses	Antebraços, Ombros, Cervical (pescoço), Torácica (parte do meio das costas), Lombar (parte inferior das costas)	Condições de trabalho em Home office (ambiente, horário e etc)	4	3	6	5	8	2	23 h
									3:00
Menos de 6 meses	Cabeça, Mãos, Ombros, Punhos, Cervical (pescoço), Torácica (parte do meio das costas), Lombar (parte inferior das costas)	Estresse	6	6	6	5	5	6	22:00
Mais de 6 meses	Lombar (parte inferior das costas)	Estresse	7	8	10	8	9	10	22
									23 horas
									23:00 h
									23:00
									22 horas
Mais de 6 meses	Cervical (pescoço), Pés	Não sei	3	1	4	0	1	1	21:30
									23 h

									0 da madrugad
									0:00
Menos de 6 meses	Mãos, Antebraços, Ombros, Cervical (pescoço)	Condições de trabalho em Home office (ambiente, horário e etc)	5	5	3	0	8	1	0h
									Quase sempre.
									20:00
Mais de 6 meses	Punhos, Lombar (parte inferior das costas), Quadril, Joelhos, Tornozelos, Pés	Sedentarismo (falta de atividade física)	5	7	9	6	7	5	23h
Mais de 6 meses	Cabeça, Ombros, Cervical (pescoço), Lombar (parte inferior das costas), Joelhos	Estresse	6	3	7	8	4	9	Meia noite
									23h00
									1:30
									22h
									23h
Menos de 6 meses	Cabeça, Ombros, Cervical (pescoço), Quadril	Condições de trabalho em Home office (ambiente, horário e etc)	5	8	4	2	7	0	5h

									23 horas
Menos de 6 meses	CARDIACA	Sedentarismo (falta de atividade física)	3	3	5	4	7	3	23H00
									23 h
									Após 1h ou 2 h
Menos de 6 meses	Joelhos	tombo de bicicleta	6	6	8	3	1	5	22:30 h
									23 h
Menos de 6 meses	Cabeça	Preocupação	3	3	3	3	3	0	[illegible]ntinuo inicia[illegible]
									10:00
									0:00h
									23 h
Mais de 6 meses	Cabeça, Ombros, Cervical (pescoço), Torácica (parte do meio das costas)	Condições de trabalho em Home office (ambiente, horário e etc)	6	9	6	5	7	6	23 horas
Menos de 6 meses	Ombros, Punhos	Estresse	8	10	7	6	9	9	23:30

									22
Mais de 6 meses	Ombros	Condições de trabalho em Home office (ambiente, horário e etc)	7	4	6	4	7	1	Meia noite
									22
Menos de 6 meses	Mãos, Cervical (pescoço), Lombar (parte inferior das costas), Quadril, Pernas.	Preocupação	5	1	2	2	3	3	23:30
									reo mesmo ho
									1h
									22
									Meia noite
									23
									Meia noite
									23
Mais de 6 meses	Lombar (parte inferior das costas)	Condições de trabalho em Home office (ambiente, horário e etc)	5	3	6	3	5	0	23 hs

									2 da madrugad[illegible]
									23:50
Mais de 6 meses	Cabeça, Cervical (pescoço)	Preocupação	5	5	5	0	0	0	0h30
									23 horas
									22h00

po (em minutos) você geralment	do você geralmente levan	Este pode ser diferente do	Durante o último mês, com que frequência você teve dificuldade de dormir porque você não conseguiu adormecer em até 30 minutos?	Durante o último mês, com que frequência você teve dificuldade de dormir porque você acordou no meio da noite ou de manhã cedo?	Durante o último mês, com que frequência você teve dificuldade de dormir porque precisou levantar para ir ao banheiro?
30 min	9h	8/9h	1 ou 2 vezes por semana	Nenhuma no último mês	Menos de 1 vez por semana
10min	7h - 8h	4 - 5	Nenhuma no último mês	Nenhuma no último mês	Nenhuma no último mês
De 30 a 50min	Entre 7:30 e 9h	6 a 8h	1 ou 2 vezes por semana	Menos de 1 vez por semana	Nenhuma no último mês
de 2h a 4h	8h	6h	3 ou mais vezes por semana	3 ou mais vezes por semana	3 ou mais vezes por semana
15 minutos	09:00	6 horas	1 ou 2 vezes por semana	1 ou 2 vezes por semana	Nenhuma no último mês
30	10	7/8	3 ou mais vezes por semana	1 ou 2 vezes por semana	Menos de 1 vez por semana
90	06:30	05:30	3 ou mais vezes por semana	Nenhuma no último mês	Nenhuma no último mês
30 minutos	7 - 8h	7 horas	1 ou 2 vezes por semana	Menos de 1 vez por semana	Nenhuma no último mês
40 minutos	8h 00	5h ou 6h	1 ou 2 vezes por semana	Nenhuma no último mês	Nenhuma no último mês

30 minutos	08:30	4 ou 5 horas	3 ou mais vezes por semana	3 ou mais vezes por semana	Nenhuma no último mês
Mais de 50	9:40am	5	3 ou mais vezes por semana	Nenhuma no último mês	1 ou 2 vezes por semana
20	7h00	8h	1 ou 2 vezes por semana	Menos de 1 vez por semana	1 ou 2 vezes por semana
15 minutos	9h da manhã	6-8h por noite	Menos de 1 vez por semana	Menos de 1 vez por semana	1 ou 2 vezes por semana
20 minutos	6 horas	6 horas	Menos de 1 vez por semana	Nenhuma no último mês	Nenhuma no último mês
30	8h00	6 a 7 horas	1 ou 2 vezes por semana	1 ou 2 vezes por semana	3 ou mais vezes por semana
não sei	05~06	6~7	1 ou 2 vezes por semana	3 ou mais vezes por semana	Nenhuma no último mês
20	7h	6h	Menos de 1 vez por semana	Menos de 1 vez por semana	3 ou mais vezes por semana
20	6:30	7	Menos de 1 vez por semana	Menos de 1 vez por semana	Nenhuma no último mês
00:30	08:00	08h00	1 ou 2 vezes por semana	Menos de 1 vez por semana	1 ou 2 vezes por semana
Rápido	6:30am	7 em meia	Nenhuma no último mês	Nenhuma no último mês	Nenhuma no último mês

1	11	6	1 ou 2 vezes por semana	Nenhuma no último mês	Nenhuma no último mês
30	7h20	6	1 ou 2 vezes por semana	1 ou 2 vezes por semana	Nenhuma no último mês
0	8:00hrs	6-8 hrs	Menos de 1 vez por semana	Menos de 1 vez por semana	Menos de 1 vez por semana
15 minutos	04h50	De 6h a 7h	Nenhuma no último mês	Nenhuma no último mês	Menos de 1 vez por semana
Depende, as vezes hora[illegible]	08 h dependendo da[illegible]	De 4 a 6 horas	3 ou mais vezes por semana	3 ou mais vezes por semana	Nenhuma no último mês
Vinte minutos.	8 horas.	7	Menos de 1 vez por semana	Menos de 1 vez por semana	1 ou 2 vezes por semana
70	9	7	3 ou mais vezes por semana	Nenhuma no último mês	Menos de 1 vez por semana
90 min	8h	6h	Nenhuma no último mês	3 ou mais vezes por semana	3 ou mais vezes por semana
10	9 horas	6 horas	1 ou 2 vezes por semana	Nenhuma no último mês	Nenhuma no último mês
30min	10h	6h	1 ou 2 vezes por semana	Nenhuma no último mês	Nenhuma no último mês

10 min	8h	7h	1 ou 2 vezes por semana	Nenhuma no último mês	Menos de 1 vez por semana
30 minutos	8h 30	7h	1 ou 2 vezes por semana	Nenhuma no último mês	Nenhuma no último mês
60 minutos	08 h	7 horas	1 ou 2 vezes por semana	Menos de 1 vez por semana	Nenhuma no último mês
30	09:00	7	1 ou 2 vezes por semana	1 ou 2 vezes por semana	3 ou mais vezes por semana
15	7:30	8	Menos de 1 vez por semana	Menos de 1 vez por semana	Menos de 1 vez por semana
20 min	Sim, 9 am	6:34	Nenhuma no último mês	Menos de 1 vez por semana	Nenhuma no último mês
s de 1 hora , 60 min	7h oras	5 ou 6 horas	3 ou mais vezes por semana	3 ou mais vezes por semana	3 ou mais vezes por semana
Muito tempo	9h	7h	3 ou mais vezes por semana	1 ou 2 vezes por semana	Nenhuma no último mês
30 a 45 min	8h 10	de 7 a 8h	1 ou 2 vezes por semana	Menos de 1 vez por semana	3 ou mais vezes por semana
90	08	7h 30	3 ou mais vezes por semana	Nenhuma no último mês	Menos de 1 vez por semana
30m	10 hrs	Em média 7hrs	1 ou 2 vezes por semana	1 ou 2 vezes por semana	Nenhuma no último mês
30 minutos	7:50	6, 7 horas	Menos de 1 vez por semana	Menos de 1 vez por semana	Menos de 1 vez por semana

15	6:30	7h	Menos de 1 vez por semana	Nenhuma no último mês	Nenhuma no último mês
15 minutos	08h	7	1 ou 2 vezes por semana	1 ou 2 vezes por semana	Menos de 1 vez por semana
90m	6h30	5h	3 ou mais vezes por semana	1 ou 2 vezes por semana	Menos de 1 vez por semana
30	3	7	3 ou mais vezes por semana	3 ou mais vezes por semana	Nenhuma no último mês
1h	07h30	7h	3 ou mais vezes por semana	3 ou mais vezes por semana	Nenhuma no último mês
60 minutos	08:30	6 horas	1 ou 2 vezes por semana	3 ou mais vezes por semana	Nenhuma no último mês
90	08h30	8	3 ou mais vezes por semana	1 ou 2 vezes por semana	Nenhuma no último mês
20	8	7	1 ou 2 vezes por semana	Menos de 1 vez por semana	Nenhuma no último mês
40 minutos	07:30	6 horas	Nenhuma no último mês	Menos de 1 vez por semana	Nenhuma no último mês
[illegible]ca de 45minut	6h	Entre 6h e 7h	1 ou 2 vezes por semana	1 ou 2 vezes por semana	Menos de 1 vez por semana

2h	10 h	7h	3 ou mais vezes por semana	3 ou mais vezes por semana	3 ou mais vezes por semana
20 minutos	06:00	Em média 6hrs	3 ou mais vezes por semana	1 ou 2 vezes por semana	Menos de 1 vez por semana
Meia-hora	8h	6h30	1 ou 2 vezes por semana	Menos de 1 vez por semana	Menos de 1 vez por semana
30min	8h	6h	1 ou 2 vezes por semana	3 ou mais vezes por semana	3 ou mais vezes por semana
1 a 2 horas	5:55	[illegible] horas por [illegible]	1 ou 2 vezes por semana	3 ou mais vezes por semana	Nenhuma no último mês
60 minutos	6:30h	5:00h	1 ou 2 vezes por semana	1 ou 2 vezes por semana	1 ou 2 vezes por semana
40	12 h	7	3 ou mais vezes por semana	1 ou 2 vezes por semana	Nenhuma no último mês
20	08:30	7h	1 ou 2 vezes por semana	Nenhuma no último mês	Menos de 1 vez por semana
10 a 15 minutos	5h30	7h	Menos de 1 vez por semana	1 ou 2 vezes por semana	1 ou 2 vezes por semana
30 min	7h	7h	1 ou 2 vezes por semana	Menos de 1 vez por semana	Nenhuma no último mês
10minutos	7h30	8h	Nenhuma no último mês	Menos de 1 vez por semana	Nenhuma no último mês

1:30 hrs	8:00	8 a 9 horas	3 ou mais vezes por semana	Menos de 1 vez por semana	3 ou mais vezes por semana
5 minutos	7:30h	8h	Nenhuma no último mês	Nenhuma no último mês	Nenhuma no último mês
10	7	8 a 9 hrs	Menos de 1 vez por semana	Menos de 1 vez por semana	1 ou 2 vezes por semana
Mais de uma hora	8	7	1 ou 2 vezes por semana	Menos de 1 vez por semana	Menos de 1 vez por semana
10	06:00	8	1 ou 2 vezes por semana	Menos de 1 vez por semana	Menos de 1 vez por semana
10 minutos	06:30	Por volta de 9	Menos de 1 vez por semana	Menos de 1 vez por semana	Nenhuma no último mês
20 minutos	6h da manhã	8h de sono	Nenhuma no último mês	Nenhuma no último mês	Nenhuma no último mês
20 minutos	6h	7h	Menos de 1 vez por semana	Nenhuma no último mês	Menos de 1 vez por semana
60	09:00	7	3 ou mais vezes por semana	1 ou 2 vezes por semana	Menos de 1 vez por semana
10 minutos	[illegible]h da manh[illegible]	9h	Menos de 1 vez por semana	Menos de 1 vez por semana	1 ou 2 vezes por semana
30	07:30	7	1 ou 2 vezes por semana	1 ou 2 vezes por semana	1 ou 2 vezes por semana

De 30 minutos a 1 hora	8:00	De 6 a 8 horas	3 ou mais vezes por semana	1 ou 2 vezes por semana	Nenhuma no último mês
20 minutos	05:30 h	05 h 06 h	1 ou 2 vezes por semana	3 ou mais vezes por semana	1 ou 2 vezes por semana
20	7:50	8	Nenhuma no último mês	Nenhuma no último mês	Nenhuma no último mês
10 a 20 minutos	10 da manhã	7 a 8 horas	Nenhuma no último mês	3 ou mais vezes por semana	Nenhuma no último mês
30	7h	7	Menos de 1 vez por semana	1 ou 2 vezes por semana	Nenhuma no último mês
Uma hora ou mais	[illegible] horas da manhã [illegible]	5 a 6 horas	1 ou 2 vezes por semana	3 ou mais vezes por semana	Menos de 1 vez por semana
<15	07:30	7	Menos de 1 vez por semana	Menos de 1 vez por semana	Nenhuma no último mês
20	07:50	7	Nenhuma no último mês	Nenhuma no último mês	Menos de 1 vez por semana
1h	7h	7h	3 ou mais vezes por semana	1 ou 2 vezes por semana	1 ou 2 vezes por semana
Entre 10 a 15	7h00	6 horas	Menos de 1 vez por semana	Menos de 1 vez por semana	Nenhuma no último mês
20min	7h	6h	Nenhuma no último mês	Nenhuma no último mês	Nenhuma no último mês
+60 min	07 h	07 h a 8h	3 ou mais vezes por semana	3 ou mais vezes por semana	Nenhuma no último mês

ca de 20-30 minutos	Entre às 6:50 e 7:3[illegible]	stante, mas se	Nenhuma no último mês	Nenhuma no último mês	Nenhuma no último mês
25	8	7	1 ou 2 vezes por semana	3 ou mais vezes por semana	Menos de 1 vez por semana
de 5 a 15 min	8:00	7-9	Menos de 1 vez por semana	Menos de 1 vez por semana	Menos de 1 vez por semana
45	8	8	3 ou mais vezes por semana	Menos de 1 vez por semana	Nenhuma no último mês
00:15	10:00	08:00	Menos de 1 vez por semana	Menos de 1 vez por semana	Nenhuma no último mês
1h	9h	9	3 ou mais vezes por semana	3 ou mais vezes por semana	1 ou 2 vezes por semana
30-40 min	8:30	8h	Menos de 1 vez por semana	1 ou 2 vezes por semana	Nenhuma no último mês
90	7:30	6	3 ou mais vezes por semana	1 ou 2 vezes por semana	Menos de 1 vez por semana
30	9h 30	7	Nenhuma no último mês	3 ou mais vezes por semana	Menos de 1 vez por semana
30 minutos	7 da manhã	4h30 a 6h	3 ou mais vezes por semana	1 ou 2 vezes por semana	Menos de 1 vez por semana
80	07	08	3 ou mais vezes por semana	3 ou mais vezes por semana	3 ou mais vezes por semana
120	7:30	7	3 ou mais vezes por semana	Menos de 1 vez por semana	Nenhuma no último mês

20 minutos maximo	05	6	Nenhuma no último mês	Nenhuma no último mês	Nenhuma no último mês
60	06:00	4	3 ou mais vezes por semana	3 ou mais vezes por semana	1 ou 2 vezes por semana
20	7:00	7	Menos de 1 vez por semana	1 ou 2 vezes por semana	Menos de 1 vez por semana
30	09:00	8	1 ou 2 vezes por semana	1 ou 2 vezes por semana	3 ou mais vezes por semana
3 Minutos	7:30	7	Nenhuma no último mês	Menos de 1 vez por semana	Menos de 1 vez por semana
20-30 minutos	9:00	8 horas em média	Menos de 1 vez por semana	1 ou 2 vezes por semana	Menos de 1 vez por semana
Mais de uma hora	[illegible] de manhã. Meu [illegible]	5h	3 ou mais vezes por semana	Menos de 1 vez por semana	Nenhuma no último mês
150	08:30	7	1 ou 2 vezes por semana	Menos de 1 vez por semana	Menos de 1 vez por semana
[illegible] máximo vinte min[illegible]	Oito da manhã	[illegible]is a oito ho[illegible]	Menos de 1 vez por semana	Menos de 1 vez por semana	Nenhuma no último mês
10 minutos	8horas	7	Menos de 1 vez por semana	1 ou 2 vezes por semana	3 ou mais vezes por semana
40	8h	7h	3 ou mais vezes por semana	Nenhuma no último mês	1 ou 2 vezes por semana

de 30 min a 1h	o entendi essa pergunt	que fiquei acordadade	3 ou mais vezes por semana	3 ou mais vezes por semana	1 ou 2 vezes por semana
1h	10 h	7h	3 ou mais vezes por semana	Menos de 1 vez por semana	Nenhuma no último mês
60	08:00	6	1 ou 2 vezes por semana	1 ou 2 vezes por semana	Menos de 1 vez por semana
20 minutos	7 horas	6	Nenhuma no último mês	Nenhuma no último mês	Nenhuma no último mês
Em torno de 1h	7:30	8h	3 ou mais vezes por semana	3 ou mais vezes por semana	1 ou 2 vezes por semana
60min	9h	7 a 8	1 ou 2 vezes por semana	1 ou 2 vezes por semana	Nenhuma no último mês
240 m	8:30	6h 30 a 7h	3 ou mais vezes por semana	3 ou mais vezes por semana	3 ou mais vezes por semana
30	7:10	7	Nenhuma no último mês	3 ou mais vezes por semana	3 ou mais vezes por semana
15 minutos	08 horas	07h30	Nenhuma no último mês	Nenhuma no último mês	Nenhuma no último mês
15	05:30	7	Nenhuma no último mês	Nenhuma no último mês	Menos de 1 vez por semana

10min	5h	7h	Nenhuma no último mês	Menos de 1 vez por semana	Menos de 1 vez por semana
60	7:00	6	1 ou 2 vezes por semana	Menos de 1 vez por semana	Nenhuma no último mês
5 min	06:45	7h	Menos de 1 vez por semana	1 ou 2 vezes por semana	1 ou 2 vezes por semana
15	7:30	6	1 ou 2 vezes por semana	Nenhuma no último mês	Nenhuma no último mês
Meia hora	11 horas da manhã	7 horas	Menos de 1 vez por semana	Menos de 1 vez por semana	Nenhuma no último mês
5	7h	8	Menos de 1 vez por semana	Menos de 1 vez por semana	Nenhuma no último mês
60 minutos	6:00	5h e meia/6h	3 ou mais vezes por semana	1 ou 2 vezes por semana	1 ou 2 vezes por semana
20	6h30	5	Nenhuma no último mês	Nenhuma no último mês	Nenhuma no último mês
15min	7:30	8	Menos de 1 vez por semana	Nenhuma no último mês	Nenhuma no último mês
30	10:00	7	3 ou mais vezes por semana	Menos de 1 vez por semana	Nenhuma no último mês
5 mim	09h00	8h	Nenhuma no último mês	1 ou 2 vezes por semana	1 ou 2 vezes por semana

40	07:00	7	3 ou mais vezes por semana	1 ou 2 vezes por semana	Menos de 1 vez por semana
60	08:00	07	3 ou mais vezes por semana	Menos de 1 vez por semana	Nenhuma no último mês
30	8	10	1 ou 2 vezes por semana	3 ou mais vezes por semana	3 ou mais vezes por semana
Mais ou menos meia [illegible]	7h - 8h	Por volta de 7h	1 ou 2 vezes por semana	3 ou mais vezes por semana	3 ou mais vezes por semana
15 min	07:00	7	Nenhuma no último mês	Nenhuma no último mês	1 ou 2 vezes por semana
30min	06:30	7, 8 horas	1 ou 2 vezes por semana	1 ou 2 vezes por semana	3 ou mais vezes por semana
30min	06:30	7	1 ou 2 vezes por semana	Menos de 1 vez por semana	1 ou 2 vezes por semana
40	10	8	3 ou mais vezes por semana	1 ou 2 vezes por semana	Nenhuma no último mês
40min	7h	7h	3 ou mais vezes por semana	3 ou mais vezes por semana	1 ou 2 vezes por semana
30 minutos	08:00	8 horas	Menos de 1 vez por semana	3 ou mais vezes por semana	3 ou mais vezes por semana
30	7h	7	1 ou 2 vezes por semana	3 ou mais vezes por semana	3 ou mais vezes por semana

30min	6h	6h 30 mim	Menos de 1 vez por semana	Menos de 1 vez por semana	Menos de 1 vez por semana
30 minutos	7:30	6	Menos de 1 vez por semana	1 ou 2 vezes por semana	Nenhuma no último mês
Uma hora	7 da manhã	5	3 ou mais vezes por semana	Nenhuma no último mês	Nenhuma no último mês
Não sei	8h	7 horas	Nenhuma no último mês	Nenhuma no último mês	Nenhuma no último mês
15	8	8	Nenhuma no último mês	Menos de 1 vez por semana	Nenhuma no último mês
20 min	5:15	5h	1 ou 2 vezes por semana	Nenhuma no último mês	Nenhuma no último mês
2	8	6	Nenhuma no último mês	Nenhuma no último mês	Nenhuma no último mês
30min	7H	8h	1 ou 2 vezes por semana	Nenhuma no último mês	1 ou 2 vezes por semana
10 minutos	4h	5h	Menos de 1 vez por semana	Nenhuma no último mês	Nenhuma no último mês
30min	7hs	8hs	1 ou 2 vezes por semana	Nenhuma no último mês	1 ou 2 vezes por semana
[illegible] dia, mais ou menos [illegible]	[illegible]nde, 6:00- [illegible]	7 horas de so[illegible]	1 ou 2 vezes por semana	Menos de 1 vez por semana	Menos de 1 vez por semana
50	10:00	7	3 ou mais vezes por semana	1 ou 2 vezes por semana	Menos de 1 vez por semana

5	7:00	7,5 horas	Nenhuma no último mês	Menos de 1 vez por semana	Nenhuma no último mês
15 min	7h	6h	Menos de 1 vez por semana	Menos de 1 vez por semana	Nenhuma no último mês
40 min	7 hrs	6 a 7 hrs	Nenhuma no último mês	Menos de 1 vez por semana	Menos de 1 vez por semana
30	9 am	8h	1 ou 2 vezes por semana	Nenhuma no último mês	Menos de 1 vez por semana
40	7	8	Menos de 1 vez por semana	Menos de 1 vez por semana	Nenhuma no último mês
45	8	8	Menos de 1 vez por semana	Nenhuma no último mês	Menos de 1 vez por semana
15	06:00	6	1 ou 2 vezes por semana	3 ou mais vezes por semana	3 ou mais vezes por semana
1h	06h30	5h	3 ou mais vezes por semana	3 ou mais vezes por semana	3 ou mais vezes por semana
10	6:20	8	Menos de 1 vez por semana	Menos de 1 vez por semana	Menos de 1 vez por semana
de 30 a 60	9hr	6-7	3 ou mais vezes por semana	3 ou mais vezes por semana	Nenhuma no último mês
40 min no minimo	8h	6h	3 ou mais vezes por semana	3 ou mais vezes por semana	3 ou mais vezes por semana
0	6:00	8	Nenhuma no último mês	Menos de 1 vez por semana	Nenhuma no último mês

60	6h	7h	Menos de 1 vez por semana	Menos de 1 vez por semana	Nenhuma no último mês
10	06:30	De 6 a 7 horas	Menos de 1 vez por semana	Menos de 1 vez por semana	Nenhuma no último mês
2 horas	9:00h	6 horas	3 ou mais vezes por semana	3 ou mais vezes por semana	Menos de 1 vez por semana
40	8h	5	Menos de 1 vez por semana	3 ou mais vezes por semana	Menos de 1 vez por semana
30 min	05:45 h	6h	1 ou 2 vezes por semana	1 ou 2 vezes por semana	1 ou 2 vezes por semana
30 minutos	08:00	9h	Menos de 1 vez por semana	1 ou 2 vezes por semana	3 ou mais vezes por semana
15 min.	às 8hs.	7 a 9 horas	Nenhuma no último mês	Menos de 1 vez por semana	Nenhuma no último mês
15	8:00	8	1 ou 2 vezes por semana	1 ou 2 vezes por semana	Menos de 1 vez por semana
60	09:50	7:30	1 ou 2 vezes por semana	1 ou 2 vezes por semana	3 ou mais vezes por semana
10	7	7	Nenhuma no último mês	Nenhuma no último mês	Nenhuma no último mês
30 min	7:30	7h	1 ou 2 vezes por semana	Menos de 1 vez por semana	Nenhuma no último mês

60 min	08h40min	Entre 5h e 6h	3 ou mais vezes por semana	Menos de 1 vez por semana	Nenhuma no último mês
30	09:00	06:30	Menos de 1 vez por semana	Nenhuma no último mês	Nenhuma no último mês
demoro pra dormir dep	5h40min	7h40min	Nenhuma no último mês	Nenhuma no último mês	Nenhuma no último mês
20 minutos	6:40	8	Menos de 1 vez por semana	1 ou 2 vezes por semana	Menos de 1 vez por semana
3h a 4h.	7h20min	5h a 6h	1 ou 2 vezes por semana	3 ou mais vezes por semana	Nenhuma no último mês
180	12	8	3 ou mais vezes por semana	3 ou mais vezes por semana	3 ou mais vezes por semana
15-30	7h	6-8	3 ou mais vezes por semana	1 ou 2 vezes por semana	1 ou 2 vezes por semana
20	06:00	8	Nenhuma no último mês	1 ou 2 vezes por semana	Nenhuma no último mês
30	07	07	Menos de 1 vez por semana	Menos de 1 vez por semana	Nenhuma no último mês
1 hora	7:30	8 hrs	1 ou 2 vezes por semana	Menos de 1 vez por semana	Nenhuma no último mês
10 minutos	8 da manhã	7 horas	Nenhuma no último mês	Menos de 1 vez por semana	1 ou 2 vezes por semana

1 hr	7:30	7	3 ou mais vezes por semana	3 ou mais vezes por semana	Menos de 1 vez por semana
10min	6h	8h	Menos de 1 vez por semana	Menos de 1 vez por semana	Menos de 1 vez por semana
5 minutos	[illegible]s os dias às [illegible]	Umas 7horas	Nenhuma no último mês	3 ou mais vezes por semana	Nenhuma no último mês
15	7:30	6	1 ou 2 vezes por semana	1 ou 2 vezes por semana	Nenhuma no último mês
60	9h	6h30	1 ou 2 vezes por semana	Menos de 1 vez por semana	Nenhuma no último mês
30	8:00	7:30	Menos de 1 vez por semana	Menos de 1 vez por semana	1 ou 2 vezes por semana
15 minutos	7 horas	[illegible]oras e 30 minut[illegible]	1 ou 2 vezes por semana	3 ou mais vezes por semana	Menos de 1 vez por semana
5	6:45	8	Nenhuma no último mês	Nenhuma no último mês	Nenhuma no último mês
15 min	7:00	6	Menos de 1 vez por semana	Nenhuma no último mês	Nenhuma no último mês
30 min	5:00 hr	6 horas	1 ou 2 vezes por semana	1 ou 2 vezes por semana	Menos de 1 vez por semana
60 minutos	9:00	7 a 8 horas	3 ou mais vezes por semana	3 ou mais vezes por semana	Menos de 1 vez por semana

30	10	6	1 ou 2 vezes por semana	Nenhuma no último mês	Nenhuma no último mês
+/- 20	8h	7h	1 ou 2 vezes por semana	1 ou 2 vezes por semana	1 ou 2 vezes por semana
30 minutos	[illegible] horas da manhã	5 a 6 horas	1 ou 2 vezes por semana	3 ou mais vezes por semana	1 ou 2 vezes por semana
20	7h	6	1 ou 2 vezes por semana	3 ou mais vezes por semana	1 ou 2 vezes por semana
15	7:00	7	Menos de 1 vez por semana	Nenhuma no último mês	Nenhuma no último mês
120	08:00	6	3 ou mais vezes por semana	3 ou mais vezes por semana	Menos de 1 vez por semana
Cerca de 30 minutos	8h da manhã	8h	Menos de 1 vez por semana	Menos de 1 vez por semana	Menos de 1 vez por semana
1 hr	10	5 a 6 hrs	3 ou mais vezes por semana	3 ou mais vezes por semana	Menos de 1 vez por semana
90 minutos	5h 50 min	6 horas	1 ou 2 vezes por semana	1 ou 2 vezes por semana	1 ou 2 vezes por semana
50 minutos	05:30	6 horas	3 ou mais vezes por semana	3 ou mais vezes por semana	Menos de 1 vez por semana
5-10min	7-7:30	7-8horas	Nenhuma no último mês	Nenhuma no último mês	Menos de 1 vez por semana
30	6:00	6	1 ou 2 vezes por semana	3 ou mais vezes por semana	Menos de 1 vez por semana

10	6hrs	7 hrs	Nenhuma no último mês	Nenhuma no último mês	Menos de 1 vez por semana
30	5	6	1 ou 2 vezes por semana	1 ou 2 vezes por semana	Menos de 1 vez por semana
30	6	7	1 ou 2 vezes por semana	1 ou 2 vezes por semana	Menos de 1 vez por semana
10	7	6	Nenhuma no último mês	Nenhuma no último mês	Nenhuma no último mês
00:15	05:00	6:00	Nenhuma no último mês	Menos de 1 vez por semana	Menos de 1 vez por semana
30	8h	7h	Nenhuma no último mês	Nenhuma no último mês	Nenhuma no último mês
30/45 minutos	[illegible] horas da manh[illegible]	Cerca de 4 horas	3 ou mais vezes por semana	3 ou mais vezes por semana	Nenhuma no último mês
30	8h30	7h	1 ou 2 vezes por semana	1 ou 2 vezes por semana	Nenhuma no último mês
6 a 8 horas	Sim 6:30	6 a 8 horas	1 ou 2 vezes por semana	1 ou 2 vezes por semana	3 ou mais vezes por semana
[illegible]co tempo menos que 15	05:30	5 a 6 hrs	Menos de 1 vez por semana	Menos de 1 vez por semana	Nenhuma no último mês
30:00	8:00	6	Menos de 1 vez por semana	Menos de 1 vez por semana	Menos de 1 vez por semana

Dez minutos	8 horas	7 horas	Menos de 1 vez por semana	Menos de 1 vez por semana	3 ou mais vezes por semana
[illegible] ava no sono rápido e acordava no mei [illegible]	8 horas	chá forte de maracuja e val [illegible]	3 ou mais vezes por semana	3 ou mais vezes por semana	Menos de 1 vez por semana
15 min	6h	5h	1 ou 2 vezes por semana	3 ou mais vezes por semana	Menos de 1 vez por semana
10	7:30	6/7	Menos de 1 vez por semana	3 ou mais vezes por semana	1 ou 2 vezes por semana
60	6	7	1 ou 2 vezes por semana	1 ou 2 vezes por semana	3 ou mais vezes por semana
30 minutos	7:00	7:30	Menos de 1 vez por semana	Menos de 1 vez por semana	Nenhuma no último mês
15	06:30	5	1 ou 2 vezes por semana	Menos de 1 vez por semana	Nenhuma no último mês
60	06h30	8h	1 ou 2 vezes por semana	3 ou mais vezes por semana	Menos de 1 vez por semana
10	4	5	Nenhuma no último mês	3 ou mais vezes por semana	Nenhuma no último mês
60 a 120 minutos	7 hs	6 horas	3 ou mais vezes por semana	3 ou mais vezes por semana	1 ou 2 vezes por semana

30 m a 1h	09	5h	3 ou mais vezes por semana	3 ou mais vezes por semana	3 ou mais vezes por semana
20	6h 30	7	Menos de 1 vez por semana	Menos de 1 vez por semana	3 ou mais vezes por semana
15 minutos	6:00	8	Menos de 1 vez por semana	Menos de 1 vez por semana	1 ou 2 vezes por semana
40	06:30	6	1 ou 2 vezes por semana	1 ou 2 vezes por semana	1 ou 2 vezes por semana
5	8	8	Nenhuma no último mês	Menos de 1 vez por semana	Nenhuma no último mês
10-20 min	10 h	7-8h	Nenhuma no último mês	1 ou 2 vezes por semana	Nenhuma no último mês
60	7h ou 7h 30	Cerca de 7h ou 6h	1 ou 2 vezes por semana	Nenhuma no último mês	Nenhuma no último mês
30 min	8h e 30 min	7h	Menos de 1 vez por semana	Nenhuma no último mês	Nenhuma no último mês
20min	7am	6h	3 ou mais vezes por semana	Menos de 1 vez por semana	Nenhuma no último mês
120	7	6	3 ou mais vezes por semana	3 ou mais vezes por semana	3 ou mais vezes por semana
180	7	9	Menos de 1 vez por semana	Menos de 1 vez por semana	3 ou mais vezes por semana

30	8	6-8	Nenhuma no último mês	Nenhuma no último mês	Nenhuma no último mês
15min	8hs	8 hs	Menos de 1 vez por semana	Menos de 1 vez por semana	Nenhuma no último mês
60	7	6	3 ou mais vezes por semana	1 ou 2 vezes por semana	Nenhuma no último mês
1 hora	4h 30	5 horas	3 ou mais vezes por semana	1 ou 2 vezes por semana	Nenhuma no último mês
30min	8h	7h	Nenhuma no último mês	Nenhuma no último mês	3 ou mais vezes por semana
mais de 1(uma) hora	08 horas	06 horas	3 ou mais vezes por semana	3 ou mais vezes por semana	3 ou mais vezes por semana
30 minutos	08 horas	05 horas	Menos de 1 vez por semana	3 ou mais vezes por semana	Menos de 1 vez por semana
60	7	6/7	1 ou 2 vezes por semana	1 ou 2 vezes por semana	Menos de 1 vez por semana
10	10	10	Nenhuma no último mês	Nenhuma no último mês	Nenhuma no último mês
20 minutos	5 horas	4 horas	1 ou 2 vezes por semana	3 ou mais vezes por semana	3 ou mais vezes por semana
1a 2h	6h	4h	3 ou mais vezes por semana	3 ou mais vezes por semana	3 ou mais vezes por semana

60	7:40	7h	3 ou mais vezes por semana	3 ou mais vezes por semana	3 ou mais vezes por semana
40	9	5	1 ou 2 vezes por semana	1 ou 2 vezes por semana	Menos de 1 vez por semana
30	7h	7h	1 ou 2 vezes por semana	Menos de 1 vez por semana	Menos de 1 vez por semana
120 min	6h	5h	3 ou mais vezes por semana	3 ou mais vezes por semana	Nenhuma no último mês
30 minutos	7:30 hrs	6 hrs	1 ou 2 vezes por semana	1 ou 2 vezes por semana	Menos de 1 vez por semana
Até 40 minutos	07:00	do. Estimo entre	1 ou 2 vezes por semana	Nenhuma no último mês	Nenhuma no último mês
1	7	6	3 ou mais vezes por semana	1 ou 2 vezes por semana	3 ou mais vezes por semana
1 hora	8 horas	8 horas	3 ou mais vezes por semana	1 ou 2 vezes por semana	Menos de 1 vez por semana
1h/2h	5/6:00	4/5	3 ou mais vezes por semana	3 ou mais vezes por semana	1 ou 2 vezes por semana
15	7h 30	6h	1 ou 2 vezes por semana	1 ou 2 vezes por semana	Nenhuma no último mês

Poucos minutos	05:30	7 horas	Nenhuma no último mês	Nenhuma no último mês	Nenhuma no último mês
0h30	7h30	7 horas	Menos de 1 vez por semana	Menos de 1 vez por semana	Menos de 1 vez por semana
30 min	8h45	8h	1 ou 2 vezes por semana	Nenhuma no último mês	Nenhuma no último mês
120	Sempre	6	3 ou mais vezes por semana	3 ou mais vezes por semana	3 ou mais vezes por semana
nte mais de 2 horas	11h	4horas	3 ou mais vezes por semana	3 ou mais vezes por semana	3 ou mais vezes por semana
30	6:30	7h	1 ou 2 vezes por semana	Nenhuma no último mês	Nenhuma no último mês
30	10	6	3 ou mais vezes por semana	1 ou 2 vezes por semana	1 ou 2 vezes por semana
30 min	6:20	8	Menos de 1 vez por semana	Menos de 1 vez por semana	Nenhuma no último mês
15	6	6-7	Menos de 1 vez por semana	3 ou mais vezes por semana	Nenhuma no último mês
5	06:00	Entre 6-7h	Nenhuma no último mês	Nenhuma no último mês	Menos de 1 vez por semana
10min	07 e 08h da ma	7	Menos de 1 vez por semana	Menos de 1 vez por semana	Nenhuma no último mês
10	30	5	Nenhuma no último mês	Menos de 1 vez por semana	Menos de 1 vez por semana

30m	7h	6h	1 ou 2 vezes por semana	3 ou mais vezes por semana	Menos de 1 vez por semana
60	7	6	1 ou 2 vezes por semana	1 ou 2 vezes por semana	1 ou 2 vezes por semana
10 min	8:30	8 horas	Menos de 1 vez por semana	Menos de 1 vez por semana	1 ou 2 vezes por semana
15	11:30	8	Menos de 1 vez por semana	Nenhuma no último mês	Nenhuma no último mês
40 minutos	6:30	Geralmente 6 hs	3 ou mais vezes por semana	1 ou 2 vezes por semana	1 ou 2 vezes por semana
30	7	6	Menos de 1 vez por semana	3 ou mais vezes por semana	Nenhuma no último mês
10 minutos	6 horas	7 horas	1 ou 2 vezes por semana	1 ou 2 vezes por semana	1 ou 2 vezes por semana
30 minutos	6 horas	6 a 7 horas	Menos de 1 vez por semana	Menos de 1 vez por semana	Menos de 1 vez por semana
20	07:30	7 a 8	1 ou 2 vezes por semana	1 ou 2 vezes por semana	3 ou mais vezes por semana
60	6:30	8 horas	1 ou 2 vezes por semana	Menos de 1 vez por semana	Menos de 1 vez por semana
1h	5:30	7	3 ou mais vezes por semana	Menos de 1 vez por semana	3 ou mais vezes por semana
20	6h	6	3 ou mais vezes por semana	3 ou mais vezes por semana	Nenhuma no último mês

20 min	6:10	4 horas	Nenhuma no último mês	Nenhuma no último mês	Nenhuma no último mês
60	7.00	5 a 7	3 ou mais vezes por semana	1 ou 2 vezes por semana	3 ou mais vezes por semana
30-40	8h	6/7 h	1 ou 2 vezes por semana	3 ou mais vezes por semana	Menos de 1 vez por semana
10 minutos em média.	Todos os dias.	[illegible] horas em média	Nenhuma no último mês	Nenhuma no último mês	Menos de 1 vez por semana
30	05:00	8	Nenhuma no último mês	3 ou mais vezes por semana	Nenhuma no último mês
10 min	6h	de 6h à 7h	1 ou 2 vezes por semana	3 ou mais vezes por semana	3 ou mais vezes por semana
30 minutos	5:30	4:00 na média	1 ou 2 vezes por semana	3 ou mais vezes por semana	3 ou mais vezes por semana
30	5h 00	5h 30	Menos de 1 vez por semana	Nenhuma no último mês	Nenhuma no último mês
20 a 30 minutos	7:15	6 a 7 horas	Menos de 1 vez por semana	1 ou 2 vezes por semana	Nenhuma no último mês
10min	5:30	6h	Menos de 1 vez por semana	Menos de 1 vez por semana	Menos de 1 vez por semana
[illegible] mo em função do [illegible]	06 h	De 5 a 6h	Nenhuma no último mês	Nenhuma no último mês	Nenhuma no último mês
20-30h	7h	6	3 ou mais vezes por semana	3 ou mais vezes por semana	Menos de 1 vez por semana

10	07 horas	8	Nenhuma no último mês	Menos de 1 vez por semana	Menos de 1 vez por semana
30MIN	7H00	6H00 A 7H00	Menos de 1 vez por semana	Menos de 1 vez por semana	1 ou 2 vezes por semana
15	7h	5h	Menos de 1 vez por semana	3 ou mais vezes por semana	Nenhuma no último mês
30 a 40 min	Entre 8 e 9h	7h	3 ou mais vezes por semana	1 ou 2 vezes por semana	Nenhuma no último mês
30 minutos	7h	8	1 ou 2 vezes por semana	Menos de 1 vez por semana	1 ou 2 vezes por semana
10min	6h	8	1 ou 2 vezes por semana	Menos de 1 vez por semana	Nenhuma no último mês
15 minutos	7h30	8h	Nenhuma no último mês	Menos de 1 vez por semana	Nenhuma no último mês
120 minutos	7:00	6 horas	3 ou mais vezes por semana	3 ou mais vezes por semana	3 ou mais vezes por semana
10 minutos	7:30h	5 a 6h	Menos de 1 vez por semana	3 ou mais vezes por semana	3 ou mais vezes por semana
10	7h	7	Nenhuma no último mês	3 ou mais vezes por semana	3 ou mais vezes por semana
1 hora	5:45	6 horas	3 ou mais vezes por semana	3 ou mais vezes por semana	Nenhuma no último mês
30 min	06 h	6-7h	Nenhuma no último mês	1 ou 2 vezes por semana	Nenhuma no último mês

120	7	6	3 ou mais vezes por semana	Nenhuma no último mês	Nenhuma no último mês
15	6:40	6 h	Menos de 1 vez por semana	1 ou 2 vezes por semana	1 ou 2 vezes por semana
15	6	8	Nenhuma no último mês	1 ou 2 vezes por semana	1 ou 2 vezes por semana
10 a 15 minutos.	06:00	6	Nenhuma no último mês	Menos de 1 vez por semana	Nenhuma no último mês
15	mpre acordo 6:3	9h	Nenhuma no último mês	Nenhuma no último mês	3 ou mais vezes por semana
5	8	7	Nenhuma no último mês	Nenhuma no último mês	Nenhuma no último mês
5	6	6	Menos de 1 vez por semana	1 ou 2 vezes por semana	3 ou mais vezes por semana
2	7.30	6h	Nenhuma no último mês	Nenhuma no último mês	Nenhuma no último mês
20	7	8	Nenhuma no último mês	Nenhuma no último mês	Nenhuma no último mês
10 minutos	7h	6 horas	Nenhuma no último mês	1 ou 2 vezes por semana	Nenhuma no último mês
10 minutos	6.00	8.00	Nenhuma no último mês	1 ou 2 vezes por semana	1 ou 2 vezes por semana
20 min	7 hs	6 hs	Nenhuma no último mês	Nenhuma no último mês	Nenhuma no último mês

1h	9	5	3 ou mais vezes por semana	Menos de 1 vez por semana	Nenhuma no último mês
:20	7:00	6	Menos de 1 vez por semana	3 ou mais vezes por semana	Nenhuma no último mês
30 minutos	6h30	6h	Menos de 1 vez por semana	Menos de 1 vez por semana	Menos de 1 vez por semana
50	6	6	3 ou mais vezes por semana	3 ou mais vezes por semana	3 ou mais vezes por semana
15 minutos	7h30	8 horas	Nenhuma no último mês	Nenhuma no último mês	Nenhuma no último mês

Durante o último mês, com que frequência você teve dificuldade de dormir porque não conseguiu respirar confortavelmente?	Durante o último mês, com que frequência você teve dificuldade de dormir porque tossiu ou roncou forte?	Durante o último mês, com que frequência você teve dificuldade de dormir porque sentiu muito frio?
Nenhuma no último mês	Nenhuma no último mês	Menos de 1 vez por semana
Nenhuma no último mês	Nenhuma no último mês	Nenhuma no último mês
1 ou 2 vezes por semana	Nenhuma no último mês	Nenhuma no último mês
1 ou 2 vezes por semana	Nenhuma no último mês	Nenhuma no último mês
Nenhuma no último mês	Nenhuma no último mês	Menos de 1 vez por semana
Nenhuma no último mês	Nenhuma no último mês	Nenhuma no último mês
Nenhuma no último mês	Nenhuma no último mês	Menos de 1 vez por semana
Nenhuma no último mês	Nenhuma no último mês	Nenhuma no último mês
Nenhuma no último mês	Nenhuma no último mês	Nenhuma no último mês

3 ou mais vezes por semana	3 ou mais vezes por semana	Nenhuma no último mês
Nenhuma no último mês	Nenhuma no último mês	Nenhuma no último mês
Nenhuma no último mês	Nenhuma no último mês	Menos de 1 vez por semana
Nenhuma no último mês	Nenhuma no último mês	Nenhuma no último mês
Menos de 1 vez por semana	Nenhuma no último mês	Nenhuma no último mês
3 ou mais vezes por semana	Nenhuma no último mês	Nenhuma no último mês
Nenhuma no último mês	Nenhuma no último mês	Nenhuma no último mês
Nenhuma no último mês	Nenhuma no último mês	Menos de 1 vez por semana
Nenhuma no último mês	Nenhuma no último mês	Nenhuma no último mês
Nenhuma no último mês	Nenhuma no último mês	1 ou 2 vezes por semana
Nenhuma no último mês	Nenhuma no último mês	Nenhuma no último mês

Nenhuma no último mês	Nenhuma no último mês	Nenhuma no último mês
Nenhuma no último mês	Nenhuma no último mês	Menos de 1 vez por semana
Menos de 1 vez por semana	Menos de 1 vez por semana	Menos de 1 vez por semana
Nenhuma no último mês	Nenhuma no último mês	Menos de 1 vez por semana
Nenhuma no último mês	Nenhuma no último mês	Menos de 1 vez por semana
Nenhuma no último mês	3 ou mais vezes por semana	Nenhuma no último mês
Nenhuma no último mês	Nenhuma no último mês	1 ou 2 vezes por semana
Nenhuma no último mês	Nenhuma no último mês	Nenhuma no último mês
Nenhuma no último mês	Nenhuma no último mês	Nenhuma no último mês
Nenhuma no último mês	Nenhuma no último mês	Nenhuma no último mês

Menos de 1 vez por semana	Menos de 1 vez por semana	Nenhuma no último mês
Nenhuma no último mês	Nenhuma no último mês	Nenhuma no último mês
Nenhuma no último mês	Nenhuma no último mês	Nenhuma no último mês
Nenhuma no último mês	Nenhuma no último mês	Nenhuma no último mês
Menos de 1 vez por semana	Nenhuma no último mês	Nenhuma no último mês
Nenhuma no último mês	Nenhuma no último mês	Nenhuma no último mês
Menos de 1 vez por semana	3 ou mais vezes por semana	1 ou 2 vezes por semana
Nenhuma no último mês	Nenhuma no último mês	Nenhuma no último mês
Nenhuma no último mês	Nenhuma no último mês	1 ou 2 vezes por semana
Nenhuma no último mês	Nenhuma no último mês	Nenhuma no último mês
Nenhuma no último mês	Nenhuma no último mês	Menos de 1 vez por semana
Nenhuma no último mês	Nenhuma no último mês	Menos de 1 vez por semana

Nenhuma no último mês	Nenhuma no último mês	Menos de 1 vez por semana
Nenhuma no último mês	Nenhuma no último mês	Menos de 1 vez por semana
Menos de 1 vez por semana	Nenhuma no último mês	Menos de 1 vez por semana
Nenhuma no último mês	Nenhuma no último mês	Menos de 1 vez por semana
Nenhuma no último mês	Nenhuma no último mês	Nenhuma no último mês
Nenhuma no último mês	Nenhuma no último mês	Nenhuma no último mês
Nenhuma no último mês	Nenhuma no último mês	Menos de 1 vez por semana
Nenhuma no último mês	Nenhuma no último mês	Nenhuma no último mês
Menos de 1 vez por semana	Nenhuma no último mês	Nenhuma no último mês
Menos de 1 vez por semana	Nenhuma no último mês	Nenhuma no último mês

Nenhuma no último mês	Nenhuma no último mês	Menos de 1 vez por semana
Nenhuma no último mês	Nenhuma no último mês	Nenhuma no último mês
1 ou 2 vezes por semana	Menos de 1 vez por semana	Nenhuma no último mês
3 ou mais vezes por semana	Nenhuma no último mês	Nenhuma no último mês
Nenhuma no último mês	Nenhuma no último mês	Nenhuma no último mês
3 ou mais vezes por semana	1 ou 2 vezes por semana	1 ou 2 vezes por semana
Nenhuma no último mês	Nenhuma no último mês	Nenhuma no último mês
Nenhuma no último mês	Nenhuma no último mês	Nenhuma no último mês
Nenhuma no último mês	Nenhuma no último mês	Nenhuma no último mês
1 ou 2 vezes por semana	Nenhuma no último mês	Menos de 1 vez por semana
Menos de 1 vez por semana	Nenhuma no último mês	Nenhuma no último mês

Nenhuma no último mês	Nenhuma no último mês	1 ou 2 vezes por semana
Nenhuma no último mês	Menos de 1 vez por semana	Nenhuma no último mês
Nenhuma no último mês	Nenhuma no último mês	Menos de 1 vez por semana
Nenhuma no último mês	Menos de 1 vez por semana	Nenhuma no último mês
Nenhuma no último mês	Nenhuma no último mês	Nenhuma no último mês
Nenhuma no último mês	Nenhuma no último mês	3 ou mais vezes por semana
Nenhuma no último mês	Nenhuma no último mês	Nenhuma no último mês
Nenhuma no último mês	Nenhuma no último mês	Nenhuma no último mês
Nenhuma no último mês	Nenhuma no último mês	Nenhuma no último mês
Nenhuma no último mês	Nenhuma no último mês	Nenhuma no último mês
1 ou 2 vezes por semana	1 ou 2 vezes por semana	Nenhuma no último mês

1 ou 2 vezes por semana	Nenhuma no último mês	Nenhuma no último mês
Nenhuma no último mês	Nenhuma no último mês	Nenhuma no último mês
Nenhuma no último mês	Menos de 1 vez por semana	Nenhuma no último mês
Nenhuma no último mês	Nenhuma no último mês	Nenhuma no último mês
Nenhuma no último mês	Nenhuma no último mês	Menos de 1 vez por semana
Menos de 1 vez por semana	Menos de 1 vez por semana	Menos de 1 vez por semana
Nenhuma no último mês	Nenhuma no último mês	Menos de 1 vez por semana
Nenhuma no último mês	Nenhuma no último mês	Nenhuma no último mês
Nenhuma no último mês	Nenhuma no último mês	Nenhuma no último mês
Nenhuma no último mês	Nenhuma no último mês	Nenhuma no último mês
Nenhuma no último mês	Nenhuma no último mês	Nenhuma no último mês
Nenhuma no último mês	Menos de 1 vez por semana	Menos de 1 vez por semana

Nenhuma no último mês	Nenhuma no último mês	Nenhuma no último mês
Nenhuma no último mês	Nenhuma no último mês	Nenhuma no último mês
Nenhuma no último mês	Nenhuma no último mês	Menos de 1 vez por semana
Nenhuma no último mês	Nenhuma no último mês	Nenhuma no último mês
Menos de 1 vez por semana	Nenhuma no último mês	Nenhuma no último mês
Nenhuma no último mês	Nenhuma no último mês	Nenhuma no último mês
Nenhuma no último mês	Nenhuma no último mês	Menos de 1 vez por semana
1 ou 2 vezes por semana	Nenhuma no último mês	Menos de 1 vez por semana
Nenhuma no último mês	Nenhuma no último mês	Nenhuma no último mês
Nenhuma no último mês	Nenhuma no último mês	1 ou 2 vezes por semana
Menos de 1 vez por semana	Nenhuma no último mês	1 ou 2 vezes por semana
Nenhuma no último mês	Nenhuma no último mês	Nenhuma no último mês

Nenhuma no último mês	Nenhuma no último mês	Nenhuma no último mês
Menos de 1 vez por semana	Menos de 1 vez por semana	1 ou 2 vezes por semana
Menos de 1 vez por semana	Nenhuma no último mês	Nenhuma no último mês
Menos de 1 vez por semana	1 ou 2 vezes por semana	Nenhuma no último mês
Nenhuma no último mês	Nenhuma no último mês	Menos de 1 vez por semana
Nenhuma no último mês	Nenhuma no último mês	Nenhuma no último mês
1 ou 2 vezes por semana	Nenhuma no último mês	Nenhuma no último mês
Nenhuma no último mês	Nenhuma no último mês	Menos de 1 vez por semana
Nenhuma no último mês	Nenhuma no último mês	3 ou mais vezes por semana
Nenhuma no último mês	Nenhuma no último mês	Nenhuma no último mês
Nenhuma no último mês	Nenhuma no último mês	Nenhuma no último mês

Nenhuma no último mês	Nenhuma no último mês	Nenhuma no último mês
1 ou 2 vezes por semana	3 ou mais vezes por semana	Nenhuma no último mês
1 ou 2 vezes por semana	Nenhuma no último mês	Nenhuma no último mês
Nenhuma no último mês	Nenhuma no último mês	Nenhuma no último mês
Nenhuma no último mês	Nenhuma no último mês	Nenhuma no último mês
Nenhuma no último mês	Nenhuma no último mês	Menos de 1 vez por semana
1 ou 2 vezes por semana	Nenhuma no último mês	Menos de 1 vez por semana
Menos de 1 vez por semana	Nenhuma no último mês	Nenhuma no último mês
Nenhuma no último mês	Nenhuma no último mês	Nenhuma no último mês
Nenhuma no último mês	Nenhuma no último mês	Nenhuma no último mês

Nenhuma no último mês	Nenhuma no último mês	Nenhuma no último mês
Nenhuma no último mês	Nenhuma no último mês	Nenhuma no último mês
Nenhuma no último mês	Nenhuma no último mês	Nenhuma no último mês
Nenhuma no último mês	Nenhuma no último mês	Nenhuma no último mês
Nenhuma no último mês	Nenhuma no último mês	Menos de 1 vez por semana
Nenhuma no último mês	Nenhuma no último mês	Menos de 1 vez por semana
Nenhuma no último mês	Nenhuma no último mês	Nenhuma no último mês
Nenhuma no último mês	Menos de 1 vez por semana	Nenhuma no último mês
Nenhuma no último mês	Nenhuma no último mês	Nenhuma no último mês
Nenhuma no último mês	Nenhuma no último mês	Menos de 1 vez por semana
Nenhuma no último mês	3 ou mais vezes por semana	Nenhuma no último mês

Nenhuma no último mês	Nenhuma no último mês	Nenhuma no último mês
Nenhuma no último mês	Nenhuma no último mês	Nenhuma no último mês
3 ou mais vezes por semana	3 ou mais vezes por semana	Menos de 1 vez por semana
Nenhuma no último mês	Nenhuma no último mês	Nenhuma no último mês
Nenhuma no último mês	Nenhuma no último mês	Nenhuma no último mês
Nenhuma no último mês	Nenhuma no último mês	Menos de 1 vez por semana
Nenhuma no último mês	Nenhuma no último mês	Menos de 1 vez por semana
Nenhuma no último mês	Nenhuma no último mês	1 ou 2 vezes por semana
1 ou 2 vezes por semana	Nenhuma no último mês	Nenhuma no último mês
3 ou mais vezes por semana	Nenhuma no último mês	Nenhuma no último mês
Menos de 1 vez por semana	Menos de 1 vez por semana	Nenhuma no último mês

Nenhuma no último mês	Nenhuma no último mês	Nenhuma no último mês
Menos de 1 vez por semana	Nenhuma no último mês	Nenhuma no último mês
1 ou 2 vezes por semana	Nenhuma no último mês	Nenhuma no último mês
Nenhuma no último mês	Nenhuma no último mês	Nenhuma no último mês
Menos de 1 vez por semana	Nenhuma no último mês	Nenhuma no último mês
Nenhuma no último mês	Nenhuma no último mês	Nenhuma no último mês
Nenhuma no último mês	Nenhuma no último mês	Nenhuma no último mês
Nenhuma no último mês	Nenhuma no último mês	Nenhuma no último mês
Nenhuma no último mês	Nenhuma no último mês	Nenhuma no último mês
Nenhuma no último mês	Menos de 1 vez por semana	3 ou mais vezes por semana
Nenhuma no último mês	Nenhuma no último mês	Nenhuma no último mês
Nenhuma no último mês	Nenhuma no último mês	Nenhuma no último mês

Nenhuma no último mês	Nenhuma no último mês	Nenhuma no último mês
Nenhuma no último mês	Nenhuma no último mês	Menos de 1 vez por semana
Nenhuma no último mês	Nenhuma no último mês	Nenhuma no último mês
Nenhuma no último mês	Nenhuma no último mês	Nenhuma no último mês
Menos de 1 vez por semana	1 ou 2 vezes por semana	Nenhuma no último mês
Nenhuma no último mês	Nenhuma no último mês	Menos de 1 vez por semana
Menos de 1 vez por semana	Nenhuma no último mês	Nenhuma no último mês
Nenhuma no último mês	Nenhuma no último mês	Nenhuma no último mês
Nenhuma no último mês	Nenhuma no último mês	Nenhuma no último mês
Nenhuma no último mês	Nenhuma no último mês	Nenhuma no último mês
1 ou 2 vezes por semana	Nenhuma no último mês	3 ou mais vezes por semana
Nenhuma no último mês	Nenhuma no último mês	Nenhuma no último mês

Nenhuma no último mês	Nenhuma no último mês	Menos de 1 vez por semana
Nenhuma no último mês	Nenhuma no último mês	Nenhuma no último mês
Nenhuma no último mês	Nenhuma no último mês	1 ou 2 vezes por semana
Menos de 1 vez por semana	Nenhuma no último mês	Nenhuma no último mês
Nenhuma no último mês	Nenhuma no último mês	Menos de 1 vez por semana
Nenhuma no último mês	Menos de 1 vez por semana	Nenhuma no último mês
Nenhuma no último mês	1 ou 2 vezes por semana	Nenhuma no último mês
Nenhuma no último mês	Nenhuma no último mês	Nenhuma no último mês
Nenhuma no último mês	Menos de 1 vez por semana	Menos de 1 vez por semana
Nenhuma no último mês	Nenhuma no último mês	Nenhuma no último mês
Nenhuma no último mês	Nenhuma no último mês	Nenhuma no último mês

3 ou mais vezes por semana	3 ou mais vezes por semana	Menos de 1 vez por semana
Nenhuma no último mês	Nenhuma no último mês	Nenhuma no último mês
Nenhuma no último mês	Nenhuma no último mês	Nenhuma no último mês
Nenhuma no último mês	Nenhuma no último mês	Nenhuma no último mês
Menos de 1 vez por semana	Nenhuma no último mês	Nenhuma no último mês
Nenhuma no último mês	Nenhuma no último mês	Nenhuma no último mês
1 ou 2 vezes por semana	Nenhuma no último mês	Nenhuma no último mês
Nenhuma no último mês	Nenhuma no último mês	Nenhuma no último mês
Menos de 1 vez por semana	Nenhuma no último mês	Nenhuma no último mês
Nenhuma no último mês	Nenhuma no último mês	Nenhuma no último mês
3 ou mais vezes por semana	1 ou 2 vezes por semana	Nenhuma no último mês

Nenhuma no último mês	Nenhuma no último mês	Menos de 1 vez por semana
Nenhuma no último mês	Nenhuma no último mês	Nenhuma no último mês
Nenhuma no último mês	Nenhuma no último mês	Nenhuma no último mês
Menos de 1 vez por semana	Nenhuma no último mês	Nenhuma no último mês
Nenhuma no último mês	Nenhuma no último mês	Nenhuma no último mês
Nenhuma no último mês	3 ou mais vezes por semana	Menos de 1 vez por semana
Menos de 1 vez por semana	Nenhuma no último mês	Nenhuma no último mês
Nenhuma no último mês	Nenhuma no último mês	Nenhuma no último mês
Nenhuma no último mês	Nenhuma no último mês	Nenhuma no último mês
Nenhuma no último mês	Nenhuma no último mês	Menos de 1 vez por semana
Nenhuma no último mês	Nenhuma no último mês	Nenhuma no último mês

Nenhuma no último mês	Nenhuma no último mês	Nenhuma no último mês
3 ou mais vezes por semana	3 ou mais vezes por semana	Nenhuma no último mês
1 ou 2 vezes por semana	Menos de 1 vez por semana	Menos de 1 vez por semana
1 ou 2 vezes por semana	Nenhuma no último mês	Nenhuma no último mês
Nenhuma no último mês	Nenhuma no último mês	Nenhuma no último mês
Nenhuma no último mês	Nenhuma no último mês	Nenhuma no último mês
Nenhuma no último mês	Nenhuma no último mês	Nenhuma no último mês
Nenhuma no último mês	Nenhuma no último mês	Nenhuma no último mês
Nenhuma no último mês	Nenhuma no último mês	Nenhuma no último mês
Nenhuma no último mês	Nenhuma no último mês	Menos de 1 vez por semana
Nenhuma no último mês	Nenhuma no último mês	Nenhuma no último mês
Nenhuma no último mês	Menos de 1 vez por semana	Nenhuma no último mês

Nenhuma no último mês	Nenhuma no último mês	Nenhuma no último mês
Nenhuma no último mês	Menos de 1 vez por semana	Nenhuma no último mês
Menos de 1 vez por semana	Nenhuma no último mês	Menos de 1 vez por semana
Nenhuma no último mês	Nenhuma no último mês	Nenhuma no último mês
Nenhuma no último mês	Nenhuma no último mês	Nenhuma no último mês
Nenhuma no último mês	Nenhuma no último mês	Nenhuma no último mês
Nenhuma no último mês	Nenhuma no último mês	Nenhuma no último mês
Nenhuma no último mês	Nenhuma no último mês	3 ou mais vezes por semana
Nenhuma no último mês	Menos de 1 vez por semana	Menos de 1 vez por semana
Nenhuma no último mês	Nenhuma no último mês	Nenhuma no último mês
Nenhuma no último mês	Nenhuma no último mês	Nenhuma no último mês

1 ou 2 vezes por semana	Menos de 1 vez por semana	Nenhuma no último mês
3 ou mais vezes por semana	Nenhuma no último mês	1 ou 2 vezes por semana
Nenhuma no último mês	Nenhuma no último mês	Nenhuma no último mês
Nenhuma no último mês	Nenhuma no último mês	Menos de 1 vez por semana
Menos de 1 vez por semana	Nenhuma no último mês	Nenhuma no último mês
Nenhuma no último mês	Nenhuma no último mês	Menos de 1 vez por semana
Nenhuma no último mês	Nenhuma no último mês	Nenhuma no último mês
Menos de 1 vez por semana	Nenhuma no último mês	Nenhuma no último mês
Nenhuma no último mês	Nenhuma no último mês	Nenhuma no último mês
Menos de 1 vez por semana	Nenhuma no último mês	Nenhuma no último mês

Nenhuma no último mês	Nenhuma no último mês	Menos de 1 vez por semana
1 ou 2 vezes por semana	Menos de 1 vez por semana	Nenhuma no último mês
Nenhuma no último mês	Nenhuma no último mês	Nenhuma no último mês
Nenhuma no último mês	Nenhuma no último mês	Nenhuma no último mês
Nenhuma no último mês	Nenhuma no último mês	Nenhuma no último mês
Nenhuma no último mês	Nenhuma no último mês	Nenhuma no último mês
Nenhuma no último mês	Nenhuma no último mês	Nenhuma no último mês
Menos de 1 vez por semana	Nenhuma no último mês	Menos de 1 vez por semana
Nenhuma no último mês	Nenhuma no último mês	Nenhuma no último mês
Nenhuma no último mês	Nenhuma no último mês	Menos de 1 vez por semana
Nenhuma no último mês	Nenhuma no último mês	Nenhuma no último mês

Nenhuma no último mês	Nenhuma no último mês	Nenhuma no último mês
Nenhuma no último mês	Nenhuma no último mês	Nenhuma no último mês
Menos de 1 vez por semana	Nenhuma no último mês	Menos de 1 vez por semana
Nenhuma no último mês	Nenhuma no último mês	Menos de 1 vez por semana
Menos de 1 vez por semana	Menos de 1 vez por semana	Nenhuma no último mês
Nenhuma no último mês	1 ou 2 vezes por semana	Nenhuma no último mês
Menos de 1 vez por semana	Nenhuma no último mês	Nenhuma no último mês
Menos de 1 vez por semana	Nenhuma no último mês	Nenhuma no último mês
Nenhuma no último mês	Nenhuma no último mês	Nenhuma no último mês
Nenhuma no último mês	1 ou 2 vezes por semana	3 ou mais vezes por semana
Menos de 1 vez por semana	Menos de 1 vez por semana	3 ou mais vezes por semana

1 ou 2 vezes por semana	Nenhuma no último mês	Nenhuma no último mês
Nenhuma no último mês	Nenhuma no último mês	Nenhuma no último mês
Nenhuma no último mês	Nenhuma no último mês	Nenhuma no último mês
Nenhuma no último mês	Nenhuma no último mês	Nenhuma no último mês
Nenhuma no último mês	Nenhuma no último mês	Nenhuma no último mês
3 ou mais vezes por semana	3 ou mais vezes por semana	Nenhuma no último mês
Nenhuma no último mês	Nenhuma no último mês	Nenhuma no último mês
1 ou 2 vezes por semana	Nenhuma no último mês	Menos de 1 vez por semana
Menos de 1 vez por semana	Nenhuma no último mês	Nenhuma no último mês
Nenhuma no último mês	Nenhuma no último mês	Nenhuma no último mês

Nenhuma no último mês	Nenhuma no último mês	Nenhuma no último mês
Nenhuma no último mês	Nenhuma no último mês	Menos de 1 vez por semana
Nenhuma no último mês	Nenhuma no último mês	Menos de 1 vez por semana
1 ou 2 vezes por semana	Menos de 1 vez por semana	Nenhuma no último mês
Nenhuma no último mês	Nenhuma no último mês	Nenhuma no último mês
Nenhuma no último mês	Nenhuma no último mês	Nenhuma no último mês
Menos de 1 vez por semana	Menos de 1 vez por semana	Nenhuma no último mês
Nenhuma no último mês	Nenhuma no último mês	Menos de 1 vez por semana
Nenhuma no último mês	Nenhuma no último mês	Nenhuma no último mês
Nenhuma no último mês	Menos de 1 vez por semana	Nenhuma no último mês
Menos de 1 vez por semana	Nenhuma no último mês	1 ou 2 vezes por semana
Menos de 1 vez por semana	Nenhuma no último mês	Nenhuma no último mês

Nenhuma no último mês	Nenhuma no último mês	Nenhuma no último mês
Menos de 1 vez por semana	1 ou 2 vezes por semana	Nenhuma no último mês
Nenhuma no último mês	Nenhuma no último mês	Nenhuma no último mês
Menos de 1 vez por semana	Nenhuma no último mês	Nenhuma no último mês
Menos de 1 vez por semana	Menos de 1 vez por semana	Nenhuma no último mês
Nenhuma no último mês	Nenhuma no último mês	Nenhuma no último mês
Menos de 1 vez por semana	Nenhuma no último mês	Nenhuma no último mês
Nenhuma no último mês	Nenhuma no último mês	Nenhuma no último mês
Nenhuma no último mês	Menos de 1 vez por semana	Nenhuma no último mês
Menos de 1 vez por semana	Nenhuma no último mês	Nenhuma no último mês
Nenhuma no último mês	Nenhuma no último mês	Nenhuma no último mês
Nenhuma no último mês	Nenhuma no último mês	Nenhuma no último mês

Nenhuma no último mês	Nenhuma no último mês	Nenhuma no último mês
Nenhuma no último mês	Nenhuma no último mês	Nenhuma no último mês
Nenhuma no último mês	Nenhuma no último mês	Menos de 1 vez por semana
Nenhuma no último mês	Nenhuma no último mês	Menos de 1 vez por semana
Nenhuma no último mês	Nenhuma no último mês	Nenhuma no último mês
Nenhuma no último mês	Nenhuma no último mês	Menos de 1 vez por semana
Menos de 1 vez por semana	Nenhuma no último mês	Menos de 1 vez por semana
Nenhuma no último mês	Nenhuma no último mês	Nenhuma no último mês
Nenhuma no último mês	Nenhuma no último mês	Menos de 1 vez por semana
Nenhuma no último mês	Nenhuma no último mês	Nenhuma no último mês
Menos de 1 vez por semana	Nenhuma no último mês	1 ou 2 vezes por semana
Nenhuma no último mês	1 ou 2 vezes por semana	Nenhuma no último mês

Nenhuma no último mês	Nenhuma no último mês	Nenhuma no último mês
Nenhuma no último mês	Nenhuma no último mês	Nenhuma no último mês
Nenhuma no último mês	Nenhuma no último mês	Nenhuma no último mês
Menos de 1 vez por semana	Nenhuma no último mês	Menos de 1 vez por semana
Nenhuma no último mês	Nenhuma no último mês	1 ou 2 vezes por semana
Nenhuma no último mês	Nenhuma no último mês	Nenhuma no último mês
Menos de 1 vez por semana	Nenhuma no último mês	Nenhuma no último mês
Nenhuma no último mês	Nenhuma no último mês	Nenhuma no último mês
Nenhuma no último mês	Nenhuma no último mês	Nenhuma no último mês
1 ou 2 vezes por semana	3 ou mais vezes por semana	Nenhuma no último mês
Nenhuma no último mês	Nenhuma no último mês	Nenhuma no último mês
Nenhuma no último mês	Nenhuma no último mês	Menos de 1 vez por semana

Nenhuma no último mês	Nenhuma no último mês	Nenhuma no último mês
Nenhuma no último mês	Nenhuma no último mês	Nenhuma no último mês
1 ou 2 vezes por semana	Menos de 1 vez por semana	Nenhuma no último mês
Nenhuma no último mês	Nenhuma no último mês	Nenhuma no último mês
Menos de 1 vez por semana	Nenhuma no último mês	Nenhuma no último mês
Nenhuma no último mês	Menos de 1 vez por semana	Nenhuma no último mês
Nenhuma no último mês	Nenhuma no último mês	Nenhuma no último mês
Nenhuma no último mês	Nenhuma no último mês	Nenhuma no último mês
Nenhuma no último mês	Menos de 1 vez por semana	Nenhuma no último mês
Nenhuma no último mês	Nenhuma no último mês	Nenhuma no último mês
Menos de 1 vez por semana	Nenhuma no último mês	Nenhuma no último mês
Nenhuma no último mês	Nenhuma no último mês	Nenhuma no último mês

Nenhuma no último mês	Nenhuma no último mês	Nenhuma no último mês
Nenhuma no último mês	Nenhuma no último mês	Nenhuma no último mês
Nenhuma no último mês	Nenhuma no último mês	Menos de 1 vez por semana
Nenhuma no último mês	Nenhuma no último mês	Nenhuma no último mês
Nenhuma no último mês	Nenhuma no último mês	Menos de 1 vez por semana

Questi[illegible]		
Durante o último mês, com que frequência você teve dificuldade de dormir porque sentiu muito calor?	Durante o último mês, com que frequência você teve dificuldade de dormir porque teve sonhos ruins?	Durante o último mês, com que frequência você teve dificuldade de dormir porque teve dor?
Menos de 1 vez por semana	Menos de 1 vez por semana	Menos de 1 vez por semana
Nenhuma no último mês	Nenhuma no último mês	Nenhuma no último mês
Nenhuma no último mês	Menos de 1 vez por semana	Nenhuma no último mês
3 ou mais vezes por semana	1 ou 2 vezes por semana	Nenhuma no último mês
Menos de 1 vez por semana	Menos de 1 vez por semana	Nenhuma no último mês
Menos de 1 vez por semana	1 ou 2 vezes por semana	Menos de 1 vez por semana
1 ou 2 vezes por semana	Nenhuma no último mês	Nenhuma no último mês
Menos de 1 vez por semana	Menos de 1 vez por semana	Nenhuma no último mês
Menos de 1 vez por semana	Nenhuma no último mês	Menos de 1 vez por semana

1 ou 2 vezes por semana	1 ou 2 vezes por semana	Menos de 1 vez por semana
Nenhuma no último mês	Nenhuma no último mês	Nenhuma no último mês
Nenhuma no último mês	Menos de 1 vez por semana	Nenhuma no último mês
Menos de 1 vez por semana	Menos de 1 vez por semana	Menos de 1 vez por semana
1 ou 2 vezes por semana	Nenhuma no último mês	Menos de 1 vez por semana
Menos de 1 vez por semana	Menos de 1 vez por semana	Menos de 1 vez por semana
Menos de 1 vez por semana	Nenhuma no último mês	Nenhuma no último mês
Menos de 1 vez por semana	1 ou 2 vezes por semana	Nenhuma no último mês
Menos de 1 vez por semana	Nenhuma no último mês	Menos de 1 vez por semana
Nenhuma no último mês	1 ou 2 vezes por semana	1 ou 2 vezes por semana
Nenhuma no último mês	Nenhuma no último mês	Nenhuma no último mês

Nenhuma no último mês	Nenhuma no último mês	Nenhuma no último mês
Nenhuma no último mês	1 ou 2 vezes por semana	Nenhuma no último mês
Menos de 1 vez por semana	Menos de 1 vez por semana	Menos de 1 vez por semana
Nenhuma no último mês	Nenhuma no último mês	Menos de 1 vez por semana
Nenhuma no último mês	3 ou mais vezes por semana	3 ou mais vezes por semana
Menos de 1 vez por semana	Nenhuma no último mês	Nenhuma no último mês
1 ou 2 vezes por semana	1 ou 2 vezes por semana	Menos de 1 vez por semana
1 ou 2 vezes por semana	1 ou 2 vezes por semana	Nenhuma no último mês
1 ou 2 vezes por semana	Nenhuma no último mês	Nenhuma no último mês
Nenhuma no último mês	Nenhuma no último mês	1 ou 2 vezes por semana

Menos de 1 vez por semana	Menos de 1 vez por semana	Nenhuma no último mês
Nenhuma no último mês	Nenhuma no último mês	Nenhuma no último mês
Nenhuma no último mês	Menos de 1 vez por semana	Nenhuma no último mês
1 ou 2 vezes por semana	Nenhuma no último mês	Nenhuma no último mês
Nenhuma no último mês	Nenhuma no último mês	Nenhuma no último mês
Nenhuma no último mês	Menos de 1 vez por semana	Nenhuma no último mês
Menos de 1 vez por semana	Menos de 1 vez por semana	1 ou 2 vezes por semana
Nenhuma no último mês	Nenhuma no último mês	Nenhuma no último mês
Nenhuma no último mês	Menos de 1 vez por semana	Nenhuma no último mês
1 ou 2 vezes por semana	Nenhuma no último mês	Menos de 1 vez por semana
Menos de 1 vez por semana	Nenhuma no último mês	Nenhuma no último mês
Nenhuma no último mês	Nenhuma no último mês	Nenhuma no último mês

Menos de 1 vez por semana	Menos de 1 vez por semana	Menos de 1 vez por semana
1 ou 2 vezes por semana	1 ou 2 vezes por semana	1 ou 2 vezes por semana
1 ou 2 vezes por semana	Menos de 1 vez por semana	1 ou 2 vezes por semana
Menos de 1 vez por semana	Nenhuma no último mês	1 ou 2 vezes por semana
Nenhuma no último mês	Nenhuma no último mês	3 ou mais vezes por semana
Menos de 1 vez por semana	Nenhuma no último mês	Menos de 1 vez por semana
Nenhuma no último mês	Nenhuma no último mês	Nenhuma no último mês
Nenhuma no último mês	Nenhuma no último mês	Menos de 1 vez por semana
Nenhuma no último mês	Nenhuma no último mês	Nenhuma no último mês
Menos de 1 vez por semana	Menos de 1 vez por semana	Nenhuma no último mês

Nenhuma no último mês	1 ou 2 vezes por semana	1 ou 2 vezes por semana
Nenhuma no último mês	Menos de 1 vez por semana	1 ou 2 vezes por semana
Menos de 1 vez por semana	Nenhuma no último mês	Menos de 1 vez por semana
1 ou 2 vezes por semana	3 ou mais vezes por semana	1 ou 2 vezes por semana
Nenhuma no último mês	Menos de 1 vez por semana	Menos de 1 vez por semana
Nenhuma no último mês	Menos de 1 vez por semana	Menos de 1 vez por semana
Nenhuma no último mês	Nenhuma no último mês	Nenhuma no último mês
Menos de 1 vez por semana	Nenhuma no último mês	Menos de 1 vez por semana
Menos de 1 vez por semana	Nenhuma no último mês	Nenhuma no último mês
Menos de 1 vez por semana	Nenhuma no último mês	1 ou 2 vezes por semana
Nenhuma no último mês	1 ou 2 vezes por semana	Nenhuma no último mês

Menos de 1 vez por semana	Menos de 1 vez por semana	Menos de 1 vez por semana
Nenhuma no último mês	Nenhuma no último mês	Menos de 1 vez por semana
Nenhuma no último mês	Menos de 1 vez por semana	Nenhuma no último mês
Menos de 1 vez por semana	Menos de 1 vez por semana	Menos de 1 vez por semana
Nenhuma no último mês	Nenhuma no último mês	Nenhuma no último mês
Nenhuma no último mês	Menos de 1 vez por semana	Menos de 1 vez por semana
Nenhuma no último mês	Nenhuma no último mês	Nenhuma no último mês
Nenhuma no último mês	Nenhuma no último mês	Nenhuma no último mês
Menos de 1 vez por semana	Nenhuma no último mês	Nenhuma no último mês
3 ou mais vezes por semana	Menos de 1 vez por semana	Nenhuma no último mês
3 ou mais vezes por semana	Nenhuma no último mês	Menos de 1 vez por semana

Menos de 1 vez por semana	Menos de 1 vez por semana	Nenhuma no último mês
Nenhuma no último mês	Menos de 1 vez por semana	Nenhuma no último mês
Nenhuma no último mês	Nenhuma no último mês	Menos de 1 vez por semana
1 ou 2 vezes por semana	Nenhuma no último mês	Nenhuma no último mês
Menos de 1 vez por semana	Nenhuma no último mês	Nenhuma no último mês
Menos de 1 vez por semana	3 ou mais vezes por semana	1 ou 2 vezes por semana
Menos de 1 vez por semana	Nenhuma no último mês	1 ou 2 vezes por semana
Nenhuma no último mês	Nenhuma no último mês	Nenhuma no último mês
Menos de 1 vez por semana	Menos de 1 vez por semana	Nenhuma no último mês
Nenhuma no último mês	Menos de 1 vez por semana	Nenhuma no último mês
Nenhuma no último mês	Nenhuma no último mês	Nenhuma no último mês
Nenhuma no último mês	1 ou 2 vezes por semana	Nenhuma no último mês

Nenhuma no último mês	Nenhuma no último mês	Nenhuma no último mês
1 ou 2 vezes por semana	Menos de 1 vez por semana	Nenhuma no último mês
1 ou 2 vezes por semana	Menos de 1 vez por semana	Menos de 1 vez por semana
1 ou 2 vezes por semana	Menos de 1 vez por semana	Nenhuma no último mês
Nenhuma no último mês	Nenhuma no último mês	Nenhuma no último mês
3 ou mais vezes por semana	Menos de 1 vez por semana	Menos de 1 vez por semana
3 ou mais vezes por semana	1 ou 2 vezes por semana	Menos de 1 vez por semana
1 ou 2 vezes por semana	3 ou mais vezes por semana	1 ou 2 vezes por semana
Nenhuma no último mês	Nenhuma no último mês	Nenhuma no último mês
Menos de 1 vez por semana	1 ou 2 vezes por semana	Menos de 1 vez por semana
Menos de 1 vez por semana	1 ou 2 vezes por semana	3 ou mais vezes por semana
1 ou 2 vezes por semana	Nenhuma no último mês	1 ou 2 vezes por semana

Nenhuma no último mês	3 ou mais vezes por semana	Nenhuma no último mês
Menos de 1 vez por semana	Nenhuma no último mês	Nenhuma no último mês
Menos de 1 vez por semana	Menos de 1 vez por semana	1 ou 2 vezes por semana
Menos de 1 vez por semana	Menos de 1 vez por semana	Nenhuma no último mês
Menos de 1 vez por semana	Nenhuma no último mês	Nenhuma no último mês
Nenhuma no último mês	1 ou 2 vezes por semana	Nenhuma no último mês
Nenhuma no último mês	Nenhuma no último mês	Nenhuma no último mês
Menos de 1 vez por semana	Menos de 1 vez por semana	Nenhuma no último mês
Nenhuma no último mês	3 ou mais vezes por semana	Menos de 1 vez por semana
1 ou 2 vezes por semana	3 ou mais vezes por semana	Menos de 1 vez por semana
Nenhuma no último mês	Menos de 1 vez por semana	Nenhuma no último mês

1 ou 2 vezes por semana	3 ou mais vezes por semana	Nenhuma no último mês
Nenhuma no último mês	Nenhuma no último mês	Nenhuma no último mês
1 ou 2 vezes por semana	Nenhuma no último mês	1 ou 2 vezes por semana
Nenhuma no último mês	Nenhuma no último mês	Nenhuma no último mês
3 ou mais vezes por semana	Menos de 1 vez por semana	Nenhuma no último mês
1 ou 2 vezes por semana	Menos de 1 vez por semana	Menos de 1 vez por semana
1 ou 2 vezes por semana	Nenhuma no último mês	Nenhuma no último mês
Menos de 1 vez por semana	1 ou 2 vezes por semana	Nenhuma no último mês
Nenhuma no último mês	Nenhuma no último mês	Nenhuma no último mês
Nenhuma no último mês	Nenhuma no último mês	Nenhuma no último mês

Menos de 1 vez por semana	Nenhuma no último mês	Nenhuma no último mês
Nenhuma no último mês	1 ou 2 vezes por semana	Menos de 1 vez por semana
Nenhuma no último mês	Menos de 1 vez por semana	Nenhuma no último mês
Menos de 1 vez por semana	Nenhuma no último mês	Nenhuma no último mês
Nenhuma no último mês	Nenhuma no último mês	Menos de 1 vez por semana
Nenhuma no último mês	Nenhuma no último mês	Nenhuma no último mês
1 ou 2 vezes por semana	Menos de 1 vez por semana	Menos de 1 vez por semana
Nenhuma no último mês	Nenhuma no último mês	Nenhuma no último mês
Menos de 1 vez por semana	Nenhuma no último mês	Nenhuma no último mês
Nenhuma no último mês	Nenhuma no último mês	Nenhuma no último mês
Nenhuma no último mês	Nenhuma no último mês	Nenhuma no último mês

Menos de 1 vez por semana	Nenhuma no último mês	1 ou 2 vezes por semana
1 ou 2 vezes por semana	Nenhuma no último mês	Menos de 1 vez por semana
Nenhuma no último mês	3 ou mais vezes por semana	Menos de 1 vez por semana
1 ou 2 vezes por semana	Nenhuma no último mês	Nenhuma no último mês
Nenhuma no último mês	Menos de 1 vez por semana	Nenhuma no último mês
Nenhuma no último mês	Nenhuma no último mês	1 ou 2 vezes por semana
Nenhuma no último mês	Nenhuma no último mês	Nenhuma no último mês
1 ou 2 vezes por semana	Menos de 1 vez por semana	1 ou 2 vezes por semana
3 ou mais vezes por semana	1 ou 2 vezes por semana	1 ou 2 vezes por semana
Menos de 1 vez por semana	Nenhuma no último mês	3 ou mais vezes por semana
3 ou mais vezes por semana	Nenhuma no último mês	Nenhuma no último mês

Nenhuma no último mês	Nenhuma no último mês	Nenhuma no último mês
Nenhuma no último mês	Nenhuma no último mês	Nenhuma no último mês
1 ou 2 vezes por semana	Menos de 1 vez por semana	1 ou 2 vezes por semana
Menos de 1 vez por semana	Menos de 1 vez por semana	Menos de 1 vez por semana
Nenhuma no último mês	Nenhuma no último mês	Nenhuma no último mês
Nenhuma no último mês	Nenhuma no último mês	Menos de 1 vez por semana
Nenhuma no último mês	Nenhuma no último mês	Nenhuma no último mês
Nenhuma no último mês	Nenhuma no último mês	Nenhuma no último mês
Nenhuma no último mês	Nenhuma no último mês	Nenhuma no último mês
Nenhuma no último mês	1 ou 2 vezes por semana	Menos de 1 vez por semana
Nenhuma no último mês	Nenhuma no último mês	Nenhuma no último mês
Nenhuma no último mês	Menos de 1 vez por semana	Nenhuma no último mês

Nenhuma no último mês	Nenhuma no último mês	Nenhuma no último mês
Nenhuma no último mês	1 ou 2 vezes por semana	Menos de 1 vez por semana
Nenhuma no último mês	Menos de 1 vez por semana	Nenhuma no último mês
Nenhuma no último mês	Nenhuma no último mês	Nenhuma no último mês
Nenhuma no último mês	Menos de 1 vez por semana	Nenhuma no último mês
Menos de 1 vez por semana	Nenhuma no último mês	Menos de 1 vez por semana
3 ou mais vezes por semana	Nenhuma no último mês	Nenhuma no último mês
Menos de 1 vez por semana	Nenhuma no último mês	1 ou 2 vezes por semana
Menos de 1 vez por semana	Menos de 1 vez por semana	Menos de 1 vez por semana
Menos de 1 vez por semana	Nenhuma no último mês	Menos de 1 vez por semana
Nenhuma no último mês	Nenhuma no último mês	Menos de 1 vez por semana
Nenhuma no último mês	Nenhuma no último mês	Nenhuma no último mês

Menos de 1 vez por semana	Nenhuma no último mês	Nenhuma no último mês
Nenhuma no último mês	Nenhuma no último mês	Nenhuma no último mês
Menos de 1 vez por semana	1 ou 2 vezes por semana	Menos de 1 vez por semana
3 ou mais vezes por semana	1 ou 2 vezes por semana	Menos de 1 vez por semana
Nenhuma no último mês	Menos de 1 vez por semana	Nenhuma no último mês
Menos de 1 vez por semana	Nenhuma no último mês	Nenhuma no último mês
Menos de 1 vez por semana	Nenhuma no último mês	Menos de 1 vez por semana
Menos de 1 vez por semana	Menos de 1 vez por semana	Nenhuma no último mês
1 ou 2 vezes por semana	1 ou 2 vezes por semana	Menos de 1 vez por semana
Menos de 1 vez por semana	Nenhuma no último mês	Nenhuma no último mês
Nenhuma no último mês	Nenhuma no último mês	Nenhuma no último mês

Nenhuma no último mês	Nenhuma no último mês	1 ou 2 vezes por semana
Nenhuma no último mês	Nenhuma no último mês	Nenhuma no último mês
Menos de 1 vez por semana	Menos de 1 vez por semana	Nenhuma no último mês
Nenhuma no último mês	Nenhuma no último mês	1 ou 2 vezes por semana
Nenhuma no último mês	1 ou 2 vezes por semana	1 ou 2 vezes por semana
Nenhuma no último mês	3 ou mais vezes por semana	1 ou 2 vezes por semana
Nenhuma no último mês	Nenhuma no último mês	Nenhuma no último mês
Menos de 1 vez por semana	Menos de 1 vez por semana	Nenhuma no último mês
Nenhuma no último mês	Nenhuma no último mês	Nenhuma no último mês
Nenhuma no último mês	Menos de 1 vez por semana	Nenhuma no último mês
Nenhuma no último mês	Menos de 1 vez por semana	1 ou 2 vezes por semana

Menos de 1 vez por semana	Nenhuma no último mês	1 ou 2 vezes por semana
Nenhuma no último mês	Menos de 1 vez por semana	Nenhuma no último mês
Nenhuma no último mês	1 ou 2 vezes por semana	1 ou 2 vezes por semana
1 ou 2 vezes por semana	Nenhuma no último mês	Nenhuma no último mês
Nenhuma no último mês	Nenhuma no último mês	Menos de 1 vez por semana
Nenhuma no último mês	Nenhuma no último mês	Menos de 1 vez por semana
Menos de 1 vez por semana	3 ou mais vezes por semana	Nenhuma no último mês
Nenhuma no último mês	Nenhuma no último mês	Nenhuma no último mês
Nenhuma no último mês	Menos de 1 vez por semana	Nenhuma no último mês
1 ou 2 vezes por semana	Menos de 1 vez por semana	Menos de 1 vez por semana
Nenhuma no último mês	1 ou 2 vezes por semana	Menos de 1 vez por semana

Nenhuma no último mês	Nenhuma no último mês	Nenhuma no último mês
3 ou mais vezes por semana	Menos de 1 vez por semana	1 ou 2 vezes por semana
1 ou 2 vezes por semana	Nenhuma no último mês	3 ou mais vezes por semana
Nenhuma no último mês	Menos de 1 vez por semana	Menos de 1 vez por semana
Menos de 1 vez por semana	Nenhuma no último mês	Nenhuma no último mês
1 ou 2 vezes por semana	3 ou mais vezes por semana	Menos de 1 vez por semana
Menos de 1 vez por semana	Nenhuma no último mês	Nenhuma no último mês
Nenhuma no último mês	Nenhuma no último mês	1 ou 2 vezes por semana
Menos de 1 vez por semana	Nenhuma no último mês	Menos de 1 vez por semana
Nenhuma no último mês	Menos de 1 vez por semana	3 ou mais vezes por semana
Menos de 1 vez por semana	Nenhuma no último mês	Nenhuma no último mês
Menos de 1 vez por semana	1 ou 2 vezes por semana	1 ou 2 vezes por semana

Nenhuma no último mês	1 ou 2 vezes por semana	Nenhuma no último mês
1 ou 2 vezes por semana	Nenhuma no último mês	Nenhuma no último mês
Nenhuma no último mês	Menos de 1 vez por semana	Menos de 1 vez por semana
Menos de 1 vez por semana	Nenhuma no último mês	Nenhuma no último mês
Menos de 1 vez por semana	Menos de 1 vez por semana	Nenhuma no último mês
Nenhuma no último mês	Nenhuma no último mês	Nenhuma no último mês
Nenhuma no último mês	3 ou mais vezes por semana	1 ou 2 vezes por semana
Menos de 1 vez por semana	Menos de 1 vez por semana	Menos de 1 vez por semana
Menos de 1 vez por semana	Menos de 1 vez por semana	Nenhuma no último mês
Nenhuma no último mês	Nenhuma no último mês	Nenhuma no último mês
Nenhuma no último mês	Nenhuma no último mês	Menos de 1 vez por semana

Menos de 1 vez por semana	Nenhuma no último mês	1 ou 2 vezes por semana
Nenhuma no último mês	Nenhuma no último mês	1 ou 2 vezes por semana
1 ou 2 vezes por semana	3 ou mais vezes por semana	3 ou mais vezes por semana
1 ou 2 vezes por semana	Nenhuma no último mês	1 ou 2 vezes por semana
Nenhuma no último mês	Menos de 1 vez por semana	Nenhuma no último mês
Nenhuma no último mês	Menos de 1 vez por semana	Nenhuma no último mês
1 ou 2 vezes por semana	Nenhuma no último mês	Nenhuma no último mês
Nenhuma no último mês	Menos de 1 vez por semana	Menos de 1 vez por semana
3 ou mais vezes por semana	3 ou mais vezes por semana	1 ou 2 vezes por semana
Menos de 1 vez por semana	Nenhuma no último mês	Menos de 1 vez por semana

1 ou 2 vezes por semana	Menos de 1 vez por semana	1 ou 2 vezes por semana
1 ou 2 vezes por semana	Nenhuma no último mês	Menos de 1 vez por semana
Nenhuma no último mês	Menos de 1 vez por semana	1 ou 2 vezes por semana
Nenhuma no último mês	Nenhuma no último mês	3 ou mais vezes por semana
Nenhuma no último mês	Nenhuma no último mês	1 ou 2 vezes por semana
Nenhuma no último mês	Menos de 1 vez por semana	Nenhuma no último mês
Nenhuma no último mês	Nenhuma no último mês	Menos de 1 vez por semana
Nenhuma no último mês	Nenhuma no último mês	Nenhuma no último mês
Menos de 1 vez por semana	Nenhuma no último mês	Menos de 1 vez por semana
Menos de 1 vez por semana	1 ou 2 vezes por semana	3 ou mais vezes por semana
Nenhuma no último mês	Menos de 1 vez por semana	Menos de 1 vez por semana

Nenhuma no último mês	Nenhuma no último mês	Nenhuma no último mês
1 ou 2 vezes por semana	Menos de 1 vez por semana	Menos de 1 vez por semana
Menos de 1 vez por semana	Menos de 1 vez por semana	Menos de 1 vez por semana
3 ou mais vezes por semana	Nenhuma no último mês	Menos de 1 vez por semana
Menos de 1 vez por semana	Menos de 1 vez por semana	Menos de 1 vez por semana
Nenhuma no último mês	Nenhuma no último mês	Nenhuma no último mês
Menos de 1 vez por semana	Menos de 1 vez por semana	Menos de 1 vez por semana
Menos de 1 vez por semana	Menos de 1 vez por semana	1 ou 2 vezes por semana
Nenhuma no último mês	Nenhuma no último mês	Nenhuma no último mês
1 ou 2 vezes por semana	Nenhuma no último mês	Nenhuma no último mês
Menos de 1 vez por semana	3 ou mais vezes por semana	1 ou 2 vezes por semana

1 ou 2 vezes por semana	Menos de 1 vez por semana	Menos de 1 vez por semana
Menos de 1 vez por semana	Menos de 1 vez por semana	Nenhuma no último mês
1 ou 2 vezes por semana	Menos de 1 vez por semana	Nenhuma no último mês
Nenhuma no último mês	Menos de 1 vez por semana	Nenhuma no último mês
Nenhuma no último mês	Menos de 1 vez por semana	3 ou mais vezes por semana
Nenhuma no último mês	Nenhuma no último mês	Menos de 1 vez por semana
3 ou mais vezes por semana	Nenhuma no último mês	Nenhuma no último mês
1 ou 2 vezes por semana	1 ou 2 vezes por semana	Nenhuma no último mês
Nenhuma no último mês	Menos de 1 vez por semana	3 ou mais vezes por semana
Menos de 1 vez por semana	Menos de 1 vez por semana	3 ou mais vezes por semana

Nenhuma no último mês	Nenhuma no último mês	Nenhuma no último mês
Nenhuma no último mês	Nenhuma no último mês	Menos de 1 vez por semana
Nenhuma no último mês	Menos de 1 vez por semana	Menos de 1 vez por semana
3 ou mais vezes por semana	Nenhuma no último mês	3 ou mais vezes por semana
1 ou 2 vezes por semana	Menos de 1 vez por semana	1 ou 2 vezes por semana
Nenhuma no último mês	Nenhuma no último mês	Nenhuma no último mês
Menos de 1 vez por semana	Nenhuma no último mês	Nenhuma no último mês
Menos de 1 vez por semana	Nenhuma no último mês	Nenhuma no último mês
Menos de 1 vez por semana	Menos de 1 vez por semana	Nenhuma no último mês
Nenhuma no último mês	Nenhuma no último mês	Nenhuma no último mês
1 ou 2 vezes por semana	Menos de 1 vez por semana	Menos de 1 vez por semana
Menos de 1 vez por semana	Menos de 1 vez por semana	1 ou 2 vezes por semana

Nenhuma no último mês	Nenhuma no último mês	3 ou mais vezes por semana
1 ou 2 vezes por semana	Menos de 1 vez por semana	1 ou 2 vezes por semana
Nenhuma no último mês	Nenhuma no último mês	Nenhuma no último mês
Nenhuma no último mês	Menos de 1 vez por semana	Nenhuma no último mês
Nenhuma no último mês	Menos de 1 vez por semana	Nenhuma no último mês
Nenhuma no último mês	Nenhuma no último mês	3 ou mais vezes por semana
3 ou mais vezes por semana	Nenhuma no último mês	3 ou mais vezes por semana
Nenhuma no último mês	Nenhuma no último mês	Nenhuma no último mês
Nenhuma no último mês	Menos de 1 vez por semana	Nenhuma no último mês
Nenhuma no último mês	Nenhuma no último mês	Nenhuma no último mês
Nenhuma no último mês	Nenhuma no último mês	Nenhuma no último mês
Menos de 1 vez por semana	Nenhuma no último mês	Nenhuma no último mês

Nenhuma no último mês	Nenhuma no último mês	Nenhuma no último mês
Menos de 1 vez por semana	Nenhuma no último mês	Nenhuma no último mês
Nenhuma no último mês	3 ou mais vezes por semana	Menos de 1 vez por semana
Nenhuma no último mês	Menos de 1 vez por semana	Nenhuma no último mês
1 ou 2 vezes por semana	Nenhuma no último mês	Nenhuma no último mês
1 ou 2 vezes por semana	Menos de 1 vez por semana	3 ou mais vezes por semana
3 ou mais vezes por semana	1 ou 2 vezes por semana	Menos de 1 vez por semana
Nenhuma no último mês	Nenhuma no último mês	Nenhuma no último mês
Nenhuma no último mês	Nenhuma no último mês	Nenhuma no último mês
Nenhuma no último mês	Menos de 1 vez por semana	Menos de 1 vez por semana
Nenhuma no último mês	Nenhuma no último mês	Menos de 1 vez por semana
1 ou 2 vezes por semana	Nenhuma no último mês	Menos de 1 vez por semana

Nenhuma no último mês	Nenhuma no último mês	Menos de 1 vez por semana
Nenhuma no último mês	1 ou 2 vezes por semana	Nenhuma no último mês
Menos de 1 vez por semana	3 ou mais vezes por semana	Nenhuma no último mês
Menos de 1 vez por semana	Menos de 1 vez por semana	1 ou 2 vezes por semana
1 ou 2 vezes por semana	Nenhuma no último mês	Menos de 1 vez por semana
Nenhuma no último mês	Nenhuma no último mês	Nenhuma no último mês
3 ou mais vezes por semana	Menos de 1 vez por semana	Menos de 1 vez por semana
Nenhuma no último mês	1 ou 2 vezes por semana	3 ou mais vezes por semana
1 ou 2 vezes por semana	Menos de 1 vez por semana	1 ou 2 vezes por semana
Nenhuma no último mês	1 ou 2 vezes por semana	1 ou 2 vezes por semana
Nenhuma no último mês	Menos de 1 vez por semana	1 ou 2 vezes por semana
Nenhuma no último mês	Menos de 1 vez por semana	3 ou mais vezes por semana

Nenhuma no último mês	Nenhuma no último mês	Nenhuma no último mês
1 ou 2 vezes por semana	Menos de 1 vez por semana	Nenhuma no último mês
Nenhuma no último mês	Nenhuma no último mês	Nenhuma no último mês
Nenhuma no último mês	Nenhuma no último mês	Nenhuma no último mês
Menos de 1 vez por semana	Nenhuma no último mês	Nenhuma no último mês
Menos de 1 vez por semana	Nenhuma no último mês	Menos de 1 vez por semana
Nenhuma no último mês	Nenhuma no último mês	Nenhuma no último mês
3 ou mais vezes por semana	1 ou 2 vezes por semana	Nenhuma no último mês
Nenhuma no último mês	Nenhuma no último mês	Nenhuma no último mês
Nenhuma no último mês	Nenhuma no último mês	Nenhuma no último mês
1 ou 2 vezes por semana	Menos de 1 vez por semana	Nenhuma no último mês
Nenhuma no último mês	Nenhuma no último mês	Nenhuma no último mês

3 ou mais vezes por semana	Nenhuma no último mês	Nenhuma no último mês
1 ou 2 vezes por semana	3 ou mais vezes por semana	Nenhuma no último mês
Menos de 1 vez por semana	1 ou 2 vezes por semana	Menos de 1 vez por semana
Menos de 1 vez por semana	Nenhuma no último mês	1 ou 2 vezes por semana
Nenhuma no último mês	Menos de 1 vez por semana	Menos de 1 vez por semana

onário Índice de Qualidade do Sono de Pit

Outra(s) razão(ões), por favor descreva?	Durante o último mês, com que frequência você teve dificuldade para dormir devido a essa razão?	Durante o último mês, como você classificaria a qualidade do seu sono de maneira geral?
	Nenhuma no último mês	Boa
	Nenhuma no último mês	Ruim
Ansiedade e agitação mais de 3 vezes por semana	3 ou mais vezes por semana	Muito boa
	Nenhuma no último mês	Muito Ruim
	Nenhuma no último mês	Boa
	Nenhuma no último mês	Boa
Agitação	3 ou mais vezes por semana	Boa
	Nenhuma no último mês	Boa
	Nenhuma no último mês	Boa

	Nenhuma no último mês	Muito Ruim
	Nenhuma no último mês	Muito Ruim
	Nenhuma no último mês	Boa
Nenhuma	Nenhuma no último mês	Boa
	Nenhuma no último mês	Boa
	Nenhuma no último mês	Ruim
	Nenhuma no último mês	Ruim
	Nenhuma no último mês	Boa
	Nenhuma no último mês	Boa
Ansiedade	3 ou mais vezes por semana	Boa
	Nenhuma no último mês	Muito boa

	Nenhuma no último mês	Boa
	Nenhuma no último mês	Boa
Não	Nenhuma no último mês	Boa
	Nenhuma no último mês	Boa
Tenho ansiedade, terror absoluto do escuro, meu sono é muito leve e alerta, qualquer barulho me acorda. E nesse último mês tive dengue.	3 ou mais vezes por semana	Muito Ruim
	Nenhuma no último mês	Boa
	Nenhuma no último mês	Ruim
Ansiedade.	3 ou mais vezes por semana	Ruim
	Nenhuma no último mês	Boa
	Nenhuma no último mês	Boa

	Nenhuma no último mês	Ruim
	Nenhuma no último mês	Boa
Maioria das vezes por estar no celular pouco antes de adormecer ou com crise depressiva	Menos de 1 vez por semana	Boa
	Nenhuma no último mês	Ruim
	Nenhuma no último mês	Boa
	Nenhuma no último mês	Muito boa
	Nenhuma no último mês	Ruim
	Nenhuma no último mês	Ruim
Ansiedade	3 ou mais vezes por semana	Boa
	Nenhuma no último mês	Ruim
	Nenhuma no último mês	Boa
	Nenhuma no último mês	Boa

Sofri um acidente de ônibus e dormi mal Poe sonhar com ele	1 ou 2 vezes por semana	Boa
	Nenhuma no último mês	Ruim
	Nenhuma no último mês	Boa
	Nenhuma no último mês	Ruim
	Nenhuma no último mês	Ruim
	Nenhuma no último mês	Ruim
Tenho dificuldade para dormir porque minha cama é pequena e divido com a minha cachorra que mesmo tendo sua cama, ela invade a minha por medo de sons externos.	3 ou mais vezes por semana	Boa
	Nenhuma no último mês	Boa
Fome	1 ou 2 vezes por semana	Boa
	Nenhuma no último mês	Boa

	Nenhuma no último mês	Ruim
	Nenhuma no último mês	Boa
	Nenhuma no último mês	Boa
.	Nenhuma no último mês	Ruim
Preocupações com cenário atual da minha profissão	3 ou mais vezes por semana	Ruim
Não	Nenhuma no último mês	Ruim
	Nenhuma no último mês	Boa
	Nenhuma no último mês	Boa
	Nenhuma no último mês	Boa
	Nenhuma no último mês	Ruim
	Nenhuma no último mês	Muito boa

Não consegui descansar a mente para adormecer.	3 ou mais vezes por semana	Boa
	Nenhuma no último mês	Muito boa
	Nenhuma no último mês	Muito boa
Sinto que eu durmo mal por que eu tenho muita dificuldade na hora de acordar, então geralmente a partir das 7 da manhã eu já estou com um sono bem ruim	3 ou mais vezes por semana	Ruim
Preocupação com trabalho, stress	1 ou 2 vezes por semana	Boa
	Nenhuma no último mês	Boa
Não tive nenhuma razão para atrapalhar meu sono.	Nenhuma no último mês	Muito boa
	Nenhuma no último mês	Boa
	Nenhuma no último mês	Boa
	Nenhuma no último mês	Muito boa
	Nenhuma no último mês	Ruim

Ansiedade, preocupação	1 ou 2 vezes por semana	Boa
Ansiedade	1 ou 2 vezes por semana	Ruim
	Nenhuma no último mês	Boa
Barulhos na casa, geralmente próximos a porta do meu quarto me acordam antes da hora.	3 ou mais vezes por semana	Boa
	Nenhuma no último mês	Boa
Ver muitas noticias durante o dia	1 ou 2 vezes por semana	Ruim
	Nenhuma no último mês	Ruim
	Nenhuma no último mês	Muito boa
	Nenhuma no último mês	Boa
	Nenhuma no último mês	Boa
	Nenhuma no último mês	Boa
	Nenhuma no último mês	Ruim

	Nenhuma no último mês	Muito boa
	Nenhuma no último mês	Boa
	Nenhuma no último mês	Boa
	Nenhuma no último mês	Boa
	Nenhuma no último mês	Boa
	Nenhuma no último mês	Ruim
	Nenhuma no último mês	Boa
	Nenhuma no último mês	Muito Ruim
	Nenhuma no último mês	Boa
	Nenhuma no último mês	Ruim
	Nenhuma no último mês	Boa
	Nenhuma no último mês	Ruim

	Nenhuma no último mês	Muito boa
	Nenhuma no último mês	Ruim
	Nenhuma no último mês	Boa
	Nenhuma no último mês	Boa
	Nenhuma no último mês	Boa
	Nenhuma no último mês	Boa
	Nenhuma no último mês	Boa
	Nenhuma no último mês	Boa
	Menos de 1 vez por semana	Boa
Dificuldade em dormir por ansiedade	3 ou mais vezes por semana	Boa
Ansiedade na hora que deito	1 ou 2 vezes por semana	Ruim

	Nenhuma no último mês	Ruim
Stress	3 ou mais vezes por semana	Muito Ruim
	Nenhuma no último mês	Ruim
	Nenhuma no último mês	Boa
	Nenhuma no último mês	Ruim
	Nenhuma no último mês	Boa
Tempo seco e calor com ventilador ligado. Colega de casa que acorda mais cedo e fica fazendo barulho. Adoção de 2 filhotes de gatos que estão energéticos de madrugada :p	1 ou 2 vezes por semana	Ruim
	Nenhuma no último mês	Boa
	Nenhuma no último mês	Boa
	Nenhuma no último mês	Boa

	Nenhuma no último mês	Boa
	Nenhuma no último mês	Ruim
Nao	Nenhuma no último mês	Boa
	Nenhuma no último mês	Boa
	Nenhuma no último mês	Ruim
	Nenhuma no último mês	Boa
Ansiedade /preocupação	3 ou mais vezes por semana	Ruim
	Nenhuma no último mês	Boa
Se durmo à tarde tenho dificuldade de dormir à noite	Menos de 1 vez por semana	Muito boa
A falta de uma rotina usual, sem necessidade de deslocamento para o local de trabalho e o contato com outras pessoas afetou bastante meu horário biológico e o ciclo de sono	3 ou mais vezes por semana	Ruim
	Nenhuma no último mês	Boa

	1 ou 2 vezes por semana	Ruim
	Nenhuma no último mês	Boa
	Nenhuma no último mês	Boa
	Nenhuma no último mês	Ruim
Fui ao banheiro durante noite devido estar grávida e a gestação intensificar a necessidade	Nenhuma no último mês	Boa
	Nenhuma no último mês	Ruim
	Nenhuma no último mês	Boa
	Nenhuma no último mês	Ruim
	Nenhuma no último mês	Ruim
Gatos kkkk	3 ou mais vezes por semana	Ruim
	Nenhuma no último mês	Ruim

Nenhuma	Nenhuma no último mês	Boa
	Nenhuma no último mês	Boa
	Nenhuma no último mês	Ruim
	Nenhuma no último mês	Boa
	Nenhuma no último mês	Boa
	Nenhuma no último mês	Boa
	Nenhuma no último mês	Boa
	Nenhuma no último mês	Boa
	Nenhuma no último mês	Muito boa
	Nenhuma no último mês	Boa
	Nenhuma no último mês	Boa
Excesso de pensamentos	1 ou 2 vezes por semana	Ruim

Ansiedade	Menos de 1 vez por semana	Muito boa
	Nenhuma no último mês	Boa
	Nenhuma no último mês	Boa
	Nenhuma no último mês	Boa
	Nenhuma no último mês	Boa
	Nenhuma no último mês	Boa
	Nenhuma no último mês	Ruim
Cansaço mental/Estresse	3 ou mais vezes por semana	Ruim
	Nenhuma no último mês	Boa
ansiedade	1 ou 2 vezes por semana	Ruim
	Nenhuma no último mês	Ruim
	Nenhuma no último mês	Boa

Excesso de estimulante	Menos de 1 vez por semana	Boa
Filho com idade inferior a 1 ano que acorda a noite inteira é o principal fator de minhas noites mal dormidas.	3 ou mais vezes por semana	Ruim
Ansiedade e preocupações	1 ou 2 vezes por semana	Ruim
Não	Nenhuma no último mês	Ruim
	Nenhuma no último mês	Boa
	Nenhuma no último mês	Boa
	Nenhuma no último mês	Boa
Barulho de vizinhos :)	1 ou 2 vezes por semana	Boa
Gatos da casa fazendo barulho	1 ou 2 vezes por semana	Ruim
Exercício de força ajudou na tensão também	Nenhuma no último mês	Boa
	Nenhuma no último mês	Boa

	3 ou mais vezes por semana	Muito Ruim
Já tive dificuldade para dormir por conta de zumbido no ouvido, mas como já me acostumei, acredito não ter afetado meu sono durante o último mês.	Nenhuma no último mês	Boa
	Nenhuma no último mês	Muito boa
Pensamento acelerado, preocupação	1 ou 2 vezes por semana	Boa
Ansiedade.	3 ou mais vezes por semana	Ruim
Ansiedade	3 ou mais vezes por semana	Muito Ruim
	Nenhuma no último mês	Boa
	Nenhuma no último mês	Boa
	Nenhuma no último mês	Boa
	Nenhuma no último mês	Boa
	Menos de 1 vez por semana	Boa

	Nenhuma no último mês	Ruim
	Nenhuma no último mês	Muito boa
Crianças chorando	3 ou mais vezes por semana	Boa
	Nenhuma no último mês	Boa
	Nenhuma no último mês	Ruim
	Nenhuma no último mês	Boa
Tive um término de casamento recente, portanto desencadeou alguns desconfortos.	3 ou mais vezes por semana	Ruim
	Nenhuma no último mês	Muito boa
Nenhuma	Nenhuma no último mês	Boa
Preocupações	1 ou 2 vezes por semana	Ruim
	Nenhuma no último mês	Ruim

	1 ou 2 vezes por semana	Boa
	Nenhuma no último mês	Ruim
	Nenhuma no último mês	Ruim
moro numa rua ruidosa que atrapanha demais o sono	3 ou mais vezes por semana	Ruim
	Nenhuma no último mês	Boa
	Nenhuma no último mês	Muito Ruim
	Nenhuma no último mês	Boa
	Nenhuma no último mês	Ruim
Por não conseguir parar de pensar e refletir sobre questões do cotidiano	3 ou mais vezes por semana	Ruim
Insônia, estresse	3 ou mais vezes por semana	Ruim
	Nenhuma no último mês	Boa
Não	Nenhuma no último mês	Ruim

	Nenhuma no último mês	Boa
	Nenhuma no último mês	Boa
	Nenhuma no último mês	Boa
	Nenhuma no último mês	Boa
	Nenhuma no último mês	Boa
	Nenhuma no último mês	Boa
Poluição sonora (Causada por festas de vizinhos)	1 ou 2 vezes por semana	Muito Ruim
	Nenhuma no último mês	Boa
Dor no nervo ciático	Menos de 1 vez por semana	Boa
	Nenhuma no último mês	Boa
	Nenhuma no último mês	Boa

	Nenhuma no último mês	Boa
A rotina de home office, apesar de mais confortável pelo fato de não ter que acordar muito cedo e pegar metro lotado, é mais estrassante e exaustiva do que trabalhar presencialmente na empresa.	3 ou mais vezes por semana	Ruim
Perder o sono	3 ou mais vezes por semana	Ruim
Amamentação, sono do bebê pequeno	3 ou mais vezes por semana	Ruim
	Nenhuma no último mês	Ruim
Preocupações diversas	Menos de 1 vez por semana	Boa
Preocupação com a nova rotina familiar (bebê com menos de 1 ano)	1 ou 2 vezes por semana	Boa
	Nenhuma no último mês	Boa
	Nenhuma no último mês	Boa
Minha ansiedade e preocupação afetam o sono	1 ou 2 vezes por semana	Boa

	1 ou 2 vezes por semana	Ruim
Sintomas relatados acredito ser devido gestação, estou grávida de 35 semanas.	1 ou 2 vezes por semana	Ruim
torci o tornozelo	Menos de 1 vez por semana	Boa
	Nenhuma no último mês	Ruim
	Nenhuma no último mês	Boa
	Nenhuma no último mês	Boa
Tenho dificuldade para começar a dormir. Isso se acentua quando tenho algum compromisso fora de casa, bem cedo, na manhã do dia seguinte	Menos de 1 vez por semana	Boa
	Nenhuma no último mês	Boa
	Nenhuma no último mês	Boa
	Nenhuma no último mês	Ruim
	Nenhuma no último mês	Muito boa

nada	Nenhuma no último mês	Boa
	Nenhuma no último mês	Boa
Filho acorda durante a noite	3 ou mais vezes por semana	Ruim
	Nenhuma no último mês	Ruim
	Nenhuma no último mês	Boa
nenhuma razão	Nenhuma no último mês	Ruim
	Nenhuma no último mês	Ruim
	Nenhuma no último mês	Boa
	Nenhuma no último mês	Muito boa
	Nenhuma no último mês	Boa
Preocupações em casa	3 ou mais vezes por semana	Muito Ruim

	Nenhuma no último mês	Ruim
	Nenhuma no último mês	Ruim
	Nenhuma no último mês	Muito boa
	Nenhuma no último mês	Ruim
	Nenhuma no último mês	Ruim
	Nenhuma no último mês	Muito Ruim
	Nenhuma no último mês	Boa
	Nenhuma no último mês	Ruim
Nunca tive outrora quaisquer problemas de saúde pelo trabalho. Mas, esses últimos meses desde julho/2020 estou muito doente com 3 hérnias de disco e vários problemas emocionais.	3 ou mais vezes por semana	Ruim
	Nenhuma no último mês	Ruim

	Nenhuma no último mês	Muito boa
	Nenhuma no último mês	Ruim
barulho no vizinho	Menos de 1 vez por semana	Boa
	Nenhuma no último mês	Ruim
Cansaço mental e estresse.	3 ou mais vezes por semana	Muito Ruim
	Nenhuma no último mês	Boa
	Nenhuma no último mês	Ruim
	Nenhuma no último mês	Boa
Cessação de antidepressivo	3 ou mais vezes por semana	Boa
	Nenhuma no último mês	Muito boa
	Menos de 1 vez por semana	Ruim
	Nenhuma no último mês	Ruim

	Nenhuma no último mês	Ruim
nenhuma	Nenhuma no último mês	Ruim
Preocupação e planejamento do dia seguinte	Menos de 1 vez por semana	Boa
	Nenhuma no último mês	Boa
	Nenhuma no último mês	Boa
	3 ou mais vezes por semana	Ruim
	3 ou mais vezes por semana	Ruim
	Nenhuma no último mês	Boa
	Nenhuma no último mês	Boa
	Nenhuma no último mês	Boa
	Nenhuma no último mês	Boa
Bebê com 8 meses acordando a noite toda	3 ou mais vezes por semana	Muito Ruim

Pensando em atividades Do trabalho	1 ou 2 vezes por semana	Muito Ruim
	Nenhuma no último mês	Ruim
	Nenhuma no último mês	Boa
	Nenhuma no último mês	Muito boa
	Nenhuma no último mês	Boa
Preocupações com a família na pandemia	3 ou mais vezes por semana	Ruim
Preocupação	1 ou 2 vezes por semana	Ruim
	Nenhuma no último mês	Boa
Ansiedade, preocupação com a situação sanitária	1 ou 2 vezes por semana	Boa
	Nenhuma no último mês	Boa
	Nenhuma no último mês	Boa
	Nenhuma no último mês	Boa

	Nenhuma no último mês	Boa
	Nenhuma no último mês	Boa
	Nenhuma no último mês	Muito boa
	Nenhuma no último mês	Ruim
	Menos de 1 vez por semana	Boa
	Nenhuma no último mês	Boa
Ansiedade é a causa de maior desconforto	Menos de 1 vez por semana	Boa
Agitação	Menos de 1 vez por semana	Ruim
Simplesmente, acordo e não durmo mais	Menos de 1 vez por semana	Ruim
	Nenhuma no último mês	Ruim
	Nenhuma no último mês	Ruim
Dor no ombro no último mês piorou	3 ou mais vezes por semana	Muito Ruim

	Nenhuma no último mês	Ruim
Preocupação	Menos de 1 vez por semana	Boa
criança pequena acordando	3 ou mais vezes por semana	Boa
	Nenhuma no último mês	Boa
A dor que sinto é devido o exército físico frito 3 x por semana, tempo de exercício é de. 1:30h	Nenhuma no último mês	Muito boa
Não	Nenhuma no último mês	Boa
	Nenhuma no último mês	Boa
Meu cachorro sobe na cama	1 ou 2 vezes por semana	Muito boa
não	Nenhuma no último mês	Muito boa
	Nenhuma no último mês	Boa
Dormi bem	Nenhuma no último mês	Boa
	Nenhuma no último mês	Boa

Ronco do marido. Preocupações com saúde de familiares e com a pandemia e a política.	3 ou mais vezes por semana	Ruim
	Nenhuma no último mês	Ruim
	Nenhuma no último mês	Boa
	Nenhuma no último mês	Ruim
	Nenhuma no último mês	Boa

ttsburgh (PSQI)

Durante o último mês, com que frequência você tomou medicamento (prescrito ou "por conta própria")para lhe ajudar a dormir?	No último mês, com que frequência você teve dificuldade de ficar acordado enquanto dirigia, comia ou participava de uma atividade social?	Durante o último mês, quão problemático foi para você manter o entusiasmo (ânimo) para fazer as coisas (suas atividades habituais)?
3 ou mais vezes por semana	1 ou 2 vezes por semana	Um problema razoável
Nenhuma no último mês	Nenhuma no último mês	Um problema leve
Nenhuma no último mês	Nenhuma no último mês	Um problema razoável
1 ou 2 vezes por semana	1 ou 2 vezes por semana	Um grande problema
Nenhuma no último mês	Nenhuma no último mês	Um problema razoável
Nenhuma no último mês	Menos de 1 vez por semana	Um problema razoável
Nenhuma no último mês	Menos de 1 vez por semana	Um problema leve
Nenhuma no último mês	Nenhuma no último mês	Um problema leve
Menos de 1 vez por semana	Nenhuma no último mês	Um problema leve

3 ou mais vezes por semana	Nenhuma no último mês	Um grande problema
Nenhuma no último mês	Menos de 1 vez por semana	Um grande problema
1 ou 2 vezes por semana	Nenhuma no último mês	Um grande problema
Nenhuma no último mês	Menos de 1 vez por semana	Um problema leve
Nenhuma no último mês	Menos de 1 vez por semana	Um grande problema
Nenhuma no último mês	1 ou 2 vezes por semana	Um grande problema
Nenhuma no último mês	Nenhuma no último mês	Um problema razoável
Nenhuma no último mês	Nenhuma no último mês	Um problema leve
Nenhuma no último mês	Nenhuma no último mês	Um problema razoável
Nenhuma no último mês	Nenhuma no último mês	Um grande problema
Nenhuma no último mês	Nenhuma no último mês	Nenhuma dificuldade

Nenhuma no último mês	Menos de 1 vez por semana	Um problema razoável
Nenhuma no último mês	Menos de 1 vez por semana	Um grande problema
Nenhuma no último mês	Nenhuma no último mês	Um problema leve
Nenhuma no último mês	Menos de 1 vez por semana	Um problema razoável
3 ou mais vezes por semana	1 ou 2 vezes por semana	Um grande problema
Nenhuma no último mês	Menos de 1 vez por semana	Um problema razoável
Menos de 1 vez por semana	Nenhuma no último mês	Um problema razoável
1 ou 2 vezes por semana	Menos de 1 vez por semana	Um grande problema
Nenhuma no último mês	Menos de 1 vez por semana	Um problema razoável
Nenhuma no último mês	Menos de 1 vez por semana	Um problema razoável

Nenhuma no último mês	Nenhuma no último mês	Um problema razoável
Nenhuma no último mês	Nenhuma no último mês	Um grande problema
Nenhuma no último mês	Nenhuma no último mês	Um grande problema
Nenhuma no último mês	Nenhuma no último mês	Um grande problema
Nenhuma no último mês	Nenhuma no último mês	Um grande problema
Nenhuma no último mês	Menos de 1 vez por semana	Nenhuma dificuldade
Nenhuma no último mês	Menos de 1 vez por semana	Um problema razoável
Nenhuma no último mês	Nenhuma no último mês	Um grande problema
Nenhuma no último mês	Menos de 1 vez por semana	Um grande problema
Nenhuma no último mês	Nenhuma no último mês	Um problema razoável
Nenhuma no último mês	Nenhuma no último mês	Um grande problema
Nenhuma no último mês	Nenhuma no último mês	Um problema leve

Nenhuma no último mês	Menos de 1 vez por semana	Um grande problema
Menos de 1 vez por semana	Nenhuma no último mês	Um problema razoável
Nenhuma no último mês	Menos de 1 vez por semana	Um problema razoável
Nenhuma no último mês	Nenhuma no último mês	Um problema razoável
Nenhuma no último mês	Nenhuma no último mês	Nenhuma dificuldade
Nenhuma no último mês	Menos de 1 vez por semana	Um problema razoável
1 ou 2 vezes por semana	Nenhuma no último mês	Um problema leve
3 ou mais vezes por semana	Nenhuma no último mês	Um problema leve
Menos de 1 vez por semana	Menos de 1 vez por semana	Um problema razoável
Nenhuma no último mês	Menos de 1 vez por semana	Um problema leve

3 ou mais vezes por semana	3 ou mais vezes por semana	Um problema razoável
Nenhuma no último mês	Menos de 1 vez por semana	Um grande problema
Nenhuma no último mês	Menos de 1 vez por semana	Um problema leve
Nenhuma no último mês	Nenhuma no último mês	Um problema razoável
Nenhuma no último mês	Nenhuma no último mês	Um problema razoável
Nenhuma no último mês	Menos de 1 vez por semana	Um grande problema
3 ou mais vezes por semana	Menos de 1 vez por semana	Um problema leve
Nenhuma no último mês	Menos de 1 vez por semana	Um grande problema
Nenhuma no último mês	Nenhuma no último mês	Nenhuma dificuldade
Nenhuma no último mês	Menos de 1 vez por semana	Um grande problema
Nenhuma no último mês	Nenhuma no último mês	Um problema razoável

Nenhuma no último mês	Menos de 1 vez por semana	Um grande problema
Nenhuma no último mês	Nenhuma no último mês	Um problema leve
Nenhuma no último mês	Menos de 1 vez por semana	Um problema leve
Nenhuma no último mês	Menos de 1 vez por semana	Um grande problema
Nenhuma no último mês	Nenhuma no último mês	Nenhuma dificuldade
Nenhuma no último mês	Menos de 1 vez por semana	Um problema razoável
Nenhuma no último mês	Nenhuma no último mês	Nenhuma dificuldade
Nenhuma no último mês	Nenhuma no último mês	Nenhuma dificuldade
Nenhuma no último mês	Nenhuma no último mês	Um problema leve
Nenhuma no último mês	Nenhuma no último mês	Um problema razoável
Nenhuma no último mês	Nenhuma no último mês	Um problema razoável

3 ou mais vezes por semana	Menos de 1 vez por semana	Um problema razoável
Nenhuma no último mês	Nenhuma no último mês	Um problema leve
Nenhuma no último mês	Nenhuma no último mês	Nenhuma dificuldade
Nenhuma no último mês	Nenhuma no último mês	Um problema razoável
Nenhuma no último mês	Nenhuma no último mês	Um problema leve
Nenhuma no último mês	Menos de 1 vez por semana	Um problema razoável
Nenhuma no último mês	1 ou 2 vezes por semana	Um problema leve
Nenhuma no último mês	Nenhuma no último mês	Um problema leve
Nenhuma no último mês	Nenhuma no último mês	Um problema razoável
Nenhuma no último mês	3 ou mais vezes por semana	Um grande problema
3 ou mais vezes por semana	Nenhuma no último mês	Um problema leve
3 ou mais vezes por semana	1 ou 2 vezes por semana	Um problema razoável

Nenhuma no último mês	Nenhuma no último mês	Nenhuma dificuldade
Nenhuma no último mês	Menos de 1 vez por semana	Um problema leve
Nenhuma no último mês	Nenhuma no último mês	Um problema razoável
Nenhuma no último mês	Menos de 1 vez por semana	Um problema leve
Nenhuma no último mês	Nenhuma no último mês	Um grande problema
Nenhuma no último mês	Nenhuma no último mês	Um problema razoável
Nenhuma no último mês	Nenhuma no último mês	Um problema leve
3 ou mais vezes por semana	1 ou 2 vezes por semana	Um grande problema
Menos de 1 vez por semana	Nenhuma no último mês	Um problema leve
3 ou mais vezes por semana	1 ou 2 vezes por semana	Um problema leve
Nenhuma no último mês	Nenhuma no último mês	Um problema razoável
1 ou 2 vezes por semana	Nenhuma no último mês	Um problema razoável

Nenhuma no último mês	Menos de 1 vez por semana	Um grande problema
Nenhuma no último mês	1 ou 2 vezes por semana	Um problema razoável
Nenhuma no último mês	Nenhuma no último mês	Um grande problema
Nenhuma no último mês	Nenhuma no último mês	Um problema leve
Nenhuma no último mês	Nenhuma no último mês	Nenhuma dificuldade
Nenhuma no último mês	Menos de 1 vez por semana	Um problema leve
Nenhuma no último mês	Nenhuma no último mês	Um grande problema
Nenhuma no último mês	Nenhuma no último mês	Um grande problema
Nenhuma no último mês	Menos de 1 vez por semana	Um grande problema
3 ou mais vezes por semana	1 ou 2 vezes por semana	Um grande problema
Nenhuma no último mês	Nenhuma no último mês	Um problema leve

3 ou mais vezes por semana	Menos de 1 vez por semana	Um problema razoável
Menos de 1 vez por semana	Menos de 1 vez por semana	Um problema razoável
3 ou mais vezes por semana	3 ou mais vezes por semana	Um grande problema
Nenhuma no último mês	1 ou 2 vezes por semana	Um problema leve
Nenhuma no último mês	Nenhuma no último mês	Um problema razoável
3 ou mais vezes por semana	Menos de 1 vez por semana	Um problema razoável
Nenhuma no último mês	Menos de 1 vez por semana	Um grande problema
Nenhuma no último mês	Nenhuma no último mês	Um problema razoável
Nenhuma no último mês	Nenhuma no último mês	Nenhuma dificuldade
Nenhuma no último mês	Menos de 1 vez por semana	Um problema razoável

Nenhuma no último mês	Nenhuma no último mês	Um problema leve
Nenhuma no último mês	Menos de 1 vez por semana	Um grande problema
Nenhuma no último mês	Nenhuma no último mês	Um problema leve
Nenhuma no último mês	Nenhuma no último mês	Um problema razoável
Nenhuma no último mês	Menos de 1 vez por semana	Um problema leve
Nenhuma no último mês	Menos de 1 vez por semana	Um problema razoável
Nenhuma no último mês	Menos de 1 vez por semana	Um problema leve
Nenhuma no último mês	Menos de 1 vez por semana	Um problema leve
Nenhuma no último mês	Nenhuma no último mês	Um problema leve
Nenhuma no último mês	Nenhuma no último mês	Um grande problema
Nenhuma no último mês	3 ou mais vezes por semana	Um grande problema

Nenhuma no último mês	1 ou 2 vezes por semana	Um problema razoável
1 ou 2 vezes por semana	Menos de 1 vez por semana	Um grande problema
Menos de 1 vez por semana	Nenhuma no último mês	Um problema razoável
Menos de 1 vez por semana	Nenhuma no último mês	Um problema razoável
Nenhuma no último mês	Nenhuma no último mês	Um problema razoável
Nenhuma no último mês	Menos de 1 vez por semana	Um problema razoável
Nenhuma no último mês	Menos de 1 vez por semana	Um problema leve
Menos de 1 vez por semana	Nenhuma no último mês	Um problema razoável
Nenhuma no último mês	Nenhuma no último mês	Um grande problema
Menos de 1 vez por semana	Menos de 1 vez por semana	Um grande problema
Nenhuma no último mês	Nenhuma no último mês	Um problema razoável

Nenhuma no último mês	Nenhuma no último mês	Nenhuma dificuldade
Nenhuma no último mês	Menos de 1 vez por semana	Um problema razoável
3 ou mais vezes por semana	Nenhuma no último mês	Um grande problema
Nenhuma no último mês	Menos de 1 vez por semana	Nenhuma dificuldade
Menos de 1 vez por semana	Nenhuma no último mês	Um grande problema
Nenhuma no último mês	Nenhuma no último mês	Um problema razoável
Menos de 1 vez por semana	Menos de 1 vez por semana	Um problema leve
Nenhuma no último mês	Nenhuma no último mês	Um problema leve
1 ou 2 vezes por semana	Nenhuma no último mês	Um grande problema
Nenhuma no último mês	Nenhuma no último mês	Um grande problema
Nenhuma no último mês	Nenhuma no último mês	Nenhuma dificuldade
Nenhuma no último mês	Nenhuma no último mês	Nenhuma dificuldade

Nenhuma no último mês	Nenhuma no último mês	Um problema leve
Nenhuma no último mês	Nenhuma no último mês	Um problema leve
Nenhuma no último mês	Nenhuma no último mês	Um problema leve
Nenhuma no último mês	Menos de 1 vez por semana	Um problema leve
Nenhuma no último mês	Nenhuma no último mês	Nenhuma dificuldade
Nenhuma no último mês	Nenhuma no último mês	Um problema razoável
Nenhuma no último mês	1 ou 2 vezes por semana	Um problema razoável
Nenhuma no último mês	Menos de 1 vez por semana	Um problema razoável
Menos de 1 vez por semana	Nenhuma no último mês	Um problema razoável
Nenhuma no último mês	Nenhuma no último mês	Um problema razoável
Menos de 1 vez por semana	Nenhuma no último mês	Um grande problema
Nenhuma no último mês	Nenhuma no último mês	Um problema leve

Nenhuma no último mês	Nenhuma no último mês	Um problema leve
Nenhuma no último mês	Menos de 1 vez por semana	Um problema leve
Nenhuma no último mês	1 ou 2 vezes por semana	Um grande problema
Nenhuma no último mês	1 ou 2 vezes por semana	Um grande problema
Nenhuma no último mês	1 ou 2 vezes por semana	Um problema leve
Nenhuma no último mês	Nenhuma no último mês	Um problema razoável
Nenhuma no último mês	Menos de 1 vez por semana	Um problema leve
Nenhuma no último mês	Menos de 1 vez por semana	Um problema leve
1 ou 2 vezes por semana	Menos de 1 vez por semana	Um problema leve
Nenhuma no último mês	Menos de 1 vez por semana	Um problema leve
Nenhuma no último mês	Menos de 1 vez por semana	Um problema leve

Nenhuma no último mês	Nenhuma no último mês	Um grande problema
Nenhuma no último mês	Nenhuma no último mês	Um problema leve
Nenhuma no último mês	Nenhuma no último mês	Nenhuma dificuldade
Nenhuma no último mês	1 ou 2 vezes por semana	Um problema leve
Nenhuma no último mês	Menos de 1 vez por semana	Um problema razoável
3 ou mais vezes por semana	Menos de 1 vez por semana	Um problema leve
Nenhuma no último mês	Menos de 1 vez por semana	Um problema razoável
Nenhuma no último mês	Nenhuma no último mês	Um problema razoável
Menos de 1 vez por semana	Nenhuma no último mês	Um problema leve
Nenhuma no último mês	Nenhuma no último mês	Um problema leve
Nenhuma no último mês	Nenhuma no último mês	Um grande problema

1 ou 2 vezes por semana	Nenhuma no último mês	Um problema leve
Nenhuma no último mês	Nenhuma no último mês	Um problema leve
3 ou mais vezes por semana	Nenhuma no último mês	Um problema razoável
Nenhuma no último mês	Nenhuma no último mês	Um problema razoável
Nenhuma no último mês	Nenhuma no último mês	Um grande problema
Nenhuma no último mês	Nenhuma no último mês	Um problema leve
3 ou mais vezes por semana	Menos de 1 vez por semana	Um grande problema
Nenhuma no último mês	Nenhuma no último mês	Nenhuma dificuldade
Nenhuma no último mês	Nenhuma no último mês	Um problema leve
1 ou 2 vezes por semana	1 ou 2 vezes por semana	Um problema razoável
Menos de 1 vez por semana	Nenhuma no último mês	Um problema razoável

3 ou mais vezes por semana	Nenhuma no último mês	Nenhuma dificuldade
Nenhuma no último mês	Nenhuma no último mês	Um problema leve
Nenhuma no último mês	Nenhuma no último mês	Um problema razoável
Nenhuma no último mês	Nenhuma no último mês	Um problema razoável
Nenhuma no último mês	Nenhuma no último mês	Um problema leve
Nenhuma no último mês	Nenhuma no último mês	Um grande problema
Nenhuma no último mês	Nenhuma no último mês	Um problema leve
1 ou 2 vezes por semana	Nenhuma no último mês	Um problema razoável
Menos de 1 vez por semana	Menos de 1 vez por semana	Um problema razoável
3 ou mais vezes por semana	3 ou mais vezes por semana	Um grande problema
Nenhuma no último mês	Nenhuma no último mês	Um problema leve
Nenhuma no último mês	Menos de 1 vez por semana	Um problema leve

Nenhuma no último mês	Menos de 1 vez por semana	Um grande problema
Nenhuma no último mês	Nenhuma no último mês	Um problema leve
Nenhuma no último mês	Menos de 1 vez por semana	Um problema leve
Nenhuma no último mês	Menos de 1 vez por semana	Um problema razoável
Nenhuma no último mês	Nenhuma no último mês	Um problema leve
Nenhuma no último mês	Nenhuma no último mês	Um problema razoável
Nenhuma no último mês	Nenhuma no último mês	Nenhuma dificuldade
Nenhuma no último mês	Nenhuma no último mês	Um problema razoável
Menos de 1 vez por semana	Nenhuma no último mês	Um grande problema
Nenhuma no último mês	Nenhuma no último mês	Um problema razoável
Nenhuma no último mês	1 ou 2 vezes por semana	Um problema razoável

Nenhuma no último mês	Nenhuma no último mês	Um problema leve
3 ou mais vezes por semana	Nenhuma no último mês	Um grande problema
Nenhuma no último mês	Nenhuma no último mês	Um grande problema
Menos de 1 vez por semana	Nenhuma no último mês	Um problema leve
Nenhuma no último mês	Nenhuma no último mês	Um grande problema
Nenhuma no último mês	Nenhuma no último mês	Um problema leve
Nenhuma no último mês	Nenhuma no último mês	Um problema leve
Nenhuma no último mês	Menos de 1 vez por semana	Um grande problema
Nenhuma no último mês	Nenhuma no último mês	Um grande problema
3 ou mais vezes por semana	Nenhuma no último mês	Um problema razoável

Nenhuma no último mês	Menos de 1 vez por semana	Um problema leve
Nenhuma no último mês	Menos de 1 vez por semana	Um problema razoável
Menos de 1 vez por semana	Nenhuma no último mês	Um problema leve
Nenhuma no último mês	Nenhuma no último mês	Um problema razoável
Nenhuma no último mês	Nenhuma no último mês	Um problema leve
Nenhuma no último mês	Menos de 1 vez por semana	Um problema razoável
Nenhuma no último mês	Nenhuma no último mês	Um problema razoável
1 ou 2 vezes por semana	Nenhuma no último mês	Um problema razoável
Nenhuma no último mês	Nenhuma no último mês	Um problema razoável
3 ou mais vezes por semana	Nenhuma no último mês	Um problema razoável
Nenhuma no último mês	Nenhuma no último mês	Um problema leve

Nenhuma no último mês	Nenhuma no último mês	Um problema razoável
Nenhuma no último mês	Nenhuma no último mês	Um problema razoável
Nenhuma no último mês	Nenhuma no último mês	Um problema razoável
Menos de 1 vez por semana	Nenhuma no último mês	Um problema razoável
3 ou mais vezes por semana	Nenhuma no último mês	Um problema razoável
Nenhuma no último mês	Menos de 1 vez por semana	Um problema leve
Nenhuma no último mês	Menos de 1 vez por semana	Um grande problema
Menos de 1 vez por semana	Nenhuma no último mês	Um problema razoável
Nenhuma no último mês	Nenhuma no último mês	Um problema leve
Nenhuma no último mês	3 ou mais vezes por semana	Um grande problema
3 ou mais vezes por semana	1 ou 2 vezes por semana	Um grande problema

Nenhuma no último mês	Nenhuma no último mês	Um problema leve
Nenhuma no último mês	Menos de 1 vez por semana	Um problema razoável
Nenhuma no último mês	Nenhuma no último mês	Nenhuma dificuldade
3 ou mais vezes por semana	Nenhuma no último mês	Um grande problema
Nenhuma no último mês	Menos de 1 vez por semana	Um problema leve
Nenhuma no último mês	Menos de 1 vez por semana	Um problema leve
Nenhuma no último mês	Nenhuma no último mês	Um problema leve
Nenhuma no último mês	Menos de 1 vez por semana	Um problema razoável
Menos de 1 vez por semana	Menos de 1 vez por semana	Um grande problema
1 ou 2 vezes por semana	1 ou 2 vezes por semana	Um problema razoável

Nenhuma no último mês	Nenhuma no último mês	Nenhuma dificuldade
Menos de 1 vez por semana	Nenhuma no último mês	Um problema leve
Nenhuma no último mês	Menos de 1 vez por semana	Um problema razoável
Nenhuma no último mês	Menos de 1 vez por semana	Um grande problema
Nenhuma no último mês	Nenhuma no último mês	Um problema razoável
Nenhuma no último mês	Nenhuma no último mês	Nenhuma dificuldade
Nenhuma no último mês	1 ou 2 vezes por semana	Um problema razoável
Nenhuma no último mês	Nenhuma no último mês	Um problema leve
Nenhuma no último mês	Nenhuma no último mês	Um problema leve
Nenhuma no último mês	Nenhuma no último mês	Um problema razoável
Nenhuma no último mês	1 ou 2 vezes por semana	Um problema razoável
Nenhuma no último mês	Nenhuma no último mês	Um problema leve

Nenhuma no último mês	Nenhuma no último mês	Um problema razoável
Nenhuma no último mês	Menos de 1 vez por semana	Um problema leve
Nenhuma no último mês	Nenhuma no último mês	Um problema leve
Nenhuma no último mês	Nenhuma no último mês	Nenhuma dificuldade
Menos de 1 vez por semana	Nenhuma no último mês	Um problema leve
Nenhuma no último mês	Menos de 1 vez por semana	Um grande problema
Menos de 1 vez por semana	Menos de 1 vez por semana	Um problema razoável
Nenhuma no último mês	Nenhuma no último mês	Um problema leve
Nenhuma no último mês	Nenhuma no último mês	Um problema leve
Nenhuma no último mês	Nenhuma no último mês	Um problema leve
Menos de 1 vez por semana	1 ou 2 vezes por semana	Um problema leve
Nenhuma no último mês	Nenhuma no último mês	Um problema razoável

Nenhuma no último mês	1 ou 2 vezes por semana	Nenhuma dificuldade
Nenhuma no último mês	Nenhuma no último mês	Um problema razoável
Nenhuma no último mês	Nenhuma no último mês	Um problema razoável
Nenhuma no último mês	Nenhuma no último mês	Um problema leve
Nenhuma no último mês	Nenhuma no último mês	Nenhuma dificuldade
Nenhuma no último mês	Nenhuma no último mês	Um grande problema
Nenhuma no último mês	Menos de 1 vez por semana	Um problema leve
Nenhuma no último mês	Nenhuma no último mês	Um problema leve
Nenhuma no último mês	Nenhuma no último mês	Um problema leve
Nenhuma no último mês	Menos de 1 vez por semana	Um problema razoável
Nenhuma no último mês	Nenhuma no último mês	Nenhuma dificuldade
Nenhuma no último mês	Nenhuma no último mês	Um problema razoável

Nenhuma no último mês	Nenhuma no último mês	Nenhuma dificuldade
Nenhuma no último mês	Nenhuma no último mês	Nenhuma dificuldade
Nenhuma no último mês	Menos de 1 vez por semana	Um grande problema
3 ou mais vezes por semana	Nenhuma no último mês	Um grande problema
Nenhuma no último mês	Nenhuma no último mês	Um problema leve
Nenhuma no último mês	Nenhuma no último mês	Nenhuma dificuldade
Nenhuma no último mês	Menos de 1 vez por semana	Um problema razoável
3 ou mais vezes por semana	Nenhuma no último mês	Um grande problema
Menos de 1 vez por semana	3 ou mais vezes por semana	Um grande problema
Menos de 1 vez por semana	1 ou 2 vezes por semana	Um problema razoável
Menos de 1 vez por semana	Nenhuma no último mês	Um problema razoável
3 ou mais vezes por semana	1 ou 2 vezes por semana	Um grande problema

3 ou mais vezes por semana	Nenhuma no último mês	Um problema razoável
Nenhuma no último mês	Nenhuma no último mês	Um problema razoável
Nenhuma no último mês	Nenhuma no último mês	Nenhuma dificuldade
Nenhuma no último mês	Nenhuma no último mês	Nenhuma dificuldade
3 ou mais vezes por semana	Nenhuma no último mês	Um problema leve
Nenhuma no último mês	Menos de 1 vez por semana	Um problema leve
Nenhuma no último mês	Menos de 1 vez por semana	Nenhuma dificuldade
Nenhuma no último mês	Menos de 1 vez por semana	Um problema razoável
Nenhuma no último mês	Nenhuma no último mês	Nenhuma dificuldade
Nenhuma no último mês	Nenhuma no último mês	Nenhuma dificuldade
Nenhuma no último mês	Nenhuma no último mês	Um problema razoável
Nenhuma no último mês	Nenhuma no último mês	Um problema leve

Nenhuma no último mês	Menos de 1 vez por semana	Um problema razoável
Menos de 1 vez por semana	Nenhuma no último mês	Um problema leve
Nenhuma no último mês	Nenhuma no último mês	Um problema leve
Nenhuma no último mês	Nenhuma no último mês	Um problema razoável
Nenhuma no último mês	Nenhuma no último mês	Nenhuma dificuldade

Você tem um(a) parceiro(a) ou colega de quarto?	Se você tem um parceiro ou colega de quarto, pergunta a ele/ela com que frequência no último mês você teve ronco forte?	Longas paradas na respiração enquanto dormia?
Parceiro na mesma cama	Nenhuma no último mês	Nenhuma no último mês
Não	Não se aplica (Não tenho parceiro para responder)	Não se aplica (Não tenho parceiro para responder)
Parceiro na mesma cama	Nenhuma no último mês	Nenhuma no último mês
Não	Não se aplica (Não tenho parceiro para responder)	Não se aplica (Não tenho parceiro para responder)
Parceiro na mesma cama	Nenhuma no último mês	Nenhuma no último mês
Não	Não se aplica (Não tenho parceiro para responder)	Não se aplica (Não tenho parceiro para responder)
Não	Não se aplica (Não tenho parceiro para responder)	Não se aplica (Não tenho parceiro para responder)
Parceiro ou colega, mas em outro quarto	Não se aplica (Não tenho parceiro para responder)	Não se aplica (Não tenho parceiro para responder)
Parceiro na mesma cama	Nenhuma no último mês	Nenhuma no último mês

Não	Não se aplica (Não tenho parceiro para responder)	Não se aplica (Não tenho parceiro para responder)
Não	Não se aplica (Não tenho parceiro para responder)	Não se aplica (Não tenho parceiro para responder)
Parceiro na mesma cama	Menos que 1 vez por semana	Nenhuma no último mês
Parceiro na mesma cama	Nenhuma no último mês	Nenhuma no último mês
Não	Não se aplica (Não tenho parceiro para responder)	Não se aplica (Não tenho parceiro para responder)
Não	Não se aplica (Não tenho parceiro para responder)	Não se aplica (Não tenho parceiro para responder)
Não	Não se aplica (Não tenho parceiro para responder)	Não se aplica (Não tenho parceiro para responder)
Parceiro na mesma cama	Nenhuma no último mês	Nenhuma no último mês
Parceiro no mesmo quarto, mas não na mesma cama	Nenhuma no último mês	Nenhuma no último mês
Não	Não se aplica (Não tenho parceiro para responder)	Não se aplica (Não tenho parceiro para responder)
Parceiro na mesma cama	Nenhuma no último mês	Nenhuma no último mês

Parceiro no mesmo quarto, mas não na mesma cama	Nenhuma no último mês	Nenhuma no último mês
Parceiro ou colega, mas em outro quarto	Não se aplica (Não tenho parceiro para responder)	Não se aplica (Não tenho parceiro para responder)
Parceiro na mesma cama	1 ou 2 vezes por semana	Nenhuma no último mês
Parceiro na mesma cama	Nenhuma no último mês	Nenhuma no último mês
Parceiro no mesmo quarto, mas não na mesma cama	Nenhuma no último mês	Nenhuma no último mês
Não	Não se aplica (Não tenho parceiro para responder)	Não se aplica (Não tenho parceiro para responder)
Parceiro na mesma cama	Nenhuma no último mês	Nenhuma no último mês
Parceiro na mesma cama	Nenhuma no último mês	Nenhuma no último mês
Parceiro no mesmo quarto, mas não na mesma cama	Menos que 1 vez por semana	Nenhuma no último mês
Parceiro na mesma cama	Nenhuma no último mês	Nenhuma no último mês

Parceiro na mesma cama	Nenhuma no último mês	Nenhuma no último mês
Não	Não se aplica (Não tenho parceiro para responder)	Não se aplica (Não tenho parceiro para responder)
Não	Nenhuma no último mês	Não se aplica (Não tenho parceiro para responder)
Não	Não se aplica (Não tenho parceiro para responder)	Não se aplica (Não tenho parceiro para responder)
Parceiro na mesma cama	Nenhuma no último mês	Nenhuma no último mês
Parceiro no mesmo quarto, mas não na mesma cama	Nenhuma no último mês	Nenhuma no último mês
Parceiro na mesma cama	Menos que 1 vez por semana	Menos que uma vez por semana
Parceiro ou colega, mas em outro quarto	Não se aplica (Não tenho parceiro para responder)	Não se aplica (Não tenho parceiro para responder)
Não	Não se aplica (Não tenho parceiro para responder)	Não se aplica (Não tenho parceiro para responder)
Não	Não se aplica (Não tenho parceiro para responder)	Não se aplica (Não tenho parceiro para responder)
Parceiro no mesmo quarto, mas não na mesma cama	Menos que 1 vez por semana	Nenhuma no último mês
Não	Não se aplica (Não tenho parceiro para responder)	Não se aplica (Não tenho parceiro para responder)

Parceiro na mesma cama	Menos que 1 vez por semana	Nenhuma no último mês
Parceiro no mesmo quarto, mas não na mesma cama	Nenhuma no último mês	Nenhuma no último mês
Parceiro na mesma cama	Nenhuma no último mês	Nenhuma no último mês
Parceiro na mesma cama	Menos que 1 vez por semana	Menos que uma vez por semana
Não	Não se aplica (Não tenho parceiro para responder)	Não se aplica (Não tenho parceiro para responder)
Parceiro ou colega, mas em outro quarto	Não se aplica (Não tenho parceiro para responder)	Não se aplica (Não tenho parceiro para responder)
Não	Não se aplica (Não tenho parceiro para responder)	Não se aplica (Não tenho parceiro para responder)
Não	Não se aplica (Não tenho parceiro para responder)	Não se aplica (Não tenho parceiro para responder)
Não	Não se aplica (Não tenho parceiro para responder)	Não se aplica (Não tenho parceiro para responder)
Não	Não se aplica (Não tenho parceiro para responder)	Não se aplica (Não tenho parceiro para responder)

Parceiro ou colega, mas em outro quarto	Nenhuma no último mês	Nenhuma no último mês
Parceiro na mesma cama	Nenhuma no último mês	Nenhuma no último mês
Não	Não se aplica (Não tenho parceiro para responder)	Não se aplica (Não tenho parceiro para responder)
Parceiro na mesma cama	Nenhuma no último mês	Nenhuma no último mês
Não	Não se aplica (Não tenho parceiro para responder)	Não se aplica (Não tenho parceiro para responder)
Parceiro ou colega, mas em outro quarto	Nenhuma no último mês	Nenhuma no último mês
Parceiro ou colega, mas em outro quarto	Não se aplica (Não tenho parceiro para responder)	Não se aplica (Não tenho parceiro para responder)
Não	Não se aplica (Não tenho parceiro para responder)	Não se aplica (Não tenho parceiro para responder)
Parceiro na mesma cama	Menos que 1 vez por semana	Nenhuma no último mês
Não	Não se aplica (Não tenho parceiro para responder)	Não se aplica (Não tenho parceiro para responder)
Parceiro na mesma cama	3 ou mais vezes por semana	1 ou 2 vezes por semana

Não	Não se aplica (Não tenho parceiro para responder)	Não se aplica (Não tenho parceiro para responder)
Parceiro na mesma cama	Nenhuma no último mês	Nenhuma no último mês
Parceiro na mesma cama	Menos que 1 vez por semana	Nenhuma no último mês
Parceiro na mesma cama	Nenhuma no último mês	Nenhuma no último mês
Parceiro na mesma cama	Nenhuma no último mês	Nenhuma no último mês
Parceiro na mesma cama	Nenhuma no último mês	Nenhuma no último mês
Parceiro na mesma cama	Nenhuma no último mês	Nenhuma no último mês
Não	Não se aplica (Não tenho parceiro para responder)	Não se aplica (Não tenho parceiro para responder)
Parceiro na mesma cama	Nenhuma no último mês	Nenhuma no último mês
Parceiro na mesma cama	3 ou mais vezes por semana	Nenhuma no último mês
Parceiro ou colega, mas em outro quarto	3 ou mais vezes por semana	Não se aplica (Não tenho parceiro para responder)

Parceiro na mesma cama	3 ou mais vezes por semana	Nenhuma no último mês
Parceiro no mesmo quarto, mas não na mesma cama	Nenhuma no último mês	1 ou 2 vezes por semana
Parceiro na mesma cama	Nenhuma no último mês	Nenhuma no último mês
Não	Não se aplica (Não tenho parceiro para responder)	Não se aplica (Não tenho parceiro para responder)
Parceiro na mesma cama	Nenhuma no último mês	Nenhuma no último mês
Parceiro ou colega, mas em outro quarto	Não se aplica (Não tenho parceiro para responder)	Não se aplica (Não tenho parceiro para responder)
Parceiro no mesmo quarto, mas não na mesma cama	Não se aplica (Não tenho parceiro para responder)	Não se aplica (Não tenho parceiro para responder)
Parceiro na mesma cama	1 ou 2 vezes por semana	Nenhuma no último mês
Não	Não se aplica (Não tenho parceiro para responder)	Não se aplica (Não tenho parceiro para responder)
Parceiro na mesma cama	Nenhuma no último mês	Nenhuma no último mês
Não	Não se aplica (Não tenho parceiro para responder)	Não se aplica (Não tenho parceiro para responder)
Parceiro ou colega, mas em outro quarto	Menos que 1 vez por semana	Nenhuma no último mês

Parceiro ou colega, mas em outro quarto	Nenhuma no último mês	Nenhuma no último mês
Não	Não se aplica (Não tenho parceiro para responder)	Não se aplica (Não tenho parceiro para responder)
Não	Não se aplica (Não tenho parceiro para responder)	Não se aplica (Não tenho parceiro para responder)
Parceiro na mesma cama	Nenhuma no último mês	Nenhuma no último mês
Não	Não se aplica (Não tenho parceiro para responder)	Não se aplica (Não tenho parceiro para responder)
Não	Não se aplica (Não tenho parceiro para responder)	Não se aplica (Não tenho parceiro para responder)
Não	Não se aplica (Não tenho parceiro para responder)	Não se aplica (Não tenho parceiro para responder)
Não	Não se aplica (Não tenho parceiro para responder)	Não se aplica (Não tenho parceiro para responder)
Não	Não se aplica (Não tenho parceiro para responder)	Não se aplica (Não tenho parceiro para responder)
Parceiro ou colega, mas em outro quarto	Nenhuma no último mês	Nenhuma no último mês
Não	Não se aplica (Não tenho parceiro para responder)	Não se aplica (Não tenho parceiro para responder)
Parceiro ou colega, mas em outro quarto	Não se aplica (Não tenho parceiro para responder)	Não se aplica (Não tenho parceiro para responder)

Parceiro na mesma cama	Nenhuma no último mês	Nenhuma no último mês
Parceiro na mesma cama	Nenhuma no último mês	Nenhuma no último mês
Parceiro na mesma cama	Menos que 1 vez por semana	Nenhuma no último mês
Parceiro na mesma cama	1 ou 2 vezes por semana	Nenhuma no último mês
Parceiro na mesma cama	Nenhuma no último mês	Nenhuma no último mês
Não	Não se aplica (Não tenho parceiro para responder)	Não se aplica (Não tenho parceiro para responder)
Parceiro na mesma cama	Nenhuma no último mês	Nenhuma no último mês
Parceiro na mesma cama	Nenhuma no último mês	Nenhuma no último mês
Não	Não se aplica (Não tenho parceiro para responder)	Não se aplica (Não tenho parceiro para responder)
Parceiro na mesma cama	Nenhuma no último mês	Nenhuma no último mês
Não	Não se aplica (Não tenho parceiro para responder)	Não se aplica (Não tenho parceiro para responder)

Parceiro na mesma cama	Menos que 1 vez por semana	Nenhuma no último mês
Parceiro ou colega, mas em outro quarto	3 ou mais vezes por semana	3 ou mais vezes por semana
Parceiro na mesma cama	Nenhuma no último mês	Nenhuma no último mês
Não	Não se aplica (Não tenho parceiro para responder)	Nenhuma no último mês
Parceiro na mesma cama	Nenhuma no último mês	Nenhuma no último mês
Não	Não se aplica (Não tenho parceiro para responder)	Não se aplica (Não tenho parceiro para responder)
Parceiro ou colega, mas em outro quarto	Não se aplica (Não tenho parceiro para responder)	Não se aplica (Não tenho parceiro para responder)
Não	Não se aplica (Não tenho parceiro para responder)	Não se aplica (Não tenho parceiro para responder)
Parceiro na mesma cama	Nenhuma no último mês	Nenhuma no último mês
Parceiro na mesma cama	1 ou 2 vezes por semana	Nenhuma no último mês

Não	Não se aplica (Não tenho parceiro para responder)	Não se aplica (Não tenho parceiro para responder)
Não	Não se aplica (Não tenho parceiro para responder)	Não se aplica (Não tenho parceiro para responder)
Não	Não se aplica (Não tenho parceiro para responder)	Não se aplica (Não tenho parceiro para responder)
Não	Não se aplica (Não tenho parceiro para responder)	Não se aplica (Não tenho parceiro para responder)
Parceiro no mesmo quarto, mas não na mesma cama	Nenhuma no último mês	Nenhuma no último mês
Parceiro na mesma cama	Nenhuma no último mês	Nenhuma no último mês
Parceiro ou colega, mas em outro quarto	Nenhuma no último mês	Nenhuma no último mês
Parceiro na mesma cama	1 ou 2 vezes por semana	Nenhuma no último mês
Parceiro na mesma cama	Nenhuma no último mês	Nenhuma no último mês
Não	Não se aplica (Não tenho parceiro para responder)	Não se aplica (Não tenho parceiro para responder)
Parceiro ou colega, mas em outro quarto	3 ou mais vezes por semana	3 ou mais vezes por semana

Não	Não se aplica (Não tenho parceiro para responder)	Não se aplica (Não tenho parceiro para responder)
Parceiro na mesma cama	Nenhuma no último mês	Nenhuma no último mês
Parceiro na mesma cama	Nenhuma no último mês	Nenhuma no último mês
Não	Não se aplica (Não tenho parceiro para responder)	Não se aplica (Não tenho parceiro para responder)
Parceiro na mesma cama	Menos que 1 vez por semana	Não se aplica (Não tenho parceiro para responder)
Não	Não se aplica (Não tenho parceiro para responder)	Não se aplica (Não tenho parceiro para responder)
Parceiro na mesma cama	1 ou 2 vezes por semana	Menos que uma vez por semana
Não	Não se aplica (Não tenho parceiro para responder)	Não se aplica (Não tenho parceiro para responder)
Não	Não se aplica (Não tenho parceiro para responder)	Não se aplica (Não tenho parceiro para responder)
Não	Não se aplica (Não tenho parceiro para responder)	Nenhuma no último mês
Não	Não se aplica (Não tenho parceiro para responder)	Menos que uma vez por semana

Parceiro na mesma cama	Nenhuma no último mês	Nenhuma no último mês
Parceiro na mesma cama	Nenhuma no último mês	Nenhuma no último mês
Parceiro no mesmo quarto, mas não na mesma cama	Menos que 1 vez por semana	Não se aplica (Não tenho parceiro para responder)
Não	Não se aplica (Não tenho parceiro para responder)	Não se aplica (Não tenho parceiro para responder)
Não	Não se aplica (Não tenho parceiro para responder)	Não se aplica (Não tenho parceiro para responder)
Parceiro na mesma cama	Menos que 1 vez por semana	Nenhuma no último mês
Não	Não se aplica (Não tenho parceiro para responder)	Não se aplica (Não tenho parceiro para responder)
Não	Não se aplica (Não tenho parceiro para responder)	Não se aplica (Não tenho parceiro para responder)
Parceiro na mesma cama	Nenhuma no último mês	Não se aplica (Não tenho parceiro para responder)
Parceiro na mesma cama	3 ou mais vezes por semana	Nenhuma no último mês
Parceiro na mesma cama	3 ou mais vezes por semana	Menos que uma vez por semana
Parceiro na mesma cama	Nenhuma no último mês	Nenhuma no último mês

Parceiro no mesmo quarto, mas não na mesma cama	Nenhuma no último mês	Nenhuma no último mês
Parceiro na mesma cama	Nenhuma no último mês	Nenhuma no último mês
Parceiro na mesma cama	Nenhuma no último mês	Nenhuma no último mês
Parceiro na mesma cama	Nenhuma no último mês	Nenhuma no último mês
Parceiro na mesma cama	3 ou mais vezes por semana	Nenhuma no último mês
Parceiro na mesma cama	Nenhuma no último mês	Nenhuma no último mês
Parceiro na mesma cama	Nenhuma no último mês	Nenhuma no último mês
Não	Não se aplica (Não tenho parceiro para responder)	Não se aplica (Não tenho parceiro para responder)
Parceiro na mesma cama	Menos que 1 vez por semana	Nenhuma no último mês
Não	Não se aplica (Não tenho parceiro para responder)	Não se aplica (Não tenho parceiro para responder)
Não	Não se aplica (Não tenho parceiro para responder)	Não se aplica (Não tenho parceiro para responder)
Não	Não se aplica (Não tenho parceiro para responder)	Não se aplica (Não tenho parceiro para responder)

Não	1 ou 2 vezes por semana	Nenhuma no último mês
Parceiro na mesma cama	Menos que 1 vez por semana	Nenhuma no último mês
Parceiro na mesma cama	1 ou 2 vezes por semana	Nenhuma no último mês
Não	Não se aplica (Não tenho parceiro para responder)	Não se aplica (Não tenho parceiro para responder)
Não	Não se aplica (Não tenho parceiro para responder)	Não se aplica (Não tenho parceiro para responder)
Parceiro na mesma cama	Nenhuma no último mês	Nenhuma no último mês
Parceiro na mesma cama	1 ou 2 vezes por semana	Nenhuma no último mês
Parceiro na mesma cama	Nenhuma no último mês	Nenhuma no último mês
Parceiro na mesma cama	1 ou 2 vezes por semana	Menos que uma vez por semana
Parceiro na mesma cama	Nenhuma no último mês	Nenhuma no último mês
Parceiro na mesma cama	Nenhuma no último mês	Nenhuma no último mês

Parceiro na mesma cama	3 ou mais vezes por semana	3 ou mais vezes por semana
Parceiro na mesma cama	Nenhuma no último mês	Nenhuma no último mês
Não	Não se aplica (Não tenho parceiro para responder)	Não se aplica (Não tenho parceiro para responder)
Parceiro na mesma cama	Nenhuma no último mês	Nenhuma no último mês
Parceiro na mesma cama	Nenhuma no último mês	Nenhuma no último mês
Parceiro na mesma cama	1 ou 2 vezes por semana	Nenhuma no último mês
Parceiro na mesma cama	1 ou 2 vezes por semana	Nenhuma no último mês
Parceiro ou colega, mas em outro quarto	3 ou mais vezes por semana	Nenhuma no último mês
Parceiro na mesma cama	Nenhuma no último mês	Nenhuma no último mês
Parceiro na mesma cama	Nenhuma no último mês	Nenhuma no último mês
Parceiro na mesma cama	Menos que 1 vez por semana	Nenhuma no último mês

Parceiro na mesma cama	Nenhuma no último mês	Nenhuma no último mês
Parceiro na mesma cama	Nenhuma no último mês	Nenhuma no último mês
Parceiro na mesma cama	Nenhuma no último mês	Nenhuma no último mês
Parceiro na mesma cama	Nenhuma no último mês	Nenhuma no último mês
Não	Não se aplica (Não tenho parceiro para responder)	Não se aplica (Não tenho parceiro para responder)
Parceiro na mesma cama	Menos que 1 vez por semana	Nenhuma no último mês
Não	Não se aplica (Não tenho parceiro para responder)	Não se aplica (Não tenho parceiro para responder)
Parceiro na mesma cama	Nenhuma no último mês	Nenhuma no último mês
Parceiro na mesma cama	1 ou 2 vezes por semana	Não se aplica (Não tenho parceiro para responder)
Parceiro na mesma cama	Nenhuma no último mês	Nenhuma no último mês
Parceiro na mesma cama	Nenhuma no último mês	Nenhuma no último mês

Parceiro ou colega, mas em outro quarto	Nenhuma no último mês	Nenhuma no último mês
Parceiro na mesma cama	Menos que 1 vez por semana	Nenhuma no último mês
Não	Não se aplica (Não tenho parceiro para responder)	Não se aplica (Não tenho parceiro para responder)
Parceiro ou colega, mas em outro quarto	Não se aplica (Não tenho parceiro para responder)	Não se aplica (Não tenho parceiro para responder)
Não	Não se aplica (Não tenho parceiro para responder)	Não se aplica (Não tenho parceiro para responder)
Parceiro na mesma cama	Nenhuma no último mês	Nenhuma no último mês
Parceiro na mesma cama	Menos que 1 vez por semana	Nenhuma no último mês
Parceiro na mesma cama	Nenhuma no último mês	Nenhuma no último mês
Não	Não se aplica (Não tenho parceiro para responder)	Não se aplica (Não tenho parceiro para responder)
Parceiro na mesma cama	Nenhuma no último mês	Menos que uma vez por semana
Parceiro na mesma cama	Nenhuma no último mês	Nenhuma no último mês
Não	Não se aplica (Não tenho parceiro para responder)	Não se aplica (Não tenho parceiro para responder)

Parceiro na mesma cama	3 ou mais vezes por semana	Nenhuma no último mês
Parceiro na mesma cama	1 ou 2 vezes por semana	Menos que uma vez por semana
Parceiro no mesmo quarto, mas não na mesma cama	Menos que 1 vez por semana	Nenhuma no último mês
Parceiro na mesma cama	Nenhuma no último mês	Nenhuma no último mês
Parceiro na mesma cama	Menos que 1 vez por semana	Nenhuma no último mês
Não	Não se aplica (Não tenho parceiro para responder)	Não se aplica (Não tenho parceiro para responder)
Não	Não se aplica (Não tenho parceiro para responder)	Não se aplica (Não tenho parceiro para responder)
Parceiro na mesma cama	1 ou 2 vezes por semana	Menos que uma vez por semana
Parceiro na mesma cama	Menos que 1 vez por semana	Nenhuma no último mês
Não	Não se aplica (Não tenho parceiro para responder)	Não se aplica (Não tenho parceiro para responder)
Parceiro na mesma cama	Menos que 1 vez por semana	Nenhuma no último mês

Parceiro ou colega, mas em outro quarto	3 ou mais vezes por semana	3 ou mais vezes por semana
Não	Não se aplica (Não tenho parceiro para responder)	Não se aplica (Não tenho parceiro para responder)
Não	Não se aplica (Não tenho parceiro para responder)	Não se aplica (Não tenho parceiro para responder)
Parceiro ou colega, mas em outro quarto	Não se aplica (Não tenho parceiro para responder)	Não se aplica (Não tenho parceiro para responder)
Parceiro na mesma cama	Menos que 1 vez por semana	Nenhuma no último mês
Parceiro na mesma cama	Nenhuma no último mês	Nenhuma no último mês
Parceiro na mesma cama	1 ou 2 vezes por semana	Nenhuma no último mês
Parceiro na mesma cama	Nenhuma no último mês	Nenhuma no último mês
Parceiro na mesma cama	Nenhuma no último mês	Nenhuma no último mês
Parceiro na mesma cama	Menos que 1 vez por semana	Nenhuma no último mês

Parceiro na mesma cama	Nenhuma no último mês	Nenhuma no último mês
Parceiro na mesma cama	3 ou mais vezes por semana	Menos que uma vez por semana
Parceiro na mesma cama	Nenhuma no último mês	Nenhuma no último mês
Parceiro na mesma cama	Não se aplica (Não tenho parceiro para responder)	Não se aplica (Não tenho parceiro para responder)
Parceiro na mesma cama	1 ou 2 vezes por semana	Nenhuma no último mês
Não	Não se aplica (Não tenho parceiro para responder)	Não se aplica (Não tenho parceiro para responder)
Parceiro na mesma cama	Nenhuma no último mês	Nenhuma no último mês
Parceiro na mesma cama	3 ou mais vezes por semana	Nenhuma no último mês
Não	Não se aplica (Não tenho parceiro para responder)	Não se aplica (Não tenho parceiro para responder)
Parceiro na mesma cama	1 ou 2 vezes por semana	Nenhuma no último mês
Parceiro na mesma cama	Nenhuma no último mês	Nenhuma no último mês

Não	Não se aplica (Não tenho parceiro para responder)	Não se aplica (Não tenho parceiro para responder)
Parceiro na mesma cama	Nenhuma no último mês	Nenhuma no último mês
Parceiro no mesmo quarto, mas não na mesma cama	Nenhuma no último mês	Nenhuma no último mês
Parceiro na mesma cama	Menos que 1 vez por semana	Nenhuma no último mês
Não	Não se aplica (Não tenho parceiro para responder)	Não se aplica (Não tenho parceiro para responder)
Não	Não se aplica (Não tenho parceiro para responder)	Não se aplica (Não tenho parceiro para responder)
Parceiro na mesma cama	1 ou 2 vezes por semana	Não se aplica (Não tenho parceiro para responder)
Parceiro na mesma cama	Nenhuma no último mês	Nenhuma no último mês
Parceiro na mesma cama	Nenhuma no último mês	Nenhuma no último mês
Parceiro ou colega, mas em outro quarto	3 ou mais vezes por semana	Menos que uma vez por semana
Parceiro na mesma cama	Menos que 1 vez por semana	Menos que uma vez por semana

Parceiro na mesma cama	Nenhuma no último mês	Nenhuma no último mês
Não	Não se aplica (Não tenho parceiro para responder)	Não se aplica (Não tenho parceiro para responder)
Parceiro na mesma cama	Nenhuma no último mês	Nenhuma no último mês
Não	Não se aplica (Não tenho parceiro para responder)	Não se aplica (Não tenho parceiro para responder)
Parceiro na mesma cama	3 ou mais vezes por semana	Nenhuma no último mês
Parceiro na mesma cama	3 ou mais vezes por semana	3 ou mais vezes por semana
Parceiro na mesma cama	Nenhuma no último mês	Nenhuma no último mês
Parceiro na mesma cama	Nenhuma no último mês	Nenhuma no último mês
Não	Não se aplica (Não tenho parceiro para responder)	Não se aplica (Não tenho parceiro para responder)
Parceiro na mesma cama	Nenhuma no último mês	Nenhuma no último mês

Parceiro na mesma cama	Nenhuma no último mês	Nenhuma no último mês
Parceiro na mesma cama	3 ou mais vezes por semana	Nenhuma no último mês
Não	Não se aplica (Não tenho parceiro para responder)	Não se aplica (Não tenho parceiro para responder)
Parceiro na mesma cama	1 ou 2 vezes por semana	Nenhuma no último mês
Não	Não se aplica (Não tenho parceiro para responder)	Não se aplica (Não tenho parceiro para responder)
Parceiro na mesma cama	Nenhuma no último mês	Nenhuma no último mês
Não	Não se aplica (Não tenho parceiro para responder)	Não se aplica (Não tenho parceiro para responder)
Parceiro na mesma cama	Nenhuma no último mês	Nenhuma no último mês
Parceiro ou colega, mas em outro quarto	Nenhuma no último mês	Nenhuma no último mês
Parceiro na mesma cama	1 ou 2 vezes por semana	Nenhuma no último mês
Não	3 ou mais vezes por semana	3 ou mais vezes por semana
Não	Não se aplica (Não tenho parceiro para responder)	Nenhuma no último mês

Parceiro na mesma cama	Nenhuma no último mês	Nenhuma no último mês
Parceiro na mesma cama	1 ou 2 vezes por semana	Nenhuma no último mês
Parceiro na mesma cama	Nenhuma no último mês	Nenhuma no último mês
Não	Não se aplica (Não tenho parceiro para responder)	Não se aplica (Não tenho parceiro para responder)
Parceiro na mesma cama	Menos que 1 vez por semana	Menos que uma vez por semana
Parceiro na mesma cama	Nenhuma no último mês	Nenhuma no último mês
Parceiro na mesma cama	1 ou 2 vezes por semana	Nenhuma no último mês
Não	Não se aplica (Não tenho parceiro para responder)	Não se aplica (Não tenho parceiro para responder)
Parceiro na mesma cama	1 ou 2 vezes por semana	Nenhuma no último mês
Parceiro na mesma cama	3 ou mais vezes por semana	Nenhuma no último mês
Parceiro na mesma cama	Nenhuma no último mês	Nenhuma no último mês
Parceiro na mesma cama	3 ou mais vezes por semana	Nenhuma no último mês

Parceiro na mesma cama	3 ou mais vezes por semana	3 ou mais vezes por semana
Parceiro na mesma cama	Menos que 1 vez por semana	Nenhuma no último mês
Não	Não se aplica (Não tenho parceiro para responder)	Não se aplica (Não tenho parceiro para responder)
Parceiro na mesma cama	Nenhuma no último mês	Nenhuma no último mês
Parceiro na mesma cama	Nenhuma no último mês	Nenhuma no último mês
Parceiro na mesma cama	Menos que 1 vez por semana	Nenhuma no último mês
Parceiro na mesma cama	Menos que 1 vez por semana	Nenhuma no último mês
Parceiro na mesma cama	Nenhuma no último mês	Nenhuma no último mês
Não	Não se aplica (Não tenho parceiro para responder)	Não se aplica (Não tenho parceiro para responder)
Parceiro na mesma cama	Nenhuma no último mês	Nenhuma no último mês
Parceiro na mesma cama	Nenhuma no último mês	Nenhuma no último mês
Parceiro na mesma cama	3 ou mais vezes por semana	3 ou mais vezes por semana

Não	Não se aplica (Não tenho parceiro para responder)	Não se aplica (Não tenho parceiro para responder)
Parceiro na mesma cama	3 ou mais vezes por semana	Nenhuma no último mês
Parceiro na mesma cama	Nenhuma no último mês	Nenhuma no último mês
Não	Não se aplica (Não tenho parceiro para responder)	Não se aplica (Não tenho parceiro para responder)
Parceiro na mesma cama	Nenhuma no último mês	Nenhuma no último mês
Parceiro na mesma cama	Nenhuma no último mês	Nenhuma no último mês
Não	Não se aplica (Não tenho parceiro para responder)	Não se aplica (Não tenho parceiro para responder)
Parceiro na mesma cama	3 ou mais vezes por semana	Menos que uma vez por semana
Parceiro na mesma cama	1 ou 2 vezes por semana	Nenhuma no último mês
Parceiro na mesma cama	3 ou mais vezes por semana	1 ou 2 vezes por semana
Parceiro na mesma cama	Menos que 1 vez por semana	Nenhuma no último mês
Não	Não se aplica (Não tenho parceiro para responder)	Não se aplica (Não tenho parceiro para responder)

Não	Não se aplica (Não tenho parceiro para responder)	Não se aplica (Não tenho parceiro para responder)
Não	Nenhuma no último mês	Não se aplica (Não tenho parceiro para responder)
Parceiro na mesma cama	3 ou mais vezes por semana	3 ou mais vezes por semana
Não	Não se aplica (Não tenho parceiro para responder)	Não se aplica (Não tenho parceiro para responder)
Parceiro na mesma cama	Menos que 1 vez por semana	Nenhuma no último mês
Parceiro na mesma cama	3 ou mais vezes por semana	1 ou 2 vezes por semana
Parceiro na mesma cama	Nenhuma no último mês	Nenhuma no último mês
Parceiro na mesma cama	1 ou 2 vezes por semana	Nenhuma no último mês
Parceiro na mesma cama	1 ou 2 vezes por semana	Nenhuma no último mês
Parceiro na mesma cama	Nenhuma no último mês	Nenhuma no último mês
Parceiro na mesma cama	1 ou 2 vezes por semana	Menos que uma vez por semana
Parceiro na mesma cama	Nenhuma no último mês	Nenhuma no último mês

Parceiro na mesma cama	Nenhuma no último mês	Nenhuma no último mês
Parceiro na mesma cama	Nenhuma no último mês	Nenhuma no último mês
Não	Não se aplica (Não tenho parceiro para responder)	Não se aplica (Não tenho parceiro para responder)
Não	Não se aplica (Não tenho parceiro para responder)	Não se aplica (Não tenho parceiro para responder)
Parceiro na mesma cama	Menos que 1 vez por semana	Nenhuma no último mês

Contrações ou puxões de pernas enquanto você dormia?	Episódios de desorientação ou confusão durante o sono?	Outras alterações (inquietações) enquanto você dorme, por favor descreva.	na rio Ín di ce de Qu ali da de do
Nenhuma no último mês	Nenhuma no último mês		9
Não se aplica (Não tenho parceiro para responder)	Não se aplica (Não tenho parceiro para responder)		8
Nenhuma no último mês	Nenhuma no último mês		6
Não se aplica (Não tenho parceiro para responder)	Não se aplica (Não tenho parceiro para responder)		16
1 ou 2 vezes por semana	Nenhuma no último mês		6
Não se aplica (Não tenho parceiro para responder)	Não se aplica (Não tenho parceiro para responder)		6
Não se aplica (Não tenho parceiro para responder)	Não se aplica (Não tenho parceiro para responder)		8
Não se aplica (Não tenho parceiro para responder)	Não se aplica (Não tenho parceiro para responder)		7
1 ou 2 vezes por semana	Nenhuma no último mês	Falar enquanto dorme, de 1 a 2 vezes por semana	10

Não se aplica (Não tenho parceiro para responder)	Não se aplica (Não tenho parceiro para responder)		18
Não se aplica (Não tenho parceiro para responder)	Não se aplica (Não tenho parceiro para responder)		13
Menos que uma vez por semana	Nenhuma no último mês		8
3 ou mais vezes por semana	Nenhuma no último mês		5
Não se aplica (Não tenho parceiro para responder)	Não se aplica (Não tenho parceiro para responder)		7
Não se aplica (Não tenho parceiro para responder)	Não se aplica (Não tenho parceiro para responder)	Paralisia do Sono	11
Não se aplica (Não tenho parceiro para responder)	Não se aplica (Não tenho parceiro para responder)		6
Nenhuma no último mês	Nenhuma no último mês	Acordo frequentemente com barulhos	6
Menos que uma vez por semana	Nenhuma no último mês		5
Não se aplica (Não tenho parceiro para responder)	Não se aplica (Não tenho parceiro para responder)		7
Nenhuma no último mês	Nenhuma no último mês		0

Nenhuma no último mês	Nenhuma no último mês		6
Não se aplica (Não tenho parceiro para responder)	Não se aplica (Não tenho parceiro para responder)	Desde criança sofro de sonambulismo (falando e andando durante o sono). Ocasionalmente minha família comenta sobre eu estar falando (mas não andando) no sono durante os últimos meses.	8
Nenhuma no último mês	Menos que 1 vez por semana		6
1 ou 2 vezes por semana	Nenhuma no último mês	Neste último mês meu sono está muito melhor, mas posso dizer que tempos atrás estava sofrendo muito com "síndrome das pernas inquietas"	5
1 ou 2 vezes por semana	1 ou 2 vezes por semana		18
Não se aplica (Não tenho parceiro para responder)	Não se aplica (Não tenho parceiro para responder)		6
Nenhuma no último mês	Nenhuma no último mês		11
Nenhuma no último mês	Nenhuma no último mês		12
Nenhuma no último mês	Nenhuma no último mês		9
Menos que uma vez por semana	Nenhuma no último mês		9

Nenhuma no último mês	Nenhuma no último mês	6
Não se aplica (Não tenho parceiro para responder)	Não se aplica (Não tenho parceiro para responder)	8
Não se aplica (Não tenho parceiro para responder)	Não se aplica (Não tenho parceiro para responder)	7
Não se aplica (Não tenho parceiro para responder)	Não se aplica (Não tenho parceiro para responder)	8
Nenhuma no último mês	Nenhuma no último mês	5
1 ou 2 vezes por semana	Menos que 1 vez por semana	5
Menos que uma vez por semana	1 ou 2 vezes por semana	13
Não se aplica (Não tenho parceiro para responder)	Não se aplica (Não tenho parceiro para responder)	10
Não se aplica (Não tenho parceiro para responder)	Não se aplica (Não tenho parceiro para responder)	7
Não se aplica (Não tenho parceiro para responder)	Não se aplica (Não tenho parceiro para responder)	8
Nenhuma no último mês	Nenhuma no último mês	8
Não se aplica (Não tenho parceiro para responder)	Não se aplica (Não tenho parceiro para responder)	6

1 ou 2 vezes por semana	Nenhuma no último mês		6
Menos que uma vez por semana	Menos que 1 vez por semana		8
Menos que uma vez por semana	1 ou 2 vezes por semana	Me mexo muito dormindo e cheguei a chutar a perna da minha esposa quando acrodei assustado por algum motivo. Lembro-me disso e de ter me desculpado com ela imediatamente. Voltei a dormir cerca de 20 minutos depois.	12
3 ou mais vezes por semana	Nenhuma no último mês		7
Não se aplica (Não tenho parceiro para responder)	Não se aplica (Não tenho parceiro para responder)		7
Não se aplica (Não tenho parceiro para responder)	Não se aplica (Não tenho parceiro para responder)		9
Não se aplica (Não tenho parceiro para responder)	Não se aplica (Não tenho parceiro para responder)		9
Não se aplica (Não tenho parceiro para responder)	Não se aplica (Não tenho parceiro para responder)		10
Não se aplica (Não tenho parceiro para responder)	Não se aplica (Não tenho parceiro para responder)		8
Não se aplica (Não tenho parceiro para responder)	Não se aplica (Não tenho parceiro para responder)		6

Nenhuma no último mês	Menos que 1 vez por semana		16
3 ou mais vezes por semana	1 ou 2 vezes por semana		8
Não se aplica (Não tenho parceiro para responder)	Não se aplica (Não tenho parceiro para responder)		6
3 ou mais vezes por semana	Nenhuma no último mês	.	12
Não se aplica (Não tenho parceiro para responder)	Não se aplica (Não tenho parceiro para responder)		9
Menos que uma vez por semana	Nenhuma no último mês	Senti a boca seca, me levantava para tomar água.	11
Não se aplica (Não tenho parceiro para responder)	Não se aplica (Não tenho parceiro para responder)		10
Não se aplica (Não tenho parceiro para responder)	Não se aplica (Não tenho parceiro para responder)		7
1 ou 2 vezes por semana	Nenhuma no último mês		4
Não se aplica (Não tenho parceiro para responder)	Não se aplica (Não tenho parceiro para responder)		8
3 ou mais vezes por semana	3 ou mais vezes por semana		2

Não se aplica (Não tenho parceiro para responder)	Não se aplica (Não tenho parceiro para responder)		8
1 ou 2 vezes por semana	Nenhuma no último mês		2
Nenhuma no último mês	Menos que 1 vez por semana		3
3 ou mais vezes por semana	Menos que 1 vez por semana		9
Nenhuma no último mês	Menos que 1 vez por semana		3
Nenhuma no último mês	Nenhuma no último mês		5
Nenhuma no último mês	Nenhuma no último mês	Nenhuma.	1
Não se aplica (Não tenho parceiro para responder)	Não se aplica (Não tenho parceiro para responder)		4
3 ou mais vezes por semana	Nenhuma no último mês	Perna dobrada para cima no meio da noite	8
Menos que uma vez por semana	Nenhuma no último mês		3
1 ou 2 vezes por semana	Não se aplica (Não tenho parceiro para responder)		9

Nenhuma no último mês	Nenhuma no último mês		12
Menos que uma vez por semana	Nenhuma no último mês		8
Nenhuma no último mês	Nenhuma no último mês		3
Não se aplica (Não tenho parceiro para responder)	1 ou 2 vezes por semana	Eu costumo acordar sem saber aonde estou e em que dia estou, costuma passar em segundos.	3
Nenhuma no último mês	Nenhuma no último mês		5
Não se aplica (Não tenho parceiro para responder)	Não se aplica (Não tenho parceiro para responder)		14
Não se aplica (Não tenho parceiro para responder)	Não se aplica (Não tenho parceiro para responder)		7
Menos que uma vez por semana	Nenhuma no último mês		4
Não se aplica (Não tenho parceiro para responder)	Não se aplica (Não tenho parceiro para responder)		8
Menos que uma vez por semana	Nenhuma no último mês		8
Não se aplica (Não tenho parceiro para responder)	Não se aplica (Não tenho parceiro para responder)		9
Nenhuma no último mês	Menos que 1 vez por semana		11

Nenhuma no último mês	Nenhuma no último mês	1
Não se aplica (Não tenho parceiro para responder)	Não se aplica (Não tenho parceiro para responder)	6
Não se aplica (Não tenho parceiro para responder)	Não se aplica (Não tenho parceiro para responder)	4
Menos que uma vez por semana	Nenhuma no último mês	6
Menos que uma vez por semana	Menos que 1 vez por semana	5
Não se aplica (Não tenho parceiro para responder)	Não se aplica (Não tenho parceiro para responder)	8
Não se aplica (Não tenho parceiro para responder)	Não se aplica (Não tenho parceiro para responder)	5
Não se aplica (Não tenho parceiro para responder)	Não se aplica (Não tenho parceiro para responder)	17
Não se aplica (Não tenho parceiro para responder)	Não se aplica (Não tenho parceiro para responder)	7
3 ou mais vezes por semana	Nenhuma no último mês	12
Não se aplica (Não tenho parceiro para responder)	Não se aplica (Não tenho parceiro para responder)	7
Não se aplica (Não tenho parceiro para responder)	Não se aplica (Não tenho parceiro para responder)	12

Nenhuma no último mês	Nenhuma no último mês	6
3 ou mais vezes por semana	Menos que 1 vez por semana	14
Menos que uma vez por semana	Não se aplica (Não tenho parceiro para responder)	6
Menos que uma vez por semana	Nenhuma no último mês	6
1 ou 2 vezes por semana	Nenhuma no último mês	3
Não se aplica (Não tenho parceiro para responder)	Não se aplica (Não tenho parceiro para responder)	5
1 ou 2 vezes por semana	Nenhuma no último mês	9
3 ou mais vezes por semana	Nenhuma no último mês	8
Não se aplica (Não tenho parceiro para responder)	Não se aplica (Não tenho parceiro para responder)	7
Nenhuma no último mês	Menos que 1 vez por semana	11
Não se aplica (Não tenho parceiro para responder)	Não se aplica (Não tenho parceiro para responder)	8

3 ou mais vezes por semana	1 ou 2 vezes por semana	15
3 ou mais vezes por semana	Nenhuma no último mês	11
Nenhuma no último mês	Nenhuma no último mês	16
Nenhuma no último mês	Nenhuma no último mês	6
Menos que uma vez por semana	Nenhuma no último mês	8
Não se aplica (Não tenho parceiro para responder)	Não se aplica (Não tenho parceiro para responder)	12
Não se aplica (Não tenho parceiro para responder)	Não se aplica (Não tenho parceiro para responder)	13
Não se aplica (Não tenho parceiro para responder)	Não se aplica (Não tenho parceiro para responder)	6
Nenhuma no último mês	Nenhuma no último mês	1
Menos que uma vez por semana	Nenhuma no último mês	5

Não se aplica (Não tenho parceiro para responder)	Não se aplica (Não tenho parceiro para responder)		4
Não se aplica (Não tenho parceiro para responder)	Não se aplica (Não tenho parceiro para responder)		9
Não se aplica (Não tenho parceiro para responder)	Não se aplica (Não tenho parceiro para responder)		5
Não se aplica (Não tenho parceiro para responder)	Não se aplica (Não tenho parceiro para responder)		6
Nenhuma no último mês	Nenhuma no último mês		6
Nenhuma no último mês	Nenhuma no último mês		5
Menos que uma vez por semana	Nenhuma no último mês	Acordei com a perna doendo bastante .	10
Nenhuma no último mês	Nenhuma no último mês		8
Nenhuma no último mês	Nenhuma no último mês		3
Não se aplica (Não tenho parceiro para responder)	Não se aplica (Não tenho parceiro para responder)		8
Nenhuma no último mês	Nenhuma no último mês		5

Não se aplica (Não tenho parceiro para responder)	Não se aplica (Não tenho parceiro para responder)		10
1 ou 2 vezes por semana	Menos que 1 vez por semana		10
3 ou mais vezes por semana	3 ou mais vezes por semana		11
Nenhuma no último mês	Não se aplica (Não tenho parceiro para responder)		8
Não se aplica (Não tenho parceiro para responder)	Nenhuma no último mês	Nao houve	4
Não se aplica (Não tenho parceiro para responder)	Não se aplica (Não tenho parceiro para responder)		7
3 ou mais vezes por semana	Nenhuma no último mês		6
Não se aplica (Não tenho parceiro para responder)	Não se aplica (Não tenho parceiro para responder)		8
Não se aplica (Não tenho parceiro para responder)	Não se aplica (Não tenho parceiro para responder)		10
Nenhuma no último mês	Nenhuma no último mês		9
Não se aplica (Não tenho parceiro para responder)	Não se aplica (Não tenho parceiro para responder)		8

Nenhuma no último mês	Nenhuma no último mês	Nenhuma	4
3 ou mais vezes por semana	1 ou 2 vezes por semana	Falar	7
1 ou 2 vezes por semana	3 ou mais vezes por semana		15
Não se aplica (Não tenho parceiro para responder)	Não se aplica (Não tenho parceiro para responder)		4
Não se aplica (Não tenho parceiro para responder)	Não se aplica (Não tenho parceiro para responder)		5
Nenhuma no último mês	Nenhuma no último mês		9
Não se aplica (Não tenho parceiro para responder)	Não se aplica (Não tenho parceiro para responder)		5
Não se aplica (Não tenho parceiro para responder)	Não se aplica (Não tenho parceiro para responder)		5
Não se aplica (Não tenho parceiro para responder)	Não se aplica (Não tenho parceiro para responder)		7
Nenhuma no último mês	Nenhuma no último mês		6
Nenhuma no último mês	Nenhuma no último mês		4
Menos que uma vez por semana	Nenhuma no último mês		7

Nenhuma no último mês	Nenhuma no último mês	2
Nenhuma no último mês	Nenhuma no último mês	6
3 ou mais vezes por semana	Menos que 1 vez por semana	6
Menos que uma vez por semana	Nenhuma no último mês	5
Nenhuma no último mês	Nenhuma no último mês	4
Menos que uma vez por semana	Nenhuma no último mês	5
Nenhuma no último mês	Menos que 1 vez por semana	9
Não se aplica (Não tenho parceiro para responder)	Não se aplica (Não tenho parceiro para responder)	13
Nenhuma no último mês	Menos que 1 vez por semana	5
Não se aplica (Não tenho parceiro para responder)	Não se aplica (Não tenho parceiro para responder)	10
Não se aplica (Não tenho parceiro para responder)	Não se aplica (Não tenho parceiro para responder)	12
Não se aplica (Não tenho parceiro para responder)	Não se aplica (Não tenho parceiro para responder)	3

Nenhuma no último mês	Nenhuma no último mês	nenhuma	7
Nenhuma no último mês	Nenhuma no último mês		6
Menos que uma vez por semana	Nenhuma no último mês		14
Não se aplica (Não tenho parceiro para responder)	Não se aplica (Não tenho parceiro para responder)		14
Não se aplica (Não tenho parceiro para responder)	Não se aplica (Não tenho parceiro para responder)		9
Menos que uma vez por semana	Nenhuma no último mês		4
Nenhuma no último mês	Nenhuma no último mês		3
Menos que uma vez por semana	Menos que 1 vez por semana		4
3 ou mais vezes por semana	Nenhuma no último mês		9
Menos que uma vez por semana	3 ou mais vezes por semana		4
1 ou 2 vezes por semana	Nenhuma no último mês		6

Nenhuma no último mês	Nenhuma no último mês	90 microdespertares e 34 painéis do sono	14
Nenhuma no último mês	Nenhuma no último mês	Sempre tive bruxismo. Atualmente utilizo a plaquinha acrílica para este problema.	5
Não se aplica (Não tenho parceiro para responder)	Não se aplica (Não tenho parceiro para responder)		1
Nenhuma no último mês	1 ou 2 vezes por semana		5
Menos que uma vez por semana	Nenhuma no último mês		11
3 ou mais vezes por semana	3 ou mais vezes por semana		15
Menos que uma vez por semana	Nenhuma no último mês		7
Menos que uma vez por semana	Nenhuma no último mês		4
Nenhuma no último mês	Nenhuma no último mês		6
Nenhuma no último mês	Menos que 1 vez por semana		5
1 ou 2 vezes por semana	Menos que 1 vez por semana		6

Menos que uma vez por semana	Nenhuma no último mês		11
Menos que uma vez por semana	Nenhuma no último mês		3
3 ou mais vezes por semana	Nenhuma no último mês		9
Menos que uma vez por semana	Nenhuma no último mês	Gatos com frequência (2-3 vezes por semana) me acordam antes das 6 pedindo comida, mas volto a deitar em seguida. As vezes o sono demora a voltar ou nem volta.	7
Não se aplica (Não tenho parceiro para responder)	Não se aplica (Não tenho parceiro para responder)		8
Nenhuma no último mês	Nenhuma no último mês		4
Não se aplica (Não tenho parceiro para responder)	Não se aplica (Não tenho parceiro para responder)	As minhas cachorras latem de vez em quando.	10
Nenhuma no último mês	Nenhuma no último mês		0
Não se aplica (Não tenho parceiro para responder)	Não se aplica (Não tenho parceiro para responder)		6
1 ou 2 vezes por semana	Nenhuma no último mês		12
Menos que uma vez por semana	Nenhuma no último mês		8

Nenhuma no último mês	Nenhuma no último mês		10
Menos que uma vez por semana	Nenhuma no último mês		7
Menos que uma vez por semana	1 ou 2 vezes por semana	As vezes tenho ataques de pânico ao acordar subitamente a noite. Durante a pandemia esse quadro se intensificou.	9
Não se aplica (Não tenho parceiro para responder)	Não se aplica (Não tenho parceiro para responder)		10
Não se aplica (Não tenho parceiro para responder)	Não se aplica (Não tenho parceiro para responder)		5
Nenhuma no último mês	Nenhuma no último mês		13
Menos que uma vez por semana	Nenhuma no último mês		4
Nenhuma no último mês	Menos que 1 vez por semana	Tonturas	12
Não se aplica (Não tenho parceiro para responder)	Não se aplica (Não tenho parceiro para responder)	Preocupações e ansiedade	11
Menos que uma vez por semana	Nenhuma no último mês		16
Nenhuma no último mês	Nenhuma no último mês		3
Não se aplica (Não tenho parceiro para responder)	Não se aplica (Não tenho parceiro para responder)		9

Nenhuma no último mês	Nenhuma no último mês		5
Menos que uma vez por semana	Nenhuma no último mês		10
Menos que uma vez por semana	Nenhuma no último mês		6
Menos que uma vez por semana	Nenhuma no último mês		6
Nenhuma no último mês	Nenhuma no último mês		5
Menos que uma vez por semana	Não se aplica (Não tenho parceiro para responder)		4
Não se aplica (Não tenho parceiro para responder)	Não se aplica (Não tenho parceiro para responder)	Terror noturno	13
1 ou 2 vezes por semana	Nenhuma no último mês		8
1 ou 2 vezes por semana	Nenhuma no último mês		11
Não se aplica (Não tenho parceiro para responder)	Não se aplica (Não tenho parceiro para responder)	Tenho eu tido dificuldade para dormir após almoço, sempre dormia bem, de algum tempo pra cá, não consigo mais (apenas no horário do almoço)	7
Nenhuma no último mês	Nenhuma no último mês		8

Nenhuma no último mês	Nenhuma no último mês		6
Não se aplica (Não tenho parceiro para responder)	Não se aplica (Não tenho parceiro para responder)		18
Não se aplica (Não tenho parceiro para responder)	Não se aplica (Não tenho parceiro para responder)		11
Não se aplica (Não tenho parceiro para responder)	Não se aplica (Não tenho parceiro para responder)	Attualmente nenhuma.	9
Nenhuma no último mês	Nenhuma no último mês		8
Nenhuma no último mês	Nenhuma no último mês		5
Nenhuma no último mês	Menos que 1 vez por semana		6
Menos que uma vez por semana	Nenhuma no último mês		6
Menos que uma vez por semana	Nenhuma no último mês		9
Menos que uma vez por semana	Nenhuma no último mês		14

1 ou 2 vezes por semana	Nenhuma no último mês		13
Nenhuma no último mês	Menos que 1 vez por semana		9
Menos que uma vez por semana	1 ou 2 vezes por semana	Sonambulismo	5
1 ou 2 vezes por semana	1 ou 2 vezes por semana		9
Nenhuma no último mês	Menos que 1 vez por semana		3
Não se aplica (Não tenho parceiro para responder)	Não se aplica (Não tenho parceiro para responder)		4
Nenhuma no último mês	Nenhuma no último mês		6
Nenhuma no último mês	Nenhuma no último mês		7
Não se aplica (Não tenho parceiro para responder)	Não se aplica (Não tenho parceiro para responder)		7
Menos que uma vez por semana	Nenhuma no último mês	acordar várias vezes à noite	14
Nenhuma no último mês	Nenhuma no último mês		4

Não se aplica (Não tenho parceiro para responder)	Não se aplica (Não tenho parceiro para responder)	nada	4
Nenhuma no último mês	Nenhuma no último mês		4
Nenhuma no último mês	Nenhuma no último mês		11
Nenhuma no último mês	Nenhuma no último mês		11
Não se aplica (Não tenho parceiro para responder)	Não se aplica (Não tenho parceiro para responder)		9
Menos que uma vez por semana	Menos que 1 vez por semana	vertigem	9
Menos que uma vez por semana	Nenhuma no último mês		11
Nenhuma no último mês	Nenhuma no último mês		7
Menos que uma vez por semana	Nenhuma no último mês		1
Nenhuma no último mês	Não se aplica (Não tenho parceiro para responder)		11
Nenhuma no último mês	1 ou 2 vezes por semana		20

1 ou 2 vezes por semana	Nenhuma no último mês		10
Não se aplica (Não tenho parceiro para responder)	Não se aplica (Não tenho parceiro para responder)		12
Menos que uma vez por semana	Nenhuma no último mês	Me mexo bastante durante o sono. Também falo dormindo ocasionalmente	4
Não se aplica (Não tenho parceiro para responder)	Não se aplica (Não tenho parceiro para responder)		15
Nenhuma no último mês	Nenhuma no último mês		10
1 ou 2 vezes por semana	Nenhuma no último mês		10
Nenhuma no último mês	Nenhuma no último mês		9
1 ou 2 vezes por semana	Nenhuma no último mês	Medo, ansiedade	9
Não se aplica (Não tenho parceiro para responder)	Não se aplica (Não tenho parceiro para responder)		13
Nenhuma no último mês	Menos que 1 vez por semana		11

Nenhuma no último mês	Nenhuma no último mês	Nenhuma	1
Nenhuma no último mês	Menos que 1 vez por semana		8
Não se aplica (Não tenho parceiro para responder)	Não se aplica (Não tenho parceiro para responder)		6
Nenhuma no último mês	Nenhuma no último mês		11
Menos que uma vez por semana	Não se aplica (Não tenho parceiro para responder)		15
Nenhuma no último mês	Nenhuma no último mês		4
Não se aplica (Não tenho parceiro para responder)	Não se aplica (Não tenho parceiro para responder)		12
Nenhuma no último mês	Nenhuma no último mês		4
Nenhuma no último mês	Nenhuma no último mês		5
Nenhuma no último mês	Nenhuma no último mês		3
1 ou 2 vezes por semana	Nenhuma no último mês		7
Menos que uma vez por semana	Nenhuma no último mês		7

Nenhuma no último mês	Nenhuma no último mês	Devido a dor no quadril me viro muito buscando conforto.	8
Nenhuma no último mês	Nenhuma no último mês		9
Nenhuma no último mês	Nenhuma no último mês		5
Não se aplica (Não tenho parceiro para responder)	Não se aplica (Não tenho parceiro para responder)		6
Nenhuma no último mês	Nenhuma no último mês		11
1 ou 2 vezes por semana	Nenhuma no último mês		10
Nenhuma no último mês	Menos que 1 vez por semana		9
Não se aplica (Não tenho parceiro para responder)	Não se aplica (Não tenho parceiro para responder)		5
Nenhuma no último mês	Menos que 1 vez por semana		5
Nenhuma no último mês	Nenhuma no último mês		5
Menos que uma vez por semana	Nenhuma no último mês		9
Nenhuma no último mês	Nenhuma no último mês		9

Nenhuma no último mês	Nenhuma no último mês		9
Nenhuma no último mês	Nenhuma no último mês		8
Não se aplica (Não tenho parceiro para responder)	Não se aplica (Não tenho parceiro para responder)	n/a	7
Nenhuma no último mês	Nenhuma no último mês		2
Menos que uma vez por semana	Nenhuma no último mês		3
Nenhuma no último mês	Nenhuma no último mês	Resmungos	8
Menos que uma vez por semana	Nenhuma no último mês		12
1 ou 2 vezes por semana	Nenhuma no último mês		5
Não se aplica (Não tenho parceiro para responder)	Nenhuma no último mês		5
Nenhuma no último mês	Nenhuma no último mês		8
Nenhuma no último mês	Nenhuma no último mês	Nenhuma	5
Nenhuma no último mês	Nenhuma no último mês		7

Não se aplica (Não tenho parceiro para responder)	Não se aplica (Não tenho parceiro para responder)		2
Nenhuma no último mês	Nenhuma no último mês	SOU INQUIETO ENQUANTO DURMO	5
3 ou mais vezes por semana	Nenhuma no último mês		9
Não se aplica (Não tenho parceiro para responder)	Não se aplica (Não tenho parceiro para responder)		12
Nenhuma no último mês	Nenhuma no último mês		5
Não se aplica (Não tenho parceiro para responder)	Não se aplica (Não tenho parceiro para responder)		3
Não se aplica (Não tenho parceiro para responder)	Não se aplica (Não tenho parceiro para responder)		4
Nenhuma no último mês	Não se aplica (Não tenho parceiro para responder)		16
Nenhuma no último mês	Nenhuma no último mês		13
1 ou 2 vezes por semana	1 ou 2 vezes por semana		8
Menos que uma vez por semana	Nenhuma no último mês		10
Não se aplica (Não tenho parceiro para responder)	Não se aplica (Não tenho parceiro para responder)		13

Não se aplica (Não tenho parceiro para responder)	Não se aplica (Não tenho parceiro para responder)		13
Nenhuma no último mês	Nenhuma no último mês		6
Nenhuma no último mês	Nenhuma no último mês		3
Não se aplica (Não tenho parceiro para responder)	Não se aplica (Não tenho parceiro para responder)		4
Nenhuma no último mês	Nenhuma no último mês	Na resposta acima medicamento para dormi e, eu tomo chá (desinchá noite)	5
Menos que uma vez por semana	Nenhuma no último mês	Nenhum	4
1 ou 2 vezes por semana	Menos que 1 vez por semana		7
Menos que uma vez por semana	Menos que 1 vez por semana	O companheiro chuta a noite td (perna inquieta)	6
Nenhuma no último mês	Nenhuma no último mês	não	2
Nenhuma no último mês	Nenhuma no último mês		4
Nenhuma no último mês	Não se aplica (Não tenho parceiro para responder)	Apago	3
Nenhuma no último mês	Nenhuma no último mês		6

Nenhuma no último mês	Nenhuma no último mês	12
Nenhuma no último mês	Nenhuma no último mês	8
Não se aplica (Não tenho parceiro para responder)	Não se aplica (Não tenho parceiro para responder)	6
Não se aplica (Não tenho parceiro para responder)	Não se aplica (Não tenho parceiro para responder)	9
Nenhuma no último mês	Nenhuma no último mês	3

Qualidade do sono sedundo o Questionário Índice de Qualidade do Sono de Pittsburgh (PSQI)	Sentir-se nervoso(a), ansioso(a) ou muito tenso(a)	Não ser capaz de impedir ou de controlar as preocupações
ruim	Quase todos os dias	Quase todos os dias
ruim	Vários dias	Vários dias
ruim	Quase todos os dias	Mais da metade dos dias
presença de distúrbios do sono	Vários dias	Vários dias
ruim	Vários dias	Vários dias
ruim	Vários dias	Vários dias
ruim	Vários dias	Vários dias
ruim	Vários dias	Vários dias
ruim	Vários dias	Vários dias

presença de distúrbios do sono	Vários dias	Vários dias
presença de distúrbios do sono	Quase todos os dias	Mais da metade dos dias
ruim	Vários dias	Vários dias
ruim	Vários dias	Vários dias
ruim	Vários dias	Nenhuma vez
presença de distúrbios do sono	Quase todos os dias	Quase todos os dias
ruim	Mais da metade dos dias	Vários dias
ruim	Vários dias	Vários dias
ruim	Quase todos os dias	Quase todos os dias
ruim	Vários dias	Vários dias
boa	Nenhuma vez	Nenhuma vez

ruim	Vários dias	Vários dias
ruim	Vários dias	Vários dias
ruim	Vários dias	Vários dias
ruim	Quase todos os dias	Mais da metade dos dias
presença de distúrbios do sono	Quase todos os dias	Quase todos os dias
ruim	Vários dias	Vários dias
presença de distúrbios do sono	Mais da metade dos dias	Mais da metade dos dias
presença de distúrbios do sono	Quase todos os dias	Vários dias
ruim	Vários dias	Mais da metade dos dias
ruim	Vários dias	Mais da metade dos dias

ruim	Mais da metade dos dias	Vários dias
ruim	Vários dias	Mais da metade dos dias
ruim	Mais da metade dos dias	Vários dias
ruim	Quase todos os dias	Quase todos os dias
ruim	Vários dias	Vários dias
ruim	Vários dias	Nenhuma vez
presença de distúrbios do sono	Mais da metade dos dias	Mais da metade dos dias
ruim	Quase todos os dias	Quase todos os dias
ruim	Quase todos os dias	Quase todos os dias
ruim	Vários dias	Vários dias
ruim	Mais da metade dos dias	Vários dias
ruim	Vários dias	Vários dias

ruim	Quase todos os dias	Quase todos os dias
ruim	Vários dias	Mais da metade dos dias
presença de distúrbios do sono	Vários dias	Vários dias
ruim	Mais da metade dos dias	Quase todos os dias
ruim	Vários dias	Vários dias
ruim	Vários dias	Vários dias
ruim	Vários dias	Nenhuma vez
ruim	Mais da metade dos dias	Mais da metade dos dias
ruim	Vários dias	Vários dias
ruim	Vários dias	Nenhuma vez

presença de distúrbios do sono	Quase todos os dias	Mais da metade dos dias
ruim	Quase todos os dias	Quase todos os dias
ruim	Vários dias	Vários dias
presença de distúrbios do sono	Quase todos os dias	Vários dias
ruim	Quase todos os dias	Vários dias
presença de distúrbios do sono	Quase todos os dias	Mais da metade dos dias
ruim	Quase todos os dias	Vários dias
ruim	Vários dias	Vários dias
boa	Nenhuma vez	Vários dias
ruim	Vários dias	Vários dias
boa	Nenhuma vez	Nenhuma vez

ruim	Vários dias	Vários dias
boa	Vários dias	Vários dias
boa	Vários dias	Vários dias
ruim	Quase todos os dias	Mais da metade dos dias
boa	Mais da metade dos dias	Mais da metade dos dias
ruim	Mais da metade dos dias	Mais da metade dos dias
boa	Vários dias	Nenhuma vez
boa	Nenhuma vez	Vários dias
ruim	Vários dias	Vários dias
boa	Vários dias	Vários dias
ruim	Vários dias	Nenhuma vez

presença de distúrbios do sono	Vários dias	Quase todos os dias
ruim	Vários dias	Vários dias
boa	Nenhuma vez	Nenhuma vez
boa	Nenhuma vez	Nenhuma vez
ruim	Quase todos os dias	Mais da metade dos dias
presença de distúrbios do sono	Quase todos os dias	Mais da metade dos dias
ruim	Vários dias	Vários dias
boa	Nenhuma vez	Nenhuma vez
ruim	Quase todos os dias	Quase todos os dias
ruim	Vários dias	Vários dias
ruim	Quase todos os dias	Mais da metade dos dias
presença de distúrbios do sono	Quase todos os dias	Quase todos os dias

boa	Vários dias	Nenhuma vez
ruim	Vários dias	Nenhuma vez
boa	Vários dias	Vários dias
ruim	Nenhuma vez	Nenhuma vez
ruim	Quase todos os dias	Quase todos os dias
ruim	Mais da metade dos dias	Mais da metade dos dias
ruim	Vários dias	Vários dias
presença de distúrbios do sono	Quase todos os dias	Mais da metade dos dias
ruim	Vários dias	Vários dias
presença de distúrbios do sono	Mais da metade dos dias	Mais da metade dos dias
ruim	Vários dias	Mais da metade dos dias
presença de distúrbios do sono	Quase todos os dias	Mais da metade dos dias

ruim	Vários dias	Vários dias
presença de distúrbios do sono	Vários dias	Nenhuma vez
ruim	Quase todos os dias	Quase todos os dias
ruim	Vários dias	Vários dias
boa	Vários dias	Nenhuma vez
ruim	Mais da metade dos dias	Mais da metade dos dias
ruim	Quase todos os dias	Mais da metade dos dias
ruim	Vários dias	Mais da metade dos dias
ruim	Quase todos os dias	Quase todos os dias
presença de distúrbios do sono	Quase todos os dias	Quase todos os dias
ruim	Vários dias	Vários dias

presença de distúrbios do sono	Quase todos os dias	Quase todos os dias
presença de distúrbios do sono	Quase todos os dias	Quase todos os dias
presença de distúrbios do sono	Quase todos os dias	Quase todos os dias
ruim	Vários dias	Vários dias
ruim	Quase todos os dias	Quase todos os dias
presença de distúrbios do sono	Vários dias	Vários dias
presença de distúrbios do sono	Quase todos os dias	Quase todos os dias
ruim	Mais da metade dos dias	Mais da metade dos dias
boa	Nenhuma vez	Nenhuma vez
ruim	Vários dias	Vários dias

boa	Vários dias	Nenhuma vez
ruim	Mais da metade dos dias	Vários dias
ruim	Vários dias	Vários dias
ruim	Mais da metade dos dias	Vários dias
ruim	Vários dias	Vários dias
ruim	Vários dias	Vários dias
ruim	Vários dias	Vários dias
ruim	Vários dias	Vários dias
boa	Vários dias	Nenhuma vez
ruim	Quase todos os dias	Mais da metade dos dias
ruim	Nenhuma vez	Nenhuma vez

ruim	Mais da metade dos dias	Mais da metade dos dias
ruim	Mais da metade dos dias	Mais da metade dos dias
presença de distúrbios do sono	Quase todos os dias	Quase todos os dias
ruim	Vários dias	Vários dias
boa	Vários dias	Nenhuma vez
ruim	Vários dias	Nenhuma vez
ruim	Quase todos os dias	Quase todos os dias
ruim	Vários dias	Vários dias
ruim	Quase todos os dias	Quase todos os dias
ruim	Quase todos os dias	Vários dias
ruim	Quase todos os dias	Quase todos os dias

boa	Vários dias	Nenhuma vez
ruim	Quase todos os dias	Mais da metade dos dias
presença de distúrbios do sono	Mais da metade dos dias	Vários dias
boa	Nenhuma vez	Nenhuma vez
ruim	Mais da metade dos dias	Mais da metade dos dias
ruim	Vários dias	Vários dias
ruim	Nenhuma vez	Nenhuma vez
ruim	Vários dias	Nenhuma vez
ruim	Mais da metade dos dias	Vários dias
ruim	Mais da metade dos dias	Mais da metade dos dias
boa	Vários dias	Vários dias
ruim	Vários dias	Vários dias

boa	Vários dias	Nenhuma vez
ruim	Mais da metade dos dias	Mais da metade dos dias
ruim	Vários dias	Mais da metade dos dias
ruim	Vários dias	Nenhuma vez
boa	Mais da metade dos dias	Vários dias
ruim	Mais da metade dos dias	Mais da metade dos dias
ruim	Mais da metade dos dias	Vários dias
presença de distúrbios do sono	Quase todos os dias	Mais da metade dos dias
ruim	Quase todos os dias	Quase todos os dias
ruim	Quase todos os dias	Quase todos os dias
presença de distúrbios do sono	Mais da metade dos dias	Vários dias
boa	Nenhuma vez	Vários dias

ruim	Vários dias	Nenhuma vez
ruim	Vários dias	Vários dias
presença de distúrbios do sono	Vários dias	Vários dias
presença de distúrbios do sono	Quase todos os dias	Quase todos os dias
ruim	Vários dias	Nenhuma vez
boa	Mais da metade dos dias	Vários dias
boa	Vários dias	Vários dias
boa	Nenhuma vez	Vários dias
ruim	Vários dias	Vários dias
boa	Vários dias	Vários dias
ruim	Vários dias	Vários dias

presença de distúrbios do sono	Quase todos os dias	Mais da metade dos dias
ruim	Vários dias	Nenhuma vez
boa	Vários dias	Nenhuma vez
ruim	Vários dias	Vários dias
presença de distúrbios do sono	Quase todos os dias	Mais da metade dos dias
presença de distúrbios do sono	Quase todos os dias	Quase todos os dias
ruim	Vários dias	Vários dias
boa	Mais da metade dos dias	Quase todos os dias
ruim	Nenhuma vez	Nenhuma vez
ruim	Vários dias	Vários dias
ruim	Vários dias	Vários dias

presença de distúrbios do sono	Quase todos os dias	Vários dias
boa	Vários dias	Vários dias
ruim	Quase todos os dias	Quase todos os dias
ruim	Nenhuma vez	Vários dias
ruim	Mais da metade dos dias	Mais da metade dos dias
boa	Mais da metade dos dias	Nenhuma vez
ruim	Quase todos os dias	Quase todos os dias
boa	Nenhuma vez	Nenhuma vez
ruim	Vários dias	Vários dias
presença de distúrbios do sono	Quase todos os dias	Vários dias
ruim	Vários dias	Vários dias

ruim	Nenhuma vez	Nenhuma vez
ruim	Vários dias	Nenhuma vez
ruim	Vários dias	Mais da metade dos dias
ruim	Vários dias	Vários dias
ruim	Nenhuma vez	Nenhuma vez
presença de distúrbios do sono	Quase todos os dias	Quase todos os dias
boa	Vários dias	Vários dias
presença de distúrbios do sono	Quase todos os dias	Vários dias
presença de distúrbios do sono	Quase todos os dias	Quase todos os dias
presença de distúrbios do sono	Quase todos os dias	Quase todos os dias
boa	Vários dias	Vários dias
ruim	Mais da metade dos dias	Mais da metade dos dias

ruim	Mais da metade dos dias	Nenhuma vez
ruim	Vários dias	Vários dias
ruim	Vários dias	Vários dias
ruim	Quase todos os dias	Quase todos os dias
ruim	Vários dias	Vários dias
boa	Vários dias	Nenhuma vez
presença de distúrbios do sono	Quase todos os dias	Quase todos os dias
ruim	Mais da metade dos dias	Mais da metade dos dias
presença de distúrbios do sono	Vários dias	Vários dias
ruim	Vários dias	Vários dias
ruim	Vários dias	Vários dias

ruim	Vários dias	Vários dias
presença de distúrbios do sono	Mais da metade dos dias	Vários dias
presença de distúrbios do sono	Quase todos os dias	Quase todos os dias
ruim	Vários dias	Nenhuma vez
ruim	Quase todos os dias	Quase todos os dias
ruim	Quase todos os dias	Mais da metade dos dias
ruim	Vários dias	Mais da metade dos dias
ruim	Quase todos os dias	Mais da metade dos dias
ruim	Quase todos os dias	Quase todos os dias
presença de distúrbios do sono	Quase todos os dias	Quase todos os dias

presença de distúrbios do sono	Mais da metade dos dias	Vários dias
ruim	Vários dias	Vários dias
ruim	Mais da metade dos dias	Vários dias
ruim	Vários dias	Vários dias
boa	Quase todos os dias	Quase todos os dias
boa	Vários dias	Vários dias
ruim	Quase todos os dias	Quase todos os dias
ruim	Mais da metade dos dias	Vários dias
ruim	Mais da metade dos dias	Mais da metade dos dias
presença de distúrbios do sono	Quase todos os dias	Quase todos os dias
boa	Vários dias	Vários dias

boa	Vários dias	Vários dias
boa	Vários dias	Nenhuma vez
presença de distúrbios do sono	Mais da metade dos dias	Mais da metade dos dias
presença de distúrbios do sono	Vários dias	Vários dias
ruim	Quase todos os dias	Quase todos os dias
ruim	Quase todos os dias	Quase todos os dias
presença de distúrbios do sono	Quase todos os dias	Quase todos os dias
ruim	Vários dias	Vários dias
boa	Nenhuma vez	Nenhuma vez
presença de distúrbios do sono	Mais da metade dos dias	Mais da metade dos dias
presença de distúrbios do sono	Quase todos os dias	Quase todos os dias

ruim	Quase todos os dias	Mais da metade dos dias
presença de distúrbios do sono	Mais da metade dos dias	Quase todos os dias
boa	Vários dias	Vários dias
presença de distúrbios do sono	Quase todos os dias	Quase todos os dias
ruim	Quase todos os dias	Vários dias
ruim	Quase todos os dias	Nenhuma vez
ruim	Vários dias	Vários dias
ruim	Mais da metade dos dias	Vários dias
presença de distúrbios do sono	Mais da metade dos dias	Mais da metade dos dias
presença de distúrbios do sono	Quase todos os dias	Mais da metade dos dias

boa	Nenhuma vez	Nenhuma vez
ruim	Vários dias	Vários dias
ruim	Vários dias	Vários dias
presença de distúrbios do sono	Mais da metade dos dias	Vários dias
presença de distúrbios do sono	Vários dias	Vários dias
boa	Vários dias	Vários dias
presença de distúrbios do sono	Vários dias	Vários dias
boa	Vários dias	Nenhuma vez
ruim	Vários dias	Nenhuma vez
boa	Vários dias	Vários dias
ruim	Mais da metade dos dias	Mais da metade dos dias
ruim	Vários dias	Vários dias

ruim	Vários dias	Nenhuma vez
ruim	Vários dias	Vários dias
ruim	Vários dias	Vários dias
ruim	Nenhuma vez	Nenhuma vez
presença de distúrbios do sono	Vários dias	Nenhuma vez
ruim	Quase todos os dias	Mais da metade dos dias
ruim	Mais da metade dos dias	Mais da metade dos dias
ruim	Nenhuma vez	Nenhuma vez
ruim	Mais da metade dos dias	Quase todos os dias
ruim	Mais da metade dos dias	Mais da metade dos dias
ruim	Vários dias	Vários dias
ruim	Vários dias	Nenhuma vez

ruim	Quase todos os dias	Quase todos os dias
ruim	Vários dias	Nenhuma vez
ruim	Vários dias	Mais da metade dos dias
boa	Nenhuma vez	Nenhuma vez
boa	Vários dias	Vários dias
ruim	Quase todos os dias	Mais da metade dos dias
presença de distúrbios do sono	Mais da metade dos dias	Mais da metade dos dias
ruim	Vários dias	Nenhuma vez
ruim	Vários dias	Vários dias
ruim	Vários dias	Vários dias
ruim	Vários dias	Vários dias
ruim	Vários dias	Mais da metade dos dias

boa	Nenhuma vez	Nenhuma vez
ruim	Nenhuma vez	Nenhuma vez
ruim	Vários dias	Vários dias
presença de distúrbios do sono	Quase todos os dias	Mais da metade dos dias
ruim	Vários dias	Vários dias
boa	Nenhuma vez	Nenhuma vez
boa	Quase todos os dias	Quase todos os dias
presença de distúrbios do sono	Quase todos os dias	Quase todos os dias
presença de distúrbios do sono	Mais da metade dos dias	Mais da metade dos dias
ruim	Quase todos os dias	Quase todos os dias
ruim	Quase todos os dias	Quase todos os dias
presença de distúrbios do sono	Vários dias	Nenhuma vez

presença de distúrbios do sono	Vários dias	Vários dias
ruim	Vários dias	Mais da metade dos dias
boa	Nenhuma vez	Nenhuma vez
boa	Vários dias	Nenhuma vez
ruim	Mais da metade dos dias	Mais da metade dos dias
boa	Vários dias	Vários dias
ruim	Vários dias	Nenhuma vez
ruim	Vários dias	Nenhuma vez
boa	Nenhuma vez	Nenhuma vez
boa	Vários dias	Nenhuma vez
boa	Vários dias	Nenhuma vez
ruim	Vários dias	Nenhuma vez

presença de distúrbios do sono	Quase todos os dias	Quase todos os dias
ruim	Vários dias	Vários dias
ruim	Vários dias	Vários dias
ruim	Vários dias	Vários dias
boa	Nenhuma vez	Vários dias

Generalized Anxiety Disorder (GAD-7)

Preocupar-se muito com diversas coisas	Dificuldade para relaxar	Ficar tão agitado(a) que se torna difícil permanecer sentado (a)
Quase todos os dias	Mais da metade dos dias	Nenhuma vez
Vários dias	Vários dias	Nenhuma vez
Quase todos os dias	Quase todos os dias	Vários dias
Vários dias	Mais da metade dos dias	Nenhuma vez
Mais da metade dos dias	Vários dias	Mais da metade dos dias
Vários dias	Vários dias	Vários dias
Vários dias	Vários dias	Nenhuma vez
Vários dias	Vários dias	Nenhuma vez
Vários dias	Vários dias	Nenhuma vez

Mais da metade dos dias	Quase todos os dias	Nenhuma vez
Mais da metade dos dias	Mais da metade dos dias	Quase todos os dias
Vários dias	Vários dias	Nenhuma vez
Vários dias	Mais da metade dos dias	Nenhuma vez
Vários dias	Vários dias	Nenhuma vez
Quase todos os dias	Quase todos os dias	Mais da metade dos dias
Vários dias	Vários dias	Nenhuma vez
Vários dias	Vários dias	Nenhuma vez
Quase todos os dias	Mais da metade dos dias	Vários dias
Vários dias	Vários dias	Vários dias
Nenhuma vez	Nenhuma vez	Nenhuma vez

Vários dias	Vários dias	Nenhuma vez
Quase todos os dias	Vários dias	Mais da metade dos dias
Vários dias	Nenhuma vez	Nenhuma vez
Quase todos os dias	Mais da metade dos dias	Nenhuma vez
Quase todos os dias	Quase todos os dias	Quase todos os dias
Mais da metade dos dias	Vários dias	Vários dias
Quase todos os dias	Mais da metade dos dias	Quase todos os dias
Quase todos os dias	Quase todos os dias	Vários dias
Quase todos os dias	Mais da metade dos dias	Nenhuma vez
Mais da metade dos dias	Vários dias	Nenhuma vez

Mais da metade dos dias	Mais da metade dos dias	Vários dias
Quase todos os dias	Quase todos os dias	Mais da metade dos dias
Mais da metade dos dias	Vários dias	Nenhuma vez
Quase todos os dias	Quase todos os dias	Mais da metade dos dias
Vários dias	Quase todos os dias	Nenhuma vez
Vários dias	Nenhuma vez	Nenhuma vez
Mais da metade dos dias	Quase todos os dias	Vários dias
Quase todos os dias	Quase todos os dias	Vários dias
Quase todos os dias	Quase todos os dias	Vários dias
Vários dias	Vários dias	Nenhuma vez
Vários dias	Vários dias	Mais da metade dos dias
Vários dias	Nenhuma vez	Nenhuma vez

Quase todos os dias	Quase todos os dias	Mais da metade dos dias
Mais da metade dos dias	Vários dias	Nenhuma vez
Vários dias	Vários dias	Nenhuma vez
Quase todos os dias	Quase todos os dias	Quase todos os dias
Vários dias	Vários dias	Nenhuma vez
Vários dias	Vários dias	Nenhuma vez
Vários dias	Nenhuma vez	Nenhuma vez
Quase todos os dias	Mais da metade dos dias	Mais da metade dos dias
Quase todos os dias	Vários dias	Mais da metade dos dias
Vários dias	Vários dias	Vários dias

Vários dias	Mais da metade dos dias	Vários dias
Quase todos os dias	Quase todos os dias	Vários dias
Vários dias	Vários dias	Mais da metade dos dias
Quase todos os dias	Mais da metade dos dias	Vários dias
Mais da metade dos dias	Mais da metade dos dias	Vários dias
Quase todos os dias	Quase todos os dias	Vários dias
Vários dias	Vários dias	Vários dias
Vários dias	Vários dias	Vários dias
Nenhuma vez	Nenhuma vez	Nenhuma vez
Vários dias	Vários dias	Nenhuma vez
Vários dias	Nenhuma vez	Quase todos os dias

Vários dias	Mais da metade dos dias	Nenhuma vez
Quase todos os dias	Nenhuma vez	Vários dias
Vários dias	Vários dias	Nenhuma vez
Quase todos os dias	Quase todos os dias	Vários dias
Quase todos os dias	Vários dias	Nenhuma vez
Mais da metade dos dias	Mais da metade dos dias	Vários dias
Vários dias	Vários dias	Nenhuma vez
Nenhuma vez	Nenhuma vez	Nenhuma vez
Quase todos os dias	Vários dias	Nenhuma vez
Mais da metade dos dias	Vários dias	Nenhuma vez
Vários dias	Vários dias	Nenhuma vez

Quase todos os dias	Mais da metade dos dias	Nenhuma vez
Quase todos os dias	Quase todos os dias	Nenhuma vez
Nenhuma vez	Nenhuma vez	Nenhuma vez
Nenhuma vez	Nenhuma vez	Nenhuma vez
Quase todos os dias	Quase todos os dias	Vários dias
Quase todos os dias	Vários dias	Nenhuma vez
Quase todos os dias	Vários dias	Nenhuma vez
Nenhuma vez	Nenhuma vez	Nenhuma vez
Quase todos os dias	Mais da metade dos dias	Quase todos os dias
Vários dias	Mais da metade dos dias	Nenhuma vez
Quase todos os dias	Mais da metade dos dias	Nenhuma vez
Quase todos os dias	Quase todos os dias	Mais da metade dos dias

Vários dias	Nenhuma vez	Nenhuma vez
Vários dias	Vários dias	Nenhuma vez
Vários dias	Vários dias	Vários dias
Nenhuma vez	Vários dias	Nenhuma vez
Quase todos os dias	Quase todos os dias	Nenhuma vez
Mais da metade dos dias	Quase todos os dias	Mais da metade dos dias
Vários dias	Vários dias	Vários dias
Quase todos os dias	Quase todos os dias	Vários dias
Vários dias	Vários dias	Nenhuma vez
Quase todos os dias	Quase todos os dias	Mais da metade dos dias
Quase todos os dias	Vários dias	Quase todos os dias
Quase todos os dias	Mais da metade dos dias	Nenhuma vez

Mais da metade dos dias	Mais da metade dos dias	Mais da metade dos dias
Vários dias	Vários dias	Vários dias
Quase todos os dias	Mais da metade dos dias	Vários dias
Vários dias	Vários dias	Mais da metade dos dias
Nenhuma vez	Nenhuma vez	Vários dias
Quase todos os dias	Mais da metade dos dias	Nenhuma vez
Mais da metade dos dias	Quase todos os dias	Mais da metade dos dias
Vários dias	Quase todos os dias	Nenhuma vez
Quase todos os dias	Quase todos os dias	Quase todos os dias
Quase todos os dias	Quase todos os dias	Quase todos os dias
Vários dias	Vários dias	Nenhuma vez

Quase todos os dias	Vários dias	Mais da metade dos dias
Quase todos os dias	Quase todos os dias	Quase todos os dias
Quase todos os dias	Quase todos os dias	Quase todos os dias
Vários dias	Nenhuma vez	Mais da metade dos dias
Mais da metade dos dias	Quase todos os dias	Quase todos os dias
Mais da metade dos dias	Quase todos os dias	Vários dias
Quase todos os dias	Quase todos os dias	Vários dias
Mais da metade dos dias	Quase todos os dias	Mais da metade dos dias
Nenhuma vez	Nenhuma vez	Quase todos os dias
Vários dias	Vários dias	Nenhuma vez

Nenhuma vez	Nenhuma vez	Nenhuma vez
Mais da metade dos dias	Mais da metade dos dias	Vários dias
Vários dias	Vários dias	Nenhuma vez
Vários dias	Mais da metade dos dias	Vários dias
Quase todos os dias	Vários dias	Nenhuma vez
Vários dias	Vários dias	Vários dias
Vários dias	Mais da metade dos dias	Vários dias
Mais da metade dos dias	Quase todos os dias	Vários dias
Vários dias	Vários dias	Nenhuma vez
Mais da metade dos dias	Mais da metade dos dias	Vários dias
Vários dias	Nenhuma vez	Nenhuma vez

Vários dias	Vários dias	Vários dias
Mais da metade dos dias	Quase todos os dias	Vários dias
Quase todos os dias	Quase todos os dias	Quase todos os dias
Vários dias	Quase todos os dias	Nenhuma vez
Vários dias	Vários dias	Nenhuma vez
Vários dias	Vários dias	Nenhuma vez
Quase todos os dias	Mais da metade dos dias	Quase todos os dias
Vários dias	Vários dias	Vários dias
Quase todos os dias	Quase todos os dias	Quase todos os dias
Quase todos os dias	Quase todos os dias	Nenhuma vez
Quase todos os dias	Quase todos os dias	Nenhuma vez

Vários dias	Nenhuma vez	Nenhuma vez
Quase todos os dias	Quase todos os dias	Mais da metade dos dias
Mais da metade dos dias	Vários dias	Vários dias
Nenhuma vez	Nenhuma vez	Vários dias
Mais da metade dos dias	Mais da metade dos dias	Nenhuma vez
Vários dias	Vários dias	Nenhuma vez
Vários dias	Nenhuma vez	Nenhuma vez
Nenhuma vez	Nenhuma vez	Quase todos os dias
Vários dias	Vários dias	Vários dias
Mais da metade dos dias	Mais da metade dos dias	Nenhuma vez
Vários dias	Nenhuma vez	Vários dias
Vários dias	Vários dias	Nenhuma vez

Vários dias	Vários dias	Nenhuma vez
Mais da metade dos dias	Mais da metade dos dias	Nenhuma vez
Quase todos os dias	Mais da metade dos dias	Mais da metade dos dias
Nenhuma vez	Nenhuma vez	Nenhuma vez
Vários dias	Vários dias	Vários dias
Mais da metade dos dias	Mais da metade dos dias	Mais da metade dos dias
Mais da metade dos dias	Mais da metade dos dias	Vários dias
Quase todos os dias	Quase todos os dias	Vários dias
Quase todos os dias	Quase todos os dias	Quase todos os dias
Quase todos os dias	Mais da metade dos dias	Nenhuma vez
Quase todos os dias	Quase todos os dias	Quase todos os dias
Vários dias	Vários dias	Nenhuma vez

Nenhuma vez	Nenhuma vez	Nenhuma vez
Vários dias	Vários dias	Nenhuma vez
Vários dias	Vários dias	Vários dias
Quase todos os dias	Quase todos os dias	Mais da metade dos dias
Vários dias	Vários dias	Nenhuma vez
Mais da metade dos dias	Vários dias	Vários dias
Vários dias	Vários dias	Nenhuma vez
Vários dias	Vários dias	Vários dias
Mais da metade dos dias	Vários dias	Nenhuma vez
Nenhuma vez	Vários dias	Nenhuma vez
Vários dias	Vários dias	Nenhuma vez

Vários dias	Mais da metade dos dias	Vários dias
Nenhuma vez	Nenhuma vez	Nenhuma vez
Nenhuma vez	Nenhuma vez	Nenhuma vez
Vários dias	Vários dias	Vários dias
Quase todos os dias	Quase todos os dias	Nenhuma vez
Quase todos os dias	Quase todos os dias	Quase todos os dias
Vários dias	Vários dias	Nenhuma vez
Quase todos os dias	Vários dias	Vários dias
Vários dias	Vários dias	Nenhuma vez
Vários dias	Vários dias	Nenhuma vez
Vários dias	Vários dias	Vários dias

Mais da metade dos dias	Quase todos os dias	Mais da metade dos dias
Vários dias	Vários dias	Nenhuma vez
Quase todos os dias	Mais da metade dos dias	Vários dias
Vários dias	Vários dias	Nenhuma vez
Mais da metade dos dias	Mais da metade dos dias	Nenhuma vez
Mais da metade dos dias	Nenhuma vez	Nenhuma vez
Quase todos os dias	Quase todos os dias	Vários dias
Vários dias	Nenhuma vez	Nenhuma vez
Vários dias	Vários dias	Nenhuma vez
Vários dias	Mais da metade dos dias	Vários dias
Vários dias	Vários dias	Nenhuma vez

Nenhuma vez	Vários dias	Vários dias
Vários dias	Nenhuma vez	Nenhuma vez
Mais da metade dos dias	Quase todos os dias	Nenhuma vez
Vários dias	Vários dias	Nenhuma vez
Nenhuma vez	Nenhuma vez	Nenhuma vez
Quase todos os dias	Quase todos os dias	Quase todos os dias
Vários dias	Vários dias	Vários dias
Vários dias	Vários dias	Vários dias
Quase todos os dias	Quase todos os dias	Vários dias
Quase todos os dias	Quase todos os dias	Nenhuma vez
Vários dias	Nenhuma vez	Nenhuma vez
Mais da metade dos dias	Mais da metade dos dias	Vários dias

Vários dias	Vários dias	Vários dias
Vários dias	Vários dias	Nenhuma vez
Vários dias	Vários dias	Nenhuma vez
Quase todos os dias	Quase todos os dias	Nenhuma vez
Vários dias	Vários dias	Vários dias
Nenhuma vez	Nenhuma vez	Nenhuma vez
Quase todos os dias	Quase todos os dias	Quase todos os dias
Quase todos os dias	Vários dias	Vários dias
Vários dias	Vários dias	Vários dias
Vários dias	Vários dias	Vários dias
Vários dias	Vários dias	Nenhuma vez

Vários dias	Vários dias	Vários dias
Quase todos os dias	Mais da metade dos dias	Nenhuma vez
Quase todos os dias	Quase todos os dias	Mais da metade dos dias
Vários dias	Vários dias	Nenhuma vez
Quase todos os dias	Quase todos os dias	Quase todos os dias
Quase todos os dias	Quase todos os dias	Nenhuma vez
Quase todos os dias	Mais da metade dos dias	Vários dias
Quase todos os dias	Quase todos os dias	Mais da metade dos dias
Quase todos os dias	Quase todos os dias	Vários dias
Quase todos os dias	Mais da metade dos dias	Vários dias

Mais da metade dos dias	Vários dias	Nenhuma vez
Vários dias	Vários dias	Vários dias
Vários dias	Vários dias	Nenhuma vez
Quase todos os dias	Mais da metade dos dias	Vários dias
Quase todos os dias	Mais da metade dos dias	Nenhuma vez
Vários dias	Vários dias	Vários dias
Quase todos os dias	Mais da metade dos dias	Mais da metade dos dias
Quase todos os dias	Vários dias	Vários dias
Mais da metade dos dias	Mais da metade dos dias	Nenhuma vez
Quase todos os dias	Quase todos os dias	Quase todos os dias
Vários dias	Vários dias	Vários dias

Vários dias	Vários dias	Nenhuma vez
Vários dias	Vários dias	Nenhuma vez
Mais da metade dos dias	Mais da metade dos dias	Vários dias
Quase todos os dias	Mais da metade dos dias	Mais da metade dos dias
Quase todos os dias	Quase todos os dias	Mais da metade dos dias
Quase todos os dias	Quase todos os dias	Vários dias
Quase todos os dias	Quase todos os dias	Quase todos os dias
Vários dias	Vários dias	Vários dias
Nenhuma vez	Nenhuma vez	Nenhuma vez
Quase todos os dias	Quase todos os dias	Vários dias
Quase todos os dias	Quase todos os dias	Quase todos os dias

Quase todos os dias	Mais da metade dos dias	Vários dias
Quase todos os dias	Mais da metade dos dias	Vários dias
Vários dias	Vários dias	Nenhuma vez
Quase todos os dias	Quase todos os dias	Quase todos os dias
Mais da metade dos dias	Mais da metade dos dias	Nenhuma vez
Quase todos os dias	Nenhuma vez	Nenhuma vez
Vários dias	Vários dias	Vários dias
Quase todos os dias	Quase todos os dias	Vários dias
Vários dias	Mais da metade dos dias	Vários dias
Mais da metade dos dias	Quase todos os dias	Vários dias

Nenhuma vez	Nenhuma vez	Nenhuma vez
Vários dias	Vários dias	Nenhuma vez
Vários dias	Vários dias	Nenhuma vez
Vários dias	Vários dias	Nenhuma vez
Vários dias	Vários dias	Vários dias
Vários dias	Vários dias	Nenhuma vez
Vários dias	Vários dias	Nenhuma vez
Vários dias	Vários dias	Vários dias
Nenhuma vez	Nenhuma vez	Nenhuma vez
Vários dias	Vários dias	Nenhuma vez
Quase todos os dias	Quase todos os dias	Vários dias
Vários dias	Mais da metade dos dias	Vários dias

Nenhuma vez	Nenhuma vez	Nenhuma vez
Vários dias	Vários dias	Vários dias
Vários dias	Vários dias	Nenhuma vez
Nenhuma vez	Nenhuma vez	Nenhuma vez
Vários dias	Vários dias	Nenhuma vez
Quase todos os dias	Quase todos os dias	Nenhuma vez
Mais da metade dos dias	Mais da metade dos dias	Vários dias
Nenhuma vez	Nenhuma vez	Nenhuma vez
Quase todos os dias	Quase todos os dias	Nenhuma vez
Mais da metade dos dias	Mais da metade dos dias	Vários dias
Vários dias	Vários dias	Vários dias
Vários dias	Vários dias	Nenhuma vez

Quase todos os dias	Quase todos os dias	Nenhuma vez
Vários dias	Vários dias	Nenhuma vez
Mais da metade dos dias	Mais da metade dos dias	Nenhuma vez
Vários dias	Nenhuma vez	Nenhuma vez
Vários dias	Vários dias	Vários dias
Quase todos os dias	Quase todos os dias	Vários dias
Mais da metade dos dias	Mais da metade dos dias	Vários dias
Mais da metade dos dias	Vários dias	Nenhuma vez
Vários dias	Vários dias	Nenhuma vez
Vários dias	Vários dias	Nenhuma vez
Vários dias	Vários dias	Vários dias
Vários dias	Mais da metade dos dias	Mais da metade dos dias

Nenhuma vez	Nenhuma vez	Nenhuma vez
Nenhuma vez	Nenhuma vez	Nenhuma vez
Mais da metade dos dias	Nenhuma vez	Quase todos os dias
Vários dias	Vários dias	Nenhuma vez
Vários dias	Vários dias	Nenhuma vez
Nenhuma vez	Nenhuma vez	Nenhuma vez
Quase todos os dias	Quase todos os dias	Vários dias
Quase todos os dias	Quase todos os dias	Vários dias
Vários dias	Vários dias	Vários dias
Quase todos os dias	Quase todos os dias	Quase todos os dias
Quase todos os dias	Quase todos os dias	Mais da metade dos dias
Vários dias	Vários dias	Vários dias

Vários dias	Vários dias	Vários dias
Mais da metade dos dias	Vários dias	Vários dias
Vários dias	Nenhuma vez	Nenhuma vez
Vários dias	Vários dias	Nenhuma vez
Vários dias	Vários dias	Vários dias
Vários dias	Vários dias	Vários dias
Vários dias	Vários dias	Nenhuma vez
Nenhuma vez	Nenhuma vez	Nenhuma vez
Vários dias	Nenhuma vez	Nenhuma vez
Vários dias	Nenhuma vez	Nenhuma vez
Nenhuma vez	Vários dias	Vários dias
Vários dias	Nenhuma vez	Nenhuma vez

Quase todos os dias	Quase todos os dias	Vários dias
Vários dias	Vários dias	Nenhuma vez
Vários dias	Vários dias	Vários dias
Vários dias	Vários dias	Vários dias
Vários dias	Nenhuma vez	Nenhuma vez

Ficar facilmente aborrecido(a) ou irritado(a)	Sentir medo como se algo ruim fosse acontecer	To tal
Vários dias	Vários dias	13
Vários dias	Quase todos os dias	8
Quase todos os dias	Mais da metade dos dias	17
Vários dias	Nenhuma vez	6
Vários dias	Vários dias	9
Vários dias	Vários dias	7
Mais da metade dos dias	Vários dias	7
Vários dias	Vários dias	6
Vários dias	Vários dias	6

Quase todos os dias	Mais da metade dos dias	12
Quase todos os dias	Vários dias	16
Nenhuma vez	Nenhuma vez	4
Nenhuma vez	Vários dias	6
Nenhuma vez	Nenhuma vez	3
Mais da metade dos dias	Vários dias	17
Mais da metade dos dias	Vários dias	8
Vários dias	Vários dias	6
Mais da metade dos dias	Mais da metade dos dias	16
Vários dias	Vários dias	7
Nenhuma vez	Nenhuma vez	0

Vários dias	Vários dias	6
Mais da metade dos dias	Vários dias	11
Nenhuma vez	Nenhuma vez	3
Mais da metade dos dias	Vários dias	13
Quase todos os dias	Quase todos os dias	21
Vários dias	Vários dias	8
Vários dias	Vários dias	14
Quase todos os dias	Vários dias	15
Vários dias	Quase todos os dias	12
Vários dias	Nenhuma vez	7

Vários dias	Vários dias	10
Nenhuma vez	Nenhuma vez	11
Nenhuma vez	Vários dias	7
Mais da metade dos dias	Vários dias	17
Vários dias	Vários dias	8
Nenhuma vez	Nenhuma vez	2
Quase todos os dias	Mais da metade dos dias	15
Vários dias	Mais da metade dos dias	16
Quase todos os dias	Quase todos os dias	19
Vários dias	Nenhuma vez	5
Vários dias	Vários dias	9
Nenhuma vez	Nenhuma vez	3

Mais da metade dos dias	Quase todos os dias	19
Vários dias	Vários dias	8
Vários dias	Vários dias	6
Mais da metade dos dias	Nenhuma vez	16
Nenhuma vez	Nenhuma vez	4
Vários dias	Vários dias	6
Nenhuma vez	Nenhuma vez	2
Vários dias	Vários dias	13
Nenhuma vez	Nenhuma vez	8
Vários dias	Vários dias	6

Vários dias	Vários dias	11
Quase todos os dias	Quase todos os dias	19
Vários dias	Quase todos os dias	10
Vários dias	Vários dias	12
Vários dias	Vários dias	11
Mais da metade dos dias	Mais da metade dos dias	16
Nenhuma vez	Vários dias	8
Mais da metade dos dias	Vários dias	8
Vários dias	Nenhuma vez	2
Nenhuma vez	Vários dias	5
Mais da metade dos dias	Nenhuma vez	6

Vários dias	Nenhuma vez	6
Quase todos os dias	Nenhuma vez	9
Vários dias	Mais da metade dos dias	7
Mais da metade dos dias	Vários dias	15
Vários dias	Mais da metade dos dias	11
Vários dias	Mais da metade dos dias	12
Nenhuma vez	Nenhuma vez	3
Nenhuma vez	Vários dias	2
Nenhuma vez	Vários dias	7
Quase todos os dias	Vários dias	9
Nenhuma vez	Nenhuma vez	3

Mais da metade dos dias	Nenhuma vez	11
Vários dias	Vários dias	10
Nenhuma vez	Vários dias	1
Nenhuma vez	Nenhuma vez	0
Quase todos os dias	Nenhuma vez	15
Vários dias	Quase todos os dias	13
Vários dias	Nenhuma vez	7
Nenhuma vez	Nenhuma vez	0
Quase todos os dias	Quase todos os dias	20
Vários dias	Vários dias	7
Vários dias	Quase todos os dias	14
Quase todos os dias	Quase todos os dias	20

Vários dias	Vários dias	4
Vários dias	Vários dias	5
Vários dias	Nenhuma vez	6
Nenhuma vez	Nenhuma vez	1
Mais da metade dos dias	Quase todos os dias	17
Mais da metade dos dias	Mais da metade dos dias	15
Nenhuma vez	Nenhuma vez	5
Vários dias	Vários dias	14
Vários dias	Vários dias	6
Quase todos os dias	Quase todos os dias	18
Vários dias	Mais da metade dos dias	13
Vários dias	Nenhuma vez	11

Mais da metade dos dias	Mais da metade dos dias	12
Vários dias	Vários dias	6
Mais da metade dos dias	Mais da metade dos dias	16
Vários dias	Vários dias	8
Nenhuma vez	Nenhuma vez	2
Mais da metade dos dias	Nenhuma vez	11
Quase todos os dias	Mais da metade dos dias	17
Vários dias	Nenhuma vez	8
Quase todos os dias	Quase todos os dias	21
Quase todos os dias	Quase todos os dias	21
Vários dias	Mais da metade dos dias	7

Mais da metade dos dias	Quase todos os dias	17
Quase todos os dias	Quase todos os dias	21
Quase todos os dias	Quase todos os dias	21
Vários dias	Vários dias	7
Quase todos os dias	Mais da metade dos dias	19
Vários dias	Nenhuma vez	9
Vários dias	Vários dias	15
Quase todos os dias	Quase todos os dias	17
Nenhuma vez	Nenhuma vez	3
Vários dias	Vários dias	6

Vários dias	Nenhuma vez	2
Quase todos os dias	Nenhuma vez	11
Vários dias	Vários dias	6
Vários dias	Vários dias	9
Vários dias	Nenhuma vez	7
Mais da metade dos dias	Mais da metade dos dias	9
Nenhuma vez	Vários dias	7
Vários dias	Nenhuma vez	9
Vários dias	Nenhuma vez	4
Vários dias	Vários dias	12
Nenhuma vez	Vários dias	2

Quase todos os dias	Quase todos os dias	13
Quase todos os dias	Mais da metade dos dias	15
Quase todos os dias	Quase todos os dias	21
Mais da metade dos dias	Vários dias	9
Nenhuma vez	Vários dias	4
Vários dias	Nenhuma vez	4
Vários dias	Nenhuma vez	15
Vários dias	Nenhuma vez	6
Quase todos os dias	Quase todos os dias	21
Vários dias	Vários dias	12
Quase todos os dias	Vários dias	16

Nenhuma vez	Nenhuma vez	2
Mais da metade dos dias	Mais da metade dos dias	17
Mais da metade dos dias	Quase todos os dias	12
Nenhuma vez	Vários dias	2
Vários dias	Quase todos os dias	12
Vários dias	Nenhuma vez	5
Nenhuma vez	Mais da metade dos dias	3
Nenhuma vez	Nenhuma vez	4
Vários dias	Vários dias	8
Mais da metade dos dias	Quase todos os dias	13
Vários dias	Nenhuma vez	5
Nenhuma vez	Vários dias	5

Vários dias	Nenhuma vez	4
Mais da metade dos dias	Mais da metade dos dias	12
Mais da metade dos dias	Vários dias	13
Vários dias	Nenhuma vez	2
Vários dias	Vários dias	8
Quase todos os dias	Quase todos os dias	16
Vários dias	Vários dias	10
Quase todos os dias	Mais da metade dos dias	17
Quase todos os dias	Quase todos os dias	21
Quase todos os dias	Mais da metade dos dias	16
Mais da metade dos dias	Vários dias	15
Vários dias	Vários dias	5

Nenhuma vez	Nenhuma vez	1
Vários dias	Nenhuma vez	5
Quase todos os dias	Vários dias	9
Quase todos os dias	Quase todos os dias	20
Nenhuma vez	Vários dias	4
Nenhuma vez	Vários dias	8
Nenhuma vez	Nenhuma vez	4
Vários dias	Nenhuma vez	5
Vários dias	Nenhuma vez	6
Vários dias	Nenhuma vez	4
Quase todos os dias	Vários dias	8

Vários dias	Vários dias	11
Vários dias	Nenhuma vez	2
Nenhuma vez	Nenhuma vez	1
Vários dias	Vários dias	7
Vários dias	Quase todos os dias	15
Quase todos os dias	Quase todos os dias	21
Vários dias	Vários dias	6
Vários dias	Vários dias	12
Vários dias	Nenhuma vez	3
Vários dias	Vários dias	6
Vários dias	Vários dias	7

Vários dias	Mais da metade dos dias	14
Mais da metade dos dias	Mais da metade dos dias	8
Quase todos os dias	Quase todos os dias	18
Vários dias	Vários dias	5
Vários dias	Nenhuma vez	9
Vários dias	Vários dias	6
Quase todos os dias	Quase todos os dias	19
Nenhuma vez	Nenhuma vez	1
Mais da metade dos dias	Vários dias	7
Quase todos os dias	Mais da metade dos dias	13
Vários dias	Quase todos os dias	8

Nenhuma vez	Nenhuma vez	2
Vários dias	Nenhuma vez	3
Quase todos os dias	Vários dias	12
Nenhuma vez	Nenhuma vez	4
Nenhuma vez	Nenhuma vez	0
Quase todos os dias	Quase todos os dias	21
Vários dias	Nenhuma vez	6
Vários dias	Vários dias	9
Vários dias	Vários dias	15
Mais da metade dos dias	Quase todos os dias	17
Vários dias	Nenhuma vez	4
Vários dias	Vários dias	11

Mais da metade dos dias	Quase todos os dias	10
Nenhuma vez	Vários dias	5
Vários dias	Nenhuma vez	5
Quase todos os dias	Nenhuma vez	15
Vários dias	Vários dias	7
Vários dias	Nenhuma vez	2
Nenhuma vez	Quase todos os dias	18
Mais da metade dos dias	Nenhuma vez	11
Vários dias	Vários dias	7
Vários dias	Vários dias	7
Vários dias	Vários dias	6

Vários dias	Vários dias	7
Vários dias	Nenhuma vez	9
Quase todos os dias	Quase todos os dias	20
Vários dias	Nenhuma vez	4
Quase todos os dias	Quase todos os dias	21
Quase todos os dias	Mais da metade dos dias	16
Quase todos os dias	Vários dias	13
Mais da metade dos dias	Vários dias	16
Vários dias	Quase todos os dias	17
Vários dias	Vários dias	14

Vários dias	Quase todos os dias	10
Vários dias	Nenhuma vez	6
Vários dias	Vários dias	7
Quase todos os dias	Nenhuma vez	11
Quase todos os dias	Quase todos os dias	17
Mais da metade dos dias	Mais da metade dos dias	9
Quase todos os dias	Quase todos os dias	19
Vários dias	Quase todos os dias	12
Mais da metade dos dias	Nenhuma vez	10
Quase todos os dias	Quase todos os dias	21
Vários dias	Vários dias	7

Nenhuma vez	Nenhuma vez	4
Mais da metade dos dias	Vários dias	6
Vários dias	Mais da metade dos dias	12
Vários dias	Mais da metade dos dias	12
Quase todos os dias	Quase todos os dias	20
Vários dias	Vários dias	15
Mais da metade dos dias	Quase todos os dias	20
Vários dias	Vários dias	7
Nenhuma vez	Nenhuma vez	0
Vários dias	Nenhuma vez	12
Quase todos os dias	Quase todos os dias	21

Mais da metade dos dias	Quase todos os dias	16
Quase todos os dias	Vários dias	15
Vários dias	Nenhuma vez	5
Quase todos os dias	Quase todos os dias	21
Vários dias	Vários dias	10
Vários dias	Nenhuma vez	7
Vários dias	Vários dias	7
Mais da metade dos dias	Quase todos os dias	15
Vários dias	Vários dias	10
Quase todos os dias	Quase todos os dias	17

Nenhuma vez	Nenhuma vez	0
Vários dias	Vários dias	6
Vários dias	Mais da metade dos dias	7
Quase todos os dias	Nenhuma vez	8
Nenhuma vez	Nenhuma vez	5
Vários dias	Nenhuma vez	5
Vários dias	Vários dias	6
Vários dias	Nenhuma vez	5
Vários dias	Nenhuma vez	2
Vários dias	Vários dias	6
Vários dias	Vários dias	13
Vários dias	Vários dias	8

Vários dias	Nenhuma vez	2
Vários dias	Vários dias	7
Vários dias	Nenhuma vez	5
Nenhuma vez	Nenhuma vez	0
Vários dias	Vários dias	5
Quase todos os dias	Mais da metade dos dias	16
Mais da metade dos dias	Vários dias	12
Nenhuma vez	Nenhuma vez	0
Vários dias	Vários dias	13
Vários dias	Vários dias	11
Vários dias	Vários dias	7
Vários dias	Nenhuma vez	4

Mais da metade dos dias	Vários dias	15
Vários dias	Vários dias	5
Mais da metade dos dias	Vários dias	10
Nenhuma vez	Vários dias	2
Vários dias	Vários dias	7
Quase todos os dias	Quase todos os dias	18
Vários dias	Vários dias	11
Vários dias	Nenhuma vez	5
Nenhuma vez	Vários dias	5
Vários dias	Vários dias	6
Vários dias	Vários dias	7
Mais da metade dos dias	Nenhuma vez	10

Nenhuma vez	Nenhuma vez	0
Nenhuma vez	Nenhuma vez	0
Mais da metade dos dias	Nenhuma vez	9
Vários dias	Vários dias	9
Vários dias	Vários dias	6
Nenhuma vez	Nenhuma vez	0
Quase todos os dias	Quase todos os dias	19
Quase todos os dias	Vários dias	17
Mais da metade dos dias	Nenhuma vez	9
Quase todos os dias	Quase todos os dias	21
Quase todos os dias	Quase todos os dias	20
Vários dias	Nenhuma vez	5

Vários dias	Nenhuma vez	6
Vários dias	Nenhuma vez	8
Vários dias	Nenhuma vez	2
Nenhuma vez	Vários dias	4
Mais da metade dos dias	Vários dias	10
Vários dias	Nenhuma vez	6
Nenhuma vez	Nenhuma vez	3
Nenhuma vez	Nenhuma vez	1
Nenhuma vez	Nenhuma vez	1
Nenhuma vez	Nenhuma vez	2
Vários dias	Nenhuma vez	4
Vários dias	Nenhuma vez	3

Quase todos os dias	Mais da metade dos dias	18
Vários dias	Vários dias	6
Nenhuma vez	Vários dias	6
Vários dias	Vários dias	7
Nenhuma vez	Nenhuma vez	2

NULL	registro_da_	termo_de_c	questionario_para_a_avaliacao_sociodemografica_de_saude_hal					
entrevistados	data	concorda_co	ida	cidade_e_es	estado	regiao	sexo	raca_cor
E01	2021-05-26	SIM	20	Sao_Paulo_S	SP	Sudeste	Feminino	Branco
E02	2021-05-29	SIM	20	Rio_de_Jane	RJ	Sudeste	Feminino	Preto
E03	2021-06-29	SIM	21	Sao_Carlos-S	SP	Sudeste	Feminino	Branco
E04	2021-02-09	SIM	22	Sao_Carlos	SP	Sudeste	Feminino	parda
E05	2021-03-19	SIM	22	Goiania-_Go	GO	Centro-Oeste	Masculino	parda
E06	2021-04-24	SIM	22	Palmeira_da	RS	Sul	Masculino	parda
E07	2021-05-24	SIM	22	Sao_Paulo-S	SP	Sudeste	Masculino	parda
E08	2021-02-09	SIM	23	Sao_Carlos_	SP	Sudeste	Feminino	parda
E09	2021-02-09	SIM	23	Sao_Carlos_	SP	Sudeste	Feminino	Branco
E10	2021-04-24	SIM	23	Sao_Paulo-S	SP	Sudeste	Masculino	parda
E11	2021-04-26	SIM	23	Jundiai-_Sao	SP	Sudeste	Masculino	Branco
E12	2021-05-23	SIM	23	Brasilia	DF	Distrito_Fede	Feminino	parda
E13	2022-01-06	SIM	23	Sao_Paulo_S	SP	Sudeste	Feminino	Ignorado
E14	2022-01-08	SIM	23	Santa_Cruz_	PE	Nordeste	Masculino	Branco
E15	2022-01-09	SIM	23	Sao_Paulo_-	SP	Sudeste	Masculino	Branco
E16	2021-02-09	SIM	24	Quata_SP	SP	Sudeste	Feminino	Branco
E17	2021-02-26	SIM	24	Limeira-_Sao	SP	Sudeste	Feminino	Branco
E18	2021-03-10	SIM	24	Goiania_Goi	GO	Centro-Oeste	Feminino	Preto
E19	2021-03-11	SIM	24	Cirilandia-_G	GO	Centro-Oeste	Feminino	Branco
E20	2021-03-20	SIM	24	Sao_Paulo_S	SP	Sudeste	Feminino	Branco
E21	2021-04-23	SIM	24	Rio_de_Jane	RJ	Sudeste	Masculino	Branco
E22	2021-04-24	SIM	24	Guaxupe-MG	MG	Sudeste	Masculino	Branco
E23	2021-05-24	SIM	24	Goiania-_GO	GO	Centro-Oeste	Masculino	parda
E24	2021-05-25	SIM	24	Sao_Carlos_	SP	Sudeste	Feminino	parda
E25	2021-05-26	SIM	24	Garca_sp	SP	Sudeste	Feminino	Branco
E26	2022-01-09	SIM	24	JABOATaO_	PB	Nordeste	Masculino	Branco
E27	2021-02-06	SIM	25	Sao_Paulo-S	SP	Sudeste	Feminino	Branco
E28	2021-02-09	SIM	25	Sao_Carlos	SP	Sudeste	Feminino	Branco
E29	2021-03-10	SIM	25	Maceio-AL	AL	Nordeste	Feminino	parda
E30	2021-03-19	SIM	25	Curitiba_-_PI	PR	Sul	Masculino	Branco
E31	2021-03-19	SIM	25	Sao_Paulo_S	SP	Sudeste	Feminino	Ignorado
E32	2021-03-19	SIM	25	SP-SP	SP	Sudeste	Masculino	parda
E33	2021-04-24	SIM	25	Salvador	BA	Nordeste	Masculino	parda
E34	2021-04-25	SIM	25	Araraquara_-	SP	Sudeste	Masculino	Branco
E35	2021-04-25	SIM	25	Itupeva-SP	SP	Sudeste	Masculino	Branco
E36	2021-05-31	SIM	25	Goiania-GO	GO	Centro-Oeste	Masculino	parda
E37	2021-06-24	SIM	25	Guarulhos-SI	SP	Sudeste	Feminino	Branco
E38	2021-09-16	SIM	25	Teresina-Pia	PI	Nordeste	Feminino	Amarelo
E39	2022-01-05	SIM	25	Praia_Grand	SP	Sudeste	Feminino	Branco
E40	2022-01-05	SIM	25	Blumenau-_S	SC	Sul	Masculino	Branco
E41	2022-01-05	SIM	25	SaO_PAULO	SP	Sudeste	Feminino	Amarelo
E42	2022-01-06	SIM	25	Santa_Rosa_	RS	Sul	Masculino	Branco
E43	2022-01-08	SIM	25	Brasilia_DF	DF	Distrito_Fede	Masculino	Branco
E44	2022-01-09	SIM	25	Juazeiro_do_	CE	Nordeste	Feminino	Branco
E45	2022-01-09	SIM	25	SE	SE	Nordeste	Masculino	Branco
E46	2022-01-09	SIM	25	Ribeirao_Pre	SP	Sudeste	Masculino	Branco
E47	2021-02-06	SIM	26	SaO_PAULO	SP	Sudeste	Feminino	Branco
E48	2021-02-07	SIM	26	Aparecida_d	GO	Centro-Oeste	Feminino	Branco
E49	2021-02-10	SIM	26	Sao_Paulo	SP	Sudeste	Feminino	parda
E50	2021-02-10	SIM	26	Cavalcante-(	GO	Centro-Oeste	Feminino	parda
E51	2021-02-12	SIM	26	Sao_Carlos-_	SP	Sudeste	Masculino	Branco
E52	2021-02-28	SIM	26	Sao_Carlos-S	SP	Sudeste	Feminino	parda
E53	2021-03-10	SIM	26	Sao_Luiz_Go	RS	Sul	Feminino	Branco
E54	2021-03-19	SIM	26	Goiania-_Go	GO	Centro-Oeste	Feminino	parda
E55	2021-03-19	SIM	26	Sao_Carlos_	SP	Sudeste	Masculino	Branco

E56	2021-03-20	SIM	26	Goiania-_Go	GO	Centro-Oeste	Feminino	parda
E57	2021-03-20	SIM	26	Aparecidas_(	GO	Centro-Oeste	Masculino	parda
E58	2021-03-23	SIM	26	Goiania-Goia	GO	Centro-Oeste	Feminino	Branco
E59	2021-04-24	SIM	26	Sao_Paulo_-	SP	Sudeste	Masculino	parda
E60	2021-04-24	SIM	26	Sao_Paulo-S	SP	Sudeste	Masculino	Branco
E61	2021-04-24	SIM	26	Maceio	AL	Nordeste	Masculino	Branco
E62	2021-04-24	SIM	26	Niteroi-RJ	RJ	Sudeste	Masculino	Branco
E63	2021-04-24	SIM	26	Sao_Jose_d	SP	Sudeste	Masculino	Branco
E64	2021-05-23	SIM	26	Goiania-GO	GO	Centro-Oeste	Feminino	parda
E65	2021-05-24	SIM	26	Impertariz-_M	MA	Nordeste	Feminino	Branco
E66	2021-05-26	SIM	26	Goiania	GO	Centro-Oeste	Feminino	parda
E67	2021-05-31	SIM	26	Sao_Carlos_	SP	Sudeste	Masculino	Branco
E68	2021-06-24	SIM	26	Sao_Carlos_	SP	Sudeste	Masculino	Branco
E69	2021-06-24	SIM	26	Sao_Carlos_	SP	Sudeste	Feminino	Branco
E70	2022-01-04	SIM	26	Sao_Carlos_	SP	Sudeste	Feminino	Branco
E71	2022-01-06	SIM	26	Sao_Paulo_-	SP	Sudeste	Masculino	Branco
E72	2022-01-07	SIM	26	Florianopolis	SC	Sul	Feminino	Branco
E73	2022-01-10	SIM	26	Maracanau_-	CE	Nordeste	Masculino	Branco
E74	2022-01-12	SIM	26	Curitiba-_Pa	PR	Sul	Masculino	Branco
E75	2022-01-24	SIM	26	Goiania_-_G	GO	Centro-Oeste	Feminino	Branco
E76	2022-01-24	SIM	26	Goiania_-_G	GO	Centro-Oeste	Feminino	Branco
E77	2021-03-20	SIM	27	Goiania_-_G	GO	Centro-Oeste	Feminino	Branco
E78	2021-03-20	SIM	27	Aracruz-ES	ES	Sudeste	Masculino	Branco
E79	2021-03-23	SIM	27	Ananindeua_	PA	Norte	Feminino	Branco
E80	2021-04-14	SIM	27	Belo_Horizor	MG	Sudeste	Feminino	Branco
E81	2021-04-24	SIM	27	Florianopolis	SC	Sul	Masculino	Branco
E82	2021-04-26	SIM	27	Sao_Bernarc	SP	Sudeste	Masculino	Branco
E83	2021-05-24	SIM	27	Goiania-_Go	GO	Centro-Oeste	Feminino	Branco
E84	2021-05-24	SIM	27	Goiania	GO	Centro-Oeste	Feminino	parda
E85	2021-05-25	SIM	27	Aparecida_d	GO	Centro-Oeste	Feminino	Branco
E86	2021-06-24	SIM	27	SaO_PAULC	SP	Sudeste	Feminino	parda
E87	2022-01-06	SIM	27	Sao_Paulo_(	SP	Sudeste	Masculino	parda
E88	2022-01-08	SIM	27	Fortaleza-_C	CE	Nordeste	Masculino	parda
E89	2022-01-09	SIM	27	Sao_Paulo_-	SP	Sudeste	Masculino	parda
E90	2022-01-09	SIM	27	Lajeado-_RS	RS	Sul	Masculino	Branco
E91	2022-01-10	SIM	27	Recife_PE	PE	Nordeste	Masculino	Branco
E92	2021-02-06	SIM	28	Japi_RN	RN	Norte	Feminino	parda
E93	2021-02-07	SIM	28	Inhumas-_G(	GO	Centro-Oeste	Masculino	parda
E94	2021-02-10	SIM	28	Sao_Paulo-S	SP	Sudeste	Feminino	Branco
E95	2021-02-20	SIM	28	Sao_Paulo	SP	Sudeste	Feminino	Amarelo
E96	2021-02-23	SIM	28	Sao_Carlos-	SP	Sudeste	Feminino	Branco
E97	2021-03-11	SIM	28	Ceres-GO	GO	Centro-Oeste	Masculino	Branco
E98	2021-03-19	SIM	28	Sao_paulo_s	SP	Sudeste	Feminino	Branco
E99	2021-03-20	SIM	28	Ribeirao_Pre	SP	Sudeste	Feminino	Branco
E100	2021-04-24	SIM	28	Curitiba-_Pa	PR	Sul	Masculino	Branco
E101	2021-04-24	SIM	28	Rio_de_Jane	RJ	Sudeste	Feminino	Branco
E102	2021-04-24	SIM	28	Goiania_Goia	GO	Centro-Oeste	Masculino	Branco
E103	2021-04-25	SIM	28	Vinhedo	SP	Sudeste	Masculino	Amarelo
E104	2021-05-23	SIM	28	GOIaNIA_-_(	GO	Centro-Oeste	Masculino	Branco
E105	2021-05-24	SIM	28	Brasilia_DF	DF	Distrito_Fede	Feminino	Ignorado
E106	2021-05-24	SIM	28	Goiania-_GC	GO	Centro-Oeste	Feminino	Branco
E107	2021-05-24	SIM	28	Goiania-_Go	GO	Centro-Oeste	Feminino	parda
E108	2021-05-24	SIM	28	Goiania-GO	GO	Centro-Oeste	Feminino	Branco
E109	2021-05-26	SIM	28	Sao_Paulo_S	SP	Sudeste	Feminino	Branco
E110	2022-01-06	SIM	28	Sao_Paulo_-	SP	Sudeste	Feminino	Branco
E111	2022-01-09	SIM	28	Sao_Bento_(	SC	Sul	Masculino	Branco
E112	2022-01-10	SIM	28	Maracanau-(	CE	Nordeste	Masculino	parda
E113	2022-01-24	SIM	28	GOIaNIA	GO	Centro-Oeste	Masculino	Branco

E114	2021-02-06	SIM	29	Unerlandia-M	MG	Sudeste	Feminino	Branco
E115	2021-02-09	SIM	29	Sao_Carlos_	SP	Sudeste	Feminino	Branco
E116	2021-02-09	SIM	29	Sao_Carlos	SP	Sudeste	Feminino	Amarelo
E117	2021-02-09	SIM	29	Aparecida_d	GO	Centro-Oeste	Masculino	Branco
E118	2021-02-09	SIM	29	Aparecida_d	GO	Centro-Oeste	Masculino	parda
E119	2021-02-10	SIM	29	Goiania	GO	Centro-Oeste	Masculino	Branco
E120	2021-02-20	SIM	29	Sao_Paulo	SP	Sudeste	Feminino	Branco
E121	2021-03-10	SIM	29	Goiania_-_G	GO	Centro-Oeste	Feminino	parda
E122	2021-03-19	SIM	29	Aparecida_d	GO	Centro-Oeste	Feminino	Branco
E123	2021-03-20	SIM	29	Sao_Carlos_	SP	Sudeste	Masculino	Branco
E124	2021-03-20	SIM	29	Cariacica-ES	ES	Sudeste	Masculino	parda
E125	2021-03-23	SIM	29	Goiania_e_g	GO	Centro-Oeste	Feminino	Branco
E126	2021-03-23	SIM	29	Vitoria_ES	ES	Sudeste	Feminino	Branco
E127	2021-04-23	SIM	29	Goiania	GO	Centro-Oeste	Masculino	Branco
E128	2021-04-24	SIM	29	Sao_Luis_-_	MA	Nordeste	Feminino	Branco
E129	2021-04-24	SIM	29	Feira_de_Sa	BA	Nordeste	Masculino	parda
E130	2021-04-24	SIM	29	Sao_Paulo	SP	Sudeste	Masculino	Branco
E131	2021-05-24	SIM	29	Goiania-_Go	GO	Centro-Oeste	Feminino	Preto
E132	2021-05-24	SIM	29	Goiania	GO	Centro-Oeste	Masculino	Branco
E133	2021-05-24	SIM	29	Goiania-_Go	GO	Centro-Oeste	Feminino	Branco
E134	2021-05-24	SIM	29	Goiania-GO	GO	Centro-Oeste	Feminino	Branco
E135	2021-05-26	SIM	29	Sao_Carlos_	SP	Sudeste	Feminino	Branco
E136	2021-07-06	SIM	29	ARARAQUA	SP	Sudeste	Feminino	Branco
E137	2022-01-08	SIM	29	Campo_Limp	SP	Sudeste	Feminino	Branco
E138	2022-01-08	SIM	29	Feira_de_Sa	BA	Nordeste	Masculino	parda
E139	2022-01-08	SIM	29	Rio_Tinto_Pa	PB	Nordeste	Masculino	Branco
E140	2022-01-10	SIM	29	Ponta_Gross	PR	Sul	Feminino	Branco
E141	2021-02-08	SIM	30	Sao_Carlos-_	SP	Sudeste	Masculino	Branco
E142	2021-02-09	SIM	30	Sao_Carlos-_	SP	Sudeste	Feminino	Branco
E143	2021-02-10	SIM	30	Aruja-SP	SP	Sudeste	Feminino	Branco
E144	2021-02-17	SIM	30	Sao_Carlos_	SP	Sudeste	Feminino	Branco
E145	2021-02-23	SIM	30	Sao_Carlos_	SP	Sudeste	Masculino	Amarelo
E146	2021-04-15	SIM	30	Barra_do_Ga	MT	Centro-Oeste	Masculino	Branco
E147	2021-04-21	SIM	30	Goiania-Goia	GO	Centro-Oeste	Feminino	parda
E148	2021-04-23	SIM	30	DF	DF	Distrito_Fede	Masculino	parda
E149	2021-04-24	SIM	30	buri-_sp	SP	Sudeste	Masculino	Branco
E150	2021-05-20	SIM	30	Cataguases_	MG	Sudeste	Masculino	Branco
E151	2021-05-23	SIM	30	Sao_Paulo-_	SP	Sudeste	Feminino	parda
E152	2021-05-26	SIM	30	Goiania-Go	GO	Centro-Oeste	Masculino	Branco
E153	2021-05-26	SIM	30	Betim_-_MG	MG	Sudeste	Feminino	parda
E154	2021-05-27	SIM	30	Bauru-SP	SP	Sudeste	Masculino	Branco
E155	2021-05-28	SIM	30	Campinas-sp	SP	Sudeste	Feminino	Branco
E156	2021-07-06	SIM	30	Aruja-_SP	SP	Sudeste	Feminino	Branco
E157	2022-01-05	SIM	30	Santo_Andre	SP	Sudeste	Masculino	Branco
E158	2022-01-08	SIM	30	Caraguatatul	SP	Sudeste	Masculino	Branco
E159	2022-01-09	SIM	30	SaO_PAULO	SP	Sudeste	Masculino	Branco
E160	2022-01-09	SIM	30	RJ	RJ	Sudeste	Masculino	Preto
E161	2022-01-09	SIM	30	Brasilia-DF	DF	Distrito_Fede	Masculino	Preto
E162	2022-01-09	SIM	30	Campinas-_S	SP	Sudeste	Masculino	Branco
E163	2021-02-09	SIM	31	Uberlandia_-	MG	Sudeste	Masculino	Branco
E164	2021-03-19	SIM	31	Goiania	GO	Centro-Oeste	Feminino	Branco
E165	2021-03-19	SIM	31	Sao_Paulo	SP	Sudeste	Feminino	Branco
E166	2021-04-23	SIM	31	Vitoria	ES	Sudeste	Masculino	Branco
E167	2021-04-24	SIM	31	Sao_Paulo	SP	Sudeste	Masculino	parda
E168	2021-04-24	SIM	31	Pinhais	PR	Sul	Masculino	parda
E169	2021-05-24	SIM	31	Sao_Paulo-_	SP	Sudeste	Feminino	Branco
E170	2021-05-25	SIM	31	Vila_Velha_E	ES	Sudeste	Feminino	Branco
E171	2022-01-06	SIM	31	Sao_Paulo_s	SP	Sudeste	Feminino	Branco

E172	2022-01-08	SIM	31	Pedreira_SP	SP	Sudeste	Feminino	Branco
E173	2022-01-08	SIM	31	Porto_Alegre	RS	Sul	Masculino	Branco
E174	2022-01-09	SIM	31	Braganca_-_	SP	Sudeste	Masculino	Branco
E175	2021-02-06	SIM	32	SAO_PAULC	SP	Sudeste	Feminino	Branco
E176	2021-02-09	SIM	32	Barueri_SP	SP	Sudeste	Feminino	Branco
E177	2021-02-09	SIM	32	Sao_Paulo-_	SP	Sudeste	Masculino	Branco
E178	2021-02-09	SIM	32	Sao_Paulo-S	SP	Sudeste	Masculino	Amarelo
E179	2021-02-21	SIM	32	Sacramento-	MG	Sudeste	Feminino	Branco
E180	2021-02-26	SIM	32	Sao_Carlos_	SP	Sudeste	Feminino	Branco
E181	2021-03-19	SIM	32	Porto_Velho_	RO	Norte	Feminino	parda
E182	2021-04-23	SIM	32	Vitoria_ES	ES	Sudeste	Feminino	Branco
E183	2021-04-24	SIM	32	Brasilia-DF	DF	Distrito_Fede	Masculino	parda
E184	2021-04-24	SIM	32	Guararema-_	SP	Sudeste	Masculino	Branco
E185	2021-04-25	SIM	32	Maceio_alag	AL	Nordeste	Masculino	Ignorado
E186	2021-05-23	SIM	32	Goiania-_goi	GO	Centro-Oeste	Feminino	Preto
E187	2021-05-24	SIM	32	Aparecida_d	GO	Centro-Oeste	Masculino	parda
E188	2021-05-24	SIM	32	Aparecida_d	GO	Centro-Oeste	Feminino	Branco
E189	2021-05-28	SIM	32	Sao_Jose_d	SP	Sudeste	Feminino	Branco
E190	2022-01-09	SIM	32	Goiania_GO	GO	Centro-Oeste	Masculino	parda
E191	2022-01-11	SIM	32	Rio_de_Jane	RJ	Sudeste	Masculino	Branco
E192	2021-03-19	SIM	33	Santos-SP	SP	Sudeste	Masculino	Branco
E193	2021-04-25	SIM	33	Curitiba_Par	PR	Sul	Feminino	Branco
E194	2021-04-26	SIM	33	Sorocaba-sp	SP	Sudeste	Feminino	Branco
E195	2021-04-26	SIM	33	Tres_lagoas_	MS	Centro-Oeste	Feminino	Branco
E196	2021-05-29	SIM	33	PAULINIA_-_	SP	Sudeste	Masculino	parda
E197	2021-06-24	SIM	33	Rondonopoli	MT	Centro-Oeste	Feminino	Branco
E198	2021-06-24	SIM	33	Sao_Paulo_-	SP	Sudeste	Feminino	Branco
E199	2022-01-05	SIM	33	Igrejinha-_RS	RS	Sul	Masculino	Branco
E200	2022-01-08	SIM	33	Rio_de_Jane	RJ	Sudeste	Masculino	Branco
E201	2022-01-09	SIM	33	Sao_Paulo-_	SP	Sudeste	Feminino	Branco
E202	2022-01-10	SIM	33	sao_paulo_-_	SP	Sudeste	Feminino	Branco
E203	2022-01-10	SIM	33	Mogi_das_C	SP	Sudeste	Masculino	Amarelo
E204	2022-01-10	SIM	33	Sorocaba	SP	Sudeste	Masculino	parda
E205	2021-02-08	SIM	34	Sao_Carlos_	SP	Sudeste	Masculino	Preto
E206	2021-02-09	SIM	34	Piracicaba	SP	Sudeste	Feminino	Branco
E207	2021-03-06	SIM	34	Botucatu-SP	SP	Sudeste	Feminino	Preto
E208	2021-03-19	SIM	34	Goiania-Goia	GO	Centro-Oeste	Feminino	parda
E209	2021-03-29	SIM	34	Piumhi-MG	MG	Sudeste	Masculino	Branco
E210	2021-04-19	SIM	34	Sao_Carlos-S	SP	Sudeste	Feminino	Branco
E211	2021-04-23	SIM	34	Contagem_M	MG	Sudeste	Masculino	Branco
E212	2021-04-24	SIM	34	Salvador-BA	BA	Nordeste	Masculino	Ignorado
E213	2021-04-24	SIM	34	Sao_Paulo_-	SP	Sudeste	Feminino	parda
E214	2021-05-23	SIM	34	Goiania	GO	Centro-Oeste	Feminino	Branco
E215	2021-05-24	SIM	34	Anapolis-_G	GO	Centro-Oeste	Feminino	parda
E216	2022-01-06	SIM	34	Rio_de_Jane	RJ	Sudeste	Masculino	Branco
E217	2022-01-09	SIM	34	Ananindeua-	PA	Nordeste	Masculino	Preto
E218	2022-01-09	SIM	34	Gama_-_DF	DF	Distrito_Fede	Masculino	parda
E219	2021-02-23	SIM	35	Alpinopolis_-	MG	Sudeste	Feminino	parda
E220	2021-03-19	SIM	35	Goiania_Goia	GO	Centro-Oeste	Feminino	Branco
E221	2021-04-23	SIM	35	Paulistana	PI	Nordeste	Masculino	parda
E222	2021-04-24	SIM	35	Sao_Carlos_	SP	Sudeste	Masculino	Branco
E223	2021-04-25	SIM	35	Sao_Paulo-S	SP	Sudeste	Feminino	Branco
E224	2021-05-24	SIM	35	Goiania_	GO	Centro-Oeste	Feminino	parda
E225	2021-05-24	SIM	35	Sao_Carlos-S	SP	Sudeste	Feminino	Branco
E226	2021-06-12	SIM	35	Sao_Carlos-S	SP	Sudeste	Feminino	parda
E227	2021-06-24	SIM	35	Sao_Paulo_-	SP	Sudeste	Feminino	Branco
E228	2022-01-10	SIM	35	Mogi_Guacu	SP	Sudeste	Masculino	Branco
E229	2022-01-10	SIM	35	Sao_Paulo-S	SP	Sudeste	Masculino	Branco

E230	2021-02-06	SIM	36	Sao_Carlos-	SP	Sudeste	Feminino	Branco
E231	2021-02-08	SIM	36	Ribeirao_Pre	SP	Sudeste	Feminino	Branco
E232	2021-02-23	SIM	36	Rio_Claro	SP	Sudeste	Feminino	Branco
E233	2021-03-22	SIM	36	Toledo-_Par	PR	Sul	Feminino	Branco
E234	2021-04-22	SIM	36	Goiania	GO	Centro-Oest	Feminino	Amarelo
E235	2021-04-26	SIM	36	Sao_Paulo_-	SP	Sudeste	Feminino	parda
E236	2021-05-23	SIM	36	Campinas-s	SP	Sudeste	Feminino	Branco
E237	2021-05-23	SIM	36	Rio_de_Jane	RJ	Sudeste	Masculino	Branco
E238	2021-05-31	SIM	36	Sao_Carlos	SP	Sudeste	Feminino	Branco
E239	2021-06-24	SIM	36	Sao_Paulo-	SP	Sudeste	Masculino	Branco
E240	2022-01-08	SIM	36	GUARUJa_	SP	Sudeste	Masculino	Branco
E241	2022-01-08	SIM	36	Curitiba_PR	PR	Sul	Masculino	parda
E242	2022-01-09	SIM	36	Cabo_Frio_-	RJ	Sudeste	Feminino	Branco
E243	2022-01-10	SIM	36	Florianopolis	SC	Sul	Masculino	Branco
E244	2021-02-08	SIM	37	Sao_Carlos	SP	Sudeste	Masculino	Branco
E245	2021-02-09	SIM	37	Nova_Resen	MG	Sudeste	Feminino	parda
E246	2021-02-09	SIM	37	Sao_Carlos	SP	Sudeste	Feminino	Branco
E247	2021-02-09	SIM	37	Sao_Carlos_	SP	Sudeste	Feminino	Branco
E248	2021-03-19	SIM	37	Porto_Velho-	RO	Norte	Masculino	Indigeno
E249	2021-05-25	SIM	37	Sao_Paulo-	SP	Sudeste	Feminino	Branco
E250	2021-05-28	SIM	37	Sao_Jose_d	SP	Sudeste	Feminino	Branco
E251	2022-01-08	SIM	37	Campos_dos	RJ	Sudeste	Masculino	Branco
E252	2021-02-08	SIM	38	Araraquara_	SP	Sudeste	Feminino	Preto
E253	2021-03-19	SIM	38	GOIANIA	GO	Centro-Oest	Feminino	Branco
E254	2021-03-22	SIM	38	Vitoria	ES	Sudeste	Feminino	parda
E255	2021-04-24	SIM	38	Uberlandia-	MG	Sudeste	Masculino	parda
E256	2021-04-24	SIM	38	Goiania_GO	GO	Centro-Oest	Masculino	Branco
E257	2021-05-21	SIM	38	Cataguases-	MG	Sudeste	Masculino	parda
E258	2021-06-24	SIM	38	Santo_Andre	SP	Sudeste	Feminino	Branco
E259	2022-01-09	SIM	38	Porto_Alegre	RS	Sul	Masculino	Branco
E260	2021-02-09	SIM	39	Jaragua_do_	SC	Sul	Masculino	Branco
E261	2021-03-20	SIM	39	Araraquara_	SP	Sudeste	Feminino	Branco
E262	2021-03-22	SIM	39	Rio_de_Jane	RJ	Sudeste	Feminino	Branco
E263	2021-04-24	SIM	39	Sao_Paulo_	SP	Sudeste	Masculino	Branco
E264	2021-05-24	SIM	39	Goiania-_GC	GO	Centro-Oest	Masculino	Branco
E265	2021-05-27	SIM	39	Sao_Jose_d	SP	Sudeste	Feminino	parda
E266	2021-06-24	SIM	39	sao_paulo-_	SP	Sudeste	Feminino	Branco
E267	2022-01-08	SIM	39	Joao_Pesso	PB	Nordeste	Masculino	parda
E268	2021-04-01	SIM	40	Recife_-_Pe	PE	Nordeste	Feminino	Preto
E269	2021-04-23	SIM	40	Rio_das_Ost	RJ	Sudeste	Masculino	Ignorado
E270	2021-04-24	SIM	40	Sao_Paulo	SP	Sudeste	Masculino	Amarelo
E271	2021-05-05	SIM	40	Sao_carlos-	SP	Sudeste	Feminino	Branco
E272	2021-05-23	SIM	40	Goiania_GO	GO	Centro-Oest	Feminino	Branco
E273	2021-05-28	SIM	40	SaO_JOSe_	SP	Sudeste	Feminino	Branco
E274	2022-01-06	SIM	40	Belo_Horizor	MG	Sudeste	Masculino	Branco
E275	2021-02-08	SIM	41	SaO_CARLC	SP	Sudeste	Masculino	Branco
E276	2021-02-20	SIM	41	Sao_Carlos	SP	Sudeste	Feminino	Branco
E277	2021-03-19	SIM	41	Florianopolis	SC	Sul	Feminino	Branco
E278	2021-04-08	SIM	41	Aparecida_d	GO	Centro-Oest	Feminino	Branco
E279	2021-04-24	SIM	41	Sao_Paulo	SP	Sudeste	Masculino	Amarelo
E280	2021-05-28	SIM	41	Sao_Jose_d	SP	Sudeste	Feminino	Branco
E281	2021-02-09	SIM	42	Florianopolis	SC	Sul	Feminino	Branco
E282	2021-05-24	SIM	42	Goiania	GO	Centro-Oest	Masculino	Branco
E283	2021-05-25	SIM	42	Goiania-_Go	GO	Centro-Oest	Feminino	parda
E284	2021-05-27	SIM	42	Brasilia-DF	DF	Distrito_Fed	Masculino	Branco
E285	2022-01-06	SIM	42	Piranguinho-	MG	Sudeste	Feminino	Preto
E286	2021-02-10	SIM	43	Goiania	GO	Centro-Oest	Feminino	Ignorado
E287	2021-02-23	SIM	43	Sao_Carlos_	SP	Sudeste	Masculino	Branco

E288	2021-03-20	SIM	43	Araraquara	SP	Sudeste	Feminino	Preto
E289	2021-03-24	SIM	43	Rio_de_Jane	RJ	Sudeste	Masculino	Branco
E290	2021-05-01	SIM	43	RJ	RJ	Sudeste	Feminino	Branco
E291	2021-05-28	SIM	43	Sao_Jose_d	SP	Sudeste	Feminino	parda
E292	2022-01-09	SIM	43	Praia_Grand	SP	Sudeste	Masculino	Branco
E293	2021-02-08	SIM	44	Sao_Carlos-:	SP	Sudeste	Feminino	Branco
E294	2021-02-22	SIM	44	Sao_Carlos-_	SP	Sudeste	Feminino	Amarelo
E295	2021-03-19	SIM	44	Descalvado_	SP	Sudeste	Feminino	Branco
E296	2021-04-27	SIM	44	Ribeirao_Pre	SP	Sudeste	Feminino	Branco
E297	2021-05-28	SIM	44	Salvador_Ba	BA	Nordeste	Feminino	Branco
E298	2021-07-07	SIM	44	Marilia	SP	Sudeste	Feminino	Branco
E299	2021-02-25	SIM	45	Salvador-Ba	BA	Nordeste	Masculino	parda
E300	2021-02-26	SIM	45	Sao_Carlos-_	SP	Sudeste	Feminino	Branco
E301	2021-07-08	SIM	45	MARiLIA-SP	SP	Sudeste	Masculino	parda
E302	2021-02-22	SIM	46	Novo_Hambı	RS	Sul	Masculino	Branco
E303	2021-03-30	SIM	46	Maceio_AL	AL	Nordeste	Feminino	Branco
E304	2021-07-07	SIM	46	Sao_Carlos	SP	Sudeste	Feminino	Branco
E305	2021-03-19	SIM	47	Goiania_	GO	Centro-Oeste	Feminino	parda
E306	2021-03-22	SIM	47	Sao_Paulo	SP	Sudeste	Feminino	Branco
E307	2021-04-24	SIM	47	Belo_Horizor	MG	Sudeste	Masculino	Branco
E308	2021-03-29	SIM	48	Maceio	AL	Nordeste	Masculino	Branco
E309	2021-04-23	SIM	48	Petropolis-R.	RJ	Sudeste	Masculino	Branco
E310	2021-03-19	SIM	49	Goiania-Go	GO	Centro-Oeste	Feminino	Branco
E311	2021-05-28	SIM	49	Sao_Jose_C	SP	Sudeste	Feminino	Branco
E312	2021-07-07	SIM	49	Sao_Carlos	SP	Sudeste	Feminino	Branco
E313	2021-03-10	SIM	50	Goiania	GO	Centro-Oeste	Feminino	Branco
E314	2022-01-09	SIM	50	maringa=PR	PR	Sul	Masculino	Branco
E315	2021-02-08	SIM	51	Sao_Carlos_	SP	Sudeste	Feminino	Branco
E316	2021-02-10	SIM	51	Piracicaba-_:	SP	Sudeste	Masculino	Branco
E317	2021-05-27	SIM	51	Goiania_GO	GO	Centro-Oeste	Masculino	Branco
E318	2021-07-06	SIM	54	Sao_Carlos_	SP	Sudeste	Feminino	Branco
E319	2021-02-20	SIM	55	Lins	SP	Sudeste	Feminino	Amarelo
E320	2021-02-08	SIM	56	Sao_Carlos_	SP	Sudeste	Masculino	Branco
E321	2021-02-08	SIM	56	Araraquara_:	SP	Sudeste	Feminino	Branco
E322	2021-05-27	SIM	56	Sao_Paulo	SP	Sudeste	Masculino	Branco
E323	2021-05-27	SIM	56	Goiania_	GO	Centro-Oeste	Masculino	Branco
E324	2021-02-08	SIM	57	Sao_Carlos-:	SP	Sudeste	Feminino	Branco
E325	2021-02-20	SIM	57	Araraquara-S	SP	Sudeste	Masculino	Amarelo
E326	2021-03-10	SIM	57	Curitibanos-S	SC	Sul	Feminino	Branco
E327	2021-03-19	SIM	58	Sao_Paulo	SP	Sudeste	Feminino	parda
E328	2021-07-06	SIM	61	Sao_Carlos_	SP	Sudeste	Feminino	Branco

bitos_de_vida_e_caracteristicas_do_trabalho_remoto__

estado_civil	renda_famil	renda_famil	escolaridad	ocupa	tem_filhos	com_quem_	praticava_at	praticou_ati
Solteiro_a	mais_de_10_	mais_de_10_	ensino_supe	Relaco	nao	Com_2_ou_r	Sim	Nao
Solteiro_a	de_1_a_4_sa	de_1_a_4_sa	ensino_medi	Estuda	nao	Com_uma_p	Nao	Sim
Solteiro_a	de_1_a_4_sa	de_1_a_4_sa	ensino_supe	Estuda	nao	Com_2_ou_r	Sim	Sim
Solteiro_a	de_1_a_4_sa	de_1_a_4_sa	ensino_supe	Design	nao	Sozinho	Nao	Nao
Vive_com_co	de_1_a_4_sa	de_1_a_4_sa	ensino_supe	Publicit	nao	Com_uma_p	Sim	Nao
Solteiro_a	de_1_a_4_sa	de_1_a_4_sa	ensino_supe	Garcon	nao	Com_2_ou_r	Nao	Sim
Solteiro_a	de_1_a_4_sa	de_1_a_4_sa	ensino_supe	Estuda	nao	Com_uma_p	Nao	Nao
Solteiro_a	de_1_a_4_sa	de_1_a_4_sa	ensino_supe	Analista	nao	Sozinho	Nao	Sim
Vive_com_co	de_1_a_4_sa	de_1_a_4_sa	ensino_supe	Analista	nao	Com_uma_p	Nao	Sim
Solteiro_a	de_4_a_10_s	de_4_a_10_s	ensino_supe	Servido	nao	Com_2_ou_r	Nao	Sim
Solteiro_a	de_4_a_10_s	de_4_a_10_s	ensino_supe	Prograr	nao	Com_2_ou_r	Nao	Nao
Solteiro_a	mais_de_10_	mais_de_10_	ensino_supe	Psicolo	nao	Com_2_ou_r	Sim	Sim
Vive_com_co	de_1_a_4_sa	de_4_a_10_s	ensino_supe	NULL	nao	Com_uma_p	Sim	Sim
Solteiro_a	de_4_a_10_s	de_4_a_10_s	ensino_supe	Profess	nao	Com_2_ou_r	Nao	Nao
Solteiro_a	de_4_a_10_s	mais_de_10_	ensino_supe	NULL	nao	Com_2_ou_r	Nao	Nao
Solteiro_a	mais_de_10_	mais_de_10_	ensino_supe	Analista	nao	Com_2_ou_r	Sim	Nao
Vive_com_co	de_1_a_4_sa	mais_de_4_s	ensino_supe	Psicolo	nao	Com_uma_p	Nao	Sim
Solteiro_a	de_4_a_10_s	mais_de_10_	ensino_supe	Analista	nao	Com_2_ou_r	Sim	Nao
Solteiro_a	mais_de_4_s	de_1_a_4_sa	ensino_supe	Fisioter	nao	Com_2_ou_r	Nao	Sim
Vive_com_co	mais_de_10_	mais_de_10_	ensino_supe	Empres	nao	Com_uma_p	Sim	Sim
Solteiro_a	ate_um_sala	ate_um_sala	ensino_supe	Redato	nao	Com_2_ou_r	Nao	Nao
Solteiro_a	de_1_a_4_sa	mais_de_4_s	ensino_supe	Prograr	nao	Com_2_ou_r	Nao	Sim
Casado_a	mais_de_10_	de_4_a_10_s	ensino_supe	Fisioter	nao	Com_uma_p	Sim	Sim
Vive_com_co	de_4_a_10_s	de_4_a_10_s	ensino_supe	Estagia	nao	Com_uma_p	Sim	Sim
Solteiro_a	de_1_a_4_sa	de_1_a_4_sa	ensino_supe	Advoga	nao	Com_2_ou_r	Nao	Nao
Solteiro_a	de_1_a_4_sa	de_1_a_4_sa	ensino_supe	Estagia	nao	Com_uma_p	Sim	Nao
Solteiro_a	de_1_a_4_sa	de_1_a_4_sa	ensino_supe	Engenh	nao	Sozinho	Sim	Nao
Solteiro_a	mais_de_10_	mais_de_10_	ensino_supe	Cirurgia	nao	Com_2_ou_r	Sim	Nao
Solteiro_a	ate_um_sala	de_1_a_4_sa	ensino_supe	Pos_gr	nao	Com_2_ou_r	Nao	Nao
Vive_com_co	mais_de_10_	de_4_a_10_s	ensino_supe	Engenh	nao	Com_uma_p	Sim	Sim
Vive_com_co	mais_de_10_	mais_de_10_	ensino_supe	Cientist	nao	Com_uma_p	Sim	Nao
Solteiro_a	de_1_a_4_sa	de_4_a_10_s	ensino_supe	Analista	nao	Com_2_ou_r	Sim	Nao
Solteiro_a	de_4_a_10_s	mais_de_4_s	ensino_supe	Advoga	nao	Com_2_ou_r	Sim	Nao
Solteiro_a	de_4_a_10_s	de_1_a_4_sa	ensino_supe	Desenv	nao	Com_2_ou_r	Sim	Sim
Solteiro_a	de_1_a_4_sa	de_1_a_4_sa	ensino_supe	Adminis	nao	Com_uma_p	Nao	Nao
Solteiro_a	de_1_a_4_sa	de_4_a_10_s	ensino_supe	Analista	nao	Com_2_ou_r	Sim	Sim
Casado_a	de_4_a_10_s	de_4_a_10_s	ensino_supe	Engenh	nao	Com_uma_p	Nao	Nao
Solteiro_a	de_4_a_10_s	de_4_a_10_s	ensino_supe	Estuda	nao	Com_2_ou_r	Sim	Nao
Solteiro_a	de_1_a_4_sa	de_1_a_4_sa	ensino_supe	Jornalis	nao	Com_uma_p	Sim	Nao
Solteiro_a	de_1_a_4_sa	mais_de_10_	ensino_supe	Desenv	nao	Sozinho	Sim	Sim
Solteiro_a	de_4_a_10_s	de_4_a_10_s	ensino_supe	Estagia	nao	Com_2_ou_r	Sim	Nao
Solteiro_a	de_1_a_4_sa	de_1_a_4_sa	ensino_supe	Prograr	nao	Sozinho	Sim	Sim
Vive_com_co	de_1_a_4_sa	de_4_a_10_s	ensino_supe	Profess	nao	Com_uma_p	Sim	Nao
Solteiro_a	de_4_a_10_s	de_4_a_10_s	ensino_supe	Advoga	nao	Com_2_ou_r	Sim	Nao
Vive_com_co	de_1_a_4_sa	de_1_a_4_sa	ensino_supe	Advoga	nao	Com_uma_p	Sim	Sim
Vive_com_co	de_1_a_4_sa	de_4_a_10_s	ensino_supe	Engenh	nao	Com_uma_p	Sim	Sim
Solteiro_a	de_4_a_10_s	de_4_a_10_s	ensino_supe	Engenh	nao	Com_2_ou_r	Sim	Nao
Solteiro_a	mais_de_4_s	mais_de_4_s	ensino_supe	Pos_gr	nao	Com_uma_p	Nao	Sim
Solteiro_a	de_4_a_10_s	de_4_a_10_s	ensino_supe	Estuda	nao	Com_2_ou_r	Sim	Sim
Solteiro_a	mais_de_4_s	mais_de_4_s	ensino_supe	Servido	nao	Sozinho	Sim	Nao
Solteiro_a	de_4_a_10_s	de_4_a_10_s	ensino_supe	Cinegra	nao	Com_2_ou_r	Sim	Sim
Solteiro_a	de_1_a_4_sa	de_1_a_4_sa	ensino_supe	Profess	nao	Sozinho	Sim	Sim
Solteiro_a	de_1_a_4_sa	de_1_a_4_sa	ensino_supe	Estuda	nao	Com_uma_p	Nao	Sim
Solteiro_a	de_1_a_4_sa	de_1_a_4_sa	ensino_supe	Tatuad	nao	Sozinho	Sim	Nao
Solteiro_a	de_4_a_10_s	de_4_a_10_s	ensino_supe	Engenh	nao	Com_2_ou_r	Sim	Sim

Vive_com_cc	de_4_a_10_s	de_4_a_10_s	ensino_supe	Psicolo	nao	Com_uma_p	Nao	Sim
Solteiro_a	de_1_a_4_sa	de_4_a_10_s	ensino_supe	Person	nao	Com_2_ou_r	Sim	Sim
Solteiro_a	mais_de_4_s	de_1_a_4_sa	ensino_supe	Engenh	nao	Com_uma_p	Sim	Nao
Solteiro_a	de_4_a_10_s	de_4_a_10_s	ensino_supe	Prograr	nao	Com_uma_p	Sim	Sim
Solteiro_a	de_4_a_10_s	de_4_a_10_s	ensino_supe	Desenv	nao	Com_2_ou_r	Nao	Nao
Solteiro_a	mais_de_4_s	mais_de_4_s	ensino_supe	Engenh	nao	Com_uma_p	Sim	Sim
Solteiro_a	de_4_a_10_s	de_4_a_10_s	ensino_supe	Militar	nao	Sozinho	Sim	Sim
Casado_a	de_4_a_10_s	de_4_a_10_s	ensino_supe	Servidc	nao	Com_uma_p	Nao	Nao
Solteiro_a	de_1_a_4_sa	de_1_a_4_sa	ensino_supe	Farmac	nao	Com_2_ou_r	Sim	Nao
Casado_a	de_1_a_4_sa	de_1_a_4_sa	ensino_supe	Analista	nao	Com_uma_p	Nao	Nao
Casado_a	de_1_a_4_sa	de_1_a_4_sa	ensino_supe	Fisioter	nao	Com_uma_p	Nao	Sim
Solteiro_a	de_1_a_4_sa	de_4_a_10_s	ensino_supe	Engenh	nao	Com_2_ou_r	Sim	Nao
Vive_com_cc	de_1_a_4_sa	mais_de_10	ensino_supe	Analista	nao	Com_uma_p	Nao	Sim
Vive_com_cc	de_1_a_4_sa	de_4_a_10_s	ensino_supe	Analista	nao	Com_uma_p	Nao	Sim
Vive_com_cc	de_4_a_10_s	de_4_a_10_s	ensino_supe	Empres	nao	Com_uma_p	Sim	Sim
Solteiro_a	de_4_a_10_s	de_4_a_10_s	ensino_supe	NULL	nao	Com_uma_p	Sim	Sim
Solteiro_a	mais_de_10	mais_de_10	ensino_supe	Advoga	nao	Com_uma_p	Nao	Nao
Solteiro_a	de_1_a_4_sa	de_1_a_4_sa	ensino_supe	NULL	nao	Com_2_ou_r	Nao	Nao
Solteiro_a	de_4_a_10_s	de_4_a_10_s	ensino_supe	Engenh	nao	Com_uma_p	Sim	Nao
Vive_com_cc	de_1_a_4_sa	de_1_a_4_sa	ensino_supe	Advoga	nao	Com_uma_p	Sim	Sim
Solteiro_a	de_1_a_4_sa	de_1_a_4_sa	ensino_supe	Advoga	nao	Com_2_ou_r	Nao	Sim
Vive_com_cc	de_4_a_10_s	de_4_a_10_s	ensino_supe	Analista	nao	Com_uma_p	Nao	Nao
Solteiro_a	mais_de_4_s	de_1_a_4_sa	ensino_supe	Psicolo	nao	Com_2_ou_r	Nao	Sim
Casado_a	mais_de_4_s	mais_de_4_s	ensino_supe	Servidc	nao	Com_uma_p	Nao	Sim
Solteiro_a	de_1_a_4_sa	de_1_a_4_sa	ensino_supe	Profess	nao	Com_uma_p	Sim	Sim
Solteiro_a	de_1_a_4_sa	de_1_a_4_sa	ensino_supe	#REF!	nao	Com_2_ou_r	Sim	Nao
Vive_com_cc	mais_de_10	mais_de_10	ensino_supe	Profissi	sim	Com_2_ou_r	Sim	Nao
Solteiro_a	mais_de_10	mais_de_10	ensino_supe	Profess	nao	Com_2_ou_r	Sim	Sim
Solteiro_a	de_1_a_4_sa	de_1_a_4_sa	ensino_supe	Pesquis	sim	Com_2_ou_r	Nao	Nao
Solteiro_a	de_1_a_4_sa	de_4_a_10_s	ensino_supe	Advoga	nao	Com_2_ou_r	Sim	Nao
Solteiro_a	de_1_a_4_sa	de_1_a_4_sa	ensino_supe	Relaco	sim	Com_2_ou_r	Sim	Sim
Solteiro_a	de_1_a_4_sa	de_1_a_4_sa	ensino_supe	Profess	nao	Com_uma_p	Nao	Sim
Solteiro_a	de_4_a_10_s	de_4_a_10_s	ensino_supe	NULL	nao	Com_uma_p	Sim	Sim
Solteiro_a	de_4_a_10_s	de_1_a_4_sa	ensino_supe	Design	nao	Sozinho	Sim	Nao
Vive_com_cc	mais_de_10	mais_de_10	ensino_supe	Adminis	nao	Com_uma_p	Nao	Nao
Solteiro_a	mais_de_10	mais_de_10	ensino_supe	Estudai	nao	Com_2_ou_r	Sim	Sim
Solteiro_a	de_1_a_4_sa	de_1_a_4_sa	ensino_supe	Fisioter	nao	Com_2_ou_r	Nao	Nao
Solteiro_a	mais_de_10	mais_de_10	ensino_supe	Engenh	nao	Com_2_ou_r	Sim	Sim
Solteiro_a	mais_de_10	de_4_a_10_s	ensino_supe	Trainee	nao	Com_uma_p	Sim	Nao
Solteiro_a	mais_de_10	mais_de_10	ensino_supe	Engenh	nao	Com_2_ou_r	Nao	Sim
Solteiro_a	ate_um_sala	ate_um_sala	ensino_supe	Profess	nao	Com_uma_p	Sim	Nao
Solteiro_a	mais_de_10	mais_de_10	ensino_supe	Profess	nao	Com_2_ou_r	Sim	Sim
Solteiro_a	de_4_a_10_s	de_4_a_10_s	ensino_supe	Farmac	nao	Com_uma_p	Sim	Sim
Vive_com_cc	mais_de_10	mais_de_10	ensino_supe	Psicolo	nao	Com_uma_p	Sim	Sim
Vive_com_cc	de_4_a_10_s	de_4_a_10_s	ensino_supe	Engenh	nao	Com_uma_p	Nao	Nao
Vive_com_cc	mais_de_4_s	de_1_a_4_sa	ensino_supe	Confeit	sim	Com_2_ou_r	Nao	Sim
Casado_a	de_4_a_10_s	de_4_a_10_s	ensino_supe	Profissi	nao	Com_uma_p	Nao	Nao
Casado_a	de_4_a_10_s	de_4_a_10_s	ensino_supe	Consult	nao	Com_uma_p	Nao	Nao
Solteiro_a	de_1_a_4_sa	de_1_a_4_sa	ensino_supe	Profess	nao	Sozinho	Nao	Sim
Casado_a	mais_de_10	mais_de_10	ensino_supe	Engenh	nao	Com_uma_p	Sim	Nao
Vive_com_cc	de_4_a_10_s	de_4_a_10_s	ensino_supe	Estudai	nao	Com_uma_p	Sim	Sim
Solteiro_a	de_4_a_10_s	de_4_a_10_s	ensino_supe	Prograr	nao	Sozinho	Sim	Nao
Casado_a	de_4_a_10_s	de_4_a_10_s	ensino_supe	Design	nao	Com_uma_p	Sim	Nao
Solteiro_a	mais_de_10	mais_de_10	ensino_supe	Sociolo	nao	Com_2_ou_r	Nao	Sim
Casado_a	de_4_a_10_s	mais_de_10	ensino_supe	NULL	nao	Com_uma_p	Sim	Nao
Solteiro_a	de_1_a_4_sa	de_1_a_4_sa	ensino_supe	Profess	nao	Com_uma_p	Sim	Sim
Casado_a	de_4_a_10_s	de_4_a_10_s	ensino_supe	NULL	nao	Com_uma_p	Sim	Sim
Solteiro_a	de_1_a_4_sa	de_1_a_4_sa	ensino_supe	Advoga	nao	Sozinho	Sim	Sim

Vive_com_cc	mais_de_4_s	mais_de_4_s	ensino_medi	Analista	nao	Com_uma_p	Nao	Nao
Solteiro_a	mais_de_10_	mais_de_4_s	ensino_supe	Farmac	nao	Com_uma_p	Sim	Sim
Solteiro_a	de_1_a_4_sa	de_1_a_4_sa	ensino_supe	Coord_	nao	Com_uma_p	Nao	Nao
Solteiro_a	mais_de_4_s	de_4_a_10_s	ensino_supe	Adminis	nao	Com_2_ou_r	Nao	Sim
Casado_a	de_4_a_10_s	de_4_a_10_s	ensino_supe	Advoga	nao	Com_uma_p	Sim	Sim
Casado_a	de_1_a_4_sa	de_1_a_4_sa	ensino_supe	Analista	sim	Com_2_ou_r	Sim	Nao
Solteiro_a	mais_de_4_s	de_1_a_4_sa	ensino_supe	Fisioter	nao	Com_uma_p	Sim	Sim
Solteiro_a	de_1_a_4_sa	de_1_a_4_sa	ensino_supe	Fisioter	nao	Com_uma_p	Nao	Sim
Solteiro_a	de_4_a_10_s	de_4_a_10_s	ensino_supe	Gerente	nao	Com_uma_p	Sim	Sim
Solteiro_a	mais_de_10_	mais_de_10_	ensino_supe	Consult	nao	Com_2_ou_r	Sim	Nao
Solteiro_a	de_4_a_10_s	de_4_a_10_s	ensino_supe	Assiste	nao	Com_uma_p	Nao	Nao
Vive_com_cc	mais_de_10_	mais_de_10_	ensino_supe	Psicolo	sim	Com_2_ou_r	Sim	Sim
Solteiro_a	de_1_a_4_sa	mais_de_4_s	ensino_supe	Profess	nao	Com_uma_p	Nao	Sim
Vive_com_cc	de_4_a_10_s	de_4_a_10_s	ensino_supe	Profess	sim	Com_2_ou_r	Sim	Sim
Vive_com_cc	de_4_a_10_s	de_4_a_10_s	ensino_supe	Biologo	nao	Com_uma_p	Sim	Sim
Solteiro_a	de_1_a_4_sa	de_1_a_4_sa	ensino_supe	Profess	nao	Sozinho	Nao	Nao
Solteiro_a	mais_de_4_s	mais_de_4_s	ensino_supe	CS	sim	Com_2_ou_r	Nao	Nao
Solteiro_a	de_1_a_4_sa	de_1_a_4_sa	ensino_supe	Pos_gr	nao	Com_2_ou_r	Sim	Sim
Casado_a	de_4_a_10_s	de_1_a_4_sa	ensino_supe	Jornalis	nao	Com_uma_p	Sim	Nao
Vive_com_cc	mais_de_10_	mais_de_10_	ensino_supe	Desenv	nao	Com_2_ou_r	Sim	Sim
Solteiro_a	de_4_a_10_s	mais_de_10_	ensino_supe	Advoga	nao	Sozinho	Nao	Sim
Casado_a	mais_de_10_	mais_de_10_	ensino_supe	Psicolo	nao	Com_uma_p	Nao	Nao
Solteiro_a	de_4_a_10_s	de_4_a_10_s	ensino_supe	Fisioter	nao	Com_2_ou_r	Sim	Sim
Solteiro_a	de_1_a_4_sa	de_1_a_4_sa	ensino_supe	NULL	nao	Com_uma_p	Nao	Sim
Solteiro_a	de_1_a_4_sa	de_1_a_4_sa	ensino_supe	Profess	nao	Sozinho	Nao	Nao
Solteiro_a	de_1_a_4_sa	de_1_a_4_sa	ensino_supe	Profess	nao	Com_2_ou_r	Sim	Sim
Solteiro_a	de_1_a_4_sa	de_1_a_4_sa	ensino_supe	NULL	sim	Com_uma_p	Sim	Nao
Solteiro_a	de_4_a_10_s	de_4_a_10_s	ensino_supe	Profess	nao	Com_2_ou_r	Nao	Nao
Casado_a	de_4_a_10_s	de_4_a_10_s	ensino_supe	Pesquis	nao	Com_uma_p	Sim	Nao
Casado_a	de_4_a_10_s	de_4_a_10_s	ensino_supe	Fisioter	nao	Com_uma_p	Nao	Nao
Solteiro_a	de_4_a_10_s	de_4_a_10_s	ensino_supe	Estudai	nao	Com_2_ou_r	Nao	Sim
Solteiro_a	de_1_a_4_sa	de_4_a_10_s	ensino_supe	Desenv	nao	Com_uma_p	Sim	Nao
Solteiro_a	mais_de_10_	mais_de_10_	ensino_supe	Adminis	nao	Com_2_ou_r	Sim	Sim
Casado_a	mais_de_4_s	de_1_a_4_sa	ensino_supe	Fisioter	sim	Com_2_ou_r	Sim	Sim
Solteiro_a	de_4_a_10_s	de_4_a_10_s	ensino_supe	Desenv	nao	Sozinho	Sim	Sim
Solteiro_a	de_4_a_10_s	de_4_a_10_s	ensino_supe	Servidc	nao	Com_uma_p	Nao	Nao
Solteiro_a	de_4_a_10_s	de_4_a_10_s	ensino_supe	Profess	nao	Com_2_ou_r	Sim	Sim
Casado_a	mais_de_10_	mais_de_10_	ensino_supe	Consult	nao	Com_uma_p	Sim	Nao
Casado_a	de_4_a_10_s	de_4_a_10_s	ensino_supe	Fisioter	nao	Com_uma_p	Sim	Sim
Casado_a	de_1_a_4_sa	de_4_a_10_s	ensino_supe	Profess	nao	Com_uma_p	Nao	Nao
Solteiro_a	mais_de_10_	mais_de_10_	ensino_supe	Profess	nao	Com_2_ou_r	Sim	Sim
Casado_a	de_4_a_10_s	de_4_a_10_s	ensino_supe	Profess	nao	Com_uma_p	Sim	Sim
Casado_a	mais_de_10_	mais_de_10_	ensino_supe	Fisioter	nao	Com_uma_p	Nao	Nao
Casado_a	mais_de_10_	mais_de_10_	ensino_supe	NULL	nao	Com_uma_p	Nao	Sim
Casado_a	mais_de_10_	mais_de_10_	ensino_supe	Bancar	sim	Com_2_ou_r	Nao	Nao
Casado_a	mais_de_10_	mais_de_10_	ensino_supe	Adminis	sim	Com_2_ou_r	Sim	Sim
Casado_a	de_4_a_10_s	mais_de_10_	ensino_supe	NULL	nao	Com_uma_p	Nao	Sim
Solteiro_a	de_4_a_10_s	de_4_a_10_s	ensino_supe	Servidc	nao	Com_2_ou_r	Sim	Sim
Vive_com_cc	de_4_a_10_s	de_4_a_10_s	ensino_supe	Profess	nao	Com_uma_p	Nao	Sim
Solteiro_a	mais_de_4_s	mais_de_4_s	ensino_supe	Analista	nao	Sozinho	Nao	Nao
Solteiro_a	de_4_a_10_s	de_4_a_10_s	ensino_supe	Publicit	nao	Sozinho	Sim	Nao
Solteiro_a	de_4_a_10_s	de_4_a_10_s	ensino_supe	Gerente	nao	Sozinho	Sim	Sim
Casado_a	mais_de_4_s	de_1_a_4_sa	ensino_supe	Profissi	nao	Com_2_ou_r	Sim	Sim
Casado_a	mais_de_10_	mais_de_10_	ensino_supe	Advoga	sim	Com_2_ou_r	Sim	Sim
Casado_a	de_1_a_4_sa	de_1_a_4_sa	ensino_supe	Analista	sim	Com_2_ou_r	Nao	Nao
Solteiro_a	de_4_a_10_s	de_4_a_10_s	ensino_supe	Engenh	nao	Com_uma_p	Sim	Sim
Solteiro_a	de_4_a_10_s	de_4_a_10_s	ensino_supe	Fisioter	nao	Com_2_ou_r	Sim	Sim
Solteiro_a	de_1_a_4_sa	de_4_a_10_s	ensino_supe	Desenv	nao	Sozinho	Nao	Nao

Casado_a	mais_de_10_	mais_de_10_	ensino_supe	NULL	nao	Com_uma_p	Sim	Sim
Solteiro_a	mais_de_10_	mais_de_10_	ensino_supe	NULL	nao	Sozinho	Sim	Sim
Vive_com_cc	de_4_a_10_s	de_4_a_10_s	ensino_supe	Engenh	nao	Com_2_ou_r	Sim	Nao
Solteiro_a	de_4_a_10_s	de_4_a_10_s	ensino_supe	Engenh	nao	Com_2_ou_r	Sim	Sim
Vive_com_cc	mais_de_10_	mais_de_10_	ensino_supe	Publicit	nao	Com_uma_p	Sim	Sim
Vive_com_cc	mais_de_10_	mais_de_10_	ensino_supe	Gerente	nao	Com_2_ou_r	Sim	Nao
Vive_com_cc	mais_de_10_	mais_de_10_	ensino_supe	Engenh	nao	Com_2_ou_r	Nao	Nao
Solteiro_a	de_1_a_4_sa	de_1_a_4_sa	ensino_supe	Secreta	nao	Sozinho	Sim	Sim
Casado_a	mais_de_4_s	mais_de_4_s	ensino_supe	Profess	nao	Com_uma_p	Sim	Sim
Casado_a	de_1_a_4_sa	de_1_a_4_sa	ensino_supe	Profess	sim	Com_2_ou_r	Sim	Nao
Casado_a	mais_de_4_s	mais_de_4_s	ensino_supe	Assiste	nao	Com_2_ou_r	Sim	Nao
Casado_a	mais_de_10_	mais_de_10_	ensino_supe	Profissi	nao	Com_uma_p	Sim	Sim
Vive_com_cc	mais_de_4_s	de_1_a_4_sa	ensino_supe	Empres	nao	Com_uma_p	Sim	Sim
Casado_a	mais_de_10_	mais_de_10_	ensino_supe	Profess	nao	Com_uma_p	Sim	Sim
Casado_a	de_4_a_10_s	de_4_a_10_s	ensino_supe	Enferm	nao	Com_uma_p	Nao	Sim
Casado_a	de_4_a_10_s	de_4_a_10_s	ensino_supe	Analista	sim	Com_2_ou_r	Sim	Nao
Casado_a	de_4_a_10_s	de_4_a_10_s	ensino_supe	Adminis	nao	Com_uma_p	Sim	Nao
Casado_a	mais_de_10_	mais_de_10_	ensino_supe	Engenh	sim	Com_2_ou_r	Nao	Sim
Casado_a	de_1_a_4_sa	mais_de_10_	ensino_supe	Marketi	sim	Com_2_ou_r	Nao	Nao
Vive_com_cc	de_4_a_10_s	mais_de_10_	ensino_supe	NULL	nao	Com_uma_p	Nao	Nao
Solteiro_a	de_4_a_10_s	de_4_a_10_s	ensino_supe	Estatist	nao	Sozinho	Nao	Nao
Casado_a	mais_de_10_	mais_de_10_	ensino_supe	Bancar	nao	Com_uma_p	Sim	Nao
Solteiro_a	de_1_a_4_sa	de_1_a_4_sa	ensino_supe	Servido	sim	Com_uma_p	Sim	Nao
Vive_com_cc	mais_de_10_	mais_de_10_	ensino_supe	Profess	nao	Com_uma_p	Sim	Sim
Casado_a	de_4_a_10_s	de_4_a_10_s	ensino_supe	Engenh	sim	Com_2_ou_r	Sim	Nao
Casado_a	mais_de_10_	mais_de_10_	ensino_supe	Psicolo	nao	Com_uma_p	Sim	Sim
Casado_a	de_4_a_10_s	de_4_a_10_s	ensino_supe	Servido	nao	Com_uma_p	Nao	Nao
Solteiro_a	de_4_a_10_s	mais_de_10_	ensino_medi	Program	nao	Com_uma_p	Nao	Nao
Vive_com_cc	de_1_a_4_sa	de_1_a_4_sa	ensino_supe	Psicolo	sim	Com_2_ou_r	Sim	Nao
Solteiro_a	de_1_a_4_sa	de_1_a_4_sa	ensino_supe	Profess	nao	Com_uma_p	Sim	Sim
Casado_a	mais_de_10_	mais_de_10_	ensino_supe	Servido	nao	Com_uma_p	Sim	Sim
Solteiro_a	de_4_a_10_s	de_4_a_10_s	ensino_supe	Program	nao	Sozinho	Nao	Sim
Casado_a	mais_de_10_	de_4_a_10_s	ensino_supe	Empres	sim	Com_2_ou_r	Sim	Nao
Solteiro_a	de_4_a_10_s	de_4_a_10_s	ensino_supe	Profess	nao	Com_uma_p	Sim	Nao
Solteiro_a	mais_de_4_s	mais_de_4_s	ensino_supe	Psicolo	nao	Com_2_ou_r	Nao	Sim
Solteiro_a	de_1_a_4_sa	de_1_a_4_sa	ensino_supe	Profess	nao	Com_2_ou_r	Sim	Nao
Casado_a	mais_de_10_	mais_de_10_	ensino_supe	Servido	sim	Com_2_ou_r	Sim	Sim
Casado_a	de_4_a_10_s	de_4_a_10_s	ensino_supe	Cientist	nao	Com_uma_p	Sim	Sim
Divorciado_a	de_4_a_10_s	de_1_a_4_sa	ensino_supe	Fisioter	nao	Com_2_ou_r	Sim	Sim
Casado_a	de_4_a_10_s	de_4_a_10_s	ensino_supe	Compra	sim	Com_2_ou_r	Nao	Nao
Casado_a	de_4_a_10_s	de_4_a_10_s	ensino_medi	Profess	nao	Com_uma_p	Nao	Nao
Solteiro_a	de_1_a_4_sa	de_1_a_4_sa	ensino_medi	Analista	nao	Com_2_ou_r	Nao	Nao
Casado_a	de_4_a_10_s	mais_de_10_	ensino_supe	Profess	sim	Com_2_ou_r	Nao	Nao
Casado_a	de_4_a_10_s	de_4_a_10_s	ensino_supe	Empree	sim	Com_2_ou_r	Sim	Sim
Solteiro_a	de_4_a_10_s	de_4_a_10_s	ensino_supe	Oceano	nao	Sozinho	Sim	Sim
Solteiro_a	de_4_a_10_s	de_4_a_10_s	ensino_supe	Engenh	nao	Com_2_ou_r	Sim	Nao
Vive_com_cc	de_4_a_10_s	mais_de_10_	ensino_supe	Desenv	sim	Com_2_ou_r	Nao	Sim
Casado_a	de_1_a_4_sa	de_1_a_4_sa	ensino_supe	Profess	nao	Com_uma_p	Sim	Nao
Solteiro_a	de_1_a_4_sa	de_1_a_4_sa	ensino_supe	Fisioter	nao	Sozinho	Sim	Sim
Vive_com_cc	de_1_a_4_sa	de_1_a_4_sa	ensino_supe	Profissi	sim	Com_2_ou_r	Nao	Nao
Casado_a	de_4_a_10_s	de_4_a_10_s	ensino_supe	Servido	sim	Com_2_ou_r	Sim	Nao
Solteiro_a	de_4_a_10_s	de_4_a_10_s	ensino_supe	Advoga	nao	Com_2_ou_r	Sim	Nao
Solteiro_a	de_1_a_4_sa	de_1_a_4_sa	ensino_supe	Fisioter	nao	Com_2_ou_r	Sim	Sim
Casado_a	mais_de_10_	mais_de_10_	ensino_supe	Profess	sim	Com_2_ou_r	Nao	Nao
Vive_com_cc	de_1_a_4_sa	de_1_a_4_sa	ensino_supe	Profess	nao	Com_uma_p	Sim	Sim
Casado_a	mais_de_10_	mais_de_10_	ensino_supe	Comun	sim	Com_2_ou_r	Nao	Nao
Casado_a	mais_de_10_	mais_de_10_	ensino_supe	Program	sim	Com_2_ou_r	Sim	Nao
Vive_com_cc	mais_de_10_	mais_de_10_	ensino_supe	NULL	nao	Com_uma_p	Sim	Sim

Casado_a	mais_de_10_	mais_de_10_	ensino_supe	Enferm	sim	Com_2_ou_r	Sim	Nao
Casado_a	mais_de_10_	mais_de_10_	ensino_supe	Profess	nao	Com_uma_p	Sim	Nao
Casado_a	de_4_a_10_s	de_4_a_10_s	ensino_supe	Pedago	nao	Com_uma_p	Sim	Sim
Casado_a	de_4_a_10_s	de_4_a_10_s	ensino_supe	Profess	sim	Com_2_ou_r	Nao	Nao
Casado_a	de_4_a_10_s	de_4_a_10_s	ensino_supe	Biologo	sim	Com_2_ou_r	Sim	Sim
Casado_a	de_4_a_10_s	de_4_a_10_s	ensino_supe	Adminis	sim	Com_2_ou_r	Nao	Sim
Casado_a	mais_de_10_	mais_de_10_	ensino_supe	Pesquis	nao	Com_uma_p	Sim	Nao
Solteiro_a	de_1_a_4_sa	de_1_a_4_sa	ensino_medi	Musico	nao	Sozinho	Nao	Sim
Solteiro_a	ate_um_sala	ate_um_sala	ensino_supe	Profess	nao	Com_uma_p	Nao	Nao
Casado_a	de_4_a_10_s	de_4_a_10_s	ensino_supe	Arqueo	sim	Com_2_ou_r	Nao	Nao
Solteiro_a	mais_de_10_	mais_de_10_	ensino_supe	Advoga	nao	Com_uma_p	Nao	Nao
Casado_a	de_4_a_10_s	de_4_a_10_s	ensino_supe	NULL	nao	Com_uma_p	Sim	Sim
Solteiro_a	de_4_a_10_s	de_4_a_10_s	ensino_supe	Servido	sim	Com_uma_p	Sim	Sim
Solteiro_a	de_4_a_10_s	de_4_a_10_s	ensino_supe	Profess	nao	Sozinho	Nao	Sim
Casado_a	de_4_a_10_s	de_4_a_10_s	ensino_supe	Assiste	nao	Com_uma_p	Sim	Sim
Solteiro_a	de_4_a_10_s	de_4_a_10_s	ensino_supe	Profess	sim	Com_uma_p	Nao	Nao
Casado_a	de_1_a_4_sa	de_1_a_4_sa	ensino_supe	Profess	sim	Com_2_ou_r	Sim	Sim
Solteiro_a	de_4_a_10_s	de_4_a_10_s	ensino_supe	Pedago	sim	Com_2_ou_r	Sim	Sim
Solteiro_a	mais_de_4_s	mais_de_4_s	ensino_supe	Servido	nao	Com_2_ou_r	Sim	Sim
Vive_com_co	mais_de_10_	de_4_a_10_s	ensino_supe	Profess	nao	Com_2_ou_r	Sim	Nao
Vive_com_co	de_4_a_10_s	de_4_a_10_s	ensino_supe	Advoga	sim	Com_2_ou_r	Sim	Nao
Casado_a	mais_de_10_	mais_de_10_	ensino_supe	Engenh	nao	Com_uma_p	Nao	Nao
Solteiro_a	de_1_a_4_sa	de_1_a_4_sa	ensino_supe	Profess	nao	Sozinho	Sim	Nao
Casado_a	de_4_a_10_s	de_4_a_10_s	ensino_supe	Servido	nao	Com_uma_p	Sim	Nao
Vive_com_co	mais_de_4_s	de_4_a_10_s	ensino_supe	Servido	nao	Com_2_ou_r	Nao	Nao
Solteiro_a	de_1_a_4_sa	de_1_a_4_sa	ensino_supe	Analista	nao	Sozinho	Nao	Sim
Casado_a	mais_de_10_	mais_de_10_	ensino_supe	Advoga	sim	Com_2_ou_r	Sim	Sim
Solteiro_a	de_4_a_10_s	de_4_a_10_s	ensino_supe	Enferm	nao	Sozinho	Nao	Nao
Vive_com_co	de_4_a_10_s	de_4_a_10_s	ensino_supe	Coorde	nao	Com_uma_p	Sim	Nao
Vive_com_co	de_4_a_10_s	mais_de_10_	ensino_supe	Desenv	nao	Com_uma_p	Nao	Nao
Casado_a	mais_de_10_	mais_de_10_	ensino_supe	Empres	sim	Com_2_ou_r	Sim	Sim
Casado_a	mais_de_4_s	de_1_a_4_sa	ensino_supe	Profess	sim	Com_2_ou_r	Sim	Nao
Divorciado_a	de_4_a_10_s	de_4_a_10_s	ensino_supe	Profess	sim	Com_uma_p	Sim	Nao
Vive_com_co	de_4_a_10_s	mais_de_10_	ensino_supe	Editor_	nao	Com_uma_p	Nao	Nao
Casado_a	mais_de_10_	mais_de_10_	ensino_supe	Fisioter	nao	Com_uma_p	Sim	Sim
Casado_a	mais_de_10_	mais_de_10_	ensino_supe	Analista	sim	Com_2_ou_r	Sim	Sim
Solteiro_a	de_1_a_4_sa	de_1_a_4_sa	ensino_supe	produto	nao	Sozinho	Nao	Sim
Vive_com_co	de_4_a_10_s	de_4_a_10_s	ensino_supe	NULL	sim	Com_2_ou_r	Nao	Nao
Solteiro_a	de_4_a_10_s	de_4_a_10_s	ensino_supe	Profess	nao	Com_uma_p	Sim	Sim
Casado_a	mais_de_10_	mais_de_10_	ensino_supe	Engenh	sim	Com_2_ou_r	Sim	Sim
Solteiro_a	mais_de_10_	mais_de_10_	ensino_supe	Bancar	nao	Com_2_ou_r	Sim	Sim
Casado_a	mais_de_10_	mais_de_10_	ensino_supe	Psicolo	sim	Com_2_ou_r	Sim	Sim
Vive_com_co	mais_de_10_	mais_de_10_	ensino_supe	Profess	nao	Com_uma_p	Sim	Nao
Casado_a	mais_de_10_	mais_de_10_	ensino_supe	Gestao	sim	Com_2_ou_r	Sim	Sim
Solteiro_a	de_1_a_4_sa	de_1_a_4_sa	ensino_supe	Desenv	nao	Com_2_ou_r	Sim	Nao
Divorciado_a	mais_de_10_	de_4_a_10_s	ensino_supe	Vended	nao	Sozinho	Nao	Sim
Vive_com_co	mais_de_4_s	de_1_a_4_sa	ensino_supe	Profess	sim	Com_2_ou_r	Sim	Nao
Vive_com_co	de_4_a_10_s	de_4_a_10_s	ensino_supe	Assiste	nao	Com_2_ou_r	Nao	Nao
Solteiro_a	de_4_a_10_s	de_4_a_10_s	ensino_supe	Profess	nao	Com_uma_p	Nao	Sim
Solteiro_a	mais_de_10_	mais_de_10_	ensino_supe	Profess	nao	Com_uma_p	Nao	Nao
Casado_a	mais_de_10_	de_4_a_10_s	ensino_supe	Empres	sim	Com_2_ou_r	Sim	Nao
Casado_a	mais_de_10_	mais_de_10_	ensino_supe	Profess	sim	Com_2_ou_r	Nao	Nao
Casado_a	mais_de_10_	mais_de_10_	ensino_supe	Fisioter	sim	Com_2_ou_r	Nao	Sim
Solteiro_a	mais_de_10_	mais_de_10_	ensino_supe	Profess	nao	Com_2_ou_r	Sim	Sim
Casado_a	mais_de_10_	mais_de_10_	ensino_supe	Analista	nao	Com_uma_p	Sim	Sim
Vive_com_co	mais_de_10_	mais_de_10_	ensino_supe	NULL	nao	Com_uma_p	Nao	Nao
Casado_a	de_4_a_10_s	de_4_a_10_s	ensino_supe	Profess	sim	Com_2_ou_r	Nao	Sim
Casado_a	mais_de_10_	mais_de_10_	ensino_supe	Bancar	sim	Com_2_ou_r	Sim	Sim

Casado_a	de_1_a_4_sa	de_1_a_4_sa	ensino_supe	Profess	sim	Com_2_ou_r	Sim	Nao
Solteiro_a	mais_de_10_	mais_de_10_	ensino_supe	Profess	nao	Sozinho	Sim	Sim
Solteiro_a	de_4_a_10_s	de_4_a_10_s	ensino_supe	Servido	nao	Sozinho	Nao	Nao
Casado_a	de_4_a_10_s	de_4_a_10_s	ensino_supe	Gestora	sim	Com_2_ou_r	Sim	Sim
Casado_a	mais_de_10_	mais_de_10_	ensino_supe	Gerente	sim	Com_2_ou_r	Nao	Sim
Vive_com_co	de_4_a_10_s	de_4_a_10_s	ensino_supe	Servido	nao	Com_uma_p	Sim	Nao
Casado_a	mais_de_10_	mais_de_10_	ensino_supe	Servido	sim	Com_2_ou_r	Nao	Sim
Vive_com_co	mais_de_10_	mais_de_10_	ensino_supe	Profess	nao	Com_uma_p	Sim	Nao
Solteiro_a	de_4_a_10_s	de_4_a_10_s	ensino_supe	Profess	nao	Sozinho	Sim	Sim
Casado_a	mais_de_10_	mais_de_10_	ensino_supe	Fisioter	sim	Com_2_ou_r	Sim	Sim
Casado_a	de_4_a_10_s	de_4_a_10_s	ensino_supe	Escreve	sim	Com_2_ou_r	Sim	Sim
Casado_a	de_4_a_10_s	mais_de_10_	ensino_supe	Profess	nao	Com_2_ou_r	Nao	Sim
Solteiro_a	mais_de_4_s	mais_de_4_s	ensino_supe	Pos_gr	nao	Com_uma_p	Sim	Sim
Casado_a	de_4_a_10_s	de_4_a_10_s	ensino_supe	CONTA	sim	Com_2_ou_r	Sim	Sim
Casado_a	de_4_a_10_s	mais_de_10_	ensino_supe	Biologo	nao	Com_2_ou_r	Nao	Nao
Solteiro_a	de_4_a_10_s	de_4_a_10_s	ensino_supe	Profess	sim	Com_uma_p	Sim	Sim
Casado_a	de_4_a_10_s	mais_de_10_	ensino_supe	Psicolo	nao	Com_uma_p	Sim	Sim
Casado_a	mais_de_10_	mais_de_10_	ensino_supe	Profess	sim	Com_2_ou_r	Nao	Sim
Solteiro_a	de_4_a_10_s	de_4_a_10_s	ensino_supe	geologa	nao	Com_uma_p	Sim	Nao
Casado_a	mais_de_4_s	mais_de_4_s	ensino_supe	Web_D	nao	Com_uma_p	Sim	Sim
Casado_a	mais_de_10_	mais_de_10_	ensino_supe	Profess	sim	Com_2_ou_r	Sim	Nao
Casado_a	mais_de_10_	mais_de_10_	ensino_supe	Servido	nao	Com_2_ou_r	Nao	Nao
Casado_a	mais_de_10_	mais_de_10_	ensino_supe	Fisioter	sim	Com_2_ou_r	Sim	Sim
Solteiro_a	mais_de_10_	mais_de_10_	ensino_supe	Profess	nao	Sozinho	Sim	Nao
Divorciado_a	mais_de_10_	mais_de_10_	ensino_supe	Profess	sim	Com_2_ou_r	Sim	Sim
Divorciado_a	mais_de_4_s	mais_de_4_s	ensino_supe	Profess	sim	Com_2_ou_r	Nao	Nao
Casado_a	de_4_a_10_s	de_4_a_10_s	ensino_supe	Desenv	sim	Com_2_ou_r	Nao	Nao
Divorciado_a	de_1_a_4_sa	de_1_a_4_sa	ensino_supe	Servido	sim	Com_uma_p	Sim	Sim
Casado_a	mais_de_4_s	de_4_a_10_s	ensino_supe	Aposer	sim	Com_2_ou_r	Sim	Sim
Casado_a	mais_de_10_	mais_de_10_	ensino_supe	Fisioter	sim	Com_2_ou_r	Nao	Nao
Casado_a	mais_de_10_	mais_de_10_	ensino_supe	Profess	sim	Com_2_ou_r	Sim	Sim
Casado_a	mais_de_4_s	de_4_a_10_s	ensino_supe	Bancar	sim	Com_uma_p	Nao	Sim
Casado_a	de_4_a_10_s	mais_de_10_	ensino_supe	Engenh	sim	Com_2_ou_r	Nao	Nao
Casado_a	mais_de_10_	mais_de_10_	ensino_supe	Profess	sim	Com_uma_p	Sim	Sim
Casado_a	de_4_a_10_s	de_4_a_10_s	ensino_supe	Profess	sim	Com_2_ou_r	Nao	Sim
Casado_a	mais_de_10_	mais_de_10_	ensino_supe	Profess	sim	Com_2_ou_r	Nao	Nao
Casado_a	mais_de_10_	mais_de_10_	ensino_supe	Profess	sim	Com_2_ou_r	Sim	Sim
Casado_a	mais_de_10_	mais_de_10_	ensino_supe	Servido	sim	Com_2_ou_r	Nao	Nao
Divorciado_a	mais_de_10_	mais_de_10_	ensino_supe	Engenh	sim	Com_2_ou_r	Nao	Nao
Solteiro_a	mais_de_10_	mais_de_10_	ensino_supe	Pesquis	sim	Com_2_ou_r	Sim	Nao
Casado_a	de_4_a_10_s	de_4_a_10_s	ensino_supe	Profess	sim	Com_uma_p	Sim	Sim

quanto_tem	tem_o_habi	ingere_bebi	tem_algum_	Faz_uso_de	se_sim_qua	passou_a_u	qual
Mais_que_12	Nao	Socialmente	Sim	Sim	Insulina-_vac	Nao	NULL
Mais_que_12	Nao	Socialmente	Nao	Nao	NULL	Nao	NULL
De_8_a_10_	Nao	Socialmente	Nao	Nao	NULL	Nao	NULL
De_8_a_10_	Nao	Socialmente	Nao	Nao	NULL	Nao	NULL
De_10_a_12	Nao	Regularment	Nao	Nao	NULL	Nao	NULL
De_4_a_6_h	Nao	Socialmente	Sim	Nao	NULL	Nao	NULL
Mais_que_12	Nao	Socialmente	Sim	Nao	NULL	Nao	NULL
De_8_a_10_	Nao	Regularment	Nao	Sim	Tamisa_20_a	Sim	Vitamina_D
De_6_a_8_h	Nao	Socialmente	Sim	Sim	Espironolacto	Sim	Espironolacto
De_8_a_10_	Nao	Socialmente	Sim	Sim	Quetros_Que	Sim	Quetros_Que
De_10_a_12	Nao	Socialmente	Nao	Nao	NULL	Nao	NULL
De_8_a_10_	Nao	Socialmente	Nao	Nao	NULL	Nao	NULL
Mais_que_12	Sim	Nunca	Sim	Sim	Cefaliv_para	Nao	NULL
De_8_a_10_	Nao	Nunca	Nao	Nao	NULL	Nao	NULL
Mais_que_12	Nao	Socialmente	Sim	Nao	NULL	Nao	NULL
De_8_a_10_	Nao	Socialmente	Sim	Nao	NULL	Nao	NULL
De_6_a_8_h	Nao	Socialmente	Sim	Nao	NULL	Nao	NULL
De_6_a_8_h	Nao	Nunca	Nao	Sim	Anticoncepci	Sim	Vitamina_D
De_8_a_10_	Nao	Socialmente	Sim	Nao	NULL	Nao	NULL
De_4_a_6_h	Nao	Socialmente	Nao	Nao	NULL	Nao	NULL
Mais_que_12	Nao	Socialmente	Nao	Nao	NULL	Nao	NULL
De_10_a_12	Nao	Nunca	Nao	Nao	NULL	Nao	NULL
Menos_que_	Nao	Socialmente	Nao	Nao	NULL	Nao	NULL
De_10_a_12	Nao	Socialmente	Sim	Nao	NULL	Sim	Tomei_vitam
Mais_que_12	Nao	Nunca	Sim	Sim	Vitaminas-_a	Nao	NULL
Menos_que_	Nao	Socialmente	Nao	Nao	NULL	Nao	NULL
De_8_a_10_	Sim	Regularment	Nao	Nao	NULL	Nao	NULL
De_6_a_8_h	Nao	Socialmente	Nao	Sim	Anticoncepci	Nao	NULL
De_6_a_8_h	Nao	Nunca	Sim	Sim	Histamin	Nao	NULL
De_8_a_10_	Nao	Socialmente	Nao	Nao	NULL	Nao	NULL
De_8_a_10_	Nao	Socialmente	Sim	Sim	Allurene_die	Sim	colecalciferol
De_8_a_10_	Nao	Regularment	Sim	Sim	Venlafaxina-_	Nao	NULL
Mais_que_12	Nao	Socialmente	Sim	Sim	Antidepressiv	Sim	Antidepressiv
Mais_que_12	Nao	Nunca	Nao	Nao	NULL	Nao	NULL
De_8_a_10_	Nao	Regularment	Nao	Nao	NULL	Nao	NULL
De_10_a_12	Nao	Regularment	Sim	Nao	NULL	Nao	NULL
De_10_a_12	Nao	Socialmente	Nao	Sim	Vitaminas_	Nao	NULL
Menos_que_	Nao	Socialmente	Nao	Nao	NULL	Nao	NULL
De_10_a_12	Nao	Socialmente	Sim	Nao	NULL	Nao	NULL
De_8_a_10_	Nao	Socialmente	Nao	Sim	Finalop	Nao	NULL
De_8_a_10_	Nao	Socialmente	Nao	Nao	NULL	Nao	NULL
De_8_a_10_	Nao	Nunca	Nao	Nao	NULL	Nao	NULL
De_10_a_12	Nao	Nunca	Nao	Nao	NULL	Nao	NULL
De_6_a_8_h	Nao	Nunca	Sim	Nao	NULL	Sim	Eventualmen
De_10_a_12	Nao	Socialmente	Nao	Nao	NULL	Nao	NULL
De_10_a_12	Nao	Socialmente	Nao	Nao	NULL	Nao	NULL
De_8_a_10_	Nao	Socialmente	Sim	Sim	Tandrilax	Nao	NULL
De_8_a_10_	Nao	Socialmente	Nao	Sim	Anticoncepci	Nao	NULL
De_8_a_10_	Nao	Socialmente	Nao	Nao	NULL	Sim	Remedio_ma
De_6_a_8_h	Sim	Socialmente	Sim	Nao	NULL	Sim	Melatonina-_
De_10_a_12	Nao	Socialmente	Nao	Nao	_	Nao	NULL
De_4_a_6_h	Nao	Socialmente	Nao	Sim	Anticoncepci	Nao	NULL
De_6_a_8_h	Nao	Nunca	Sim	Sim	NULL	Nao	NULL
De_10_a_12	Nao	Socialmente	Sim	Sim	Pamelor-_An	Nao	NULL
Mais_que_12	Nao	Socialmente	Sim	Sim	Dexilant	Sim	Dexilant

De_6_a_8_h	Nao	Regularment	Nao	Sim	Mesigyna	Sim	oleo_de_peix
De_4_a_6_h	Nao	Socialmente	Nao	Nao	NULL	Nao	NULL
De_8_a_10_	Nao	Socialmente	Nao	Nao	NULL	Nao	NULL
De_8_a_10_	Nao	Socialmente	Sim	Sim	Pristiq-_Zolpi	Sim	Pristiq-_Zolpi
Mais_que_12	Nao	Socialmente	Nao	Nao	NULL	Nao	NULL
De_8_a_10_	Nao	Socialmente	Nao	Nao	NULL	Nao	NULL
De_8_a_10_	Nao	Socialmente	Sim	Sim	Suplementac	Nao	NULL
De_8_a_10_	Nao	Nunca	Sim	Sim	desvenlafaxi	Sim	desvenlafaxi
De_6_a_8_h	Nao	Nunca	Sim	Nao	NULL	Nao	NULL
De_8_a_10_	Nao	Nunca	Nao	Nao	NULL	Nao	NULL
De_4_a_6_h	Nao	Socialmente	Nao	Nao	NULL	Nao	NULL
De_8_a_10_	Nao	Socialmente	Sim	Nao	NULL	Nao	NULL
De_8_a_10_	Nao	Socialmente	Nao	Nao	NULL	Nao	NULL
De_8_a_10_	Nao	Socialmente	Sim	Sim	Vitaminas_va	Nao	NULL
De_4_a_6_h	Nao	Socialmente	Nao	Nao	NULL	Nao	NULL
De_6_a_8_h	Nao	Socialmente	Nao	Nao	NULL	Nao	NULL
De_8_a_10_	Nao	Socialmente	Nao	Nao	NULL	Nao	NULL
De_8_a_10_	Nao	Regularment	Nao	Nao	NULL	Nao	NULL
De_8_a_10_	Nao	Regularment	Sim	Nao	NULL	Nao	NULL
De_10_a_12	Nao	Socialmente	Nao_	Sim	Pondera_XR	Sim	Pondera_XR
De_6_a_8_h	Nao	Socialmente	Nao_	Nao	NULL	Nao	NULL
De_6_a_8_h	Nao	Socialmente	Nao	Sim	Anticoncepci	Nao	NULL
De_6_a_8_h	Nao	Regularment	Nao	Nao	NULL	Nao	NULL
De_8_a_10_	Nao	Socialmente	Nao	Nao	NULL	Nao	NULL
De_6_a_8_h	Nao	Socialmente	Nao	Nao	NULL	Nao	NULL
Mais_que_12	Nao	Socialmente	Nao	Nao	NULL	Nao	NULL
De_8_a_10_	Nao	Regularment	Sim	Nao	Nao	Nao	NULL
De_8_a_10_	Nao	Socialmente	Nao	Nao	NULL	Sim	Vitaminas_
De_6_a_8_h	Nao	Nunca	Sim	Nao	NULL	Nao	NULL
De_4_a_6_h	Nao	Nunca	Nao	Nao	NULL	Sim	Melatonina
De_10_a_12	Nao	Regularment	Sim	Sim	Brintellix_e_F	Nao	NULL
De_8_a_10_	Nao	Nunca	Nao	Nao	NULL	Nao	NULL
De_6_a_8_h	Nao	Regularment	Nao	Nao	NULL	Nao	NULL
Mais_que_12	Nao	Socialmente	Nao	Nao	NULL	Nao	NULL
De_8_a_10_	Nao	Socialmente	Sim	Sim	Pimozida	Nao	NULL
De_10_a_12	Nao	Nunca	Nao	Nao	NULL	Sim	Vitamina_D
Mais_que_12	Nao	Socialmente	Nao	Nao	NULL	Nao	NULL
De_8_a_10_	Nao	Regularment	Sim	Nao	NULL	Sim	Vitamina_D_
De_6_a_8_h	Nao	Socialmente	Sim	Sim	Patz_para_ir	Sim	Flanax_e_Mi
De_8_a_10_	Nao	Regularment	Sim	Sim	Escitalopram	Nao	NULL
De_8_a_10_	Nao	Socialmente	Sim	Sim	Antidepressi	Nao	NULL
De_4_a_6_h	Nao	Socialmente	Nao	Nao	NULL	Nao	NULL
Mais_que_12	Nao	Nunca	Sim	Nao	NULL	Nao	NULL
De_6_a_8_h	Nao	Socialmente	Sim	Nao	NULL	Sim	vitaminas
Mais_que_12	Sim	Socialmente	Nao	Nao	NULL	Nao	NULL
De_8_a_10_	Nao	Socialmente	Sim	Nao	NULL	Sim	Fluoxetina
De_8_a_10_	Nao	Nunca	Nao	Nao	NULL	Nao	NULL
Mais_que_12	Nao	Nunca	Nao	Nao	NULL	Nao	NULL
De_10_a_12	Nao	Socialmente	Nao	Nao	NULL	Nao	NULL
Mais_que_12	Nao	Socialmente	Sim	Sim	Zoloft	Nao	NULL
De_6_a_8_h	Nao	Socialmente	Nao	Nao	NULL	Nao	NULL
De_8_a_10_	Nao	Socialmente	Nao	Sim	Venlift-od	Nao	NULL
De_6_a_8_h	Nao	Socialmente	Sim	Nao	NULL	Nao	NULL
De_8_a_10_	Nao	Socialmente	Nao	Nao	NULL	Nao	NULL
De_10_a_12	Sim	Socialmente	Sim	Nao	NULL	Nao	NULL
De_10_a_12	Nao	Socialmente	Sim	Sim	Escitalopram	Nao	NULL
De_8_a_10_	Nao	Socialmente	Nao	Nao	NULL	Sim	Novalgina_pa
De_6_a_8_h	Nao	Socialmente	Nao	Nao	NULL	Nao	NULL

De_8_a_10_	Nao	Nunca	Nao	Nao	NULL	Nao	NULL
De_4_a_6_h	Nao	Socialmente	Sim	Sim	Zodel-_xarrlt	Sim	zetron_para_
De_8_a_10_	Nao	Socialmente	Nao	Nao	No_maximo_	Sim	No_maximo_
De_8_a_10_	Nao	Nunca	Sim	Nao	NULL	Nao	NULL
De_6_a_8_h	Nao	Socialmente	Nao	Nao	NULL	Nao	NULL
De_10_a_12	Nao	Nunca	Nao	Nao	NULL	Sim	vitamina_C-_
De_4_a_6_h	Nao	Socialmente	Nao	Nao	NULL	Nao	NULL
De_10_a_12	Nao	Socialmente	Nao	Nao	NULL	Nao	NULL
Menos_que_	Nao	Socialmente	Nao	Nao	NULL	Nao	NULL
Mais_que_12	Nao	Regularment	Nao	Nao	NULL	Nao	NULL
De_4_a_6_h	Nao	Socialmente	Sim	Sim	Escitalopram	Sim	Cremes_para
De_8_a_10_	Nao	Socialmente	Nao	Nao	NULL	Nao	NULL
Mais_que_12	Nao	Nunca	Sim	Sim	Roacutan	Sim	Roacutan
De_8_a_10_	Nao	Nunca	Sim	Nao	NULL	Nao	NULL
De_8_a_10_	Nao	Socialmente	Nao	Nao	NULL	Nao	NULL
Mais_que_12	Nao	Regularment	Nao	Nao	NULL	Nao	NULL
De_6_a_8_h	Nao	Nunca	Sim	Nao	NULL	Nao	NULL
De_8_a_10_	Nao	Socialmente	Sim	Nao	NULL	Nao	NULL
Mais_que_12	Nao	Regularment	Sim	Sim	Cloridrato_de	Nao	NULL
De_8_a_10_	Nao	Regularment	Nao	Sim	Anticoncepci	Nao	NULL
De_8_a_10_	Nao	Regularment	Nao	Nao	NULL	Nao	NULL
De_6_a_8_h	Nao	Socialmente	Nao	Sim	Sintroyde_	Nao	NULL
De_4_a_6_h	Nao	Socialmente	Nao	Nao	NULL	Sim	Mais_recente
De_8_a_10_	Nao	Nunca	Nao	Nao	NULL	Nao	NULL
De_10_a_12	Nao	Socialmente	Sim	Nao	NULL	Nao	NULL
De_6_a_8_h	Nao	Socialmente	Sim	Nao	NULL	Nao	NULL
De_8_a_10_	Nao	Nunca	Sim	Nao	NULL	Sim	Naproxeno-_
De_6_a_8_h	Nao	Socialmente	Sim	Sim	NULL	Nao	NULL
Mais_que_12	Nao	Socialmente	Sim	Sim	Puran_T4_-_	Nao	NULL
De_8_a_10_	Nao	Regularment	Nao	Nao	NULL	Nao	NULL
Mais_que_12	Nao	Socialmente	Sim	Sim	Amitriptilina_	Sim	Aumento_de_
Mais_que_12	Nao	Socialmente	Sim	Nao	NULL	Nao	NULL
De_8_a_10_	Nao	Nunca	Nao	Nao	NULL	Sim	Ansioliticos_f
De_8_a_10_	Nao	Socialmente	Nao	Sim	Preventivo_d	Nao	NULL
De_8_a_10_	Nao	Socialmente	Nao	Nao	NULL	Nao	NULL
De_4_a_6_h	Nao	Socialmente	Nao	Nao	NULL	Nao	NULL
De_8_a_10_	Sim	Socialmente	Nao	Sim	Fluoxetina	Nao	NULL
De_10_a_12	Nao	Socialmente	Nao	Nao	NULL	Nao	NULL
De_4_a_6_h	Nao	Socialmente	Nao	Sim	Puran__t4_-	Nao	NULL
De_10_a_12	Nao	Socialmente	Sim	Sim	Topiramato	Nao	NULL
De_8_a_10_	Nao	Nunca	Nao	Nao	NULL	Nao	NULL
De_8_a_10_	Nao	Socialmente	Nao	Nao	NULL	Nao	NULL
De_10_a_12	Nao	Socialmente	Nao	Nao	NULL	Nao	NULL
De_8_a_10_	Nao	Socialmente	Nao	Nao	NULL	Nao	NULL
Menos_que_	Nao	Socialmente	Sim	Nao	NULL	Nao	NULL
De_8_a_10_	Nao	Socialmente	Sim	Sim	Alenia	Sim	Remedio_pa
De_8_a_10_	Nao	Nunca	Sim	Nao	NULL	Nao	NULL
De_8_a_10_	Nao	Socialmente	Nao	Nao	NULL	Nao	NULL
De_8_a_10_	Nao	Socialmente	Nao	Nao	NULL	Nao	NULL
Mais_que_12	Nao	Socialmente	Nao	Nao	NULL	Nao	NULL
De_6_a_8_h	Nao	Regularment	Nao	Sim	Anticoncepci	Nao	NULL
De_6_a_8_h	Nao	Socialmente	Sim	Sim	Naprix	Nao	NULL
De_8_a_10_	Nao	Nunca	Nao	Nao	nao	Nao	NULL
De_10_a_12	Nao	Socialmente	Sim	Nao	NULL	Nao	NULL
De_10_a_12	Nao	Nunca	Nao	Nao	Nenhum	Nao	NULL
Mais_que_12	Nao	Socialmente	Nao	Sim	Tolrest_e_ze	Sim	Quetiapina
Menos_que_	Nao	Socialmente	Nao	Sim	Anticoncepci	Nao	NULL
De_8_a_10_	Nao	Socialmente	Nao	Nao	NULL	Nao	NULL

De_8_a_10_	Nao	Nunca	Nao	Nao	NULL	Nao	NULL
De_8_a_10_	Nao	Socialmente	Nao	Nao	NULL	Nao	NULL
De_10_a_12	Nao	Nunca	Sim	Sim	Tolrest_	Nao	NULL
De_4_a_6_h	Nao	Socialmente	Nao	Nao	NULL	Nao	NULL
De_6_a_8_h	Nao	Socialmente	Nao	Nao	NULL	Nao	NULL
Mais_que_12	Nao	Nunca	Sim	Sim	Aerolin-_min	Sim	escitalopram
De_6_a_8_h	Sim	Socialmente	Nao	Nao	NULL	Nao	NULL
De_6_a_8_h	Nao	Socialmente	Nao	Nao	NULL	Nao	NULL
De_8_a_10_	Nao	Regularment	Nao	Nao	NULL	Sim	Vitamina_D
De_10_a_12	Nao	Nunca	Nao	Sim	Anticocepcio	Nao	NULL
De_10_a_12	Nao	Nunca	Sim	Sim	Patz-_alcytar	Sim	Dexilant_e_tr
De_8_a_10_	Nao	Socialmente	Sim	Sim	Puran	Nao	NULL
De_4_a_6_h	Nao	Regularment	Nao	Nao	NULL	Nao	NULL
De_6_a_8_h	Nao	Socialmente	Nao	Nao	NULL	Nao	NULL
De_10_a_12	Nao	Socialmente	Sim	Sim	Fitoterapicos	Nao	NULL
De_10_a_12	Nao	Socialmente	Sim	Nao	NULL	Nao	NULL
De_8_a_10_	Nao	Socialmente	Nao	Sim	Anticoncepci	Nao	NULL
De_8_a_10_	Nao	Nunca	Nao	Nao	NULL	Nao	NULL
Mais_que_12	Nao	Socialmente	Sim	Sim	Aspirina_tod	Nao	NULL
Mais_que_12	Sim	Socialmente	Nao	Nao	NULL	Nao	NULL
Mais_que_12	Nao	Socialmente	Nao	Nao	NULL	Nao	NULL
De_8_a_10_	Nao	Regularment	Nao	Nao	NULL	Nao	NULL
De_8_a_10_	Nao	Socialmente	Nao	Nao	NULL	Nao	NULL
De_6_a_8_h	Nao	Socialmente	Nao	Nao	NULL	Nao	NULL
De_8_a_10_	Nao	Socialmente	Nao	Nao	NULL	Nao	NULL
De_6_a_8_h	Nao	Socialmente	Nao	Nao	NULL	Nao	NULL
De_10_a_12	Nao	Socialmente	Sim	Sim	Busonid-recc	Nao	NULL
Mais_que_12	Nao	Nunca	Sim	Sim	Quetros-_Ve	Nao	NULL
De_10_a_12	Nao	Socialmente	Sim	Nao	NULL	Sim	Antialergicos
De_6_a_8_h	Nao	Nunca	Sim	Nao	NULL	Nao	NULL
De_6_a_8_h	Nao	Socialmente	Sim	Sim	rosucor_10_r	Nao	NULL
De_8_a_10_	Nao	Socialmente	Nao	Nao	NULL	Nao	NULL
De_6_a_8_h	Nao	Regularment	Nao	Nao	NULL	Nao	NULL
De_10_a_12	Nao	Socialmente	Sim	Nao	NULL	Nao	NULL
De_8_a_10_	Nao	Nunca	Sim	Sim	Frontal	Sim	Frontal_
De_8_a_10_	Nao	Socialmente	Sim	Sim	Clortalidona	Sim	Clortalidona
De_6_a_8_h	Nao	Nunca	Sim	Sim	Antidepressi	Nao	NULL
De_8_a_10_	Nao	Regularment	Sim	Sim	Rosovastatin	Nao	NULL
De_4_a_6_h	Nao	Socialmente	Nao	Nao	NULL	Nao	NULL
De_8_a_10_	Nao	Nunca	Nao	Nao	Nao	Nao	NULL
De_4_a_6_h	Nao	Socialmente	Nao	Nao	NULL	Nao	NULL
De_8_a_10_	Nao	Socialmente	Sim	Nao	NULL	Nao	NULL
De_6_a_8_h	Nao	Nunca	Nao	Nao	NULL	Nao	NULL
De_6_a_8_h	Nao	Socialmente	Nao	Nao	NULL	Nao	NULL
De_6_a_8_h	Sim	Socialmente	Sim	Sim	Alenia_12-40	Nao	NULL
De_8_a_10_	Nao	Nunca	Nao	Nao	NULL	Nao	NULL
De_6_a_8_h	Nao	Socialmente	Nao	Nao	NULL	Nao	NULL
De_4_a_6_h	Nao	Socialmente	Nao	Nao	NULL	Nao	NULL
Menos_que_	Nao	Nunca	Nao	Nao	NULL	Nao	NULL
De_4_a_6_h	Nao	Socialmente	Nao	Nao	NULL	Nao	NULL
De_8_a_10_	Nao	Nunca	Sim	Sim	Colchicina-_	Nao	NULL
De_8_a_10_	Nao	Socialmente	Sim	Nao	NULL	Nao	NULL
Menos_que_	Nao	Nunca	Nao	Nao	NULL	Nao	NULL
Menos_que_	Nao	Socialmente	Sim	Sim	Sertralina_75	Sim	Aumentei_a_
De_10_a_12	Nao	Regularment	Sim	Nao	NULL	Nao	NULL
De_8_a_10_	Nao	Socialmente	Sim	Sim	Zoloft_100_n	Nao	NULL
De_10_a_12	Nao	Socialmente	Nao	Nao	NULL	Nao	NULL
De_6_a_8_h	Nao	Regularment	Nao	Nao	NULL	Nao	NULL

De_4_a_6_h	Nao	Nunca	Nao	Nao	NULL	Sim	Sertralina
De_8_a_10_	Nao	Nunca	Nao	Sim	Succinato_de	Sim	topiramato_
De_6_a_8_h	Nao	Nunca	Sim	Sim	Eutirox_100r	Nao	NULL
Mais_que_12	Nao	Nunca	Nao	Nao	NULL	Nao	NULL
De_6_a_8_h	Nao	Regularment	Nao	Sim	homeopatico	Sim	homeopatico
De_6_a_8_h	Nao	Socialmente	Sim	Sim	Sulfato_de_H	Nao	NULL
De_6_a_8_h	Nao	Socialmente	Sim	Sim	Puran	Nao	NULL
De_4_a_6_h	Nao	Socialmente	Nao	Nao	NULL	Nao	NULL
De_6_a_8_h	Nao	Socialmente	Sim	Sim	Deferiprona-_	Nao	NULL
De_8_a_10_	Nao	Nunca	Sim	Sim	Ritalina-_Ant	Nao	NULL
De_8_a_10_	Nao	Socialmente	Nao	Nao	NULL	Nao	NULL
De_4_a_6_h	Nao	Nunca	Sim	Sim	NULL	Sim	Rivotril_subli
De_8_a_10_	Nao	Socialmente	Sim	Nao	NULL	Sim	Desvelafaxin
De_6_a_8_h	Nao	Socialmente	Nao	Nao	NULL	Nao	NULL
Menos_que_	Nao	Socialmente	Sim	Sim	Humira	Nao	NULL
De_8_a_10_	Nao	Nunca	Sim	Sim	Puran	Nao	NULL
De_4_a_6_h	Nao	Socialmente	Nao	Nao	NULL	Nao	NULL
De_8_a_10_	Nao	Socialmente	Sim	Sim	Aradois_25m	Sim	Orlistate_e_p
De_8_a_10_	Nao	Nunca	Sim	Nao	nao	Nao	NULL
De_10_a_12	Sim	Regularment	Nao	Nao	nenhum	Nao	NULL
De_6_a_8_h	Nao	Socialmente	Nao	Sim	NULL	Nao	NULL
De_8_a_10_	Nao	Nunca	Nao	Nao	NULL	Nao	NULL
De_4_a_6_h	Nao	Nunca	Nao	Nao	NULL	Nao	NULL
De_4_a_6_h	Nao	Nunca	Sim	Sim	Dexilant-_do	Sim	Dimpless-_al
De_6_a_8_h	Nao	Nunca	Sim	Sim	Pilula_antico	Nao	NULL
De_8_a_10_	Sim	Socialmente	Nao	Nao	NULL	Nao	NULL
De_6_a_8_h	Nao	Nunca	Nao	Nao	NULL	Nao	NULL
De_6_a_8_h	Sim	Socialmente	Sim	Sim	Buoropiona-_	Nao	NULL
De_8_a_10_	Nao	Nunca	Nao	Nao	NULL	Nao	NULL
Mais_que_12	Nao	Regularment	Nao	Nao	NULL	Nao	NULL
De_4_a_6_h	Nao	Nunca	Nao	Nao	NULL	Nao	NULL
Menos_que_	Nao	Nunca	Nao	Nao	NULL	Nao	NULL
Mais_que_12	Nao	Nunca	Nao	Nao	NULL	Sim	remedios_pa
De_8_a_10_	Nao	Socialmente	Nao	Nao	NULL	Nao	NULL
Menos_que_	Nao	Socialmente	Nao	Nao	NULL	Nao	NULL
De_8_a_10_	Nao	Nunca	Sim	Nao	NULL	Nao	NULL
Mais_que_12	Nao	Socialmente	Sim	Nao	NULL	Sim	vitamina_d
De_6_a_8_h	Nao	Socialmente	Sim	Nao	NULL	Nao	NULL
De_8_a_10_	Nao	Socialmente	Nao	Nao	NULL	Nao	NULL
De_8_a_10_	Nao	Regularment	Nao	Nao	NULL	Nao	NULL
De_8_a_10_	Nao	Socialmente	Sim	Sim	Engov_1_ve:	Sim	Engov
De_6_a_8_h	Nao	Socialmente	Nao	Nao	NULL	Nao	NULL
De_10_a_12	Nao	Socialmente	Sim	Sim	Anticoncepci	Sim	Antidepressi
De_8_a_10_	Nao	Socialmente	Nao	Nao	NULL	Nao	NULL
De_8_a_10_	Nao	Socialmente	Sim	Sim	Venlaflaxina-	Nao	NULL
De_8_a_10_	Nao	Socialmente	Sim	Sim	Lozartana	Nao	NULL
De_6_a_8_h	Nao	Socialmente	Nao	Nao	NULL	Sim	antiinflamato
De_8_a_10_	Nao	Socialmente	Nao	Sim	Zoloft_50mg	Nao	NULL
De_8_a_10_	Nao	Regularment	Nao	Nao	NULL	Nao	NULL
De_10_a_12	Nao	Nunca	Nao	Nao	NULL	Nao	NULL
De_8_a_10_	Nao	Socialmente	Nao	Sim	Anticoncepci	Sim	As_vezes_pa
De_8_a_10_	Nao	Socialmente	Sim	Sim	Puran_T4;_C	Sim	Glifage_500r
De_6_a_8_h	Nao	Socialmente	Sim	Nao	NULL	Nao	NULL
De_4_a_6_h	Nao	Regularment	Nao	Nao	NULL	Nao	NULL
De_8_a_10_	Nao	Socialmente	Sim	Sim	Pressplus	Sim	Sinvatastina
De_4_a_6_h	Nao	Socialmente	Sim	Sim	Losartana_po	Nao	NULL
De_6_a_8_h	Nao	Nunca	Nao	Nao	NULL	Sim	Precisei_tom
De_6_a_8_h	Nao	Socialmente	Sim	Sim	Atacand_con	Nao	NULL

Mais_que_12	Nao	Nunca	Sim	Nao	NULL	Nao	NULL
De_6_a_8_h	Nao	Nunca	Nao	Nao	NULL	Nao	NULL
Mais_que_12	Nao	Socialmente	Nao	Nao	NULL	Nao	NULL
De_4_a_6_h	Nao	Socialmente	Nao	Nao	NULL	Nao	NULL
De_8_a_10_	Sim	Regularment	Nao	Nao	NULL	Nao	NULL
De_8_a_10_	Nao	Socialmente	Sim	Sim	Victoza-_bar	Nao	NULL
Menos_que_	Nao	Nunca	Sim	Nao	NULL	Nao	NULL
De_8_a_10_	Nao	Socialmente	Nao	Nao	NULL	Nao	NULL
De_10_a_12	Nao	Socialmente	Nao	Nao	NULL	Nao	NULL
Menos_que_	Nao	Socialmente	Nao	Nao	NULL	Nao	NULL
De_8_a_10_	Nao	Nunca	Nao	Sim	Propranolol_	Nao	NULL
De_10_a_12	Nao	Socialmente	Nao	Nao	NULL	Nao	NULL
De_6_a_8_h	Nao	Nunca	Sim	Sim	Analgesicos_	Nao	NULL
De_6_a_8_h	Nao	Socialmente	Sim	Sim	CORUS-_LIF	Nao	NULL
De_6_a_8_h	Nao	Regularment	Sim	Nao	NULL	Sim	Infralax
De_10_a_12	Nao	Socialmente	Sim	Sim	Donaren_e_a	Sim	Tolrest
De_6_a_8_h	Nao	Socialmente	Sim	Nao	NULL	Nao	NULL
De_8_a_10_	Nao	Socialmente	Nao	Nao	NULL	Nao	NULL
De_10_a_12	Nao	Nunca	Sim	Nao	NULL	Nao	NULL
De_8_a_10_	Nao	Nunca	Sim	Sim	Insulina_-_m	Sim	Unoprost
Menos_que_	Nao	Nunca	Sim	Sim	Pantoprazol-	Sim	Dievari
De_8_a_10_	Nao	Socialmente	Sim	Sim	Losartana	Nao	NULL
De_6_a_8_h	Nao	Socialmente	Sim	Sim	Wellbutrin_e_	Sim	Analgesico_e
Menos_que_	Nao	Socialmente	Nao	Nao	NULL	Sim	Sertralina
De_8_a_10_	Nao	Socialmente	Sim	Sim	Puran_T4-_d	Nao	NULL
Mais_que_12	Nao	Nunca	Nao	Sim	NULL	Sim	Ansiolitico
De_10_a_12	Nao	Nunca	Sim	Nao	NULL	Nao	NULL
De_6_a_8_h	Nao	Socialmente	Nao	Nao	NULL	Nao	NULL
Menos_que_	Nao	Socialmente	Nao	Nao	NULL	Nao	NULL
De_4_a_6_h	Nao	Socialmente	Nao	Nao	NULL	Nao	NULL
De_8_a_10_	Nao	Socialmente	Sim	Sim	Benicar	Nao	NULL
De_6_a_8_h	Nao	Nunca	Sim	Sim	Pressat-_ser	Nao	NULL
De_4_a_6_h	Nao	Regularment	Nao	Nao	NULL	Nao	NULL
De_6_a_8_h	Nao	Socialmente	Sim	Sim	Candesartan	Nao	NULL
De_4_a_6_h	Nao	Nunca	Sim	Sim	Metiformina	Nao	NULL
De_4_a_6_h	Nao	Socialmente	Sim	Sim	Coversyl_4_r	Nao	NULL
De_8_a_10_	Nao	Nunca	Sim	Sim	Rabeprazol_	Sim	Doss
De_8_a_10_	Nao	Nunca	Sim	Sim	Glifag	Nao	NULL
De_8_a_10_	Nao	Nunca	Nao	Nao	NULL	Nao	NULL
De_6_a_8_h	Sim	Socialmente	Nao	Nao	NULL	Nao	NULL
De_6_a_8_h	Nao	Socialmente	Sim	Sim	Zart_50_mg	Nao	NULL

						avaliacao_da_dor			
						caracterizacao_geral_da_dor			
faz_algum_	qual_	assinale_a_	quantas_ho	voce_esta	o_	voce_sentia	se_sim_ha_	m	assinale_a_
Sim	Tratamento_	Voce_esta_t	de_6_a_10_	Saindo_de_c	9	Nao	NULL	N	NULL
Nao	NULL	Voce_esta_t	ate_6_horas_	Saindo_de_c	8	Sim	1_-_5_anos	3	Cervical-_pe
Sim	Faco_suplen	Voce_esta_t	de_6_a_10_	Saindo_de_c	8	Sim	1_-_5_anos	4	Cervical-_pe
Nao	NULL	Voce_esta_t	de_6_a_10_	Saindo_de_c	7	Sim	Menos_de_1	5	Cabeca-_Om
Nao	NULL	Voce_esta_t	de_6_a_10_	Saindo_de_c	7	Nao	NULL	N	NULL
Nao	NULL	Voce_esta_t	ate_6_horas_	Saindo_de_c	10	Nao	NULL	N	NULL
Nao	NULL	Voce_esta_t	mais_de_10_	De_quarente	3	Nao	NULL	N	NULL
Sim	Terapia	Voce_esta_t	de_6_a_10_	Saindo_de_c	7	Nao	NULL	N	NULL
Sim	as_vezes-_u	Voce_esta_t	de_6_a_10_	Saindo_de_c	10	Nao	NULL	N	NULL
Nao	NULL	Voce_esta_t	de_6_a_10_	Saindo_de_c	4	Nao	NULL	N	NULL
Nao	NULL	Voce_esta_t	de_6_a_10_	Saindo_de_c	9	Nao	NULL	N	NULL
Nao	NULL	Voce_esta_t	de_6_a_10_	Saindo_de_c	7	Nao	NULL	N	NULL
Nao	NULL	Voce_esta_t	de_6_a_10_	Saindo_de_c	5	Sim	1_-_5_anos	6	Cabeca-_Col
Nao	NULL	Voce_esta_t	de_6_a_10_	Saindo_de_c	3	Nao	NULL	N	NULL
Nao	NULL	Voce_esta_t	de_6_a_10_	Saindo_de_c	8	Nao	NULL	N	NULL
Nao	NULL	Voce_esta_t	de_6_a_10_	Saindo_de_c	10	Nao	NULL	N	NULL
Nao	NULL	Voce_esta_t	de_6_a_10_	Saindo_de_c	10	Nao	NULL	N	NULL
Nao	NULL	Voce_esta_t	de_6_a_10_	Saindo_de_c	9	Sim	6_-_10_anos	7	Cervical-_pe
Nao	NULL	Voce_esta_t	de_6_a_10_	Saindo_de_c	8	Sim	Menos_de_1	8	Punhos-_Cer
Nao	NULL	Voce_esta_t	de_6_a_10_	Saindo_de_c	7	Nao	NULL	N	NULL
Nao	NULL	Voce_esta_t	ate_6_horas_	Saindo_de_c	5	Nao	NULL	N	NULL
Nao	NULL	Voce_esta_t	de_6_a_10_	Saindo_de_c	7	Nao	NULL	N	NULL
Nao	NULL	Voce_esta_t	ate_6_horas_	Saindo_de_c	8	Nao	NULL	N	NULL
Sim	Candidiase	Voce_esta_t	de_6_a_10_	Saindo_de_c	10	Nao	NULL	N	NULL
Nao	NULL	Voce_esta_t	mais_de_10_	De_quarente	10	Nao	NULL	N	NULL
Nao	NULL	Voce_esta_t	ate_6_horas_	Saindo_de_c	6	Nao	NULL	N	NULL
Nao	NULL	Voce_esta_t	de_6_a_10_	Saindo_de_c	10	Sim	1_-_5_anos	5	Cabeca-_Om
Nao	NULL	Voce_esta_t	de_6_a_10_	Saindo_de_c	10	Nao	NULL	N	NULL
Nao	NULL	Voce_esta_t	ate_6_horas_	Saindo_de_c	8	Nao	NULL	N	NULL
Nao	NULL	Voce_esta_t	ate_6_horas_	Saindo_de_c	9	Nao	NULL	N	NULL
Sim	Nutricionista	Voce_esta_t	de_6_a_10_	Saindo_de_c	8	Sim	1_-_5_anos	5	Cabeca-_Ce
Nao	NULL	Voce_esta_t	mais_de_10_	Saindo_de_c	7	Nao	NULL	N	NULL
Sim	Terapia	Voce_esta_t	de_6_a_10_	Saindo_de_c	10	Nao	NULL	N	NULL
Nao	NULL	Voce_esta_t	de_6_a_10_	Saindo_de_c	8	Nao	NULL	N	NULL
Nao	NULL	Voce_esta_t	de_6_a_10_	Saindo_de_c	4	Nao	NULL	N	NULL
Nao	NULL	Voce_esta_t	de_6_a_10_	Saindo_de_c	8	Nao	NULL	N	NULL
Sim	Acompanhar	Voce_esta_t	de_6_a_10_	Saindo_de_c	10	Nao	NULL	N	NULL
Nao	NULL	Voce_esta_t	ate_6_horas_	Saindo_de_c	5	Nao	NULL	N	NULL
Sim	Psicoterapia	Voce_esta_t	de_6_a_10_	De_quarente	10	Nao	NULL	N	NULL
Nao	NULL	Voce_esta_t	de_6_a_10_	Saindo_de_c	7	Sim	1_-_5_anos	3	Cervical-_pe
Nao	NULL	Voce_esta_t	ate_6_horas_	Saindo_de_c	0	Nao	NULL	N	NULL
Nao	NULL	Voce_esta_t	de_6_a_10_	Saindo_de_c	7	Nao	NULL	N	NULL
Nao	NULL	Voce_esta_t	de_6_a_10_	Saindo_de_c	10	Nao	NULL	N	NULL
Nao	NULL	Voce_esta_t	ate_6_horas_	Saindo_de_c	6	Nao	NULL	N	NULL
Nao	NULL	Voce_esta_t	ate_6_horas_	Saindo_de_c	1	Sim	Ha_mais_de_	1	Ombros
Nao	NULL	Voce_esta_t	de_6_a_10_	Saindo_de_c	7	Sim	1_-_5_anos	3	Ombros-_Joe
Sim	Terapia	Voce_esta_t	de_6_a_10_	Saindo_de_c	7	Sim	1_-_5_anos	4	Ombros-_Ce
Nao	NULL	Voce_esta_t	mais_de_10_	Saindo_de_c	5	Sim	1_-_5_anos	5	Cabeca-_Pu
Nao	NULL	Voce_esta_t	ate_6_horas_	Saindo_de_c	8	Nao	NULL	N	NULL
Nao	NULL	Voce_esta_t	de_6_a_10_	Saindo_de_c	5	Sim	Nao_sabe_d	2	Cabeca-_Ce
Nao	NULL	Voce_esta_t	de_6_a_10_	Saindo_de_c	9	Nao	NULL	N	NULL
Nao	NULL	Voce_esta_t	ate_6_horas_	Saindo_de_c	9	Nao	NULL	N	NULL
Nao	NULL	Voce_esta_t	ate_6_horas_	Saindo_de_c	9	Sim	Nao_sabe_d	5	Cabeca-_Ma
Nao	NULL	Voce_esta_t	mais_de_10_	Saindo_de_c	9	Nao	NULL	N	NULL
Nao	NULL	Voce_esta_t	de_6_a_10_	Saindo_de_c	8	Nao	NULL	N	NULL

Nao	NULL	Voce_esta_t	de_6_a_10_l	Saindo_de_c	10	Nao	NULL	N	NULL
Nao	NULL	Voce_esta_t	de_6_a_10_l	Saindo_de_c	10	Nao	NULL	N	NULL
Nao	NULL	Voce_esta_t	de_6_a_10_l	Saindo_de_c	10	Nao	NULL	N	NULL
Nao	NULL	Voce_esta_t	de_6_a_10_l	Saindo_de_c	8	Nao	NULL	N	NULL
Nao	NULL	Voce_esta_t	de_6_a_10_l	Saindo_de_c	7	Nao	NULL	N	NULL
Nao	NULL	Voce_esta_t	de_6_a_10_l	Saindo_de_c	7	Nao	NULL	N	NULL
Nao	NULL	Voce_esta_t	de_6_a_10_l	Saindo_de_c	8	Sim	6_-_10_anos	6	Cervical-_pes
Nao	NULL	Voce_esta_t	de_6_a_10_l	De_quarente	2	Sim	1_-_5_anos	1	Cabeca-_Om
Nao	NULL	Voce_esta_t	de_6_a_10_l	Saindo_de_c	7	Nao	NULL	N	NULL
Nao	NULL	Voce_esta_t	de_6_a_10_l	Saindo_de_c	5	Sim	1_-_5_anos	6	Cabeca-_Ma
Nao	NULL	Voce_esta_t	ate_6_horas	Saindo_de_c	10	Nao	NULL	N	NULL
Nao	NULL	Voce_esta_t	de_6_a_10_l	Saindo_de_c	9	Nao	NULL	N	NULL
Sim	Sessoes_cor	Voce_esta_t	de_6_a_10_l	Saindo_de_c	8	Nao	NULL	N	NULL
Sim	Fisioterapia_	Voce_esta_t	de_6_a_10_l	Saindo_de_c	10	Nao	NULL	N	NULL
Nao	NULL	Voce_esta_t	de_6_a_10_l	Saindo_de_c	10	Nao	NULL	N	NULL
Nao	NULL	Voce_esta_t	de_6_a_10_l	Saindo_de_c	7	Nao	NULL	N	NULL
Nao	NULL	Voce_esta_t	ate_6_horas	Saindo_de_c	8	Nao	NULL	N	NULL
Nao	NULL	Voce_esta_t	de_6_a_10_l	Saindo_de_c	1	Nao	NULL	N	NULL
Nao	NULL	Voce_esta_t	de_6_a_10_l	Saindo_de_c	7	Nao	NULL	N	NULL
Nao	NULL	Voce_esta_t	de_6_a_10_l	Saindo_de_c	8	Nao	NULL	N	NULL
Nao	NULL	Voce_esta_t	de_6_a_10_l	Saindo_de_c	10	Nao	NULL	N	NULL
Nao	NULL	Voce_esta_t	de_6_a_10_l	Saindo_de_c	10	Sim	Menos_de_1	5	Coluna_lomb
Nao	NULL	Voce_esta_t	ate_6_horas	Saindo_de_c	7	Nao	NULL	N	NULL
Nao	NULL	Voce_esta_t	ate_6_horas	Saindo_de_c	8	Sim	1_-_5_anos	2	Punhos-_Col
Nao	NULL	Voce_esta_t	de_6_a_10_l	Saindo_de_c	10	Nao	NULL	N	NULL
Nao	NULL	Voce_esta_t	mais_de_10_	Saindo_de_c	3	Nao	NULL	N	NULL
Nao	NULL	Voce_esta_t	de_6_a_10_l	Saindo_de_c	3	Nao	NULL	N	NULL
Nao	NULL	Voce_esta_t	mais_de_10_	Saindo_de_c	10	Nao	NULL	N	NULL
Nao	NULL	Voce_esta_t	de_6_a_10_l	Saindo_de_c	9	Nao	NULL	N	NULL
Nao	NULL	Voce_esta_t	ate_6_horas	Saindo_de_c	10	Nao	NULL	N	NULL
Sim	Psicoterapias	Voce_esta_t	de_6_a_10_l	Saindo_de_c	7	Nao	NULL	N	NULL
Nao	NULL	Voce_esta_t	de_6_a_10_l	Saindo_de_c	0	Nao	NULL	N	NULL
Nao	NULL	Voce_esta_t	de_6_a_10_l	Saindo_de_c	5	Nao	NULL	N	NULL
Nao	NULL	Voce_esta_t	de_6_a_10_l	Saindo_de_c	9	Nao	NULL	N	NULL
Nao	NULL	Voce_esta_t	de_6_a_10_l	Saindo_de_c	6	Nao	NULL	N	NULL
Nao	NULL	Voce_esta_t	de_6_a_10_l	Saindo_de_c	10	Nao	NULL	N	NULL
Nao	NULL	Voce_esta_t	ate_6_horas	Saindo_de_c	9	Nao	NULL	N	NULL
Sim	Fiz_dieta_e_	Voce_esta_t	mais_de_10_	Saindo_de_c	6	Nao	NULL	N	NULL
Nao	NULL	Voce_esta_t	de_6_a_10_l	De_quarente	10	Sim	6_-_10_anos	5	Ombros-_Ce
Nao	NULL	Voce_esta_t	de_6_a_10_l	Saindo_de_c	7	Nao	NULL	N	NULL
Nao	NULL	Voce_esta_t	de_6_a_10_l	Saindo_de_c	8	Sim	1_-_5_anos	7	Ombros-_Ce
Nao	NULL	Voce_esta_t	de_6_a_10_l	Saindo_de_c	7	Nao	NULL	N	NULL
Nao	NULL	Voce_esta_t	mais_de_10_	Saindo_de_c	5	Sim	Ha_mais_de_	4	Punhos-_Cer
Sim	Psicoterapia_	Voce_esta_t	de_6_a_10_l	Saindo_de_c	10	Nao	NULL	N	NULL
Nao	NULL	Voce_esta_t	mais_de_10_	De_quarente	0	Sim	Ha_mais_de_	4	Punhos-_Joe
Nao	NULL	Voce_esta_t	ate_6_horas	Saindo_de_c	10	Sim	1_-_5_anos	4	Cabeca-_Col
Nao	NULL	Voce_esta_t	de_6_a_10_l	Saindo_de_c	2	Nao	NULL	N	NULL
Nao	NULL	Voce_esta_t	de_6_a_10_l	Saindo_de_c	3	Sim	1_-_5_anos	3	Coluna_lomb
Nao	NULL	Voce_esta_t	mais_de_10_	Saindo_de_c	10	Nao	NULL	N	NULL
Nao	NULL	Voce_esta_t	de_6_a_10_l	De_quarente	4	Nao	NULL	N	NULL
Nao	NULL	Voce_esta_t	de_6_a_10_l	Saindo_de_c	7	Nao	NULL	N	NULL
Sim	Acompanhar	Voce_esta_t	de_6_a_10_l	Saindo_de_c	8	Sim	6_-_10_anos	4	Cabeca
Nao	NULL	Voce_esta_t	de_6_a_10_l	Saindo_de_c	10	Nao	NULL	N	NULL
Nao	NULL	Voce_esta_t	de_6_a_10_l	Saindo_de_c	3	Nao	NULL	N	NULL
Nao	NULL	Voce_esta_t	de_6_a_10_l	Saindo_de_c	2	Nao	NULL	N	NULL
Nao	NULL	Voce_esta_t	ate_6_horas	Saindo_de_c	3	Nao	NULL	N	NULL
Nao	NULL	Voce_esta_t	de_6_a_10_l	Saindo_de_c	7	Nao	NULL	N	NULL
Nao	NULL	Voce_esta_t	ate_6_horas	Saindo_de_c	1	Nao	NULL	N	NULL

Nao	NULL	Voce_esta_t	de_6_a_10_l	Saindo_de_c	10	Nao	NULL	N	NULL
Sim	fisioterapia_p	Voce_esta_t	ate_6_horas	Saindo_de_c	10	Nao	NULL	N	NULL
Nao	NULL	Voce_esta_t	de_6_a_10_l	Saindo_de_c	8	Sim	Ha_mais_de	7	Cabeca-_Om
Nao	NULL	Voce_esta_t	de_6_a_10_l	Saindo_de_c	9	Sim	Nao_sabe_d	5	Cabeca-_Ce
Nao	NULL	Voce_esta_t	ate_6_horas	Saindo_de_c	4	Sim	1_-_5_anos	2	Cervical-_pe
Nao	NULL	Voce_esta_t	de_6_a_10_l	Saindo_de_c	7	Nao	NULL	N	NULL
Nao	NULL	Voce_esta_t	de_6_a_10_l	Saindo_de_c	10	Nao	NULL	N	NULL
Nao	NULL	Voce_esta_t	mais_de_10	Saindo_de_c	9	Nao	NULL	N	NULL
Nao	NULL	Voce_esta_t	ate_6_horas	Saindo_de_c	10	Nao	NULL	N	NULL
Nao	NULL	Voce_esta_t	de_6_a_10_l	Saindo_de_c	7	Nao	NULL	N	NULL
Nao	NULL	Voce_esta_t	de_6_a_10_l	Saindo_de_c	9	Nao	NULL	N	NULL
Nao	NULL	Voce_esta_t	mais_de_10	Saindo_de_c	9	Nao	NULL	N	NULL
Nao	NULL	Voce_esta_t	mais_de_10	Saindo_de_c	10	Nao	NULL	N	NULL
Nao	NULL	Voce_esta_t	de_6_a_10_l	Saindo_de_c	7	Nao	NULL	N	NULL
Nao	NULL	Voce_esta_t	de_6_a_10_l	Saindo_de_c	8	Nao	NULL	N	NULL
Nao	NULL	Voce_esta_t	ate_6_horas	Saindo_de_c	8	Sim	1_-_5_anos	6	Cervical-_pe
Nao	NULL	Voce_esta_t	ate_6_horas	Saindo_de_c	3	Nao	NULL	N	NULL
Nao	NULL	Voce_esta_t	mais_de_10	Saindo_de_c	10	Nao	NULL	N	NULL
Nao	NULL	Voce_esta_t	de_6_a_10_l	Saindo_de_c	7	Nao	NULL	N	NULL
Nao	NULL	Voce_esta_t	de_6_a_10_l	Saindo_de_c	10	Nao	NULL	N	NULL
Nao	NULL	Voce_esta_t	ate_6_horas	Saindo_de_c	8	Nao	NULL	N	NULL
Sim	Tireoide_	Voce_esta_t	mais_de_10	Saindo_de_c	8	Nao	NULL	N	NULL
Nao	NULL	Voce_esta_t	de_6_a_10_l	Saindo_de_c	8	Nao	NULL	N	NULL
Sim	Rosacea	Voce_esta_t	de_6_a_10_l	Saindo_de_c	5	Nao	NULL	N	NULL
Nao	NULL	Voce_esta_t	de_6_a_10_l	Saindo_de_c	4	Sim	1_-_5_anos	4	Coluna_lomb
Nao	NULL	Voce_esta_t	de_6_a_10_l	Saindo_de_c	2	Sim	Ha_mais_de	8	Cabeca-_Ma
Nao	NULL	Voce_esta_t	de_6_a_10_l	Saindo_de_c	7	Nao	NULL	N	NULL
Sim	Terapia	Voce_esta_t	ate_6_horas	Saindo_de_c	4	Nao	NULL	N	NULL
Nao	NULL	Voce_esta_t	mais_de_10	Saindo_de_c	10	Nao	NULL	N	NULL
Nao	NULL	Voce_esta_t	mais_de_10	Saindo_de_c	3	Nao	NULL	N	NULL
Sim	Tratamento_	Voce_esta_t	mais_de_10	Saindo_de_c	9	Sim	Ha_mais_de	5	Cabeca
Nao	NULL	Voce_esta_t	de_6_a_10_l	Saindo_de_c	5	Sim	1_-_5_anos	5	Coluna_lomb
Nao	NULL	Voce_esta_t	de_6_a_10_l	Saindo_de_c	7	Sim	1_-_5_anos	2	Coluna_lomb
Nao	NULL	Voce_esta_t	mais_de_10	De_quarente	8	Nao	NULL	N	NULL
Nao	NULL	Voce_esta_t	de_6_a_10_l	Saindo_de_c	7	Nao	NULL	N	NULL
Nao	NULL	Voce_esta_t	ate_6_horas	Saindo_de_c	0	Nao	NULL	N	NULL
Nao	NULL	Voce_esta_t	de_6_a_10_l	Saindo_de_c	6	Nao	NULL	N	NULL
Nao	NULL	Voce_esta_t	mais_de_10	Saindo_de_c	3	Nao	NULL	N	NULL
Nao	NULL	Voce_esta_t	de_6_a_10_l	Saindo_de_c	7	Nao	NULL	N	NULL
Sim	Para_enxaqu	Voce_esta_t	de_6_a_10_l	Saindo_de_c	8	Nao	NULL	N	NULL
Nao	NULL	Voce_esta_t	de_6_a_10_l	Saindo_de_c	4	Nao	NULL	N	NULL
Nao	NULL	Voce_esta_t	de_6_a_10_l	Saindo_de_c	10	Nao	NULL	N	NULL
Nao	NULL	Voce_esta_t	mais_de_10	Saindo_de_c	1	Nao	NULL	N	NULL
Nao	NULL	Voce_esta_t	de_6_a_10_l	Saindo_de_c	4	Nao	NULL	N	NULL
Nao	NULL	Voce_esta_t	ate_6_horas	Saindo_de_c	3	Nao	NULL	N	NULL
Sim	Psicologo_	Voce_esta_t	de_6_a_10_l	Saindo_de_c	8	Sim	1_-_5_anos	9	Coluna_lomb
Sim	somento_o_(	Voce_esta_t	de_6_a_10_l	Saindo_de_c	8	Nao	NULL	N	NULL
Nao	NULL	Voce_esta_t	de_6_a_10_l	Saindo_de_c	7	Nao	NULL	N	NULL
Nao	NULL	Voce_esta_t	de_6_a_10_l	Saindo_de_c	3	Nao	NULL	N	NULL
Nao	NULL	Voce_esta_t	de_6_a_10_l	Saindo_de_c	7	Sim	1_-_5_anos	5	Ombros-_Pu
Nao	NULL	Voce_esta_t	de_6_a_10_l	De_quarente	7	Sim	1_-_5_anos	4	Cabeca-_Om
Sim	Hipertensao	Voce_esta_t	de_6_a_10_l	Saindo_de_c	10	Sim	1_-_5_anos	7	Cabeca-_Ma
Nao	NULL	Voce_esta_t	mais_de_10	Saindo_de_c	10	Nao	NULL	N	NULL
Nao	NULL	Voce_esta_t	de_6_a_10_l	Saindo_de_c	7	Sim	6_-_10_anos	6	Antebracos-_
Nao	NULL	Voce_esta_t	ate_6_horas	Saindo_de_c	7	Nao	NULL	N	NULL
Nao	NULL	Voce_esta_t	mais_de_10	Saindo_de_c	9	Nao	NULL	N	NULL
Nao	NULL	Voce_esta_t	de_6_a_10_l	Saindo_de_c	7	Nao	NULL	N	NULL
Nao	NULL	Voce_esta_t	de_6_a_10_l	Saindo_de_c	8	Nao	NULL	N	NULL

Nao	NULL	Voce_esta_t	de_6_a_10_l	Saindo_de_c	3	Nao	NULL	N	NULL
Nao	NULL	Voce_esta_t	de_6_a_10_l	Saindo_de_c	3	Nao	NULL	N	NULL
Nao	NULL	Voce_esta_t	de_6_a_10_l	Saindo_de_c	9	Sim	1_-_5_anos	5	Coluna_torac
Nao	NULL	Voce_esta_t	de_6_a_10_l	Saindo_de_c	5	Nao	NULL	N	NULL
Sim	Terapia_	Voce_esta_t	de_6_a_10_l	Saindo_de_c	10	Sim	6_-_10_anos	6	Cabeca-_Joe
Sim	Cabelo_	Voce_esta_t	mais_de_10_	Saindo_de_c	10	Nao	NULL	N	NULL
Nao	NULL	Voce_esta_t	de_6_a_10_l	Saindo_de_c	3	Sim	1_-_5_anos	2	Cervical-_pes
Nao	NULL	Voce_esta_t	de_6_a_10_l	Saindo_de_c	6	Nao	NULL	N	NULL
Nao	NULL	Voce_esta_t	de_6_a_10_l	Saindo_de_c	9	Nao	NULL	N	NULL
Nao	NULL	Voce_esta_t	mais_de_10_	Saindo_de_c	10	Nao	NULL	N	NULL
Sim	ulcera_estom	Voce_esta_t	ate_6_horas_	Saindo_de_c	10	Nao	NULL	N	NULL
Nao	NULL	Voce_esta_t	ate_6_horas_	Saindo_de_c	6	Nao	NULL	N	NULL
Nao	NULL	Voce_esta_t	de_6_a_10_l	Saindo_de_c	7	Nao	NULL	N	NULL
Nao	NULL	Voce_esta_t	de_6_a_10_l	Saindo_de_c	8	Sim	1_-_5_anos	5	Quadril
Sim	medicina_pre	Voce_esta_t	mais_de_10_	Saindo_de_c	5	Nao	NULL	N	NULL
Nao	NULL	Voce_esta_t	mais_de_10_	Saindo_de_c	3	Nao	NULL	N	NULL
Nao	NULL	Voce_esta_t	de_6_a_10_l	Saindo_de_c	10	Nao	NULL	N	NULL
Nao	NULL	Voce_esta_t	de_6_a_10_l	Saindo_de_c	10	Nao	NULL	N	NULL
Nao	NULL	Voce_esta_t	mais_de_10_	Saindo_de_c	8	Sim	6_-_10_anos	8	Joelhos
Nao	NULL	Voce_esta_t	de_6_a_10_l	Saindo_de_c	2	Nao	NULL	N	NULL
Nao	NULL	Voce_esta_t	de_6_a_10_l	Saindo_de_c	7	Nao	NULL	N	NULL
Nao	NULL	Voce_esta_t	ate_6_horas_	Saindo_de_c	5	Sim	1_-_5_anos	5	Cervical-_pes
Nao	NULL	Voce_esta_t	de_6_a_10_l	Saindo_de_c	5	Nao	NULL	N	NULL
Nao	NULL	Voce_esta_t	de_6_a_10_l	Saindo_de_c	8	Nao	NULL	N	NULL
Nao	NULL	Voce_esta_t	mais_de_10_	Saindo_de_c	9	Nao	NULL	N	NULL
Nao	NULL	Voce_esta_t	mais_de_10_	Saindo_de_c	5	Sim	1_-_5_anos	3	Coluna_torac
Nao	NULL	Voce_esta_t	de_6_a_10_l	De_quarente	10	Nao	NULL	N	NULL
Nao	NULL	Voce_esta_t	de_6_a_10_l	Saindo_de_c	2	Nao	NULL	N	NULL
Nao	NULL	Voce_esta_t	de_6_a_10_l	Saindo_de_c	7	Sim	6_-_10_anos	4	Coluna_lomb
Nao	NULL	Voce_esta_t	de_6_a_10_l	Saindo_de_c	5	Sim	Ha_mais_de_	4	Cabeca
Nao	NULL	Voce_esta_t	de_6_a_10_l	Saindo_de_c	2	Sim	1_-_5_anos	2	Coluna_lomb
Nao	NULL	Voce_esta_t	de_6_a_10_l	Saindo_de_c	0	Nao	NULL	N	NULL
Nao	NULL	Voce_esta_t	de_6_a_10_l	Saindo_de_c	9	Nao	NULL	N	NULL
Nao	NULL	Voce_esta_t	mais_de_10_	Saindo_de_c	6	Nao	NULL	N	NULL
Nao	NULL	Voce_esta_t	de_6_a_10_l	Saindo_de_c	7	Nao	NULL	N	NULL
Sim	Drenagem_li	Voce_esta_t	de_6_a_10_l	Saindo_de_c	10	Nao	NULL	N	NULL
Nao	NULL	Voce_esta_t	ate_6_horas_	Saindo_de_c	10	Sim	Nao_sabe_d	4	Ombros-_Ce
Nao	NULL	Voce_esta_t	de_6_a_10_l	Saindo_de_c	10	Nao	NULL	N	NULL
Nao	NULL	Voce_esta_t	de_6_a_10_l	Saindo_de_c	9	Nao	NULL	N	NULL
Nao	NULL	Voce_esta_t	de_6_a_10_l	Saindo_de_c	5	Nao	NULL	N	NULL
Sim	Aparelho_od	Voce_esta_t	de_6_a_10_l	Saindo_de_c	7	Nao	NULL	N	NULL
Nao	NULL	Voce_esta_t	de_6_a_10_l	Saindo_de_c	6	Nao	NULL	N	NULL
Nao	NULL	Voce_esta_t	de_6_a_10_l	Saindo_de_c	9	Nao	NULL	N	NULL
Nao	NULL	Voce_esta_t	de_6_a_10_l	Saindo_de_c	8	Nao	NULL	N	NULL
Nao	NULL	Voce_esta_t	de_6_a_10_l	Saindo_de_c	7	Nao	NULL	N	NULL
Nao	NULL	Voce_esta_t	mais_de_10_	Saindo_de_c	10	Nao	NULL	N	NULL
Nao	NULL	Voce_esta_t	de_6_a_10_l	Saindo_de_c	8	Nao	NULL	N	NULL
Nao	NULL	Voce_esta_t	de_6_a_10_l	Saindo_de_c	10	Nao	NULL	N	NULL
Nao	NULL	Voce_esta_t	de_6_a_10_l	Saindo_de_c	8	Nao	NULL	N	NULL
Nao	NULL	Voce_esta_t	ate_6_horas_	Saindo_de_c	8	Nao	NULL	N	NULL
Sim	Uso_de_CPA	Voce_esta_t	de_6_a_10_l	Saindo_de_c	8	Nao	NULL	N	NULL
Sim	Quiropraxia_	Voce_esta_t	de_6_a_10_l	Saindo_de_c	8	Nao	NULL	N	NULL
Nao	NULL	Voce_esta_t	de_6_a_10_l	Saindo_de_c	10	Nao	NULL	N	NULL
Nao	NULL	Voce_esta_t	ate_6_horas_	Saindo_de_c	7	Sim	1_-_5_anos	2	Cabeca-_Om
Nao	NULL	Voce_esta_t	mais_de_10_	Saindo_de_c	9	Nao	NULL	N	NULL
Sim	Ansiedade_e	Voce_esta_t	de_6_a_10_l	Saindo_de_c	7	Nao	NULL	N	NULL
Nao	NULL	Voce_esta_t	mais_de_10_	Saindo_de_c	6	Sim	Ha_mais_de_	4	Maos-_Joelh
Sim	Tive_um_pro	Trabalho_pri	de_6_a_10_l	Saindo_de_c	8	Nao	NULL	N	NULL

Nao	NULL	Voce_esta_t	de_6_a_10_l	Saindo_de_c	10	Sim	1_-_5_anos	4	Joelhos
Nao	NULL	Voce_esta_t	de_6_a_10_l	Saindo_de_c	10	Sim	1_-_5_anos	6	Coluna_lomb
Sim	Para_Sinrom	Voce_esta_t	ate_6_horas_	Saindo_de_c	8	Nao	NULL	N	NULL
Sim	Estou_gesta	Voce_esta_t	de_6_a_10_l	Saindo_de_c	8	Nao	NULL	N	NULL
Nao	NULL	Voce_esta_t	de_6_a_10_l	Saindo_de_c	10	Sim	1_-_5_anos	6	Cervical-_pe
Nao	NULL	Voce_esta_t	de_6_a_10_l	Saindo_de_c	10	Sim	1_-_5_anos	5	Cabeca-_Om
Nao	NULL	Voce_esta_t	de_6_a_10_l	Saindo_de_c	8	Nao	NULL	N	NULL
Sim	Terapia_com	Voce_esta_t	de_6_a_10_l	Saindo_de_c	7	Nao	NULL	N	NULL
Sim	Transfusoes_	Voce_esta_t	ate_6_horas_	Saindo_de_c	8	Nao	NULL	N	NULL
Nao	NULL	Voce_esta_t	de_6_a_10_l	Saindo_de_c	8	Nao	NULL	N	NULL
Nao	NULL	Voce_esta_t	mais_de_10_	Saindo_de_c	2	Nao	NULL	N	NULL
Sim	Tratamento_	Voce_esta_t	de_6_a_10_l	Saindo_de_c	0	Sim	Ha_mais_de_	8	Maos-_Cervi
Sim	Psicoterapia_	Voce_esta_t	de_6_a_10_l	Saindo_de_c	8	Nao	NULL	N	NULL
Nao	NULL	Voce_esta_t	ate_6_horas_	Saindo_de_c	10	Nao	NULL	N	NULL
Nao	NULL	Voce_esta_t	ate_6_horas_	Saindo_de_c	7	Nao	NULL	N	NULL
Nao	NULL	Voce_esta_t	de_6_a_10_l	Saindo_de_c	9	Sim	1_-_5_anos	5	Cabeca-_Col
Nao		Voce_esta_t	mais_de_10_	Saindo_de_c	8	Nao	NULL	N	NULL
Sim	Acompanhar	Voce_esta_t	ate_6_horas_	De_quarente	10	Sim	6_-_10_anos	4	Coluna_lomb
Sim	tratamento_p	Voce_esta_t	mais_de_10_	Saindo_de_c	10	Nao	NULL	N	NULL
Nao	NULL	Voce_esta_t	mais_de_10_	De_quarente	10	Nao	NULL	N	NULL
Nao	NULL	Voce_esta_t	de_6_a_10_l	Saindo_de_c	10	Nao	NULL	N	NULL
Nao	NULL	Voce_esta_t	mais_de_10_	Saindo_de_c	7	Nao	NULL	N	NULL
Nao	NULL	Voce_esta_t	ate_6_horas_	Saindo_de_c	10	Nao	NULL	N	NULL
Sim	Tomos_vario	Voce_esta_t	de_6_a_10_l	Saindo_de_c	4	Nao	NULL	N	NULL
Sim	limpeza_regu	Voce_esta_t	de_6_a_10_l	Saindo_de_c	8	Sim	1_-_5_anos	6	Coluna_lomb
Nao	NULL	Voce_esta_t	de_6_a_10_l	De_quarente	10	Nao	NULL	N	NULL
Nao	NULL	Voce_esta_t	de_6_a_10_l	Saindo_de_c	8	Nao	NULL	N	NULL
Nao	NULL	Voce_esta_t	de_6_a_10_l	Saindo_de_c	10	Nao	NULL	N	NULL
Nao	NULL	Voce_esta_t	mais_de_10_	Saindo_de_c	7	Nao	NULL	N	NULL
Nao	NULL	Voce_esta_t	mais_de_10_	Saindo_de_c	6	Nao	NULL	N	NULL
Nao	NULL	Voce_esta_t	de_6_a_10_l	Saindo_de_c	8	Nao	NULL	N	NULL
Nao	NULL	Voce_esta_t	ate_6_horas_	Saindo_de_c	10	Nao	NULL	N	NULL
Nao	NULL	Voce_esta_t	mais_de_10_	Saindo_de_c	10	Nao	NULL	N	NULL
Sim	Terapia	Voce_esta_t	de_6_a_10_l	De_quarente	7	Sim	1_-_5_anos	5	Maos-_Ombr
Nao	NULL	Voce_esta_t	de_6_a_10_l	Saindo_de_c	9	Nao	NULL	N	NULL
Nao	NULL	Voce_esta_t	de_6_a_10_l	Saindo_de_c	5	Nao	NULL	N	NULL
Sim	espinha_	Voce_esta_t	mais_de_10_	Saindo_de_c	7	Nao	NULL	N	NULL
Sim	Litiase_renal	Voce_esta_t	de_6_a_10_l	Saindo_de_c	5	Nao	NULL	N	NULL
Nao	NULL	Voce_esta_t	de_6_a_10_l	Saindo_de_c	10	Nao	NULL	N	NULL
Nao	NULL	Voce_esta_t	de_6_a_10_l	Saindo_de_c	3	Nao	NULL	N	NULL
Nao	NULL	Voce_esta_t	ate_6_horas_	Saindo_de_c	8	Sim	Ha_mais_de_	3	Cabeca-_Ce
Sim	psicoterapia	Voce_esta_t	ate_6_horas_	Saindo_de_c	8	Sim	6_-_10_anos	3	Ombros-_Ce
Nao	NULL	Voce_esta_t	de_6_a_10_l	Saindo_de_c	8	Nao	NULL	N	NULL
Sim	Terapia_para	Voce_esta_t	mais_de_10_	Saindo_de_c	10	Nao	NULL	N	NULL
Nao	NULL	Voce_esta_t	de_6_a_10_l	Saindo_de_c	5	Nao	NULL	N	NULL
Sim	acido_urico	Voce_esta_t	mais_de_10_	Saindo_de_c	8	Sim	6_-_10_anos	6	Cabeca-_Col
Sim	fisioterapia	Voce_esta_t	ate_6_horas_	Saindo_de_c	10	Nao	NULL	N	NULL
Nao	NULL	Voce_esta_t	de_6_a_10_l	Saindo_de_c	10	Nao	NULL	N	NULL
Nao	NULL	Voce_esta_t	de_6_a_10_l	Saindo_de_c	10	Nao	NULL	N	NULL
Nao	NULL	Voce_esta_t	de_6_a_10_l	Saindo_de_c	7	Nao	NULL	N	NULL
Nao	NULL	Voce_esta_t	de_6_a_10_l	Saindo_de_c	8	Nao	NULL	N	NULL
Sim	Psicoterapia_	Voce_esta_t	de_6_a_10_l	Saindo_de_c	10	Nao	NULL	N	NULL
Nao	NULL	Voce_esta_t	de_6_a_10_l	Saindo_de_c	4	Sim	1_-_5_anos	3	Ombros-_Ce
Nao	NULL	Voce_esta_t	mais_de_10_	Saindo_de_c	5	Nao	NULL	N	NULL
Nao	NULL	Voce_esta_t	mais_de_10_	Saindo_de_c	9	Sim	Nao_sabe_d	2	Joelhos-_Pe
Nao	NULL	Voce_esta_t	ate_6_horas_	Saindo_de_c	5	Nao	NULL	N	NULL
Nao	NULL	Voce_esta_t	ate_6_horas_	Saindo_de_c	0	Nao	NULL	N	NULL
Nao	NULL	Voce_esta_t	de_6_a_10_l	Saindo_de_c	8	Sim	1_-_5_anos	3	Calcanhar_

Nao	NULL	Voce_esta_t	de_6_a_10_l	Saindo_de_c	10	Nao	NULL	N	NULL
Nao	NULL	Voce_esta_t	mais_de_10_	Saindo_de_c	7	Nao	NULL	N	NULL
Nao	NULL	Voce_esta_t	de_6_a_10_l	Saindo_de_c	1	Nao	NULL	N	NULL
Sim	Sim	Voce_esta_t	de_6_a_10_l	Saindo_de_c	5	Nao	NULL	N	NULL
Nao	NULL	Voce_esta_t	de_6_a_10_l	Saindo_de_c	0	Nao	NULL	N	NULL
Sim	aplicacao_m	Voce_esta_t	de_6_a_10_l	Saindo_de_c	7	Nao	NULL	N	NULL
Sim	Fisioterapia_	Voce_esta_t	de_6_a_10_l	Saindo_de_c	8	Nao	NULL	N	NULL
Sim	Fisioterapia	Voce_esta_t	de_6_a_10_l	Saindo_de_c	10	Nao	NULL	N	NULL
Nao	NULL	Voce_esta_t	mais_de_10_	De_quarente	7	Nao	NULL	N	NULL
Nao	NULL	Voce_esta_t	mais_de_10_	Saindo_de_c	10	Nao	NULL	N	NULL
Sim	Para_gastrite	Voce_esta_t	de_6_a_10_l	Saindo_de_c	4	Sim	6_-_10_anos	8	Cabeca
Nao	NULL	Voce_esta_t	mais_de_10_	Saindo_de_c	10	Nao	NULL	N	NULL
Nao	NULL	Voce_esta_t	de_6_a_10_l	Saindo_de_c	5	Sim	Nao_sabe_d	4	Cabeca-_Col
Nao	NULL	Voce_esta_t	de_6_a_10_l	Saindo_de_c	3	Nao	NULL	N	NULL
Nao	NULL	Voce_esta_t	de_6_a_10_l	Saindo_de_c	5	Sim	1_-_5_anos	3	Coluna_torac
Sim	Terapia	Voce_esta_t	de_6_a_10_l	Saindo_de_c	5	Sim	Ñao_sabe_d	5	Cabeca-_Cer
Sim	Comecarei_f	Voce_esta_t	de_6_a_10_l	Saindo_de_c	9	Nao	NULL	N	NULL
Nao	NULL	Voce_esta_t	mais_de_10_	De_quarente	9	Nao	NULL	N	NULL
Nao	NULL	Voce_esta_t	de_6_a_10_l	Saindo_de_c	2	Nao	NULL	N	NULL
Nao	NULL	Voce_esta_t	de_6_a_10_l	De_quarente	10	Sim	1_-_5_anos	7	Ombros
Sim	Circulacao_	Voce_esta_t	ate_6_horas	Saindo_de_c	9	Sim	Nao_sabe_d	3	Ombros-_Co
Nao	NULL	Voce_esta_t	de_6_a_10_l	Saindo_de_c	10	Sim	Ha_mais_de_	6	Cabeca-_Om
Sim	Para_a_repo	Voce_esta_t	mais_de_10_	Saindo_de_c	9	Nao	NULL	N	NULL
Sim	Resistencia_	Voce_esta_t	de_6_a_10_l	Saindo_de_c	10	Nao	NULL	N	NULL
Sim	Para_hipotire	Voce_esta_t	de_6_a_10_l	Saindo_de_c	7	Sim	Menos_de_1	8	Maos-_Perna
Nao	NULL	Voce_esta_t	mais_de_10_	Saindo_de_c	9	Nao	NULL	N	NULL
Nao	NULL	Voce_esta_t	de_6_a_10_l	Saindo_de_c	0	Nao	NULL	N	NULL
Sim	Acomoanho_	Voce_esta_t	de_6_a_10_l	Saindo_de_c	9	Nao	NULL	N	NULL
Nao	NULL	Voce_esta_t	ate_6_horas	Saindo_de_c	7	Nao	NULL	N	NULL
Nao	NULL	Voce_esta_t	mais_de_10_	Saindo_de_c	7	Sim	Nao_sabe_d	4	Coluna_torac
Sim	Hipertensao	Voce_esta_t	de_6_a_10_l	Saindo_de_c	9	Nao	NULL	N	NULL
Nao	NULL	Voce_esta_t	ate_6_horas	Saindo_de_c	4	Sim	1_-_5_anos	5	Cabeca-_Bra
Nao	NULL	Voce_esta_t	de_6_a_10_l	Saindo_de_c	8	Nao	NULL	N	NULL
Nao	NULL	Voce_esta_t	de_6_a_10_l	Saindo_de_c	5	Sim	1_-_5_anos	5	Ombros
Nao	NULL	Voce_esta_t	de_6_a_10_l	Saindo_de_c	2	Nao	NULL	N	NULL
Nao	NULL	Voce_esta_t	de_6_a_10_l	Saindo_de_c	10	Nao	NULL	N	NULL
Nao	NULL	Voce_esta_t	mais_de_10_	Saindo_de_c	7	Sim	Nao_sabe_d	3	Coluna_lomb
Nao	NULL	Voce_esta_t	de_6_a_10_l	Saindo_de_c	8	Sim	Ha_mais_de_	3	Coluna_lomb
Nao	NULL	Voce_esta_t	mais_de_10_	Saindo_de_c	10	Nao	NULL	N	NULL
Nao	NULL	Voce_esta_t	de_6_a_10_l	Saindo_de_c	8	Sim	1_-_5_anos	6	Cervical-_pe
Nao	NULL	Voce_esta_t	de_6_a_10_l	Saindo_de_c	2	Sim	6_-_10_anos	4	Maos-_Braco

questionario_para_aqueles_que_sentiam_dor_antes_do_inicio_da_p										questionario_para_aqueles_que_nao		
se_voce_ja_	voce_passo	se_passou_	caso_voce_	as	as	as	as	as	as	se_voce_na	se_sim_ha_	assinale_a_
NULL	NULL	NULL	NULL	NU	NU	NU	NU	NU	NU	Sim	Mais_de_6_r	Cabeca-_Om
Sim	Punhos-_Ce	menos_que_	Estresse-_Co	4	4	3	0	0	0	NULL	NULL	NULL
Sim	Nao	Nao_tive_no	Preocupacac	7	8	5	3	5	0	NULL	NULL	NULL
Sim	Nao	Nao_tive_no	Preocupacac	8	6	4	3	6	7	NULL	NULL	NULL
NULL	NULL	NULL	NULL	NU	NU	NU	NU	NU	NU	Sim	Mais_de_6_r	Cabeca-_Ma
NULL	NULL	NULL	NULL	NU	NU	NU	NU	NU	NU	Sim	Menos_de_6	Ombros-_Co
NULL	NULL	NULL	NULL	NU	NU	NU	NU	NU	NU	Nao	NULL	NULL
NULL	NULL	NULL	NULL	NU	NU	NU	NU	NU	NU	Nao	NULL	NULL
NULL	NULL	NULL	NULL	NU	NU	NU	NU	NU	NU	Sim	Mais_de_6_r	Maos-_Anteb
NULL	NULL	NULL	NULL	NU	NU	NU	NU	NU	NU	Sim	Menos_de_6	Lombar_part
NULL	NULL	NULL	NULL	NU	NU	NU	NU	NU	NU	Nao	NULL	NULL
NULL	NULL	NULL	NULL	NU	NU	NU	NU	NU	NU	Sim	Menos_de_6	Antebracos-_
Sim	Maos	menos_que_	Tenho_dor_r	6	3	5	5	3	2	NULL	NULL	NULL
NULL	NULL	NULL	NULL	NU	NU	NU	NU	NU	NU	Sim	Mais_de_6_r	Cervical_pes
NULL	NULL	NULL	NULL	NU	NU	NU	NU	NU	NU	Sim	Mais_de_6_r	Cabeca-_Ma
NULL	NULL	NULL	NULL	NU	NU	NU	NU	NU	NU	Sim	Mais_de_6_r	Cabeca-_Lor
NULL	NULL	NULL	NULL	NU	NU	NU	NU	NU	NU	Nao	NULL	NULL
Sim	Ombros-_Pe	mais_que_6_	Sedentarism	8	10	5	8	7	6	NULL	NULL	NULL
Sim	Cabeca	menos_que_	Preocupacac	8	10	9	6	9	9	NULL	NULL	NULL
NULL	NULL	NULL	NULL	NU	NU	NU	NU	NU	NU	Nao	NULL	NULL
NULL	NULL	NULL	NULL	NU	NU	NU	NU	NU	NU	Nao	NULL	NULL
NULL	NULL	NULL	NULL	NU	NU	NU	NU	NU	NU	Sim	Menos_de_6	Toracica_par
NULL	NULL	NULL	NULL	NU	NU	NU	NU	NU	NU	Nao	NULL	NULL
NULL	NULL	NULL	NULL	NU	NU	NU	NU	NU	NU	Sim	Mais_de_6_r	Maos-_Anteb
NULL	NULL	NULL	NULL	NU	NU	NU	NU	NU	NU	Sim	Mais_de_6_r	Cabeca-_Om
NULL	NULL	NULL	NULL	NU	NU	NU	NU	NU	NU	Nao	NULL	NULL
Sim	Cabeca-_Om	mais_que_6_	Sedentarism	8	7	3	4	7	8	NULL	NULL	NULL
NULL	NULL	NULL	NULL	NU	NU	NU	NU	NU	NU	Nao	NULL	NULL
NULL	NULL	NULL	NULL	NU	NU	NU	NU	NU	NU	Sim	Menos_de_6	Lombar_part
NULL	NULL	NULL	NULL	NU	NU	NU	NU	NU	NU	Sim	Mais_de_6_r	Cervical_pes
Sim	Punhos	menos_que_	Preocupacac	7	9	8	3	5	8	NULL	NULL	NULL
NULL	NULL	NULL	NULL	NU	NU	NU	NU	NU	NU	Sim	Menos_de_6	Ombros-_Pu
NULL	NULL	NULL	NULL	NU	NU	NU	NU	NU	NU	Nao	NULL	NULL
NULL	NULL	NULL	NULL	NU	NU	NU	NU	NU	NU	Sim	Mais_de_6_r	Cervical_pes
NULL	NULL	NULL	NULL	NU	NU	NU	NU	NU	NU	Nao	NULL	NULL
NULL	NULL	NULL	NULL	NU	NU	NU	NU	NU	NU	Sim	Menos_de_6	Lombar_part
NULL	NULL	NULL	NULL	NU	NU	NU	NU	NU	NU	Sim	Mais_de_6_r	Cabeca-_Om
NULL	NULL	NULL	NULL	NU	NU	NU	NU	NU	NU	Nao	NULL	NULL
NULL	NULL	NULL	NULL	NU	NU	NU	NU	NU	NU	Sim	Menos_de_6	Punhos-_Cer
Sim	Nao	Nao_tive_no	NULL	2	5	0	0	3	4	NULL	NULL	NULL
NULL	NULL	NULL	NULL	NU	NU	NU	NU	NU	NU	Nao	NULL	NULL
NULL	NULL	NULL	NULL	NU	NU	NU	NU	NU	NU	Nao	NULL	NULL
NULL	NULL	NULL	NULL	NU	NU	NU	NU	NU	NU	Nao	NULL	NULL
NULL	NULL	NULL	NULL	NU	NU	NU	NU	NU	NU	Sim	Mais_de_6_r	Ombros-_Ce
Sim	Cervical_pes	menos_que_	Preocupacac	9	10	10	3	10	10	NULL	NULL	NULL
Sim	Maos-_Colur	menos_que_	Condicoes_d	4	2	6	2	8	10	NULL	NULL	NULL
Sim	Nao	Nao_tive_no	Sedentarism	6	2	6	0	0	9	NULL	NULL	NULL
Sim	Ombros-_Co	mais_que_6_	Preocupacac	6	3	2	1	1	2	NULL	NULL	NULL
NULL	NULL	NULL	NULL	NU	NU	NU	NU	NU	NU	Nao	NULL	NULL
Sim	Nao	mais_que_6_	Sedentarism	7	1	9	4	7	9	NULL	NULL	NULL
NULL	NULL	NULL	NULL	NU	NU	NU	NU	NU	NU	Sim	Mais_de_6_r	Lombar_part
NULL	NULL	NULL	NULL	NU	NU	NU	NU	NU	NU	Sim	Menos_de_6	Cervical_pes
Sim	Nao	Nao_tive_no	NULL	5	6	6	8	6	7	NULL	NULL	NULL
NULL	NULL	NULL	NULL	NU	NU	NU	NU	NU	NU	Sim	Menos_de_6	Cabeca-_Ma
NULL	NULL	NULL	NULL	NU	NU	NU	NU	NU	NU	Sim	Menos_de_6	Cabeca-_Lor

NULL	NULL	NULL	NULL	NU	NU	NU	NU	NU	NU	Sim	Mais_de_6_r	Cabeca-_Ma
NULL	NULL	NULL	NULL	NU	NU	NU	NU	NU	NU	Sim	Menos_de_6	Quadril-_Joe
NULL	NULL	NULL	NULL	NU	NU	NU	NU	NU	NU	Sim	Menos_de_6	Cabeca-_Om
NULL	NULL	NULL	NULL	NU	NU	NU	NU	NU	NU	Sim	Menos_de_6	Toracica_par
NULL	NULL	NULL	NULL	NU	NU	NU	NU	NU	NU	Nao	NULL	NULL
NULL	NULL	NULL	NULL	NU	NU	NU	NU	NU	NU	Nao	NULL	NULL
Sim	Maos-_Pes	mais_que_6_	Sedentarism	8	8	8	5	7	7	NULL	NULL	NULL
Sim	Nao	Nao_tive_no	NULL	2	0	0	0	2	0	NULL	NULL	NULL
NULL	NULL	NULL	NULL	NU	NU	NU	NU	NU	NU	Sim	Menos_de_6	Ombros-_Ce
Sim	Nao	mais_que_6_	Condicoes_d	5	3	7	0	7	8	NULL	NULL	NULL
NULL	NULL	NULL	NULL	NU	NU	NU	NU	NU	NU	Nao	NULL	NULL
NULL	NULL	NULL	NULL	NU	NU	NU	NU	NU	NU	Sim	Mais_de_6_r	Cabeca-_Lor
NULL	NULL	NULL	NULL	NU	NU	NU	NU	NU	NU	Sim	Menos_de_6	Lombar_part
NULL	NULL	NULL	NULL	NU	NU	NU	NU	NU	NU	Sim	Menos_de_6	Cabeca-_Ma
NULL	NULL	NULL	NULL	NU	NU	NU	NU	NU	NU	Nao	NULL	NULL
NULL	NULL	NULL	NULL	NU	NU	NU	NU	NU	NU	Nao	NULL	NULL
NULL	NULL	NULL	NULL	NU	NU	NU	NU	NU	NU	Sim	Mais_de_6_r	Maos-_Anteb
NULL	NULL	NULL	NULL	NU	NU	NU	NU	NU	NU	Nao	NULL	NULL
NULL	NULL	NULL	NULL	NU	NU	NU	NU	NU	NU	Sim	Mais_de_6_r	Cotovelos-_(
NULL	NULL	NULL	NULL	NU	NU	NU	NU	NU	NU	Nao	NULL	NULL
NULL	NULL	NULL	NULL	NU	NU	NU	NU	NU	NU	Nao	NULL	NULL
Sim	Cervical_pes	menos_que_	Sedentarism	7	6	1	0	0	4	NULL	NULL	NULL
NULL	NULL	NULL	NULL	NU	NU	NU	NU	NU	NU	Nao	NULL	NULL
Sim	Ombros	menos_que_	Condicoes_d	4	6	6	3	0	4	NULL	NULL	NULL
NULL	NULL	NULL	NULL	NU	NU	NU	NU	NU	NU	Sim	Menos_de_6	Cabeca-_Om
NULL	NULL	NULL	NULL	NU	NU	NU	NU	NU	NU	Sim	Menos_de_6	Lombar_part
NULL	NULL	NULL	NULL	NU	NU	NU	NU	NU	NU	Nao	NULL	NULL
NULL	NULL	NULL	NULL	NU	NU	NU	NU	NU	NU	Sim	Menos_de_6	Cervical_pes
NULL	NULL	NULL	NULL	NU	NU	NU	NU	NU	NU	Sim	Mais_de_6_r	Cabeca-_Om
NULL	NULL	NULL	NULL	NU	NU	NU	NU	NU	NU	Nao	NULL	NULL
NULL	NULL	NULL	NULL	NU	NU	NU	NU	NU	NU	Sim	Mais_de_6_r	Cabeca-_Pu
NULL	NULL	NULL	NULL	NU	NU	NU	NU	NU	NU	Nao	NULL	NULL
NULL	NULL	NULL	NULL	NU	NU	NU	NU	NU	NU	Sim	Mais_de_6_r	Ombros-_Pu
NULL	NULL	NULL	NULL	NU	NU	NU	NU	NU	NU	Sim	Mais_de_6_r	Ombros-_Pu
NULL	NULL	NULL	NULL	NU	NU	NU	NU	NU	NU	Nao	NULL	NULL
NULL	NULL	NULL	NULL	NU	NU	NU	NU	NU	NU	Nao	NULL	NULL
NULL	NULL	NULL	NULL	NU	NU	NU	NU	NU	NU	Sim	Menos_de_6	Ombros-_Lor
NULL	NULL	NULL	NULL	NU	NU	NU	NU	NU	NU	Sim	Menos_de_6	Cabeca-_Ma
Sim	Cabeca-_Col	mais_que_6_	Preocupacac	9	8	10	6	5	9	NULL	NULL	NULL
NULL	NULL	NULL	NULL	NU	NU	NU	NU	NU	NU	Nao	NULL	NULL
Sim	Maos-_Anteb	mais_que_6_	Sedentarism	8	10	9	8	8	8	NULL	NULL	NULL
NULL	NULL	NULL	NULL	NU	NU	NU	NU	NU	NU	Nao	NULL	NULL
Sim	Cabeca-_Ma	menos_que_	Preocupacac	7	0	4	1	6	7	NULL	NULL	NULL
NULL	NULL	NULL	NULL	NU	NU	NU	NU	NU	NU	Sim	Mais_de_6_r	Cabeca-_Om
Sim	Nao	Nao_tive_no	Sedentarism	2	2	1	1	3	2	NULL	NULL	NULL
Sim	Maos-_Bracc	menos_que_	Sedentarism	4	7	4	8	9	8	NULL	NULL	NULL
NULL	NULL	NULL	NULL	NU	NU	NU	NU	NU	NU	Nao	NULL	NULL
Sim	Nao	Nao_tive_no	Sedentarism	4	0	3	0	8	0	NULL	NULL	NULL
NULL	NULL	NULL	NULL	NU	NU	NU	NU	NU	NU	Sim	Menos_de_6	Cabeca-_Pu
NULL	NULL	NULL	NULL	NU	NU	NU	NU	NU	NU	Sim	Mais_de_6_r	Punhos-_Lor
NULL	NULL	NULL	NULL	NU	NU	NU	NU	NU	NU	Sim	Menos_de_6	Toracica_par
Sim	Cabeca-_Pu	mais_que_6_	Preocupacac	8	9	6	9	4	4	NULL	NULL	NULL
NULL	NULL	NULL	NULL	NU	NU	NU	NU	NU	NU	Sim	Mais_de_6_r	Cabeca-_Tor
NULL	NULL	NULL	NULL	NU	NU	NU	NU	NU	NU	Nao	NULL	NULL
NULL	NULL	NULL	NULL	NU	NU	NU	NU	NU	NU	Sim	Mais_de_6_r	Cabeca-_Om
NULL	NULL	NULL	NULL	NU	NU	NU	NU	NU	NU	Nao	NULL	NULL
NULL	NULL	NULL	NULL	NU	NU	NU	NU	NU	NU	Sim	Mais_de_6_r	Cabeca-_Om
NULL	NULL	NULL	NULL	NU	NU	NU	NU	NU	NU	Nao	NULL	NULL

NULL	NULL	NULL	NULL	NU	NU	NU	NU	NU	NU	Nao	NULL	NULL
NULL	NULL	NULL	NULL	NU	NU	NU	NU	NU	NU	Sim	Menos_de_6	Cabeca-_Om
Sim	Joelhos-_Tor	mais_que_6_	Preocupacac	9	8	8	3	7	9	NULL	NULL	NULL
Sim	Joelhos	menos_que_	Preocupacac	6	7	5	5	6	4	NULL	NULL	NULL
Sim	Nao	Nao_tive_no	NULL	2	0	0	0	0	0	NULL	NULL	NULL
NULL	NULL	NULL	NULL	NU	NU	NU	NU	NU	NU	Nao	NULL	NULL
NULL	NULL	NULL	NULL	NU	NU	NU	NU	NU	NU	Nao	NULL	NULL
NULL	NULL	NULL	NULL	NU	NU	NU	NU	NU	NU	Sim	Mais_de_6_r	Cabeca-_Om
NULL	NULL	NULL	NULL	NU	NU	NU	NU	NU	NU	Nao	NULL	NULL
NULL	NULL	NULL	NULL	NU	NU	NU	NU	NU	NU	Nao	NULL	NULL
NULL	NULL	NULL	NULL	NU	NU	NU	NU	NU	NU	Nao	NULL	NULL
NULL	NULL	NULL	NULL	NU	NU	NU	NU	NU	NU	Sim	Menos_de_6	Cabeca-_Lor
NULL	NULL	NULL	NULL	NU	NU	NU	NU	NU	NU	Sim	Menos_de_6	Cabeca-_Qu
NULL	NULL	NULL	NULL	NU	NU	NU	NU	NU	NU	Sim	Menos_de_6	Toracica_par
NULL	NULL	NULL	NULL	NU	NU	NU	NU	NU	NU	Nao	NULL	NULL
Sim	Nao	Nao_tive_no	Isolamento_s	6	5	6	4	8	8	NULL	NULL	NULL
NULL	NULL	NULL	NULL	NU	NU	NU	NU	NU	NU	Nao	NULL	NULL
NULL	NULL	NULL	NULL	NU	NU	NU	NU	NU	NU	Sim	Mais_de_6_r	Cabeca-_Ma
NULL	NULL	NULL	NULL	NU	NU	NU	NU	NU	NU	Sim	Mais_de_6_r	Cabeca-_Ma
NULL	NULL	NULL	NULL	NU	NU	NU	NU	NU	NU	Sim	Menos_de_6	Abdomen
NULL	NULL	NULL	NULL	NU	NU	NU	NU	NU	NU	Sim	Menos_de_6	Maos-_Punh
NULL	NULL	NULL	NULL	NU	NU	NU	NU	NU	NU	Sim	Menos_de_6	Cabeca-_Cer
NULL	NULL	NULL	NULL	NU	NU	NU	NU	NU	NU	Sim	Menos_de_6	Cabeca-_Tor
NULL	NULL	NULL	NULL	NU	NU	NU	NU	NU	NU	Sim	Mais_de_6_r	Ombros-_Ce
Sim	Nao	Nao_tive_no	Sedentarism	6	3	7	3	7	7	NULL	NULL	NULL
Sim	Nao	Nao_tive_no	Preocupacac	7	8	9	8	9	8	NULL	NULL	NULL
NULL	NULL	NULL	NULL	NU	NU	NU	NU	NU	NU	Sim	Mais_de_6_r	Lombar_part
NULL	NULL	NULL	NULL	NU	NU	NU	NU	NU	NU	Nao	NULL	NULL
NULL	NULL	NULL	NULL	NU	NU	NU	NU	NU	NU	Sim	Menos_de_6	Punhos-_Cer
NULL	NULL	NULL	NULL	NU	NU	NU	NU	NU	NU	Sim	Mais_de_6_r	Cervical_pes
Sim	Antebracos-_	Nao_tive_no	Preocupacac	9	8	6	10	10	10	NULL	NULL	NULL
Sim	Nao	Nao_tive_no	Sedentarism	4	5	5	1	3	7	NULL	NULL	NULL
Sim	Nao	Nao_tive_no	Preocupacac	2	1	0	0	0	1	NULL	NULL	NULL
NULL	NULL	NULL	NULL	NU	NU	NU	NU	NU	NU	Nao	NULL	NULL
NULL	NULL	NULL	NULL	NU	NU	NU	NU	NU	NU	Sim	Menos_de_6	Joelhos-_Tor
NULL	NULL	NULL	NULL	NU	NU	NU	NU	NU	NU	Nao	NULL	NULL
NULL	NULL	NULL	NULL	NU	NU	NU	NU	NU	NU	Nao	NULL	NULL
NULL	NULL	NULL	NULL	NU	NU	NU	NU	NU	NU	Sim	Menos_de_6	Antebracos-_
NULL	NULL	NULL	NULL	NU	NU	NU	NU	NU	NU	Sim	Menos_de_6	Cabeca-_Lor
NULL	NULL	NULL	NULL	NU	NU	NU	NU	NU	NU	Nao	NULL	NULL
NULL	NULL	NULL	NULL	NU	NU	NU	NU	NU	NU	Nao	NULL	NULL
NULL	NULL	NULL	NULL	NU	NU	NU	NU	NU	NU	Sim	Mais_de_6_r	Cabeca-_Cer
NULL	NULL	NULL	NULL	NU	NU	NU	NU	NU	NU	Nao	NULL	NULL
NULL	NULL	NULL	NULL	NU	NU	NU	NU	NU	NU	Sim	Mais_de_6_r	Lombar_part
NULL	NULL	NULL	NULL	NU	NU	NU	NU	NU	NU	Nao	NULL	NULL
Sim	Nao	Nao_tive_no	Sedentarism	9	10	10	7	5	8	NULL	NULL	NULL
NULL	NULL	NULL	NULL	NU	NU	NU	NU	NU	NU	Nao	NULL	NULL
NULL	NULL	NULL	NULL	NU	NU	NU	NU	NU	NU	Sim	Mais_de_6_r	Punhos-_Lor
NULL	NULL	NULL	NULL	NU	NU	NU	NU	NU	NU	Sim	Mais_de_6_r	Cabeca-_Om
Sim	Nao	Nao_tive_no	Preocupacac	6	6	6	2	4	6	NULL	NULL	NULL
Sim	Nao	Nao_tive_no	Preocupacac	8	9	8	6	7	9	NULL	NULL	NULL
Sim	Nao	Nao_tive_no	Preocupacac	7	3	3	1	2	0	NULL	NULL	NULL
NULL	NULL	NULL	NULL	NU	NU	NU	NU	NU	NU	Nao	NULL	NULL
Sim	Nao	Nao_tive_no	NULL	6	3	5	0	0	3	NULL	NULL	NULL
NULL	NULL	NULL	NULL	NU	NU	NU	NU	NU	NU	Nao	NULL	NULL
NULL	NULL	NULL	NULL	NU	NU	NU	NU	NU	NU	Sim	Mais_de_6_r	Cabeca-_Om
NULL	NULL	NULL	NULL	NU	NU	NU	NU	NU	NU	Nao	NULL	NULL
NULL	NULL	NULL	NULL	NU	NU	NU	NU	NU	NU	Nao	NULL	NULL

NULL	NULL	NULL	NULL	NU	NU	NU	NU	NU	NU	Sim	Menos_de_6	Cervical_pes
NULL	NULL	NULL	NULL	NU	NU	NU	NU	NU	NU	Nao	NULL	NULL
Sim	Cabeca	menos_que_	Preocupacac	5	6	3	7	4	7	NULL	NULL	NULL
NULL	NULL	NULL	NULL	NU	NU	NU	NU	NU	NU	Sim	Menos_de_6	Ombros-_Ce
Sim	Maos-_Punh	menos_que_	Condicoes_d	7	1	2	0	0	0	NULL	NULL	NULL
NULL	NULL	NULL	NULL	NU	NU	NU	NU	NU	NU	Sim	Mais_de_6_r	Cabeca-_Pes
Sim	Nao	Nao_tive_no	Sedentarism	2	1	1	0	3	2	NULL	NULL	NULL
NULL	NULL	NULL	NULL	NU	NU	NU	NU	NU	NU	Nao	NULL	NULL
NULL	NULL	NULL	NULL	NU	NU	NU	NU	NU	NU	Sim	Menos_de_6	Cabeca-_Cer
NULL	NULL	NULL	NULL	NU	NU	NU	NU	NU	NU	Sim	Mais_de_6_r	Ombros-_Tor
NULL	NULL	NULL	NULL	NU	NU	NU	NU	NU	NU	Sim	Mais_de_6_r	Cabeca-_Pun
NULL	NULL	NULL	NULL	NU	NU	NU	NU	NU	NU	Nao	NULL	NULL
NULL	NULL	NULL	NULL	NU	NU	NU	NU	NU	NU	Nao	NULL	NULL
Sim	Nao	Nao_tive_no	NULL	4	1	3	0	0	0	NULL	NULL	NULL
NULL	NULL	NULL	NULL	NU	NU	NU	NU	NU	NU	Nao	NULL	NULL
NULL	NULL	NULL	NULL	NU	NU	NU	NU	NU	NU	Sim	Menos_de_6	Cabeca-_Om
NULL	NULL	NULL	NULL	NU	NU	NU	NU	NU	NU	Sim	Mais_de_6_r	Cabeca-_Pun
NULL	NULL	NULL	NULL	NU	NU	NU	NU	NU	NU	Nao	NULL	NULL
Sim	Ombros-_Pu	mais_que_6_	Preocupacac	9	10	10	10	10	7	NULL	NULL	NULL
NULL	NULL	NULL	NULL	NU	NU	NU	NU	NU	NU	Sim	Mais_de_6_r	Maos-_Lomb
NULL	NULL	NULL	NULL	NU	NU	NU	NU	NU	NU	Sim	Menos_de_6	Cervical_pes
Sim	Punhos	menos_que_	Sedentarism	7	2	5	0	6	7	NULL	NULL	NULL
NULL	NULL	NULL	NULL	NU	NU	NU	NU	NU	NU	Nao	NULL	NULL
NULL	NULL	NULL	NULL	NU	NU	NU	NU	NU	NU	Nao	NULL	NULL
NULL	NULL	NULL	NULL	NU	NU	NU	NU	NU	NU	Nao	NULL	NULL
Sim	Nao	Nao_tive_no	Preocupacac	6	7	6	5	6	2	NULL	NULL	NULL
NULL	NULL	NULL	NULL	NU	NU	NU	NU	NU	NU	Sim	Mais_de_6_r	Cabeca-_Cer
NULL	NULL	NULL	NULL	NU	NU	NU	NU	NU	NU	Nao	NULL	NULL
Sim	Cabeca-_Ma	mais_que_6_	Sedentarism	5	5	6	4	7	5	NULL	NULL	NULL
Sim	Cabeca	Nao_tive_no	Sedentarism	8	7	7	9	8	9	NULL	NULL	NULL
Sim	Ombros	mais_que_6_	falta_de_aco	6	9	7	2	8	1	NULL	NULL	NULL
NULL	NULL	NULL	NULL	NU	NU	NU	NU	NU	NU	Sim	Menos_de_6	Lombar_part
NULL	NULL	NULL	NULL	NU	NU	NU	NU	NU	NU	Sim	Mais_de_6_r	Cabeca-_Ant
NULL	NULL	NULL	NULL	NU	NU	NU	NU	NU	NU	Sim	Mais_de_6_r	Joelhos
NULL	NULL	NULL	NULL	NU	NU	NU	NU	NU	NU	Sim	Mais_de_6_r	Cabeca-_Om
NULL	NULL	NULL	NULL	NU	NU	NU	NU	NU	NU	Sim	Mais_de_6_r	Cabeca-_Lor
Sim	Cabeca-_Pro	mais_que_6_	Preocupacac	7	7	8	4	5	9	NULL	NULL	NULL
NULL	NULL	NULL	NULL	NU	NU	NU	NU	NU	NU	Nao	NULL	NULL
NULL	NULL	NULL	NULL	NU	NU	NU	NU	NU	NU	Sim	Mais_de_6_r	Cabeca-_Cer
NULL	NULL	NULL	NULL	NU	NU	NU	NU	NU	NU	Nao	NULL	NULL
NULL	NULL	NULL	NULL	NU	NU	NU	NU	NU	NU	Nao	NULL	NULL
NULL	NULL	NULL	NULL	NU	NU	NU	NU	NU	NU	Sim	Menos_de_6	Lombar_part
NULL	NULL	NULL	NULL	NU	NU	NU	NU	NU	NU	Sim	Mais_de_6_r	Ombros-_Co
NULL	NULL	NULL	NULL	NU	NU	NU	NU	NU	NU	Nao	NULL	NULL
NULL	NULL	NULL	NULL	NU	NU	NU	NU	NU	NU	Nao	NULL	NULL
NULL	NULL	NULL	NULL	NU	NU	NU	NU	NU	NU	Sim	Mais_de_6_r	Cabeca-_Om
NULL	NULL	NULL	NULL	NU	NU	NU	NU	NU	NU	Nao	NULL	NULL
NULL	NULL	NULL	NULL	NU	NU	NU	NU	NU	NU	Nao	NULL	NULL
NULL	NULL	NULL	NULL	NU	NU	NU	NU	NU	NU	Nao	NULL	NULL
NULL	NULL	NULL	NULL	NU	NU	NU	NU	NU	NU	Sim	Menos_de_6	Joelhos
NULL	NULL	NULL	NULL	NU	NU	NU	NU	NU	NU	Sim	Menos_de_6	Cervical_pes
NULL	NULL	NULL	NULL	NU	NU	NU	NU	NU	NU	Sim	Mais_de_6_r	Ombros-_Ce
NULL	NULL	NULL	NULL	NU	NU	NU	NU	NU	NU	Sim	Menos_de_6	Toracica_par
Sim	Coluna_torac	mais_que_6_	Sedentarism	5	8	5	6	5	9	NULL	NULL	NULL
NULL	NULL	NULL	NULL	NU	NU	NU	NU	NU	NU	Sim	Mais_de_6_r	Cervical_pes
NULL	NULL	NULL	NULL	NU	NU	NU	NU	NU	NU	Nao	NULL	NULL
Sim	Coluna_torac	menos_que_	Estresse	2	2	2	2	2	4	NULL	NULL	NULL
NULL	NULL	NULL	NULL	NU	NU	NU	NU	NU	NU	Sim	Mais_de_6_r	Cabeca-_Om

Sim	Punhos-_Col	mais_que_6_	Preocupacac	6	6	5	5	5	6	NULL	NULL	NULL
Sim	Ombros-_Ce	mais_que_6_	Sedentarism	8	7	6	9	4	7	NULL	NULL	NULL
NULL	NULL	NULL	NULL	NU	NU	NU	NU	NU	NU	Sim	Menos_de_6	Lombar_part
NULL	NULL	NULL	NULL	NU	NU	NU	NU	NU	NU	Sim	Mais_de_6_r	Lombar_part
Sim	Ombros-_Ce	mais_que_6_	Preocupacac	7	6	6	2	6	5	NULL	NULL	NULL
Sim	Cabeca-_Ma	mais_que_6_	Preocupacac	10	10	10	10	10	10	NULL	NULL	NULL
NULL	NULL	NULL	NULL	NU	NU	NU	NU	NU	NU	Sim	Mais_de_6_r	Cabeca-_Om
NULL	NULL	NULL	NULL	NU	NU	NU	NU	NU	NU	Nao	NULL	NULL
NULL	NULL	NULL	NULL	NU	NU	NU	NU	NU	NU	Sim	Menos_de_6	Pernas_princ
NULL	NULL	NULL	NULL	NU	NU	NU	NU	NU	NU	Nao	NULL	NULL
NULL	NULL	NULL	NULL	NU	NU	NU	NU	NU	NU	Nao	NULL	NULL
Sim	Aparelho_dig	menos_que_	Preocupacac	8	9	9	7	7	7	NULL	NULL	NULL
NULL	NULL	NULL	NULL	NU	NU	NU	NU	NU	NU	Nao	NULL	NULL
NULL	NULL	NULL	NULL	NU	NU	NU	NU	NU	NU	Sim	Mais_de_6_r	Ombros-_Lor
NULL	NULL	NULL	NULL	NU	NU	NU	NU	NU	NU	Nao	NULL	NULL
Sim	Nao	Nao_tive_no	Preocupacac	8	6	0	5	10	8	NULL	NULL	NULL
NULL	NULL	NULL	NULL	NU	NU	NU	NU	NU	NU	Sim	Menos_de_6	Punhos-_Cer
Sim	Cervical_pes	mais_que_6_	Preocupacac	6	9	7	5	5	8	NULL	NULL	NULL
NULL	NULL	NULL	NULL	NU	NU	NU	NU	NU	NU	Sim	Menos_de_6	Maos-_Anteb
NULL	NULL	NULL	NULL	NU	NU	NU	NU	NU	NU	Sim	Menos_de_6	Maos-_Cervi
NULL	NULL	NULL	NULL	NU	NU	NU	NU	NU	NU	Sim	Mais_de_6_r	Cabeca-_Om
NULL	NULL	NULL	NULL	NU	NU	NU	NU	NU	NU	Nao	NULL	NULL
NULL	NULL	NULL	NULL	NU	NU	NU	NU	NU	NU	Nao	NULL	NULL
NULL	NULL	NULL	NULL	NU	NU	NU	NU	NU	NU	Sim	Mais_de_6_r	Cabeca-_Om
Sim	Cabeca	mais_que_6_	Preocupacac	7	5	6	4	7	8	NULL	NULL	NULL
NULL	NULL	NULL	NULL	NU	NU	NU	NU	NU	NU	Nao	NULL	NULL
NULL	NULL	NULL	NULL	NU	NU	NU	NU	NU	NU	Nao	NULL	NULL
NULL	NULL	NULL	NULL	NU	NU	NU	NU	NU	NU	Nao	NULL	NULL
NULL	NULL	NULL	NULL	NU	NU	NU	NU	NU	NU	Sim	Mais_de_6_r	Ombros-_Lor
NULL	NULL	NULL	NULL	NU	NU	NU	NU	NU	NU	Nao	NULL	NULL
NULL	NULL	NULL	NULL	NU	NU	NU	NU	NU	NU	Nao	NULL	NULL
NULL	NULL	NULL	NULL	NU	NU	NU	NU	NU	NU	Nao	NULL	NULL
NULL	NULL	NULL	NULL	NU	NU	NU	NU	NU	NU	Sim	Mais_de_6_r	Cabeca-_Ma
Sim	Nao	Nao_tive_no	Sedentarism	7	8	5	3	5	7	NULL	NULL	NULL
NULL	NULL	NULL	NULL	NU	NU	NU	NU	NU	NU	Nao	NULL	NULL
NULL	NULL	NULL	NULL	NU	NU	NU	NU	NU	NU	Sim	Menos_de_6	Lombar_part
NULL	NULL	NULL	NULL	NU	NU	NU	NU	NU	NU	Sim	Menos_de_6	Cervical_pes
NULL	NULL	NULL	NULL	NU	NU	NU	NU	NU	NU	Sim	Mais_de_6_r	Lombar_part
NULL	NULL	NULL	NULL	NU	NU	NU	NU	NU	NU	Sim	Mais_de_6_r	Cervical_pes
NULL	NULL	NULL	NULL	NU	NU	NU	NU	NU	NU	Nao	NULL	NULL
Sim	Nao	Nao_tive_no	Condicoes_d	4	3	3	2	5	3	NULL	NULL	NULL
Sim	Cervical_pes	Nao_tive_no	Estresse-_Cc	7	8	6	7	6	3	NULL	NULL	NULL
NULL	NULL	NULL	NULL	NU	NU	NU	NU	NU	NU	Sim	Menos_de_6	Lombar_part
NULL	NULL	NULL	NULL	NU	NU	NU	NU	NU	NU	Nao	NULL	NULL
NULL	NULL	NULL	NULL	NU	NU	NU	NU	NU	NU	Sim	Mais_de_6_r	Lombar_part
Sim	Quadril	mais_que_6_	Estresse-_Cc	9	7	5	4	7	9	NULL	NULL	NULL
NULL	NULL	NULL	NULL	NU	NU	NU	NU	NU	NU	Sim	Mais_de_6_r	Lombar_part
NULL	NULL	NULL	NULL	NU	NU	NU	NU	NU	NU	Sim	Menos_de_6	Cabeca-_Om
NULL	NULL	NULL	NULL	NU	NU	NU	NU	NU	NU	Sim	Mais_de_6_r	Antebracos-_
NULL	NULL	NULL	NULL	NU	NU	NU	NU	NU	NU	Nao	NULL	NULL
NULL	NULL	NULL	NULL	NU	NU	NU	NU	NU	NU	Sim	Menos_de_6	Cabeca-_Ma
NULL	NULL	NULL	NULL	NU	NU	NU	NU	NU	NU	Sim	Mais_de_6_r	Lombar_part
Sim	Nao	Nao_tive_no	Condicoes_d	5	2	4	2	2	7	NULL	NULL	NULL
NULL	NULL	NULL	NULL	NU	NU	NU	NU	NU	NU	Nao	NULL	NULL
Sim	Nao	Nao_tive_no	NULL	2	0	2	0	0	0	NULL	NULL	NULL
NULL	NULL	NULL	NULL	NU	NU	NU	NU	NU	NU	Nao	NULL	NULL
NULL	NULL	NULL	NULL	NU	NU	NU	NU	NU	NU	Sim	Mais_de_6_r	Cervical_pes
Sim	Coluna_lomb	mais_que_6_	Condicoes_d	5	3	5	0	3	1	NULL	NULL	NULL

NULL	NULL	NULL	NULL	NU	NU	NU	NU	NU	NU	Nao	NULL	NULL
NULL	NULL	NULL	NULL	NU	NU	NU	NU	NU	NU	Nao	NULL	NULL
NULL	NULL	NULL	NULL	NU	NU	NU	NU	NU	NU	Sim	Menos_de_6	Maos-_Anteb
NULL	NULL	NULL	NULL	NU	NU	NU	NU	NU	NU	Nao	NULL	NULL
NULL	NULL	NULL	NULL	NU	NU	NU	NU	NU	NU	Nao	NULL	NULL
NULL	NULL	NULL	NULL	NU	NU	NU	NU	NU	NU	Sim	Mais_de_6_r	Punhos-_Lor
NULL	NULL	NULL	NULL	NU	NU	NU	NU	NU	NU	Sim	Mais_de_6_r	Cabeca-_Om
NULL	NULL	NULL	NULL	NU	NU	NU	NU	NU	NU	Nao	NULL	NULL
NULL	NULL	NULL	NULL	NU	NU	NU	NU	NU	NU	Nao	NULL	NULL
NULL	NULL	NULL	NULL	NU	NU	NU	NU	NU	NU	Nao	NULL	NULL
Sim	Ombros-_Co	mais_que_6_	Condicoes_d	5	3	3	0	4	0	NULL	NULL	NULL
NULL	NULL	NULL	NULL	NU	NU	NU	NU	NU	NU	Sim	Menos_de_6	Cabeca-_Om
Sim	Ombros-_Co	Nao_tive_no	Condicoes_d	4	4	4	4	4	2	NULL	NULL	NULL
NULL	NULL	NULL	NULL	NU	NU	NU	NU	NU	NU	Sim	Menos_de_6	CARDIACA
Sim	Coluna_lomb	mais_que_6_	Sedentarism	7	2	6	0	3	5	NULL	NULL	NULL
Sim	Bracos-_Oml	mais_que_6_	Preocupacac	8	7	9	6	7	8	NULL	NULL	NULL
NULL	NULL	NULL	NULL	NU	NU	NU	NU	NU	NU	Sim	Menos_de_6	Joelhos
NULL	NULL	NULL	NULL	NU	NU	NU	NU	NU	NU	Nao	NULL	NULL
NULL	NULL	NULL	NULL	NU	NU	NU	NU	NU	NU	Sim	Menos_de_6	Cabeca
Sim	Coluna_lomb	mais_que_6_	Preocupacac	7	10	6	7	7	10	NULL	NULL	NULL
Sim	Quadril-_Joe	menos_que_	Sedentarism	6	7	7	7	7	8	NULL	NULL	NULL
Sim	Nao	Nao_tive_no	Preocupacac	6	7	7	7	7	10	NULL	NULL	NULL
NULL	NULL	NULL	NULL	NU	NU	NU	NU	NU	NU	Sim	Mais_de_6_r	Cabeca-_Om
NULL	NULL	NULL	NULL	NU	NU	NU	NU	NU	NU	Sim	Menos_de_6	Ombros-_Pu
Sim	Quadril	menos_que_	Sedentarism	7	3	4	4	4	5	NULL	NULL	NULL
NULL	NULL	NULL	NULL	NU	NU	NU	NU	NU	NU	Sim	Mais_de_6_r	Ombros
NULL	NULL	NULL	NULL	NU	NU	NU	NU	NU	NU	Nao	NULL	NULL
NULL	NULL	NULL	NULL	NU	NU	NU	NU	NU	NU	Sim	Menos_de_6	Maos-_Cervi
NULL	NULL	NULL	NULL	NU	NU	NU	NU	NU	NU	Nao	NULL	NULL
Sim	Coluna_torac	Nao_tive_no	Sedentarism	3	3	3	3	4	2	NULL	NULL	NULL
NULL	NULL	NULL	NULL	NU	NU	NU	NU	NU	NU	Nao	NULL	NULL
Sim	Ombros	menos_que_	Sedentarism	9	8	6	7	8	1	NULL	NULL	NULL
NULL	NULL	NULL	NULL	NU	NU	NU	NU	NU	NU	Nao	NULL	NULL
Sim	Nao	Nao_tive_no	Condicoes_d	5	3	0	0	0	2	NULL	NULL	NULL
NULL	NULL	NULL	NULL	NU	NU	NU	NU	NU	NU	Nao	NULL	NULL
NULL	NULL	NULL	NULL	NU	NU	NU	NU	NU	NU	Sim	Mais_de_6_r	Lombar_part
Sim	Maos-_Punh	mais_que_6_	Sedentarism	5	7	7	3	3	3	NULL	NULL	NULL
Sim	Quadril	mais_que_6_	Condicoes_d	4	4	0	0	5	0	NULL	NULL	NULL
NULL	NULL	NULL	NULL	NU	NU	NU	NU	NU	NU	Sim	Mais_de_6_r	Cabeca-_Cer
Sim	Antebracos-_	mais_que_6_	Condicoes_d	6	7	8	6	6	4	NULL	NULL	NULL
Sim	Nao	Nao_tive_no	NULL	4	4	4	2	3	3	NULL	NULL	NULL

_sentiam_dor_antes_do_inicio_							Avaliacao_do_sono_e_Questionario_indice_de_Qualidade_do_Sono_						
caso_voce_	as	as	as	as	as	ass	durante_o_ultim	dura	duran	dura	durante_o_u	durante_o_u	durante_o_u
Estresse	5	8	4	8	5	1	00h	30_r	9h	8-9h	1_ou_2_veze	Nenhuma_nc	Menos_de_1
NULL	NU	NU	NU	NU	NU	NU	1h	10m	7h_-_8	4h_(	Nenhuma_nc	Nenhuma_nc	Nenhuma_nc
NULL	NU	NU	NU	NU	NU	NU	Entre_22_e_00h	De_:	Entre_	6_a_	1_ou_2_veze	Menos_de_1	Nenhuma_nc
NULL	NU	NU	NU	NU	NU	NU	00h	de_2	8h	6h_	3_ou_mais_\	3_ou_mais_\	3_ou_mais_\
Sedentarism	5	2	3	0	1	0	02h00	15_r	09h00	6_hc	1_ou_2_veze	1_ou_2_veze	Nenhuma_nc
Condicoes_d	4	6	3	2	5	6	2_da_manha	30	10	7-8	3_ou_mais_\	1_ou_2_veze	Menos_de_1
NULL	NU	NU	NU	NU	NU	NU	00h20	90	06h30	05h:	3_ou_mais_\	Nenhuma_nc	Nenhuma_nc
NULL	NU	NU	NU	NU	NU	NU	23_horas	30_r	7_-_8h	7_hc	1_ou_2_veze	Menos_de_1	Nenhuma_nc
Condicoes_d	7	8	2	0	6	7	00h00	40_r	8h00	5h_(	1_ou_2_veze	Nenhuma_nc	Nenhuma_nc
Sedentarism	4	3	3	2	1	5	Por_volta_da_01h	30_r	08h30	4_ou	3_ou_mais_\	3_ou_mais_\	Nenhuma_nc
NULL	NU	NU	NU	NU	NU	NU	2h30am	Mais	9h40a	5	3_ou_mais_\	Nenhuma_nc	1_ou_2_veze
Condicoes_d	5	7	5	5	7	0	23h30	20	7h00	8h	1_ou_2_veze	Menos_de_1	1_ou_2_veze
NULL	NU	NU	NU	NU	NU	NU	1-2_da_manha	15_r	9h_da_	6-8h	Menos_de_1	Menos_de_1	1_ou_2_veze
Sedentarism	2	1	0	1	6	3	Meia_noite	20_r	6_hora	6_hc	Menos_de_1	Nenhuma_nc	Nenhuma_nc
Sedentarism	7	8	7	7	7	8	Meia-noite	30	8h00	6_a_	1_ou_2_veze	1_ou_2_veze	3_ou_mais_\
Estresse	6	8	6	7	7	2	22~23	nao_	05~06	6~7	1_ou_2_veze	3_ou_mais_\	Nenhuma_nc
NULL	NU	NU	NU	NU	NU	NU	Meia_noite	20	7h	6h	Menos_de_1	Menos_de_1	3_ou_mais_\
NULL	NU	NU	NU	NU	NU	NU	23h30	20	6h30	7	Menos_de_1	Menos_de_1	Nenhuma_nc
NULL	NU	NU	NU	NU	NU	NU	00h00-01h00	00h:	08h00	08h(	1_ou_2_veze	Menos_de_1	1_ou_2_veze
NULL	NU	NU	NU	NU	NU	NU	23h00-22h00	Rapi	6h30a	7h3(	Nenhuma_nc	Nenhuma_nc	Nenhuma_nc
NULL	NU	NU	NU	NU	NU	NU	05	1	11	6	1_ou_2_veze	Nenhuma_nc	Nenhuma_nc
Sedentarism	3	1	1	0	0	0	0h30	30	7h20	6	1_ou_2_veze	1_ou_2_veze	Nenhuma_nc
NULL	NU	NU	NU	NU	NU	NU	23hrs_	0	8h00h	6-8_	Menos_de_1	Menos_de_1	Menos_de_1
Condicoes_d	7	8	8	7	8	7	22h	15_r	04h50	De_(	Nenhuma_nc	Nenhuma_nc	Menos_de_1
Selecionei_o	6	10	7	10	7	10	00h	Dep	06h_o	De_/	3_ou_mais_\	3_ou_mais_\	Nenhuma_nc
NULL	NU	NU	NU	NU	NU	NU	Meia_noite	Vint	8_hora	7	Menos_de_1	Menos_de_1	1_ou_2_veze
NULL	NU	NU	NU	NU	NU	NU	23	70	9	7	3_ou_mais_\	Nenhuma_nc	Menos_de_1
NULL	NU	NU	NU	NU	NU	NU	1h	90_r	8h	6h	Nenhuma_nc	3_ou_mais_\	3_ou_mais_\
Condicoes_d	6	5	6	2	8	8	00h00	10	9_hora	6_hc	1_ou_2_veze	Nenhuma_nc	Nenhuma_nc
Condicoes_d	6	4	5	2	3	8	02h00	30m	10h	6h	1_ou_2_veze	Nenhuma_nc	Nenhuma_nc
NULL	NU	NU	NU	NU	NU	NU	Meia_noite	10_r	8h	7h	1_ou_2_veze	Nenhuma_nc	Menos_de_1
Sedentarism	6	4	4	1	4	0	23h	30_r	8h30	7h_	1_ou_2_veze	Nenhuma_nc	Nenhuma_nc
NULL	NU	NU	NU	NU	NU	NU	00h_meia-noite	60_r	08h	7_hc	1_ou_2_veze	Menos_de_1	Nenhuma_nc
Condicoes_d	5	5	3	0	4	2	Sim	30	09h00	7	1_ou_2_veze	1_ou_2_veze	3_ou_mais_\
NULL	NU	NU	NU	NU	NU	NU	22h30	15	7h30	8	Menos_de_1	Menos_de_1	Menos_de_1
Sedentarism	4	1	1	0	2	1	01h00	20_r	Sim-_9	6h34	Nenhuma_nc	Menos_de_1	Nenhuma_nc
Preocupacac	7	10	8	10	10	10	23_horas	Mais	7horas	5_ou	3_ou_mais_\	3_ou_mais_\	3_ou_mais_\
NULL	NU	NU	NU	NU	NU	NU	00h	Muit	9h	7h	3_ou_mais_\	1_ou_2_veze	Nenhuma_nc
Condicoes_d	4	7	5	3	7	7	23h30	30_a	8h10	de_7	1_ou_2_veze	Menos_de_1	3_ou_mais_\
NULL	NU	NU	NU	NU	NU	NU	23	90	08	7h3(	3_ou_mais_\	Nenhuma_nc	Menos_de_1
NULL	NU	NU	NU	NU	NU	NU	1hr_da_manha	30m	10hrs	Em_	1_ou_2_veze	1_ou_2_veze	Nenhuma_nc
NULL	NU	NU	NU	NU	NU	NU	23h30	30_r	7h50	6-_7	Menos_de_1	Menos_de_1	Menos_de_1
NULL	NU	NU	NU	NU	NU	NU	23h00	15	6h30	7h	Menos_de_1	Nenhuma_nc	Nenhuma_nc
Sedentarism	3	4	4	3	3	5	01h	15_r	08h	7	1_ou_2_veze	1_ou_2_veze	Menos_de_1
NULL	NU	NU	NU	NU	NU	NU	23h	90m	6h30	5h	3_ou_mais_\	1_ou_2_veze	Menos_de_1
NULL	NU	NU	NU	NU	NU	NU	23	30	3	7	3_ou_mais_\	3_ou_mais_\	Nenhuma_nc
NULL	NU	NU	NU	NU	NU	NU	23h30	1h	07h30	7h	3_ou_mais_\	3_ou_mais_\	Nenhuma_nc
NULL	NU	NU	NU	NU	NU	NU	01h30	60_r	08h30	6_hc	1_ou_2_veze	3_ou_mais_\	Nenhuma_nc
NULL	NU	NU	NU	NU	NU	NU	23	90	08h30	8	3_ou_mais_\	1_ou_2_veze	Nenhuma_nc
NULL	NU	NU	NU	NU	NU	NU	23	20	8	7	1_ou_2_veze	Menos_de_1	Nenhuma_nc
Preocupacac	10	10	10	10	10	10	01h00	40_r	07h30	6_hc	Nenhuma_nc	Menos_de_1	Nenhuma_nc
Estresse	7	8	5	5	6	8	Por_volta_das_23	Cerc	6h	Entr	1_ou_2_veze	1_ou_2_veze	Menos_de_1
NULL	NU	NU	NU	NU	NU	NU	11h_12h	2h	10h	7h	3_ou_mais_\	3_ou_mais_\	3_ou_mais_\
Estresse	6	8	3	3	3	7	00h00	20_r	06h00	Em_	3_ou_mais_\	1_ou_2_veze	Menos_de_1
Sedentarism	4	2	1	0	2	3	1h_da_manha	Meia	8h	6h3(	1_ou_2_veze	Menos_de_1	Menos_de_1

Condicoes_d	8	7	7	6	9	8	22h	30m	8h	6h	1_ou_2_veze	3_ou_mais_\	3_ou_mais_\
Estresse	4	6	3	0	6	6	23h30	1_a_	5h55	5_a_	1_ou_2_veze	3_ou_mais_\	Nenhuma_nc
Condicoes_d	7	7	8	6	6	8	00h00h	60_r	6h30	5h0(	1_ou_2_veze	1_ou_2_veze	1_ou_2_veze
Condicoes_d	2	0	1	0	0	0	4h	40	12h	7	3_ou_mais_\	1_ou_2_veze	Nenhuma_nc
NULL	NU	NU	NU	NU	NU	NU	01h00	20	08h30	7h	1_ou_2_veze	Nenhuma_nc	Menos_de_1
NULL	NU	NU	NU	NU	NU	NU	22h	10_a	5h30	7h	Menos_de_1	1_ou_2_veze	1_ou_2_veze
NULL	NU	NU	NU	NU	NU	NU	00h	30_r	7h	7h	1_ou_2_veze	Menos_de_1	Nenhuma_nc
NULL	NU	NU	NU	NU	NU	NU	23h	10m	7h30	8h	Nenhuma_nc	Menos_de_1	Nenhuma_nc
Condicoes_d	5	8	3	5	2	3	22_hrs	1h3(	8h00	8_a_	3_ou_mais_\	Menos_de_1	3_ou_mais_\
NULL	NU	NU	NU	NU	NU	NU	12h_da_noite_	5_m	7h30	8h	Nenhuma_nc	Nenhuma_nc	Nenhuma_nc
NULL	NU	NU	NU	NU	NU	NU	23_hrs_	10	7	8_a_	Menos_de_1	Menos_de_1	1_ou_2_veze
Sedentarism	3	3	2	0	3	2	Meia_noite	Mais	8	7	1_ou_2_veze	Menos_de_1	Menos_de_1
Estresse	1	1	0	0	0	1	21h30	10	06h00	8	1_ou_2_veze	Menos_de_1	Menos_de_1
Condicoes_d	4	7	0	0	4	5	21h	10_r	06h30	Por_	Menos_de_1	Menos_de_1	Nenhuma_nc
NULL	NU	NU	NU	NU	NU	NU	22h	20_r	6h_da_	8h_(	Nenhuma_nc	Nenhuma_nc	Nenhuma_nc
NULL	NU	NU	NU	NU	NU	NU	22h30	20_r	6h	7h	Menos_de_1	Nenhuma_nc	Menos_de_1
Sedentarism	2	5	5	0	5	0	00h00	60	09h00	7	3_ou_mais_\	1_ou_2_veze	Menos_de_1
NULL	NU	NU	NU	NU	NU	NU	1h30_da_manha	10_r	10h_d	9h	Menos_de_1	Menos_de_1	1_ou_2_veze
Condicoes_d	4	6	3	0	0	6	23h00	30	07h30	7	1_ou_2_veze	1_ou_2_veze	1_ou_2_veze
NULL	NU	NU	NU	NU	NU	NU	23h-_meia_noite_	De_:	8h00	De_(	3_ou_mais_\	1_ou_2_veze	Nenhuma_nc
NULL	NU	NU	NU	NU	NU	NU	23h_00h	20_r	05h30	05h_	1_ou_2_veze	3_ou_mais_\	1_ou_2_veze
NULL	NU	NU	NU	NU	NU	NU	23h00	20	7h50	8	Nenhuma_nc	Nenhuma_nc	Nenhuma_nc
NULL	NU	NU	NU	NU	NU	NU	3_da_manha	10_a	10_da_	7_a_	Nenhuma_nc	3_ou_mais_\	Nenhuma_nc
NULL	NU	NU	NU	NU	NU	NU	23h	30	7h	7	Menos_de_1	1_ou_2_veze	Nenhuma_nc
Estresse	3	8	3	4	3	7	Meia_noite	Uma	sim-_9	5_a_	1_ou_2_veze	3_ou_mais_\	Menos_de_1
Condicoes_d	3	2	1	0	2	6	02h00	<15	07h30	7	Menos_de_1	Menos_de_1	Nenhuma_nc
NULL	NU	NU	NU	NU	NU	NU	00h30	20	07h50	7	Nenhuma_nc	Nenhuma_nc	Menos_de_1
Condicoes_d	3	0	0	0	0	1	22h	1h	7h	7h	3_ou_mais_\	1_ou_2_veze	1_ou_2_veze
Sedentarism	7	7	6	4	7	5	Depois_das_00h	Entr	7h00	6_hc	Menos_de_1	Menos_de_1	Nenhuma_nc
NULL	NU	NU	NU	NU	NU	NU	23h	20m	7h	6h	Nenhuma_nc	Nenhuma_nc	Nenhuma_nc
Estresse	8	10	9	10	8	10	00h00	+60_	07h	07h_	3_ou_mais_\	3_ou_mais_\	Nenhuma_nc
NULL	NU	NU	NU	NU	NU	NU	Por_volta_das_1:	Cerc	Entre_	Cerc	Nenhuma_nc	Nenhuma_nc	Nenhuma_nc
Estresse	4	5	3	3	5	0	00	25	8	7	1_ou_2_veze	3_ou_mais_\	Menos_de_1
Sedentarism	3	3	3	0	0	0	00h00	de_5	8h00	7-9	Menos_de_1	Menos_de_1	Menos_de_1
NULL	NU	NU	NU	NU	NU	NU	23	45	8	8	3_ou_mais_\	Menos_de_1	Nenhuma_nc
NULL	NU	NU	NU	NU	NU	NU	01h30	00h1	10h00	08h(	Menos_de_1	Menos_de_1	Nenhuma_nc
Condicoes_d	4	4	4	4	6	4	23	1h	9h	9	3_ou_mais_\	3_ou_mais_\	1_ou_2_veze
Condicoes_d	3	7	3	3	8	7	0h_Meia_Noite	30-4	8h30	8h	Menos_de_1	1_ou_2_veze	Nenhuma_nc
NULL	NU	NU	NU	NU	NU	NU	23h30	90	7h30	6	3_ou_mais_\	1_ou_2_veze	Menos_de_1
NULL	NU	NU	NU	NU	NU	NU	1h	30	9h30	7	Nenhuma_nc	3_ou_mais_\	Menos_de_1
NULL	NU	NU	NU	NU	NU	NU	0.0625	30_r	7_da_	4h3(	3_ou_mais_\	1_ou_2_veze	Menos_de_1
NULL	NU	NU	NU	NU	NU	NU	11h30	80	07	08	3_ou_mais_\	3_ou_mais_\	3_ou_mais_\
NULL	NU	NU	NU	NU	NU	NU	22	120	7h30	7	3_ou_mais_\	Menos_de_1	Nenhuma_nc
Estresse	4	8	8	8	4	0	24	20_r	05	6	Nenhuma_nc	Nenhuma_nc	Nenhuma_nc
NULL	NU	NU	NU	NU	NU	NU	00h30	60	06h00	4	3_ou_mais_\	3_ou_mais_\	1_ou_2_veze
NULL	NU	NU	NU	NU	NU	NU	23	20	7h00	7	Menos_de_1	1_ou_2_veze	Menos_de_1
NULL	NU	NU	NU	NU	NU	NU	00h00	30	09h00	8	1_ou_2_veze	1_ou_2_veze	3_ou_mais_\
NULL	NU	NU	NU	NU	NU	NU	Meia_noite	3_M	7h30	7	Nenhuma_nc	Menos_de_1	Menos_de_1
Condicoes_d	4	7	6	2	5	5	23h30	20-3	9h00	8_hc	Menos_de_1	1_ou_2_veze	Menos_de_1
Sedentarism	6	6	4	0	3	5	Todas	Mais	Tem_s	5h	3_ou_mais_\	Menos_de_1	Nenhuma_nc
Condicoes_d	3	2	1	0	3	1	01h00	150	08h30	7	1_ou_2_veze	Menos_de_1	Menos_de_1
NULL	NU	NU	NU	NU	NU	NU	Entre_dez_e_mei	No_	Oito_d	Seis	Menos_de_1	Menos_de_1	Nenhuma_nc
Sedentarism	7	3	5	2	5	7	00h	10_r	8horas	7	Menos_de_1	1_ou_2_veze	3_ou_mais_\
NULL	NU	NU	NU	NU	NU	NU	00h30	40	8h	7h	3_ou_mais_\	Nenhuma_nc	1_ou_2_veze
Estresse	4	8	3	6	4	10	22h	de_3	Nao_e	Tent	3_ou_mais_\	3_ou_mais_\	1_ou_2_veze
NULL	NU	NU	NU	NU	NU	NU	02h00	1h	10h	7h	3_ou_mais_\	Menos_de_1	Nenhuma_nc
Sedentarism	5	8	10	7	9	9	00h00	60	08h00	6	1_ou_2_veze	1_ou_2_veze	Menos_de_1
NULL	NU	NU	NU	NU	NU	NU	1h00	20_r	7_hora	6	Nenhuma_nc	Nenhuma_nc	Nenhuma_nc

NULL	NU	NU	NU	NU	NU	NU	Apos_as_22h	Em_	7h30	8h	3_ou_mais_\	3_ou_mais_\	1_ou_2_veze
Condicoes_d	5	2	8	1	8	10	10h	60m	9h	7_a_	1_ou_2_veze	1_ou_2_veze	Nenhuma_nc
NULL	NU	NU	NU	NU	NU	NU	22h	240r	8h30	6h3(	3_ou_mais_\	3_ou_mais_\	3_ou_mais_\
NULL	NU	NU	NU	NU	NU	NU	23h00	30	7h10	7	Nenhuma_nc	3_ou_mais_\	3_ou_mais_\
NULL	NU	NU	NU	NU	NU	NU	Meia_noite	15_r	08_hoi	07h3	Nenhuma_nc	Nenhuma_nc	Nenhuma_nc
NULL	NU	NU	NU	NU	NU	NU	22h00	15	05h30	7	Nenhuma_nc	Nenhuma_nc	Menos_de_1
NULL	NU	NU	NU	NU	NU	NU	22h	10m	5h	7h	Nenhuma_nc	Menos_de_1	Menos_de_1
Condicoes_d	3	1	1	1	2	1	00h00	60	7h00	6	1_ou_2_veze	Menos_de_1	Nenhuma_nc
NULL	NU	NU	NU	NU	NU	NU	23h00	5_m	06h45	7h	Menos_de_1	1_ou_2_veze	1_ou_2_veze
NULL	NU	NU	NU	NU	NU	NU	1h00	15	7h30	6	1_ou_2_veze	Nenhuma_nc	Nenhuma_nc
NULL	NU	NU	NU	NU	NU	NU	3_horas_da_man	Meia	11_hoi	7_hc	Menos_de_1	Menos_de_1	Nenhuma_nc
Condicoes_d	5	7	2	5	2	0	23	5	7h	8	Menos_de_1	Menos_de_1	Nenhuma_nc
Condicoes_d	3	1	1	0	3	2	23h55_00h00	60_r	6h00	5h_(	3_ou_mais_\	1_ou_2_veze	1_ou_2_veze
Mau_jeito	2	1	2	0	0	1	23h	20	6h30	5	Nenhuma_nc	Nenhuma_nc	Nenhuma_nc
NULL	NU	NU	NU	NU	NU	NU	23h	15m	7h30	8	Menos_de_1	Nenhuma_nc	Nenhuma_nc
NULL	NU	NU	NU	NU	NU	NU	3	30	10h00	7	3_ou_mais_\	Menos_de_1	Nenhuma_nc
NULL	NU	NU	NU	NU	NU	NU	01h00	5_m	09h00	8h	Nenhuma_nc	1_ou_2_veze	1_ou_2_veze
Condicoes_d	8	10	7	9	9	10	22h00	40	07h00	7	3_ou_mais_\	1_ou_2_veze	Menos_de_1
Sedentarism	4	4	2	6	8	5	00h00	60	08h00	07	3_ou_mais_\	Menos_de_1	Nenhuma_nc
Sedentarism	6	7	9	9	6	10	10	30	8	10	1_ou_2_veze	3_ou_mais_\	3_ou_mais_\
Cadeira_ruin	4	4	3	3	7	5	23h-00h	Mais	7h-8h	Por_	1_ou_2_veze	3_ou_mais_\	3_ou_mais_\
Estresse	8	7	7	7	8	5	23h00_h_	15_r	07h00	7	Nenhuma_nc	Nenhuma_nc	1_ou_2_veze
Sedentarism	4	6	2	2	4	4	22h	30m	06h30	7-_8	1_ou_2_veze	1_ou_2_veze	3_ou_mais_\
Condicoes_d	3	4	1	0	4	3	22h50	30m	06h30	7	1_ou_2_veze	Menos_de_1	1_ou_2_veze
NULL	NU	NU	NU	NU	NU	NU	2	40	10	8	3_ou_mais_\	1_ou_2_veze	Nenhuma_nc
NULL	NU	NU	NU	NU	NU	NU	23h	40m	7h	7h	3_ou_mais_\	3_ou_mais_\	1_ou_2_veze
Sedentarism	5	7	7	2	4	10	22h30	30_r	08h00	8_hc	Menos_de_1	3_ou_mais_\	3_ou_mais_\
NULL	NU	NU	NU	NU	NU	NU	00h	30	7h	7	1_ou_2_veze	3_ou_mais_\	3_ou_mais_\
Sedentarism	5	2	3	0	3	0	23h	30m	6h	6h_3	Menos_de_1	Menos_de_1	Menos_de_1
Sedentarism	4	3	0	2	0	3	24h00-1h00	30_r	7h30	6	Menos_de_1	1_ou_2_veze	Nenhuma_nc
NULL	NU	NU	NU	NU	NU	NU	Meis_noite_	Uma	7_da_	5	3_ou_mais_\	Nenhuma_nc	Nenhuma_nc
NULL	NU	NU	NU	NU	NU	NU	meia_noite_-_1h_	Nao	8h	7_hc	Nenhuma_nc	Nenhuma_nc	Nenhuma_nc
NULL	NU	NU	NU	NU	NU	NU	Sim-_a_00h00	15	8	8	Nenhuma_nc	Menos_de_1	Nenhuma_nc
NULL	NU	NU	NU	NU	NU	NU	22h30	20_r	5h15	5h	1_ou_2_veze	Nenhuma_nc	Nenhuma_nc
posicao_do_	2	2	0	0	1	0	01	2	8	6	Nenhuma_nc	Nenhuma_nc	Nenhuma_nc
NULL	NU	NU	NU	NU	NU	NU	23h	30m	7H	8h	1_ou_2_veze	Nenhuma_nc	1_ou_2_veze
NULL	NU	NU	NU	NU	NU	NU	23h30	10_r	4h	5h	Menos_de_1	Nenhuma_nc	Nenhuma_nc
Condicoes_d	9	5	8	6	5	5	22hs	30m	7hs	8hs	1_ou_2_veze	Nenhuma_nc	1_ou_2_veze
Estresse	4	2	1	0	0	2	23h00-_00h00	Depe	Depen	6_a_	1_ou_2_veze	Menos_de_1	Menos_de_1
NULL	NU	NU	NU	NU	NU	NU	02h00	50	10h00	7	3_ou_mais_\	1_ou_2_veze	Menos_de_1
NULL	NU	NU	NU	NU	NU	NU	23h30	5	7h00	7-5_	Nenhuma_nc	Menos_de_1	Nenhuma_nc
Estresse	6	6	5	5	5	6	Meia_noite	15_r	7h	6h	Menos_de_1	Menos_de_1	Nenhuma_nc
NULL	NU	NU	NU	NU	NU	NU	23hrs	40_r	7_hrs	6_a_	Nenhuma_nc	Menos_de_1	Menos_de_1
Exercicios_m	2	0	1	0	0	0	0.04166666667	30	0.375	8h	1_ou_2_veze	Nenhuma_nc	Menos_de_1
NULL	NU	NU	NU	NU	NU	NU	22	40	7	8	Menos_de_1	Menos_de_1	Nenhuma_nc
NULL	NU	NU	NU	NU	NU	NU	23	45	8	8	Menos_de_1	Nenhuma_nc	Menos_de_1
NULL	NU	NU	NU	NU	NU	NU	00h00	15	06h00	6	1_ou_2_veze	3_ou_mais_\	3_ou_mais_\
Estresse	3	7	6	6	7	9	23h30	1h	06h30	5h	3_ou_mais_\	3_ou_mais_\	3_ou_mais_\
soma_de_pra	4	3	3	3	3	7	22h00	10	6h20	8	Menos_de_1	Menos_de_1	Menos_de_1
NULL	NU	NU	NU	NU	NU	NU	meia_noite	de_3	9hr	6-7	3_ou_mais_\	3_ou_mais_\	Nenhuma_nc
NULL	NU	NU	NU	NU	NU	NU	1h30_da_manha	40_r	8h	6h	3_ou_mais_\	3_ou_mais_\	3_ou_mais_\
NULL	NU	NU	NU	NU	NU	NU	22	0	6h00	8	Nenhuma_nc	Menos_de_1	Nenhuma_nc
NULL	NU	NU	NU	NU	NU	NU	21h	60	6h	7h	Menos_de_1	Menos_de_1	Nenhuma_nc
NULL	NU	NU	NU	NU	NU	NU	23	10	06h30	De_(	Menos_de_1	Menos_de_1	Nenhuma_nc
NULL	NU	NU	NU	NU	NU	NU	00h00h	2_hc	9h00	6_hc	3_ou_mais_\	3_ou_mais_\	Menos_de_1
Condicoes_d	5	8	6	5	7	9	Meia_noite_	40	8h	5	Menos_de_1	3_ou_mais_\	Menos_de_1
NULL	NU	NU	NU	NU	NU	NU	22h	30_r	05h45	6h	1_ou_2_veze	1_ou_2_veze	1_ou_2_veze
NULL	NU	NU	NU	NU	NU	NU	23h00	30_r	08h00	9h	Menos_de_1	1_ou_2_veze	3_ou_mais_\

Condicoes_d	7	7	7	8	5	8	23hs	15_r	as_8h	7_a_	Nenhuma_nc	Menos_de_1	Nenhuma_nc
NULL	NU	NU	NU	NU	NU	NU	23h00	15	8h00	8	1_ou_2_veze	1_ou_2_veze	Menos_de_1
NULL	NU	NU	NU	NU	NU	NU	01h00	60	09h50	7h3	1_ou_2_veze	1_ou_2_veze	3_ou_mais_
Condicoes_d	6	3	3	2	4	2	23	10	7	7	Nenhuma_nc	Nenhuma_nc	Nenhuma_nc
NULL	NU	NU	NU	NU	NU	NU	00h30	30_r	7h30	7h	1_ou_2_veze	Menos_de_1	Nenhuma_nc
Condicoes_d	8	8	8	8	8	10	12h30min	60_r	08h40	Entr	3_ou_mais_	Menos_de_1	Nenhuma_nc
NULL	NU	NU	NU	NU	NU	NU	02h00	30	09h00	06h3	Menos_de_1	Nenhuma_nc	Nenhuma_nc
NULL	NU	NU	NU	NU	NU	NU	22h	Nao_	5h40m	7h4	Nenhuma_nc	Nenhuma_nc	Nenhuma_nc
Condicoes_d	6	5	3	3	5	7	22h30	20_r	6h40	8	Menos_de_1	1_ou_2_veze	Menos_de_1
Tenho_varia	5	1	0	0	1	7	1h	3h_a	7h20m	5h_a	1_ou_2_veze	3_ou_mais_	Nenhuma_nc
Sedentarism	4	9	7	10	10	9	21	180	12	8	3_ou_mais_	3_ou_mais_	3_ou_mais_
NULL	NU	NU	NU	NU	NU	NU	23h	15-3	7h	6-8	3_ou_mais_	1_ou_2_veze	1_ou_2_veze
NULL	NU	NU	NU	NU	NU	NU	21h30	20	06h00	8	Nenhuma_nc	1_ou_2_veze	Nenhuma_nc
NULL	NU	NU	NU	NU	NU	NU	23	30	07	07	Menos_de_1	Menos_de_1	Nenhuma_nc
NULL	NU	NU	NU	NU	NU	NU	23h30	1_hc	7h30	8_hr	1_ou_2_veze	Menos_de_1	Nenhuma_nc
Sedentarism	5	5	6	2	1	5	1_hora_da_manh	10_r	8_da_	7_hc	Nenhuma_nc	Menos_de_1	1_ou_2_veze
Condicoes_d	6	7	5	5	6	5	23_hrs	1_hr	7h30	7	3_ou_mais_	3_ou_mais_	Menos_de_1
NULL	NU	NU	NU	NU	NU	NU	22h	10m	6h	8h	Menos_de_1	Menos_de_1	Menos_de_1
NULL	NU	NU	NU	NU	NU	NU	22h30	5_m	Todos_	Uma	Nenhuma_nc	3_ou_mais_	Nenhuma_nc
Sedentarism	2	1	1	0	2	0	11h30	15	7h30	6	1_ou_2_veze	1_ou_2_veze	Nenhuma_nc
Condicoes_d	8	7	3	1	9	8	1h30	60	9h	6h3	1_ou_2_veze	Menos_de_1	Nenhuma_nc
NULL	NU	NU	NU	NU	NU	NU	23h30	30	8h00	7h3	Menos_de_1	Menos_de_1	1_ou_2_veze
NULL	NU	NU	NU	NU	NU	NU	23_horas	15_r	7_hora	7hor	1_ou_2_veze	3_ou_mais_	Menos_de_1
NULL	NU	NU	NU	NU	NU	NU	22h30	5	6h45	8	Nenhuma_nc	Nenhuma_nc	Nenhuma_nc
NULL	NU	NU	NU	NU	NU	NU	00h40	15_r	7h00	6	Menos_de_1	Nenhuma_nc	Nenhuma_nc
NULL	NU	NU	NU	NU	NU	NU	23h30	30_r	5h00_	6_hc	1_ou_2_veze	1_ou_2_veze	Menos_de_1
Preocupacac	5	7	4	6	3	3	1h00	60_r	9h00	7_a_	3_ou_mais_	3_ou_mais_	Menos_de_1
NULL	NU	NU	NU	NU	NU	NU	2	30	10	6	1_ou_2_veze	Nenhuma_nc	Nenhuma_nc
NULL	NU	NU	NU	NU	NU	NU	1h_da_manha	+--_	8h	7h	1_ou_2_veze	1_ou_2_veze	1_ou_2_veze
NULL	NU	NU	NU	NU	NU	NU	1_da_manha	30_r	7_hora	5_a_	1_ou_2_veze	3_ou_mais_	1_ou_2_veze
NULL	NU	NU	NU	NU	NU	NU	23h	20	7h	6	1_ou_2_veze	3_ou_mais_	1_ou_2_veze
Condicoes_d	3	1	2	0	1	0	23h30	15	7h00	7	Menos_de_1	Nenhuma_nc	Nenhuma_nc
Preocupacac	6	7	7	8	10	8	Meia_noite	120	08h00	6	3_ou_mais_	3_ou_mais_	Menos_de_1
Sedentarism	4	5	6	1	4	4	Meia_Noite	Cerc	8h_da_	8h	Menos_de_1	Menos_de_1	Menos_de_1
Estresse	7	5	5	5	8	7	3	1_hr	10	5_a_	3_ou_mais_	3_ou_mais_	Menos_de_1
Sedentarism	5	1	7	0	6	3	23h	90_r	5h50m	6_hc	1_ou_2_veze	1_ou_2_veze	1_ou_2_veze
NULL	NU	NU	NU	NU	NU	NU	22h00_horas	50_r	05h30	6_hc	3_ou_mais_	3_ou_mais_	Menos_de_1
NULL	NU	NU	NU	NU	NU	NU	23h30	5-10	7-7h3	7-8h	Nenhuma_nc	Nenhuma_nc	Menos_de_1
Condicoes_d	6	6	2	2	7	7	23h00	30	6h00	6	1_ou_2_veze	3_ou_mais_	Menos_de_1
NULL	NU	NU	NU	NU	NU	NU	23_hrs	10	6hrs	7_hr	Nenhuma_nc	Nenhuma_nc	Menos_de_1
NULL	NU	NU	NU	NU	NU	NU	10	30	5	6	1_ou_2_veze	1_ou_2_veze	Menos_de_1
Sedentarism	3	3	2	0	2	2	23_horas_	30	6	7	1_ou_2_veze	1_ou_2_veze	Menos_de_1
Condicoes_d	6	2	2	1	7	1	00h00	10	7	6	Nenhuma_nc	Nenhuma_nc	Nenhuma_nc
NULL	NU	NU	NU	NU	NU	NU	22h00	00h1	05h00	6h0	Nenhuma_nc	Menos_de_1	Menos_de_1
NULL	NU	NU	NU	NU	NU	NU	0h	30	8h	7h	Nenhuma_nc	Nenhuma_nc	Nenhuma_nc
Isolamento_s	4	2	8	0	6	10	23_horas	30-4	5_hora	Cerc	3_ou_mais_	3_ou_mais_	Nenhuma_nc
NULL	NU	NU	NU	NU	NU	NU	23h	30	8h30	7h	1_ou_2_veze	1_ou_2_veze	Nenhuma_nc
NULL	NU	NU	NU	NU	NU	NU	22h	6_a_	Sim_6	6_a_	1_ou_2_veze	1_ou_2_veze	3_ou_mais_
NULL	NU	NU	NU	NU	NU	NU	22h30	Pou	05h30	5_a_	Menos_de_1	Menos_de_1	Nenhuma_nc
Sedentarism	5	5	2	1	0	0	00h00	30m	8h00	6	Menos_de_1	Menos_de_1	Menos_de_1
Sedentarism	4	5	2	4	5	9	Meia_noite	Dez_	8_hora	7_hc	Menos_de_1	Menos_de_1	3_ou_mais_
Condicoes_d	5	8	7	8	9	6	Por_volta_das_2	Muit	8_hora	Pou	3_ou_mais_	3_ou_mais_	Menos_de_1
Preocupacac	6	9	8	7	7	6	23h	15_r	6h	5h	1_ou_2_veze	3_ou_mais_	Menos_de_1
NULL	NU	NU	NU	NU	NU	NU	23h00	10	7h30	6-7	Menos_de_1	3_ou_mais_	1_ou_2_veze
Condicao_fin	6	7	5	6	5	8	22	60	6	7	1_ou_2_veze	1_ou_2_veze	3_ou_mais_
NULL	NU	NU	NU	NU	NU	NU	22h00	30_r	7h00	7h3	Menos_de_1	Menos_de_1	Nenhuma_nc
NULL	NU	NU	NU	NU	NU	NU	01h00	15	06h30	5	1_ou_2_veze	Menos_de_1	Nenhuma_nc
Condicoes_d	8	9	2	1	1	7	22h	60	06h30	8h	1_ou_2_veze	3_ou_mais_	Menos_de_1

NULL	NU	NU	NU	NU	NU	NU	21	10	4	5	Nenhuma_nc	3_ou_mais_\	Nenhuma_nc
NULL	NU	NU	NU	NU	NU	NU	22_hs_60_	60_a	7_hs_	6_hc	3_ou_mais_\	3_ou_mais_\	1_ou_2_veze
Sedentarism	5	7	4	0	3	8	23_h	30_r	09	5h	3_ou_mais_\	3_ou_mais_\	3_ou_mais_\
Acredito_ser_	7	6	6	4	5	5	22h	20	6h30	7	Menos_de_1	Menos_de_1	3_ou_mais_\
NULL	NU	NU	NU	NU	NU	NU	20h30-21h00	15_r	6h00	8	Menos_de_1	Menos_de_1	1_ou_2_veze
NULL	NU	NU	NU	NU	NU	NU	23h00	40	06h30	6	1_ou_2_veze	1_ou_2_veze	1_ou_2_veze
Sedentarism	6	2	0	3	1	3	0h00	5	8	8	Nenhuma_nc	Menos_de_1	Nenhuma_nc
NULL	NU	NU	NU	NU	NU	NU	2h_da_madrugad	10-2	10h	7-8h	Nenhuma_nc	1_ou_2_veze	Nenhuma_nc
Sedentarism	5	3	4	0	0	3	23h30_a_24h	60	7h_ou_	Cerc	1_ou_2_veze	Nenhuma_nc	Nenhuma_nc
NULL	NU	NU	NU	NU	NU	NU	1h	30_r	8h_e_:	7h	Menos_de_1	Nenhuma_nc	Nenhuma_nc
NULL	NU	NU	NU	NU	NU	NU	1am	20m	7am	6h	3_ou_mais_\	Menos_de_1	Nenhuma_nc
NULL	NU	NU	NU	NU	NU	NU	23_hrs	120	7	6	3_ou_mais_\	3_ou_mais_\	3_ou_mais_\
NULL	NU	NU	NU	NU	NU	NU	22	180	7	9	Menos_de_1	Menos_de_1	3_ou_mais_\
Condicoes_d	2	0	2	0	0	0	2	30	8	6-8	Nenhuma_nc	Nenhuma_nc	Nenhuma_nc
NULL	NU	NU	NU	NU	NU	NU	22h45	15m	8hs	8_hs	Menos_de_1	Menos_de_1	Nenhuma_nc
NULL	NU	NU	NU	NU	NU	NU	23	60	7	6	3_ou_mais_\	1_ou_2_veze	Nenhuma_nc
Condicoes_d	6	9	3	0	3	7	22_horas	1_hc	4h30	5_hc	3_ou_mais_\	1_ou_2_veze	Nenhuma_nc
NULL	NU	NU	NU	NU	NU	NU	23h	30m	8h	7h	Nenhuma_nc	Nenhuma_nc	3_ou_mais_\
Condicoes_d	5	5	5	5	5	5	2_horas_da_mad	mais	08_ho	06_l	3_ou_mais_\	3_ou_mais_\	3_ou_mais_\
Sedentarism	5	1	1	1	1	2	22_horas	30_r	08_ho	05_l	Menos_de_1	3_ou_mais_\	Menos_de_1
Estresse	7	8	6	7	8	8	Meia_noite	60	7	6-7	1_ou_2_veze	1_ou_2_veze	Menos_de_1
NULL	NU	NU	NU	NU	NU	NU	24	10	10	10	Nenhuma_nc	Nenhuma_nc	Nenhuma_nc
NULL	NU	NU	NU	NU	NU	NU	1_da_manha_	20_r	5_hora	4_hc	1_ou_2_veze	3_ou_mais_\	3_ou_mais_\
Estresse	8	9	9	9	9	9	00h	1a_2	6h	4h	3_ou_mais_\	3_ou_mais_\	3_ou_mais_\
NULL	NU	NU	NU	NU	NU	NU	23h	60	7h40	7h	3_ou_mais_\	3_ou_mais_\	3_ou_mais_\
NULL	NU	NU	NU	NU	NU	NU	03h00	40	9	5	1_ou_2_veze	1_ou_2_veze	Menos_de_1
NULL	NU	NU	NU	NU	NU	NU	23h30	30	7h	7h	1_ou_2_veze	Menos_de_1	Menos_de_1
NULL	NU	NU	NU	NU	NU	NU	22h30	120r	6h	5h	3_ou_mais_\	3_ou_mais_\	Nenhuma_nc
Estresse	9	6	8	2	5	10	23_horas	30_r	7h30_l	6_hr	1_ou_2_veze	1_ou_2_veze	Menos_de_1
NULL	NU	NU	NU	NU	NU	NU	Entre_00_e_02	Ate_	07h00	Nao_	1_ou_2_veze	Nenhuma_nc	Nenhuma_nc
NULL	NU	NU	NU	NU	NU	NU	22	1	7	6	3_ou_mais_\	1_ou_2_veze	3_ou_mais_\
NULL	NU	NU	NU	NU	NU	NU	23_horas	1_hc	8_hora	8_hc	3_ou_mais_\	1_ou_2_veze	Menos_de_1
Condicoes_d	10	9	10	9	10	10	01h_da_madruga	1h-2	5-6h0(	4-5	3_ou_mais_\	3_ou_mais_\	1_ou_2_veze
NULL	NU	NU	NU	NU	NU	NU	0_h	15	7h30	6h	1_ou_2_veze	1_ou_2_veze	Nenhuma_nc
NULL	NU	NU	NU	NU	NU	NU	22h00	Pou	05h30	7_hc	Nenhuma_nc	Nenhuma_nc	Nenhuma_nc
Condicoes_d	2	2	1	1	1	1	23h00	0h3(	7h30	7_hc	Menos_de_1	Menos_de_1	Menos_de_1
Sedentarism	7	9	9	8	10	10	00h00	30_r	8h45	8h	1_ou_2_veze	Nenhuma_nc	Nenhuma_nc
Condicoes_d	5	5	5	8	5	8	Sim_	120	Sempr	6	3_ou_mais_\	3_ou_mais_\	3_ou_mais_\
Condicoes_d	7	4	7	3	4	8	Entre_3_e_5_da_	120	11h	4hor	3_ou_mais_\	3_ou_mais_\	3_ou_mais_\
NULL	NU	NU	NU	NU	NU	NU	23h00	30	6h30	7h	1_ou_2_veze	Nenhuma_nc	Nenhuma_nc
NULL	NU	NU	NU	NU	NU	NU	4h00	30	10	6	3_ou_mais_\	1_ou_2_veze	1_ou_2_veze
NULL	NU	NU	NU	NU	NU	NU	22h00	30_r	6h20	8	Menos_de_1	Menos_de_1	Nenhuma_nc
Sedentarism	4	2	2	1	3	3	23_h	15	6	6-7	Menos_de_1	3_ou_mais_\	Nenhuma_nc
NULL	NU	NU	NU	NU	NU	NU	23_maximo-_fds_	5	06h00	Entr	Nenhuma_nc	Nenhuma_nc	Menos_de_1
Sedentarism	5	6	3	3	2	5	00h00	10m	Entre_	7	Menos_de_1	Menos_de_1	Nenhuma_nc
NULL	NU	NU	NU	NU	NU	NU	0	10	30	5	Nenhuma_nc	Menos_de_1	Menos_de_1
Sedentarism	7	10	8	8	7	9	24h	30m	7h	6h	1_ou_2_veze	3_ou_mais_\	Menos_de_1
Condicoes_d	5	5	5	5	5	5	24	60	7	6	1_ou_2_veze	1_ou_2_veze	1_ou_2_veze
Condicoes_d	4	3	6	5	8	2	23_h	10_r	8h30	8_hc	Menos_de_1	Menos_de_1	1_ou_2_veze
NULL	NU	NU	NU	NU	NU	NU	3h00	15	11h30	8	Menos_de_1	Nenhuma_nc	Nenhuma_nc
Estresse	6	6	6	5	5	6	22h00_	40_r	6h30_	Gera	3_ou_mais_\	1_ou_2_veze	1_ou_2_veze
Estresse	7	8	10	8	9	10	22	30	7	6	Menos_de_1	3_ou_mais_\	Nenhuma_nc
NULL	NU	NU	NU	NU	NU	NU	23_horas	10_r	6_hora	7_hc	1_ou_2_veze	1_ou_2_veze	1_ou_2_veze
NULL	NU	NU	NU	NU	NU	NU	23h00_h	30_r	6_hora	6_a_	Menos_de_1	Menos_de_1	Menos_de_1
NULL	NU	NU	NU	NU	NU	NU	23h00	20	07h30	7_a_	1_ou_2_veze	1_ou_2_veze	3_ou_mais_\
NULL	NU	NU	NU	NU	NU	NU	22_horas	60	6h30	8_hc	1_ou_2_veze	Menos_de_1	Menos_de_1
Nao_sei	3	1	4	0	1	1	21h30	1h	5h30	7	3_ou_mais_\	Menos_de_1	3_ou_mais_\
NULL	NU	NU	NU	NU	NU	NU	23h	20	6h	6	3_ou_mais_\	3_ou_mais_\	Nenhuma_nc

NULL	NU	NU	NU	NU	NU	NU	1h30_da_madrug	20_r	6h10	4_hc	Nenhuma_nc	Nenhuma_nc	Nenhuma_nc
NULL	NU	NU	NU	NU	NU	NU	0h00	60	700	5_a_	3_ou_mais_\	1_ou_2_veze	3_ou_mais_\
Condicoes_d	5	5	3	0	8	1	0h	30-4	8h	6-7h	1_ou_2_veze	3_ou_mais_\	Menos_de_1
NULL	NU	NU	NU	NU	NU	NU	Quase_sempre	10_r	Todos_	8_hc	Nenhuma_nc	Nenhuma_nc	Menos_de_1
NULL	NU	NU	NU	NU	NU	NU	20h00	30	05h00	8	Nenhuma_nc	3_ou_mais_\	Nenhuma_nc
Sedentarism	5	7	9	6	7	5	23h	10_r	6h	de_(	1_ou_2_veze	3_ou_mais_\	3_ou_mais_\
Estresse	6	3	7	8	4	9	Meia_noite	30_r	5h30	4h0(	1_ou_2_veze	3_ou_mais_\	3_ou_mais_\
NULL	NU	NU	NU	NU	NU	NU	23h00	30	5h00	5h3(	Menos_de_1	Nenhuma_nc	Nenhuma_nc
NULL	NU	NU	NU	NU	NU	NU	1h30	20_a	7h15	6__a	Menos_de_1	1_ou_2_veze	Nenhuma_nc
NULL	NU	NU	NU	NU	NU	NU	22h	10m	5h30	6h	Menos_de_1	Menos_de_1	Menos_de_1
NULL	NU	NU	NU	NU	NU	NU	23_h	Deit	06h	De_!	Nenhuma_nc	Nenhuma_nc	Nenhuma_nc
Condicoes_d	5	8	4	2	7	0	5h	20-3	7h	6	3_ou_mais_\	3_ou_mais_\	Menos_de_1
NULL	NU	NU	NU	NU	NU	NU	23_horas	10	07_ho	8	Nenhuma_nc	Menos_de_1	Menos_de_1
Sedentarism	3	3	5	4	7	3	23H00	30M	7H00	6H0(	Menos_de_1	Menos_de_1	1_ou_2_veze
NULL	NU	NU	NU	NU	NU	NU	23h	15	7h	5h	Menos_de_1	3_ou_mais_\	Nenhuma_nc
NULL	NU	NU	NU	NU	NU	NU	Apos_1h_ou_2_h	30_a	Entre_	7h	3_ou_mais_\	1_ou_2_veze	Nenhuma_nc
tombo_de_bi	6	6	8	3	1	5	22h30	30_r	7h	8	1_ou_2_veze	Menos_de_1	1_ou_2_veze
NULL	NU	NU	NU	NU	NU	NU	23h	10m	6h	8	1_ou_2_veze	Menos_de_1	Nenhuma_nc
Preocupacac	3	3	3	3	3	0	Sempre_continue	15_r	7h30	8h	Nenhuma_nc	Menos_de_1	Nenhuma_nc
NULL	NU	NU	NU	NU	NU	NU	10h00	120_	7h00	6_hc	3_ou_mais_\	3_ou_mais_\	3_ou_mais_\
NULL	NU	NU	NU	NU	NU	NU	0h00h	10_r	7h30	5_a_	Menos_de_1	3_ou_mais_\	3_ou_mais_\
NULL	NU	NU	NU	NU	NU	NU	23h	10	7h	7	Nenhuma_nc	3_ou_mais_\	3_ou_mais_\
Condicoes_d	6	9	6	5	7	6	23_horas_	1_hc	5h45_	6_hc	3_ou_mais_\	3_ou_mais_\	Nenhuma_nc
Estresse	8	10	7	6	9	9	23h30	30_r	06h	6-7h	Nenhuma_nc	1_ou_2_veze	Nenhuma_nc
NULL	NU	NU	NU	NU	NU	NU	22	120	7	6	3_ou_mais_\	Nenhuma_nc	Nenhuma_nc
Condicoes_d	7	4	6	4	7	1	Meia_noite	15	6h40	6_h	Menos_de_1	1_ou_2_veze	1_ou_2_veze
NULL	NU	NU	NU	NU	NU	NU	22	15	6	8	Nenhuma_nc	1_ou_2_veze	1_ou_2_veze
Preocupacac	5	1	2	2	3	3	23h30	10_a	06h00	6	Nenhuma_nc	Menos_de_1	Nenhuma_nc
NULL	NU	NU	NU	NU	NU	NU	Sempre_o_mesm	15	Sempr	9h	Nenhuma_nc	Nenhuma_nc	3_ou_mais_\
NULL	NU	NU	NU	NU	NU	NU	1h	5	8	7	Nenhuma_nc	Nenhuma_nc	Nenhuma_nc
NULL	NU	NU	NU	NU	NU	NU	22	5	6	6	Menos_de_1	1_ou_2_veze	3_ou_mais_\
NULL	NU	NU	NU	NU	NU	NU	Meia_noite	2	730	6h	Nenhuma_nc	Nenhuma_nc	Nenhuma_nc
NULL	NU	NU	NU	NU	NU	NU	23	20	7	8	Nenhuma_nc	Nenhuma_nc	Nenhuma_nc
NULL	NU	NU	NU	NU	NU	NU	Meia_noite_	10_r	7h	6_hc	Nenhuma_nc	1_ou_2_veze	Nenhuma_nc
NULL	NU	NU	NU	NU	NU	NU	23	10_r	600	800	Nenhuma_nc	1_ou_2_veze	1_ou_2_veze
Condicoes_d	5	3	6	3	5	0	23_hs	20_r	7_hs	6_hs	Nenhuma_nc	Nenhuma_nc	Nenhuma_nc
NULL	NU	NU	NU	NU	NU	NU	2_da_madrugada	1h	9	5	3_ou_mais_\	Menos_de_1	Nenhuma_nc
NULL	NU	NU	NU	NU	NU	NU	23h50	20	7h00	6	Menos_de_1	3_ou_mais_\	Nenhuma_nc
Preocupacac	5	5	5	0	0	0	0h30	30_r	6h30	6h	Menos_de_1	Menos_de_1	Menos_de_1
NULL	NU	NU	NU	NU	NU	NU	23_horas	50	6	6	3_ou_mais_\	3_ou_mais_\	3_ou_mais_\
NULL	NU	NU	NU	NU	NU	NU	22h00	15_r	7h30	8_hc	Nenhuma_nc	Nenhuma_nc	Nenhuma_nc

_de_Pittsburgh_PSQI

durante_o_u	durante_o_u	durante_o_u	durante_o_u	durante_o_u	durante_o_u	Outra_s_raz	durante_o_u
Nenhuma_nc	Nenhuma_nc	Menos_de_1	Menos_de_1	Menos_de_1	Menos_de_1	NULL	Nenhuma_nc
Nenhuma_nc	Nenhuma_nc	Nenhuma_nc	Nenhuma_nc	Nenhuma_nc	Nenhuma_nc	NULL	Nenhuma_nc
1_ou_2_veze	Nenhuma_nc	Nenhuma_nc	Nenhuma_nc	Menos_de_1	Nenhuma_nc	Ansiedade_e	3_ou_mais_v
1_ou_2_veze	Nenhuma_nc	Nenhuma_nc	3_ou_mais_v	1_ou_2_veze	Nenhuma_nc	NULL	Nenhuma_nc
Nenhuma_nc	Nenhuma_nc	Menos_de_1	Menos_de_1	Menos_de_1	Nenhuma_nc	NULL	Nenhuma_nc
Nenhuma_nc	Nenhuma_nc	Nenhuma_nc	Menos_de_1	1_ou_2_veze	Menos_de_1	NULL	Nenhuma_nc
Nenhuma_nc	Nenhuma_nc	Menos_de_1	1_ou_2_veze	Nenhuma_nc	Nenhuma_nc	Agitacao	3_ou_mais_v
Nenhuma_nc	Nenhuma_nc	Nenhuma_nc	Menos_de_1	Menos_de_1	Nenhuma_nc	NULL	Nenhuma_nc
Nenhuma_nc	Nenhuma_nc	Nenhuma_nc	Menos_de_1	Nenhuma_nc	Menos_de_1	NULL	Nenhuma_nc
3_ou_mais_v	3_ou_mais_v	Nenhuma_nc	1_ou_2_veze	1_ou_2_veze	Menos_de_1	NULL	Nenhuma_nc
Nenhuma_nc	Nenhuma_nc	Nenhuma_nc	Nenhuma_nc	Nenhuma_nc	Nenhuma_nc	NULL	Nenhuma_nc
Nenhuma_nc	Nenhuma_nc	Menos_de_1	Nenhuma_nc	Menos_de_1	Nenhuma_nc	NULL	Nenhuma_nc
Nenhuma_nc	Nenhuma_nc	Nenhuma_nc	Menos_de_1	Menos_de_1	Menos_de_1	Nenhuma	Nenhuma_nc
Menos_de_1	Nenhuma_nc	Nenhuma_nc	1_ou_2_veze	Nenhuma_nc	Menos_de_1	NULL	Nenhuma_nc
3_ou_mais_v	Nenhuma_nc	Nenhuma_nc	Menos_de_1	Menos_de_1	Menos_de_1	NULL	Nenhuma_nc
Nenhuma_nc	Nenhuma_nc	Nenhuma_nc	Menos_de_1	Nenhuma_nc	Nenhuma_nc	NULL	Nenhuma_nc
Nenhuma_nc	Nenhuma_nc	Menos_de_1	Menos_de_1	1_ou_2_veze	Nenhuma_nc	NULL	Nenhuma_nc
Nenhuma_nc	Nenhuma_nc	Nenhuma_nc	Menos_de_1	Nenhuma_nc	Menos_de_1	NULL	Nenhuma_nc
Nenhuma_nc	Nenhuma_nc	1_ou_2_veze	Nenhuma_nc	1_ou_2_veze	1_ou_2_veze	Ansiedade	3_ou_mais_v
Nenhuma_nc	Nenhuma_nc	Nenhuma_nc	Nenhuma_nc	Nenhuma_nc	Nenhuma_nc	NULL	Nenhuma_nc
Nenhuma_nc	Nenhuma_nc	Nenhuma_nc	Nenhuma_nc	Nenhuma_nc	Nenhuma_nc	NULL	Nenhuma_nc
Nenhuma_nc	Nenhuma_nc	Menos_de_1	Nenhuma_nc	1_ou_2_veze	Nenhuma_nc	NULL	Nenhuma_nc
Menos_de_1	Menos_de_1	Menos_de_1	Menos_de_1	Menos_de_1	Menos_de_1	Nao_	Nenhuma_nc
Nenhuma_nc	Nenhuma_nc	Menos_de_1	Nenhuma_nc	Nenhuma_nc	Menos_de_1	NULL	Nenhuma_nc
Nenhuma_nc	Nenhuma_nc	Menos_de_1	Nenhuma_nc	3_ou_mais_v	3_ou_mais_v	Tenho_ansie	3_ou_mais_v
Nenhuma_nc	3_ou_mais_v	Nenhuma_nc	Menos_de_1	Nenhuma_nc	Nenhuma_nc	NULL	Nenhuma_nc
Nenhuma_nc	Nenhuma_nc	1_ou_2_veze	1_ou_2_veze	1_ou_2_veze	Menos_de_1	NULL	Nenhuma_nc
Nenhuma_nc	Nenhuma_nc	Nenhuma_nc	1_ou_2_veze	1_ou_2_veze	Nenhuma_nc	Ansiedade	3_ou_mais_v
Nenhuma_nc	Nenhuma_nc	Nenhuma_nc	1_ou_2_veze	Nenhuma_nc	Nenhuma_nc	NULL	Nenhuma_nc
Nenhuma_nc	Nenhuma_nc	Nenhuma_nc	Nenhuma_nc	Nenhuma_nc	1_ou_2_veze	NULL	Nenhuma_nc
Menos_de_1	Menos_de_1	Nenhuma_nc	Menos_de_1	Menos_de_1	Nenhuma_nc	NULL	Nenhuma_nc
Nenhuma_nc	Nenhuma_nc	Nenhuma_nc	Nenhuma_nc	Nenhuma_nc	Nenhuma_nc	NULL	Nenhuma_nc
Nenhuma_nc	Nenhuma_nc	Nenhuma_nc	Nenhuma_nc	Menos_de_1	Nenhuma_nc	Maioria_das_	Menos_de_1
Nenhuma_nc	Nenhuma_nc	Nenhuma_nc	1_ou_2_veze	Nenhuma_nc	Nenhuma_nc	NULL	Nenhuma_nc
Menos_de_1	Nenhuma_nc	Nenhuma_nc	Nenhuma_nc	Nenhuma_nc	Nenhuma_nc	NULL	Nenhuma_nc
Nenhuma_nc	Nenhuma_nc	Nenhuma_nc	Nenhuma_nc	Menos_de_1	Nenhuma_nc	NULL	Nenhuma_nc
Menos_de_1	3_ou_mais_v	1_ou_2_veze	Menos_de_1	Menos_de_1	1_ou_2_veze	NULL	Nenhuma_nc
Nenhuma_nc	Nenhuma_nc	Nenhuma_nc	Nenhuma_nc	Nenhuma_nc	Nenhuma_nc	NULL	Nenhuma_nc
Nenhuma_nc	Nenhuma_nc	1_ou_2_veze	Nenhuma_nc	Menos_de_1	Nenhuma_nc	Ansiedade	3_ou_mais_v
Nenhuma_nc	Nenhuma_nc	Nenhuma_nc	1_ou_2_veze	Nenhuma_nc	Menos_de_1	NULL	Nenhuma_nc
Nenhuma_nc	Nenhuma_nc	Menos_de_1	Menos_de_1	Nenhuma_nc	Nenhuma_nc	NULL	Nenhuma_nc
Nenhuma_nc	Nenhuma_nc	Menos_de_1	Nenhuma_nc	Nenhuma_nc	Nenhuma_nc	NULL	Nenhuma_nc
Nenhuma_nc	Nenhuma_nc	Menos_de_1	Menos_de_1	Menos_de_1	Menos_de_1	Sofri_um_aci	1_ou_2_veze
Nenhuma_nc	Nenhuma_nc	Menos_de_1	1_ou_2_veze	1_ou_2_veze	1_ou_2_veze	NULL	Nenhuma_nc
Menos_de_1	Nenhuma_nc	Menos_de_1	1_ou_2_veze	Menos_de_1	1_ou_2_veze	NULL	Nenhuma_nc
Nenhuma_nc	Nenhuma_nc	Menos_de_1	Menos_de_1	Nenhuma_nc	1_ou_2_veze	NULL	Nenhuma_nc
Nenhuma_nc	Nenhuma_nc	Nenhuma_nc	Nenhuma_nc	Nenhuma_nc	3_ou_mais_v	NULL	Nenhuma_nc
Nenhuma_nc	Nenhuma_nc	Nenhuma_nc	Menos_de_1	Nenhuma_nc	Menos_de_1	NULL	Nenhuma_nc
Nenhuma_nc	Nenhuma_nc	Menos_de_1	Nenhuma_nc	Nenhuma_nc	Nenhuma_nc	Tenho_dificu	3_ou_mais_v
Nenhuma_nc	Nenhuma_nc	Nenhuma_nc	Nenhuma_nc	Nenhuma_nc	Menos_de_1	NULL	Nenhuma_nc
Menos_de_1	Nenhuma_nc	Nenhuma_nc	Nenhuma_nc	Nenhuma_nc	Nenhuma_nc	Fome	1_ou_2_veze
Menos_de_1	Nenhuma_nc	Nenhuma_nc	Menos_de_1	Menos_de_1	Nenhuma_nc	NULL	Nenhuma_nc
Nenhuma_nc	Nenhuma_nc	Menos_de_1	Nenhuma_nc	1_ou_2_veze	1_ou_2_veze	NULL	Nenhuma_nc
Nenhuma_nc	Nenhuma_nc	Nenhuma_nc	Nenhuma_nc	Menos_de_1	1_ou_2_veze	NULL	Nenhuma_nc
1_ou_2_veze	Menos_de_1	Nenhuma_nc	Menos_de_1	Nenhuma_nc	Menos_de_1	NULL	Nenhuma_nc

3_ou_mais_\	Nenhuma_nc	Nenhuma_nc	1_ou_2_veze	3_ou_mais_\	1_ou_2_vezes_por_semai		Nenhuma_nc
Nenhuma_nc	Nenhuma_nc	Nenhuma_nc	Nenhuma_nc	Menos_de_1	Menos_de_1	Preocupacoe	3_ou_mais_\
3_ou_mais_\	1_ou_2_veze	1_ou_2_veze	Nenhuma_nc	Menos_de_1	Menos_de_1	Nao	Nenhuma_nc
Nenhuma_nc	Nenhuma_nc	Nenhuma_nc	Nenhuma_nc	Nenhuma_nc	Nenhuma_nc	NULL	Nenhuma_nc
Nenhuma_nc	Nenhuma_nc	Nenhuma_nc	Menos_de_1	Nenhuma_nc	Menos_de_1	NULL	Nenhuma_nc
Nenhuma_nc	Nenhuma_nc	Nenhuma_nc	Menos_de_1	Nenhuma_nc	Nenhuma_nc	NULL	Nenhuma_nc
1_ou_2_veze	Nenhuma_nc	Menos_de_1	Menos_de_1	Nenhuma_nc	1_ou_2_veze	NULL	Nenhuma_nc
Menos_de_1	Nenhuma_nc	Nenhuma_nc	Nenhuma_nc	1_ou_2_veze	Nenhuma_nc	NULL	Nenhuma_nc
Nenhuma_nc	Nenhuma_nc	1_ou_2_veze	Menos_de_1	Menos_de_1	Menos_de_1	Nao_conseg	3_ou_mais_\
Nenhuma_nc	Menos_de_1	Nenhuma_nc	Nenhuma_nc	Nenhuma_nc	Menos_de_1	NULL	Nenhuma_nc
Nenhuma_nc	Nenhuma_nc	Menos_de_1	Nenhuma_nc	Menos_de_1	Nenhuma_nc	NULL	Nenhuma_nc
Nenhuma_nc	Menos_de_1	Nenhuma_nc	Menos_de_1	Menos_de_1	Menos_de_1	Sinto_que_e	3_ou_mais_\
Nenhuma_nc	Nenhuma_nc	Nenhuma_nc	Nenhuma_nc	Nenhuma_nc	Nenhuma_nc	Preocupacac	1_ou_2_veze
Nenhuma_nc	Nenhuma_nc	3_ou_mais_\	Nenhuma_nc	Menos_de_1	Menos_de_1	NULL	Nenhuma_nc
Nenhuma_nc	Nenhuma_nc	Nenhuma_nc	Nenhuma_nc	Nenhuma_nc	Nenhuma_nc	Nao_tive_ne	Nenhuma_nc
Nenhuma_nc	Nenhuma_nc	Nenhuma_nc	Nenhuma_nc	Nenhuma_nc	Nenhuma_nc	NULL	Nenhuma_nc
Nenhuma_nc	Nenhuma_nc	Nenhuma_nc	Menos_de_1	Nenhuma_nc	Nenhuma_nc	NULL	Nenhuma_nc
Nenhuma_nc	Nenhuma_nc	Nenhuma_nc	3_ou_mais_\	Menos_de_1	Nenhuma_nc	NULL	Nenhuma_nc
1_ou_2_veze	1_ou_2_veze	Nenhuma_nc	3_ou_mais_\	Nenhuma_nc	Menos_de_1	NULL	Nenhuma_nc
1_ou_2_veze	Nenhuma_nc	Nenhuma_nc	Menos_de_1	Menos_de_1	Nenhuma_nc	Ansiedade-_	1_ou_2_veze
Nenhuma_nc	Nenhuma_nc	Nenhuma_nc	Nenhuma_nc	Menos_de_1	Nenhuma_nc	Ansiedade_	1_ou_2_veze
Nenhuma_nc	Menos_de_1	Nenhuma_nc	Nenhuma_nc	Nenhuma_nc	Menos_de_1	NULL	Nenhuma_nc
Nenhuma_nc	Nenhuma_nc	Nenhuma_nc	1_ou_2_veze	Nenhuma_nc	Nenhuma_nc	Barulhos_na	3_ou_mais_\
Nenhuma_nc	Nenhuma_nc	Menos_de_1	Menos_de_1	Nenhuma_nc	Nenhuma_nc	NULL	Nenhuma_nc
Menos_de_1	Menos_de_1	Menos_de_1	Menos_de_1	3_ou_mais_\	1_ou_2_veze	Ver_muitas_	1_ou_2_veze
Nenhuma_nc	Nenhuma_nc	Menos_de_1	Menos_de_1	Nenhuma_nc	1_ou_2_veze	NULL	Nenhuma_nc
Nenhuma_nc	Nenhuma_nc	Nenhuma_nc	Nenhuma_nc	Nenhuma_nc	Nenhuma_nc	NULL	Nenhuma_nc
Nenhuma_nc	Nenhuma_nc	Nenhuma_nc	Menos_de_1	Menos_de_1	Nenhuma_nc	NULL	Nenhuma_nc
Nenhuma_nc	Nenhuma_nc	Nenhuma_nc	Nenhuma_nc	Menos_de_1	Nenhuma_nc	NULL	Nenhuma_nc
Nenhuma_nc	Nenhuma_nc	Nenhuma_nc	Nenhuma_nc	Nenhuma_nc	Nenhuma_nc	NULL	Nenhuma_nc
Nenhuma_nc	Menos_de_1	Menos_de_1	Nenhuma_nc	1_ou_2_veze	Nenhuma_nc	NULL	Nenhuma_nc
Nenhuma_nc	Nenhuma_nc	Nenhuma_nc	Nenhuma_nc	Nenhuma_nc	Nenhuma_nc	NULL	Nenhuma_nc
Nenhuma_nc	Nenhuma_nc	Nenhuma_nc	1_ou_2_veze	Menos_de_1	Nenhuma_nc	NULL	Nenhuma_nc
Nenhuma_nc	Nenhuma_nc	Menos_de_1	1_ou_2_veze	Menos_de_1	Menos_de_1	NULL	Nenhuma_nc
Nenhuma_nc	Nenhuma_nc	Nenhuma_nc	1_ou_2_veze	Menos_de_1	Nenhuma_nc	NULL	Nenhuma_nc
Menos_de_1	Nenhuma_nc	Nenhuma_nc	Nenhuma_nc	Nenhuma_nc	Nenhuma_nc	NULL	Nenhuma_nc
Nenhuma_nc	Nenhuma_nc	Nenhuma_nc	3_ou_mais_\	Menos_de_1	Menos_de_1	NULL	Nenhuma_nc
Nenhuma_nc	Nenhuma_nc	Menos_de_1	3_ou_mais_\	1_ou_2_veze	Menos_de_1	NULL	Nenhuma_nc
1_ou_2_veze	Nenhuma_nc	Menos_de_1	1_ou_2_veze	3_ou_mais_\	1_ou_2_veze	NULL	Nenhuma_nc
Nenhuma_nc	Nenhuma_nc	Nenhuma_nc	Nenhuma_nc	Nenhuma_nc	Nenhuma_nc	NULL	Nenhuma_nc
Nenhuma_nc	Nenhuma_nc	1_ou_2_veze	Menos_de_1	1_ou_2_veze	Menos_de_1	NULL	Nenhuma_nc
Menos_de_1	Nenhuma_nc	1_ou_2_veze	Menos_de_1	1_ou_2_veze	3_ou_mais_\	NULL	Nenhuma_nc
Nenhuma_nc	Nenhuma_nc	Nenhuma_nc	1_ou_2_veze	Nenhuma_nc	1_ou_2_veze	NULL	Nenhuma_nc
Nenhuma_nc	Nenhuma_nc	Nenhuma_nc	Nenhuma_nc	3_ou_mais_\	Nenhuma_nc	NULL	Nenhuma_nc
Menos_de_1	Menos_de_1	1_ou_2_veze	Menos_de_1	Nenhuma_nc	Nenhuma_nc	NULL	Nenhuma_nc
Menos_de_1	Nenhuma_nc	Nenhuma_nc	Menos_de_1	Menos_de_1	1_ou_2_veze	NULL	Nenhuma_nc
Menos_de_1	1_ou_2_veze	Nenhuma_nc	Menos_de_1	Menos_de_1	Nenhuma_nc	NULL	Nenhuma_nc
Nenhuma_nc	Nenhuma_nc	Menos_de_1	Menos_de_1	Nenhuma_nc	Nenhuma_nc	NULL	Nenhuma_nc
Nenhuma_nc	Nenhuma_nc	Nenhuma_nc	Nenhuma_nc	1_ou_2_veze	Nenhuma_nc	NULL	Nenhuma_nc
1_ou_2_veze	Nenhuma_nc	Nenhuma_nc	Nenhuma_nc	Nenhuma_nc	Nenhuma_nc	NULL	Nenhuma_nc
Nenhuma_nc	Nenhuma_nc	Menos_de_1	Menos_de_1	Menos_de_1	Nenhuma_nc	NULL	Nenhuma_nc
Nenhuma_nc	Nenhuma_nc	3_ou_mais_\	Nenhuma_nc	3_ou_mais_\	Menos_de_1	NULL	Menos_de_1
Nenhuma_nc	Nenhuma_nc	Nenhuma_nc	1_ou_2_veze	3_ou_mais_\	Menos_de_1	Dificuldade_e	3_ou_mais_\
Nenhuma_nc	Nenhuma_nc	Nenhuma_nc	Nenhuma_nc	Menos_de_1	Nenhuma_nc	Ansiedade_n	1_ou_2_veze
Nenhuma_nc	Nenhuma_nc	Nenhuma_nc	1_ou_2_veze	3_ou_mais_\	Nenhuma_nc	NULL	Nenhuma_nc
1_ou_2_veze	3_ou_mais_\	Nenhuma_nc	Nenhuma_nc	Nenhuma_nc	Nenhuma_nc	Stress	3_ou_mais_\
1_ou_2_veze	Nenhuma_nc	Nenhuma_nc	1_ou_2_veze	Nenhuma_nc	1_ou_2_veze	NULL	Nenhuma_nc
Nenhuma_nc	Nenhuma_nc	Nenhuma_nc	Nenhuma_nc	Nenhuma_nc	Nenhuma_nc	NULL	Nenhuma_nc

Nenhuma_nc	Nenhuma_nc	Nenhuma_nc	3_ou_mais_\	Menos_de_1	Nenhuma_nc	NULL	Nenhuma_nc
Nenhuma_nc	Nenhuma_nc	Menos_de_1	1_ou_2_veze	Menos_de_1	Menos_de_1	NULL	Nenhuma_nc
1_ou_2_veze	Nenhuma_nc	Menos_de_1	1_ou_2_veze	Nenhuma_nc	Nenhuma_nc	Tempo_seco	1_ou_2_veze
Menos_de_1	Nenhuma_nc	Nenhuma_nc	Menos_de_1	1_ou_2_veze	Nenhuma_nc	NULL	Nenhuma_nc
Nenhuma_nc	Nenhuma_nc	Nenhuma_nc	Nenhuma_nc	Nenhuma_nc	Nenhuma_nc	NULL	Nenhuma_nc
Nenhuma_nc	Nenhuma_nc	Nenhuma_nc	Nenhuma_nc	Nenhuma_nc	Nenhuma_nc	NULL	Nenhuma_nc
Nenhuma_nc	Nenhuma_nc	Nenhuma_nc	Menos_de_1	Nenhuma_nc	Nenhuma_nc	NULL	Nenhuma_nc
Nenhuma_nc	Nenhuma_nc	Nenhuma_nc	Nenhuma_nc	1_ou_2_veze	Menos_de_1	NULL	Nenhuma_nc
Nenhuma_nc	Nenhuma_nc	Nenhuma_nc	Nenhuma_nc	Menos_de_1	Nenhuma_nc	Nao	Nenhuma_nc
Nenhuma_nc	Nenhuma_nc	Nenhuma_nc	Menos_de_1	Nenhuma_nc	Nenhuma_nc	NULL	Nenhuma_nc
Nenhuma_nc	Nenhuma_nc	Menos_de_1	Nenhuma_nc	Nenhuma_nc	Menos_de_1	NULL	Nenhuma_nc
Nenhuma_nc	Nenhuma_nc	Menos_de_1	Nenhuma_nc	Nenhuma_nc	Nenhuma_nc	NULL	Nenhuma_nc
Nenhuma_nc	Nenhuma_nc	Nenhuma_nc	1_ou_2_veze	Menos_de_1	Menos_de_1	Ansiedade_-	3_ou_mais_\
Nenhuma_nc	Menos_de_1	Nenhuma_nc	Nenhuma_nc	Nenhuma_nc	Nenhuma_nc	NULL	Nenhuma_nc
Nenhuma_nc	Nenhuma_nc	Nenhuma_nc	Menos_de_1	Nenhuma_nc	Nenhuma_nc	Se_durmo_a	Menos_de_1
Nenhuma_nc	Nenhuma_nc	Menos_de_1	Nenhuma_nc	Nenhuma_nc	Nenhuma_nc	A_falta_de_u	3_ou_mais_\
Nenhuma_nc	3_ou_mais_\	Nenhuma_nc	Nenhuma_nc	Nenhuma_nc	Nenhuma_nc	NULL	Nenhuma_nc
Nenhuma_nc	Nenhuma_nc	Nenhuma_nc	Menos_de_1	Nenhuma_nc	1_ou_2_veze	NULL	1_ou_2_veze
Nenhuma_nc	Nenhuma_nc	Nenhuma_nc	1_ou_2_veze	Nenhuma_nc	Menos_de_1	NULL	Nenhuma_nc
3_ou_mais_\	3_ou_mais_\	Menos_de_1	Nenhuma_nc	3_ou_mais_\	Menos_de_1	NULL	Nenhuma_nc
Nenhuma_nc	Nenhuma_nc	Nenhuma_nc	1_ou_2_veze	Nenhuma_nc	Nenhuma_nc	NULL	Nenhuma_nc
Nenhuma_nc	Nenhuma_nc	Nenhuma_nc	Nenhuma_nc	Menos_de_1	Nenhuma_nc	Fui_ao_banh	Nenhuma_nc
Nenhuma_nc	Nenhuma_nc	Menos_de_1	Nenhuma_nc	Nenhuma_nc	1_ou_2_veze	NULL	Nenhuma_nc
Nenhuma_nc	Nenhuma_nc	Menos_de_1	Nenhuma_nc	Nenhuma_nc	Nenhuma_nc	NULL	Nenhuma_nc
Nenhuma_nc	Nenhuma_nc	1_ou_2_veze	1_ou_2_veze	Menos_de_1	1_ou_2_veze	NULL	Nenhuma_nc
1_ou_2_veze	Nenhuma_nc	Nenhuma_nc	3_ou_mais_\	1_ou_2_veze	1_ou_2_veze	NULL	Nenhuma_nc
3_ou_mais_\	Nenhuma_nc	Nenhuma_nc	Menos_de_1	Nenhuma_nc	3_ou_mais_\	Gatos_kkkk	3_ou_mais_\
Menos_de_1	Menos_de_1	Nenhuma_nc	3_ou_mais_\	Nenhuma_nc	Nenhuma_nc	NULL	Nenhuma_nc
Nenhuma_nc	Nenhuma_nc	Nenhuma_nc	Nenhuma_nc	Nenhuma_nc	Nenhuma_nc	Nenhuma	Nenhuma_nc
Menos_de_1	Nenhuma_nc	Nenhuma_nc	Nenhuma_nc	Nenhuma_nc	Nenhuma_nc	NULL	Nenhuma_nc
1_ou_2_veze	Nenhuma_nc	Nenhuma_nc	1_ou_2_veze	Menos_de_1	1_ou_2_veze	NULL	Nenhuma_nc
Nenhuma_nc	Nenhuma_nc	Nenhuma_nc	Menos_de_1	Menos_de_1	Menos_de_1	NULL	Nenhuma_nc
Menos_de_1	Nenhuma_nc	Nenhuma_nc	Nenhuma_nc	Nenhuma_nc	Nenhuma_nc	NULL	Nenhuma_nc
Nenhuma_nc	Nenhuma_nc	Nenhuma_nc	Nenhuma_nc	Nenhuma_nc	Menos_de_1	NULL	Nenhuma_nc
Nenhuma_nc	Nenhuma_nc	Nenhuma_nc	Nenhuma_nc	Nenhuma_nc	Nenhuma_nc	NULL	Nenhuma_nc
Nenhuma_nc	Nenhuma_nc	Nenhuma_nc	Nenhuma_nc	Nenhuma_nc	Nenhuma_nc	NULL	Nenhuma_nc
Nenhuma_nc	Nenhuma_nc	Nenhuma_nc	Nenhuma_nc	Nenhuma_nc	Nenhuma_nc	NULL	Nenhuma_nc
Nenhuma_nc	Menos_de_1	3_ou_mais_\	Nenhuma_nc	1_ou_2_veze	Menos_de_1	NULL	Nenhuma_nc
Nenhuma_nc	Nenhuma_nc	Nenhuma_nc	Nenhuma_nc	Nenhuma_nc	Nenhuma_nc	NULL	Nenhuma_nc
Nenhuma_nc	Nenhuma_nc	Nenhuma_nc	Nenhuma_nc	Menos_de_1	Nenhuma_nc	Excesso_de_	1_ou_2_veze
Nenhuma_nc	Nenhuma_nc	Nenhuma_nc	Nenhuma_nc	Nenhuma_nc	Nenhuma_nc	Ansiedade	Menos_de_1
Nenhuma_nc	Nenhuma_nc	Menos_de_1	Nenhuma_nc	1_ou_2_veze	Menos_de_1	NULL	Nenhuma_nc
Nenhuma_nc	Nenhuma_nc	Nenhuma_nc	Nenhuma_nc	Menos_de_1	Nenhuma_nc	NULL	Nenhuma_nc
Nenhuma_nc	Nenhuma_nc	Nenhuma_nc	Nenhuma_nc	Nenhuma_nc	Nenhuma_nc	NULL	Nenhuma_nc
Menos_de_1	1_ou_2_veze	Nenhuma_nc	Nenhuma_nc	Menos_de_1	Nenhuma_nc	NULL	Nenhuma_nc
Nenhuma_nc	Nenhuma_nc	Menos_de_1	Menos_de_1	Nenhuma_nc	Menos_de_1	NULL	Nenhuma_nc
Menos_de_1	Nenhuma_nc	Nenhuma_nc	3_ou_mais_\	Nenhuma_nc	Nenhuma_nc	NULL	Nenhuma_nc
Nenhuma_nc	Nenhuma_nc	Nenhuma_nc	Menos_de_1	Nenhuma_nc	1_ou_2_veze	Cansaco_me	3_ou_mais_\
Nenhuma_nc	Nenhuma_nc	Nenhuma_nc	Menos_de_1	Menos_de_1	Menos_de_1	NULL	Nenhuma_nc
Nenhuma_nc	Nenhuma_nc	Nenhuma_nc	Menos_de_1	Nenhuma_nc	Menos_de_1	ansiedade	1_ou_2_veze
1_ou_2_veze	Nenhuma_nc	3_ou_mais_\	Nenhuma_nc	Nenhuma_nc	Menos_de_1	NULL	Nenhuma_nc
Nenhuma_nc	Nenhuma_nc	Nenhuma_nc	Nenhuma_nc	Nenhuma_nc	Nenhuma_nc	NULL	Nenhuma_nc
Nenhuma_nc	Nenhuma_nc	Menos_de_1	Menos_de_1	Nenhuma_nc	Nenhuma_nc	Excesso_de_	Menos_de_1
Nenhuma_nc	Nenhuma_nc	Nenhuma_nc	Nenhuma_nc	Nenhuma_nc	Nenhuma_nc	Filho_com_ic	3_ou_mais_\
Nenhuma_nc	Nenhuma_nc	1_ou_2_veze	Menos_de_1	1_ou_2_veze	Menos_de_1	Ansiedade_e	1_ou_2_veze
Menos_de_1	Nenhuma_nc	Nenhuma_nc	3_ou_mais_\	1_ou_2_veze	Menos_de_1	Nao_	Nenhuma_nc
Nenhuma_nc	Nenhuma_nc	Menos_de_1	Nenhuma_nc	Menos_de_1	Nenhuma_nc	NULL	Nenhuma_nc
Nenhuma_nc	Menos_de_1	Nenhuma_nc	Menos_de_1	Nenhuma_nc	Nenhuma_nc	NULL	Nenhuma_nc

Nenhuma_nc	1_ou_2_veze	Nenhuma_nc	Menos_de_1	Nenhuma_nc	Menos_de_1	NULL	Nenhuma_nc
Nenhuma_nc	Nenhuma_nc	Nenhuma_nc	Menos_de_1	Menos_de_1	Nenhuma_nc	Barulho_de_	1_ou_2_veze
Nenhuma_nc	Menos_de_1	Menos_de_1	1_ou_2_veze	1_ou_2_veze	Menos_de_1	Gatos_da_ca	1_ou_2_veze
Nenhuma_nc	Nenhuma_nc	Nenhuma_nc	Menos_de_1	Nenhuma_nc	Nenhuma_nc	Exercicio_de	Nenhuma_nc
Nenhuma_nc	Nenhuma_nc	Nenhuma_nc	Nenhuma_nc	Nenhuma_nc	Nenhuma_nc	NULL	Nenhuma_nc
3_ou_mais_\	3_ou_mais_\	Menos_de_1	Nenhuma_nc	Nenhuma_nc	1_ou_2_veze	NULL	3_ou_mais_\
Nenhuma_nc	Nenhuma_nc	Nenhuma_nc	Nenhuma_nc	Nenhuma_nc	Nenhuma_nc	Ja_tive_dificu	Nenhuma_nc
Nenhuma_nc	Nenhuma_nc	Nenhuma_nc	Menos_de_1	Menos_de_1	Nenhuma_nc	NULL	Nenhuma_nc
Nenhuma_nc	Nenhuma_nc	Nenhuma_nc	Nenhuma_nc	Nenhuma_nc	1_ou_2_veze	Pensamento_	1_ou_2_veze
Menos_de_1	Nenhuma_nc	Nenhuma_nc	Nenhuma_nc	1_ou_2_veze	1_ou_2_veze	Ansiedade	3_ou_mais_\
Nenhuma_nc	Nenhuma_nc	Nenhuma_nc	Nenhuma_nc	3_ou_mais_\	1_ou_2_veze	Ansiedade	3_ou_mais_\
1_ou_2_veze	Nenhuma_nc	Nenhuma_nc	Nenhuma_nc	Nenhuma_nc	Nenhuma_nc	NULL	Nenhuma_nc
Nenhuma_nc	Nenhuma_nc	Nenhuma_nc	Menos_de_1	Menos_de_1	Nenhuma_nc	NULL	Nenhuma_nc
Menos_de_1	Nenhuma_nc	Nenhuma_nc	Nenhuma_nc	Nenhuma_nc	Nenhuma_nc	NULL	Nenhuma_nc
Nenhuma_nc	Nenhuma_nc	Nenhuma_nc	Nenhuma_nc	Menos_de_1	Nenhuma_nc	NULL	Nenhuma_nc
3_ou_mais_\	1_ou_2_veze	Nenhuma_nc	Nenhuma_nc	Menos_de_1	1_ou_2_veze	NULL	Menos_de_1
Nenhuma_nc	Nenhuma_nc	Menos_de_1	Menos_de_1	Nenhuma_nc	1_ou_2_veze	NULL	Nenhuma_nc
Nenhuma_nc	Nenhuma_nc	Nenhuma_nc	Nenhuma_nc	Menos_de_1	Nenhuma_nc	NULL	Nenhuma_nc
Nenhuma_nc	Nenhuma_nc	Nenhuma_nc	Nenhuma_nc	1_ou_2_veze	1_ou_2_veze	Criancas_ch	3_ou_mais_\
Menos_de_1	Nenhuma_nc	Nenhuma_nc	1_ou_2_veze	Nenhuma_nc	Nenhuma_nc	NULL	Nenhuma_nc
Nenhuma_nc	Nenhuma_nc	Nenhuma_nc	Nenhuma_nc	Nenhuma_nc	Menos_de_1	NULL	Nenhuma_nc
Nenhuma_nc	3_ou_mais_\	Menos_de_1	Nenhuma_nc	Nenhuma_nc	Menos_de_1	NULL	Nenhuma_nc
Menos_de_1	Nenhuma_nc	Nenhuma_nc	Menos_de_1	3_ou_mais_\	Nenhuma_nc	Tive_um_ter	3_ou_mais_\
Nenhuma_nc	Nenhuma_nc	Nenhuma_nc	Nenhuma_nc	Nenhuma_nc	Nenhuma_nc	NULL	Nenhuma_nc
Nenhuma_nc	Nenhuma_nc	Nenhuma_nc	Nenhuma_nc	Menos_de_1	Nenhuma_nc	Nenhuma	Nenhuma_nc
Nenhuma_nc	Nenhuma_nc	Menos_de_1	1_ou_2_veze	Menos_de_1	Menos_de_1	Preocupacoe	1_ou_2_veze
Nenhuma_nc	Nenhuma_nc	Nenhuma_nc	Nenhuma_nc	1_ou_2_veze	Menos_de_1	NULL	Nenhuma_nc
Nenhuma_nc	Nenhuma_nc	Nenhuma_nc	Nenhuma_nc	Nenhuma_nc	Nenhuma_nc	NULL	1_ou_2_veze
3_ou_mais_\	3_ou_mais_\	Nenhuma_nc	3_ou_mais_\	Menos_de_1	1_ou_2_veze	NULL	Nenhuma_nc
1_ou_2_veze	Menos_de_1	Menos_de_1	1_ou_2_veze	Nenhuma_nc	3_ou_mais_\	NULL	Nenhuma_nc
1_ou_2_veze	Nenhuma_nc	Nenhuma_nc	Nenhuma_nc	Menos_de_1	Menos_de_1	moro_numa_	3_ou_mais_\
Nenhuma_nc	Nenhuma_nc	Nenhuma_nc	Menos_de_1	Nenhuma_nc	Nenhuma_nc	NULL	Nenhuma_nc
Nenhuma_nc	Nenhuma_nc	Nenhuma_nc	1_ou_2_veze	3_ou_mais_\	Menos_de_1	NULL	Nenhuma_nc
Nenhuma_nc	Nenhuma_nc	Nenhuma_nc	Menos_de_1	Nenhuma_nc	Nenhuma_nc	NULL	Nenhuma_nc
Nenhuma_nc	Nenhuma_nc	Nenhuma_nc	Nenhuma_nc	Nenhuma_nc	1_ou_2_veze	NULL	Nenhuma_nc
Nenhuma_nc	Nenhuma_nc	Nenhuma_nc	Menos_de_1	Nenhuma_nc	Menos_de_1	Por_nao_cor	3_ou_mais_\
Nenhuma_nc	Nenhuma_nc	Menos_de_1	Nenhuma_nc	Menos_de_1	3_ou_mais_\	Insonia-_estr	3_ou_mais_\
Nenhuma_nc	Nenhuma_nc	Nenhuma_nc	Menos_de_1	Nenhuma_nc	Nenhuma_nc	NULL	Nenhuma_nc
Nenhuma_nc	Menos_de_1	Nenhuma_nc	Menos_de_1	1_ou_2_veze	1_ou_2_veze	Nao_	Nenhuma_nc
Nenhuma_nc	Nenhuma_nc	Nenhuma_nc	Nenhuma_nc	1_ou_2_veze	Nenhuma_nc	NULL	Nenhuma_nc
Nenhuma_nc	Menos_de_1	Nenhuma_nc	1_ou_2_veze	Nenhuma_nc	Nenhuma_nc	NULL	Nenhuma_nc
Menos_de_1	Nenhuma_nc	Menos_de_1	Nenhuma_nc	Menos_de_1	Menos_de_1	NULL	Nenhuma_nc
Nenhuma_nc	Nenhuma_nc	Nenhuma_nc	Menos_de_1	Nenhuma_nc	Nenhuma_nc	NULL	Nenhuma_nc
Nenhuma_nc	Nenhuma_nc	Nenhuma_nc	Menos_de_1	Menos_de_1	Nenhuma_nc	NULL	Nenhuma_nc
Nenhuma_nc	Nenhuma_nc	Nenhuma_nc	Nenhuma_nc	Nenhuma_nc	Nenhuma_nc	NULL	Nenhuma_nc
Nenhuma_nc	Nenhuma_nc	Nenhuma_nc	Nenhuma_nc	3_ou_mais_\	1_ou_2_veze	Poluicao_sor	1_ou_2_veze
Nenhuma_nc	Nenhuma_nc	3_ou_mais_\	Menos_de_1	Menos_de_1	Menos_de_1	NULL	Nenhuma_nc
Nenhuma_nc	Menos_de_1	Menos_de_1	Menos_de_1	Menos_de_1	Nenhuma_nc	Dor_no_nerv	Menos_de_1
Nenhuma_nc	Nenhuma_nc	Nenhuma_nc	Nenhuma_nc	Nenhuma_nc	Nenhuma_nc	NULL	Nenhuma_nc
Nenhuma_nc	Nenhuma_nc	Nenhuma_nc	Nenhuma_nc	Nenhuma_nc	Menos_de_1	NULL	Nenhuma_nc
1_ou_2_veze	Menos_de_1	Nenhuma_nc	Menos_de_1	Nenhuma_nc	1_ou_2_veze	NULL	Nenhuma_nc
3_ou_mais_\	Nenhuma_nc	1_ou_2_veze	Nenhuma_nc	Nenhuma_nc	1_ou_2_veze	A_rotina_de_	3_ou_mais_\
Nenhuma_nc	Nenhuma_nc	Nenhuma_nc	1_ou_2_veze	3_ou_mais_\	3_ou_mais_\	Perder_o_so	3_ou_mais_\
Nenhuma_nc	Nenhuma_nc	Menos_de_1	1_ou_2_veze	Nenhuma_nc	1_ou_2_veze	Amamentaca	3_ou_mais_\
Menos_de_1	Nenhuma_nc	Nenhuma_nc	Nenhuma_nc	Menos_de_1	Nenhuma_nc	NULL	Nenhuma_nc
Nenhuma_nc	Nenhuma_nc	Menos_de_1	Nenhuma_nc	Menos_de_1	Nenhuma_nc	Preocupacoe	Menos_de_1
Nenhuma_nc	Nenhuma_nc	Nenhuma_nc	1_ou_2_veze	Nenhuma_nc	Nenhuma_nc	Preocupacac	1_ou_2_veze
Menos_de_1	Nenhuma_nc	Nenhuma_nc	Nenhuma_nc	Menos_de_1	Menos_de_1	NULL	Nenhuma_nc

Nenhuma_nc	Nenhuma_nc	Nenhuma_nc	3_ou_mais_v	3_ou_mais_v	1_ou_2_veze	NULL	Nenhuma_nc
Menos_de_1	Nenhuma_nc	Nenhuma_nc	Menos_de_1	Nenhuma_nc	Menos_de_1	Minha_ansie	1_ou_2_veze
Nenhuma_nc	Nenhuma_nc	Menos_de_1	1_ou_2_veze	Menos_de_1	1_ou_2_veze	NULL	1_ou_2_veze
1_ou_2_veze	Menos_de_1	Nenhuma_nc	1_ou_2_veze	Nenhuma_nc	Menos_de_1	Sintomas_rel	1_ou_2_veze
Nenhuma_nc	Nenhuma_nc	Nenhuma_nc	Nenhuma_nc	Menos_de_1	1_ou_2_veze	torci_o_tornc	Menos_de_1
Nenhuma_nc	Nenhuma_nc	Nenhuma_nc	Nenhuma_nc	Nenhuma_nc	3_ou_mais_v	NULL	Nenhuma_nc
Nenhuma_nc	Nenhuma_nc	Nenhuma_nc	Nenhuma_nc	Nenhuma_nc	1_ou_2_veze	NULL	Nenhuma_nc
Nenhuma_nc	Nenhuma_nc	Nenhuma_nc	Nenhuma_nc	Menos_de_1	Nenhuma_nc	NULL	Nenhuma_nc
Nenhuma_nc	Nenhuma_nc	Nenhuma_nc	Nenhuma_nc	Nenhuma_nc	Menos_de_1	Tenho_dificu	Menos_de_1
Menos_de_1	Nenhuma_nc	Menos_de_1	Nenhuma_nc	Nenhuma_nc	Nenhuma_nc	NULL	Nenhuma_nc
Nenhuma_nc	Nenhuma_nc	Nenhuma_nc	Menos_de_1	Nenhuma_nc	Menos_de_1	NULL	Nenhuma_nc
Nenhuma_nc	Nenhuma_nc	Menos_de_1	Menos_de_1	1_ou_2_veze	3_ou_mais_v	NULL	Nenhuma_nc
Nenhuma_nc	Nenhuma_nc	Nenhuma_nc	Nenhuma_nc	Menos_de_1	Menos_de_1	NULL	Nenhuma_nc
Nenhuma_nc	Nenhuma_nc	Nenhuma_nc	Nenhuma_nc	Nenhuma_nc	Nenhuma_nc	nada	Nenhuma_nc
Nenhuma_nc	Nenhuma_nc	Nenhuma_nc	1_ou_2_veze	Menos_de_1	Menos_de_1	NULL	Nenhuma_nc
Menos_de_1	Nenhuma_nc	Menos_de_1	Menos_de_1	Menos_de_1	Menos_de_1	Filho_acorda	3_ou_mais_v
Nenhuma_nc	Nenhuma_nc	Menos_de_1	3_ou_mais_v	Nenhuma_nc	Menos_de_1	NULL	Nenhuma_nc
Menos_de_1	Menos_de_1	Nenhuma_nc	Menos_de_1	Menos_de_1	Menos_de_1	NULL	Nenhuma_nc
Nenhuma_nc	1_ou_2_veze	Nenhuma_nc	Nenhuma_nc	Nenhuma_nc	Nenhuma_nc	nenhuma_ra:	Nenhuma_nc
Menos_de_1	Nenhuma_nc	Nenhuma_nc	Menos_de_1	Menos_de_1	Menos_de_1	NULL	Nenhuma_nc
Menos_de_1	Nenhuma_nc	Nenhuma_nc	Menos_de_1	Menos_de_1	1_ou_2_veze	NULL	Nenhuma_nc
Nenhuma_nc	Nenhuma_nc	Nenhuma_nc	Nenhuma_nc	Nenhuma_nc	Nenhuma_nc	NULL	Nenhuma_nc
Nenhuma_nc	1_ou_2_veze	3_ou_mais_v	1_ou_2_veze	Nenhuma_nc	Nenhuma_nc	NULL	Nenhuma_nc
Menos_de_1	Menos_de_1	3_ou_mais_v	Menos_de_1	3_ou_mais_v	1_ou_2_veze	Preocupacoe	3_ou_mais_v
1_ou_2_veze	Nenhuma_nc	Nenhuma_nc	1_ou_2_veze	Menos_de_1	Menos_de_1	NULL	Nenhuma_nc
Nenhuma_nc	Nenhuma_nc	Nenhuma_nc	Menos_de_1	Menos_de_1	Nenhuma_nc	NULL	Nenhuma_nc
Nenhuma_nc	Nenhuma_nc	Nenhuma_nc	1_ou_2_veze	Menos_de_1	Nenhuma_nc	NULL	Nenhuma_nc
Nenhuma_nc	Nenhuma_nc	Nenhuma_nc	Nenhuma_nc	Menos_de_1	Nenhuma_nc	NULL	Nenhuma_nc
Nenhuma_nc	Nenhuma_nc	Nenhuma_nc	Nenhuma_nc	Menos_de_1	3_ou_mais_v	NULL	Nenhuma_nc
3_ou_mais_v	3_ou_mais_v	Nenhuma_nc	Nenhuma_nc	Nenhuma_nc	Menos_de_1	NULL	Nenhuma_nc
Nenhuma_nc	Nenhuma_nc	Nenhuma_nc	3_ou_mais_v	Nenhuma_nc	Nenhuma_nc	NULL	Nenhuma_nc
1_ou_2_veze	Nenhuma_nc	Menos_de_1	1_ou_2_veze	1_ou_2_veze	Nenhuma_nc	NULL	Nenhuma_nc
Menos_de_1	Nenhuma_nc	Nenhuma_nc	Nenhuma_nc	Menos_de_1	3_ou_mais_v	Nunca_tive_(	3_ou_mais_v
Nenhuma_nc	Nenhuma_nc	Nenhuma_nc	Menos_de_1	Menos_de_1	3_ou_mais_v	NULL	Nenhuma_nc
Nenhuma_nc	Nenhuma_nc	Nenhuma_nc	Nenhuma_nc	Nenhuma_nc	Nenhuma_nc	NULL	Nenhuma_nc
Nenhuma_nc	Nenhuma_nc	Menos_de_1	Nenhuma_nc	Nenhuma_nc	Menos_de_1	NULL	Nenhuma_nc
Nenhuma_nc	Nenhuma_nc	Menos_de_1	Nenhuma_nc	Menos_de_1	Menos_de_1	barulho_no_v	Menos_de_1
1_ou_2_veze	Menos_de_1	Nenhuma_nc	3_ou_mais_v	Nenhuma_nc	3_ou_mais_v	NULL	Nenhuma_nc
Nenhuma_nc	Nenhuma_nc	Nenhuma_nc	1_ou_2_veze	Menos_de_1	1_ou_2_veze	Cansaco_me	3_ou_mais_v
Nenhuma_nc	Nenhuma_nc	Nenhuma_nc	Nenhuma_nc	Nenhuma_nc	Nenhuma_nc	NULL	Nenhuma_nc
Menos_de_1	Menos_de_1	Nenhuma_nc	Menos_de_1	Nenhuma_nc	Nenhuma_nc	NULL	Nenhuma_nc
Nenhuma_nc	Nenhuma_nc	Menos_de_1	Menos_de_1	Nenhuma_nc	Nenhuma_nc	NULL	Nenhuma_nc
Nenhuma_nc	Nenhuma_nc	Nenhuma_nc	Menos_de_1	Menos_de_1	Nenhuma_nc	Cessacao_de	3_ou_mais_v
Nenhuma_nc	Menos_de_1	Nenhuma_nc	Nenhuma_nc	Nenhuma_nc	Nenhuma_nc	NULL	Nenhuma_nc
Menos_de_1	Nenhuma_nc	1_ou_2_veze	1_ou_2_veze	Menos_de_1	Menos_de_1	NULL	Menos_de_1
Menos_de_1	Nenhuma_nc	Nenhuma_nc	Menos_de_1	Menos_de_1	1_ou_2_veze	NULL	Nenhuma_nc
Nenhuma_nc	Nenhuma_nc	Nenhuma_nc	Nenhuma_nc	Nenhuma_nc	3_ou_mais_v	NULL	Nenhuma_nc
Menos_de_1	1_ou_2_veze	Nenhuma_nc	1_ou_2_veze	Menos_de_1	1_ou_2_veze	nenhuma	Nenhuma_nc
Nenhuma_nc	Nenhuma_nc	Nenhuma_nc	Nenhuma_nc	Nenhuma_nc	Nenhuma_nc	Preocupacac	Menos_de_1
Menos_de_1	Nenhuma_nc	Nenhuma_nc	Nenhuma_nc	Menos_de_1	Nenhuma_nc	NULL	Nenhuma_nc
Menos_de_1	Menos_de_1	Nenhuma_nc	Nenhuma_nc	Menos_de_1	Nenhuma_nc	NULL	Nenhuma_nc
Nenhuma_nc	Nenhuma_nc	Nenhuma_nc	Nenhuma_nc	Nenhuma_nc	3_ou_mais_v	NULL	3_ou_mais_v
Menos_de_1	Nenhuma_nc	Nenhuma_nc	3_ou_mais_v	Nenhuma_nc	3_ou_mais_v	NULL	3_ou_mais_v
Nenhuma_nc	Nenhuma_nc	Nenhuma_nc	Nenhuma_nc	Nenhuma_nc	Nenhuma_nc	NULL	Nenhuma_nc
Nenhuma_nc	Menos_de_1	Nenhuma_nc	Nenhuma_nc	Menos_de_1	Nenhuma_nc	NULL	Nenhuma_nc
Menos_de_1	Nenhuma_nc	Nenhuma_nc	Nenhuma_nc	Nenhuma_nc	Nenhuma_nc	NULL	Nenhuma_nc
Nenhuma_nc	Nenhuma_nc	Nenhuma_nc	Nenhuma_nc	Nenhuma_nc	Nenhuma_nc	NULL	Nenhuma_nc
Nenhuma_nc	Nenhuma_nc	Nenhuma_nc	Menos_de_1	Nenhuma_nc	Nenhuma_nc	Bebe_com_8	3_ou_mais_v

Nenhuma_nc	Nenhuma_nc	Nenhuma_nc	Nenhuma_nc	Nenhuma_nc	Nenhuma_nc	Pensando_e	1_ou_2_veze
Nenhuma_nc	Nenhuma_nc	Nenhuma_nc	Menos_de_1	Nenhuma_nc	Nenhuma_nc	NULL	Nenhuma_nc
Nenhuma_nc	Nenhuma_nc	Menos_de_1	Nenhuma_nc	3_ou_mais_v	Menos_de_1	NULL	Nenhuma_nc
Nenhuma_nc	Nenhuma_nc	Menos_de_1	Nenhuma_nc	Menos_de_1	Nenhuma_nc	NULL	Nenhuma_nc
Nenhuma_nc	Nenhuma_nc	Nenhuma_nc	1_ou_2_veze	Nenhuma_nc	Nenhuma_nc	NULL	Nenhuma_nc
Nenhuma_nc	Nenhuma_nc	Menos_de_1	1_ou_2_veze	Menos_de_1	3_ou_mais_v	Preocupacoe	3_ou_mais_v
Menos_de_1	Nenhuma_nc	Menos_de_1	3_ou_mais_v	1_ou_2_veze	Menos_de_1	Preocupacac	1_ou_2_veze
Nenhuma_nc	Nenhuma_nc	Nenhuma_nc	Nenhuma_nc	Nenhuma_nc	Nenhuma_nc	NULL	Nenhuma_nc
Nenhuma_nc	Nenhuma_nc	Menos_de_1	Nenhuma_nc	Nenhuma_nc	Nenhuma_nc	Ansiedade-_	1_ou_2_veze
Nenhuma_nc	Nenhuma_nc	Nenhuma_nc	Nenhuma_nc	Menos_de_1	Menos_de_1	NULL	Nenhuma_nc
Menos_de_1	Nenhuma_nc	1_ou_2_veze	Nenhuma_nc	Nenhuma_nc	Menos_de_1	NULL	Nenhuma_nc
Nenhuma_nc	1_ou_2_veze	Nenhuma_nc	1_ou_2_veze	Nenhuma_nc	Menos_de_1	NULL	Nenhuma_nc
Nenhuma_nc	Nenhuma_nc	Nenhuma_nc	Nenhuma_nc	Nenhuma_nc	Menos_de_1	NULL	Nenhuma_nc
Nenhuma_nc	Nenhuma_nc	Nenhuma_nc	Nenhuma_nc	1_ou_2_veze	Nenhuma_nc	NULL	Nenhuma_nc
Nenhuma_nc	Nenhuma_nc	Nenhuma_nc	Menos_de_1	3_ou_mais_v	Nenhuma_nc	NULL	Nenhuma_nc
Menos_de_1	Nenhuma_nc	Menos_de_1	Menos_de_1	Menos_de_1	1_ou_2_veze	NULL	Nenhuma_nc
Nenhuma_nc	Nenhuma_nc	1_ou_2_veze	1_ou_2_veze	Nenhuma_nc	Menos_de_1	NULL	Menos_de_1
Nenhuma_nc	Nenhuma_nc	Nenhuma_nc	Nenhuma_nc	Nenhuma_nc	Nenhuma_nc	NULL	Nenhuma_nc
Menos_de_1	Nenhuma_nc	Nenhuma_nc	3_ou_mais_v	Menos_de_1	Menos_de_1	Ansiedade_e	Menos_de_1
Nenhuma_nc	Nenhuma_nc	Nenhuma_nc	Nenhuma_nc	1_ou_2_veze	3_ou_mais_v	Agitacao	Menos_de_1
Nenhuma_nc	Nenhuma_nc	Nenhuma_nc	1_ou_2_veze	Menos_de_1	1_ou_2_veze	Simplesment	Menos_de_1
1_ou_2_veze	3_ou_mais_v	Nenhuma_nc	Nenhuma_nc	1_ou_2_veze	1_ou_2_veze	NULL	Nenhuma_nc
Nenhuma_nc	Nenhuma_nc	Nenhuma_nc	Nenhuma_nc	Menos_de_1	1_ou_2_veze	NULL	Nenhuma_nc
Nenhuma_nc	Nenhuma_nc	Menos_de_1	Nenhuma_nc	Menos_de_1	3_ou_mais_v	Dor_no_omb	3_ou_mais_v
Nenhuma_nc	Nenhuma_nc	Nenhuma_nc	Nenhuma_nc	Nenhuma_nc	Nenhuma_nc	NULL	Nenhuma_nc
Nenhuma_nc	Nenhuma_nc	Nenhuma_nc	1_ou_2_veze	Menos_de_1	Nenhuma_nc	Preocupacac	Menos_de_1
1_ou_2_veze	Menos_de_1	Nenhuma_nc	Nenhuma_nc	Nenhuma_nc	Nenhuma_nc	crianca_pequ	3_ou_mais_v
Nenhuma_nc	Nenhuma_nc	Nenhuma_nc	Nenhuma_nc	Nenhuma_nc	Nenhuma_nc	NULL	Nenhuma_nc
Menos_de_1	Nenhuma_nc	Nenhuma_nc	Menos_de_1	Nenhuma_nc	Nenhuma_nc	A_dor_que_s	Nenhuma_nc
Nenhuma_nc	Menos_de_1	Nenhuma_nc	Menos_de_1	Nenhuma_nc	Menos_de_1	Nao_	Nenhuma_nc
Nenhuma_nc	Nenhuma_nc	Nenhuma_nc	Nenhuma_nc	Nenhuma_nc	Nenhuma_nc	NULL	Nenhuma_nc
Nenhuma_nc	Nenhuma_nc	Nenhuma_nc	3_ou_mais_v	1_ou_2_veze	Nenhuma_nc	Meu_cachorr	1_ou_2_veze
Nenhuma_nc	Menos_de_1	Nenhuma_nc	Nenhuma_nc	Nenhuma_nc	Nenhuma_nc	nao	Nenhuma_nc
Nenhuma_nc	Nenhuma_nc	Nenhuma_nc	Nenhuma_nc	Nenhuma_nc	Nenhuma_nc	NULL	Nenhuma_nc
Menos_de_1	Nenhuma_nc	Nenhuma_nc	1_ou_2_veze	Menos_de_1	Nenhuma_nc	Dormi_bem	Nenhuma_nc
Nenhuma_nc	Nenhuma_nc	Nenhuma_nc	Nenhuma_nc	Nenhuma_nc	Nenhuma_nc	NULL	Nenhuma_nc
Nenhuma_nc	Nenhuma_nc	Nenhuma_nc	3_ou_mais_v	Nenhuma_nc	Nenhuma_nc	Ronco_do_m	3_ou_mais_v
Nenhuma_nc	Nenhuma_nc	Nenhuma_nc	1_ou_2_veze	3_ou_mais_v	Nenhuma_nc	NULL	Nenhuma_nc
Nenhuma_nc	Nenhuma_nc	Menos_de_1	Menos_de_1	1_ou_2_veze	Menos_de_1	NULL	Nenhuma_nc
Nenhuma_nc	Nenhuma_nc	Nenhuma_nc	Menos_de_1	Nenhuma_nc	1_ou_2_veze	NULL	Nenhuma_nc
Nenhuma_nc	Nenhuma_nc	Menos_de_1	Nenhuma_nc	Menos_de_1	Menos_de_1	NULL	Nenhuma_nc

durante_o_u	durante_o_u	no_ultimo_r	durante_o_u	voce_tem_u	se_voce_ter	longas_para	contracoes_
Boa	3_ou_mais_\	1_ou_2_veze	Um_problem	Parceiro_na_	Nenhuma_nc	Nenhuma_nc	Nenhuma_nc
Ruim	Nenhuma_nc	Nenhuma_nc	Um_problem	Nao	Nao_se_aplic	Nao_se_aplic	Nao_se_aplic
Muito_boa	Nenhuma_nc	Nenhuma_nc	Um_problem	Parceiro_na_	Nenhuma_nc	Nenhuma_nc	Nenhuma_nc
Muito_Ruim	1_ou_2_veze	1_ou_2_veze	Um_grande_	Nao	Nao_se_aplic	Nao_se_aplic	Nao_se_aplic
Boa	Nenhuma_nc	Nenhuma_nc	Um_problem	Parceiro_na_	Nenhuma_nc	Nenhuma_nc	1_ou_2_veze
Boa	Nenhuma_nc	Menos_de_1	Um_problem	Nao	Nao_se_aplic	Nao_se_aplic	Nao_se_aplic
Boa	Nenhuma_nc	Menos_de_1	Um_problem	Nao	Nao_se_aplic	Nao_se_aplic	Nao_se_aplic
Boa	Nenhuma_nc	Nenhuma_nc	Um_problem	Parceiro_ou_	Nao_se_aplic	Nao_se_aplic	Nao_se_aplic
Boa	Menos_de_1	Nenhuma_nc	Um_problem	Parceiro_na_	Nenhuma_nc	Nenhuma_nc	1_ou_2_veze
Muito_Ruim	3_ou_mais_\	Nenhuma_nc	Um_grande_	Nao	Nao_se_aplic	Nao_se_aplic	Nao_se_aplic
Muito_Ruim	Nenhuma_nc	Menos_de_1	Um_grande_	Nao	Nao_se_aplic	Nao_se_aplic	Nao_se_aplic
Boa	1_ou_2_veze	Nenhuma_nc	Um_grande_	Parceiro_na_	Menos_que_	Nenhuma_nc	Menos_que_
Boa	Nenhuma_nc	Menos_de_1	Um_problem	Parceiro_na_	Nenhuma_nc	Nenhuma_nc	3_ou_mais_\
Boa	Nenhuma_nc	Menos_de_1	Um_grande_	Nao	Nao_se_aplic	Nao_se_aplic	Nao_se_aplic
Ruim	Nenhuma_nc	1_ou_2_veze	Um_grande_	Nao	Nao_se_aplic	Nao_se_aplic	Nao_se_aplic
Ruim	Nenhuma_nc	Nenhuma_nc	Um_problem	Nao	Nao_se_aplic	Nao_se_aplic	Nao_se_aplic
Boa	Nenhuma_nc	Nenhuma_nc	Um_problem	Parceiro_na_	Nenhuma_nc	Nenhuma_nc	Nenhuma_nc
Boa	Nenhuma_nc	Nenhuma_nc	Um_problem	Parceiro_no_	Nenhuma_nc	Nenhuma_nc	Menos_que_
Boa	Nenhuma_nc	Nenhuma_nc	Um_grande_	Nao	Nao_se_aplic	Nao_se_aplic	Nao_se_aplic
Muito_boa	Nenhuma_nc	Nenhuma_nc	Nenhuma_di	Parceiro_na_	Nenhuma_nc	Nenhuma_nc	Nenhuma_nc
Boa	Nenhuma_nc	Menos_de_1	Um_problem	Parceiro_no_	Nenhuma_nc	Nenhuma_nc	Nenhuma_nc
Boa	Nenhuma_nc	Menos_de_1	Um_grande_	Parceiro_ou_	Nao_se_aplic	Nao_se_aplic	Nao_se_aplic
Boa	Nenhuma_nc	Nenhuma_nc	Um_problem	Parceiro_na_	1_ou_2_veze	Nenhuma_nc	Nenhuma_nc
Boa	Nenhuma_nc	Menos_de_1	Um_problem	Parceiro_na_	Nenhuma_nc	Nenhuma_nc	1_ou_2_veze
Muito_Ruim	3_ou_mais_\	1_ou_2_veze	Um_grande_	Parceiro_no_	Nenhuma_nc	Nenhuma_nc	1_ou_2_veze
Boa	Nenhuma_nc	Menos_de_1	Um_problem	Nao	Nao_se_aplic	Nao_se_aplic	Nao_se_aplic
Ruim	Menos_de_1	Nenhuma_nc	Um_problem	Parceiro_na_	Nenhuma_nc	Nenhuma_nc	Nenhuma_nc
Ruim	1_ou_2_veze	Menos_de_1	Um_grande_	Parceiro_na_	Nenhuma_nc	Nenhuma_nc	Nenhuma_nc
Boa	Nenhuma_nc	Menos_de_1	Um_problem	Parceiro_no_	Menos_que_	Nenhuma_nc	Nenhuma_nc
Boa	Nenhuma_nc	Menos_de_1	Um_problem	Parceiro_na_	Nenhuma_nc	Nenhuma_nc	Menos_que_
Ruim	Nenhuma_nc	Nenhuma_nc	Um_problem	Parceiro_na_	Nenhuma_nc	Nenhuma_nc	Nenhuma_nc
Boa	Nenhuma_nc	Nenhuma_nc	Um_grande_	Nao	Nao_se_aplic	Nao_se_aplic	Nao_se_aplic
Boa	Nenhuma_nc	Nenhuma_nc	Um_grande_	Nao	Nenhuma_nc	Nao_se_aplic	Nao_se_aplic
Ruim	Nenhuma_nc	Nenhuma_nc	Um_grande_	Nao	Nao_se_aplic	Nao_se_aplic	Nao_se_aplic
Boa	Nenhuma_nc	Nenhuma_nc	Um_grande_	Parceiro_na_	Nenhuma_nc	Nenhuma_nc	Nenhuma_nc
Muito_boa	Nenhuma_nc	Menos_de_1	Nenhuma_di	Parceiro_no_	Nenhuma_nc	Nenhuma_nc	1_ou_2_veze
Ruim	Nenhuma_nc	Menos_de_1	Um_problem	Parceiro_na_	Menos_que_	Menos_que_	Menos_que_
Ruim	Nenhuma_nc	Nenhuma_nc	Um_grande_	Parceiro_ou_	Nao_se_aplic	Nao_se_aplic	Nao_se_aplic
Boa	Nenhuma_nc	Menos_de_1	Um_grande_	Nao	Nao_se_aplic	Nao_se_aplic	Nao_se_aplic
Ruim	Nenhuma_nc	Nenhuma_nc	Um_problem	Nao	Nao_se_aplic	Nao_se_aplic	Nao_se_aplic
Boa	Nenhuma_nc	Nenhuma_nc	Um_grande_	Parceiro_no_	Menos_que_	Nenhuma_nc	Nenhuma_nc
Boa	Nenhuma_nc	Nenhuma_nc	Um_problem	Nao	Nao_se_aplic	Nao_se_aplic	Nao_se_aplic
Boa	Nenhuma_nc	Menos_de_1	Um_grande_	Parceiro_na_	Menos_que_	Nenhuma_nc	1_ou_2_veze
Ruim	Menos_de_1	Nenhuma_nc	Um_problem	Parceiro_no_	Nenhuma_nc	Nenhuma_nc	Menos_que_
Boa	Nenhuma_nc	Menos_de_1	Um_problem	Parceiro_na_	Nenhuma_nc	Nenhuma_nc	Menos_que_
Ruim	Nenhuma_nc	Nenhuma_nc	Um_problem	Parceiro_na_	Menos_que_	Menos_que_	3_ou_mais_\
Ruim	Nenhuma_nc	Nenhuma_nc	Nenhuma_di	Nao	Nao_se_aplic	Nao_se_aplic	Nao_se_aplic
Ruim	Nenhuma_nc	Menos_de_1	Um_problem	Parceiro_ou_	Nao_se_aplic	Nao_se_aplic	Nao_se_aplic
Boa	1_ou_2_veze	Nenhuma_nc	Um_problem	Nao	Nao_se_aplic	Nao_se_aplic	Nao_se_aplic
Boa	3_ou_mais_\	Nenhuma_nc	Um_problem	Nao	Nao_se_aplic	Nao_se_aplic	Nao_se_aplic
Boa	Menos_de_1	Menos_de_1	Um_problem	Nao	Nao_se_aplic	Nao_se_aplic	Nao_se_aplic
Boa	Nenhuma_nc	Menos_de_1	Um_problem	Nao	Nao_se_aplic	Nao_se_aplic	Nao_se_aplic
Ruim	3_ou_mais_\	3_ou_mais_\	Um_problem	Parceiro_ou_	Nenhuma_nc	Nenhuma_nc	Nenhuma_nc
Boa	Nenhuma_nc	Menos_de_1	Um_grande_	Parceiro_na_	Nenhuma_nc	Nenhuma_nc	3_ou_mais_\
Boa	Nenhuma_nc	Menos_de_1	Um_problem	Nao	Nao_se_aplic	Nao_se_aplic	Nao_se_aplic

Ruim	Nenhuma_nc	Nenhuma_nc	Um_problem	Parceiro_na_	Nenhuma_nc	Nenhuma_nc	3_ou_mais_\
Ruim	Nenhuma_nc	Nenhuma_nc	Um_problem	Nao	Nao_se_aplic	Nao_se_aplic	Nao_se_aplic
Ruim	Nenhuma_nc	Menos_de_1	Um_grande_	Parceiro_ou_	Nenhuma_nc	Nenhuma_nc	Menos_que_
Boa	3_ou_mais_\	Menos_de_1	Um_problem	Parceiro_ou_	Nao_se_aplic	Nao_se_aplic	Nao_se_aplic
Boa	Nenhuma_nc	Menos_de_1	Um_grande_	Nao	Nao_se_aplic	Nao_se_aplic	Nao_se_aplic
Boa	Nenhuma_nc	Nenhuma_nc	Nenhuma_di	Parceiro_na_	Menos_que_	Nenhuma_nc	1_ou_2_veze
Ruim	Nenhuma_nc	Menos_de_1	Um_grande_	Nao	Nao_se_aplic	Nao_se_aplic	Nao_se_aplic
Muito_boa	Nenhuma_nc	Nenhuma_nc	Um_problem	Parceiro_na_	3_ou_mais_\	1_ou_2_veze	3_ou_mais_\
Boa	Nenhuma_nc	Menos_de_1	Um_grande_	Nao	Nao_se_aplic	Nao_se_aplic	Nao_se_aplic
Muito_boa	Nenhuma_nc	Nenhuma_nc	Um_problem	Parceiro_na_	Nenhuma_nc	Nenhuma_nc	1_ou_2_veze
Muito_boa	Nenhuma_nc	Menos_de_1	Um_problem	Parceiro_na_	Menos_que_	Nenhuma_nc	Nenhuma_nc
Ruim	Nenhuma_nc	Menos_de_1	Um_grande_	Parceiro_na_	Nenhuma_nc	Nenhuma_nc	3_ou_mais_\
Boa	Nenhuma_nc	Nenhuma_nc	Nenhuma_di	Parceiro_na_	Nenhuma_nc	Nenhuma_nc	Nenhuma_nc
Boa	Nenhuma_nc	Menos_de_1	Um_problem	Parceiro_na_	Nenhuma_nc	Nenhuma_nc	Nenhuma_nc
Muito_boa	Nenhuma_nc	Nenhuma_nc	Nenhuma_di	Parceiro_na_	Nenhuma_nc	Nenhuma_nc	Nenhuma_nc
Boa	Nenhuma_nc	Nenhuma_nc	Nenhuma_di	Nao	Nao_se_aplic	Nao_se_aplic	Nao_se_aplic
Boa	Nenhuma_nc	Nenhuma_nc	Um_problem	Parceiro_na_	Nenhuma_nc	Nenhuma_nc	3_ou_mais_\
Muito_boa	Nenhuma_nc	Nenhuma_nc	Um_problem	Parceiro_na_	3_ou_mais_\	Nenhuma_nc	Menos_que_
Ruim	Nenhuma_nc	Nenhuma_nc	Um_problem	Parceiro_ou_	3_ou_mais_\	Nao_se_aplic	1_ou_2_veze
Boa	3_ou_mais_\	Menos_de_1	Um_problem	Parceiro_na_	3_ou_mais_\	Nenhuma_nc	Nenhuma_nc
Ruim	Nenhuma_nc	Nenhuma_nc	Um_problem	Parceiro_no_	Nenhuma_nc	1_ou_2_veze	Menos_que_
Boa	Nenhuma_nc	Nenhuma_nc	Nenhuma_di	Parceiro_na_	Nenhuma_nc	Nenhuma_nc	Nenhuma_nc
Boa	Nenhuma_nc	Nenhuma_nc	Um_problem	Nao	Nao_se_aplic	Nao_se_aplic	Nao_se_aplic
Boa	Nenhuma_nc	Nenhuma_nc	Um_problem	Parceiro_na_	Nenhuma_nc	Nenhuma_nc	Nenhuma_nc
Ruim	Nenhuma_nc	Menos_de_1	Um_problem	Parceiro_ou_	Nao_se_aplic	Nao_se_aplic	Nao_se_aplic
Ruim	Nenhuma_nc	1_ou_2_veze	Um_problem	Parceiro_no_	Nao_se_aplic	Nao_se_aplic	Nao_se_aplic
Muito_boa	Nenhuma_nc	Nenhuma_nc	Um_problem	Parceiro_na_	1_ou_2_veze	Nenhuma_nc	Menos_que_
Boa	Nenhuma_nc	Nenhuma_nc	Um_problem	Nao	Nao_se_aplic	Nao_se_aplic	Nao_se_aplic
Boa	Nenhuma_nc	3_ou_mais_\	Um_grande_	Parceiro_na_	Nenhuma_nc	Nenhuma_nc	Menos_que_
Boa	3_ou_mais_\	Nenhuma_nc	Um_problem	Nao	Nao_se_aplic	Nao_se_aplic	Nao_se_aplic
Ruim	3_ou_mais_\	1_ou_2_veze	Um_problem	Parceiro_ou_	Menos_que_	Nenhuma_nc	Nenhuma_nc
Muito_boa	Nenhuma_nc	Nenhuma_nc	Nenhuma_di	Parceiro_ou_	Nenhuma_nc	Nenhuma_nc	Nenhuma_nc
Boa	Nenhuma_nc	Menos_de_1	Um_problem	Nao	Nao_se_aplic	Nao_se_aplic	Nao_se_aplic
Boa	Nenhuma_nc	Nenhuma_nc	Um_problem	Nao	Nao_se_aplic	Nao_se_aplic	Nao_se_aplic
Boa	Nenhuma_nc	Menos_de_1	Um_problem	Parceiro_na_	Nenhuma_nc	Nenhuma_nc	Menos_que_
Boa	Nenhuma_nc	Nenhuma_nc	Um_grande_	Nao	Nao_se_aplic	Nao_se_aplic	Menos_que_
Ruim	Nenhuma_nc	Nenhuma_nc	Um_problem	Nao	Nao_se_aplic	Nao_se_aplic	Nao_se_aplic
Boa	Nenhuma_nc	Nenhuma_nc	Um_problem	Nao	Nao_se_aplic	Nao_se_aplic	Nao_se_aplic
Muito_Ruim	3_ou_mais_\	1_ou_2_veze	Um_grande_	Nao	Nao_se_aplic	Nao_se_aplic	Nao_se_aplic
Boa	Menos_de_1	Nenhuma_nc	Um_problem	Nao	Nao_se_aplic	Nao_se_aplic	Nao_se_aplic
Ruim	3_ou_mais_\	1_ou_2_veze	Um_problem	Parceiro_ou_	Nenhuma_nc	Nenhuma_nc	3_ou_mais_\
Boa	Nenhuma_nc	Nenhuma_nc	Um_problem	Nao	Nao_se_aplic	Nao_se_aplic	Nao_se_aplic
Ruim	1_ou_2_veze	Nenhuma_nc	Um_problem	Parceiro_ou_	Nao_se_aplic	Nao_se_aplic	Nao_se_aplic
Muito_boa	Nenhuma_nc	Menos_de_1	Um_grande_	Parceiro_na_	Nenhuma_nc	Nenhuma_nc	Nenhuma_nc
Ruim	Nenhuma_nc	1_ou_2_veze	Um_problem	Parceiro_na_	Nenhuma_nc	Nenhuma_nc	3_ou_mais_\
Boa	Nenhuma_nc	Nenhuma_nc	Um_grande_	Parceiro_na_	Menos_que_	Nenhuma_nc	Menos_que_
Boa	Nenhuma_nc	Nenhuma_nc	Um_problem	Parceiro_na_	1_ou_2_veze	Nenhuma_nc	Menos_que_
Boa	Nenhuma_nc	Nenhuma_nc	Nenhuma_di	Parceiro_na_	Nenhuma_nc	Nenhuma_nc	1_ou_2_veze
Boa	Nenhuma_nc	Menos_de_1	Um_problem	Nao	Nao_se_aplic	Nao_se_aplic	Nao_se_aplic
Boa	Nenhuma_nc	Nenhuma_nc	Um_grande_	Parceiro_na_	Nenhuma_nc	Nenhuma_nc	1_ou_2_veze
Boa	Nenhuma_nc	Nenhuma_nc	Um_grande_	Parceiro_na_	Nenhuma_nc	Nenhuma_nc	3_ou_mais_\
Boa	Nenhuma_nc	Menos_de_1	Um_grande_	Nao	Nao_se_aplic	Nao_se_aplic	Nao_se_aplic
Boa	3_ou_mais_\	1_ou_2_veze	Um_grande_	Parceiro_na_	Nenhuma_nc	Nenhuma_nc	Nenhuma_nc
Ruim	Nenhuma_nc	Nenhuma_nc	Um_problem	Nao	Nao_se_aplic	Nao_se_aplic	Nao_se_aplic
Ruim	3_ou_mais_\	Menos_de_1	Um_problem	Parceiro_na_	Menos_que_	Nenhuma_nc	3_ou_mais_\
Muito_Ruim	Menos_de_1	Menos_de_1	Um_problem	Parceiro_ou_	3_ou_mais_\	3_ou_mais_\	3_ou_mais_\
Ruim	3_ou_mais_\	3_ou_mais_\	Um_grande_	Parceiro_na_	Nenhuma_nc	Nenhuma_nc	Nenhuma_nc
Boa	Nenhuma_nc	1_ou_2_veze	Um_problem	Nao	Nao_se_aplic	Nenhuma_nc	Nenhuma_nc

Ruim	Nenhuma_nc	Nenhuma_nc	Um_problem	Parceiro_na_	Nenhuma_nc	Nenhuma_nc	Menos_que_
Boa	3_ou_mais_\	Menos_de_1	Um_problem	Nao	Nao_se_aplic	Nao_se_aplic	Nao_se_aplic
Ruim	Nenhuma_nc	Menos_de_1	Um_grande_	Parceiro_ou_	Nao_se_aplic	Nao_se_aplic	Nao_se_aplic
Boa	Nenhuma_nc	Nenhuma_nc	Um_problem	Nao	Nao_se_aplic	Nao_se_aplic	Nao_se_aplic
Boa	Nenhuma_nc	Nenhuma_nc	Nenhuma_di	Parceiro_na_	Nenhuma_nc	Nenhuma_nc	Nenhuma_nc
Boa	Nenhuma_nc	Menos_de_1	Um_problem	Parceiro_na_	1_ou_2_veze	Nenhuma_nc	Menos_que_
Boa	Nenhuma_nc	Nenhuma_nc	Um_problem	Nao	Nao_se_aplic	Nao_se_aplic	Nao_se_aplic
Ruim	Nenhuma_nc	Menos_de_1	Um_grande_	Nao	Nao_se_aplic	Nao_se_aplic	Nao_se_aplic
Boa	Nenhuma_nc	Nenhuma_nc	Um_problem	Nao	Nao_se_aplic	Nao_se_aplic	Nao_se_aplic
Boa	Nenhuma_nc	Nenhuma_nc	Um_problem	Nao	Nao_se_aplic	Nao_se_aplic	Nao_se_aplic
Ruim	Nenhuma_nc	Menos_de_1	Um_problem	Parceiro_no_	Nenhuma_nc	Nenhuma_nc	Nenhuma_nc
Boa	Nenhuma_nc	Menos_de_1	Um_problem	Parceiro_na_	Nenhuma_nc	Nenhuma_nc	Nenhuma_nc
Ruim	Nenhuma_nc	Menos_de_1	Um_problem	Parceiro_ou_	Nenhuma_nc	Nenhuma_nc	Menos_que_
Boa	Nenhuma_nc	Menos_de_1	Um_problem	Parceiro_na_	1_ou_2_veze	Nenhuma_nc	Nenhuma_nc
Muito_boa	Nenhuma_nc	Nenhuma_nc	Um_problem	Parceiro_na_	Nenhuma_nc	Nenhuma_nc	Nenhuma_nc
Ruim	Nenhuma_nc	Nenhuma_nc	Um_grande_	Nao	Nao_se_aplic	Nao_se_aplic	Nao_se_aplic
Boa	Nenhuma_nc	3_ou_mais_\	Um_grande_	Parceiro_ou_	3_ou_mais_\	3_ou_mais_\	Nenhuma_nc
Ruim	Nenhuma_nc	1_ou_2_veze	Um_problem	Nao	Nao_se_aplic	Nao_se_aplic	Nao_se_aplic
Boa	1_ou_2_veze	Menos_de_1	Um_grande_	Parceiro_na_	Nenhuma_nc	Nenhuma_nc	1_ou_2_veze
Boa	Menos_de_1	Nenhuma_nc	Um_problem	Parceiro_na_	Nenhuma_nc	Nenhuma_nc	3_ou_mais_\
Ruim	Menos_de_1	Nenhuma_nc	Um_problem	Nao	Nao_se_aplic	Nao_se_aplic	Nenhuma_nc
Boa	Nenhuma_nc	Nenhuma_nc	Um_problem	Parceiro_na_	Menos_que_	Nao_se_aplic	Nao_se_aplic
Ruim	Nenhuma_nc	Menos_de_1	Um_problem	Nao	Nao_se_aplic	Nao_se_aplic	Nao_se_aplic
Boa	Nenhuma_nc	Menos_de_1	Um_problem	Parceiro_na_	1_ou_2_veze	Menos_que_	3_ou_mais_\
Ruim	Menos_de_1	Nenhuma_nc	Um_problem	Nao	Nao_se_aplic	Nao_se_aplic	Nao_se_aplic
Ruim	Nenhuma_nc	Nenhuma_nc	Um_grande_	Nao	Nao_se_aplic	Nao_se_aplic	Nao_se_aplic
Ruim	Menos_de_1	Menos_de_1	Um_grande_	Nao	Nao_se_aplic	Nenhuma_nc	Nenhuma_nc
Ruim	Nenhuma_nc	Nenhuma_nc	Um_problem	Nao	Nao_se_aplic	Menos_que_	Nao_se_aplic
Boa	Nenhuma_nc	Nenhuma_nc	Nenhuma_di	Parceiro_na_	Nenhuma_nc	Nenhuma_nc	Nenhuma_nc
Boa	Nenhuma_nc	Menos_de_1	Um_problem	Parceiro_na_	Nenhuma_nc	Nenhuma_nc	3_ou_mais_\
Ruim	3_ou_mais_\	Nenhuma_nc	Um_grande_	Parceiro_no_	Menos_que_	Nao_se_aplic	1_ou_2_veze
Boa	Nenhuma_nc	Menos_de_1	Nenhuma_di	Nao	Nao_se_aplic	Nao_se_aplic	Nao_se_aplic
Boa	Menos_de_1	Nenhuma_nc	Um_grande_	Nao	Nao_se_aplic	Nao_se_aplic	Nao_se_aplic
Boa	Nenhuma_nc	Nenhuma_nc	Um_problem	Parceiro_na_	Menos_que_	Nenhuma_nc	Nenhuma_nc
Boa	Menos_de_1	Menos_de_1	Um_problem	Nao	Nao_se_aplic	Nao_se_aplic	Nao_se_aplic
Boa	Nenhuma_nc	Nenhuma_nc	Um_problem	Nao	Nao_se_aplic	Nao_se_aplic	Nao_se_aplic
Muito_boa	1_ou_2_veze	Nenhuma_nc	Um_grande_	Parceiro_na_	Nenhuma_nc	Nao_se_aplic	Nao_se_aplic
Boa	Nenhuma_nc	Nenhuma_nc	Um_grande_	Parceiro_na_	3_ou_mais_\	Nenhuma_nc	Nenhuma_nc
Boa	Nenhuma_nc	Nenhuma_nc	Nenhuma_di	Parceiro_na_	3_ou_mais_\	Menos_que_	Nenhuma_nc
Ruim	Nenhuma_nc	Nenhuma_nc	Nenhuma_di	Parceiro_na_	Nenhuma_nc	Nenhuma_nc	Menos_que_
Muito_boa	Nenhuma_nc	Nenhuma_nc	Um_problem	Parceiro_no_	Nenhuma_nc	Nenhuma_nc	Nenhuma_nc
Boa	Nenhuma_nc	Nenhuma_nc	Um_problem	Parceiro_na_	Nenhuma_nc	Nenhuma_nc	Nenhuma_nc
Boa	Nenhuma_nc	Nenhuma_nc	Um_problem	Parceiro_na_	Nenhuma_nc	Nenhuma_nc	3_ou_mais_\
Boa	Nenhuma_nc	Menos_de_1	Um_problem	Parceiro_na_	Nenhuma_nc	Nenhuma_nc	Menos_que_
Boa	Nenhuma_nc	Nenhuma_nc	Nenhuma_di	Parceiro_na_	3_ou_mais_\	Nenhuma_nc	Nenhuma_nc
Boa	Nenhuma_nc	Nenhuma_nc	Um_problem	Parceiro_na_	Nenhuma_nc	Nenhuma_nc	Menos_que_
Ruim	Nenhuma_nc	1_ou_2_veze	Um_problem	Parceiro_na_	Nenhuma_nc	Nenhuma_nc	Nenhuma_nc
Ruim	Nenhuma_nc	Menos_de_1	Um_problem	Nao	Nao_se_aplic	Nao_se_aplic	Nao_se_aplic
Boa	Menos_de_1	Nenhuma_nc	Um_problem	Parceiro_na_	Menos_que_	Nenhuma_nc	Nenhuma_nc
Ruim	Nenhuma_nc	Nenhuma_nc	Um_problem	Nao	Nao_se_aplic	Nao_se_aplic	Nao_se_aplic
Ruim	Menos_de_1	Nenhuma_nc	Um_grande_	Nao	Nao_se_aplic	Nao_se_aplic	Nao_se_aplic
Boa	Nenhuma_nc	Nenhuma_nc	Um_problem	Nao	Nao_se_aplic	Nao_se_aplic	Nao_se_aplic
Boa	Nenhuma_nc	Nenhuma_nc	Um_problem	Nao	1_ou_2_veze	Nenhuma_nc	Nenhuma_nc
Ruim	Nenhuma_nc	Menos_de_1	Um_problem	Parceiro_na_	Menos_que_	Nenhuma_nc	Nenhuma_nc
Ruim	Nenhuma_nc	1_ou_2_veze	Um_grande_	Parceiro_na_	1_ou_2_veze	Nenhuma_nc	Menos_que_
Ruim	Nenhuma_nc	1_ou_2_veze	Um_grande_	Nao	Nao_se_aplic	Nao_se_aplic	Nao_se_aplic
Boa	Nenhuma_nc	1_ou_2_veze	Um_problem	Nao	Nao_se_aplic	Nao_se_aplic	Nao_se_aplic
Boa	Nenhuma_nc	Nenhuma_nc	Um_problem	Parceiro_na_	Nenhuma_nc	Nenhuma_nc	Menos_que_

Boa	Nenhuma_nc	Menos_de_1	Um_problem	Parceiro_na_	1_ou_2_veze	Nenhuma_nc	Nenhuma_nc
Boa	Nenhuma_nc	Menos_de_1	Um_problem	Parceiro_na_	Nenhuma_nc	Nenhuma_nc	Menos_que_
Ruim	1_ou_2_veze	Menos_de_1	Um_problem	Parceiro_na_	1_ou_2_veze	Menos_que_	3_ou_mais_\
Boa	Nenhuma_nc	Menos_de_1	Um_problem	Parceiro_na_	Nenhuma_nc	Nenhuma_nc	Menos_que_
Boa	Nenhuma_nc	Menos_de_1	Um_problem	Parceiro_na_	Nenhuma_nc	Nenhuma_nc	1_ou_2_veze
Muito_Ruim	Nenhuma_nc	Nenhuma_nc	Um_grande_	Parceiro_na_	3_ou_mais_\	3_ou_mais_\	Nenhuma_nc
Boa	Nenhuma_nc	Nenhuma_nc	Um_problem	Parceiro_na_	Nenhuma_nc	Nenhuma_nc	Nenhuma_nc
Muito_boa	Nenhuma_nc	Nenhuma_nc	Nenhuma_di	Nao	Nao_se_aplic	Nao_se_aplic	Nao_se_aplic
Boa	Nenhuma_nc	1_ou_2_veze	Um_problem	Parceiro_na_	Nenhuma_nc	Nenhuma_nc	Nenhuma_nc
Ruim	Nenhuma_nc	Menos_de_1	Um_problem	Parceiro_na_	Nenhuma_nc	Nenhuma_nc	Menos_que_
Muito_Ruim	3_ou_mais_\	Menos_de_1	Um_problem	Parceiro_na_	1_ou_2_veze	Nenhuma_nc	3_ou_mais_\
Boa	Nenhuma_nc	Menos_de_1	Um_problem	Parceiro_na_	1_ou_2_veze	Nenhuma_nc	Menos_que_
Boa	Nenhuma_nc	Nenhuma_nc	Um_problem	Parceiro_ou_	3_ou_mais_\	Nenhuma_nc	Menos_que_
Boa	Menos_de_1	Nenhuma_nc	Um_problem	Parceiro_na_	Nenhuma_nc	Nenhuma_nc	Nenhuma_nc
Boa	Nenhuma_nc	Nenhuma_nc	Um_problem	Parceiro_na_	Nenhuma_nc	Nenhuma_nc	Nenhuma_nc
Boa	Nenhuma_nc	Nenhuma_nc	Um_grande_	Parceiro_na_	Menos_que_	Nenhuma_nc	1_ou_2_veze
Ruim	1_ou_2_veze	Nenhuma_nc	Um_problem	Parceiro_na_	Nenhuma_nc	Nenhuma_nc	Menos_que_
Muito_boa	Nenhuma_nc	Nenhuma_nc	Um_problem	Parceiro_na_	Nenhuma_nc	Nenhuma_nc	Menos_que_
Boa	3_ou_mais_\	Nenhuma_nc	Um_problem	Parceiro_na_	Nenhuma_nc	Nenhuma_nc	3_ou_mais_\
Boa	Nenhuma_nc	Nenhuma_nc	Um_problem	Parceiro_na_	Nenhuma_nc	Nenhuma_nc	Menos_que_
Ruim	Nenhuma_nc	Nenhuma_nc	Um_grande_	Nao	Nao_se_aplic	Nao_se_aplic	Nao_se_aplic
Boa	Nenhuma_nc	Nenhuma_nc	Um_problem	Parceiro_na_	Menos_que_	Nenhuma_nc	Nenhuma_nc
Ruim	3_ou_mais_\	Menos_de_1	Um_grande_	Nao	Nao_se_aplic	Nao_se_aplic	Nao_se_aplic
Muito_boa	Nenhuma_nc	Nenhuma_nc	Nenhuma_di	Parceiro_na_	Nenhuma_nc	Nenhuma_nc	Nenhuma_nc
Boa	Nenhuma_nc	Nenhuma_nc	Um_problem	Parceiro_na_	1_ou_2_veze	Nao_se_aplic	Nao_se_aplic
Ruim	1_ou_2_veze	1_ou_2_veze	Um_problem	Parceiro_na_	Nenhuma_nc	Nenhuma_nc	1_ou_2_veze
Ruim	Menos_de_1	Nenhuma_nc	Um_problem	Parceiro_na_	Nenhuma_nc	Nenhuma_nc	Menos_que_
Boa	3_ou_mais_\	Nenhuma_nc	Nenhuma_di	Parceiro_ou_	Nenhuma_nc	Nenhuma_nc	Nenhuma_nc
Ruim	Nenhuma_nc	Nenhuma_nc	Um_problem	Parceiro_na_	Menos_que_	Nenhuma_nc	Menos_que_
Ruim	Nenhuma_nc	Nenhuma_nc	Um_problem	Nao	Nao_se_aplic	Nao_se_aplic	Menos_que_
Ruim	Nenhuma_nc	Nenhuma_nc	Um_problem	Parceiro_ou_	Nao_se_aplic	Nao_se_aplic	Nao_se_aplic
Boa	Nenhuma_nc	Nenhuma_nc	Um_problem	Nao	Nao_se_aplic	Nao_se_aplic	Nao_se_aplic
Muito_Ruim	Nenhuma_nc	Nenhuma_nc	Um_grande_	Parceiro_na_	Nenhuma_nc	Nenhuma_nc	Nenhuma_nc
Boa	Nenhuma_nc	Nenhuma_nc	Um_problem	Parceiro_na_	Menos_que_	Nenhuma_nc	Menos_que_
Ruim	1_ou_2_veze	Nenhuma_nc	Um_problem	Parceiro_na_	Nenhuma_nc	Nenhuma_nc	Nenhuma_nc
Ruim	Menos_de_1	Menos_de_1	Um_problem	Nao	Nao_se_aplic	Nao_se_aplic	Nao_se_aplic
Ruim	3_ou_mais_\	3_ou_mais_\	Um_grande_	Parceiro_na_	Nenhuma_nc	Menos_que_	Menos_que_
Boa	Nenhuma_nc	Nenhuma_nc	Um_problem	Parceiro_na_	Nenhuma_nc	Nenhuma_nc	Nenhuma_nc
Ruim	Nenhuma_nc	Menos_de_1	Um_problem	Nao	Nao_se_aplic	Nao_se_aplic	Nao_se_aplic
Boa	Nenhuma_nc	Menos_de_1	Um_grande_	Parceiro_na_	3_ou_mais_\	Nenhuma_nc	Nenhuma_nc
Boa	Nenhuma_nc	Nenhuma_nc	Um_problem	Parceiro_na_	1_ou_2_veze	Menos_que_	Menos_que_
Boa	Nenhuma_nc	Menos_de_1	Um_problem	Parceiro_no_	Menos_que_	Nenhuma_nc	Menos_que_
Boa	Nenhuma_nc	Menos_de_1	Um_problem	Parceiro_na_	Nenhuma_nc	Nenhuma_nc	Menos_que_
Boa	Nenhuma_nc	Nenhuma_nc	Um_problem	Parceiro_na_	Menos_que_	Nenhuma_nc	Nenhuma_nc
Boa	Nenhuma_nc	Nenhuma_nc	Um_problem	Nao	Nao_se_aplic	Nao_se_aplic	Menos_que_
Muito_Ruim	Nenhuma_nc	Nenhuma_nc	Nenhuma_di	Nao	Nao_se_aplic	Nao_se_aplic	Nao_se_aplic
Boa	Nenhuma_nc	Nenhuma_nc	Um_problem	Parceiro_na_	1_ou_2_veze	Menos_que_	1_ou_2_veze
Boa	Menos_de_1	Nenhuma_nc	Um_grande_	Parceiro_na_	Menos_que_	Nenhuma_nc	1_ou_2_veze
Boa	Nenhuma_nc	Nenhuma_nc	Um_problem	Nao	Nao_se_aplic	Nao_se_aplic	Nao_se_aplic
Boa	Nenhuma_nc	1_ou_2_veze	Um_problem	Parceiro_na_	Menos_que_	Nenhuma_nc	Nenhuma_nc
Boa	Nenhuma_nc	Nenhuma_nc	Um_problem	Parceiro_ou_	3_ou_mais_\	3_ou_mais_\	Nenhuma_nc
Ruim	3_ou_mais_\	Nenhuma_nc	Um_grande_	Nao	Nao_se_aplic	Nao_se_aplic	Nao_se_aplic
Ruim	Nenhuma_nc	Nenhuma_nc	Um_grande_	Nao	Nao_se_aplic	Nao_se_aplic	Nao_se_aplic
Ruim	Menos_de_1	Nenhuma_nc	Um_problem	Parceiro_ou_	Nao_se_aplic	Nao_se_aplic	Nao_se_aplic
Ruim	Nenhuma_nc	Nenhuma_nc	Um_grande_	Parceiro_na_	Menos_que_	Nenhuma_nc	Nenhuma_nc
Boa	Nenhuma_nc	Nenhuma_nc	Um_problem	Parceiro_na_	Nenhuma_nc	Nenhuma_nc	Nenhuma_nc
Boa	Nenhuma_nc	Nenhuma_nc	Um_problem	Parceiro_na_	1_ou_2_veze	Nenhuma_nc	Nenhuma_nc
Boa	Nenhuma_nc	Menos_de_1	Um_grande_	Parceiro_na_	Nenhuma_nc	Nenhuma_nc	Menos_que_

Boa	Nenhuma_nc	Nenhuma_nc	Um_grande_	Parceiro_na_	Nenhuma_nc	Nenhuma_nc	Menos_que_
Boa	3_ou_mais_\	Nenhuma_nc	Um_problem	Parceiro_na_	Menos_que_	Nenhuma_nc	Menos_que_
Ruim	Nenhuma_nc	Menos_de_1	Um_problem	Parceiro_na_	Nenhuma_nc	Nenhuma_nc	1_ou_2_veze
Ruim	Nenhuma_nc	Menos_de_1	Um_problem	Parceiro_na_	3_ou_mais_\	Menos_que_	Nenhuma_nc
Boa	Menos_de_1	Nenhuma_nc	Um_problem	Parceiro_na_	Nenhuma_nc	Nenhuma_nc	Menos_que_
Ruim	Nenhuma_nc	Nenhuma_nc	Um_problem	Parceiro_na_	Nao_se_aplic	Nao_se_aplic	1_ou_2_veze
Boa	Nenhuma_nc	Nenhuma_nc	Um_problem	Parceiro_na_	1_ou_2_veze	Nenhuma_nc	Nenhuma_nc
Boa	Nenhuma_nc	Menos_de_1	Um_problem	Nao	Nao_se_aplic	Nao_se_aplic	Nao_se_aplic
Boa	Nenhuma_nc	Nenhuma_nc	Um_problem	Parceiro_na_	Nenhuma_nc	Nenhuma_nc	Nenhuma_nc
Boa	1_ou_2_veze	Nenhuma_nc	Um_problem	Parceiro_na_	3_ou_mais_\	Nenhuma_nc	Nenhuma_nc
Boa	Nenhuma_nc	Nenhuma_nc	Um_problem	Nao	Nao_se_aplic	Nao_se_aplic	Nao_se_aplic
Ruim	3_ou_mais_\	Nenhuma_nc	Um_problem	Parceiro_na_	1_ou_2_veze	Nenhuma_nc	Menos_que_
Muito_boa	Nenhuma_nc	Nenhuma_nc	Um_problem	Parceiro_na_	Nenhuma_nc	Nenhuma_nc	Nenhuma_nc
Boa	Nenhuma_nc	Nenhuma_nc	Um_problem	Nao	Nao_se_aplic	Nao_se_aplic	Nao_se_aplic
Boa	Nenhuma_nc	Nenhuma_nc	Um_problem	Parceiro_na_	Nenhuma_nc	Nenhuma_nc	Nenhuma_nc
Ruim	Nenhuma_nc	Nenhuma_nc	Um_problem	Parceiro_no_	Nenhuma_nc	Nenhuma_nc	Nenhuma_nc
Ruim	Menos_de_1	Nenhuma_nc	Um_problem	Parceiro_na_	Menos_que_	Nenhuma_nc	Nenhuma_nc
Boa	3_ou_mais_\	Nenhuma_nc	Um_problem	Nao	Nao_se_aplic	Nao_se_aplic	Nao_se_aplic
Ruim	Nenhuma_nc	Menos_de_1	Um_problem	Nao	Nao_se_aplic	Nao_se_aplic	Menos_que_
Ruim	Nenhuma_nc	Menos_de_1	Um_grande_	Parceiro_na_	1_ou_2_veze	Nao_se_aplic	Menos_que_
Boa	Menos_de_1	Nenhuma_nc	Um_problem	Parceiro_na_	Nenhuma_nc	Nenhuma_nc	Nenhuma_nc
Muito_boa	Nenhuma_nc	Nenhuma_nc	Um_problem	Parceiro_na_	Nenhuma_nc	Nenhuma_nc	Menos_que_
Boa	Nenhuma_nc	3_ou_mais_\	Um_grande_	Parceiro_ou_	3_ou_mais_\	Menos_que_	Nenhuma_nc
Muito_Ruim	3_ou_mais_\	1_ou_2_veze	Um_grande_	Parceiro_na_	Menos_que_	Menos_que_	Nenhuma_nc
Ruim	Nenhuma_nc	Nenhuma_nc	Um_problem	Parceiro_na_	Nenhuma_nc	Nenhuma_nc	1_ou_2_veze
Ruim	Nenhuma_nc	Menos_de_1	Um_problem	Nao	Nao_se_aplic	Nao_se_aplic	Nao_se_aplic
Muito_boa	Nenhuma_nc	Nenhuma_nc	Nenhuma_di	Parceiro_na_	Nenhuma_nc	Nenhuma_nc	Menos_que_
Ruim	3_ou_mais_\	Nenhuma_nc	Um_grande_	Nao	Nao_se_aplic	Nao_se_aplic	Nao_se_aplic
Ruim	Nenhuma_nc	Menos_de_1	Um_problem	Parceiro_na_	3_ou_mais_\	Nenhuma_nc	Nenhuma_nc
Muito_Ruim	Nenhuma_nc	Menos_de_1	Um_problem	Parceiro_na_	3_ou_mais_\	3_ou_mais_\	1_ou_2_veze
Boa	Nenhuma_nc	Nenhuma_nc	Um_problem	Parceiro_na_	Nenhuma_nc	Nenhuma_nc	Nenhuma_nc
Ruim	Nenhuma_nc	Menos_de_1	Um_problem	Parceiro_na_	Nenhuma_nc	Nenhuma_nc	1_ou_2_veze
Ruim	Menos_de_1	Menos_de_1	Um_grande_	Nao	Nao_se_aplic	Nao_se_aplic	Nao_se_aplic
Ruim	1_ou_2_veze	1_ou_2_veze	Um_problem	Parceiro_na_	Nenhuma_nc	Nenhuma_nc	Nenhuma_nc
Muito_boa	Nenhuma_nc	Nenhuma_nc	Nenhuma_di	Parceiro_na_	Nenhuma_nc	Nenhuma_nc	Nenhuma_nc
Ruim	Menos_de_1	Nenhuma_nc	Um_problem	Parceiro_na_	3_ou_mais_\	Nenhuma_nc	Nenhuma_nc
Boa	Nenhuma_nc	Menos_de_1	Um_problem	Nao	Nao_se_aplic	Nao_se_aplic	Nao_se_aplic
Ruim	Nenhuma_nc	Menos_de_1	Um_grande_	Parceiro_na_	1_ou_2_veze	Nenhuma_nc	Nenhuma_nc
Muito_Ruim	Nenhuma_nc	Nenhuma_nc	Um_problem	Nao	Nao_se_aplic	Nao_se_aplic	Menos_que_
Boa	Nenhuma_nc	Nenhuma_nc	Nenhuma_di	Parceiro_na_	Nenhuma_nc	Nenhuma_nc	Nenhuma_nc
Ruim	Nenhuma_nc	1_ou_2_veze	Um_problem	Nao	Nao_se_aplic	Nao_se_aplic	Nao_se_aplic
Boa	Nenhuma_nc	Nenhuma_nc	Um_problem	Parceiro_na_	Nenhuma_nc	Nenhuma_nc	Nenhuma_nc
Boa	Nenhuma_nc	Nenhuma_nc	Um_problem	Parceiro_ou_	Nenhuma_nc	Nenhuma_nc	Nenhuma_nc
Muito_boa	Nenhuma_nc	Nenhuma_nc	Um_problem	Parceiro_na_	1_ou_2_veze	Nenhuma_nc	Nenhuma_nc
Ruim	Nenhuma_nc	1_ou_2_veze	Um_problem	Nao	3_ou_mais_\	3_ou_mais_\	1_ou_2_veze
Ruim	Nenhuma_nc	Nenhuma_nc	Um_problem	Nao	Nao_se_aplic	Nenhuma_nc	Menos_que_
Ruim	Nenhuma_nc	Nenhuma_nc	Um_problem	Parceiro_na_	Nenhuma_nc	Nenhuma_nc	Nenhuma_nc
Ruim	Nenhuma_nc	Menos_de_1	Um_problem	Parceiro_na_	1_ou_2_veze	Nenhuma_nc	Nenhuma_nc
Boa	Nenhuma_nc	Nenhuma_nc	Um_problem	Parceiro_na_	Nenhuma_nc	Nenhuma_nc	Nenhuma_nc
Boa	Nenhuma_nc	Nenhuma_nc	Nenhuma_di	Nao	Nao_se_aplic	Nao_se_aplic	Nao_se_aplic
Boa	Menos_de_1	Nenhuma_nc	Um_problem	Parceiro_na_	Menos_que_	Menos_que_	Nenhuma_nc
Ruim	Nenhuma_nc	Menos_de_1	Um_grande_	Parceiro_na_	Nenhuma_nc	Nenhuma_nc	1_ou_2_veze
Ruim	Menos_de_1	Menos_de_1	Um_problem	Parceiro_na_	1_ou_2_veze	Nenhuma_nc	Nenhuma_nc
Boa	Nenhuma_nc	Nenhuma_nc	Um_problem	Nao	Nao_se_aplic	Nao_se_aplic	Nao_se_aplic
Boa	Nenhuma_nc	Nenhuma_nc	Um_problem	Parceiro_na_	1_ou_2_veze	Nenhuma_nc	Nenhuma_nc
Boa	Nenhuma_nc	Nenhuma_nc	Um_problem	Parceiro_na_	3_ou_mais_\	Nenhuma_nc	Nenhuma_nc
Boa	Menos_de_1	1_ou_2_veze	Um_problem	Parceiro_na_	Nenhuma_nc	Nenhuma_nc	Menos_que_
Muito_Ruim	Nenhuma_nc	Nenhuma_nc	Um_problem	Parceiro_na_	3_ou_mais_\	Nenhuma_nc	Nenhuma_nc

Muito_Ruim	Nenhuma_nc	1_ou_2_veze	Nenhuma_di	Parceiro_na_	3_ou_mais_\	3_ou_mais_\	Nenhuma_nc
Ruim	Nenhuma_nc	Nenhuma_nc	Um_problem	Parceiro_na_	Menos_que_	Nenhuma_nc	Nenhuma_nc
Boa	Nenhuma_nc	Nenhuma_nc	Um_problem	Nao	Nao_se_aplic	Nao_se_aplic	Nao_se_aplic
Muito_boa	Nenhuma_nc	Nenhuma_nc	Um_problem	Parceiro_na_	Nenhuma_nc	Nenhuma_nc	Nenhuma_nc
Boa	Nenhuma_nc	Nenhuma_nc	Nenhuma_di	Parceiro_na_	Nenhuma_nc	Nenhuma_nc	Menos_que_
Ruim	Nenhuma_nc	Nenhuma_nc	Um_grande_	Parceiro_na_	Menos_que_	Nenhuma_nc	Nenhuma_nc
Ruim	Nenhuma_nc	Menos_de_1	Um_problem	Parceiro_na_	Menos_que_	Nenhuma_nc	Menos_que_
Boa	Nenhuma_nc	Nenhuma_nc	Um_problem	Parceiro_na_	Nenhuma_nc	Nenhuma_nc	1_ou_2_veze
Boa	Nenhuma_nc	Nenhuma_nc	Um_problem	Nao	Nao_se_aplic	Nao_se_aplic	Nao_se_aplic
Boa	Nenhuma_nc	Menos_de_1	Um_problem	Parceiro_na_	Nenhuma_nc	Nenhuma_nc	Nenhuma_nc
Boa	Nenhuma_nc	Nenhuma_nc	Nenhuma_di	Parceiro_na_	Nenhuma_nc	Nenhuma_nc	Nenhuma_nc
Boa	Nenhuma_nc	Nenhuma_nc	Um_problem	Parceiro_na_	3_ou_mais_\	3_ou_mais_\	Nenhuma_nc
Boa	Nenhuma_nc	Nenhuma_nc	Nenhuma_di	Nao	Nao_se_aplic	Nao_se_aplic	Nao_se_aplic
Boa	Nenhuma_nc	Nenhuma_nc	Nenhuma_di	Parceiro_na_	3_ou_mais_\	Nenhuma_nc	Nenhuma_nc
Muito_boa	Nenhuma_nc	Menos_de_1	Um_grande_	Parceiro_na_	Nenhuma_nc	Nenhuma_nc	3_ou_mais_\
Ruim	3_ou_mais_\	Nenhuma_nc	Um_grande_	Nao	Nao_se_aplic	Nao_se_aplic	Nao_se_aplic
Boa	Nenhuma_nc	Nenhuma_nc	Um_problem	Parceiro_na_	Nenhuma_nc	Nenhuma_nc	Nenhuma_nc
Boa	Nenhuma_nc	Nenhuma_nc	Nenhuma_di	Parceiro_na_	Nenhuma_nc	Nenhuma_nc	Nao_se_aplic
Boa	Nenhuma_nc	Menos_de_1	Um_problem	Nao	Nao_se_aplic	Nao_se_aplic	Nao_se_aplic
Ruim	3_ou_mais_\	Nenhuma_nc	Um_grande_	Parceiro_na_	3_ou_mais_\	Menos_que_	Nenhuma_nc
Ruim	Menos_de_1	3_ou_mais_\	Um_grande_	Parceiro_na_	1_ou_2_veze	Nenhuma_nc	Nenhuma_nc
Ruim	Menos_de_1	1_ou_2_veze	Um_problem	Parceiro_na_	3_ou_mais_\	1_ou_2_veze	1_ou_2_veze
Ruim	Menos_de_1	Nenhuma_nc	Um_problem	Parceiro_na_	Menos_que_	Nenhuma_nc	Menos_que_
Muito_Ruim	3_ou_mais_\	1_ou_2_veze	Um_grande_	Nao	Nao_se_aplic	Nao_se_aplic	Nao_se_aplic
Ruim	3_ou_mais_\	Nenhuma_nc	Um_problem	Nao	Nao_se_aplic	Nao_se_aplic	Nao_se_aplic
Boa	Nenhuma_nc	Nenhuma_nc	Um_problem	Nao	Nenhuma_nc	Nao_se_aplic	Nenhuma_nc
Boa	Nenhuma_nc	Nenhuma_nc	Nenhuma_di	Parceiro_na_	3_ou_mais_\	3_ou_mais_\	Nenhuma_nc
Boa	Nenhuma_nc	Nenhuma_nc	Nenhuma_di	Nao	Nao_se_aplic	Nao_se_aplic	Nao_se_aplic
Muito_boa	3_ou_mais_\	Nenhuma_nc	Um_problem	Parceiro_na_	Menos_que_	Nenhuma_nc	Nenhuma_nc
Boa	Nenhuma_nc	Menos_de_1	Um_problem	Parceiro_na_	3_ou_mais_\	1_ou_2_veze	Menos_que_
Boa	Nenhuma_nc	Menos_de_1	Nenhuma_di	Parceiro_na_	Nenhuma_nc	Nenhuma_nc	1_ou_2_veze
Muito_boa	Nenhuma_nc	Menos_de_1	Um_problem	Parceiro_na_	1_ou_2_veze	Nenhuma_nc	Menos_que_
Muito_boa	Nenhuma_nc	Nenhuma_nc	Nenhuma_di	Parceiro_na_	1_ou_2_veze	Nenhuma_nc	Nenhuma_nc
Boa	Nenhuma_nc	Nenhuma_nc	Nenhuma_di	Parceiro_na_	Nenhuma_nc	Nenhuma_nc	Nenhuma_nc
Boa	Nenhuma_nc	Nenhuma_nc	Um_problem	Parceiro_na_	1_ou_2_veze	Menos_que_	Nenhuma_nc
Boa	Nenhuma_nc	Nenhuma_nc	Um_problem	Parceiro_na_	Nenhuma_nc	Nenhuma_nc	Nenhuma_nc
Ruim	Nenhuma_nc	Menos_de_1	Um_problem	Parceiro_na_	Nenhuma_nc	Nenhuma_nc	Nenhuma_nc
Ruim	Menos_de_1	Nenhuma_nc	Um_problem	Parceiro_na_	Nenhuma_nc	Nenhuma_nc	Nenhuma_nc
Boa	Nenhuma_nc	Nenhuma_nc	Um_problem	Nao	Nao_se_aplic	Nao_se_aplic	Nao_se_aplic
Ruim	Nenhuma_nc	Nenhuma_nc	Um_problem	Nao	Nao_se_aplic	Nao_se_aplic	Nao_se_aplic
Boa	Nenhuma_nc	Nenhuma_nc	Nenhuma_di	Parceiro_na_	Menos_que_	Nenhuma_nc	Nenhuma_nc

				Generalized_Anxiety_Disorder_GAD-7				
episodios_d	outras_alter	po	qualidade_d	sentir-se_ne	nao_ser_ca	preocupar-s	dificuldade_	ficar_tao_ag
Nenhuma_nc	NULL	9	ruim	Quase_todos	Quase_todos	Quase_todos	Mais_da_me	Nenhuma_ve
Nao_se_aplic	NULL	8	ruim	Varios_dias	Varios_dias	Varios_dias	Varios_dias	Nenhuma_ve
Nenhuma_nc	NULL	6	ruim	Quase_todos	Mais_da_me	Quase_todos	Quase_todos	Varios_dias
Nao_se_aplic	NULL	16	presenca_de	Varios_dias	Varios_dias	Varios_dias	Mais_da_me	Nenhuma_ve
Nenhuma_nc	NULL	6	ruim	Varios_dias	Varios_dias	Mais_da_me	Varios_dias	Mais_da_me
Nao_se_aplic	NULL	6	ruim	Varios_dias	Varios_dias	Varios_dias	Varios_dias	Varios_dias
Nao_se_aplic	NULL	8	ruim	Varios_dias	Varios_dias	Varios_dias	Varios_dias	Nenhuma_ve
Nao_se_aplic	NULL	7	ruim	Varios_dias	Varios_dias	Varios_dias	Varios_dias	Nenhuma_ve
Nenhuma_nc	Falar_enqua	10	ruim	Varios_dias	Varios_dias	Varios_dias	Varios_dias	Nenhuma_ve
Nao_se_aplic	NULL	18	presenca_de	Varios_dias	Varios_dias	Mais_da_me	Quase_todos	Nenhuma_ve
Nao_se_aplic	NULL	13	presenca_de	Quase_todos	Mais_da_me	Mais_da_me	Mais_da_me	Quase_todos
Nenhuma_nc	NULL	8	ruim	Varios_dias	Varios_dias	Varios_dias	Varios_dias	Nenhuma_ve
Nenhuma_nc	NULL	5	ruim	Varios_dias	Varios_dias	Varios_dias	Mais_da_me	Nenhuma_ve
Nao_se_aplic	NULL	7	ruim	Varios_dias	Nenhuma_ve	Varios_dias	Varios_dias	Nenhuma_ve
Nao_se_aplic	Paralisia_do_	11	presenca_de	Quase_todos	Quase_todos	Quase_todos	Quase_todos	Mais_da_me
Nao_se_aplic	NULL	6	ruim	Mais_da_me	Varios_dias	Varios_dias	Varios_dias	Nenhuma_ve
Nenhuma_nc	Acordo_frequ	6	ruim	Varios_dias	Varios_dias	Varios_dias	Varios_dias	Nenhuma_ve
Nenhuma_nc	NULL	5	ruim	Quase_todos	Quase_todos	Quase_todos	Mais_da_me	Varios_dias
Nao_se_aplic	NULL	7	ruim	Varios_dias	Varios_dias	Varios_dias	Varios_dias	Varios_dias
Nenhuma_nc	NULL	0	boa	Nenhuma_ve	Nenhuma_ve	Nenhuma_ve	Nenhuma_ve	Nenhuma_ve
Nenhuma_nc	NULL	6	ruim	Varios_dias	Varios_dias	Varios_dias	Varios_dias	Nenhuma_ve
Nao_se_aplic	Desde_crian	8	ruim	Varios_dias	Varios_dias	Quase_todos	Varios_dias	Mais_da_me
Menos_que_	NULL	6	ruim	Varios_dias	Varios_dias	Varios_dias	Nenhuma_ve	Nenhuma_ve
Nenhuma_nc	Neste_ultimo	5	ruim	Quase_todos	Mais_da_me	Quase_todos	Mais_da_me	Nenhuma_ve
1_ou_2_veze	NULL	18	presenca_de	Quase_todos	Quase_todos	Quase_todos	Quase_todos	Quase_todos
Nao_se_aplic	NULL	6	ruim	Varios_dias	Varios_dias	Mais_da_me	Varios_dias	Varios_dias
Nenhuma_nc	NULL	11	presenca_de	Mais_da_me	Mais_da_me	Quase_todos	Mais_da_me	Quase_todos
Nenhuma_nc	NULL	12	presenca_de	Quase_todos	Varios_dias	Quase_todos	Quase_todos	Varios_dias
Nenhuma_nc	NULL	9	ruim	Varios_dias	Mais_da_me	Quase_todos	Mais_da_me	Nenhuma_ve
Nenhuma_nc	NULL	9	ruim	Varios_dias	Mais_da_me	Mais_da_me	Varios_dias	Nenhuma_ve
Nenhuma_nc	NULL	6	ruim	Mais_da_me	Varios_dias	Mais_da_me	Mais_da_me	Varios_dias
Nao_se_aplic	NULL	8	ruim	Varios_dias	Mais_da_me	Quase_todos	Quase_todos	Mais_da_me
Nao_se_aplic	NULL	7	ruim	Mais_da_me	Varios_dias	Mais_da_me	Varios_dias	Nenhuma_ve
Nao_se_aplic	NULL	8	ruim	Quase_todos	Quase_todos	Quase_todos	Quase_todos	Mais_da_me
Nenhuma_nc	NULL	5	ruim	Varios_dias	Varios_dias	Varios_dias	Quase_todos	Nenhuma_ve
Menos_que_	NULL	5	ruim	Varios_dias	Nenhuma_ve	Varios_dias	Nenhuma_ve	Nenhuma_ve
1_ou_2_veze	NULL	13	presenca_de	Mais_da_me	Mais_da_me	Mais_da_me	Quase_todos	Varios_dias
Nao_se_aplic	NULL	10	ruim	Quase_todos	Quase_todos	Quase_todos	Quase_todos	Varios_dias
Nao_se_aplic	NULL	7	ruim	Quase_todos	Quase_todos	Quase_todos	Quase_todos	Varios_dias
Nao_se_aplic	NULL	8	ruim	Varios_dias	Varios_dias	Varios_dias	Varios_dias	Nenhuma_ve
Nenhuma_nc	NULL	8	ruim	Mais_da_me	Varios_dias	Varios_dias	Varios_dias	Mais_da_me
Nao_se_aplic	NULL	6	ruim	Varios_dias	Varios_dias	Varios_dias	Nenhuma_ve	Nenhuma_ve
Nenhuma_nc	NULL	6	ruim	Quase_todos	Quase_todos	Quase_todos	Quase_todos	Mais_da_me
Menos_que_	NULL	8	ruim	Varios_dias	Mais_da_me	Mais_da_me	Varios_dias	Nenhuma_ve
1_ou_2_veze	Me_mexo_m	12	presenca_de	Varios_dias	Varios_dias	Varios_dias	Varios_dias	Nenhuma_ve
Nenhuma_nc	NULL	7	ruim	Mais_da_me	Quase_todos	Quase_todos	Quase_todos	Quase_todos
Nao_se_aplic	NULL	7	ruim	Varios_dias	Varios_dias	Varios_dias	Varios_dias	Nenhuma_ve
Nao_se_aplic	NULL	9	ruim	Varios_dias	Varios_dias	Varios_dias	Varios_dias	Nenhuma_ve
Nao_se_aplic	NULL	9	ruim	Varios_dias	Nenhuma_ve	Varios_dias	Nenhuma_ve	Nenhuma_ve
Nao_se_aplic	NULL	10	ruim	Mais_da_me	Mais_da_me	Quase_todos	Mais_da_me	Mais_da_me
Nao_se_aplic	NULL	8	ruim	Varios_dias	Varios_dias	Quase_todos	Varios_dias	Mais_da_me
Nao_se_aplic	NULL	6	ruim	Varios_dias	Nenhuma_ve	Varios_dias	Varios_dias	Varios_dias
Menos_que_	NULL	16	presenca_de	Quase_todos	Mais_da_me	Varios_dias	Mais_da_me	Varios_dias
1_ou_2_veze	NULL	8	ruim	Quase_todos	Quase_todos	Quase_todos	Quase_todos	Varios_dias
Nao_se_aplic	NULL	6	ruim	Varios_dias	Varios_dias	Varios_dias	Varios_dias	Mais_da_me

Nenhuma_no_ultimo_mes		12	presenca_de	Quase_todos	Varios_dias	Quase_todos	Mais_da_me	Varios_dias
Nao_se_apli	NULL	9	ruim	Quase_todos	Varios_dias	Mais_da_me	Mais_da_me	Varios_dias
Nenhuma_no	Senti_a_boca	11	presenca_de	Quase_todos	Mais_da_me	Quase_todos	Quase_todos	Varios_dias
Nao_se_apli	NULL	10	ruim	Quase_todos	Varios_dias	Varios_dias	Varios_dias	Varios_dias
Nao_se_apli	NULL	7	ruim	Varios_dias	Varios_dias	Varios_dias	Varios_dias	Varios_dias
Nenhuma_no	NULL	4	boa	Nenhuma_ve	Varios_dias	Nenhuma_ve	Nenhuma_ve	Nenhuma_ve
Nao_se_apli	NULL	8	ruim	Varios_dias	Varios_dias	Varios_dias	Varios_dias	Nenhuma_ve
3_ou_mais_v	NULL	2	boa	Nenhuma_ve	Nenhuma_ve	Varios_dias	Nenhuma_ve	Quase_todos
Nao_se_apli	NULL	8	ruim	Varios_dias	Varios_dias	Varios_dias	Mais_da_me	Nenhuma_ve
Nenhuma_no	NULL	2	boa	Varios_dias	Varios_dias	Quase_todos	Nenhuma_ve	Varios_dias
Menos_que_	NULL	3	boa	Varios_dias	Varios_dias	Varios_dias	Varios_dias	Nenhuma_ve
Menos_que_	NULL	9	ruim	Quase_todos	Mais_da_me	Quase_todos	Quase_todos	Varios_dias
Menos_que_	NULL	3	boa	Mais_da_me	Mais_da_me	Quase_todos	Varios_dias	Nenhuma_ve
Nenhuma_no	NULL	5	ruim	Mais_da_me	Mais_da_me	Mais_da_me	Mais_da_me	Varios_dias
Nenhuma_no	Nenhuma_	1	boa	Varios_dias	Nenhuma_ve	Varios_dias	Varios_dias	Nenhuma_ve
Nao_se_apli	NULL	4	boa	Nenhuma_ve	Varios_dias	Nenhuma_ve	Nenhuma_ve	Nenhuma_ve
Nenhuma_no	Perna_dobra	8	ruim	Varios_dias	Varios_dias	Quase_todos	Varios_dias	Nenhuma_ve
Nenhuma_no	NULL	3	boa	Varios_dias	Varios_dias	Mais_da_me	Varios_dias	Nenhuma_ve
Nao_se_apli	NULL	9	ruim	Varios_dias	Nenhuma_ve	Varios_dias	Varios_dias	Nenhuma_ve
Nenhuma_no	NULL	12	presenca_de	Varios_dias	Quase_todos	Quase_todos	Mais_da_me	Nenhuma_ve
Nenhuma_no	NULL	8	ruim	Varios_dias	Varios_dias	Quase_todos	Quase_todos	Nenhuma_ve
Nenhuma_no	NULL	3	boa	Nenhuma_ve	Nenhuma_ve	Nenhuma_ve	Nenhuma_ve	Nenhuma_ve
1_ou_2_veze	Eu_costumo_	3	boa	Nenhuma_ve	Nenhuma_ve	Nenhuma_ve	Nenhuma_ve	Nenhuma_ve
Nenhuma_no	NULL	5	ruim	Quase_todos	Mais_da_me	Quase_todos	Quase_todos	Varios_dias
Nao_se_apli	NULL	14	presenca_de	Quase_todos	Mais_da_me	Quase_todos	Varios_dias	Nenhuma_ve
Nao_se_apli	NULL	7	ruim	Varios_dias	Varios_dias	Quase_todos	Varios_dias	Nenhuma_ve
Nenhuma_no	NULL	4	boa	Nenhuma_ve	Nenhuma_ve	Nenhuma_ve	Nenhuma_ve	Nenhuma_ve
Nao_se_apli	NULL	8	ruim	Quase_todos	Quase_todos	Quase_todos	Mais_da_me	Quase_todos
Nenhuma_no	NULL	8	ruim	Varios_dias	Varios_dias	Varios_dias	Mais_da_me	Nenhuma_ve
Nao_se_apli	NULL	9	ruim	Quase_todos	Mais_da_me	Quase_todos	Mais_da_me	Nenhuma_ve
Menos_que_	NULL	11	presenca_de	Quase_todos	Quase_todos	Quase_todos	Quase_todos	Mais_da_me
Nenhuma_no	NULL	1	boa	Varios_dias	Nenhuma_ve	Varios_dias	Nenhuma_ve	Nenhuma_ve
Nao_se_apli	NULL	6	ruim	Varios_dias	Nenhuma_ve	Varios_dias	Varios_dias	Nenhuma_ve
Nao_se_apli	NULL	4	boa	Varios_dias	Varios_dias	Varios_dias	Varios_dias	Varios_dias
Nenhuma_no	NULL	6	ruim	Nenhuma_ve	Nenhuma_ve	Nenhuma_ve	Varios_dias	Nenhuma_ve
Menos_que_	NULL	5	ruim	Quase_todos	Quase_todos	Quase_todos	Quase_todos	Nenhuma_ve
Nao_se_apli	NULL	8	ruim	Mais_da_me	Mais_da_me	Mais_da_me	Quase_todos	Mais_da_me
Nao_se_apli	NULL	5	ruim	Varios_dias	Varios_dias	Varios_dias	Varios_dias	Varios_dias
Nao_se_apli	NULL	17	presenca_de	Quase_todos	Mais_da_me	Quase_todos	Quase_todos	Varios_dias
Nao_se_apli	NULL	7	ruim	Varios_dias	Varios_dias	Varios_dias	Varios_dias	Nenhuma_ve
Nenhuma_no	NULL	12	presenca_de	Mais_da_me	Mais_da_me	Quase_todos	Quase_todos	Mais_da_me
Nao_se_apli	NULL	7	ruim	Varios_dias	Mais_da_me	Quase_todos	Varios_dias	Quase_todos
Nao_se_apli	NULL	12	presenca_de	Quase_todos	Mais_da_me	Quase_todos	Mais_da_me	Nenhuma_ve
Nenhuma_no	NULL	6	ruim	Varios_dias	Varios_dias	Mais_da_me	Mais_da_me	Mais_da_me
Menos_que_	NULL	14	presenca_de	Varios_dias	Nenhuma_ve	Varios_dias	Varios_dias	Varios_dias
Nao_se_apli	NULL	6	ruim	Quase_todos	Quase_todos	Quase_todos	Mais_da_me	Varios_dias
Nenhuma_no	NULL	6	ruim	Varios_dias	Varios_dias	Varios_dias	Varios_dias	Mais_da_me
Nenhuma_no	NULL	3	boa	Varios_dias	Nenhuma_ve	Nenhuma_ve	Nenhuma_ve	Varios_dias
Nao_se_apli	NULL	5	ruim	Mais_da_me	Mais_da_me	Quase_todos	Mais_da_me	Nenhuma_ve
Nenhuma_no	NULL	9	ruim	Quase_todos	Mais_da_me	Mais_da_me	Quase_todos	Mais_da_me
Nenhuma_no	NULL	8	ruim	Varios_dias	Mais_da_me	Varios_dias	Quase_todos	Nenhuma_ve
Nao_se_apli	NULL	7	ruim	Quase_todos	Quase_todos	Quase_todos	Quase_todos	Quase_todos
Menos_que_	NULL	11	presenca_de	Quase_todos	Quase_todos	Quase_todos	Quase_todos	Quase_todos
Nao_se_apli	NULL	8	ruim	Varios_dias	Varios_dias	Varios_dias	Varios_dias	Nenhuma_ve
1_ou_2_veze	NULL	15	presenca_de	Quase_todos	Quase_todos	Quase_todos	Varios_dias	Mais_da_me
Nenhuma_no	NULL	11	presenca_de	Quase_todos	Quase_todos	Quase_todos	Quase_todos	Quase_todos
Nenhuma_no	NULL	16	presenca_de	Quase_todos	Quase_todos	Quase_todos	Quase_todos	Quase_todos
Nenhuma_no	NULL	6	ruim	Varios_dias	Varios_dias	Varios_dias	Nenhuma_ve	Mais_da_me

Nenhuma_nc	NULL	8	ruim	Quase_todos	Quase_todos	Mais_da_me	Quase_todos	Quase_todos
Nao_se_aplic	NULL	12	presenca_de	Varios_dias	Varios_dias	Mais_da_me	Quase_todos	Varios_dias
Nao_se_aplic	NULL	13	presenca_de	Quase_todos	Quase_todos	Quase_todos	Quase_todos	Varios_dias
Nao_se_aplic	NULL	6	ruim	Mais_da_me	Mais_da_me	Mais_da_me	Quase_todos	Mais_da_me
Nenhuma_nc	NULL	1	boa	Nenhuma_ve	Nenhuma_ve	Nenhuma_ve	Nenhuma_ve	Quase_todos
Nenhuma_nc	NULL	5	ruim	Varios_dias	Varios_dias	Varios_dias	Varios_dias	Nenhuma_ve
Nao_se_aplic	NULL	4	boa	Varios_dias	Nenhuma_ve	Nenhuma_ve	Nenhuma_ve	Nenhuma_ve
Nao_se_aplic	NULL	9	ruim	Mais_da_me	Varios_dias	Mais_da_me	Mais_da_me	Varios_dias
Nao_se_aplic	NULL	5	ruim	Varios_dias	Varios_dias	Varios_dias	Varios_dias	Nenhuma_ve
Nao_se_aplic	NULL	6	ruim	Mais_da_me	Varios_dias	Varios_dias	Mais_da_me	Varios_dias
Nenhuma_nc	NULL	6	ruim	Varios_dias	Varios_dias	Quase_todos	Varios_dias	Nenhuma_ve
Nenhuma_nc	NULL	5	ruim	Varios_dias	Varios_dias	Varios_dias	Varios_dias	Varios_dias
Nenhuma_nc	Acordei_com	10	ruim	Varios_dias	Varios_dias	Varios_dias	Mais_da_me	Varios_dias
Nenhuma_nc	NULL	8	ruim	Varios_dias	Varios_dias	Mais_da_me	Quase_todos	Varios_dias
Nenhuma_nc	NULL	3	boa	Varios_dias	Nenhuma_ve	Varios_dias	Varios_dias	Nenhuma_ve
Nao_se_aplic	NULL	8	ruim	Quase_todos	Mais_da_me	Mais_da_me	Mais_da_me	Varios_dias
Nenhuma_nc	NULL	5	ruim	Nenhuma_ve	Nenhuma_ve	Varios_dias	Nenhuma_ve	Nenhuma_ve
Nao_se_aplic	NULL	10	ruim	Mais_da_me	Mais_da_me	Varios_dias	Varios_dias	Varios_dias
Menos_que_	NULL	10	ruim	Mais_da_me	Mais_da_me	Mais_da_me	Quase_todos	Varios_dias
3_ou_mais_\	NULL	11	presenca_de	Quase_todos	Quase_todos	Quase_todos	Quase_todos	Quase_todos
Nao_se_aplic	NULL	8	ruim	Varios_dias	Varios_dias	Varios_dias	Quase_todos	Nenhuma_ve
Nenhuma_nc	Nao_houve_	4	boa	Varios_dias	Nenhuma_ve	Varios_dias	Varios_dias	Nenhuma_ve
Nao_se_aplic	NULL	7	ruim	Varios_dias	Nenhuma_ve	Varios_dias	Varios_dias	Nenhuma_ve
Nenhuma_nc	NULL	6	ruim	Quase_todos	Quase_todos	Quase_todos	Mais_da_me	Quase_todos
Nao_se_aplic	NULL	8	ruim	Varios_dias	Varios_dias	Varios_dias	Varios_dias	Varios_dias
Nao_se_aplic	NULL	10	ruim	Quase_todos	Quase_todos	Quase_todos	Quase_todos	Quase_todos
Nenhuma_nc	NULL	9	ruim	Quase_todos	Varios_dias	Quase_todos	Quase_todos	Nenhuma_ve
Nao_se_aplic	NULL	8	ruim	Quase_todos	Quase_todos	Quase_todos	Quase_todos	Nenhuma_ve
Nenhuma_nc	Nenhuma	4	boa	Varios_dias	Nenhuma_ve	Varios_dias	Nenhuma_ve	Nenhuma_ve
1_ou_2_veze	Falar_	7	ruim	Quase_todos	Mais_da_me	Quase_todos	Quase_todos	Mais_da_me
3_ou_mais_\	NULL	15	presenca_de	Mais_da_me	Varios_dias	Mais_da_me	Varios_dias	Varios_dias
Nao_se_aplic	NULL	4	boa	Nenhuma_ve	Nenhuma_ve	Nenhuma_ve	Nenhuma_ve	Varios_dias
Nao_se_aplic	NULL	5	ruim	Mais_da_me	Mais_da_me	Mais_da_me	Mais_da_me	Nenhuma_ve
Nenhuma_nc	NULL	9	ruim	Varios_dias	Varios_dias	Varios_dias	Varios_dias	Nenhuma_ve
Nao_se_aplic	NULL	5	ruim	Nenhuma_ve	Nenhuma_ve	Varios_dias	Nenhuma_ve	Nenhuma_ve
Nao_se_aplic	NULL	5	ruim	Varios_dias	Nenhuma_ve	Nenhuma_ve	Nenhuma_ve	Quase_todos
Nao_se_aplic	NULL	7	ruim	Mais_da_me	Varios_dias	Varios_dias	Varios_dias	Varios_dias
Nenhuma_nc	NULL	6	ruim	Mais_da_me	Mais_da_me	Mais_da_me	Mais_da_me	Nenhuma_ve
Nenhuma_nc	NULL	4	boa	Varios_dias	Varios_dias	Varios_dias	Nenhuma_ve	Varios_dias
Nenhuma_nc	NULL	7	ruim	Varios_dias	Varios_dias	Varios_dias	Varios_dias	Nenhuma_ve
Nenhuma_nc	NULL	2	boa	Varios_dias	Nenhuma_ve	Varios_dias	Varios_dias	Nenhuma_ve
Nenhuma_nc	NULL	6	ruim	Mais_da_me	Mais_da_me	Mais_da_me	Mais_da_me	Nenhuma_ve
Menos_que_	NULL	6	ruim	Varios_dias	Mais_da_me	Quase_todos	Mais_da_me	Mais_da_me
Nenhuma_nc	NULL	5	ruim	Varios_dias	Nenhuma_ve	Nenhuma_ve	Nenhuma_ve	Nenhuma_ve
Nenhuma_nc	NULL	4	boa	Mais_da_me	Varios_dias	Varios_dias	Varios_dias	Varios_dias
Nenhuma_nc	NULL	5	ruim	Mais_da_me	Mais_da_me	Mais_da_me	Mais_da_me	Mais_da_me
Menos_que_	NULL	9	ruim	Mais_da_me	Varios_dias	Mais_da_me	Mais_da_me	Varios_dias
Nao_se_aplic	NULL	13	presenca_de	Quase_todos	Mais_da_me	Quase_todos	Quase_todos	Varios_dias
Menos_que_	NULL	5	ruim	Quase_todos	Quase_todos	Quase_todos	Quase_todos	Quase_todos
Nao_se_aplic	NULL	10	ruim	Quase_todos	Quase_todos	Quase_todos	Mais_da_me	Nenhuma_ve
Nao_se_aplic	NULL	12	presenca_de	Mais_da_me	Varios_dias	Quase_todos	Quase_todos	Quase_todos
Nao_se_aplic	NULL	3	boa	Nenhuma_ve	Varios_dias	Varios_dias	Varios_dias	Nenhuma_ve
Nenhuma_nc	nenhuma	7	ruim	Varios_dias	Nenhuma_ve	Nenhuma_ve	Nenhuma_ve	Nenhuma_ve
Nenhuma_nc	NULL	6	ruim	Varios_dias	Varios_dias	Varios_dias	Varios_dias	Nenhuma_ve
Nenhuma_nc	NULL	14	presenca_de	Varios_dias	Varios_dias	Varios_dias	Varios_dias	Varios_dias
Nao_se_aplic	NULL	14	presenca_de	Quase_todos	Quase_todos	Quase_todos	Quase_todos	Mais_da_me
Nao_se_aplic	NULL	9	ruim	Varios_dias	Nenhuma_ve	Varios_dias	Varios_dias	Nenhuma_ve
Nenhuma_nc	NULL	4	boa	Mais_da_me	Varios_dias	Mais_da_me	Varios_dias	Varios_dias

Nenhuma_nc	NULL	3	boa	Varios_dias	Varios_dias	Varios_dias	Varios_dias	Nenhuma_ve
Menos_que_	NULL	4	boa	Nenhuma_ve	Varios_dias	Varios_dias	Varios_dias	Varios_dias
Nenhuma_nc	NULL	9	ruim	Varios_dias	Varios_dias	Mais_da_me	Varios_dias	Nenhuma_ve
3_ou_mais_\	NULL	4	boa	Varios_dias	Varios_dias	Nenhuma_ve	Varios_dias	Nenhuma_ve
Nenhuma_nc	NULL	6	ruim	Varios_dias	Varios_dias	Varios_dias	Varios_dias	Nenhuma_ve
Nenhuma_nc	90_microdes	14	presenca_de	Quase_todos	Mais_da_me	Varios_dias	Mais_da_me	Varios_dias
Nenhuma_nc	Sempre_tive_	5	ruim	Varios_dias	Nenhuma_ve	Nenhuma_ve	Nenhuma_ve	Nenhuma_ve
Nao_se_aplic	NULL	1	boa	Varios_dias	Nenhuma_ve	Nenhuma_ve	Nenhuma_ve	Nenhuma_ve
1_ou_2_veze	NULL	5	ruim	Varios_dias	Varios_dias	Varios_dias	Varios_dias	Varios_dias
Nenhuma_nc	NULL	11	presenca_de	Quase_todos	Mais_da_me	Quase_todos	Quase_todos	Nenhuma_ve
3_ou_mais_\	NULL	15	presenca_de	Quase_todos	Quase_todos	Quase_todos	Quase_todos	Quase_todos
Nenhuma_nc	NULL	7	ruim	Varios_dias	Varios_dias	Varios_dias	Varios_dias	Nenhuma_ve
Nenhuma_nc	NULL	4	boa	Mais_da_me	Quase_todos	Quase_todos	Varios_dias	Varios_dias
Nenhuma_nc	NULL	6	ruim	Nenhuma_ve	Nenhuma_ve	Varios_dias	Varios_dias	Nenhuma_ve
Menos_que_	NULL	5	ruim	Varios_dias	Varios_dias	Varios_dias	Varios_dias	Nenhuma_ve
Menos_que_	NULL	6	ruim	Varios_dias	Varios_dias	Varios_dias	Varios_dias	Varios_dias
Nenhuma_nc	NULL	11	presenca_de	Quase_todos	Varios_dias	Mais_da_me	Quase_todos	Mais_da_me
Nenhuma_nc	NULL	3	boa	Varios_dias	Varios_dias	Varios_dias	Varios_dias	Nenhuma_ve
Nenhuma_nc	NULL	9	ruim	Quase_todos	Quase_todos	Quase_todos	Mais_da_me	Varios_dias
Nenhuma_nc	Gatos_com_	7	ruim	Nenhuma_ve	Varios_dias	Varios_dias	Varios_dias	Nenhuma_ve
Nao_se_aplic	NULL	8	ruim	Mais_da_me	Mais_da_me	Mais_da_me	Mais_da_me	Nenhuma_ve
Nenhuma_nc	NULL	4	boa	Mais_da_me	Nenhuma_ve	Mais_da_me	Nenhuma_ve	Nenhuma_ve
Nao_se_aplic	As_minhas_(	10	ruim	Quase_todos	Quase_todos	Quase_todos	Quase_todos	Varios_dias
Nenhuma_nc	NULL	0	boa	Nenhuma_ve	Nenhuma_ve	Varios_dias	Nenhuma_ve	Nenhuma_ve
Nao_se_aplic	NULL	6	ruim	Varios_dias	Varios_dias	Varios_dias	Varios_dias	Nenhuma_ve
Nenhuma_nc	NULL	12	presenca_de	Quase_todos	Varios_dias	Varios_dias	Mais_da_me	Varios_dias
Nenhuma_nc	NULL	8	ruim	Varios_dias	Varios_dias	Varios_dias	Varios_dias	Nenhuma_ve
Nenhuma_nc	NULL	10	ruim	Nenhuma_ve	Nenhuma_ve	Nenhuma_ve	Varios_dias	Varios_dias
Nenhuma_nc	NULL	7	ruim	Varios_dias	Nenhuma_ve	Varios_dias	Nenhuma_ve	Nenhuma_ve
1_ou_2_veze	As_vezes_te	9	ruim	Varios_dias	Mais_da_me	Mais_da_me	Quase_todos	Nenhuma_ve
Nao_se_aplic	NULL	10	ruim	Varios_dias	Varios_dias	Varios_dias	Varios_dias	Nenhuma_ve
Nao_se_aplic	NULL	5	ruim	Nenhuma_ve	Nenhuma_ve	Nenhuma_ve	Nenhuma_ve	Nenhuma_ve
Nenhuma_nc	NULL	13	presenca_de	Quase_todos	Quase_todos	Quase_todos	Quase_todos	Quase_todos
Nenhuma_nc	NULL	4	boa	Varios_dias	Varios_dias	Varios_dias	Varios_dias	Varios_dias
Menos_que_	Tonturas	12	presenca_de	Quase_todos	Varios_dias	Varios_dias	Varios_dias	Varios_dias
Nao_se_aplic	Preocupacoe	11	presenca_de	Quase_todos	Quase_todos	Quase_todos	Quase_todos	Varios_dias
Nenhuma_nc	NULL	16	presenca_de	Quase_todos	Quase_todos	Quase_todos	Quase_todos	Nenhuma_ve
Nenhuma_nc	NULL	3	boa	Varios_dias	Varios_dias	Varios_dias	Nenhuma_ve	Nenhuma_ve
Nao_se_aplic	NULL	9	ruim	Mais_da_me	Mais_da_me	Mais_da_me	Mais_da_me	Varios_dias
Nenhuma_nc	NULL	5	ruim	Mais_da_me	Nenhuma_ve	Varios_dias	Varios_dias	Varios_dias
Nenhuma_nc	NULL	10	ruim	Varios_dias	Varios_dias	Varios_dias	Varios_dias	Nenhuma_ve
Nenhuma_nc	NULL	6	ruim	Varios_dias	Varios_dias	Varios_dias	Varios_dias	Nenhuma_ve
Nenhuma_nc	NULL	6	ruim	Quase_todos	Quase_todos	Quase_todos	Quase_todos	Nenhuma_ve
Nenhuma_nc	NULL	5	ruim	Varios_dias	Varios_dias	Varios_dias	Varios_dias	Varios_dias
Nao_se_aplic	NULL	4	boa	Varios_dias	Nenhuma_ve	Nenhuma_ve	Nenhuma_ve	Nenhuma_ve
Nao_se_aplic	Terror_noturi	13	presenca_de	Quase_todos	Quase_todos	Quase_todos	Quase_todos	Quase_todos
Nenhuma_nc	NULL	8	ruim	Mais_da_me	Mais_da_me	Quase_todos	Varios_dias	Varios_dias
Nenhuma_nc	NULL	11	presenca_de	Varios_dias	Varios_dias	Varios_dias	Varios_dias	Varios_dias
Nao_se_aplic	Tenho_eu_tio	7	ruim	Varios_dias	Varios_dias	Varios_dias	Varios_dias	Varios_dias
Nenhuma_nc	NULL	8	ruim	Varios_dias	Varios_dias	Varios_dias	Varios_dias	Nenhuma_ve
Nenhuma_nc	NULL	6	ruim	Varios_dias	Varios_dias	Varios_dias	Varios_dias	Varios_dias
Nao_se_aplic	NULL	18	presenca_de	Mais_da_me	Varios_dias	Quase_todos	Mais_da_me	Nenhuma_ve
Nao_se_aplic	NULL	11	presenca_de	Quase_todos	Quase_todos	Quase_todos	Quase_todos	Mais_da_me
Nao_se_aplic	Attualmente_	9	ruim	Varios_dias	Nenhuma_ve	Varios_dias	Varios_dias	Nenhuma_ve
Nenhuma_nc	NULL	8	ruim	Quase_todos	Quase_todos	Quase_todos	Quase_todos	Quase_todos
Nenhuma_nc	NULL	5	ruim	Quase_todos	Mais_da_me	Quase_todos	Quase_todos	Nenhuma_ve
Menos_que_	NULL	6	ruim	Varios_dias	Mais_da_me	Quase_todos	Mais_da_me	Varios_dias
Nenhuma_nc	NULL	6	ruim	Quase_todos	Mais_da_me	Quase_todos	Quase_todos	Mais_da_me

Nenhuma_nc	NULL	9 ruim	Quase_todos	Quase_todos	Quase_todos	Quase_todos	Varios_dias
Nenhuma_nc	NULL	14 presenca_de	Quase_todos	Quase_todos	Quase_todos	Mais_da_me	Varios_dias
Nenhuma_nc	NULL	13 presenca_de	Mais_da_me	Varios_dias	Mais_da_me	Varios_dias	Nenhuma_ve
Menos_que_	NULL	9 ruim	Varios_dias	Varios_dias	Varios_dias	Varios_dias	Varios_dias
1_ou_2_veze	Sonambulisn	5 ruim	Mais_da_me	Varios_dias	Varios_dias	Varios_dias	Nenhuma_ve
1_ou_2_veze	NULL	9 ruim	Varios_dias	Varios_dias	Quase_todos	Mais_da_me	Varios_dias
Menos_que_	NULL	3 boa	Quase_todos	Quase_todos	Quase_todos	Mais_da_me	Nenhuma_ve
Nao_se_aplic	NULL	4 boa	Varios_dias	Varios_dias	Varios_dias	Varios_dias	Varios_dias
Nenhuma_nc	NULL	6 ruim	Quase_todos	Quase_todos	Quase_todos	Mais_da_me	Mais_da_me
Nenhuma_nc	NULL	7 ruim	Mais_da_me	Varios_dias	Quase_todos	Varios_dias	Varios_dias
Nao_se_aplic	NULL	7 ruim	Mais_da_me	Mais_da_me	Mais_da_me	Mais_da_me	Nenhuma_ve
Nenhuma_nc	acordar_varia	14 presenca_de	Quase_todos	Quase_todos	Quase_todos	Quase_todos	Quase_todos
Nenhuma_nc	NULL	4 boa	Varios_dias	Varios_dias	Varios_dias	Varios_dias	Varios_dias
Nao_se_aplic	nada	4 boa	Varios_dias	Varios_dias	Varios_dias	Varios_dias	Nenhuma_ve
Nenhuma_nc	NULL	4 boa	Varios_dias	Nenhuma_ve	Varios_dias	Varios_dias	Nenhuma_ve
Nenhuma_nc	NULL	11 presenca_de	Mais_da_me	Mais_da_me	Mais_da_me	Mais_da_me	Varios_dias
Nenhuma_nc	NULL	11 presenca_de	Varios_dias	Varios_dias	Quase_todos	Mais_da_me	Mais_da_me
Nao_se_aplic	NULL	9 ruim	Quase_todos	Quase_todos	Quase_todos	Quase_todos	Mais_da_me
Menos_que_	vertigem	9 ruim	Quase_todos	Quase_todos	Quase_todos	Quase_todos	Varios_dias
Nenhuma_nc	NULL	11 presenca_de	Quase_todos	Quase_todos	Quase_todos	Quase_todos	Quase_todos
Nenhuma_nc	NULL	7 ruim	Varios_dias	Varios_dias	Varios_dias	Varios_dias	Varios_dias
Nenhuma_nc	NULL	1 boa	Nenhuma_ve	Nenhuma_ve	Nenhuma_ve	Nenhuma_ve	Nenhuma_ve
Nao_se_aplic	NULL	11 presenca_de	Mais_da_me	Mais_da_me	Quase_todos	Quase_todos	Varios_dias
1_ou_2_veze	NULL	20 presenca_de	Quase_todos	Quase_todos	Quase_todos	Quase_todos	Quase_todos
Nenhuma_nc	NULL	10 ruim	Quase_todos	Mais_da_me	Quase_todos	Mais_da_me	Varios_dias
Nao_se_aplic	NULL	12 presenca_de	Mais_da_me	Quase_todos	Quase_todos	Mais_da_me	Varios_dias
Nenhuma_nc	Me_mexo_ba	4 boa	Varios_dias	Varios_dias	Varios_dias	Varios_dias	Nenhuma_ve
Nao_se_aplic	NULL	15 presenca_de	Quase_todos	Quase_todos	Quase_todos	Quase_todos	Quase_todos
Nenhuma_nc	NULL	10 ruim	Quase_todos	Varios_dias	Mais_da_me	Mais_da_me	Nenhuma_ve
Nenhuma_nc	NULL	10 ruim	Quase_todos	Nenhuma_ve	Quase_todos	Nenhuma_ve	Nenhuma_ve
Nenhuma_nc	NULL	9 ruim	Varios_dias	Varios_dias	Varios_dias	Varios_dias	Varios_dias
Nenhuma_nc	Medo-_ansie	9 ruim	Mais_da_me	Varios_dias	Quase_todos	Quase_todos	Varios_dias
Nao_se_aplic	NULL	13 presenca_de	Mais_da_me	Mais_da_me	Varios_dias	Mais_da_me	Varios_dias
Menos_que_	NULL	11 presenca_de	Quase_todos	Mais_da_me	Mais_da_me	Quase_todos	Varios_dias
Nenhuma_nc	Nenhuma_	1 boa	Nenhuma_ve	Nenhuma_ve	Nenhuma_ve	Nenhuma_ve	Nenhuma_ve
Menos_que_	NULL	8 ruim	Varios_dias	Varios_dias	Varios_dias	Varios_dias	Nenhuma_ve
Nao_se_aplic	NULL	6 ruim	Varios_dias	Varios_dias	Varios_dias	Varios_dias	Nenhuma_ve
Nenhuma_nc	NULL	11 presenca_de	Mais_da_me	Varios_dias	Varios_dias	Varios_dias	Nenhuma_ve
Nao_se_aplic	NULL	15 presenca_de	Varios_dias	Varios_dias	Varios_dias	Varios_dias	Varios_dias
Nenhuma_nc	NULL	4 boa	Varios_dias	Varios_dias	Varios_dias	Varios_dias	Nenhuma_ve
Nao_se_aplic	NULL	12 presenca_de	Varios_dias	Varios_dias	Varios_dias	Varios_dias	Nenhuma_ve
Nenhuma_nc	NULL	4 boa	Varios_dias	Nenhuma_ve	Varios_dias	Varios_dias	Varios_dias
Nenhuma_nc	NULL	5 ruim	Varios_dias	Nenhuma_ve	Nenhuma_ve	Nenhuma_ve	Nenhuma_ve
Nenhuma_nc	NULL	3 boa	Varios_dias	Varios_dias	Varios_dias	Varios_dias	Nenhuma_ve
Nenhuma_nc	NULL	7 ruim	Mais_da_me	Mais_da_me	Quase_todos	Quase_todos	Varios_dias
Nenhuma_nc	NULL	7 ruim	Varios_dias	Varios_dias	Varios_dias	Mais_da_me	Varios_dias
Nenhuma_nc	Devido_a_dc	8 ruim	Varios_dias	Nenhuma_ve	Nenhuma_ve	Nenhuma_ve	Nenhuma_ve
Nenhuma_nc	NULL	9 ruim	Varios_dias	Varios_dias	Varios_dias	Varios_dias	Varios_dias
Nenhuma_nc	NULL	5 ruim	Varios_dias	Varios_dias	Varios_dias	Varios_dias	Nenhuma_ve
Nao_se_aplic	NULL	6 ruim	Nenhuma_ve	Nenhuma_ve	Nenhuma_ve	Nenhuma_ve	Nenhuma_ve
Nenhuma_nc	NULL	11 presenca_de	Varios_dias	Nenhuma_ve	Varios_dias	Varios_dias	Nenhuma_ve
Nenhuma_nc	NULL	10 ruim	Quase_todos	Mais_da_me	Quase_todos	Quase_todos	Nenhuma_ve
Menos_que_	NULL	9 ruim	Mais_da_me	Mais_da_me	Mais_da_me	Mais_da_me	Varios_dias
Nao_se_aplic	NULL	5 ruim	Nenhuma_ve	Nenhuma_ve	Nenhuma_ve	Nenhuma_ve	Nenhuma_ve
Menos_que_	NULL	5 ruim	Mais_da_me	Quase_todos	Quase_todos	Quase_todos	Nenhuma_ve
Nenhuma_nc	NULL	5 ruim	Mais_da_me	Mais_da_me	Mais_da_me	Mais_da_me	Varios_dias
Nenhuma_nc	NULL	9 ruim	Varios_dias	Varios_dias	Varios_dias	Varios_dias	Varios_dias
Nenhuma_nc	NULL	9 ruim	Varios_dias	Nenhuma_ve	Varios_dias	Varios_dias	Nenhuma_ve

Nenhuma_nc	NULL	9 ruim	Quase_todos	Quase_todos	Quase_todos	Quase_todos	Nenhuma_ve
Nenhuma_nc	NULL	8 ruim	Varios_dias	Nenhuma_ve	Varios_dias	Varios_dias	Nenhuma_ve
Nao_se_aplic	n-a	7 ruim	Varios_dias	Mais_da_me	Mais_da_me	Mais_da_me	Nenhuma_ve
Nenhuma_nc	NULL	2 boa	Nenhuma_ve	Nenhuma_ve	Varios_dias	Nenhuma_ve	Nenhuma_ve
Nenhuma_nc	NULL	3 boa	Varios_dias	Varios_dias	Varios_dias	Varios_dias	Varios_dias
Nenhuma_nc	Resmungos	8 ruim	Quase_todos	Mais_da_me	Quase_todos	Quase_todos	Varios_dias
Nenhuma_nc	NULL	12 presenca_de	Mais_da_me	Mais_da_me	Mais_da_me	Mais_da_me	Varios_dias
Nenhuma_nc	NULL	5 ruim	Varios_dias	Nenhuma_ve	Mais_da_me	Varios_dias	Nenhuma_ve
Nenhuma_nc	NULL	5 ruim	Varios_dias	Varios_dias	Varios_dias	Varios_dias	Nenhuma_ve
Nenhuma_nc	NULL	8 ruim	Varios_dias	Varios_dias	Varios_dias	Varios_dias	Nenhuma_ve
Nenhuma_nc	Nenhuma_	5 ruim	Varios_dias	Varios_dias	Varios_dias	Varios_dias	Varios_dias
Nenhuma_nc	NULL	7 ruim	Varios_dias	Mais_da_me	Varios_dias	Mais_da_me	Mais_da_me
Nao_se_aplic	NULL	2 boa	Nenhuma_ve	Nenhuma_ve	Nenhuma_ve	Nenhuma_ve	Nenhuma_ve
Nenhuma_nc	SOU_INQUIE	5 ruim	Nenhuma_ve	Nenhuma_ve	Nenhuma_ve	Nenhuma_ve	Nenhuma_ve
Nenhuma_nc	NULL	9 ruim	Varios_dias	Varios_dias	Mais_da_me	Nenhuma_ve	Quase_todos
Nao_se_aplic	NULL	12 presenca_de	Quase_todos	Mais_da_me	Varios_dias	Varios_dias	Nenhuma_ve
Nenhuma_nc	NULL	5 ruim	Varios_dias	Varios_dias	Varios_dias	Varios_dias	Nenhuma_ve
Nao_se_aplic	NULL	3 boa	Nenhuma_ve	Nenhuma_ve	Nenhuma_ve	Nenhuma_ve	Nenhuma_ve
Nao_se_aplic	NULL	4 boa	Quase_todos	Quase_todos	Quase_todos	Quase_todos	Varios_dias
Nao_se_aplic	NULL	16 presenca_de	Quase_todos	Quase_todos	Quase_todos	Quase_todos	Varios_dias
Nenhuma_nc	NULL	13 presenca_de	Mais_da_me	Mais_da_me	Varios_dias	Varios_dias	Varios_dias
1_ou_2_veze	NULL	8 ruim	Quase_todos	Quase_todos	Quase_todos	Quase_todos	Quase_todos
Nenhuma_nc	NULL	10 ruim	Quase_todos	Quase_todos	Quase_todos	Quase_todos	Mais_da_me
Nao_se_aplic	NULL	13 presenca_de	Varios_dias	Nenhuma_ve	Varios_dias	Varios_dias	Varios_dias
Nao_se_aplic	NULL	13 presenca_de	Varios_dias	Varios_dias	Varios_dias	Varios_dias	Varios_dias
Nenhuma_nc	NULL	6 ruim	Varios_dias	Mais_da_me	Mais_da_me	Varios_dias	Varios_dias
Nenhuma_nc	NULL	3 boa	Nenhuma_ve	Nenhuma_ve	Varios_dias	Nenhuma_ve	Nenhuma_ve
Nao_se_aplic	NULL	4 boa	Varios_dias	Nenhuma_ve	Varios_dias	Varios_dias	Nenhuma_ve
Nenhuma_nc	Na_resposta	5 ruim	Mais_da_me	Mais_da_me	Varios_dias	Varios_dias	Varios_dias
Nenhuma_nc	Nenhum_	4 boa	Varios_dias	Varios_dias	Varios_dias	Varios_dias	Varios_dias
Menos_que_	NULL	7 ruim	Varios_dias	Nenhuma_ve	Varios_dias	Varios_dias	Nenhuma_ve
Menos_que_	O_companhe	6 ruim	Varios_dias	Nenhuma_ve	Nenhuma_ve	Nenhuma_ve	Nenhuma_ve
Nenhuma_nc	nao	2 boa	Nenhuma_ve	Nenhuma_ve	Varios_dias	Nenhuma_ve	Nenhuma_ve
Nenhuma_nc	NULL	4 boa	Varios_dias	Nenhuma_ve	Varios_dias	Nenhuma_ve	Nenhuma_ve
Nao_se_aplic	Apago	3 boa	Varios_dias	Nenhuma_ve	Nenhuma_ve	Varios_dias	Varios_dias
Nenhuma_nc	NULL	6 ruim	Varios_dias	Nenhuma_ve	Varios_dias	Nenhuma_ve	Nenhuma_ve
Nenhuma_nc	NULL	12 presenca_de	Quase_todos	Quase_todos	Quase_todos	Quase_todos	Varios_dias
Nenhuma_nc	NULL	8 ruim	Varios_dias	Varios_dias	Varios_dias	Varios_dias	Nenhuma_ve
Nao_se_aplic	NULL	6 ruim	Varios_dias	Varios_dias	Varios_dias	Varios_dias	Varios_dias
Nao_se_aplic	NULL	9 ruim	Varios_dias	Varios_dias	Varios_dias	Varios_dias	Varios_dias
Nenhuma_nc	NULL	3 boa	Nenhuma_ve	Varios_dias	Varios_dias	Nenhuma_ve	Nenhuma_ve

ficar_facilm	sentir_medo	total_GAD-7
Varios_dias	Varios_dias	13
Varios_dias	Quase_todos	8
Quase_todos	Mais_da_me	17
Varios_dias	Nenhuma_ve	6
Varios_dias	Varios_dias	9
Varios_dias	Varios_dias	7
Mais_da_me	Varios_dias	7
Varios_dias	Varios_dias	6
Varios_dias	Varios_dias	6
Quase_todos	Mais_da_me	12
Quase_todos	Varios_dias	16
Nenhuma_ve	Nenhuma_ve	4
Nenhuma_ve	Varios_dias	6
Nenhuma_ve	Nenhuma_ve	3
Mais_da_me	Varios_dias	17
Mais_da_me	Varios_dias	8
Varios_dias	Varios_dias	6
Mais_da_me	Mais_da_me	16
Varios_dias	Varios_dias	7
Nenhuma_ve	Nenhuma_ve	0
Varios_dias	Varios_dias	6
Mais_da_me	Varios_dias	11
Nenhuma_ve	Nenhuma_ve	3
Mais_da_me	Varios_dias	13
Quase_todos	Quase_todos	21
Varios_dias	Varios_dias	8
Varios_dias	Varios_dias	14
Quase_todos	Varios_dias	15
Varios_dias	Quase_todos	12
Varios_dias	Nenhuma_ve	7
Varios_dias	Varios_dias	10
Nenhuma_ve	Nenhuma_ve	11
Nenhuma_ve	Varios_dias	7
Mais_da_me	Varios_dias	17
Varios_dias	Varios_dias	8
Nenhuma_ve	Nenhuma_ve	2
Quase_todos	Mais_da_me	15
Varios_dias	Mais_da_me	16
Quase_todos	Quase_todos	19
Varios_dias	Nenhuma_ve	5
Varios_dias	Varios_dias	9
Nenhuma_ve	Nenhuma_ve	3
Mais_da_me	Quase_todos	19
Varios_dias	Varios_dias	8
Varios_dias	Varios_dias	6
Mais_da_me	Nenhuma_ve	16
Nenhuma_ve	Nenhuma_ve	4
Varios_dias	Varios_dias	6
Nenhuma_ve	Nenhuma_ve	2
Varios_dias	Varios_dias	13
Nenhuma_ve	Nenhuma_ve	8
Varios_dias	Varios_dias	6
Varios_dias	Varios_dias	11
Quase_todos	Quase_todos	19
Varios_dias	Quase_todos	10

Varios_dias	Varios_dias	12
Varios_dias	Varios_dias	11
Mais_da_me	Mais_da_me	16
Nenhuma_ve	Varios_dias	8
Mais_da_me	Varios_dias	8
Varios_dias	Nenhuma_ve	2
Nenhuma_ve	Varios_dias	5
Mais_da_me	Nenhuma_ve	6
Varios_dias	Nenhuma_ve	6
Quase_todos	Nenhuma_ve	9
Varios_dias	Mais_da_me	7
Mais_da_me	Varios_dias	15
Varios_dias	Mais_da_me	11
Varios_dias	Mais_da_me	12
Nenhuma_ve	Nenhuma_ve	3
Nenhuma_ve	Varios_dias	2
Nenhuma_ve	Varios_dias	7
Quase_todos	Varios_dias	9
Nenhuma_ve	Nenhuma_ve	3
Mais_da_me	Nenhuma_ve	11
Varios_dias	Varios_dias	10
Nenhuma_ve	Varios_dias	1
Nenhuma_ve	Nenhuma_ve	0
Quase_todos	Nenhuma_ve	15
Varios_dias	Quase_todos	13
Varios_dias	Nenhuma_ve	7
Nenhuma_ve	Nenhuma_ve	0
Quase_todos	Quase_todos	20
Varios_dias	Varios_dias	7
Varios_dias	Quase_todos	14
Quase_todos	Quase_todos	20
Varios_dias	Varios_dias	4
Varios_dias	Varios_dias	5
Varios_dias	Nenhuma_ve	6
Nenhuma_ve	Nenhuma_ve	1
Mais_da_me	Quase_todos	17
Mais_da_me	Mais_da_me	15
Nenhuma_ve	Nenhuma_ve	5
Varios_dias	Varios_dias	14
Varios_dias	Varios_dias	6
Quase_todos	Quase_todos	18
Varios_dias	Mais_da_me	13
Varios_dias	Nenhuma_ve	11
Mais_da_me	Mais_da_me	12
Varios_dias	Varios_dias	6
Mais_da_me	Mais_da_me	16
Varios_dias	Varios_dias	8
Nenhuma_ve	Nenhuma_ve	2
Mais_da_me	Nenhuma_ve	11
Quase_todos	Mais_da_me	17
Varios_dias	Nenhuma_ve	8
Quase_todos	Quase_todos	21
Quase_todos	Quase_todos	21
Varios_dias	Mais_da_me	7
Mais_da_me	Quase_todos	17
Quase_todos	Quase_todos	21
Quase_todos	Quase_todos	21
Varios_dias	Varios_dias	7

Quase_todos	Mais_da_me	19
Varios_dias	Nenhuma_ve	9
Varios_dias	Varios_dias	15
Quase_todos	Quase_todos	17
Nenhuma_ve	Nenhuma_ve	3
Varios_dias	Varios_dias	6
Varios_dias	Nenhuma_ve	2
Quase_todos	Nenhuma_ve	11
Varios_dias	Varios_dias	6
Varios_dias	Varios_dias	9
Varios_dias	Nenhuma_ve	7
Mais_da_me	Mais_da_me	9
Nenhuma_ve	Varios_dias	7
Varios_dias	Nenhuma_ve	9
Varios_dias	Nenhuma_ve	4
Varios_dias	Varios_dias	12
Nenhuma_ve	Varios_dias	2
Quase_todos	Quase_todos	13
Quase_todos	Mais_da_me	15
Quase_todos	Quase_todos	21
Mais_da_me	Varios_dias	9
Nenhuma_ve	Varios_dias	4
Varios_dias	Nenhuma_ve	4
Varios_dias	Nenhuma_ve	15
Varios_dias	Nenhuma_ve	6
Quase_todos	Quase_todos	21
Varios_dias	Varios_dias	12
Quase_todos	Varios_dias	16
Nenhuma_ve	Nenhuma_ve	2
Mais_da_me	Mais_da_me	17
Mais_da_me	Quase_todos	12
Nenhuma_ve	Varios_dias	2
Varios_dias	Quase_todos	12
Varios_dias	Nenhuma_ve	5
Nenhuma_ve	Mais_da_me	3
Nenhuma_ve	Nenhuma_ve	4
Varios_dias	Varios_dias	8
Mais_da_me	Quase_todos	13
Varios_dias	Nenhuma_ve	5
Nenhuma_ve	Varios_dias	5
Varios_dias	Nenhuma_ve	4
Mais_da_me	Mais_da_me	12
Mais_da_me	Varios_dias	13
Varios_dias	Nenhuma_ve	2
Varios_dias	Varios_dias	8
Quase_todos	Quase_todos	16
Varios_dias	Varios_dias	10
Quase_todos	Mais_da_me	17
Quase_todos	Quase_todos	21
Quase_todos	Mais_da_me	16
Mais_da_me	Varios_dias	15
Varios_dias	Varios_dias	5
Nenhuma_ve	Nenhuma_ve	1
Varios_dias	Nenhuma_ve	5
Quase_todos	Varios_dias	9
Quase_todos	Quase_todos	20
Nenhuma_ve	Varios_dias	4
Nenhuma_ve	Varios_dias	8

Nenhuma_ve	Nenhuma_ve	4
Varios_dias	Nenhuma_ve	5
Varios_dias	Nenhuma_ve	6
Varios_dias	Nenhuma_ve	4
Quase_todos	Varios_dias	8
Varios_dias	Varios_dias	11
Varios_dias	Nenhuma_ve	2
Nenhuma_ve	Nenhuma_ve	1
Varios_dias	Varios_dias	7
Varios_dias	Quase_todos	15
Quase_todos	Quase_todos	21
Varios_dias	Varios_dias	6
Varios_dias	Varios_dias	12
Varios_dias	Nenhuma_ve	3
Varios_dias	Varios_dias	6
Varios_dias	Varios_dias	7
Varios_dias	Mais_da_me	14
Mais_da_me	Mais_da_me	8
Quase_todos	Quase_todos	18
Varios_dias	Varios_dias	5
Varios_dias	Nenhuma_ve	9
Varios_dias	Varios_dias	6
Quase_todos	Quase_todos	19
Nenhuma_ve	Nenhuma_ve	1
Mais_da_me	Varios_dias	7
Quase_todos	Mais_da_me	13
Varios_dias	Quase_todos	8
Nenhuma_ve	Nenhuma_ve	2
Varios_dias	Nenhuma_ve	3
Quase_todos	Varios_dias	12
Nenhuma_ve	Nenhuma_ve	4
Nenhuma_ve	Nenhuma_ve	0
Quase_todos	Quase_todos	21
Varios_dias	Nenhuma_ve	6
Varios_dias	Varios_dias	9
Varios_dias	Varios_dias	15
Mais_da_me	Quase_todos	17
Varios_dias	Nenhuma_ve	4
Varios_dias	Varios_dias	11
Mais_da_me	Quase_todos	10
Nenhuma_ve	Varios_dias	5
Varios_dias	Nenhuma_ve	5
Quase_todos	Nenhuma_ve	15
Varios_dias	Varios_dias	7
Varios_dias	Nenhuma_ve	2
Nenhuma_ve	Quase_todos	18
Mais_da_me	Nenhuma_ve	11
Varios_dias	Varios_dias	7
Varios_dias	Varios_dias	7
Varios_dias	Varios_dias	6
Varios_dias	Varios_dias	7
Varios_dias	Nenhuma_ve	9
Quase_todos	Quase_todos	20
Varios_dias	Nenhuma_ve	4
Quase_todos	Quase_todos	21
Quase_todos	Mais_da_me	16
Quase_todos	Varios_dias	13
Mais_da_me	Varios_dias	16

Varios_dias	Quase_todos	17
Varios_dias	Varios_dias	14
Varios_dias	Quase_todos	10
Varios_dias	Nenhuma_ve	6
Varios_dias	Varios_dias	7
Quase_todos	Nenhuma_ve	11
Quase_todos	Quase_todos	17
Mais_da_me	Mais_da_me	9
Quase_todos	Quase_todos	19
Varios_dias	Quase_todos	12
Mais_da_me	Nenhuma_ve	10
Quase_todos	Quase_todos	21
Varios_dias	Varios_dias	7
Nenhuma_ve	Nenhuma_ve	4
Mais_da_me	Varios_dias	6
Varios_dias	Mais_da_me	12
Varios_dias	Mais_da_me	12
Quase_todos	Quase_todos	20
Varios_dias	Varios_dias	15
Mais_da_me	Quase_todos	20
Varios_dias	Varios_dias	7
Nenhuma_ve	Nenhuma_ve	0
Varios_dias	Nenhuma_ve	12
Quase_todos	Quase_todos	21
Mais_da_me	Quase_todos	16
Quase_todos	Varios_dias	15
Varios_dias	Nenhuma_ve	5
Quase_todos	Quase_todos	21
Varios_dias	Varios_dias	10
Varios_dias	Nenhuma_ve	7
Varios_dias	Varios_dias	7
Mais_da_me	Quase_todos	15
Varios_dias	Varios_dias	10
Quase_todos	Quase_todos	17
Nenhuma_ve	Nenhuma_ve	0
Varios_dias	Varios_dias	6
Varios_dias	Mais_da_me	7
Quase_todos	Nenhuma_ve	8
Nenhuma_ve	Nenhuma_ve	5
Varios_dias	Nenhuma_ve	5
Varios_dias	Varios_dias	6
Varios_dias	Nenhuma_ve	5
Varios_dias	Nenhuma_ve	2
Varios_dias	Varios_dias	6
Varios_dias	Varios_dias	13
Varios_dias	Varios_dias	8
Varios_dias	Nenhuma_ve	2
Varios_dias	Varios_dias	7
Varios_dias	Nenhuma_ve	5
Nenhuma_ve	Nenhuma_ve	0
Varios_dias	Varios_dias	5
Quase_todos	Mais_da_me	16
Mais_da_me	Varios_dias	12
Nenhuma_ve	Nenhuma_ve	0
Varios_dias	Varios_dias	13
Varios_dias	Varios_dias	11
Varios_dias	Varios_dias	7
Varios_dias	Nenhuma_ve	4

Mais_da_me	Varios_dias	15
Varios_dias	Varios_dias	5
Mais_da_me	Varios_dias	10
Nenhuma_ve	Varios_dias	2
Varios_dias	Varios_dias	7
Quase_todos	Quase_todos	18
Varios_dias	Varios_dias	11
Varios_dias	Nenhuma_ve	5
Nenhuma_ve	Varios_dias	5
Varios_dias	Varios_dias	6
Varios_dias	Varios_dias	7
Mais_da_me	Nenhuma_ve	10
Nenhuma_ve	Nenhuma_ve	0
Nenhuma_ve	Nenhuma_ve	0
Mais_da_me	Nenhuma_ve	9
Varios_dias	Varios_dias	9
Varios_dias	Varios_dias	6
Nenhuma_ve	Nenhuma_ve	0
Quase_todos	Quase_todos	19
Quase_todos	Varios_dias	17
Mais_da_me	Nenhuma_ve	9
Quase_todos	Quase_todos	21
Quase_todos	Quase_todos	20
Varios_dias	Nenhuma_ve	5
Varios_dias	Nenhuma_ve	6
Varios_dias	Nenhuma_ve	8
Varios_dias	Nenhuma_ve	2
Nenhuma_ve	Varios_dias	4
Mais_da_me	Varios_dias	10
Varios_dias	Nenhuma_ve	6
Nenhuma_ve	Nenhuma_ve	3
Nenhuma_ve	Nenhuma_ve	1
Nenhuma_ve	Nenhuma_ve	1
Nenhuma_ve	Nenhuma_ve	2
Varios_dias	Nenhuma_ve	4
Varios_dias	Nenhuma_ve	3
Quase_todos	Mais_da_me	18
Varios_dias	Varios_dias	6
Nenhuma_ve	Varios_dias	6
Varios_dias	Varios_dias	7
Nenhuma_ve	Nenhuma_ve	2

Todos os direitos reservados.

#Portuguese#

Título: Dados Brutos de Dor Crônica e Fatores Associados ao Teletrabalho Durante a Pandemia da COVID-19 no Brasil.
Descritores: Dor Crônica; Qualidade do Sono; Ansiedade; Teletrabalho; COVID-19.

Autora principal: Brenda Alves Silvestre.

Instituição: Universidade Federal de São Carlos.

e-mail: brenda.asilvestre@hotmail.com

Coleta de dados realizada no período de fevereiro de 2021 a janeiro de 2022.

Os dados brutos compartilhados no arquivo 'dados_de_dor_cronica_e_fatores_associados_ao_teletrabalho.xlsx' foram obtidos em questionários que foram respondidos de forma online, através de entrevistas realizadas durante o desenvolvimento do trabalho "Dor e fatores associados em adultos em trabalho remoto no contexto da pandemia da COVID-19 no Brasil", que pode ser encontrado no link https://repositorio.ufscar.br/handle/ufscar/17026 e que servem de material suplementar ao artigo "Dor Crônica e Fatores Associados ao Teletrabalho Durante a Pandemia da COVID-19 no Brasil". Os dados estão em sua língua original (português) e foram trabalhados através de estatística descritiva para caracterização da amostra. As variáveis elencadas nesta base de dados foram idade, cidade, estado, região, sexo, cor da pele, estado civil, escolaridade, ocupação/profissão, renda familiar antes da pandemia, filhos, com quem mora, prática de atividades físicas antes e durante a pandemia, hábito de fumar, uso de bebida alcoólica, problemas de saúde, uso de medicamentos, tipo de medicamentos utilizados, uso de medicamentos após inicio da pandemia, período de trabalho remoto durante a pandemia, horas de trabalho por dia durante, tempo sentado por dia (em horas), comportamento social, dor crônica, além de características da dor, do sono, do trabalho remoto e respostas referentes ao Questionário Índice de Qualidade do Sono de Pittsburgh (PSQI) e ao GAD-7. As variáveis categóricas foram apresentadas por frequências bruta e relativa, e para as variáveis contínuas foram calculadas médias e desvio-padrão. Em relação às análises de associação, para estimar a Razão de Prevalência foi utilizado o modelo de regressão de Poisson com variância robusta, simples e múltiplo. Para todas as análises adotou-se um nível de significância de 5% ($p<0{,}05$). O processamento dos dados pode ser acessado no trabalho linkado acima. Todos os entrevistados consentiram virtualmente o uso de suas respostas para o estudo e, seus nomes e formas de contato foram omitidos como forma de preservação de identidade, privacidade e ética.

Todos os dados são compartilhados de forma *open access* e podem ser utilizados desde que os autores sejam devidamente citados e creditados.
